# Supplementary material for: Altered microRNA Transcriptome in Cultured Human Liver Cells upon Infection with Ebola Virus
Source: Int J Mol Sci. 2021 Apr 6;22(7):3792. doi: 10.3390/ijms22073792 (PMC8038836; doi:10.3390/ijms22073792)
Supplement: Supplementary file 1 [file ijms-22-03792-s001.zip › Supplementary_File/C_ GO_Analysis_Results/16-30nt_go_Mayinga-24h-Huh7_vs_Control-24h-Huh7_up.mature_mirna_targets/BP_result(Human).html]

| GO.ID | Term | Ontology | Count | Pop.Hits | List.Total | Pop.Total | Fold.Enrichment | Pvalue | FDR | Enrichment.Score | Gene.Ratio | GENES |
| --- | --- | --- | --- | --- | --- | --- | --- | --- | --- | --- | --- | --- |
| GO:0006357 | regulation of transcription by RNA polymerase II | Biological process | 213 | 2227 | 934 | 17653 | 1.80771945242782 | 5.66255403786867e-19 | 3.82958529581058e-15 | 18.2469876404782 | 0.228051391862955 | TSHZ1//HMG20A//KLF2//ZBTB18//CEBPB//ZNF268//TCERG1//WWP2//KLF12//OSR1//CRY2//GLIS3//ARX//DNMT3B//DR1//E2F1//EDN1//EFNA1//CC2D1B//EN1//ESR1//FHL2//CPEB3//FOXF1//FOXC1//MYT1L//CIC//ZFPM2//DNAJB5//GATA6//CNOT7//HSBP1//AR//JARID2//JUN//ZNF568//MAF//MDM4//MYB//NFIA//NFIB//NR4A2//RLIM//KLF3//ZBTB7A//PIAS4//POU4F1//PPARG//CC2D1A//TSHZ3//ZBTB4//RARB//RBBP7//RBBP8//REST//RNF2//PRDM16//SOX17//SOX11//STAT3//ZEB1//TCF21//TFAP2A//TFAP2C//TGIF1//KLF10//TNF//UBE2I//VLDLR//WFS1//ZNF217//LRP8//BTG2//BHLHE41//TBL1XR1//NR4A3//HMGA2//ARID5B//BRMS1L//BHLHE40//RUNX3//URI1//PER2//SQSTM1//NOG//KLF4//HAND1//FNIP1//PHF14//USP3//KAT2B//THRA//TOB1//SOSTDC1//RGMB//TMEM100//RGMA//BMP3//BMPR2//TGFB2//ACVR1//SULF1//CTDSPL2//ZMYND11//CDK8//KLF6//CREBRF//NKX2-3//E2F3//E2F5//S1PR1//EGR2//ELK3//ERG//ETS1//EZH1//FOXJ3//FOXF2//FLI1//NR5A2//DLL1//ATAD2//GRHL1//H2AFZ//NRG1//IGF1//IL2//ITGA6//IRF1//LRP6//MEF2D//MEOX2//MET//MYBL1//PPP1R12A//NCK1//NEUROD1//NEUROG1//NHLH2//NPAS2//OSM//OTX2//P2RY1//PAX9//PBX3//IER5//ARID4B//PKD2//POU3F2//EGLN1//PPP3R1//DCAF6//RAD21//ARID4A//RNF4//SHOX//BCL11B//CAPRIN2//SOX4//STAT4//TEAD1//TEF//WNT7A//FOSL1//PCGF5//KLF7//CBFB//CCNC//FUBP3//RPS6KA4//RPS6KA5//HAND2//CLOCK//ST18//EGLN3//FBXW7//NPNT//CDKN1A//IRF9//TADA1//CRK//ZNF367//EMX2//EPC2//LHX6//HBP1//GTF2E1//HIVEP2//MNX1//HOXA3//HOXD1//FOXN2//BOLA3//NR3C2//LHX8//NFE2//PLEK//BRWD1//TRERF1//SLC2A4RG//ZNFX1//MIER1//RBL2//DMRTA1//NKX2-4//RFX7//SMARCD2//ZNF3//ZNF131//DEK//BRD3//TCF7L1//LCOR// |
| GO:0006366 | transcription by RNA polymerase II | Biological process | 222 | 2405 | 934 | 17653 | 1.74465491681766 | 5.74228127535964e-18 | 1.94175241326286e-14 | 17.2409155381814 | 0.237687366167024 | TSHZ1//HMG20A//KLF2//ZBTB18//CEBPB//ZNF268//TCERG1//WWP2//KLF12//OSR1//CRY2//GLIS3//ARX//DNMT3B//DR1//E2F1//EDN1//EFNA1//CC2D1B//EN1//ESR1//FHL2//CPEB3//FOXF1//FOXC1//MYT1L//CIC//ZFPM2//DNAJB5//GATA6//CNOT7//HSBP1//AR//JARID2//JUN//ZNF568//MAF//MDM4//MYB//NFIA//NFIB//NR4A2//RLIM//KLF3//ZBTB7A//PIAS4//POU4F1//PPARG//CC2D1A//TSHZ3//ZBTB4//RARB//RBBP7//RBBP8//REST//RNF2//PRDM16//SOX17//SOX11//STAT3//ZEB1//TCF21//TFAP2A//TFAP2C//TGIF1//KLF10//TNF//UBE2I//VLDLR//WFS1//ZNF217//LRP8//BTG2//BHLHE41//TBL1XR1//NR4A3//HMGA2//ARID5B//BRMS1L//BHLHE40//RUNX3//URI1//PER2//SQSTM1//NOG//KLF4//HAND1//FNIP1//PHF14//USP3//GTF2E1//SRSF2//CDKN1A//IRF9//TADA1//CRK//ZNF367//ELK3//EMX2//ERG//ETS1//FOXJ3//FOXF2//FLI1//EPC2//LHX6//HBP1//ATAD2//GRHL1//HIVEP2//MNX1//HOXA3//HOXD1//FOXN2//BOLA3//NR3C2//LHX8//MYBL1//NEUROG1//NFE2//NHLH2//ARID4B//PLEK//BRWD1//POU3F2//TRERF1//SLC2A4RG//ZNFX1//MIER1//RAD21//ARID4A//RBL2//DMRTA1//NKX2-4//RFX7//SMARCD2//TEF//THRA//ZNF3//ZNF131//DEK//BRD3//FOSL1//TCF7L1//LCOR//KLF7//CBFB//CLOCK//CDK8//E2F3//NR5A2//NRBF2//TEAD1//KAT2B//CCNC//TOB1//SOSTDC1//RGMB//TMEM100//RGMA//BMP3//BMPR2//TGFB2//ACVR1//SULF1//CTDSPL2//ZMYND11//INTS8//NABP1//SNAPC1//KLF6//CREBRF//NKX2-3//E2F5//S1PR1//EGR2//EZH1//DLL1//H2AFZ//NRG1//IGF1//IL2//ITGA6//IRF1//LRP6//MEF2D//MEOX2//MET//PPP1R12A//NCK1//NEUROD1//NPAS2//OSM//OTX2//P2RY1//PAX9//PBX3//IER5//PKD2//EGLN1//PPP3R1//DCAF6//RNF4//SHOX//BCL11B//CAPRIN2//SOX4//STAT4//WNT7A//PCGF5//FUBP3//RPS6KA4//RPS6KA5//HAND2//ST18//EGLN3//FBXW7//NPNT//LHX1//BTF3//TGFB1I1//TMF1// |
| GO:0031323 | regulation of cellular metabolic process | Biological process | 463 | 6454 | 934 | 17653 | 1.35588755607963 | 4.94229087498485e-17 | 1.11415710625075e-13 | 16.3060716980915 | 0.495717344753747 | CDKN1A//GADD45A//PTEN//CCNE2//E2F1//NPAT//RRM2//TSHZ1//HMG20A//KLF2//ZBTB18//CEBPB//ZNF268//TCERG1//WWP2//KLF12//OSR1//CRY2//GLIS3//ARX//DNMT3B//DR1//EDN1//EFNA1//CC2D1B//EN1//ESR1//FHL2//CPEB3//FOXF1//FOXC1//MYT1L//CIC//ZFPM2//DNAJB5//GATA6//CNOT7//HSBP1//AR//JARID2//JUN//ZNF568//MAF//MDM4//MYB//NFIA//NFIB//NR4A2//RLIM//KLF3//ZBTB7A//PIAS4//POU4F1//PPARG//CC2D1A//TSHZ3//ZBTB4//RARB//RBBP7//RBBP8//REST//RNF2//PRDM16//SOX17//SOX11//STAT3//ZEB1//TCF21//TFAP2A//TFAP2C//TGIF1//KLF10//TNF//UBE2I//VLDLR//WFS1//ZNF217//LRP8//BTG2//BHLHE41//TBL1XR1//NR4A3//HMGA2//ARID5B//BRMS1L//BHLHE40//RUNX3//URI1//PER2//SQSTM1//NOG//KLF4//HAND1//FNIP1//PHF14//USP3//MINK1//MAP3K2//CRK//CRKL//MAP3K5//TAOK3//LAMTOR3//ADAM9//DUSP5//LPAR1//LPAR3//NRG1//IGF1//NTF3//PIK3CB//PRKAA1//MAPK1//MAP2K4//DUSP10//SPRED1//DUSP1//DUSP2//PPP2CA//RGS3//RBFOX3//FMR1//MBNL1//RBM25//SRSF2//SECISBP2L//EPS15//NSF//DRD1//FAM129A//IL2//LRP6//PPP1R15B//SLIT2//EFNA5//EPHA7//NPTN//KDR//P2RY1//AKTIP//CCND2//PKD2//THRA//EREG//FBXW7//ERRFI1//TSG101//SOCS5//NUP35//NUP43//NLN//ESCO2//BAHD1//H2AFZ//KMT2B//DPY30//HIPK3//KHDRBS3//ZMYND11//ZNF597//CTNND2//ZNF782//ZNF800//ELAVL2//ZBTB9//KDM2A//MYCBP2//FLII//CNOT6L//NR5A2//GTF2E1//SERTAD3//NRBF2//HIVEP2//HMGB3//ZBTB41//ZBTB34//ATXN3//MLLT6//RNF141//BNC2//ZNF532//CDCA7L//LMO3//SLC2A4RG//PAK6//PHTF2//ZNF630//CNOT6//ZNF512B//ZBTB26//BCL11B//SNAPC1//SOX4//BTF3//BTG1//TMF1//ZNF2//ZNF3//ZNF131//ZNF655//SNIP1//TCF7L1//WASL//RPS6KA4//RPS6KA5//ZBTB47//CLOCK//IRF9//TADA1//ZNF367//ELK3//EMX2//ERG//ETS1//FOXJ3//FOXF2//FLI1//EPC2//LHX6//HBP1//ATAD2//GRHL1//MNX1//HOXA3//HOXD1//FOXN2//BOLA3//NR3C2//LHX8//MYBL1//NEUROG1//NFE2//NHLH2//ARID4B//PLEK//BRWD1//POU3F2//TRERF1//ZNFX1//MIER1//RAD21//ARID4A//RBL2//DMRTA1//NKX2-4//RFX7//SMARCD2//TEF//DEK//BRD3//FOSL1//LCOR//KLF7//CBFB//POLR3G//BDP1//MTPN//DDX6//MKNK2//ACO1//TSC1//KBTBD8//PPM1E//FLRT1//GNAQ//NCK1//NF2//ASPN//DUS2//YWHAG//CAMK2N2//PPM1F//DLC1//EGLN3//F3//PMAIP1//KAT2B//COPS8//G3BP2//MDFIC//RRAGD//ULK2//MET//MTMR9//BNIP3//PPP4R2//GRB2//TGFBR2//TGFB2//NPNT//CALM2//GDF11//INHBB//BMP3//BMPR2//ACVR1//CNEP1R1//LDLR//PPP1R37//PPP1R12A//PPP1R12C//PLEKHF2//SERPINI1//SPOCK1//RECK//PCOLCE2//SERP1//PRR16//FXR1//SESN3//RAB12//SESN1//SNX5//RHEB//TOPORS//KLF6//EGR2//MAPRE3//BAMBI//RGMB//IRF1//LHX1//NEUROD1//NPAS2//OTX2//ZNF287//RNF4//TEAD1//TGFB1I1//WNT7A//FUBP3//HAND2//DKK3//PAX9//RASD1//ATXN1//BTAF1//TOB1//NANOS1//CAPRIN2//SOSTDC1//TMEM100//RGMA//SULF1//CTDSPL2//ADRB1//PTHLH//EGLN1//SPOPL//BAG5//NDFIP2//CDK6//RAP2C//CMKLR1//SLK//DCP2//CCT6A//BAG6//RNF180//ANKIB1//LONRF3//LONRF1//MASTL//EPHA5//MYLIP//OSM//PFN2//IFNA1//EPHA2//EPHA4//DSTYK//ADCY3//PRKACB//PRKAR2B//NSMF//RNF128//ADIPOR2//PTGES3//KCTD20//ITGA6//BTBD10//ITM2B//ARL6IP1//BCL2L11//ITGAV//FZD6//FASTK//ESRP1//AHNAK//YTHDF2//FZD10//AIDA//TWF1//PIK3R3//UBXN2B//MID1IP1//LATS2//IPO5//CCNY//CCNC//MMD//ASPH//UGT1A8//CDK8//CREBRF//NKX2-3//E2F3//E2F5//S1PR1//EZH1//DLL1//MEF2D//MEOX2//PBX3//IER5//PPP3R1//DCAF6//SHOX//STAT4//PCGF5//ST18//PAIP2//HPRT1//ITGA5//SH3D19//FAM20C//NOVA1//ARRDC3//ARRDC4//CHRM5//PMEPA1//LDLRAD4//IDH1//QKI//RANBP9//DNAJC27//CCL2//CCL8//ACSL1//PLAA//ORMDL3//CPEB2//CALCOCO2//FYCO1//CRTAP//FEZ2//RAB3GAP1//FBXL5//MSN//TOB2//DERL2//EDEM1//FRY//CTSA//KLHL15//GJA1//NSMAF//CPEB1//CPEB4//UNK//PIKFYVE//TBC1D12// |
| GO:0031325 | positive regulation of cellular metabolic process | Biological process | 262 | 3074 | 934 | 17653 | 1.61090182354179 | 7.69090831902742e-17 | 1.30034032403956e-13 | 16.1140223657062 | 0.28051391862955 | GADD45A//MINK1//TNF//MAP3K2//CRK//CRKL//MAP3K5//TAOK3//LAMTOR3//ADAM9//DUSP5//LPAR1//LPAR3//NRG1//IGF1//NTF3//PIK3CB//PRKAA1//MAPK1//MAP2K4//EPS15//NSF//FAM129A//EFNA1//EFNA5//EPHA7//FMR1//NPTN//KDR//P2RY1//AKTIP//SQSTM1//CCND2//FNIP1//EREG//FBXW7//KMT2B//DLC1//EGLN3//F3//PMAIP1//PPARG//KAT2B//COPS8//MDFIC//BNIP3//CDKN1A//GRB2//TGFBR2//NPNT//CALM2//GDF11//INHBB//BMP3//BMPR2//TGFB2//ACVR1//CNEP1R1//LDLR//EDN1//MTMR9//PLEK//PCOLCE2//CPEB3//SERP1//PRR16//SOX4//FXR1//SESN3//RAB12//SESN1//TSC1//TOPORS//KLF2//KLF6//E2F1//EGR2//ESR1//ETS1//FHL2//MAPRE3//FOXF1//FOXF2//FOXC1//FLI1//NR5A2//BAMBI//GATA6//RGMB//ATAD2//SERTAD3//FOXN2//IRF1//AR//JUN//LHX1//LRP6//MYB//MYBL1//NEUROD1//NFE2//NPAS2//NPAT//OTX2//TRERF1//ZNF287//REST//RNF4//PRDM16//SOX17//SOX11//STAT3//TEAD1//TFAP2A//TGFB1I1//WNT7A//TBL1XR1//HMGA2//RUNX3//FUBP3//KLF4//HAND2//CLOCK//ADRB1//PTHLH//NDFIP2//WFS1//PKD2//RAP2C//PAK6//SLK//CCT6A//RNF180//ANKIB1//LONRF3//LONRF1//SOCS5//PPP1R15B//RPS6KA4//RPS6KA5//OSM//PFN2//CAPRIN2//IFNA1//PIAS4//EPHA2//EPHA4//EPHA5//DSTYK//MET//ADCY3//PRKACB//PRKAR2B//NSMF//PPP1R12A//NCK1//PTGES3//KCTD20//ITGA6//BTBD10//IL2//BCL2L11//PPM1F//ITGAV//FZD10//MID1IP1//CCNY//CCNC//MMD//VLDLR//ASPH//CDK8//ZBTB18//CEBPB//ZNF268//WWP2//OSR1//CREBRF//NKX2-3//GLIS3//E2F3//E2F5//S1PR1//ELK3//EN1//ERG//EZH1//FOXJ3//ZFPM2//DLL1//GRHL1//CNOT7//H2AFZ//MAF//MEF2D//MEOX2//NEUROG1//NFIA//NFIB//NHLH2//NR4A2//PAX9//PBX3//IER5//ARID4B//POU3F2//POU4F1//EGLN1//PPP3R1//DCAF6//RGMA//RAD21//RARB//ARID4A//SHOX//BCL11B//STAT4//ZEB1//TCF21//TEF//TFAP2C//THRA//KLF10//NR4A3//FOSL1//PCGF5//KLF7//CBFB//NOG//HAND1//ST18//HPRT1//ITGA5//LRP8//SH3D19//ARRDC3//ARRDC4//DNMT3B//JARID2//TOB1//BTG2//CNOT6L//YTHDF2//QKI//DNAJC27//PTEN//CCL2//CCL8//ACSL1//PPP2CA//NANOS1//CALCOCO2//FYCO1//RAB3GAP1//FBXL5//MSN//UBE2I//EDEM1//ATXN3//BAG6//RNF128//NSMAF// |
| GO:0051173 | positive regulation of nitrogen compound metabolic process | Biological process | 252 | 2965 | 934 | 17653 | 1.60637703976803 | 5.69602710205209e-16 | 7.70444625823566e-13 | 15.2444279529661 | 0.269807280513919 | GADD45A//MINK1//TNF//MAP3K2//CRK//CRKL//MAP3K5//TAOK3//LAMTOR3//ADAM9//DUSP5//LPAR1//LPAR3//NRG1//IGF1//NTF3//PIK3CB//PRKAA1//MAPK1//MAP2K4//FAM129A//EFNA1//EFNA5//EPHA7//FMR1//NPTN//KDR//P2RY1//AKTIP//SQSTM1//CCND2//FNIP1//EREG//FBXW7//KMT2B//DLC1//EGLN3//F3//PMAIP1//PPARG//KAT2B//COPS8//MDFIC//CALM2//GDF11//INHBB//BMP3//BMPR2//TGFB2//ACVR1//PCOLCE2//CPEB3//SERP1//PRR16//SOX4//FXR1//TOPORS//KLF2//KLF6//E2F1//EGR2//ESR1//ETS1//FHL2//MAPRE3//FOXF1//FOXF2//FOXC1//FLI1//NR5A2//BAMBI//GATA6//RGMB//ATAD2//SERTAD3//FOXN2//IRF1//AR//JUN//LHX1//LRP6//MYB//MYBL1//NEUROD1//NFE2//NPAS2//NPAT//OTX2//TRERF1//ZNF287//REST//RNF4//PRDM16//SOX17//SOX11//STAT3//TEAD1//TFAP2A//TGFB1I1//WNT7A//TBL1XR1//HMGA2//RUNX3//FUBP3//KLF4//HAND2//CLOCK//ADRB1//PTHLH//NDFIP2//WFS1//PKD2//RAP2C//PAK6//TGFBR2//SLK//CCT6A//RNF180//ANKIB1//LONRF3//LONRF1//SOCS5//PPP1R15B//RPS6KA4//RPS6KA5//OSM//PFN2//CAPRIN2//IFNA1//PIAS4//ADCY3//PRKACB//PRKAR2B//CNEP1R1//NSMF//PPP1R12A//NCK1//IL2//BCL2L11//PPM1F//EDN1//EPHA2//EPHA4//EPHA5//ITGAV//MET//FZD10//WWP2//GJA1//MYLIP//NSF//HECW2//CCNY//CCNC//CDKN1A//MMD//VLDLR//ASPH//CDK8//ZBTB18//CEBPB//ZNF268//OSR1//CREBRF//NKX2-3//GLIS3//E2F3//E2F5//S1PR1//ELK3//EN1//ERG//EZH1//FOXJ3//ZFPM2//DLL1//GRHL1//CNOT7//H2AFZ//ITGA6//MAF//MEF2D//MEOX2//NEUROG1//NFIA//NFIB//NHLH2//NR4A2//PAX9//PBX3//IER5//ARID4B//POU3F2//POU4F1//EGLN1//PPP3R1//DCAF6//RGMA//RAD21//RARB//ARID4A//SHOX//BCL11B//STAT4//ZEB1//TCF21//TEF//TFAP2C//THRA//KLF10//NR4A3//FOSL1//PCGF5//KLF7//CBFB//NOG//HAND1//ST18//HPRT1//ITGA5//LRP8//SH3D19//MTPN//ARRDC3//ARRDC4//DNMT3B//JARID2//PTGES3//TOB1//BTG2//CNOT6L//YTHDF2//QKI//NPNT//DSTYK//DNAJC27//PTEN//CCL2//CCL8//ACSL1//PPP2CA//NANOS1//RAB3GAP1//FBXL5//MSN//UBE2I//EDEM1//ATXN3//BAG6//RNF128//LDLR//NSMAF// |
| GO:0009893 | positive regulation of metabolic process | Biological process | 273 | 3329 | 934 | 17653 | 1.54996002297634 | 2.02168201252166e-15 | 2.278772575114e-12 | 14.6942871529326 | 0.292291220556745 | GADD45A//MINK1//TNF//MAP3K2//CRK//CRKL//MAP3K5//TAOK3//LAMTOR3//ADAM9//DUSP5//LPAR1//LPAR3//NRG1//IGF1//NTF3//PIK3CB//PRKAA1//MAPK1//MAP2K4//EPS15//NSF//FAM129A//EFNA1//EFNA5//EPHA7//FMR1//NPTN//KDR//P2RY1//AKTIP//SQSTM1//CCND2//FNIP1//EREG//FBXW7//KMT2B//DLC1//EGLN3//F3//PMAIP1//PPARG//KAT2B//COPS8//MDFIC//BNIP3//MTPN//OSR1//ARX//DNMT3B//DRD1//E2F1//ETS1//RAB3GAP1//GJA1//AR//ITGB8//LDLR//MSN//POU4F1//PTEN//SOX11//STAT3//ACTC1//TFAP2A//HMGA2//FUBP3//ZNF804A//NOG//KLF4//QKI//HAND2//PPM1F//CDKN1A//GRB2//TGFBR2//NPNT//CALM2//GDF11//INHBB//BMP3//BMPR2//TGFB2//ACVR1//CNEP1R1//EDN1//MTMR9//PLEK//PCOLCE2//CPEB3//SERP1//PRR16//SOX4//FXR1//SESN3//RAB12//SESN1//TSC1//TOPORS//KLF2//KLF6//EGR2//ESR1//FHL2//MAPRE3//FOXF1//FOXF2//FOXC1//FLI1//NR5A2//BAMBI//GATA6//RGMB//ATAD2//SERTAD3//FOXN2//IRF1//JUN//LHX1//LRP6//MYB//MYBL1//NEUROD1//NFE2//NPAS2//NPAT//OTX2//TRERF1//ZNF287//REST//RNF4//PRDM16//SOX17//TEAD1//TGFB1I1//WNT7A//TBL1XR1//RUNX3//CLOCK//ADRB1//PTHLH//NDFIP2//WFS1//PKD2//RAP2C//PAK6//SLK//CCT6A//RNF180//ANKIB1//LONRF3//LONRF1//SOCS5//PPP1R15B//RPS6KA4//RPS6KA5//OSM//PFN2//CAPRIN2//IFNA1//PIAS4//EPHA2//EPHA4//EPHA5//DSTYK//MET//ADCY3//PRKACB//PRKAR2B//NSMF//PPP1R12A//NCK1//PTGES3//KCTD20//ITGA6//BTBD10//IL2//BCL2L11//ITGAV//FZD10//MID1IP1//WWP2//MYLIP//HECW2//CCNY//CCNC//DEK//MMD//VLDLR//ASPH//CDK8//ZBTB18//CEBPB//ZNF268//CREBRF//NKX2-3//GLIS3//E2F3//E2F5//S1PR1//ELK3//EN1//ERG//EZH1//FOXJ3//ZFPM2//DLL1//GRHL1//CNOT7//H2AFZ//MAF//MEF2D//MEOX2//NEUROG1//NFIA//NFIB//NHLH2//NR4A2//PAX9//PBX3//IER5//ARID4B//POU3F2//EGLN1//PPP3R1//DCAF6//RGMA//RAD21//RARB//ARID4A//SHOX//BCL11B//STAT4//ZEB1//TCF21//TEF//TFAP2C//THRA//KLF10//NR4A3//FOSL1//PCGF5//KLF7//CBFB//HAND1//ST18//HPRT1//ITGA5//LRP8//SH3D19//ARRDC3//ARRDC4//JARID2//CCL2//TOB1//BTG2//CNOT6L//YTHDF2//DNAJC27//CCL8//ACSL1//PPP2CA//NANOS1//CALCOCO2//FYCO1//FBXL5//UBE2I//EDEM1//ATXN3//BAG6//RNF128//NSMAF//PIGA// |
| GO:0048468 | cell development | Biological process | 189 | 2045 | 934 | 17653 | 1.74678774678932 | 3.2747450070964e-15 | 3.16387149757042e-12 | 14.4848225113432 | 0.202355460385439 | EREG//YTHDF2//TBC1D20//AGFG1//TMF1//SEMA4B//ACVR1//LATS2//SOX17//CLIC4//MET//SULF1//BMPR2//ESR1//RARB//GJA1//PTHLH//HAND2//CDK6//DLL1//PALLD//BCL11B//AR//FRMD6//TGFBR2//DZIP1//RNF2//TDRD5//QKI//NTNG1//SLITRK3//NEFH//OGN//POU4F1//DSCAML1//WNT7A//SLIT2//RANBP9//ARX//DPYSL2//EFNA1//EFNA5//EPHA5//GAB1//GRB2//KIF5A//OTX2//PIK3CB//ENAH//MAPK1//NTN4//SOS1//VLDLR//NR4A3//KLF7//RPS6KA5//FEZ2//EGR2//EPHA4//LHX1//NOG//KMT2B//FRZB//PTEN//SNAP25//ZNF804A//STMN2//NPTN//NCK1//NME1//SERPINI1//TWF1//LPAR1//MYLIP//PTPRG//SPOCK1//TSC1//BAG5//DRD1//POU3F2//WASF3//SOX11//NF2//FOXC1//RDH10//EDN1//NRG1//KBTBD8//IGF1//GNAQ//FLRT1//STRN//SOX4//LHX6//LHX8//NR4A2//HPRT1//BTG2//NFIB//KCNJ10//PPP3R1//TMOD3//TMOD1//ACTC1//RAB21//JUN//FRY//GPM6A//LRP12//PSD//EPHA7//ITGAV//SYCP3//TOPORS//FLI1//FAM20C//KLF2//CFL2//NTF3//YWHAG//HMG20A//DDX6//REST//DNMT3B//MMD//NEUROD1//NEUROG1//ZEB1//GOLGA4//GDF11//PPARG//CBFB//INHBB//EN1//MYT1L//FMR1//PBX3//TGFB2//FXR1//UNK//ULK2//LPAR3//KLF4//RGMA//TNFRSF21//RHEB//DUSP10//MNX1//MINK1//NSMF//HECW2//ARID4A//RUNX3//PER2//MAN2A1//CAPRIN2//FHL2//MYB//CDKN1A//SLC12A5//CTNND2//WASL//CPEB3//IL2//C21ORF91//LRP8//MSN//RAP1B//MAP2K4//NEFM//LDLR//EPHA2//OSR1//RAP2C//ARID4B//NANOS1//CRK//CRKL//DOCK5//TNF//PLAA//CLOCK//FBXW7//STAT3//GJC1//GATA6//MAF//BMP3//ARID5B// |
| GO:0080090 | regulation of primary metabolic process | Biological process | 450 | 6394 | 934 | 17653 | 1.33018340936598 | 1.00172973734708e-14 | 8.46837276709788e-12 | 13.9992494335694 | 0.481798715203426 | CDKN1A//GADD45A//PTEN//CCNE2//E2F1//NPAT//RRM2//TSHZ1//HMG20A//KLF2//ZBTB18//CEBPB//ZNF268//TCERG1//WWP2//KLF12//OSR1//CRY2//GLIS3//ARX//DNMT3B//DR1//EDN1//EFNA1//CC2D1B//EN1//ESR1//FHL2//CPEB3//FOXF1//FOXC1//MYT1L//CIC//ZFPM2//DNAJB5//GATA6//CNOT7//HSBP1//AR//JARID2//JUN//ZNF568//MAF//MDM4//MYB//NFIA//NFIB//NR4A2//RLIM//KLF3//ZBTB7A//PIAS4//POU4F1//PPARG//CC2D1A//TSHZ3//ZBTB4//RARB//RBBP7//RBBP8//REST//RNF2//PRDM16//SOX17//SOX11//STAT3//ZEB1//TCF21//TFAP2A//TFAP2C//TGIF1//KLF10//TNF//UBE2I//VLDLR//WFS1//ZNF217//LRP8//BTG2//BHLHE41//TBL1XR1//NR4A3//HMGA2//ARID5B//BRMS1L//BHLHE40//RUNX3//URI1//PER2//SQSTM1//NOG//KLF4//HAND1//FNIP1//PHF14//USP3//MINK1//MAP3K2//CRK//CRKL//MAP3K5//TAOK3//LAMTOR3//ADAM9//DUSP5//LPAR1//LPAR3//NRG1//IGF1//NTF3//PIK3CB//PRKAA1//MAPK1//MAP2K4//DUSP10//SPRED1//DUSP1//DUSP2//PPP2CA//RGS3//RBFOX3//FMR1//MBNL1//RBM25//SRSF2//SECISBP2L//DRD1//FAM129A//IL2//LRP6//PPP1R15B//SLIT2//EFNA5//EPHA7//NPTN//KDR//P2RY1//AKTIP//CCND2//PKD2//THRA//EREG//FBXW7//ERRFI1//TSG101//SOCS5//NUP35//NUP43//NLN//ESCO2//BAHD1//H2AFZ//KMT2B//DPY30//HIPK3//KHDRBS3//ZMYND11//ZNF597//CTNND2//ZNF782//ZNF800//ELAVL2//ZBTB9//KDM2A//MYCBP2//FLII//CNOT6L//NR5A2//GTF2E1//SERTAD3//NRBF2//HIVEP2//HMGB3//ZBTB41//ZBTB34//ATXN3//MLLT6//RNF141//BNC2//ZNF532//CDCA7L//LMO3//SLC2A4RG//PAK6//PHTF2//ZNF630//CNOT6//ZNF512B//ZBTB26//BCL11B//SNAPC1//SOX4//BTF3//BTG1//TMF1//ZNF2//ZNF3//ZNF131//ZNF655//SNIP1//TCF7L1//WASL//RPS6KA4//RPS6KA5//ZBTB47//CLOCK//IRF9//TADA1//ZNF367//ELK3//EMX2//ERG//ETS1//FOXJ3//FOXF2//FLI1//EPC2//LHX6//HBP1//ATAD2//GRHL1//MNX1//HOXA3//HOXD1//FOXN2//BOLA3//NR3C2//LHX8//MYBL1//NEUROG1//NFE2//NHLH2//ARID4B//PLEK//BRWD1//POU3F2//TRERF1//ZNFX1//MIER1//RAD21//ARID4A//RBL2//DMRTA1//NKX2-4//RFX7//SMARCD2//TEF//DEK//BRD3//FOSL1//LCOR//KLF7//CBFB//POLR3G//BDP1//MTPN//DDX6//MKNK2//ACO1//TSC1//KBTBD8//PPM1E//FLRT1//GNAQ//NCK1//NF2//ASPN//DUS2//YWHAG//CAMK2N2//PPM1F//DLC1//EGLN3//F3//PMAIP1//KAT2B//COPS8//G3BP2//MDFIC//PPP4R2//CALM2//GDF11//INHBB//BMP3//BMPR2//TGFB2//ACVR1//CNEP1R1//LDLR//SERPINI1//SPOCK1//RECK//PCOLCE2//SERP1//PRR16//FXR1//TOPORS//KLF6//EGR2//MAPRE3//BAMBI//RGMB//IRF1//LHX1//NEUROD1//NPAS2//OTX2//ZNF287//RNF4//TEAD1//TGFB1I1//WNT7A//FUBP3//HAND2//DKK3//PAX9//RASD1//ATXN1//BTAF1//TOB1//NANOS1//CAPRIN2//ACSL1//RGL1//TNFRSF21//ACOX1//UGT1A9//SPOPL//SOSTDC1//TMEM100//RGMA//SULF1//CTDSPL2//ADRB1//PTHLH//EGLN1//BAG5//NDFIP2//CDK6//RAP2C//CMKLR1//TGFBR2//SLK//DCP2//CCT6A//BAG6//RNF180//ANKIB1//LONRF3//LONRF1//PPP1R37//MASTL//EPHA5//MYLIP//OSM//PFN2//IFNA1//ADCY3//PRKACB//PRKAR2B//NSMF//PPP1R12A//RNF128//ADIPOR2//ITM2B//ARL6IP1//BCL2L11//GRB2//EPHA2//EPHA4//ITGAV//MET//FZD6//FASTK//ESRP1//AHNAK//YTHDF2//FZD10//AIDA//TWF1//PIK3R3//UBXN2B//MID1IP1//GJA1//NSF//HECW2//LATS2//IPO5//CCNY//CCNC//MMD//ASPH//UGT1A8//UGT1A1//CDK8//CREBRF//NKX2-3//E2F3//E2F5//S1PR1//EZH1//DLL1//ITGA6//MEF2D//MEOX2//PBX3//IER5//PPP3R1//DCAF6//SHOX//STAT4//PCGF5//ST18//PAIP2//HPRT1//ITGA5//SH3D19//NOVA1//ARRDC3//ARRDC4//PTGES3//CHRM5//MTMR9//PMEPA1//LDLRAD4//IDH1//QKI//RANBP9//NPNT//DSTYK//DNAJC27//CCL2//CCL8//PLAA//ORMDL3//CPEB2//CRTAP//RAB3GAP1//FBXL5//MSN//TOB2//DERL2//EDEM1//FRY//CTSA//KLHL15//NSMAF//CPEB1//CPEB4//UNK// |
| GO:0019222 | regulation of metabolic process | Biological process | 478 | 6920 | 934 | 17653 | 1.30554981371687 | 1.72697305072233e-14 | 1.29772430467057e-11 | 13.7627144395107 | 0.511777301927195 | CDKN1A//GADD45A//PTEN//CCNE2//E2F1//NPAT//RRM2//TSHZ1//HMG20A//KLF2//ZBTB18//CEBPB//ZNF268//TCERG1//WWP2//KLF12//OSR1//CRY2//GLIS3//ARX//DNMT3B//DR1//EDN1//EFNA1//CC2D1B//EN1//ESR1//FHL2//CPEB3//FOXF1//FOXC1//MYT1L//CIC//ZFPM2//DNAJB5//GATA6//CNOT7//HSBP1//AR//JARID2//JUN//ZNF568//MAF//MDM4//MYB//NFIA//NFIB//NR4A2//RLIM//KLF3//ZBTB7A//PIAS4//POU4F1//PPARG//CC2D1A//TSHZ3//ZBTB4//RARB//RBBP7//RBBP8//REST//RNF2//PRDM16//SOX17//SOX11//STAT3//ZEB1//TCF21//TFAP2A//TFAP2C//TGIF1//KLF10//TNF//UBE2I//VLDLR//WFS1//ZNF217//LRP8//BTG2//BHLHE41//TBL1XR1//NR4A3//HMGA2//ARID5B//BRMS1L//BHLHE40//RUNX3//URI1//PER2//SQSTM1//NOG//KLF4//HAND1//FNIP1//PHF14//USP3//DCP2//PPP2CA//RPL15//MINK1//MAP3K2//CRK//CRKL//MAP3K5//TAOK3//LAMTOR3//ADAM9//DUSP5//LPAR1//LPAR3//NRG1//IGF1//NTF3//PIK3CB//PRKAA1//MAPK1//MAP2K4//DUSP10//SPRED1//DUSP1//DUSP2//RGS3//CNOT6L//PAN3//CNOT6//RBFOX3//FMR1//MBNL1//RBM25//SRSF2//SECISBP2L//EPS15//NSF//DRD1//FAM129A//IL2//LRP6//PPP1R15B//SLIT2//EFNA5//EPHA7//NPTN//KDR//P2RY1//AKTIP//CCND2//PKD2//THRA//EREG//FBXW7//ERRFI1//TSG101//SOCS5//NUP35//NUP43//NLN//ESCO2//BAHD1//H2AFZ//KMT2B//DPY30//ARID4B//ARID4A//HIPK3//KHDRBS3//ZMYND11//ZNF597//CTNND2//ZNF782//ZNF800//ELAVL2//ZBTB9//KDM2A//MYCBP2//FLII//NR5A2//GTF2E1//SERTAD3//NRBF2//HIVEP2//HMGB3//ZBTB41//ZBTB34//ATXN3//MLLT6//RNF141//BNC2//ZNF532//CDCA7L//LMO3//SLC2A4RG//PAK6//PHTF2//ZNF630//ZNF512B//ZBTB26//BCL11B//SNAPC1//SOX4//BTF3//BTG1//TMF1//ZNF2//ZNF3//ZNF131//ZNF655//SNIP1//TCF7L1//WASL//RPS6KA4//RPS6KA5//ZBTB47//CLOCK//IRF9//TADA1//ZNF367//ELK3//EMX2//ERG//ETS1//FOXJ3//FOXF2//FLI1//EPC2//LHX6//HBP1//ATAD2//GRHL1//MNX1//HOXA3//HOXD1//FOXN2//BOLA3//NR3C2//LHX8//MYBL1//NEUROG1//NFE2//NHLH2//PLEK//BRWD1//POU3F2//TRERF1//ZNFX1//MIER1//RAD21//RBL2//DMRTA1//NKX2-4//RFX7//SMARCD2//TEF//DEK//BRD3//FOSL1//LCOR//KLF7//CBFB//POLR3G//BDP1//MTPN//DDX6//MKNK2//ACO1//TSC1//KBTBD8//PPM1E//FLRT1//GNAQ//NCK1//NF2//ASPN//DUS2//YWHAG//CAMK2N2//PPM1F//DLC1//EGLN3//F3//PMAIP1//KAT2B//COPS8//G3BP2//MDFIC//PIGA//TOB1//CDK6//TOB2//LHX1//PTHLH//TGFBR2//RRAGD//ULK2//MET//MTMR9//BNIP3//PPP4R2//NANOS1//RAB3GAP1//GJA1//ITGB8//LDLR//MSN//ACTC1//FUBP3//ZNF804A//QKI//HAND2//NME1//NDFIP2//TGFB2//GRB2//NPNT//CALM2//GDF11//INHBB//BMP3//BMPR2//ACVR1//CNEP1R1//PPP1R37//PPP1R12A//PPP1R12C//PLEKHF2//SERPINI1//SPOCK1//RECK//PCOLCE2//SERP1//PRR16//FXR1//SESN3//RAB12//SESN1//SNX5//RHEB//TOPORS//KLF6//EGR2//MAPRE3//BAMBI//RGMB//IRF1//NEUROD1//NPAS2//OTX2//ZNF287//RNF4//TEAD1//TGFB1I1//WNT7A//DKK3//PAX9//RASD1//ATXN1//BTAF1//CAPRIN2//ACSL1//RGL1//TNFRSF21//ACOX1//UGT1A9//SPOPL//SOSTDC1//TMEM100//RGMA//SULF1//CTDSPL2//ADRB1//EGLN1//BAG5//RAP2C//CMKLR1//SLK//CCT6A//BAG6//RNF180//ANKIB1//LONRF3//LONRF1//MASTL//EPHA5//MYLIP//OSM//PFN2//IFNA1//EPHA2//EPHA4//DSTYK//ADCY3//PRKACB//PRKAR2B//NSMF//RNF128//ADIPOR2//PTGES3//KCTD20//ITGA6//BTBD10//ITM2B//ARL6IP1//BCL2L11//ITGAV//FZD6//FASTK//ESRP1//AHNAK//YTHDF2//FZD10//AIDA//TWF1//PIK3R3//UBXN2B//TMEFF2//MID1IP1//HECW2//LATS2//IPO5//CCNY//CCNC//MMD//ASPH//UGT1A8//UGT1A1//CDK8//CREBRF//NKX2-3//E2F3//E2F5//S1PR1//EZH1//DLL1//MEF2D//MEOX2//PBX3//IER5//PPP3R1//DCAF6//SHOX//STAT4//PCGF5//ST18//PAIP2//HPRT1//ITGA5//LYPLA1//SH3D19//FAM20C//NOVA1//ARRDC3//ARRDC4//CCL2//CHRM5//PMEPA1//LDLRAD4//IDH1//RANBP9//DNAJC27//CCL8//PLAA//ORMDL3//CPEB2//CALCOCO2//FYCO1//CRTAP//FEZ2//FBXL5//DERL2//EDEM1//FRY//CTSA//KLHL15//NSMAF//CPEB1//CPEB4//UNK//PIKFYVE//TBC1D12// |
| GO:0045944 | positive regulation of transcription by RNA polymerase II | Biological process | 122 | 1132 | 934 | 17653 | 2.0369719508781 | 1.96723593292051e-14 | 1.33044166143414e-11 | 13.7061435515088 | 0.130620985010707 | KAT2B//KLF2//ESR1//JUN//STAT3//TEAD1//TGFB2//CEBPB//FBXW7//NPNT//HAND2//CDK8//ZBTB18//ZNF268//WWP2//OSR1//KLF6//CREBRF//NKX2-3//GLIS3//E2F1//E2F3//E2F5//S1PR1//EDN1//EGR2//ELK3//EN1//ERG//ETS1//EZH1//FOXJ3//FOXF1//FOXF2//FOXC1//FLI1//ZFPM2//NR5A2//GATA6//DLL1//ATAD2//GRHL1//CNOT7//H2AFZ//NRG1//IGF1//IL2//ITGA6//IRF1//AR//LRP6//MAF//MEF2D//MEOX2//MET//MYB//MYBL1//PPP1R12A//NCK1//NEUROD1//NEUROG1//NFIA//NFIB//NHLH2//NPAS2//NR4A2//OSM//OTX2//P2RY1//PAX9//PBX3//IER5//ARID4B//PKD2//POU3F2//POU4F1//EGLN1//PPARG//PPP3R1//DCAF6//RGMA//RAD21//RARB//ARID4A//REST//RNF4//PRDM16//SOX17//SHOX//BCL11B//BMPR2//CAPRIN2//SOX4//SOX11//STAT4//ZEB1//TCF21//TEF//TFAP2A//TFAP2C//THRA//KLF10//TNF//WNT7A//TBL1XR1//NR4A3//FOSL1//HMGA2//PCGF5//KLF7//CBFB//SQSTM1//CCNC//FUBP3//RPS6KA4//ACVR1//NOG//RPS6KA5//KLF4//HAND1//CLOCK//ST18// |
| GO:0010604 | positive regulation of macromolecule metabolic process | Biological process | 255 | 3106 | 934 | 17653 | 1.55170933924945 | 2.4151455083252e-14 | 1.48487537025485e-11 | 13.6170566986354 | 0.273019271948608 | GADD45A//MINK1//TNF//MAP3K2//CRK//CRKL//MAP3K5//TAOK3//LAMTOR3//ADAM9//DUSP5//LPAR1//LPAR3//NRG1//IGF1//NTF3//PIK3CB//PRKAA1//MAPK1//MAP2K4//EPS15//NSF//FAM129A//EFNA1//EFNA5//EPHA7//FMR1//NPTN//KDR//P2RY1//AKTIP//SQSTM1//CCND2//FNIP1//EREG//FBXW7//KMT2B//DLC1//EGLN3//F3//PMAIP1//PPARG//KAT2B//COPS8//MDFIC//MTPN//OSR1//ARX//DNMT3B//DRD1//E2F1//ETS1//RAB3GAP1//GJA1//AR//ITGB8//LDLR//MSN//POU4F1//PTEN//SOX11//STAT3//ACTC1//TFAP2A//HMGA2//FUBP3//ZNF804A//NOG//KLF4//QKI//HAND2//PPM1F//CALM2//GDF11//INHBB//BMP3//BMPR2//TGFB2//ACVR1//EDN1//PCOLCE2//CPEB3//SERP1//PRR16//SOX4//FXR1//TOPORS//KLF2//KLF6//EGR2//ESR1//FHL2//MAPRE3//FOXF1//FOXF2//FOXC1//FLI1//NR5A2//BAMBI//GATA6//RGMB//ATAD2//SERTAD3//FOXN2//IRF1//JUN//LHX1//LRP6//MYB//MYBL1//NEUROD1//NFE2//NPAS2//NPAT//OTX2//TRERF1//ZNF287//REST//RNF4//PRDM16//SOX17//TEAD1//TGFB1I1//WNT7A//TBL1XR1//RUNX3//CLOCK//NDFIP2//WFS1//PKD2//RAP2C//PAK6//TGFBR2//SLK//CCT6A//RNF180//ANKIB1//LONRF3//LONRF1//SOCS5//PPP1R15B//RPS6KA4//RPS6KA5//OSM//PFN2//CAPRIN2//IFNA1//PIAS4//ADCY3//PRKACB//PRKAR2B//CNEP1R1//NSMF//PPP1R12A//NCK1//IL2//BCL2L11//EPHA2//EPHA4//EPHA5//ITGAV//MET//FZD10//WWP2//MYLIP//HECW2//CCNY//CCNC//DEK//CDKN1A//MMD//VLDLR//ASPH//CDK8//ZBTB18//CEBPB//ZNF268//CREBRF//NKX2-3//GLIS3//E2F3//E2F5//S1PR1//ELK3//EN1//ERG//EZH1//FOXJ3//ZFPM2//DLL1//GRHL1//CNOT7//H2AFZ//ITGA6//MAF//MEF2D//MEOX2//NEUROG1//NFIA//NFIB//NHLH2//NR4A2//PAX9//PBX3//IER5//ARID4B//POU3F2//EGLN1//PPP3R1//DCAF6//RGMA//RAD21//RARB//ARID4A//SHOX//BCL11B//STAT4//ZEB1//TCF21//TEF//TFAP2C//THRA//KLF10//NR4A3//FOSL1//PCGF5//KLF7//CBFB//HAND1//ST18//ITGA5//LRP8//SH3D19//ARRDC3//ARRDC4//JARID2//CCL2//PTGES3//TOB1//BTG2//CNOT6L//YTHDF2//NPNT//DSTYK//DNAJC27//CCL8//ACSL1//PPP2CA//NANOS1//FBXL5//UBE2I//EDEM1//ATXN3//BAG6//RNF128// |
| GO:0009653 | anatomical structure morphogenesis | Biological process | 226 | 2656 | 934 | 17653 | 1.60824427259359 | 3.07211123611425e-14 | 1.73139069082006e-11 | 13.5125630632642 | 0.241970021413276 | VASH2//FRY//KLF2//CDH20//NRG1//TGFB2//THRA//WNT7A//S1PR1//EFNA1//EPHA2//ELK3//EREG//FOXC1//GAB1//CLIC4//HOXA3//ITGA5//ITGAV//JUN//KDR//MEOX2//MMP2//TMEM100//PTEN//CCL2//SOX17//VEGFB//HAND1//HAND2//EDN1//PPP3R1//TGFBR2//ACVR1//GJC1//FOXF1//ZFPM2//QKI//NPNT//LHX1//PKD2//TCF21//TBC1D20//AGFG1//TMF1//LRP6//RNF2//DUSP1//DUSP2//DUSP5//NOG//NF2//BMPR2//NR4A3//STAT3//DLL1//LATS2//SOX4//SOX11//DLC1//PRKACB//TSC1//FZD6//MET//GJA1//MEF2D//ARHGAP12//SLIT2//GATA6//RARB//ERG//SOS1//POU4F1//PALLD//AR//BCL11B//FRMD6//TFAP2A//CRKL//E2F5//STX2//FLI1//BHLHE41//NTNG1//SLITRK3//NEFH//OGN//DSCAML1//RANBP9//ARX//DPYSL2//EFNA5//EPHA5//GRB2//KIF5A//OTX2//PIK3CB//ENAH//MAPK1//NTN4//VLDLR//KLF7//RPS6KA5//FEZ2//KLF4//ADAM12//EGR2//EPHA4//CDC42EP3//WASF3//PALM2//FGD4//CRK//FGD5//LPAR1//EPS8//BAMBI//RHOC//RND3//MSN//BRWD1//WASL//BNIP3//TOPORS//FJX1//KBTBD8//TGFB1I1//SPRED1//SULF1//PPARG//NEUROG1//MTPN//NR4A2//NFIB//SH3D19//NKX2-3//TMOD3//TMOD1//ACTC1//MBNL1//RAB21//SOSTDC1//KRT27//EPHA7//PIKFYVE//LRIG1//ADAM9//ASPH//OSR1//RDH10//EN1//RECK//BCL2L11//HSBP1//LAMA3//HMGA2//FOXF2//SOBP//TSHZ1//LHX8//COL1A2//PAX9//GNAQ//ERRFI1//TMEFF2//CFL2//ETS1//EGLN1//F3//ITGB8//BTG1//GOLGA4//NEUROD1//GDF11//ZEB1//YTHDF2//SERP1//ARID5B//UNK//ULK2//LPAR3//PLEKHA1//GPM6A//MNX1//NTF3//MINK1//HPRT1//FMR1//NSMF//HECW2//FXR1//SEMA4B//POU3F2//CAPRIN2//FRZB//MAN2A1//VANGL1//RAB3GAP1//FHL2//ESR1//TNF//ZNF568//BTBD7//CTNND2//LRP8//ADIPOR2//NR5A2//FAM20C//ASPN//KLHL3//CEBPB//SNX2//TMEM135//MIEF1//DDHD1//RAB23//ITGA6//DOCK5//EPN1//JAK1//PLAA//FLRT1//FBXW7//EZH1//FOXJ3//DKK3// |
| GO:0051254 | positive regulation of RNA metabolic process | Biological process | 149 | 1523 | 934 | 17653 | 1.84908983031068 | 5.96553256431219e-14 | 3.10345359480333e-11 | 13.2243507792497 | 0.159528907922912 | KMT2B//KAT2B//TOPORS//KLF2//MAP3K2//KLF6//E2F1//EGR2//ESR1//ETS1//FHL2//MAPRE3//FOXF1//FOXF2//FOXC1//FLI1//NR5A2//BAMBI//GATA6//RGMB//ATAD2//SERTAD3//MDFIC//FOXN2//IGF1//IRF1//AR//JUN//LHX1//LRP6//MAP3K5//MYB//MYBL1//NEUROD1//NFE2//NPAS2//NPAT//OTX2//PPARG//TRERF1//MAPK1//ZNF287//REST//RNF4//PRDM16//SOX17//SOX4//SOX11//STAT3//TEAD1//TFAP2A//TGFB1I1//TNF//WNT7A//TBL1XR1//HMGA2//RUNX3//FUBP3//ACVR1//KLF4//HAND2//CLOCK//CDK8//ZBTB18//CEBPB//ZNF268//WWP2//OSR1//CREBRF//NKX2-3//GLIS3//E2F3//E2F5//S1PR1//EDN1//ELK3//EN1//ERG//EZH1//FOXJ3//ZFPM2//DLL1//GRHL1//CNOT7//H2AFZ//NRG1//IL2//ITGA6//MAF//MEF2D//MEOX2//MET//PPP1R12A//NCK1//NEUROG1//NFIA//NFIB//NHLH2//NR4A2//OSM//P2RY1//PAX9//PBX3//IER5//ARID4B//PKD2//POU3F2//POU4F1//EGLN1//PPP3R1//DCAF6//RGMA//RAD21//RARB//ARID4A//SHOX//BCL11B//BMPR2//CAPRIN2//STAT4//ZEB1//TCF21//TEF//TFAP2C//THRA//KLF10//NR4A3//FOSL1//PCGF5//KLF7//CBFB//SQSTM1//CCNC//RPS6KA4//NOG//RPS6KA5//HAND1//ST18//TOB1//CPEB3//BTG2//CNOT6L//YTHDF2//QKI//NANOS1//TGFB2//PRKAA1//FBXW7//NPNT// |
| GO:0051171 | regulation of nitrogen compound metabolic process | Biological process | 437 | 6240 | 934 | 17653 | 1.32363576154395 | 9.92900268977518e-14 | 4.79641751363925e-11 | 13.0030943716536 | 0.467880085653105 | CDKN1A//GADD45A//PTEN//CCNE2//E2F1//NPAT//RRM2//TSHZ1//HMG20A//KLF2//ZBTB18//CEBPB//ZNF268//TCERG1//WWP2//KLF12//OSR1//CRY2//GLIS3//ARX//DNMT3B//DR1//EDN1//EFNA1//CC2D1B//EN1//ESR1//FHL2//CPEB3//FOXF1//FOXC1//MYT1L//CIC//ZFPM2//DNAJB5//GATA6//CNOT7//HSBP1//AR//JARID2//JUN//ZNF568//MAF//MDM4//MYB//NFIA//NFIB//NR4A2//RLIM//KLF3//ZBTB7A//PIAS4//POU4F1//PPARG//CC2D1A//TSHZ3//ZBTB4//RARB//RBBP7//RBBP8//REST//RNF2//PRDM16//SOX17//SOX11//STAT3//ZEB1//TCF21//TFAP2A//TFAP2C//TGIF1//KLF10//TNF//UBE2I//VLDLR//WFS1//ZNF217//LRP8//BTG2//BHLHE41//TBL1XR1//NR4A3//HMGA2//ARID5B//BRMS1L//BHLHE40//RUNX3//URI1//PER2//SQSTM1//NOG//KLF4//HAND1//FNIP1//PHF14//USP3//MINK1//MAP3K2//CRK//CRKL//MAP3K5//TAOK3//LAMTOR3//ADAM9//DUSP5//LPAR1//LPAR3//NRG1//IGF1//NTF3//PIK3CB//PRKAA1//MAPK1//MAP2K4//DUSP10//SPRED1//DUSP1//DUSP2//PPP2CA//RGS3//RBFOX3//FMR1//MBNL1//RBM25//SRSF2//SECISBP2L//DRD1//FAM129A//IL2//LRP6//PPP1R15B//SLIT2//EFNA5//EPHA7//NPTN//KDR//P2RY1//AKTIP//CCND2//PKD2//THRA//EREG//FBXW7//ERRFI1//TSG101//SOCS5//NUP35//NUP43//ESCO2//BAHD1//H2AFZ//KMT2B//DPY30//HIPK3//KHDRBS3//ZMYND11//ZNF597//CTNND2//ZNF782//ZNF800//ELAVL2//ZBTB9//KDM2A//MYCBP2//FLII//CNOT6L//NR5A2//GTF2E1//SERTAD3//NRBF2//HIVEP2//HMGB3//ZBTB41//ZBTB34//ATXN3//MLLT6//RNF141//BNC2//ZNF532//CDCA7L//LMO3//SLC2A4RG//PAK6//PHTF2//ZNF630//CNOT6//ZNF512B//ZBTB26//BCL11B//SNAPC1//SOX4//BTF3//BTG1//TMF1//ZNF2//ZNF3//ZNF131//ZNF655//SNIP1//TCF7L1//WASL//RPS6KA4//RPS6KA5//ZBTB47//CLOCK//IRF9//TADA1//ZNF367//ELK3//EMX2//ERG//ETS1//FOXJ3//FOXF2//FLI1//EPC2//LHX6//HBP1//ATAD2//GRHL1//MNX1//HOXA3//HOXD1//FOXN2//BOLA3//NR3C2//LHX8//MYBL1//NEUROG1//NFE2//NHLH2//ARID4B//PLEK//BRWD1//POU3F2//TRERF1//ZNFX1//MIER1//RAD21//ARID4A//RBL2//DMRTA1//NKX2-4//RFX7//SMARCD2//TEF//DEK//BRD3//FOSL1//LCOR//KLF7//CBFB//POLR3G//BDP1//MTPN//DDX6//MKNK2//ACO1//TSC1//KBTBD8//PPM1E//FLRT1//GNAQ//NCK1//NF2//ASPN//DUS2//YWHAG//CAMK2N2//PPM1F//DLC1//EGLN3//F3//PMAIP1//KAT2B//COPS8//G3BP2//MDFIC//PPP4R2//CALM2//GDF11//INHBB//BMP3//BMPR2//TGFB2//ACVR1//LDLR//SERPINI1//SPOCK1//RECK//PCOLCE2//SERP1//PRR16//FXR1//TOPORS//KLF6//EGR2//MAPRE3//BAMBI//RGMB//IRF1//LHX1//NEUROD1//NPAS2//OTX2//ZNF287//RNF4//TEAD1//TGFB1I1//WNT7A//FUBP3//HAND2//DKK3//PAX9//RASD1//ATXN1//BTAF1//TOB1//NANOS1//CAPRIN2//SPOPL//SOSTDC1//TMEM100//RGMA//SULF1//CTDSPL2//ADRB1//PTHLH//EGLN1//BAG5//NDFIP2//CDK6//RAP2C//CMKLR1//TGFBR2//SLK//DCP2//CCT6A//BAG6//RNF180//ANKIB1//LONRF3//LONRF1//PPP1R37//MASTL//EPHA5//MYLIP//OSM//PFN2//IFNA1//ADCY3//PRKACB//PRKAR2B//CNEP1R1//NSMF//PPP1R12A//RNF128//ITM2B//ARL6IP1//BCL2L11//GRB2//EPHA2//EPHA4//ITGAV//MET//FZD6//FASTK//ESRP1//AHNAK//YTHDF2//FZD10//AIDA//TWF1//UBXN2B//GJA1//NSF//HECW2//LATS2//IPO5//CCNY//CCNC//MMD//ASPH//CDK8//CREBRF//NKX2-3//E2F3//E2F5//S1PR1//EZH1//DLL1//ITGA6//MEF2D//MEOX2//PBX3//IER5//PPP3R1//DCAF6//SHOX//STAT4//PCGF5//ST18//PAIP2//HPRT1//ITGA5//SH3D19//NOVA1//ARRDC3//ARRDC4//PTGES3//PMEPA1//LDLRAD4//QKI//RANBP9//NPNT//DSTYK//DNAJC27//CCL2//CCL8//ACSL1//PLAA//ORMDL3//CPEB2//CRTAP//RAB3GAP1//FBXL5//MSN//TOB2//DERL2//EDEM1//FRY//CTSA//KLHL15//NSMAF//CPEB1//CPEB4//UNK// |
| GO:0045893 | positive regulation of transcription, DNA-templated | Biological process | 142 | 1450 | 934 | 17653 | 1.85093849221 | 2.41864232882584e-13 | 1.01516372310051e-10 | 12.6164283507792 | 0.15203426124197 | KMT2B//KAT2B//ATAD2//KLF2//ESR1//CDK8//ZBTB18//CEBPB//ZNF268//WWP2//OSR1//KLF6//CREBRF//NKX2-3//GLIS3//E2F1//E2F3//E2F5//S1PR1//EDN1//EGR2//ELK3//EN1//ERG//ETS1//EZH1//FOXJ3//FOXF1//FOXF2//FOXC1//FLI1//ZFPM2//NR5A2//GATA6//DLL1//GRHL1//CNOT7//H2AFZ//NRG1//IGF1//IL2//ITGA6//IRF1//AR//JUN//LRP6//MAF//MEF2D//MEOX2//MET//MYB//MYBL1//PPP1R12A//NCK1//NEUROD1//NEUROG1//NFIA//NFIB//NHLH2//NPAS2//NR4A2//OSM//OTX2//P2RY1//PAX9//PBX3//IER5//ARID4B//PKD2//POU3F2//POU4F1//EGLN1//PPARG//PPP3R1//DCAF6//RGMA//RAD21//RARB//ARID4A//REST//RNF4//PRDM16//SOX17//SHOX//BCL11B//BMPR2//CAPRIN2//SOX4//SOX11//STAT3//STAT4//ZEB1//TCF21//TEAD1//TEF//TFAP2A//TFAP2C//THRA//KLF10//TNF//WNT7A//TBL1XR1//NR4A3//FOSL1//HMGA2//PCGF5//KLF7//CBFB//SQSTM1//CCNC//FUBP3//RPS6KA4//ACVR1//NOG//RPS6KA5//KLF4//HAND1//HAND2//CLOCK//ST18//TGFB2//PRKAA1//FBXW7//NPNT//TOPORS//MAP3K2//FHL2//MAPRE3//BAMBI//RGMB//SERTAD3//MDFIC//FOXN2//LHX1//MAP3K5//NFE2//NPAT//TRERF1//MAPK1//ZNF287//TGFB1I1//RUNX3// |
| GO:1903508 | positive regulation of nucleic acid-templated transcription | Biological process | 142 | 1450 | 934 | 17653 | 1.85093849221 | 2.41864232882584e-13 | 1.01516372310051e-10 | 12.6164283507792 | 0.15203426124197 | KMT2B//KAT2B//TOPORS//KLF2//MAP3K2//KLF6//E2F1//EGR2//ESR1//ETS1//FHL2//MAPRE3//FOXF1//FOXF2//FOXC1//FLI1//NR5A2//BAMBI//GATA6//RGMB//ATAD2//SERTAD3//MDFIC//FOXN2//IGF1//IRF1//AR//JUN//LHX1//LRP6//MAP3K5//MYB//MYBL1//NEUROD1//NFE2//NPAS2//NPAT//OTX2//PPARG//TRERF1//MAPK1//ZNF287//REST//RNF4//PRDM16//SOX17//SOX4//SOX11//STAT3//TEAD1//TFAP2A//TGFB1I1//TNF//WNT7A//TBL1XR1//HMGA2//RUNX3//FUBP3//ACVR1//KLF4//HAND2//CLOCK//CDK8//ZBTB18//CEBPB//ZNF268//WWP2//OSR1//CREBRF//NKX2-3//GLIS3//E2F3//E2F5//S1PR1//EDN1//ELK3//EN1//ERG//EZH1//FOXJ3//ZFPM2//DLL1//GRHL1//CNOT7//H2AFZ//NRG1//IL2//ITGA6//MAF//MEF2D//MEOX2//MET//PPP1R12A//NCK1//NEUROG1//NFIA//NFIB//NHLH2//NR4A2//OSM//P2RY1//PAX9//PBX3//IER5//ARID4B//PKD2//POU3F2//POU4F1//EGLN1//PPP3R1//DCAF6//RGMA//RAD21//RARB//ARID4A//SHOX//BCL11B//BMPR2//CAPRIN2//STAT4//ZEB1//TCF21//TEF//TFAP2C//THRA//KLF10//NR4A3//FOSL1//PCGF5//KLF7//CBFB//SQSTM1//CCNC//RPS6KA4//NOG//RPS6KA5//HAND1//ST18//TGFB2//PRKAA1//FBXW7//NPNT// |
| GO:1902680 | positive regulation of RNA biosynthetic process | Biological process | 142 | 1451 | 934 | 17653 | 1.84966286264955 | 2.55179406960057e-13 | 1.01516372310051e-10 | 12.5931543762066 | 0.15203426124197 | KMT2B//KAT2B//TOPORS//KLF2//MAP3K2//KLF6//E2F1//EGR2//ESR1//ETS1//FHL2//MAPRE3//FOXF1//FOXF2//FOXC1//FLI1//NR5A2//BAMBI//GATA6//RGMB//ATAD2//SERTAD3//MDFIC//FOXN2//IGF1//IRF1//AR//JUN//LHX1//LRP6//MAP3K5//MYB//MYBL1//NEUROD1//NFE2//NPAS2//NPAT//OTX2//PPARG//TRERF1//MAPK1//ZNF287//REST//RNF4//PRDM16//SOX17//SOX4//SOX11//STAT3//TEAD1//TFAP2A//TGFB1I1//TNF//WNT7A//TBL1XR1//HMGA2//RUNX3//FUBP3//ACVR1//KLF4//HAND2//CLOCK//CDK8//ZBTB18//CEBPB//ZNF268//WWP2//OSR1//CREBRF//NKX2-3//GLIS3//E2F3//E2F5//S1PR1//EDN1//ELK3//EN1//ERG//EZH1//FOXJ3//ZFPM2//DLL1//GRHL1//CNOT7//H2AFZ//NRG1//IL2//ITGA6//MAF//MEF2D//MEOX2//MET//PPP1R12A//NCK1//NEUROG1//NFIA//NFIB//NHLH2//NR4A2//OSM//P2RY1//PAX9//PBX3//IER5//ARID4B//PKD2//POU3F2//POU4F1//EGLN1//PPP3R1//DCAF6//RGMA//RAD21//RARB//ARID4A//SHOX//BCL11B//BMPR2//CAPRIN2//STAT4//ZEB1//TCF21//TEF//TFAP2C//THRA//KLF10//NR4A3//FOSL1//PCGF5//KLF7//CBFB//SQSTM1//CCNC//RPS6KA4//NOG//RPS6KA5//HAND1//ST18//TGFB2//PRKAA1//FBXW7//NPNT// |
| GO:0060255 | regulation of macromolecule metabolic process | Biological process | 445 | 6425 | 934 | 17653 | 1.30905689932427 | 3.15698799435752e-13 | 1.18615054476888e-10 | 12.5007270696739 | 0.47644539614561 | CDKN1A//GADD45A//PTEN//CCNE2//E2F1//NPAT//RRM2//TSHZ1//HMG20A//KLF2//ZBTB18//CEBPB//ZNF268//TCERG1//WWP2//KLF12//OSR1//CRY2//GLIS3//ARX//DNMT3B//DR1//EDN1//EFNA1//CC2D1B//EN1//ESR1//FHL2//CPEB3//FOXF1//FOXC1//MYT1L//CIC//ZFPM2//DNAJB5//GATA6//CNOT7//HSBP1//AR//JARID2//JUN//ZNF568//MAF//MDM4//MYB//NFIA//NFIB//NR4A2//RLIM//KLF3//ZBTB7A//PIAS4//POU4F1//PPARG//CC2D1A//TSHZ3//ZBTB4//RARB//RBBP7//RBBP8//REST//RNF2//PRDM16//SOX17//SOX11//STAT3//ZEB1//TCF21//TFAP2A//TFAP2C//TGIF1//KLF10//TNF//UBE2I//VLDLR//WFS1//ZNF217//LRP8//BTG2//BHLHE41//TBL1XR1//NR4A3//HMGA2//ARID5B//BRMS1L//BHLHE40//RUNX3//URI1//PER2//SQSTM1//NOG//KLF4//HAND1//FNIP1//PHF14//USP3//DCP2//PPP2CA//RPL15//MINK1//MAP3K2//CRK//CRKL//MAP3K5//TAOK3//LAMTOR3//ADAM9//DUSP5//LPAR1//LPAR3//NRG1//IGF1//NTF3//PIK3CB//PRKAA1//MAPK1//MAP2K4//DUSP10//SPRED1//DUSP1//DUSP2//RGS3//CNOT6L//PAN3//CNOT6//RBFOX3//FMR1//MBNL1//RBM25//SRSF2//SECISBP2L//EPS15//NSF//DRD1//FAM129A//IL2//LRP6//PPP1R15B//SLIT2//EFNA5//EPHA7//NPTN//KDR//P2RY1//AKTIP//CCND2//PKD2//THRA//EREG//FBXW7//ERRFI1//TSG101//SOCS5//ESCO2//BAHD1//H2AFZ//KMT2B//DPY30//ARID4B//ARID4A//HIPK3//KHDRBS3//ZMYND11//NUP35//ZNF597//CTNND2//ZNF782//ZNF800//ELAVL2//ZBTB9//KDM2A//MYCBP2//FLII//NR5A2//GTF2E1//SERTAD3//NRBF2//HIVEP2//HMGB3//ZBTB41//ZBTB34//ATXN3//MLLT6//RNF141//BNC2//ZNF532//CDCA7L//LMO3//SLC2A4RG//PAK6//PHTF2//ZNF630//ZNF512B//ZBTB26//BCL11B//SNAPC1//SOX4//BTF3//BTG1//TMF1//ZNF2//ZNF3//ZNF131//ZNF655//SNIP1//TCF7L1//WASL//RPS6KA4//RPS6KA5//ZBTB47//CLOCK//IRF9//TADA1//ZNF367//ELK3//EMX2//ERG//ETS1//FOXJ3//FOXF2//FLI1//EPC2//LHX6//HBP1//ATAD2//GRHL1//MNX1//HOXA3//HOXD1//FOXN2//BOLA3//NR3C2//LHX8//MYBL1//NEUROG1//NFE2//NHLH2//PLEK//BRWD1//POU3F2//TRERF1//ZNFX1//MIER1//RAD21//RBL2//DMRTA1//NKX2-4//RFX7//SMARCD2//TEF//DEK//BRD3//FOSL1//LCOR//KLF7//CBFB//POLR3G//BDP1//MTPN//DDX6//MKNK2//ACO1//TSC1//KBTBD8//PPM1E//FLRT1//GNAQ//NCK1//NF2//ASPN//DUS2//YWHAG//CAMK2N2//PPM1F//DLC1//EGLN3//F3//PMAIP1//KAT2B//COPS8//G3BP2//MDFIC//TOB1//CDK6//TOB2//LHX1//PTHLH//TGFBR2//PPP4R2//NANOS1//RAB3GAP1//GJA1//ITGB8//LDLR//MSN//ACTC1//FUBP3//ZNF804A//QKI//HAND2//NME1//NDFIP2//TGFB2//CALM2//GDF11//INHBB//BMP3//BMPR2//ACVR1//SERPINI1//SPOCK1//RECK//PCOLCE2//SERP1//PRR16//FXR1//TOPORS//KLF6//EGR2//MAPRE3//BAMBI//RGMB//IRF1//NEUROD1//NPAS2//OTX2//ZNF287//RNF4//TEAD1//TGFB1I1//WNT7A//DKK3//PAX9//RASD1//ATXN1//BTAF1//CAPRIN2//SPOPL//SOSTDC1//TMEM100//RGMA//SULF1//CTDSPL2//BAG5//RAP2C//CMKLR1//SLK//CCT6A//BAG6//RNF180//ANKIB1//LONRF3//LONRF1//PPP1R37//MASTL//EPHA5//MYLIP//OSM//PFN2//IFNA1//ADCY3//PRKACB//PRKAR2B//CNEP1R1//NSMF//PPP1R12A//RNF128//ITM2B//ARL6IP1//BCL2L11//GRB2//EPHA2//EPHA4//ITGAV//MET//EGLN1//FZD6//FASTK//ESRP1//AHNAK//YTHDF2//FZD10//AIDA//TWF1//UBXN2B//TMEFF2//HECW2//LATS2//IPO5//CCNY//CCNC//MMD//ASPH//CDK8//CREBRF//NKX2-3//E2F3//E2F5//S1PR1//EZH1//DLL1//ITGA6//MEF2D//MEOX2//PBX3//IER5//PPP3R1//DCAF6//SHOX//STAT4//PCGF5//ST18//PAIP2//ITGA5//SH3D19//NOVA1//ARRDC3//ARRDC4//CCL2//PTGES3//CHRM5//MTMR9//PMEPA1//LDLRAD4//NUP43//RANBP9//NPNT//DSTYK//DNAJC27//CCL8//ACSL1//PLAA//ORMDL3//CPEB2//CRTAP//FBXL5//DERL2//EDEM1//FRY//CTSA//KLHL15//NSMAF//CPEB1//CPEB4//UNK// |
| GO:0048523 | negative regulation of cellular process | Biological process | 353 | 4821 | 934 | 17653 | 1.38391436999174 | 4.98302608507146e-13 | 1.77369502175465e-10 | 12.3025068393846 | 0.377944325481799 | E2F1//USP28//CLOCK//TSHZ1//HMG20A//KLF2//ZBTB18//CEBPB//ZNF268//TCERG1//WWP2//KLF12//OSR1//CRY2//GLIS3//ARX//DNMT3B//DR1//EDN1//EFNA1//CC2D1B//EN1//ESR1//FHL2//CPEB3//FOXF1//FOXC1//MYT1L//CIC//ZFPM2//DNAJB5//GATA6//CNOT7//HSBP1//AR//JARID2//JUN//ZNF568//MAF//MDM4//MYB//NFIA//NFIB//NR4A2//RLIM//KLF3//ZBTB7A//PIAS4//POU4F1//PPARG//CC2D1A//TSHZ3//ZBTB4//RARB//RBBP7//RBBP8//REST//RNF2//PRDM16//SOX17//SOX11//STAT3//ZEB1//TCF21//TFAP2A//TFAP2C//TGIF1//KLF10//TNF//UBE2I//VLDLR//WFS1//ZNF217//LRP8//BTG2//BHLHE41//TBL1XR1//NR4A3//HMGA2//ARID5B//BRMS1L//BHLHE40//RUNX3//URI1//PER2//SQSTM1//NOG//KLF4//HAND1//FNIP1//PHF14//USP3//DUSP10//SPRED1//DUSP1//DUSP2//DUSP5//PPP2CA//RGS3//FAM129A//IL2//LRP6//PTEN//PPP1R15B//SLIT2//SULF1//GJA1//NF2//NME1//BMPR2//EPS8//TWF1//ERRFI1//TSG101//SOCS5//BAHD1//H2AFZ//DPY30//GADD45A//PPM1E//FLRT1//GNAQ//NCK1//TAOK3//ASPN//DUS2//YWHAG//CAMK2N2//PPM1F//CDKN1A//CNOT6L//CNOT6//RBL2//SOX4//STMN2//MID1IP1//CDK6//HBP1//MTBP//IRF1//PKD2//PRKAA1//RRAGD//RHEB//TGFB2//TSC1//LAMTOR3//KAT2B//NABP1//ADAM10//MIA3//HIPK3//TOPORS//EPHA2//EPHA4//EPHA5//EPHA7//ARL6IP1//DSTYK//IGF1//KDR//MET//ZFAND6//SPHK2//BCL11B//BNIP3//ACTC1//TMF1//WNT7A//BAG6//CCND2//TOB1//GDF11//DLC1//TOB2//COPS8//BTG3//EREG//ETS1//FRZB//TES//DLL1//ASPH//OSM//PTHLH//STRN//BTG1//TGFB1I1//FOSL1//ST18//RGS17//ACVR1//SNIP1//SOSTDC1//MTMR9//MYBL1//PFN2//PTPRG//HAND2//P2RY1//LDLRAD4//ITGAV//C5ORF30//CALM2//SPOCK1//FBXW7//PLEK//PPP1R37//PPP1R12A//PPP1R12C//PLEKHF2//SERPINI1//RECK//RAD21//SKP1//LPAR1//MYLIP//BAG5//PMEPA1//ELK3//FOXF2//DKK3//MDFIC//NRG1//LHX1//PAX9//RASD1//ARID4A//ATXN1//THRA//BTAF1//RPS6KA5//INHBB//FMR1//NANOS1//CAPRIN2//FXR1//APCDD1//SHISA2//SERTAD3//ADIPOR2//TMEFF2//CLIC4//RAP2C//BAMBI//RASL11B//TGFBR2//CTDSPL2//EGLN1//LIMA1//TNFRSF21//SPOPL//ATAD2//MIER1//DCP2//SLC6A1//MASTL//RNF4//PTGER4//CCL2//NPLOC4//RNF128//EPS15//GRB2//EPN1//CD274//MARCH7//PDCD1LG2//ITM2B//LYPLA1//PKDCC//ZMYND11//AIDA//NTF3//CPEB4//HMGB3//MAPK1//ZADH2//CXCL14//DDX6//TMEM64//CEP97//LATS2//IPO5//YTHDF2//UGT1A8//PAIP2//NEUROD1//MDM1//MORC3//OGN//ULK2//RGMA//SEMA4B//CD2AP//ARHGAP12//DRD1//PRKACB//S1PR1//ARHGAP6//PPFIA1//TMOD3//TMOD1//OTUD3//PLEKHA1//GLRA1//SLC24A2//GRIA1//NPAS2//NUP35//NUP43//QKI//LDLR//RRM2//E2F3//RANBP9//LMO3//CCL8//CDC14A//MEOX2//SACS//AMER2//FZD6//EFNA5//PLAA//ORMDL3//CREBRF//CPEB2//BTBD10//CRTAP//FEZ2//NPAT//SRSF2//WASL//DNAJB9//DERL2//SESN3//SESN1//FRY//CTSA//KLHL15//ZNF655//CRK//LYPD6//LRCH1//PRKAR2B//MAP2K4//CPEB1//UNK//ITGA5//MTPN//ARHGAP1//ITGA6// |
| GO:0007275 | multicellular organism development | Biological process | 383 | 5351 | 934 | 17653 | 1.35280583548793 | 6.69692742787646e-13 | 2.26456600973642e-10 | 12.1741244073269 | 0.410064239828694 | WNT7A//GDF11//CMKLR1//COL1A2//HAPLN1//EN1//FRZB//SERP1//GNAQ//IGF1//PTHLH//SHOX//BMP3//SOX4//SOX11//TGFB2//KLF10//PKDCC//NOG//THRA//S1PR1//EFNA1//EPHA2//ELK3//EREG//FOXC1//GAB1//CLIC4//HOXA3//ITGA5//ITGAV//JUN//KDR//MEOX2//MMP2//TMEM100//PTEN//CCL2//SOX17//VEGFB//HAND1//HAND2//CEBPB//INHBB//DMRTA1//IMMP2L//KMT2B//ESR1//PLEKHA1//CRKL//FOXF1//SPHK2//TGFBR2//EDN1//PPP3R1//ACVR1//GJC1//ZFPM2//QKI//SMOC1//OSR1//LHX1//RDH10//NPNT//RARB//TCF21//SLIT2//PKD2//BCL2L11//ADAM10//KLF2//GATA6//GJA1//EPN1//AR//ZNF568//MAN2A1//MBNL1//BTF3//FOSL1//LRP6//RNF2//DUSP1//DUSP2//DUSP5//NF2//BMPR2//NR4A3//STAT3//SEMA4B//DLL1//DCX//GPM6A//LRP12//NR4A2//SPOCK1//UNK//SULF1//TFAP2A//TSC1//WFS1//BAG6//ARID5B//LATS2//RBBP8//FOXF2//HMGA2//DLC1//PRKACB//FZD6//JARID2//MET//UGT1A1//PPARG//APCDD1//SOS1//FBXW7//RECK//MEF2D//NFIB//RUNX3//ESCO2//PLEK//RBM47//REST//NKX2-3//FNIP1//MARCH7//NME1//NRG1//ERG//POU4F1//CDK6//NEUROD1//PALLD//BCL11B//SLC17A7//NEUROG1//PBX3//SOSTDC1//ASPH//ZEB1//E2F5//STX2//FLI1//BHLHE41//TRIM3//FUT9//CHRM2//SCRG1//RBFOX3//DPYSL2//EFNA5//MYT1L//FMR1//GLRB//MYLIP//ATXN3//NTF3//TAOK3//POU3F2//VLDLR//ZIC5//ITM2B//FEZ2//NTNG1//SLITRK3//NEFH//OGN//DSCAML1//RANBP9//ARX//EPHA5//GRB2//KIF5A//OTX2//PIK3CB//ENAH//MAPK1//NTN4//KLF7//RPS6KA5//DRD1//NHLH2//NPAS2//SERPINI1//FXR1//B3GNT5//EGR2//EPHA7//RAB3GAP1//CIC//PTPRG//ATXN1//KLF4//ADIPOR2//KAT2B//SGCE//ZBTB18//CFL2//EPHA4//P2RY1//CLDN11//RNF38//TFAP2C//IDH1//LHX8//MORC3//ACO1//RAI2//NR5A2//TSHZ1//EMX2//BTG2//AIDA//BAMBI//TGFB1I1//PPP2CA//LDLRAD4//TOPORS//FJX1//SNAP25//ZNF804A//STMN2//NPTN//NCK1//TWF1//LPAR1//BAG5//WASF3//KBTBD8//MTPN//FLRT1//STRN//SPRED1//MNX1//LRP8//HPRT1//CALM2//SLC32A1//EZH1//LHX6//ETS1//C21ORF91//TACC2//BNIP3//PRDM16//TCF7L1//KCNJ10//MSN//MKNK2//CBFB//FZD10//HOXD1//KLF6//IFNA1//DNAJB9//TSG101//ADAM9//IL2//ASPN//OSTM1//TNF//DKK3//FAM20C//RAB21//TDRD5//KIAA1109//E2F1//KRT27//CDKN1A//FRY//PSD//DSC2//TNFRSF21//PIKFYVE//MAF//LRIG1//TMF1//ITGA6//FOXN2//HSBP1//LAMA3//PTGER4//SOBP//PAX9//SBF2//IRF1//BNC2//ARRDC3//ERRFI1//HMGB3//DUSP10//BTG1//GRHL1//MYB//SOCS5//NFE2//DPY30//YWHAG//HMG20A//DDX6//DNMT3B//MMD//TOB2//TMEM64//EGLN1//VASH2//F3//ITGB8//ADAM12//GOLGA4//LHFPL2//GJB2//PHF14//TEAD1//YTHDF2//PRKAA1//ULK2//LPAR3//RGMA//SLC35D1//RHEB//MINK1//NSMF//HECW2//TMOD3//ARID4A//CXCL14//PER2//CAPRIN2//ACTC1//FHL2//MDM1//VANGL1//NFIA//SYBU//MFAP5//TBL1XR1//BTBD7//SLC12A5//CTNND2//WASL//CPEB3//MAP2K4//NEFM//LDLR//TMOD1//TBC1D20//KLHL3//RAB23//ARID4B//NANOS1//NLN//JAK1//PLAA//CLOCK//PRR15//DZIP1//WDR47//WDFY3//FLII//LCLAT1//CDH20//HIVEP2//AGFG1//SHISA2//OSM//KLF3//ZBTB7A//ZFR//TRERF1//TSHZ3//RBBP7//NKX2-4//TEF//TGIF1//ZNF3//ZNF217//PTP4A1//EVI5//ST6GAL2//ADAM18// |
| GO:0000122 | negative regulation of transcription by RNA polymerase II | Biological process | 92 | 800 | 934 | 17653 | 2.17354925053533 | 1.17670834625642e-12 | 3.7860312236854e-10 | 11.9293311661347 | 0.0985010706638116 | THRA//NFIB//JUN//NCK1//TSHZ1//HMG20A//KLF2//ZBTB18//CEBPB//ZNF268//TCERG1//WWP2//KLF12//OSR1//CRY2//GLIS3//ARX//DNMT3B//DR1//E2F1//EDN1//EFNA1//CC2D1B//EN1//ESR1//FHL2//CPEB3//FOXF1//FOXC1//MYT1L//CIC//ZFPM2//DNAJB5//GATA6//CNOT7//HSBP1//AR//JARID2//ZNF568//MAF//MDM4//MYB//NFIA//NR4A2//RLIM//KLF3//ZBTB7A//PIAS4//POU4F1//PPARG//CC2D1A//TSHZ3//ZBTB4//RARB//RBBP7//RBBP8//REST//RNF2//PRDM16//SOX17//SOX11//STAT3//ZEB1//TCF21//TFAP2A//TFAP2C//TGIF1//KLF10//TNF//UBE2I//VLDLR//WFS1//ZNF217//LRP8//BTG2//BHLHE41//TBL1XR1//NR4A3//HMGA2//ARID5B//BRMS1L//BHLHE40//RUNX3//URI1//PER2//SQSTM1//NOG//KLF4//HAND1//FNIP1//PHF14//USP3// |
| GO:0010557 | positive regulation of macromolecule biosynthetic process | Biological process | 157 | 1698 | 934 | 17653 | 1.74756609993367 | 1.23159377378499e-12 | 3.7860312236854e-10 | 11.9095325153012 | 0.168094218415418 | KMT2B//KAT2B//IGF1//EDN1//FAM129A//CPEB3//FMR1//SERP1//PRR16//MAPK1//SOX4//FXR1//TOPORS//KLF2//MAP3K2//KLF6//E2F1//EGR2//ESR1//ETS1//FHL2//MAPRE3//FOXF1//FOXF2//FOXC1//FLI1//NR5A2//BAMBI//GATA6//RGMB//ATAD2//SERTAD3//MDFIC//FOXN2//IRF1//AR//JUN//LHX1//LRP6//MAP3K5//MYB//MYBL1//NEUROD1//NFE2//NPAS2//NPAT//OTX2//PPARG//TRERF1//ZNF287//REST//RNF4//PRDM16//SOX17//SOX11//STAT3//TEAD1//TFAP2A//TGFB1I1//TNF//WNT7A//TBL1XR1//HMGA2//RUNX3//FUBP3//ACVR1//KLF4//HAND2//CLOCK//CCT6A//NCK1//EREG//TGFB2//MAP2K4//CDK8//ZBTB18//CEBPB//ZNF268//WWP2//OSR1//CREBRF//NKX2-3//GLIS3//E2F3//E2F5//S1PR1//ELK3//EN1//ERG//EZH1//FOXJ3//ZFPM2//DLL1//GRHL1//CNOT7//H2AFZ//NRG1//IL2//ITGA6//MAF//MEF2D//MEOX2//MET//PPP1R12A//NEUROG1//NFIA//NFIB//NHLH2//NR4A2//OSM//P2RY1//PAX9//PBX3//IER5//ARID4B//PKD2//POU3F2//POU4F1//EGLN1//PPP3R1//DCAF6//RGMA//RAD21//RARB//ARID4A//SHOX//BCL11B//BMPR2//CAPRIN2//STAT4//ZEB1//TCF21//TEF//TFAP2C//THRA//KLF10//NR4A3//FOSL1//PCGF5//KLF7//CBFB//SQSTM1//CCNC//RPS6KA4//NOG//RPS6KA5//HAND1//ST18//KDR//CCL2//PTGES3//RAB3GAP1//PRKAA1//YTHDF2//FBXW7//NPNT//MTPN// |
| GO:0048856 | anatomical structure development | Biological process | 409 | 5836 | 934 | 17653 | 1.32458450318704 | 1.43563984973222e-12 | 4.22140534945174e-10 | 11.8429544952548 | 0.437901498929336 | WNT7A//VASH2//FRY//KLF2//CDH20//NRG1//TGFB2//GDF11//CMKLR1//COL1A2//HAPLN1//EN1//FRZB//SERP1//GNAQ//IGF1//PTHLH//SHOX//BMP3//SOX4//SOX11//KLF10//PKDCC//NOG//THRA//S1PR1//EFNA1//EPHA2//ELK3//EREG//FOXC1//GAB1//CLIC4//HOXA3//ITGA5//ITGAV//JUN//KDR//MEOX2//MMP2//TMEM100//PTEN//CCL2//SOX17//VEGFB//HAND1//HAND2//CEBPB//INHBB//DMRTA1//IMMP2L//KMT2B//ESR1//PLEKHA1//YTHDF2//CRKL//FOXF1//SPHK2//TGFBR2//EDN1//PPP3R1//ACVR1//GJC1//ZFPM2//QKI//SMOC1//OSR1//LHX1//RDH10//NPNT//RARB//TCF21//SLIT2//PKD2//TBC1D20//AGFG1//TMF1//BCL2L11//ADAM10//GATA6//GJA1//EPN1//AR//ZNF568//MAN2A1//MBNL1//BTF3//FOSL1//LRP6//RNF2//DUSP1//DUSP2//DUSP5//NF2//BMPR2//NR4A3//STAT3//SEMA4B//DLL1//DCX//GPM6A//LRP12//NR4A2//SPOCK1//UNK//SULF1//TFAP2A//TSC1//WFS1//BAG6//ARID5B//LATS2//RBBP8//FOXF2//HMGA2//DLC1//PRKACB//FZD6//MET//JARID2//UGT1A1//PPARG//APCDD1//SOS1//FBXW7//RECK//MEF2D//ARHGAP12//NFIB//RUNX3//ESCO2//PLEK//RBM47//REST//NKX2-3//FNIP1//MARCH7//NME1//ERG//POU4F1//CDK6//NEUROD1//PALLD//BCL11B//FRMD6//SLC17A7//TSHZ1//EPHA4//EPHA5//EPHA7//PRR15//DZIP1//WDR47//WDFY3//FLII//LCLAT1//TNFRSF21//HIVEP2//HMGB3//SHISA2//NFE2//OSM//MINK1//KLF3//ZBTB7A//ZFR//BTBD7//TRERF1//TSHZ3//RBBP7//NKX2-4//TEF//TGIF1//ZNF3//ZNF217//PTP4A1//EVI5//VANGL1//ST6GAL2//ADAM18//TDRD5//EZH1//FOXJ3//DKK3//MNX1//NEUROG1//PBX3//SOSTDC1//ASPH//ZEB1//E2F5//STX2//FLI1//BHLHE41//NR5A2//AMER2//TRIM3//FUT9//CHRM2//SCRG1//RBFOX3//DPYSL2//EFNA5//MYT1L//FMR1//GLRB//MYLIP//ATXN3//NTF3//TAOK3//POU3F2//VLDLR//ZIC5//ITM2B//FEZ2//NTNG1//SLITRK3//NEFH//OGN//DSCAML1//RANBP9//ARX//GRB2//KIF5A//OTX2//PIK3CB//ENAH//MAPK1//NTN4//KLF7//RPS6KA5//DRD1//NHLH2//NPAS2//SERPINI1//FXR1//B3GNT5//EGR2//RAB3GAP1//CIC//PTPRG//ATXN1//PAX9//PPP2CA//KLF4//ADIPOR2//KAT2B//SGCE//ZBTB18//CFL2//ADAM12//P2RY1//CDC42EP3//WASF3//PALM2//FGD4//CRK//FGD5//LPAR1//EPS8//BAMBI//RHOC//RND3//MSN//BRWD1//WASL//CLDN11//GRHL1//LAMA3//RNF38//TFAP2C//IDH1//LHX8//MORC3//ACO1//RAI2//EMX2//BTG2//AIDA//BNIP3//ITGA6//TGFB1I1//LDLRAD4//TOPORS//FJX1//SNAP25//ZNF804A//STMN2//NPTN//NCK1//TWF1//BAG5//KBTBD8//MTPN//FLRT1//STRN//SPRED1//LRP8//HPRT1//CALM2//SLC32A1//LHX6//ETS1//C21ORF91//TACC2//PRDM16//TCF7L1//KCNJ10//SH3D19//MKNK2//CBFB//FZD10//HOXD1//KLF6//IFNA1//DNAJB9//TSG101//ADAM9//IL2//TMOD3//TMOD1//ACTC1//ASPN//OSTM1//TNF//FAM20C//RAB21//CNN3//GSTA2//KIAA1109//E2F1//KRT27//CDKN1A//PSD//DSC2//PIKFYVE//MAF//LRIG1//SYCP3//FOXN2//HSBP1//PTGER4//SOBP//SBF2//IRF1//BNC2//ARRDC3//ERRFI1//TMEFF2//DUSP10//BTG1//MYB//SOCS5//DPY30//CXCL14//MAP3K5//YWHAG//HMG20A//DDX6//DNMT3B//MMD//TOB2//TMEM64//EGLN1//F3//ITGB8//GOLGA4//LHFPL2//GJB2//PHF14//TEAD1//PRKAA1//ULK2//LPAR3//RGMA//SLC35D1//RHEB//NSMF//HECW2//ARID4A//PER2//CAPRIN2//FHL2//MDM1//NFIA//SYBU//MFAP5//TBL1XR1//SLC12A5//CTNND2//CPEB3//RAP1B//MAP2K4//NEFM//LDLR//KLHL3//SNX2//TMEM135//MIEF1//DDHD1//RAP2C//RAB23//ARID4B//NANOS1//DOCK5//NLN//JAK1//PLAA//CLOCK// |
| GO:1903506 | regulation of nucleic acid-templated transcription | Biological process | 288 | 3766 | 934 | 17653 | 1.44538591090576 | 1.87151645431474e-12 | 5.27377740855441e-10 | 11.7278063502291 | 0.308351177730193 | E2F1//NPAT//RRM2//TSHZ1//HMG20A//KLF2//ZBTB18//CEBPB//ZNF268//TCERG1//WWP2//KLF12//OSR1//CRY2//GLIS3//ARX//DNMT3B//DR1//EDN1//EFNA1//CC2D1B//EN1//ESR1//FHL2//CPEB3//FOXF1//FOXC1//MYT1L//CIC//ZFPM2//DNAJB5//GATA6//CNOT7//HSBP1//AR//JARID2//JUN//ZNF568//MAF//MDM4//MYB//NFIA//NFIB//NR4A2//RLIM//KLF3//ZBTB7A//PIAS4//POU4F1//PPARG//CC2D1A//TSHZ3//ZBTB4//RARB//RBBP7//RBBP8//REST//RNF2//PRDM16//SOX17//SOX11//STAT3//ZEB1//TCF21//TFAP2A//TFAP2C//TGIF1//KLF10//TNF//UBE2I//VLDLR//WFS1//ZNF217//LRP8//BTG2//BHLHE41//TBL1XR1//NR4A3//HMGA2//ARID5B//BRMS1L//BHLHE40//RUNX3//URI1//PER2//SQSTM1//NOG//KLF4//HAND1//FNIP1//PHF14//USP3//PKD2//THRA//BAHD1//H2AFZ//KMT2B//DPY30//HIPK3//KHDRBS3//ZMYND11//NUP35//ZNF597//CTNND2//ZNF782//ZNF800//ELAVL2//ZBTB9//KDM2A//MYCBP2//FLII//CNOT6L//NR5A2//GTF2E1//SERTAD3//NRBF2//HIVEP2//HMGB3//ZBTB41//ZBTB34//ATXN3//MLLT6//RNF141//BNC2//PPP2CA//ZNF532//CDCA7L//LMO3//SLC2A4RG//PAK6//PHTF2//ZNF630//CNOT6//ZNF512B//ZBTB26//BCL11B//SNAPC1//SOX4//BTF3//BTG1//TMF1//ZNF2//ZNF3//ZNF131//ZNF655//SNIP1//TCF7L1//WASL//RPS6KA4//RPS6KA5//ZBTB47//CLOCK//CDKN1A//IRF9//TADA1//CRK//ZNF367//ELK3//EMX2//ERG//ETS1//FOXJ3//FOXF2//FLI1//EPC2//LHX6//HBP1//ATAD2//GRHL1//MNX1//HOXA3//HOXD1//FOXN2//BOLA3//NR3C2//LHX8//MYBL1//NEUROG1//NFE2//NHLH2//ARID4B//PLEK//BRWD1//POU3F2//TRERF1//ZNFX1//MIER1//RAD21//ARID4A//RBL2//DMRTA1//NKX2-4//RFX7//SMARCD2//TEF//DEK//BRD3//FOSL1//LCOR//KLF7//CBFB//POLR3G//BDP1//KAT2B//G3BP2//TOPORS//MAP3K2//KLF6//EGR2//MAPRE3//BAMBI//RGMB//MDFIC//IGF1//IRF1//LHX1//LRP6//MAP3K5//NEUROD1//NPAS2//OTX2//MAPK1//ZNF287//RNF4//TEAD1//TGFB1I1//WNT7A//FUBP3//ACVR1//HAND2//EREG//DKK3//NRG1//PAX9//RASD1//ATXN1//TSG101//BTAF1//PPM1F//TOB1//SOSTDC1//TMEM100//RGMA//BMP3//BMPR2//TGFB2//SULF1//CTDSPL2//CMKLR1//EPHA5//EGLN1//FZD6//CDK8//CREBRF//NKX2-3//E2F3//E2F5//S1PR1//EZH1//DLL1//IL2//ITGA6//MEF2D//MEOX2//MET//PPP1R12A//NCK1//OSM//P2RY1//PBX3//IER5//PPP3R1//DCAF6//SHOX//CAPRIN2//STAT4//PCGF5//CCNC//ST18//PTEN//MTPN//EGLN3//PRKAA1//TOB2//SRSF2//FBXW7//NPNT//RAP2C// |
| GO:0051252 | regulation of RNA metabolic process | Biological process | 301 | 3984 | 934 | 17653 | 1.42796910339431 | 1.96803154150283e-12 | 5.32391892607346e-10 | 11.7059679454418 | 0.322269807280514 | E2F1//NPAT//RRM2//TSHZ1//HMG20A//KLF2//ZBTB18//CEBPB//ZNF268//TCERG1//WWP2//KLF12//OSR1//CRY2//GLIS3//ARX//DNMT3B//DR1//EDN1//EFNA1//CC2D1B//EN1//ESR1//FHL2//CPEB3//FOXF1//FOXC1//MYT1L//CIC//ZFPM2//DNAJB5//GATA6//CNOT7//HSBP1//AR//JARID2//JUN//ZNF568//MAF//MDM4//MYB//NFIA//NFIB//NR4A2//RLIM//KLF3//ZBTB7A//PIAS4//POU4F1//PPARG//CC2D1A//TSHZ3//ZBTB4//RARB//RBBP7//RBBP8//REST//RNF2//PRDM16//SOX17//SOX11//STAT3//ZEB1//TCF21//TFAP2A//TFAP2C//TGIF1//KLF10//TNF//UBE2I//VLDLR//WFS1//ZNF217//LRP8//BTG2//BHLHE41//TBL1XR1//NR4A3//HMGA2//ARID5B//BRMS1L//BHLHE40//RUNX3//URI1//PER2//SQSTM1//NOG//KLF4//HAND1//FNIP1//PHF14//USP3//RBFOX3//FMR1//MBNL1//RBM25//SRSF2//PKD2//THRA//BAHD1//H2AFZ//KMT2B//DPY30//HIPK3//KHDRBS3//ZMYND11//NUP35//ZNF597//CTNND2//ZNF782//ZNF800//ELAVL2//ZBTB9//KDM2A//MYCBP2//FLII//CNOT6L//NR5A2//GTF2E1//SERTAD3//NRBF2//HIVEP2//HMGB3//ZBTB41//ZBTB34//ATXN3//MLLT6//RNF141//BNC2//PPP2CA//ZNF532//CDCA7L//LMO3//SLC2A4RG//PAK6//PHTF2//ZNF630//CNOT6//ZNF512B//ZBTB26//BCL11B//SNAPC1//SOX4//BTF3//BTG1//TMF1//ZNF2//ZNF3//ZNF131//ZNF655//SNIP1//TCF7L1//WASL//RPS6KA4//RPS6KA5//ZBTB47//CLOCK//CDKN1A//IRF9//TADA1//CRK//ZNF367//ELK3//EMX2//ERG//ETS1//FOXJ3//FOXF2//FLI1//EPC2//LHX6//HBP1//ATAD2//GRHL1//MNX1//HOXA3//HOXD1//FOXN2//BOLA3//NR3C2//LHX8//MYBL1//NEUROG1//NFE2//NHLH2//ARID4B//PLEK//BRWD1//POU3F2//TRERF1//ZNFX1//MIER1//RAD21//ARID4A//RBL2//DMRTA1//NKX2-4//RFX7//SMARCD2//TEF//DEK//BRD3//FOSL1//LCOR//KLF7//CBFB//POLR3G//BDP1//KAT2B//G3BP2//TOPORS//MAP3K2//KLF6//EGR2//MAPRE3//BAMBI//RGMB//MDFIC//IGF1//IRF1//LHX1//LRP6//MAP3K5//NEUROD1//NPAS2//OTX2//MAPK1//ZNF287//RNF4//TEAD1//TGFB1I1//WNT7A//FUBP3//ACVR1//HAND2//EREG//DKK3//NRG1//PAX9//RASD1//ATXN1//TSG101//BTAF1//PPM1F//TOB1//SOSTDC1//TMEM100//RGMA//BMP3//BMPR2//TGFB2//SULF1//CTDSPL2//CMKLR1//EPHA5//EGLN1//FZD6//FASTK//ESRP1//AHNAK//DCP2//YTHDF2//FXR1//CDK8//CREBRF//NKX2-3//E2F3//E2F5//S1PR1//EZH1//DLL1//IL2//ITGA6//MEF2D//MEOX2//MET//PPP1R12A//NCK1//OSM//P2RY1//PBX3//IER5//PPP3R1//DCAF6//SHOX//CAPRIN2//STAT4//PCGF5//CCNC//ST18//PTEN//MTPN//QKI//EGLN3//NANOS1//PRKAA1//RAP2C//TOB2//FBXW7//NPNT//NOVA1// |
| GO:2001141 | regulation of RNA biosynthetic process | Biological process | 288 | 3774 | 934 | 17653 | 1.44232202980156 | 2.42353843952567e-12 | 6.30399633327389e-10 | 11.6155500875808 | 0.308351177730193 | E2F1//NPAT//RRM2//TSHZ1//HMG20A//KLF2//ZBTB18//CEBPB//ZNF268//TCERG1//WWP2//KLF12//OSR1//CRY2//GLIS3//ARX//DNMT3B//DR1//EDN1//EFNA1//CC2D1B//EN1//ESR1//FHL2//CPEB3//FOXF1//FOXC1//MYT1L//CIC//ZFPM2//DNAJB5//GATA6//CNOT7//HSBP1//AR//JARID2//JUN//ZNF568//MAF//MDM4//MYB//NFIA//NFIB//NR4A2//RLIM//KLF3//ZBTB7A//PIAS4//POU4F1//PPARG//CC2D1A//TSHZ3//ZBTB4//RARB//RBBP7//RBBP8//REST//RNF2//PRDM16//SOX17//SOX11//STAT3//ZEB1//TCF21//TFAP2A//TFAP2C//TGIF1//KLF10//TNF//UBE2I//VLDLR//WFS1//ZNF217//LRP8//BTG2//BHLHE41//TBL1XR1//NR4A3//HMGA2//ARID5B//BRMS1L//BHLHE40//RUNX3//URI1//PER2//SQSTM1//NOG//KLF4//HAND1//FNIP1//PHF14//USP3//PKD2//THRA//BAHD1//H2AFZ//KMT2B//DPY30//HIPK3//KHDRBS3//ZMYND11//NUP35//ZNF597//CTNND2//ZNF782//ZNF800//ELAVL2//ZBTB9//KDM2A//MYCBP2//FLII//CNOT6L//NR5A2//GTF2E1//SERTAD3//NRBF2//HIVEP2//HMGB3//ZBTB41//ZBTB34//ATXN3//MLLT6//RNF141//BNC2//PPP2CA//ZNF532//CDCA7L//LMO3//SLC2A4RG//PAK6//PHTF2//ZNF630//CNOT6//ZNF512B//ZBTB26//BCL11B//SNAPC1//SOX4//BTF3//BTG1//TMF1//ZNF2//ZNF3//ZNF131//ZNF655//SNIP1//TCF7L1//WASL//RPS6KA4//RPS6KA5//ZBTB47//CLOCK//CDKN1A//IRF9//TADA1//CRK//ZNF367//ELK3//EMX2//ERG//ETS1//FOXJ3//FOXF2//FLI1//EPC2//LHX6//HBP1//ATAD2//GRHL1//MNX1//HOXA3//HOXD1//FOXN2//BOLA3//NR3C2//LHX8//MYBL1//NEUROG1//NFE2//NHLH2//ARID4B//PLEK//BRWD1//POU3F2//TRERF1//ZNFX1//MIER1//RAD21//ARID4A//RBL2//DMRTA1//NKX2-4//RFX7//SMARCD2//TEF//DEK//BRD3//FOSL1//LCOR//KLF7//CBFB//POLR3G//BDP1//KAT2B//G3BP2//TOPORS//MAP3K2//KLF6//EGR2//MAPRE3//BAMBI//RGMB//MDFIC//IGF1//IRF1//LHX1//LRP6//MAP3K5//NEUROD1//NPAS2//OTX2//MAPK1//ZNF287//RNF4//TEAD1//TGFB1I1//WNT7A//FUBP3//ACVR1//HAND2//EREG//DKK3//NRG1//PAX9//RASD1//ATXN1//TSG101//BTAF1//PPM1F//TOB1//SOSTDC1//TMEM100//RGMA//BMP3//BMPR2//TGFB2//SULF1//CTDSPL2//CMKLR1//EPHA5//EGLN1//FZD6//CDK8//CREBRF//NKX2-3//E2F3//E2F5//S1PR1//EZH1//DLL1//IL2//ITGA6//MEF2D//MEOX2//MET//PPP1R12A//NCK1//OSM//P2RY1//PBX3//IER5//PPP3R1//DCAF6//SHOX//CAPRIN2//STAT4//PCGF5//CCNC//ST18//PTEN//MTPN//EGLN3//PRKAA1//RAP2C//TOB2//SRSF2//FBXW7//NPNT// |
| GO:0010556 | regulation of macromolecule biosynthetic process | Biological process | 328 | 4467 | 934 | 17653 | 1.38780847797002 | 4.52380059780617e-12 | 1.1331282756653e-09 | 11.3444965463595 | 0.351177730192719 | E2F1//NPAT//RRM2//TSHZ1//HMG20A//KLF2//ZBTB18//CEBPB//ZNF268//TCERG1//WWP2//KLF12//OSR1//CRY2//GLIS3//ARX//DNMT3B//DR1//EDN1//EFNA1//CC2D1B//EN1//ESR1//FHL2//CPEB3//FOXF1//FOXC1//MYT1L//CIC//ZFPM2//DNAJB5//GATA6//CNOT7//HSBP1//AR//JARID2//JUN//ZNF568//MAF//MDM4//MYB//NFIA//NFIB//NR4A2//RLIM//KLF3//ZBTB7A//PIAS4//POU4F1//PPARG//CC2D1A//TSHZ3//ZBTB4//RARB//RBBP7//RBBP8//REST//RNF2//PRDM16//SOX17//SOX11//STAT3//ZEB1//TCF21//TFAP2A//TFAP2C//TGIF1//KLF10//TNF//UBE2I//VLDLR//WFS1//ZNF217//LRP8//BTG2//BHLHE41//TBL1XR1//NR4A3//HMGA2//ARID5B//BRMS1L//BHLHE40//RUNX3//URI1//PER2//SQSTM1//NOG//KLF4//HAND1//FNIP1//PHF14//USP3//SECISBP2L//PKD2//THRA//ESCO2//PPP2CA//BAHD1//H2AFZ//KMT2B//DPY30//HIPK3//KHDRBS3//ZMYND11//NUP35//ZNF597//CTNND2//ZNF782//ZNF800//ELAVL2//ZBTB9//KDM2A//MYCBP2//FLII//CNOT6L//NR5A2//GTF2E1//SERTAD3//NRBF2//HIVEP2//HMGB3//ZBTB41//ZBTB34//ATXN3//MLLT6//RNF141//BNC2//ZNF532//CDCA7L//LMO3//SLC2A4RG//PAK6//PHTF2//ZNF630//CNOT6//ZNF512B//ZBTB26//BCL11B//SNAPC1//SOX4//BTF3//BTG1//TMF1//ZNF2//ZNF3//ZNF131//ZNF655//SNIP1//TCF7L1//WASL//RPS6KA4//RPS6KA5//ZBTB47//CLOCK//CDKN1A//IRF9//TADA1//CRK//ZNF367//ELK3//EMX2//ERG//ETS1//FOXJ3//FOXF2//FLI1//EPC2//LHX6//HBP1//ATAD2//GRHL1//MNX1//HOXA3//HOXD1//FOXN2//BOLA3//NR3C2//LHX8//MYBL1//NEUROG1//NFE2//NHLH2//ARID4B//PLEK//BRWD1//POU3F2//TRERF1//ZNFX1//MIER1//RAD21//ARID4A//RBL2//DMRTA1//NKX2-4//RFX7//SMARCD2//TEF//DEK//BRD3//FOSL1//LCOR//KLF7//CBFB//POLR3G//BDP1//MTPN//DDX6//MKNK2//ACO1//TSC1//KBTBD8//KAT2B//G3BP2//NF2//IGF1//FAM129A//FMR1//SERP1//PRR16//MAPK1//FXR1//TOPORS//MAP3K2//KLF6//EGR2//MAPRE3//BAMBI//RGMB//MDFIC//IRF1//LHX1//LRP6//MAP3K5//NEUROD1//NPAS2//OTX2//ZNF287//RNF4//TEAD1//TGFB1I1//WNT7A//FUBP3//ACVR1//HAND2//EREG//DKK3//NRG1//PAX9//RASD1//ATXN1//TSG101//BTAF1//PPM1F//TOB1//NANOS1//CAPRIN2//SOSTDC1//TMEM100//RGMA//BMP3//BMPR2//TGFB2//SULF1//CTDSPL2//CMKLR1//DCP2//CCT6A//EPHA5//FBXW7//NCK1//RNF128//ERRFI1//ITM2B//EGLN1//FZD6//ITGAV//TMEFF2//MAP2K4//CDK8//CREBRF//NKX2-3//E2F3//E2F5//S1PR1//EZH1//DLL1//IL2//ITGA6//MEF2D//MEOX2//MET//PPP1R12A//OSM//P2RY1//PBX3//IER5//PPP3R1//DCAF6//SHOX//STAT4//PCGF5//CCNC//ST18//PAIP2//PRKAA1//INHBB//PTEN//KDR//CCL2//PTGES3//NUP43//YTHDF2//QKI//EGLN3//ORMDL3//CPEB2//RAB3GAP1//RAP2C//TOB2//SRSF2//PPP1R15B//DUSP1//GJA1//NSMAF//NPNT//CPEB1//CPEB4//UNK// |
| GO:0006355 | regulation of transcription, DNA-templated | Biological process | 285 | 3745 | 934 | 17653 | 1.43835034864473 | 4.71635311529813e-12 | 1.13916771852719e-09 | 11.3263936865531 | 0.305139186295503 | E2F1//NPAT//RRM2//TSHZ1//HMG20A//KLF2//ZBTB18//CEBPB//ZNF268//TCERG1//WWP2//KLF12//OSR1//CRY2//GLIS3//ARX//DNMT3B//DR1//EDN1//EFNA1//CC2D1B//EN1//ESR1//FHL2//CPEB3//FOXF1//FOXC1//MYT1L//CIC//ZFPM2//DNAJB5//GATA6//CNOT7//HSBP1//AR//JARID2//JUN//ZNF568//MAF//MDM4//MYB//NFIA//NFIB//NR4A2//RLIM//KLF3//ZBTB7A//PIAS4//POU4F1//PPARG//CC2D1A//TSHZ3//ZBTB4//RARB//RBBP7//RBBP8//REST//RNF2//PRDM16//SOX17//SOX11//STAT3//ZEB1//TCF21//TFAP2A//TFAP2C//TGIF1//KLF10//TNF//UBE2I//VLDLR//WFS1//ZNF217//LRP8//BTG2//BHLHE41//TBL1XR1//NR4A3//HMGA2//ARID5B//BRMS1L//BHLHE40//RUNX3//URI1//PER2//SQSTM1//NOG//KLF4//HAND1//FNIP1//PHF14//USP3//PKD2//THRA//BAHD1//H2AFZ//KMT2B//DPY30//CDKN1A//IRF9//TADA1//CRK//ZNF367//ELK3//EMX2//ERG//ETS1//FOXJ3//FOXF2//FLI1//EPC2//LHX6//HBP1//ATAD2//GTF2E1//GRHL1//HIVEP2//MNX1//HOXA3//HOXD1//FOXN2//BOLA3//NR3C2//LHX8//MYBL1//NEUROG1//NFE2//NHLH2//ARID4B//PLEK//BRWD1//POU3F2//TRERF1//SLC2A4RG//ZNFX1//MIER1//RAD21//ARID4A//RBL2//DMRTA1//NKX2-4//RFX7//SMARCD2//TEF//ZNF3//ZNF131//DEK//BRD3//FOSL1//TCF7L1//LCOR//KLF7//CBFB//CLOCK//POLR3G//BDP1//KAT2B//G3BP2//TOPORS//MAP3K2//KLF6//EGR2//MAPRE3//NR5A2//BAMBI//RGMB//SERTAD3//MDFIC//IGF1//IRF1//LHX1//LRP6//MAP3K5//NEUROD1//NPAS2//OTX2//MAPK1//ZNF287//RNF4//SOX4//TEAD1//TGFB1I1//WNT7A//FUBP3//ACVR1//HAND2//EREG//DKK3//NRG1//PAX9//RASD1//ATXN1//TSG101//BTAF1//RPS6KA5//PPM1F//TOB1//SOSTDC1//TMEM100//RGMA//BMP3//BMPR2//TGFB2//SULF1//CTDSPL2//CMKLR1//EPHA5//RPS6KA4//ZMYND11//EGLN1//FZD6//CDK8//CREBRF//NKX2-3//E2F3//E2F5//S1PR1//EZH1//DLL1//IL2//ITGA6//MEF2D//MEOX2//MET//PPP1R12A//NCK1//OSM//P2RY1//PBX3//IER5//PPP3R1//DCAF6//SHOX//BCL11B//CAPRIN2//STAT4//PCGF5//CCNC//ST18//PTEN//MTPN//EGLN3//PRKAA1//FBXW7//NPNT//HIPK3//KHDRBS3//NUP35//ZNF597//CTNND2//ZNF782//ZNF800//ELAVL2//ZBTB9//KDM2A//MYCBP2//FLII//CNOT6L//NRBF2//HMGB3//ZBTB41//ZBTB34//ATXN3//MLLT6//RNF141//BNC2//PPP2CA//ZNF532//CDCA7L//LMO3//PAK6//PHTF2//ZNF630//CNOT6//ZNF512B//ZBTB26//SNAPC1//BTF3//BTG1//TMF1//ZNF2//ZNF655//SNIP1//WASL//ZBTB47// |
| GO:0048519 | negative regulation of biological process | Biological process | 376 | 5303 | 934 | 17653 | 1.34010202297516 | 5.50765766144278e-12 | 1.2844237504944e-09 | 11.2590330619458 | 0.402569593147752 | E2F1//USP28//CLOCK//TSHZ1//HMG20A//KLF2//ZBTB18//CEBPB//ZNF268//TCERG1//WWP2//KLF12//OSR1//CRY2//GLIS3//ARX//DNMT3B//DR1//EDN1//EFNA1//CC2D1B//EN1//ESR1//FHL2//CPEB3//FOXF1//FOXC1//MYT1L//CIC//ZFPM2//DNAJB5//GATA6//CNOT7//HSBP1//AR//JARID2//JUN//ZNF568//MAF//MDM4//MYB//NFIA//NFIB//NR4A2//RLIM//KLF3//ZBTB7A//PIAS4//POU4F1//PPARG//CC2D1A//TSHZ3//ZBTB4//RARB//RBBP7//RBBP8//REST//RNF2//PRDM16//SOX17//SOX11//STAT3//ZEB1//TCF21//TFAP2A//TFAP2C//TGIF1//KLF10//TNF//UBE2I//VLDLR//WFS1//ZNF217//LRP8//BTG2//BHLHE41//TBL1XR1//NR4A3//HMGA2//ARID5B//BRMS1L//BHLHE40//RUNX3//URI1//PER2//SQSTM1//NOG//KLF4//HAND1//FNIP1//PHF14//USP3//DCP2//PPP2CA//RPL15//DUSP10//SPRED1//DUSP1//DUSP2//DUSP5//RGS3//CNOT6L//PAN3//CNOT6//RPS6KA4//RPS6KA5//FAM129A//IL2//LRP6//PTEN//PPP1R15B//SLIT2//SULF1//GJA1//NF2//NME1//BMPR2//PKD2//THRA//EPS8//TWF1//ERRFI1//TSG101//SOCS5//BAHD1//H2AFZ//DPY30//GADD45A//PPM1E//FLRT1//GNAQ//NCK1//TAOK3//ASPN//DUS2//YWHAG//CAMK2N2//PPM1F//CDKN1A//RBL2//SOX4//STMN2//MID1IP1//CDK6//HBP1//MTBP//IRF1//PRKAA1//RRAGD//RHEB//TGFB2//TSC1//LAMTOR3//KAT2B//NABP1//ADAM10//MIA3//G3BP2//HIPK3//TOPORS//EPHA2//EPHA4//EPHA5//EPHA7//ARL6IP1//DSTYK//IGF1//KDR//MET//ZFAND6//SPHK2//BCL11B//BNIP3//ACTC1//TMF1//WNT7A//BAG6//CCND2//TOB1//GDF11//DLC1//TOB2//COPS8//BTG3//EREG//ETS1//FRZB//TES//DLL1//ASPH//OSM//PTHLH//STRN//BTG1//TGFB1I1//FOSL1//ST18//HS3ST5//RGS17//ACVR1//SNIP1//SOSTDC1//MTMR9//MYBL1//ITGB8//LDLR//POU3F2//NDFIP2//HAND2//PFN2//PTPRG//P2RY1//LDLRAD4//ITGAV//C5ORF30//CALM2//SPOCK1//FBXW7//PLEK//PPP1R37//PPP1R12A//PPP1R12C//PLEKHF2//SERPINI1//RECK//RAD21//SKP1//LPAR1//MYLIP//BAG5//PMEPA1//IQGAP2//FZD10//CPEB2//LRCH1//IPO5//KMT2B//ELK3//FOXF2//DKK3//MDFIC//NRG1//LHX1//PAX9//RASD1//ARID4A//ATXN1//BTAF1//INHBB//FMR1//NANOS1//CAPRIN2//FXR1//APCDD1//SHISA2//SERTAD3//ADIPOR2//TMEFF2//CLIC4//RAP2C//BAMBI//RASL11B//TGFBR2//CTDSPL2//EGLN1//LIMA1//TNFRSF21//SPOPL//ARRDC3//ATAD2//MIER1//CMKLR1//SLC6A1//NPLOC4//MASTL//CD274//PDCD1LG2//CD2AP//RNF4//PTGER4//CCL2//ADRB1//RNF128//EPS15//GRB2//EPN1//MARCH7//ITM2B//LYPLA1//PKDCC//ZMYND11//PXK//FZD6//YTHDF2//AIDA//NTF3//CPEB4//DDX6//HMGB3//MAPK1//ZADH2//CXCL14//TMEM64//CEP97//LATS2//UGT1A8//UGT1A1//PAIP2//CRK//NEUROD1//MDM1//MORC3//OGN//ULK2//RGMA//SEMA4B//ARHGAP12//DRD1//PRKACB//S1PR1//ARHGAP6//PPFIA1//TMOD3//TMOD1//OTUD3//PLEKHA1//GLRA1//SLC24A2//GRIA1//NPAS2//NUP35//NUP43//QKI//RRM2//E2F3//RANBP9//LMO3//CCL8//CDC14A//MEOX2//SACS//AMER2//EFNA5//PLAA//ORMDL3//CREBRF//NEUROG1//BTBD10//GNB5//CRTAP//CHMP3//FEZ2//NPAT//SRSF2//WASL//DNAJB9//NAPEPLD//DERL2//SESN3//SESN1//FRY//DOCK5//CTSA//SESTD1//KLHL15//ZNF655//LYPD6//PRKAR2B//HECW2//MAP2K4//CPEB1//UNK//ITGA5//MTPN//ARHGAP1//ITGA6// |
| GO:0009891 | positive regulation of biosynthetic process | Biological process | 164 | 1841 | 934 | 17653 | 1.68368834087237 | 7.54718918215906e-12 | 1.70138801463139e-09 | 11.1222147635984 | 0.17558886509636 | KMT2B//KAT2B//MTPN//IGF1//CNEP1R1//LDLR//EDN1//FAM129A//CPEB3//FMR1//SERP1//PRR16//MAPK1//SOX4//FXR1//TOPORS//KLF2//MAP3K2//KLF6//E2F1//EGR2//ESR1//ETS1//FHL2//MAPRE3//FOXF1//FOXF2//FOXC1//FLI1//NR5A2//BAMBI//GATA6//RGMB//ATAD2//SERTAD3//MDFIC//FOXN2//IRF1//AR//JUN//LHX1//LRP6//MAP3K5//MYB//MYBL1//NEUROD1//NFE2//NPAS2//NPAT//OTX2//PPARG//TRERF1//ZNF287//REST//RNF4//PRDM16//SOX17//SOX11//STAT3//TEAD1//TFAP2A//TGFB1I1//TNF//WNT7A//TBL1XR1//HMGA2//RUNX3//FUBP3//ACVR1//KLF4//HAND2//CLOCK//ADRB1//PTHLH//CALM2//CCT6A//P2RY1//NCK1//EREG//PKD2//PRKAA1//MID1IP1//TGFB2//MAP2K4//CDK8//ZBTB18//CEBPB//ZNF268//WWP2//OSR1//CREBRF//NKX2-3//GLIS3//E2F3//E2F5//S1PR1//ELK3//EN1//ERG//EZH1//FOXJ3//ZFPM2//DLL1//GRHL1//CNOT7//H2AFZ//NRG1//IL2//ITGA6//MAF//MEF2D//MEOX2//MET//PPP1R12A//NEUROG1//NFIA//NFIB//NHLH2//NR4A2//OSM//PAX9//PBX3//IER5//ARID4B//POU3F2//POU4F1//EGLN1//PPP3R1//DCAF6//RGMA//RAD21//RARB//ARID4A//SHOX//BCL11B//BMPR2//CAPRIN2//STAT4//ZEB1//TCF21//TEF//TFAP2C//THRA//KLF10//NR4A3//FOSL1//PCGF5//KLF7//CBFB//SQSTM1//CCNC//RPS6KA4//NOG//RPS6KA5//HAND1//ST18//KDR//CCL2//PTGES3//RAB3GAP1//YTHDF2//NSMAF//FBXW7//NPNT// |
| GO:0048522 | positive regulation of cellular process | Biological process | 365 | 5142 | 934 | 17653 | 1.34162899978928 | 1.30341813357971e-11 | 2.84355381851599e-09 | 10.8849162412514 | 0.390792291220557 | GADD45A//MINK1//TNF//MAP3K2//CRK//CRKL//MAP3K5//TAOK3//LAMTOR3//ADAM9//DUSP5//LPAR1//LPAR3//NRG1//IGF1//NTF3//PIK3CB//PRKAA1//MAPK1//MAP2K4//EPS15//NSF//FAM129A//EFNA1//EFNA5//EPHA7//FMR1//NPTN//KDR//P2RY1//AKTIP//SQSTM1//CCND2//FNIP1//F3//JUN//BMPR2//VEGFB//VASH2//FOXF1//TGFBR2//MIA3//CCL8//EREG//FBXW7//KMT2B//DLC1//EGLN3//PMAIP1//PPARG//CDKN1A//E2F1//CNOT6L//CNOT7//MDM4//CNOT6//RBL2//SOX4//BTG2//MTBP//KAT2B//COPS8//MDFIC//ADAM10//ZNF268//E2F3//EDN1//ETS1//BAMBI//DLL1//HOXA3//IL2//AR//ITGAV//OSM//DERL2//POU3F2//CDCA7L//SPHK2//PTEN//PTHLH//RARB//SOX11//TGFB2//FOSL1//TOB1//EPS8//ARHGAP1//ARHGAP6//BNIP3//TRPC3//PLEK//WNT7A//PAN3//DOCK5//PPM1F//TMED9//GJA1//GRB2//NPNT//FOXC1//TGFB1I1//CMKLR1//CALM2//THRA//GDF11//INHBB//BMP3//ACVR1//CNEP1R1//LDLR//MTMR9//PCOLCE2//STMN2//NCK1//NME1//SERPINI1//TWF1//ZNF804A//CPEB3//SERP1//PRR16//FXR1//SESN3//RAB12//SESN1//TSC1//TOPORS//KLF2//KLF6//EGR2//ESR1//FHL2//MAPRE3//FOXF2//FLI1//NR5A2//GATA6//RGMB//ATAD2//SERTAD3//FOXN2//IRF1//LHX1//LRP6//MYB//MYBL1//NEUROD1//NFE2//NPAS2//NPAT//OTX2//TRERF1//ZNF287//REST//RNF4//PRDM16//SOX17//STAT3//TEAD1//TFAP2A//TBL1XR1//HMGA2//RUNX3//FUBP3//KLF4//HAND2//CLOCK//SULF1//MTPN//SEMA4B//DRD1//S1PR1//CD274//ITGA6//ITGA5//PTP4A1//ADRB1//CFL2//CDC42EP3//PFN2//MET//SLAIN2//CLIP1//PDCD1LG2//NDFIP2//WFS1//NEUROG1//PKD2//WASF3//MIER1//RAP2C//RRAGD//RHEB//PAK6//SLK//CCT6A//PKDCC//RNF180//ANKIB1//LONRF3//LONRF1//SOCS5//BCL2L11//RHOC//KIF23//PPP1R15B//RPS6KA4//RPS6KA5//CAPRIN2//IFNA1//PIAS4//EPHA2//EPHA4//EPHA5//DSTYK//ADCY3//PRKACB//PRKAR2B//IQGAP2//ARFIP1//NSMF//LMO3//PPP1R12A//SYBU//GAB1//NR4A3//PTGES3//KCTD20//BTBD10//FGD4//DUSP1//CDK19//FRZB//LATS2//USP27X//ARHGEF4//POU4F1//MOAP1//ZMAT3//UBE2Z//SOS1//SOS2//NSMAF//SLIT2//CC2D1A//SLC20A1//UBE2I//FZD10//SPRED1//DUSP10//NF2//CEBPB//CCDC71L//TMEM64//TMEM100//BTG1//DNMT3B//MMD//ZEB1//FAM20C//KLF10//MID1IP1//CCNY//CCNC//GOLGA4//VLDLR//ASPH//RAD21//CDK8//ZBTB18//WWP2//OSR1//CREBRF//NKX2-3//GLIS3//E2F5//ELK3//EN1//ERG//EZH1//FOXJ3//ZFPM2//GRHL1//H2AFZ//MAF//MEF2D//MEOX2//NFIA//NFIB//NHLH2//NR4A2//PAX9//PBX3//IER5//ARID4B//EGLN1//PPP3R1//DCAF6//RGMA//ARID4A//SHOX//BCL11B//STAT4//TCF21//TEF//TFAP2C//PCGF5//KLF7//CBFB//NOG//HAND1//ST18//HPRT1//RASGEF1A//CDK6//RAB21//PTGER4//LRP8//MAN2A1//CCL2//SH3D19//WASL//ARRDC3//ARRDC4//GPM6A//PPM1E//JARID2//SLITRK3//FLRT1//TSHZ3//SLC24A2//SNAP25//C21ORF91//YTHDF2//QKI//RAB3GAP1//DNAJC27//MSN//ACSL1//PPP2CA//MIEF1//DDHD1//NANOS1//PAIP2//DYNLL2//YWHAG//CALCOCO2//FYCO1//TMOD3//SERINC3//FBXL5//TSG101//PLAA//ELMOD1//SNIP1//EDEM1//ATXN3//BAG6//RNF128//MMP2//STK17B//CXCL14//TMF1//PIK3R3// |
| GO:0031328 | positive regulation of cellular biosynthetic process | Biological process | 161 | 1812 | 934 | 17653 | 1.67934268805158 | 1.51051378678816e-11 | 3.1923764812651e-09 | 10.8208753064723 | 0.17237687366167 | KMT2B//KAT2B//IGF1//CNEP1R1//LDLR//FAM129A//CPEB3//FMR1//SERP1//PRR16//MAPK1//SOX4//FXR1//TOPORS//KLF2//MAP3K2//KLF6//E2F1//EGR2//ESR1//ETS1//FHL2//MAPRE3//FOXF1//FOXF2//FOXC1//FLI1//NR5A2//BAMBI//GATA6//RGMB//ATAD2//SERTAD3//MDFIC//FOXN2//IRF1//AR//JUN//LHX1//LRP6//MAP3K5//MYB//MYBL1//NEUROD1//NFE2//NPAS2//NPAT//OTX2//PPARG//TRERF1//ZNF287//REST//RNF4//PRDM16//SOX17//SOX11//STAT3//TEAD1//TFAP2A//TGFB1I1//TNF//WNT7A//TBL1XR1//HMGA2//RUNX3//FUBP3//ACVR1//KLF4//HAND2//CLOCK//ADRB1//PTHLH//CALM2//CCT6A//P2RY1//NCK1//EREG//EDN1//PKD2//MID1IP1//MAP2K4//PRKAA1//CDK8//ZBTB18//CEBPB//ZNF268//WWP2//OSR1//CREBRF//NKX2-3//GLIS3//E2F3//E2F5//S1PR1//ELK3//EN1//ERG//EZH1//FOXJ3//ZFPM2//DLL1//GRHL1//CNOT7//H2AFZ//NRG1//IL2//ITGA6//MAF//MEF2D//MEOX2//MET//PPP1R12A//NEUROG1//NFIA//NFIB//NHLH2//NR4A2//OSM//PAX9//PBX3//IER5//ARID4B//POU3F2//POU4F1//EGLN1//PPP3R1//DCAF6//RGMA//RAD21//RARB//ARID4A//SHOX//BCL11B//BMPR2//CAPRIN2//STAT4//ZEB1//TCF21//TEF//TFAP2C//THRA//KLF10//NR4A3//FOSL1//PCGF5//KLF7//CBFB//SQSTM1//CCNC//RPS6KA4//NOG//RPS6KA5//HAND1//ST18//PTGES3//TGFB2//RAB3GAP1//YTHDF2//NSMAF//FBXW7//NPNT// |
| GO:0009719 | response to endogenous stimulus | Biological process | 147 | 1606 | 934 | 17653 | 1.72998938669497 | 1.600057794312e-11 | 3.27914874634305e-09 | 10.7958643302832 | 0.157387580299786 | THRA//PKD2//COL1A2//JUN//TGFB2//TGFBR2//KLF10//ADAM9//ACVR1//ZFYVE9//CHRM2//CHRM5//GNAQ//GAB1//APPL1//GRB2//SOS1//PIK3R3//SPRED1//FLRT1//ESRP1//MAPK1//FHL2//STEAP2//NR5A2//LATS2//PPARG//RARB//ADIPOR2//DKK3//SOX11//ZEB1//CEBPB//SESN3//CPEB3//NSMF//SESN1//GLRA1//IPO5//MMP2//RRAGD//CPEB1//TNF//CPEB4//LAMTOR3//TOB1//SOSTDC1//RGMB//TMEM100//RGMA//BMP3//BMPR2//NOG//NPNT//TGFB1I1//BAMBI//ASPN//PMEPA1//PRDM16//RASL11B//LDLRAD4//SULF1//GATA6//CTDSPL2//ESR1//AR//RNF4//DNMT3B//DUSP1//ETS1//GJB2//PTEN//RBBP8//SLC6A1//STAT3//WNT7A//FOSL1//EGR2//IGF1//TSC1//CPEB2//INHBB//ERRFI1//SLC2A4//YWHAG//KAT2B//SLIT2//UBA5//CBFB//ACSL1//EDN1//NME1//NTF3//OTX2//FAM20C//SHISA2//PGRMC2//NR3C2//NR4A2//NR4A3//EREG//GJA1//LRP6//BTG1//BTG2//DSTYK//LHX1//CCL2//NPTN//NCK1//IDH1//RBBP7//CDKN1A//TCF21//CRK//RAP1B//HCN2//FOXC1//EFNA5//ADCY3//PRKACB//PRKAR2B//PRKAA1//PTGER4//URI1//BCL2L11//UGT1A1//REST//H2AFZ//POU4F1//MSN//LDLR//SOCS5//KLF2//KLF4//FIBIN//RUNX3//DRD1//CREBRF//KLF3//EPHA4//STMN2//DYNC1LI2//E2F1//CRY2//CLOCK//LMO3// |
| GO:0045935 | positive regulation of nucleobase-containing compound metabolic process | Biological process | 156 | 1742 | 934 | 17653 | 1.69257566556937 | 1.84280038633215e-11 | 3.52801932283366e-09 | 10.7345217053721 | 0.167023554603854 | KMT2B//KAT2B//TOPORS//KLF2//MAP3K2//KLF6//E2F1//EGR2//ESR1//ETS1//FHL2//MAPRE3//FOXF1//FOXF2//FOXC1//FLI1//NR5A2//BAMBI//GATA6//RGMB//ATAD2//SERTAD3//MDFIC//FOXN2//IGF1//IRF1//AR//JUN//LHX1//LRP6//MAP3K5//MYB//MYBL1//NEUROD1//NFE2//NPAS2//NPAT//OTX2//PPARG//TRERF1//MAPK1//ZNF287//REST//RNF4//PRDM16//SOX17//SOX4//SOX11//STAT3//TEAD1//TFAP2A//TGFB1I1//TNF//WNT7A//TBL1XR1//HMGA2//RUNX3//FUBP3//ACVR1//KLF4//HAND2//CLOCK//CALM2//ADRB1//PTHLH//CCT6A//EREG//MAP2K4//PRKAA1//CDK8//ZBTB18//CEBPB//ZNF268//WWP2//OSR1//CREBRF//NKX2-3//GLIS3//E2F3//E2F5//S1PR1//EDN1//ELK3//EN1//ERG//EZH1//FOXJ3//ZFPM2//DLL1//GRHL1//CNOT7//H2AFZ//NRG1//IL2//ITGA6//MAF//MEF2D//MEOX2//MET//PPP1R12A//NCK1//NEUROG1//NFIA//NFIB//NHLH2//NR4A2//OSM//P2RY1//PAX9//PBX3//IER5//ARID4B//PKD2//POU3F2//POU4F1//EGLN1//PPP3R1//DCAF6//RGMA//RAD21//RARB//ARID4A//SHOX//BCL11B//BMPR2//CAPRIN2//STAT4//ZEB1//TCF21//TEF//TFAP2C//THRA//KLF10//NR4A3//FOSL1//PCGF5//KLF7//CBFB//SQSTM1//CCNC//RPS6KA4//NOG//RPS6KA5//HAND1//ST18//PTGES3//TOB1//CPEB3//BTG2//CNOT6L//YTHDF2//QKI//NANOS1//TGFB2//FBXW7//NPNT// |
| GO:0022008 | neurogenesis | Biological process | 142 | 1536 | 934 | 17653 | 1.74730521725553 | 1.84951588092424e-11 | 3.52801932283366e-09 | 10.7329419352498 | 0.15203426124197 | STAT3//DCX//GJA1//GPM6A//LRP12//NR4A2//SPOCK1//UNK//SOX4//SOX11//HAND2//NTNG1//SLITRK3//NEFH//OGN//POU4F1//DSCAML1//WNT7A//SLIT2//RANBP9//ARX//DPYSL2//EFNA1//EFNA5//EPHA5//GAB1//GRB2//KIF5A//OTX2//PIK3CB//ENAH//MAPK1//NTN4//SOS1//VLDLR//NR4A3//KLF7//RPS6KA5//FEZ2//EGR2//EPHA4//LHX1//NOG//P2RY1//TGFB2//NFIB//PTEN//SNAP25//ZNF804A//STMN2//NPTN//NCK1//NME1//SERPINI1//TWF1//LPAR1//MYLIP//PTPRG//TSC1//BAG5//CDK6//DRD1//DLL1//POU3F2//WASF3//NF2//GNAQ//FLRT1//STRN//MNX1//MTPN//BCL11B//NRG1//LHX6//LHX8//HPRT1//C21ORF91//BTG2//KCNJ10//PPP3R1//CEBPB//FZD10//S1PR1//EMX2//MYT1L//HOXD1//NEUROG1//RUNX3//RAB21//JUN//FRY//PSD//EPHA7//TOPORS//NEUROD1//APCDD1//CCL2//NTF3//YWHAG//HMG20A//DDX6//REST//DNMT3B//MMD//RARB//ZEB1//GOLGA4//EN1//FMR1//PBX3//FXR1//ULK2//LPAR3//KLF4//RGMA//TCF7L1//DUSP10//BNIP3//TNFRSF21//PPARG//RHEB//MINK1//NSMF//HECW2//BMPR2//SEMA4B//PER2//MAN2A1//CAPRIN2//MYB//SLC12A5//CTNND2//WASL//CPEB3//IL2//LRP8//NEFM//LDLR//LRP6//NANOS1//PLAA//PRDM16// |
| GO:0010628 | positive regulation of gene expression | Biological process | 165 | 1877 | 934 | 17653 | 1.66146545754479 | 1.87799342927712e-11 | 3.52801932283366e-09 | 10.7263059315767 | 0.176659528907923 | KMT2B//KAT2B//FAM129A//CPEB3//FMR1//SERP1//PRR16//MAPK1//SOX4//FXR1//TOPORS//KLF2//MAP3K2//KLF6//E2F1//EGR2//ESR1//ETS1//FHL2//MAPRE3//FOXF1//FOXF2//FOXC1//FLI1//NR5A2//BAMBI//GATA6//RGMB//ATAD2//SERTAD3//MDFIC//FOXN2//IGF1//IRF1//AR//JUN//LHX1//LRP6//MAP3K5//MYB//MYBL1//NEUROD1//NFE2//NPAS2//NPAT//OTX2//PPARG//TRERF1//ZNF287//REST//RNF4//PRDM16//SOX17//SOX11//STAT3//TEAD1//TFAP2A//TGFB1I1//TNF//WNT7A//TBL1XR1//HMGA2//RUNX3//FUBP3//ACVR1//KLF4//HAND2//CLOCK//NCK1//DEK//CDK8//ZBTB18//CEBPB//ZNF268//WWP2//OSR1//CREBRF//NKX2-3//GLIS3//E2F3//E2F5//S1PR1//EDN1//ELK3//EN1//ERG//EZH1//FOXJ3//ZFPM2//DLL1//GRHL1//CNOT7//H2AFZ//NRG1//IL2//ITGA6//MAF//MEF2D//MEOX2//MET//PPP1R12A//NEUROG1//NFIA//NFIB//NHLH2//NR4A2//OSM//P2RY1//PAX9//PBX3//IER5//ARID4B//PKD2//POU3F2//POU4F1//EGLN1//PPP3R1//DCAF6//RGMA//RAD21//RARB//ARID4A//SHOX//BCL11B//BMPR2//CAPRIN2//STAT4//ZEB1//TCF21//TEF//TFAP2C//THRA//KLF10//NR4A3//FOSL1//PCGF5//KLF7//CBFB//SQSTM1//CCNC//RPS6KA4//NOG//RPS6KA5//HAND1//ST18//TOB1//TGFB2//PRKAA1//YTHDF2//FBXW7//NPNT//ARX//DNMT3B//DRD1//RAB3GAP1//GJA1//ITGB8//LDLR//MSN//PIK3CB//PTEN//ACTC1//ZNF804A//QKI//PPM1F// |
| GO:0071495 | cellular response to endogenous stimulus | Biological process | 129 | 1350 | 934 | 17653 | 1.80604092315013 | 1.99311696839719e-11 | 3.64309461007303e-09 | 10.7004672134727 | 0.138115631691649 | THRA//COL1A2//JUN//TGFB2//TGFBR2//KLF10//ADAM9//ACVR1//ZFYVE9//CHRM2//CHRM5//GNAQ//GAB1//APPL1//GRB2//SOS1//PIK3R3//SPRED1//FLRT1//ESRP1//MAPK1//NR5A2//LATS2//PPARG//RARB//ADIPOR2//DKK3//SOX11//ZEB1//CEBPB//SESN3//CPEB3//NSMF//SESN1//GLRA1//IPO5//MMP2//RRAGD//CPEB1//TNF//CPEB4//LAMTOR3//TOB1//SOSTDC1//RGMB//TMEM100//RGMA//BMP3//BMPR2//NOG//NPNT//TGFB1I1//BAMBI//ASPN//PMEPA1//PRDM16//RASL11B//LDLRAD4//SULF1//GATA6//CTDSPL2//ESR1//FHL2//AR//RNF4//CPEB2//INHBB//ERRFI1//PTEN//SLC2A4//YWHAG//KAT2B//DUSP1//STAT3//SLIT2//UBA5//CBFB//ACSL1//NTF3//OTX2//FAM20C//SHISA2//PGRMC2//NR3C2//NR4A2//NR4A3//DSTYK//LHX1//CCL2//NPTN//NCK1//TSC1//IGF1//TCF21//PKD2//RAP1B//HCN2//FOXC1//EFNA5//GJA1//EDN1//ADCY3//GJB2//PRKACB//PRKAR2B//PRKAA1//PTGER4//URI1//BCL2L11//UGT1A1//REST//H2AFZ//POU4F1//MSN//KLF2//KLF4//DNMT3B//CRK//WNT7A//DRD1//CREBRF//KLF3//EPHA4//STMN2//DYNC1LI2//E2F1//CRY2//CLOCK//LMO3// |
| GO:0007399 | nervous system development | Biological process | 192 | 2296 | 934 | 17653 | 1.58052361802297 | 2.42259186634984e-11 | 4.31157599792736e-09 | 10.6157197452195 | 0.205567451820128 | STAT3//DCX//GJA1//GPM6A//LRP12//NR4A2//SPOCK1//UNK//SOX4//SOX11//DLC1//LRP6//PRKACB//TGFB2//TSC1//FZD6//NOG//HAND2//NTNG1//SLITRK3//NEFH//OGN//POU4F1//DSCAML1//WNT7A//SLIT2//RANBP9//ARX//DPYSL2//EFNA1//EFNA5//EPHA5//GAB1//GRB2//KIF5A//OTX2//PIK3CB//ENAH//MAPK1//NTN4//SOS1//VLDLR//NR4A3//KLF7//RPS6KA5//FEZ2//DRD1//NRG1//PTEN//HAPLN1//FMR1//JARID2//NHLH2//NPAS2//SERPINI1//ZEB1//FXR1//B3GNT5//BCL2L11//S1PR1//EGR2//EPHA7//RAB3GAP1//CIC//SPHK2//SLC17A7//PTPRG//RARB//ATXN1//BMPR2//TGFBR2//THRA//BAG6//IMMP2L//NTF3//EPHA4//LHX1//P2RY1//CLDN11//NFIB//SNAP25//ZNF804A//STMN2//NPTN//NCK1//NME1//TWF1//LPAR1//MYLIP//BAG5//CDK6//DLL1//POU3F2//WASF3//NF2//GNAQ//FLRT1//STRN//PKD2//GDF11//PBX3//MNX1//LRP8//EMX2//NEUROD1//BTG2//EN1//FOXC1//NEUROG1//TFAP2A//HOXA3//MTPN//HPRT1//CALM2//SLC32A1//EZH1//BCL11B//LHX6//ETS1//LHX8//C21ORF91//SOX17//TACC2//BNIP3//PRDM16//TCF7L1//KCNJ10//PPP3R1//CEBPB//FZD10//MYT1L//HOXD1//RUNX3//RAB21//E2F1//JUN//FRY//PSD//TNFRSF21//PIKFYVE//TOPORS//SBF2//QKI//APCDD1//CCL2//YWHAG//HMG20A//DDX6//REST//DNMT3B//MMD//GOLGA4//ULK2//LPAR3//KLF4//RGMA//DUSP10//PPARG//RHEB//MINK1//NSMF//HECW2//SEMA4B//PER2//MAN2A1//CAPRIN2//NFIA//SYBU//MYB//SULF1//LRIG1//SLC12A5//CTNND2//WASL//CPEB3//IL2//NEFM//LDLR//NANOS1//PLAA//TRIM3//FUT9//CHRM2//SCRG1//RBFOX3//GLRB//MBNL1//MEF2D//ATXN3//TAOK3//ZIC5//ITM2B// |
| GO:0050793 | regulation of developmental process | Biological process | 209 | 2563 | 934 | 17653 | 1.54123663967797 | 2.51250988074347e-11 | 4.35694982653028e-09 | 10.5998922216535 | 0.223768736616702 | MARCH7//TGFBR2//NME1//BMPR2//HAND2//SOX17//DLC1//CDC42EP3//WASF3//PALM2//FGD4//CRK//FGD5//LPAR1//EPS8//BAMBI//KDR//RHOC//RND3//MSN//BRWD1//CCL2//WASL//SOSTDC1//BNIP3//FOXC1//TGFB1I1//TGFB2//EFNA1//PPP2CA//PTEN//LDLRAD4//FRZB//ITGAV//PPARG//THRA//BHLHE41//SNAP25//ZNF804A//STMN2//NPTN//NCK1//SERPINI1//TWF1//EPHA4//MYLIP//PTPRG//SPOCK1//TSC1//BAG5//NF2//DLL1//MTPN//SPRED1//SULF1//KLF4//LHX1//SH3D19//EFNA5//S1PR1//GJA1//OSR1//FAM20C//TFAP2A//ACVR1//PKDCC//DPYSL2//RAB21//KIAA1109//WNT7A//RARB//TNFRSF21//NRG1//MAF//PTHLH//SOS1//ZEB1//ADAM9//IGF1//STAT3//ADRB1//POU3F2//OTX2//PAX9//EDN1//HMGB3//IL2//CBFB//IRF1//DUSP10//PLEKHB2//RUNX3//CDK6//GDF11//MAPK1//ZNF268//AR//JUN//NEUROD1//E2F1//ZFPM2//ZADH2//TNF//CEBPB//CMKLR1//CCDC71L//TMEM64//LMO3//TMEM100//BTG1//GRHL1//ERRFI1//MYB//SOCS5//GNAQ//ETS1//NFE2//NR4A3//DPY30//KAT2B//KMT2B//CXCL14//MAP3K5//NTF3//BCL11B//YWHAG//HMG20A//DDX6//REST//DNMT3B//MMD//NEUROG1//SOX11//SMOC1//TOB1//NOG//NPNT//TOB2//POU4F1//KLF10//EPHA2//EGLN1//VASH2//F3//GAB1//GATA6//ITGB8//VEGFB//ADAM12//HMGA2//GOLGA4//LAMA3//BCL2L11//LATS2//ARX//SERP1//PRKAA1//EPHA7//ULK2//LPAR3//RGMA//RHEB//FMR1//NSMF//HECW2//FXR1//SEMA4B//PER2//MAN2A1//SLIT2//CAPRIN2//FOXF1//EREG//SLITRK3//FLRT1//JARID2//VANGL1//FZD6//NTN4//ESR1//BTBD7//CPEB3//C21ORF91//LRP8//LDLR//ASPN//MEOX2//TMEM135//MIEF1//DDHD1//PRDM16//VLDLR//CRKL//DOCK5//PRKACB//YTHDF2//NLN//KLF2//EPN1//ITGA5//JAK1//PLAA//LRP6//CLOCK//FBXW7//ERG//NFIB//PHF14//FNIP1// |
| GO:0070887 | cellular response to chemical stimulus | Biological process | 247 | 3175 | 934 | 17653 | 1.47036402569593 | 2.76520257251323e-11 | 4.67526624947674e-09 | 10.5582730477581 | 0.264453961456103 | CBFB//THRA//CCL2//CCL8//SLIT2//PTGES3//UGT1A6//UGT1A9//COL1A2//JUN//TGFB2//TGFBR2//KLF10//ADAM9//ACVR1//ZFYVE9//CHRM2//CHRM5//GNAQ//GAB1//APPL1//GRB2//SOS1//PIK3R3//SPRED1//FLRT1//ESRP1//MAPK1//MAP3K5//NR5A2//LATS2//PPARG//RARB//ADIPOR2//SNIP1//CMKLR1//TNF//PIAS4//ADAM10//DKK3//SOX11//ZEB1//CDKN1A//CRK//CRKL//S1PR1//EREG//F3//IFNA1//IL2//JAK1//MMP2//OSM//PIK3CB//ASPN//STAT3//STAT4//LRP8//SOCS5//CEBPB//SESN3//CPEB3//NSMF//SESN1//GLRA1//IPO5//RRAGD//CPEB1//CPEB4//LAMTOR3//TOB1//SOSTDC1//RGMB//TMEM100//RGMA//BMP3//BMPR2//NOG//NPNT//TGFB1I1//BAMBI//PMEPA1//PRDM16//RASL11B//LDLRAD4//SULF1//GATA6//CTDSPL2//ESR1//FHL2//AR//RNF4//ITGA5//CREBRF//SERP1//DERL2//CPEB2//INHBB//ERRFI1//PTEN//SLC2A4//YWHAG//KAT2B//DUSP1//UBA5//TNFRSF21//ST18//ACSL1//VKORC1L1//GJB2//NR4A2//PKD2//HSPA13//IRF1//EDN1//PPM1E//PPP1R12A//NME1//RAP1B//REST//PPM1F//CD69//GSTA2//MSN//SYBU//KDR//DNAJB9//WFS1//EDEM1//FOXC1//BRWD1//NTF3//OTX2//FAM20C//SHISA2//PGRMC2//NR3C2//NR4A3//DSTYK//LHX1//NPTN//NCK1//TSC1//IGF1//UGT1A10//UGT1A8//UGT1A7//UGT1A5//UGT1A4//UGT1A1//UGT1A3//LPAR1//EPHA2//CXCL14//IRF9//CNOT7//VEGFB//TCF21//EFNA5//EPHA5//EGLN3//EGLN1//KLF2//ETS1//MYB//PRKAA1//PLEKHA1//BNIP3//KLF4//SKP1//SQSTM1//RPS6KA4//RPS6KA5//ATXN3//DNAJB12//UBE2W//CDK19//CD274//PLSCR4//PDCD1LG2//PLAA//MKNK2//CLIC4//TFAP2A//SLC41A1//FZD10//OSR1//EGR2//POU3F2//HCN2//NEUROD1//SOX4//FOXF1//POU4F1//PTPN4//RAB12//ZFAND6//PAX9//TGIF1//URI1//GJA1//ADCY3//PRKACB//PRKAR2B//PTGER4//BCL2L11//H2AFZ//LRP6//E2F1//LDLR//ITGA6//P2RY1//TMF1//DNMT3B//MDM4//PMAIP1//WNT7A//KLHL15//BAG6//DRD1//MAP2K4//FMR1//MPP1//DLL1//MET//KLF3//BAG5//FBXW7//PPP1R15B//EPHA4//STMN2//DYNC1LI2//EPS8//PCOLCE2//JARID2//NEFH//PFKP//PIGA//BCAT2//SOX17//ARID5B//GFPT2//LRCH1//CRY2//CLOCK//LMO3//HMGA2// |
| GO:0006464 | cellular protein modification process | Biological process | 308 | 4192 | 934 | 17653 | 1.38867650424179 | 3.0495377456473e-11 | 4.91048185090778e-09 | 10.5157659867939 | 0.329764453961456 | CDKN1A//GADD45A//PTEN//CCNE2//RANBP9//PPM1L//SPRED1//DUSP5//EREG//RASGEF1A//GRB2//NRG1//IL2//JAK1//MAP3K5//MET//MINK1//TAOK3//MAPK1//CCL2//SOS1//TNF//LAMTOR3//MAP3K2//CRK//CRKL//ADAM9//LPAR1//LPAR3//IGF1//NTF3//PIK3CB//PRKAA1//MAP2K4//DUSP10//DUSP1//DUSP2//PPP2CA//RGS3//TOPORS//WWP2//HECTD2//RNF145//ZNRF2//FBXL5//RNF180//MYLIP//RLIM//ANKIB1//UBE2W//FBXW7//HECW2//SKP1//THOP1//LONRF3//FBXO30//CBFB//UBE3B//LONRF1//COPS8//DRD1//FNIP1//FAM129A//LRP6//PPP1R15B//SLIT2//EFNA1//EFNA5//EPHA7//FMR1//NPTN//KDR//P2RY1//AKTIP//SQSTM1//CCND2//LYPLA1//ERRFI1//TSG101//SOCS5//TRIM3//KLHL2//RNF38//MYCBP2//FBXO33//KLHL3//SPOPL//MDM4//DCAF8//DCAF6//FEM1C//KLHL14//MARCH4//RNF4//MARCH7//UBE2Z//RNF128//KLHL15//KBTBD8//ARRDC4//ZFAND4//HIPK3//ADAM10//CDK6//CDK8//FASTK//PDIK1L//ERG//CDK19//MORC3//MMD//PAN3//DSTYK//LATS2//MKNK2//CDK17//PRKACB//FAM20C//CCL8//TGFB2//TGFBR2//RUNX3//CCNC//RPS6KA4//ACVR1//RPS6KA5//STK17B//PPM1E//FLRT1//GNAQ//NCK1//NF2//ASPN//DUS2//YWHAG//CAMK2N2//PPM1F//PTPN21//PPP4R2//PPP1R12A//CTDSPL2//PPP3R1//PPP6C//PTPN4//KAT2B//CLOCK//NAA30//HS3ST5//PCMTD1//BTG1//BTG2//ASPH//FUT9//SERP1//ST8SIA5//MAN1A1//MAN2A1//ST8SIA3//B3GNT5//B3GALT2//CCDC126//GFPT2//VEGFB//NTNG1//RECK//PTAR1//PIGA//PTGER4//MDFIC//CALM2//GDF11//INHBB//BMP3//BMPR2//ST3GAL3//EPC2//ARID4B//MIER1//ARID4A//RBBP7//TBL1XR1//BRMS1L//ARID5B//USP3//DMWD//ESR1//OTUD3//USP24//AR//USP27X//ATXN3//USP28//USP46//USP45//WDR20//JOSD1//NUP35//EGR2//NUP43//PIAS4//UBE2I//VKORC1L1//CEP41//STK33//MASTL//OSR1//EPHA2//EPHA4//EPHA5//PKDCC//EGLN3//TMX3//ZDHHC1//ST6GALNAC3//ST6GALNAC6//ST6GAL2//ST3GAL5//MCFD2//CRY2//ZDHHC5//BAG6//CRTAP//EGLN1//PAK6//SLK//JARID2//SOX4//PER2//BAG5//NDFIP2//WFS1//PKD2//JUN//RAP2C//PPP1R37//URI1//OSM//PFN2//CAPRIN2//IFNA1//HMG20A//ADCY3//PRKAR2B//ESCO2//PRDM16//DLC1//CNEP1R1//NSMF//PTPDC1//PTPRG//PTP4A1//CDC14A//RNF2//HMGA2//PCGF5//EDEM1//CNOT7//STAT3//EDN1//ITGAV//FZD10//AIDA//TWF1//UBXN2B//TSC1//CKAP4//RAB38//MIA3//ANO8//TADA1//DR1//IPO5//CCNY//MAPRE3//VLDLR//ZMYND11//WNT7A//ULK2//ITGA5//LRP8//ARRDC3//DPY30//KMT2B//DNMT3B//MYB//RNF141//PMEPA1//LDLRAD4//NOG//ATG16L1//LMO3//KLF4//NPNT//DNAJC27//HAND2//KDM2A//EZH1//REST//CDC23//UBA5//ACSL1//GLRX//PLAA//RAB3GAP1//DERL2//FRY//UEVLD//RIMKLB// |
| GO:0036211 | protein modification process | Biological process | 308 | 4192 | 934 | 17653 | 1.38867650424179 | 3.0495377456473e-11 | 4.91048185090778e-09 | 10.5157659867939 | 0.329764453961456 | CDKN1A//GADD45A//PTEN//CCNE2//RANBP9//PPM1L//SPRED1//DUSP5//EREG//RASGEF1A//GRB2//NRG1//IL2//JAK1//MAP3K5//MET//MINK1//TAOK3//MAPK1//CCL2//SOS1//TNF//LAMTOR3//MAP3K2//CRK//CRKL//ADAM9//LPAR1//LPAR3//IGF1//NTF3//PIK3CB//PRKAA1//MAP2K4//DUSP10//DUSP1//DUSP2//PPP2CA//RGS3//TOPORS//WWP2//HECTD2//RNF145//ZNRF2//FBXL5//RNF180//MYLIP//RLIM//ANKIB1//UBE2W//FBXW7//HECW2//SKP1//THOP1//LONRF3//FBXO30//CBFB//UBE3B//LONRF1//COPS8//DRD1//FNIP1//FAM129A//LRP6//PPP1R15B//SLIT2//EFNA1//EFNA5//EPHA7//FMR1//NPTN//KDR//P2RY1//AKTIP//SQSTM1//CCND2//LYPLA1//ERRFI1//TSG101//SOCS5//TRIM3//KLHL2//RNF38//MYCBP2//FBXO33//KLHL3//SPOPL//MDM4//DCAF8//DCAF6//FEM1C//KLHL14//MARCH4//RNF4//MARCH7//UBE2Z//RNF128//KLHL15//KBTBD8//ARRDC4//ZFAND4//PPP4R2//SERP1//UEVLD//RIMKLB//UBE2I//HIPK3//ADAM10//CDK6//CDK8//FASTK//PDIK1L//ERG//CDK19//MORC3//MMD//PAN3//DSTYK//LATS2//MKNK2//CDK17//PRKACB//FAM20C//CCL8//TGFB2//TGFBR2//RUNX3//CCNC//RPS6KA4//ACVR1//RPS6KA5//STK17B//PPM1E//FLRT1//GNAQ//NCK1//NF2//ASPN//DUS2//YWHAG//CAMK2N2//PPM1F//PTPN21//PPP1R12A//CTDSPL2//PPP3R1//PPP6C//PTPN4//KAT2B//CLOCK//NAA30//HS3ST5//PCMTD1//BTG1//BTG2//ASPH//FUT9//ST8SIA5//MAN1A1//MAN2A1//ST8SIA3//B3GNT5//B3GALT2//CCDC126//GFPT2//VEGFB//NTNG1//RECK//PTAR1//PIGA//PTGER4//MDFIC//CALM2//GDF11//INHBB//BMP3//BMPR2//ST3GAL3//EPC2//ARID4B//MIER1//ARID4A//RBBP7//TBL1XR1//BRMS1L//ARID5B//USP3//DMWD//ESR1//OTUD3//USP24//AR//USP27X//ATXN3//USP28//USP46//USP45//WDR20//JOSD1//NUP35//EGR2//NUP43//PIAS4//VKORC1L1//CEP41//STK33//MASTL//OSR1//EPHA2//EPHA4//EPHA5//PKDCC//EGLN3//TMX3//ZDHHC1//ST6GALNAC3//ST6GALNAC6//ST6GAL2//ST3GAL5//MCFD2//CRY2//ZDHHC5//BAG6//CRTAP//EGLN1//PAK6//SLK//JARID2//SOX4//PER2//BAG5//NDFIP2//WFS1//PKD2//JUN//RAP2C//PPP1R37//URI1//OSM//PFN2//CAPRIN2//IFNA1//HMG20A//ADCY3//PRKAR2B//ESCO2//PRDM16//DLC1//CNEP1R1//NSMF//PTPDC1//PTPRG//PTP4A1//CDC14A//RNF2//HMGA2//PCGF5//EDEM1//CNOT7//STAT3//EDN1//ITGAV//FZD10//AIDA//TWF1//UBXN2B//TSC1//CKAP4//RAB38//MIA3//ANO8//TADA1//DR1//IPO5//CCNY//MAPRE3//VLDLR//ZMYND11//WNT7A//ULK2//ITGA5//LRP8//ARRDC3//DPY30//KMT2B//DNMT3B//MYB//RNF141//PMEPA1//LDLRAD4//NOG//ATG16L1//LMO3//KLF4//NPNT//DNAJC27//HAND2//KDM2A//EZH1//REST//CDC23//UBA5//ACSL1//GLRX//PLAA//RAB3GAP1//DERL2//FRY// |
| GO:0048699 | generation of neurons | Biological process | 134 | 1437 | 934 | 17653 | 1.76246164758546 | 4.19985185581597e-11 | 6.605487930438e-09 | 10.3767660284917 | 0.143468950749465 | STAT3//DCX//GJA1//GPM6A//LRP12//NR4A2//SPOCK1//UNK//SOX4//SOX11//HAND2//NTNG1//SLITRK3//NEFH//OGN//POU4F1//DSCAML1//WNT7A//SLIT2//RANBP9//ARX//DPYSL2//EFNA1//EFNA5//EPHA5//GAB1//GRB2//KIF5A//OTX2//PIK3CB//ENAH//MAPK1//NTN4//SOS1//VLDLR//NR4A3//KLF7//RPS6KA5//FEZ2//EGR2//EPHA4//LHX1//NOG//PTEN//SNAP25//ZNF804A//STMN2//NPTN//NCK1//NME1//SERPINI1//TWF1//LPAR1//MYLIP//PTPRG//TSC1//BAG5//NF2//GNAQ//FLRT1//STRN//MNX1//MTPN//BCL11B//DRD1//LHX6//LHX8//HPRT1//C21ORF91//BTG2//NFIB//CEBPB//FZD10//S1PR1//EMX2//MYT1L//HOXD1//NEUROG1//POU3F2//RUNX3//RAB21//JUN//FRY//PSD//EPHA7//TOPORS//NEUROD1//DLL1//NTF3//YWHAG//HMG20A//DDX6//REST//DNMT3B//MMD//RARB//ZEB1//GOLGA4//NRG1//EN1//FMR1//PBX3//TGFB2//FXR1//ULK2//LPAR3//KLF4//RGMA//TNFRSF21//PPARG//RHEB//DUSP10//MINK1//NSMF//HECW2//BMPR2//SEMA4B//PER2//MAN2A1//CAPRIN2//MYB//SLC12A5//CTNND2//WASL//CPEB3//IL2//LRP8//NEFM//LDLR//LRP6//NANOS1//PLAA//CDK6//TCF7L1// |
| GO:1903507 | negative regulation of nucleic acid-templated transcription | Biological process | 119 | 1224 | 934 | 17653 | 1.83754163692601 | 4.51662697250067e-11 | 6.94226095795955e-09 | 10.3451857762693 | 0.127408993576017 | TSHZ1//HMG20A//KLF2//ZBTB18//CEBPB//ZNF268//TCERG1//WWP2//KLF12//OSR1//CRY2//GLIS3//ARX//DNMT3B//DR1//E2F1//EDN1//EFNA1//CC2D1B//EN1//ESR1//FHL2//CPEB3//FOXF1//FOXC1//MYT1L//CIC//ZFPM2//DNAJB5//GATA6//CNOT7//HSBP1//AR//JARID2//JUN//ZNF568//MAF//MDM4//MYB//NFIA//NFIB//NR4A2//RLIM//KLF3//ZBTB7A//PIAS4//POU4F1//PPARG//CC2D1A//TSHZ3//ZBTB4//RARB//RBBP7//RBBP8//REST//RNF2//PRDM16//SOX17//SOX11//STAT3//ZEB1//TCF21//TFAP2A//TFAP2C//TGIF1//KLF10//TNF//UBE2I//VLDLR//WFS1//ZNF217//LRP8//BTG2//BHLHE41//TBL1XR1//NR4A3//HMGA2//ARID5B//BRMS1L//BHLHE40//RUNX3//URI1//PER2//SQSTM1//NOG//KLF4//HAND1//FNIP1//PHF14//USP3//BAHD1//H2AFZ//DPY30//ELK3//EREG//FOXF2//DKK3//MDFIC//NRG1//IRF1//LHX1//PAX9//RASD1//ARID4A//ATXN1//THRA//TSG101//BTAF1//RPS6KA5//CLOCK//PPM1F//ATAD2//MIER1//NCK1//TOB1//TOB2//ZMYND11//NPAT//SRSF2// |
| GO:1902679 | negative regulation of RNA biosynthetic process | Biological process | 119 | 1226 | 934 | 17653 | 1.83454401598485 | 5.01298401934171e-11 | 7.53395798284622e-09 | 10.2999036798519 | 0.127408993576017 | TSHZ1//HMG20A//KLF2//ZBTB18//CEBPB//ZNF268//TCERG1//WWP2//KLF12//OSR1//CRY2//GLIS3//ARX//DNMT3B//DR1//E2F1//EDN1//EFNA1//CC2D1B//EN1//ESR1//FHL2//CPEB3//FOXF1//FOXC1//MYT1L//CIC//ZFPM2//DNAJB5//GATA6//CNOT7//HSBP1//AR//JARID2//JUN//ZNF568//MAF//MDM4//MYB//NFIA//NFIB//NR4A2//RLIM//KLF3//ZBTB7A//PIAS4//POU4F1//PPARG//CC2D1A//TSHZ3//ZBTB4//RARB//RBBP7//RBBP8//REST//RNF2//PRDM16//SOX17//SOX11//STAT3//ZEB1//TCF21//TFAP2A//TFAP2C//TGIF1//KLF10//TNF//UBE2I//VLDLR//WFS1//ZNF217//LRP8//BTG2//BHLHE41//TBL1XR1//NR4A3//HMGA2//ARID5B//BRMS1L//BHLHE40//RUNX3//URI1//PER2//SQSTM1//NOG//KLF4//HAND1//FNIP1//PHF14//USP3//BAHD1//H2AFZ//DPY30//ELK3//EREG//FOXF2//DKK3//MDFIC//NRG1//IRF1//LHX1//PAX9//RASD1//ARID4A//ATXN1//THRA//TSG101//BTAF1//RPS6KA5//CLOCK//PPM1F//ATAD2//MIER1//TOB1//TOB2//ZMYND11//NPAT//SRSF2//NCK1// |
| GO:0009889 | regulation of biosynthetic process | Biological process | 337 | 4707 | 934 | 17653 | 1.3531855375997 | 5.25173487772323e-11 | 7.7211919517483e-09 | 10.2796972064303 | 0.360813704496788 | E2F1//NPAT//RRM2//TSHZ1//HMG20A//KLF2//ZBTB18//CEBPB//ZNF268//TCERG1//WWP2//KLF12//OSR1//CRY2//GLIS3//ARX//DNMT3B//DR1//EDN1//EFNA1//CC2D1B//EN1//ESR1//FHL2//CPEB3//FOXF1//FOXC1//MYT1L//CIC//ZFPM2//DNAJB5//GATA6//CNOT7//HSBP1//AR//JARID2//JUN//ZNF568//MAF//MDM4//MYB//NFIA//NFIB//NR4A2//RLIM//KLF3//ZBTB7A//PIAS4//POU4F1//PPARG//CC2D1A//TSHZ3//ZBTB4//RARB//RBBP7//RBBP8//REST//RNF2//PRDM16//SOX17//SOX11//STAT3//ZEB1//TCF21//TFAP2A//TFAP2C//TGIF1//KLF10//TNF//UBE2I//VLDLR//WFS1//ZNF217//LRP8//BTG2//BHLHE41//TBL1XR1//NR4A3//HMGA2//ARID5B//BRMS1L//BHLHE40//RUNX3//URI1//PER2//SQSTM1//NOG//KLF4//HAND1//FNIP1//PHF14//USP3//SECISBP2L//PKD2//THRA//NUP35//NUP43//NLN//ESCO2//PPP2CA//BAHD1//H2AFZ//KMT2B//DPY30//HIPK3//KHDRBS3//ZMYND11//ZNF597//CTNND2//ZNF782//ZNF800//ELAVL2//ZBTB9//KDM2A//MYCBP2//FLII//CNOT6L//NR5A2//GTF2E1//SERTAD3//NRBF2//HIVEP2//HMGB3//ZBTB41//ZBTB34//ATXN3//MLLT6//RNF141//BNC2//ZNF532//CDCA7L//LMO3//SLC2A4RG//PAK6//PHTF2//ZNF630//CNOT6//ZNF512B//ZBTB26//BCL11B//SNAPC1//SOX4//BTF3//BTG1//TMF1//ZNF2//ZNF3//ZNF131//ZNF655//SNIP1//TCF7L1//WASL//RPS6KA4//RPS6KA5//ZBTB47//CLOCK//CDKN1A//IRF9//TADA1//CRK//ZNF367//ELK3//EMX2//ERG//ETS1//FOXJ3//FOXF2//FLI1//EPC2//LHX6//HBP1//ATAD2//GRHL1//MNX1//HOXA3//HOXD1//FOXN2//BOLA3//NR3C2//LHX8//MYBL1//NEUROG1//NFE2//NHLH2//ARID4B//PLEK//BRWD1//POU3F2//TRERF1//ZNFX1//MIER1//RAD21//ARID4A//RBL2//DMRTA1//NKX2-4//RFX7//SMARCD2//TEF//DEK//BRD3//FOSL1//LCOR//KLF7//CBFB//POLR3G//BDP1//MTPN//DDX6//MKNK2//ACO1//TSC1//KBTBD8//KAT2B//G3BP2//NF2//IGF1//CNEP1R1//LDLR//FBXW7//FAM129A//FMR1//SERP1//PRR16//MAPK1//FXR1//TOPORS//MAP3K2//KLF6//EGR2//MAPRE3//BAMBI//RGMB//MDFIC//IRF1//LHX1//LRP6//MAP3K5//NEUROD1//NPAS2//OTX2//ZNF287//RNF4//TEAD1//TGFB1I1//WNT7A//FUBP3//ACVR1//HAND2//EREG//DKK3//NRG1//PAX9//RASD1//ATXN1//TSG101//BTAF1//PPM1F//TOB1//NANOS1//CAPRIN2//SOSTDC1//TMEM100//RGMA//BMP3//BMPR2//TGFB2//SULF1//CTDSPL2//ADRB1//PTHLH//CALM2//EGLN1//CMKLR1//DCP2//CCT6A//EPHA5//P2RY1//ERRFI1//NCK1//RNF128//ADIPOR2//ITM2B//FZD6//PRKAA1//ITGAV//TMEFF2//MID1IP1//MAP2K4//CDK8//CREBRF//NKX2-3//E2F3//E2F5//S1PR1//EZH1//DLL1//IL2//ITGA6//MEF2D//MEOX2//MET//PPP1R12A//OSM//PBX3//IER5//PPP3R1//DCAF6//SHOX//STAT4//PCGF5//CCNC//ST18//PAIP2//INHBB//PTEN//KDR//CCL2//PTGES3//YTHDF2//QKI//EGLN3//IDH1//ORMDL3//CPEB2//RAB3GAP1//RAP2C//TOB2//SRSF2//PPP1R15B//DUSP1//GJA1//NSMAF//NPNT//CPEB1//CPEB4//UNK// |
| GO:0048731 | system development | Biological process | 340 | 4760 | 934 | 17653 | 1.35003059039462 | 5.42982143375309e-11 | 7.81316645882386e-09 | 10.2652144524758 | 0.364025695931477 | GDF11//CMKLR1//COL1A2//HAPLN1//EN1//FRZB//SERP1//GNAQ//IGF1//PTHLH//SHOX//BMP3//SOX4//SOX11//TGFB2//KLF10//PKDCC//NOG//THRA//WNT7A//S1PR1//EFNA1//EPHA2//ELK3//EREG//FOXC1//GAB1//CLIC4//HOXA3//ITGA5//ITGAV//JUN//KDR//MEOX2//MMP2//TMEM100//PTEN//CCL2//SOX17//VEGFB//HAND1//HAND2//CEBPB//INHBB//DMRTA1//IMMP2L//KMT2B//ESR1//PLEKHA1//CRKL//FOXF1//SPHK2//TGFBR2//EDN1//PPP3R1//ACVR1//GJC1//ZFPM2//QKI//SMOC1//OSR1//LHX1//RDH10//NPNT//RARB//TCF21//SLIT2//PKD2//STAT3//SEMA4B//DCX//GJA1//GPM6A//LRP12//NR4A2//SPOCK1//UNK//BCL2L11//SULF1//TFAP2A//TSC1//WFS1//BAG6//ARID5B//FOXF2//HMGA2//DLC1//LRP6//PRKACB//FZD6//GATA6//JARID2//MAN2A1//MET//UGT1A1//PPARG//BMPR2//APCDD1//SOS1//FBXW7//DLL1//RECK//MEF2D//NFIB//RUNX3//ESCO2//PLEK//RBM47//REST//NKX2-3//FNIP1//MARCH7//NME1//NRG1//ERG//POU4F1//CDK6//NEUROD1//PALLD//BCL11B//SLC17A7//E2F5//STX2//FLI1//BHLHE41//TRIM3//FUT9//CHRM2//SCRG1//RBFOX3//DPYSL2//EFNA5//MYT1L//FMR1//GLRB//MYLIP//MBNL1//ATXN3//NEUROG1//NTF3//TAOK3//POU3F2//VLDLR//ZIC5//ITM2B//FEZ2//NTNG1//SLITRK3//NEFH//OGN//DSCAML1//RANBP9//ARX//EPHA5//GRB2//KIF5A//OTX2//PIK3CB//ENAH//MAPK1//NTN4//NR4A3//KLF7//RPS6KA5//DRD1//NHLH2//NPAS2//SERPINI1//ZEB1//FXR1//B3GNT5//EGR2//EPHA7//RAB3GAP1//CIC//PTPRG//ATXN1//ADIPOR2//KAT2B//SGCE//ZBTB18//CFL2//EPHA4//P2RY1//CLDN11//RNF38//TFAP2C//IDH1//LHX8//BAMBI//TGFB1I1//PPP2CA//LDLRAD4//TOPORS//FJX1//SNAP25//ZNF804A//STMN2//NPTN//NCK1//TWF1//LPAR1//BAG5//WASF3//NF2//KBTBD8//MTPN//FLRT1//STRN//SPRED1//KLF4//PBX3//MNX1//LRP8//EMX2//BTG2//HPRT1//CALM2//SLC32A1//EZH1//LHX6//ETS1//C21ORF91//TACC2//BNIP3//PRDM16//TCF7L1//KCNJ10//MSN//MKNK2//CBFB//FZD10//HOXD1//KLF6//IFNA1//DNAJB9//LATS2//TSG101//ADAM9//IL2//ASPN//OSTM1//TNF//DKK3//FAM20C//RAB21//AR//E2F1//SOSTDC1//KRT27//CDKN1A//FRY//PSD//DSC2//TNFRSF21//PIKFYVE//MAF//LRIG1//TMF1//FOXN2//PTGER4//SOBP//TSHZ1//PAX9//SBF2//KLF2//IRF1//BNC2//ITGA6//ARRDC3//ERRFI1//HMGB3//DUSP10//GRHL1//MYB//SOCS5//NFE2//DPY30//YWHAG//HMG20A//DDX6//DNMT3B//MMD//TOB2//TMEM64//EGLN1//VASH2//F3//ITGB8//BTG1//ADAM12//GOLGA4//GJB2//PHF14//EPN1//TEAD1//PRKAA1//ULK2//LPAR3//RGMA//SLC35D1//RHEB//MINK1//NSMF//HECW2//TMOD3//ARID4A//CXCL14//PER2//CAPRIN2//ACTC1//FHL2//MDM1//VANGL1//NFIA//SYBU//MFAP5//TBL1XR1//ZNF568//FOSL1//BTBD7//SLC12A5//CTNND2//WASL//CPEB3//MAP2K4//NR5A2//NEFM//LDLR//TMOD1//TBC1D20//KLHL3//RAB23//ARID4B//NANOS1//YTHDF2//NLN//JAK1//PLAA//CLOCK// |
| GO:0019219 | regulation of nucleobase-containing compound metabolic process | Biological process | 314 | 4328 | 934 | 17653 | 1.37124179190729 | 8.08762529407011e-11 | 1.13951270549575e-08 | 10.0921789781499 | 0.336188436830835 | E2F1//NPAT//RRM2//TSHZ1//HMG20A//KLF2//ZBTB18//CEBPB//ZNF268//TCERG1//WWP2//KLF12//OSR1//CRY2//GLIS3//ARX//DNMT3B//DR1//EDN1//EFNA1//CC2D1B//EN1//ESR1//FHL2//CPEB3//FOXF1//FOXC1//MYT1L//CIC//ZFPM2//DNAJB5//GATA6//CNOT7//HSBP1//AR//JARID2//JUN//ZNF568//MAF//MDM4//MYB//NFIA//NFIB//NR4A2//RLIM//KLF3//ZBTB7A//PIAS4//POU4F1//PPARG//CC2D1A//TSHZ3//ZBTB4//RARB//RBBP7//RBBP8//REST//RNF2//PRDM16//SOX17//SOX11//STAT3//ZEB1//TCF21//TFAP2A//TFAP2C//TGIF1//KLF10//TNF//UBE2I//VLDLR//WFS1//ZNF217//LRP8//BTG2//BHLHE41//TBL1XR1//NR4A3//HMGA2//ARID5B//BRMS1L//BHLHE40//RUNX3//URI1//PER2//SQSTM1//NOG//KLF4//HAND1//FNIP1//PHF14//USP3//RBFOX3//FMR1//MBNL1//RBM25//SRSF2//PKD2//THRA//NUP35//NUP43//ESCO2//PPP2CA//BAHD1//H2AFZ//KMT2B//DPY30//HIPK3//KHDRBS3//ZMYND11//ZNF597//CTNND2//ZNF782//ZNF800//ELAVL2//ZBTB9//KDM2A//MYCBP2//FLII//CNOT6L//NR5A2//GTF2E1//SERTAD3//NRBF2//HIVEP2//HMGB3//ZBTB41//ZBTB34//ATXN3//MLLT6//RNF141//BNC2//ZNF532//CDCA7L//LMO3//SLC2A4RG//PAK6//PHTF2//ZNF630//CNOT6//ZNF512B//ZBTB26//BCL11B//SNAPC1//SOX4//BTF3//BTG1//TMF1//ZNF2//ZNF3//ZNF131//ZNF655//SNIP1//TCF7L1//WASL//RPS6KA4//RPS6KA5//ZBTB47//CLOCK//CDKN1A//IRF9//TADA1//CRK//ZNF367//ELK3//EMX2//ERG//ETS1//FOXJ3//FOXF2//FLI1//EPC2//LHX6//HBP1//ATAD2//GRHL1//MNX1//HOXA3//HOXD1//FOXN2//BOLA3//NR3C2//LHX8//MYBL1//NEUROG1//NFE2//NHLH2//ARID4B//PLEK//BRWD1//POU3F2//TRERF1//ZNFX1//MIER1//RAD21//ARID4A//RBL2//DMRTA1//NKX2-4//RFX7//SMARCD2//TEF//DEK//BRD3//FOSL1//LCOR//KLF7//CBFB//POLR3G//BDP1//KAT2B//G3BP2//NF2//PPP4R2//TOPORS//MAP3K2//KLF6//EGR2//MAPRE3//BAMBI//RGMB//MDFIC//IGF1//IRF1//LHX1//LRP6//MAP3K5//NEUROD1//NPAS2//OTX2//MAPK1//ZNF287//RNF4//TEAD1//TGFB1I1//WNT7A//FUBP3//ACVR1//HAND2//EREG//DKK3//NRG1//PAX9//RASD1//ATXN1//TSG101//BTAF1//PPM1F//TOB1//SOSTDC1//TMEM100//RGMA//BMP3//BMPR2//TGFB2//SULF1//CTDSPL2//CALM2//ADRB1//PTHLH//EGLN1//CMKLR1//DCP2//CCT6A//EPHA5//FBXW7//FZD6//FASTK//ESRP1//AHNAK//YTHDF2//FXR1//MAP2K4//PRKAA1//CDK8//CREBRF//NKX2-3//E2F3//E2F5//S1PR1//EZH1//DLL1//IL2//ITGA6//MEF2D//MEOX2//MET//PPP1R12A//NCK1//OSM//P2RY1//PBX3//IER5//PPP3R1//DCAF6//SHOX//CAPRIN2//STAT4//PCGF5//CCNC//ST18//PTEN//MTPN//NOVA1//PTGES3//QKI//EGLN3//NANOS1//RAP2C//TOB2//KLHL15//DUSP1//GJA1//NPNT// |
| GO:0044260 | cellular macromolecule metabolic process | Biological process | 548 | 8555 | 934 | 17653 | 1.21068786551812 | 8.27489655510565e-11 | 1.14210460004448e-08 | 10.0822374266553 | 0.586723768736617 | IGF1//NFIA//NFIB//RBBP7//RBBP8//RRM2//CDKN1A//GADD45A//PTEN//CCNE2//E2F1//NPAT//TSHZ1//HMG20A//KLF2//ZBTB18//CEBPB//ZNF268//TCERG1//WWP2//KLF12//OSR1//CRY2//GLIS3//ARX//DNMT3B//DR1//EDN1//EFNA1//CC2D1B//EN1//ESR1//FHL2//CPEB3//FOXF1//FOXC1//MYT1L//CIC//ZFPM2//DNAJB5//GATA6//CNOT7//HSBP1//AR//JARID2//JUN//ZNF568//MAF//MDM4//MYB//NR4A2//RLIM//KLF3//ZBTB7A//PIAS4//POU4F1//PPARG//CC2D1A//TSHZ3//ZBTB4//RARB//REST//RNF2//PRDM16//SOX17//SOX11//STAT3//ZEB1//TCF21//TFAP2A//TFAP2C//TGIF1//KLF10//TNF//UBE2I//VLDLR//WFS1//ZNF217//LRP8//BTG2//BHLHE41//TBL1XR1//NR4A3//HMGA2//ARID5B//BRMS1L//BHLHE40//RUNX3//URI1//PER2//SQSTM1//NOG//KLF4//HAND1//FNIP1//PHF14//USP3//RANBP9//PPM1L//SPRED1//DUSP5//EREG//RASGEF1A//GRB2//NRG1//IL2//JAK1//MAP3K5//MET//MINK1//TAOK3//MAPK1//CCL2//SOS1//LAMTOR3//DCP2//PPP2CA//RPL15//MAP3K2//CRK//CRKL//ADAM9//LPAR1//LPAR3//NTF3//PIK3CB//PRKAA1//MAP2K4//DUSP10//DUSP1//DUSP2//RGS3//TOPORS//HECTD2//RNF145//ZNRF2//FBXL5//RNF180//MYLIP//ANKIB1//UBE2W//FBXW7//HECW2//SKP1//THOP1//LONRF3//FBXO30//CBFB//UBE3B//LONRF1//ZFAND4//CNOT6L//PAN3//CNOT6//COPS8//PTGES3//NABP1//GTF2E1//SECISBP2L//CAMLG//EPS15//NSF//DRD1//FAM129A//LRP6//PPP1R15B//SLIT2//EFNA5//EPHA7//FMR1//NPTN//KDR//P2RY1//AKTIP//CCND2//LYPLA1//IRF9//KLF6//CREBRF//EGR2//ELK3//ERG//FOXJ3//FOXF2//FLI1//GRHL1//HIVEP2//IRF1//LHX1//MEF2D//MEOX2//MYBL1//NEUROD1//NHLH2//OTX2//PAX9//PBX3//POU3F2//RAD21//ARID4A//SHOX//BCL11B//BMPR2//SOX4//BTF3//TEF//TGFB1I1//THRA//TMF1//DEK//FOSL1//KLF7//FUBP3//HAND2//CLOCK//POLR3G//SRSF2//PKD2//ERRFI1//TSG101//SOCS5//TRIM3//KLHL2//RNF38//MYCBP2//FBXO33//KLHL3//SPOPL//DCAF8//DCAF6//FEM1C//KLHL14//MARCH4//RNF4//MARCH7//UBE2Z//RNF128//KLHL15//KBTBD8//ARRDC4//UGP2//NME1//REV3L//GMNC//ESCO2//EPC2//USP28//USP45//ATXN3//KDM2A//MTRR//HMGB3//BAHD1//H2AFZ//KMT2B//DPY30//KHDRBS3//ZMYND11//TADA1//ZNF597//CTNND2//ZNF782//NKX2-3//ZNF800//E2F5//ZNF367//EZH1//ZBTB9//FLII//LHX6//HBP1//ATAD2//SERTAD3//MDFIC//MNX1//HOXA3//HOXD1//FOXN2//ZBTB41//ZBTB34//LHX8//NEUROG1//NFE2//NPAS2//IER5//ARID4B//BRWD1//BNC2//ZNF532//CDCA7L//TRERF1//LMO3//SLC2A4RG//PHTF2//ZNF630//ZNF287//ZNF512B//ZBTB26//MIER1//RBL2//ATXN1//DMRTA1//SMARCD2//STAT4//ZNF2//ZNF3//ZNF131//ZNF655//BRD3//TCF7L1//PCGF5//LCOR//WASL//ZBTB47//ST18//HIPK3//NUP35//ELAVL2//NR5A2//NRBF2//MLLT6//RNF141//PAK6//SNAPC1//BTG1//SNIP1//RPS6KA4//RPS6KA5//EMX2//ETS1//BOLA3//NR3C2//PLEK//ZNFX1//NKX2-4//RFX7//BDP1//CDK8//E2F3//TEAD1//KAT2B//CCNC//CPEB2//CPEB1//CPEB4//MTPN//DDX6//MKNK2//ACO1//TSC1//PPP4R2//SERP1//UEVLD//RIMKLB//IMMP2L//ADAM10//CDK6//FASTK//PDIK1L//CDK19//MORC3//MMD//DSTYK//LATS2//CDK17//PRKACB//FAM20C//CCL8//TGFB2//TGFBR2//ACVR1//STK17B//PPM1E//FLRT1//GNAQ//NCK1//NF2//ASPN//DUS2//YWHAG//CAMK2N2//PPM1F//PTPN21//PPP1R12A//CTDSPL2//PPP3R1//PPP6C//PTPN4//NAA30//HS3ST5//PCMTD1//ASPH//FUT9//ST8SIA5//MAN1A1//MAN2A1//ST8SIA3//B3GNT5//B3GALT2//CCDC126//GFPT2//EDEM1//VEGFB//NTNG1//RECK//PTAR1//PIGA//CTSA//USP24//USP27X//NPLOC4//USP46//BAG6//CDC23//DLC1//EGLN3//F3//PMAIP1//DLL1//G3BP2//PTGER4//ST6GALNAC3//ST6GALNAC6//CALM2//GDF11//INHBB//BMP3//SERPINI1//SPOCK1//PCOLCE2//XYLT2//HS2ST1//PRR16//FXR1//GNPTG//ST3GAL3//MAPRE3//BAMBI//RGMB//WNT7A//DKK3//RASD1//BTAF1//DMWD//OTUD3//WDR20//JOSD1//NUP43//TOB1//NANOS1//CAPRIN2//VKORC1L1//CEP41//STK33//MASTL//EPHA2//EPHA4//EPHA5//PKDCC//TMX3//ZDHHC1//ST6GAL2//ST3GAL5//MCFD2//ZDHHC5//CRTAP//EGLN1//SLK//SULF1//DSEL//SLC35D1//CHST7//UBXN4//DNAJB9//DERL2//JKAMP//SOSTDC1//TMEM100//RGMA//BAG5//NDFIP2//GRIA1//RAP2C//CMKLR1//CCT6A//PPP1R37//OSM//PFN2//IFNA1//ADCY3//PRKAR2B//CNEP1R1//NSMF//PTPDC1//PTPRG//PTP4A1//CDC14A//BNIP3//DNAJB12//DCTPP1//INTS8//ITM2B//TDRD5//ARL6IP1//UBXN2B//CD2AP//PLAA//VPS37A//BCL2L11//ITGAV//FZD6//YTHDF2//FZD10//AIDA//TWF1//CKAP4//RAB38//MIA3//ANO8//RAB12//MMP2//TTR//IPO5//CCNY//S1PR1//ITGA6//PAIP2//ULK2//ITGA5//SH3D19//ARRDC3//PMEPA1//LDLRAD4//QKI//ATG16L1//NPNT//DNAJC27//UBA5//ACSL1//GLRX//CMTR2//SNAP25//RAB3GAP1//MSN//FRY//LDLR//GJA1//UNK// |
| GO:0097659 | nucleic acid-templated transcription | Biological process | 290 | 3924 | 934 | 17653 | 1.39682064143785 | 8.70708667677627e-11 | 1.17772054390076e-08 | 10.0601271322307 | 0.310492505353319 | E2F1//NPAT//RRM2//TSHZ1//HMG20A//KLF2//ZBTB18//CEBPB//ZNF268//TCERG1//WWP2//KLF12//OSR1//CRY2//GLIS3//ARX//DNMT3B//DR1//EDN1//EFNA1//CC2D1B//EN1//ESR1//FHL2//CPEB3//FOXF1//FOXC1//MYT1L//CIC//ZFPM2//DNAJB5//GATA6//CNOT7//HSBP1//AR//JARID2//JUN//ZNF568//MAF//MDM4//MYB//NFIA//NFIB//NR4A2//RLIM//KLF3//ZBTB7A//PIAS4//POU4F1//PPARG//CC2D1A//TSHZ3//ZBTB4//RARB//RBBP7//RBBP8//REST//RNF2//PRDM16//SOX17//SOX11//STAT3//ZEB1//TCF21//TFAP2A//TFAP2C//TGIF1//KLF10//TNF//UBE2I//VLDLR//WFS1//ZNF217//LRP8//BTG2//BHLHE41//TBL1XR1//NR4A3//HMGA2//ARID5B//BRMS1L//BHLHE40//RUNX3//URI1//PER2//SQSTM1//NOG//KLF4//HAND1//FNIP1//PHF14//USP3//GTF2E1//IRF9//KLF6//CREBRF//EGR2//ELK3//ERG//FOXJ3//FOXF2//FLI1//GRHL1//HIVEP2//IRF1//LHX1//MEF2D//MEOX2//MYBL1//NEUROD1//NHLH2//OTX2//PAX9//PBX3//POU3F2//RAD21//ARID4A//SHOX//BCL11B//BMPR2//SOX4//BTF3//TEF//TGFB1I1//THRA//TMF1//DEK//FOSL1//KLF7//CBFB//FUBP3//HAND2//CLOCK//POLR3G//SRSF2//PKD2//BAHD1//H2AFZ//KMT2B//DPY30//TOPORS//KHDRBS3//ZMYND11//TADA1//ZNF597//CTNND2//ZNF782//NKX2-3//ZNF800//E2F5//ZNF367//EZH1//ZBTB9//KDM2A//MYCBP2//FLII//CNOT6L//EPC2//LHX6//HBP1//ATAD2//SERTAD3//MDFIC//MNX1//HMGB3//HOXA3//HOXD1//FOXN2//ZBTB41//ZBTB34//ATXN3//LHX8//NEUROG1//NFE2//NPAS2//IER5//ARID4B//BRWD1//BNC2//ZNF532//CDCA7L//PRKAA1//TRERF1//LMO3//MAPK1//SLC2A4RG//PHTF2//ZNF630//ZNF287//CNOT6//ZNF512B//ZBTB26//MIER1//RBL2//RNF4//ATXN1//DMRTA1//SMARCD2//STAT4//ZNF2//ZNF3//ZNF131//ZNF655//BRD3//TCF7L1//PCGF5//LCOR//WASL//ZBTB47//ST18//HIPK3//NUP35//ELAVL2//NR5A2//NRBF2//MLLT6//RNF141//PPP2CA//PAK6//SNAPC1//BTG1//SNIP1//RPS6KA4//RPS6KA5//CDKN1A//CRK//EMX2//ETS1//BOLA3//NR3C2//PLEK//ZNFX1//NKX2-4//RFX7//BDP1//CDK8//E2F3//TEAD1//KAT2B//CCNC//G3BP2//EREG//MAP3K2//MAPRE3//BAMBI//RGMB//IGF1//LRP6//MAP3K5//WNT7A//ACVR1//DKK3//NRG1//RASD1//TSG101//BTAF1//PPM1F//TOB1//SOSTDC1//TMEM100//RGMA//BMP3//TGFB2//SULF1//CTDSPL2//CMKLR1//EPHA5//INTS8//NABP1//EGLN1//FZD6//S1PR1//DLL1//IL2//ITGA6//MET//PPP1R12A//NCK1//OSM//P2RY1//PPP3R1//DCAF6//CAPRIN2//PTEN//MTPN//EGLN3//RAP2C//TOB2//FBXW7//NPNT// |
| GO:0031326 | regulation of cellular biosynthetic process | Biological process | 332 | 4640 | 934 | 17653 | 1.35235822934357 | 8.89710012527167e-11 | 1.17982525778848e-08 | 10.0507515219856 | 0.355460385438972 | E2F1//NPAT//RRM2//TSHZ1//HMG20A//KLF2//ZBTB18//CEBPB//ZNF268//TCERG1//WWP2//KLF12//OSR1//CRY2//GLIS3//ARX//DNMT3B//DR1//EDN1//EFNA1//CC2D1B//EN1//ESR1//FHL2//CPEB3//FOXF1//FOXC1//MYT1L//CIC//ZFPM2//DNAJB5//GATA6//CNOT7//HSBP1//AR//JARID2//JUN//ZNF568//MAF//MDM4//MYB//NFIA//NFIB//NR4A2//RLIM//KLF3//ZBTB7A//PIAS4//POU4F1//PPARG//CC2D1A//TSHZ3//ZBTB4//RARB//RBBP7//RBBP8//REST//RNF2//PRDM16//SOX17//SOX11//STAT3//ZEB1//TCF21//TFAP2A//TFAP2C//TGIF1//KLF10//TNF//UBE2I//VLDLR//WFS1//ZNF217//LRP8//BTG2//BHLHE41//TBL1XR1//NR4A3//HMGA2//ARID5B//BRMS1L//BHLHE40//RUNX3//URI1//PER2//SQSTM1//NOG//KLF4//HAND1//FNIP1//PHF14//USP3//SECISBP2L//PKD2//THRA//NUP35//NUP43//ESCO2//PPP2CA//BAHD1//H2AFZ//KMT2B//DPY30//HIPK3//KHDRBS3//ZMYND11//ZNF597//CTNND2//ZNF782//ZNF800//ELAVL2//ZBTB9//KDM2A//MYCBP2//FLII//CNOT6L//NR5A2//GTF2E1//SERTAD3//NRBF2//HIVEP2//HMGB3//ZBTB41//ZBTB34//ATXN3//MLLT6//RNF141//BNC2//ZNF532//CDCA7L//LMO3//SLC2A4RG//PAK6//PHTF2//ZNF630//CNOT6//ZNF512B//ZBTB26//BCL11B//SNAPC1//SOX4//BTF3//BTG1//TMF1//ZNF2//ZNF3//ZNF131//ZNF655//SNIP1//TCF7L1//WASL//RPS6KA4//RPS6KA5//ZBTB47//CLOCK//CDKN1A//IRF9//TADA1//CRK//ZNF367//ELK3//EMX2//ERG//ETS1//FOXJ3//FOXF2//FLI1//EPC2//LHX6//HBP1//ATAD2//GRHL1//MNX1//HOXA3//HOXD1//FOXN2//BOLA3//NR3C2//LHX8//MYBL1//NEUROG1//NFE2//NHLH2//ARID4B//PLEK//BRWD1//POU3F2//TRERF1//ZNFX1//MIER1//RAD21//ARID4A//RBL2//DMRTA1//NKX2-4//RFX7//SMARCD2//TEF//DEK//BRD3//FOSL1//LCOR//KLF7//CBFB//POLR3G//BDP1//MTPN//DDX6//MKNK2//ACO1//TSC1//KBTBD8//KAT2B//G3BP2//NF2//IGF1//CNEP1R1//LDLR//FBXW7//FAM129A//FMR1//SERP1//PRR16//MAPK1//FXR1//TOPORS//MAP3K2//KLF6//EGR2//MAPRE3//BAMBI//RGMB//MDFIC//IRF1//LHX1//LRP6//MAP3K5//NEUROD1//NPAS2//OTX2//ZNF287//RNF4//TEAD1//TGFB1I1//WNT7A//FUBP3//ACVR1//HAND2//EREG//DKK3//NRG1//PAX9//RASD1//ATXN1//TSG101//BTAF1//PPM1F//TOB1//NANOS1//CAPRIN2//SOSTDC1//TMEM100//RGMA//BMP3//BMPR2//TGFB2//SULF1//CTDSPL2//ADRB1//PTHLH//CALM2//EGLN1//CMKLR1//DCP2//CCT6A//EPHA5//P2RY1//NCK1//RNF128//ADIPOR2//ERRFI1//ITM2B//FZD6//MID1IP1//MAP2K4//PRKAA1//CDK8//CREBRF//NKX2-3//E2F3//E2F5//S1PR1//EZH1//DLL1//IL2//ITGA6//MEF2D//MEOX2//MET//PPP1R12A//OSM//PBX3//IER5//PPP3R1//DCAF6//SHOX//STAT4//PCGF5//CCNC//ST18//PAIP2//INHBB//PTEN//PTGES3//YTHDF2//QKI//EGLN3//IDH1//ORMDL3//CPEB2//RAB3GAP1//RAP2C//TOB2//SRSF2//PPP1R15B//DUSP1//GJA1//NSMAF//NPNT//CPEB1//CPEB4//UNK// |
| GO:0031399 | regulation of protein modification process | Biological process | 157 | 1800 | 934 | 17653 | 1.64853735427076 | 1.17488744142538e-10 | 1.52068144240494e-08 | 9.93000373842151 | 0.168094218415418 | CDKN1A//GADD45A//PTEN//CCNE2//MINK1//TNF//MAP3K2//CRK//CRKL//MAP3K5//TAOK3//LAMTOR3//ADAM9//DUSP5//LPAR1//LPAR3//NRG1//IGF1//NTF3//PIK3CB//PRKAA1//MAPK1//MAP2K4//DUSP10//SPRED1//DUSP1//DUSP2//PPP2CA//RGS3//DRD1//FNIP1//FAM129A//IL2//LRP6//PPP1R15B//SLIT2//EFNA1//EFNA5//EPHA7//FMR1//NPTN//KDR//P2RY1//AKTIP//SQSTM1//CCND2//EREG//FBXW7//ERRFI1//TSG101//SOCS5//PPM1E//FLRT1//GNAQ//NCK1//NF2//ASPN//DUS2//YWHAG//CAMK2N2//PPM1F//COPS8//MDFIC//CALM2//KAT2B//GDF11//INHBB//BMP3//BMPR2//TGFB2//ACVR1//JARID2//SPOPL//SOX4//PER2//BAG5//NDFIP2//WFS1//CDK6//PKD2//JUN//RAP2C//PAK6//TGFBR2//SLK//CRY2//PPP1R37//MASTL//URI1//RPS6KA4//RPS6KA5//OSM//PFN2//CAPRIN2//IFNA1//HMG20A//RNF4//PIAS4//ADCY3//PRKACB//PRKAR2B//DLC1//CNEP1R1//NSMF//PPP1R12A//CNOT7//STAT3//EDN1//GRB2//EPHA2//EPHA4//EPHA5//AR//ITGAV//MET//FZD10//HIPK3//AIDA//TWF1//UBXN2B//PPP4R2//TSC1//LATS2//IPO5//CCNY//MAPRE3//CCNC//MMD//VLDLR//ZMYND11//WNT7A//ITGA5//LRP8//TOPORS//ARRDC3//ARRDC4//KMT2B//DNMT3B//MYB//PMEPA1//LDLRAD4//NOG//RANBP9//LMO3//KLF4//NPNT//DSTYK//DNAJC27//CCL2//CCL8//HAND2//ACSL1//PLAA//CRTAP//RAB3GAP1//UBE2I//FRY// |
| GO:2000112 | regulation of cellular macromolecule biosynthetic process | Biological process | 316 | 4376 | 934 | 17653 | 1.36483896981394 | 1.1917213728739e-10 | 1.52068144240494e-08 | 9.92382527175017 | 0.338329764453961 | E2F1//NPAT//RRM2//TSHZ1//HMG20A//KLF2//ZBTB18//CEBPB//ZNF268//TCERG1//WWP2//KLF12//OSR1//CRY2//GLIS3//ARX//DNMT3B//DR1//EDN1//EFNA1//CC2D1B//EN1//ESR1//FHL2//CPEB3//FOXF1//FOXC1//MYT1L//CIC//ZFPM2//DNAJB5//GATA6//CNOT7//HSBP1//AR//JARID2//JUN//ZNF568//MAF//MDM4//MYB//NFIA//NFIB//NR4A2//RLIM//KLF3//ZBTB7A//PIAS4//POU4F1//PPARG//CC2D1A//TSHZ3//ZBTB4//RARB//RBBP7//RBBP8//REST//RNF2//PRDM16//SOX17//SOX11//STAT3//ZEB1//TCF21//TFAP2A//TFAP2C//TGIF1//KLF10//TNF//UBE2I//VLDLR//WFS1//ZNF217//LRP8//BTG2//BHLHE41//TBL1XR1//NR4A3//HMGA2//ARID5B//BRMS1L//BHLHE40//RUNX3//URI1//PER2//SQSTM1//NOG//KLF4//HAND1//FNIP1//PHF14//USP3//SECISBP2L//PKD2//THRA//ESCO2//PPP2CA//BAHD1//H2AFZ//KMT2B//DPY30//HIPK3//KHDRBS3//ZMYND11//NUP35//ZNF597//CTNND2//ZNF782//ZNF800//ELAVL2//ZBTB9//KDM2A//MYCBP2//FLII//CNOT6L//NR5A2//GTF2E1//SERTAD3//NRBF2//HIVEP2//HMGB3//ZBTB41//ZBTB34//ATXN3//MLLT6//RNF141//BNC2//ZNF532//CDCA7L//LMO3//SLC2A4RG//PAK6//PHTF2//ZNF630//CNOT6//ZNF512B//ZBTB26//BCL11B//SNAPC1//SOX4//BTF3//BTG1//TMF1//ZNF2//ZNF3//ZNF131//ZNF655//SNIP1//TCF7L1//WASL//RPS6KA4//RPS6KA5//ZBTB47//CLOCK//CDKN1A//IRF9//TADA1//CRK//ZNF367//ELK3//EMX2//ERG//ETS1//FOXJ3//FOXF2//FLI1//EPC2//LHX6//HBP1//ATAD2//GRHL1//MNX1//HOXA3//HOXD1//FOXN2//BOLA3//NR3C2//LHX8//MYBL1//NEUROG1//NFE2//NHLH2//ARID4B//PLEK//BRWD1//POU3F2//TRERF1//ZNFX1//MIER1//RAD21//ARID4A//RBL2//DMRTA1//NKX2-4//RFX7//SMARCD2//TEF//DEK//BRD3//FOSL1//LCOR//KLF7//CBFB//POLR3G//BDP1//MTPN//DDX6//MKNK2//ACO1//TSC1//KBTBD8//KAT2B//G3BP2//NF2//IGF1//FAM129A//FMR1//SERP1//PRR16//MAPK1//FXR1//TOPORS//MAP3K2//KLF6//EGR2//MAPRE3//BAMBI//RGMB//MDFIC//IRF1//LHX1//LRP6//MAP3K5//NEUROD1//NPAS2//OTX2//ZNF287//RNF4//TEAD1//TGFB1I1//WNT7A//FUBP3//ACVR1//HAND2//EREG//DKK3//NRG1//PAX9//RASD1//ATXN1//TSG101//BTAF1//PPM1F//TOB1//NANOS1//CAPRIN2//SOSTDC1//TMEM100//RGMA//BMP3//BMPR2//TGFB2//SULF1//CTDSPL2//CMKLR1//DCP2//CCT6A//EPHA5//FBXW7//NCK1//ITM2B//EGLN1//FZD6//MAP2K4//CDK8//CREBRF//NKX2-3//E2F3//E2F5//S1PR1//EZH1//DLL1//IL2//ITGA6//MEF2D//MEOX2//MET//PPP1R12A//OSM//P2RY1//PBX3//IER5//PPP3R1//DCAF6//SHOX//STAT4//PCGF5//CCNC//ST18//PAIP2//PTEN//PTGES3//NUP43//YTHDF2//QKI//EGLN3//CPEB2//RAB3GAP1//PRKAA1//PPP1R15B//DUSP1//GJA1//NPNT//CPEB1//CPEB4//UNK// |
| GO:0071310 | cellular response to organic substance | Biological process | 212 | 2656 | 934 | 17653 | 1.50861852119398 | 1.25640023256871e-10 | 1.57352495793744e-08 | 9.9008719918825 | 0.226980728051392 | CBFB//THRA//COL1A2//JUN//TGFB2//TGFBR2//KLF10//ADAM9//ACVR1//ZFYVE9//CHRM2//CHRM5//GNAQ//GAB1//APPL1//GRB2//SOS1//PIK3R3//SPRED1//FLRT1//ESRP1//MAPK1//NR5A2//LATS2//PPARG//RARB//ADIPOR2//SNIP1//TNF//PIAS4//DKK3//SOX11//ZEB1//CDKN1A//CRK//CRKL//S1PR1//EREG//F3//IFNA1//IL2//JAK1//MMP2//OSM//PIK3CB//ASPN//CCL2//STAT3//STAT4//LRP8//SOCS5//CEBPB//SESN3//CPEB3//NSMF//SESN1//GLRA1//IPO5//RRAGD//CPEB1//CPEB4//LAMTOR3//TOB1//SOSTDC1//RGMB//TMEM100//RGMA//BMP3//BMPR2//NOG//NPNT//TGFB1I1//BAMBI//PMEPA1//PRDM16//RASL11B//LDLRAD4//SULF1//GATA6//CTDSPL2//ESR1//FHL2//AR//RNF4//ITGA5//CREBRF//SERP1//DERL2//CPEB2//INHBB//ERRFI1//PTEN//SLC2A4//YWHAG//KAT2B//DUSP1//SLIT2//UBA5//TNFRSF21//ST18//ACSL1//HSPA13//IRF1//GSTA2//MSN//RAP1B//SYBU//KDR//DNAJB9//WFS1//EDEM1//FOXC1//BRWD1//NTF3//OTX2//FAM20C//SHISA2//PGRMC2//NR3C2//NR4A2//NR4A3//DSTYK//LHX1//NPTN//NCK1//TSC1//IGF1//IRF9//CNOT7//TCF21//EFNA5//EPHA5//CMKLR1//CCL8//EDN1//SKP1//SQSTM1//RPS6KA4//RPS6KA5//ATXN3//DNAJB12//UBE2W//CDK19//CD274//PLSCR4//PDCD1LG2//PLAA//FZD10//OSR1//MYB//KLF4//PKD2//HCN2//NEUROD1//NME1//SOX4//FOXF1//POU4F1//PTPN4//RAB12//KLF2//MAP3K5//ZFAND6//UGT1A1//PRKAA1//PAX9//TGIF1//URI1//GJA1//ADCY3//GJB2//PRKACB//PRKAR2B//PTGER4//BCL2L11//REST//H2AFZ//LRP6//E2F1//LDLR//ITGA6//P2RY1//TMF1//DNMT3B//WNT7A//KLHL15//BAG6//DRD1//MAP2K4//DLL1//KLF3//PPP1R15B//LPAR1//EPHA4//STMN2//DYNC1LI2//EPS8//PCOLCE2//JARID2//NEFH//PFKP//PIGA//BCAT2//SOX17//ARID5B//GFPT2//LRCH1//CRY2//CLOCK//LMO3//EGR2//POU3F2// |
| GO:0032774 | RNA biosynthetic process | Biological process | 290 | 3940 | 934 | 17653 | 1.3911482733508 | 1.38647427440266e-10 | 1.70485918505185e-08 | 9.8580881842099 | 0.310492505353319 | E2F1//NPAT//RRM2//TSHZ1//HMG20A//KLF2//ZBTB18//CEBPB//ZNF268//TCERG1//WWP2//KLF12//OSR1//CRY2//GLIS3//ARX//DNMT3B//DR1//EDN1//EFNA1//CC2D1B//EN1//ESR1//FHL2//CPEB3//FOXF1//FOXC1//MYT1L//CIC//ZFPM2//DNAJB5//GATA6//CNOT7//HSBP1//AR//JARID2//JUN//ZNF568//MAF//MDM4//MYB//NFIA//NFIB//NR4A2//RLIM//KLF3//ZBTB7A//PIAS4//POU4F1//PPARG//CC2D1A//TSHZ3//ZBTB4//RARB//RBBP7//RBBP8//REST//RNF2//PRDM16//SOX17//SOX11//STAT3//ZEB1//TCF21//TFAP2A//TFAP2C//TGIF1//KLF10//TNF//UBE2I//VLDLR//WFS1//ZNF217//LRP8//BTG2//BHLHE41//TBL1XR1//NR4A3//HMGA2//ARID5B//BRMS1L//BHLHE40//RUNX3//URI1//PER2//SQSTM1//NOG//KLF4//HAND1//FNIP1//PHF14//USP3//GTF2E1//IRF9//KLF6//CREBRF//EGR2//ELK3//ERG//FOXJ3//FOXF2//FLI1//GRHL1//HIVEP2//IRF1//LHX1//MEF2D//MEOX2//MYBL1//NEUROD1//NHLH2//OTX2//PAX9//PBX3//POU3F2//RAD21//ARID4A//SHOX//BCL11B//BMPR2//SOX4//BTF3//TEF//TGFB1I1//THRA//TMF1//DEK//FOSL1//KLF7//CBFB//FUBP3//HAND2//CLOCK//POLR3G//SRSF2//PKD2//BAHD1//H2AFZ//KMT2B//DPY30//TOPORS//KHDRBS3//ZMYND11//TADA1//ZNF597//CTNND2//ZNF782//NKX2-3//ZNF800//E2F5//ZNF367//EZH1//ZBTB9//KDM2A//MYCBP2//FLII//CNOT6L//EPC2//LHX6//HBP1//ATAD2//SERTAD3//MDFIC//MNX1//HMGB3//HOXA3//HOXD1//FOXN2//ZBTB41//ZBTB34//ATXN3//LHX8//NEUROG1//NFE2//NPAS2//IER5//ARID4B//BRWD1//BNC2//ZNF532//CDCA7L//PRKAA1//TRERF1//LMO3//MAPK1//SLC2A4RG//PHTF2//ZNF630//ZNF287//CNOT6//ZNF512B//ZBTB26//MIER1//RBL2//RNF4//ATXN1//DMRTA1//SMARCD2//STAT4//ZNF2//ZNF3//ZNF131//ZNF655//BRD3//TCF7L1//PCGF5//LCOR//WASL//ZBTB47//ST18//HIPK3//NUP35//ELAVL2//NR5A2//NRBF2//MLLT6//RNF141//PPP2CA//PAK6//SNAPC1//BTG1//SNIP1//RPS6KA4//RPS6KA5//CDKN1A//CRK//EMX2//ETS1//BOLA3//NR3C2//PLEK//ZNFX1//NKX2-4//RFX7//BDP1//CDK8//E2F3//TEAD1//KAT2B//CCNC//G3BP2//EREG//MAP3K2//MAPRE3//BAMBI//RGMB//IGF1//LRP6//MAP3K5//WNT7A//ACVR1//DKK3//NRG1//RASD1//TSG101//BTAF1//PPM1F//TOB1//SOSTDC1//TMEM100//RGMA//BMP3//TGFB2//SULF1//CTDSPL2//CMKLR1//EPHA5//INTS8//NABP1//EGLN1//FZD6//S1PR1//DLL1//IL2//ITGA6//MET//PPP1R12A//NCK1//OSM//P2RY1//PPP3R1//DCAF6//CAPRIN2//PTEN//MTPN//EGLN3//RAP2C//TOB2//FBXW7//NPNT// |
| GO:0006351 | transcription, DNA-templated | Biological process | 288 | 3907 | 934 | 17653 | 1.39322327629155 | 1.41420034604155e-10 | 1.70789945362125e-08 | 9.84948906082424 | 0.308351177730193 | E2F1//NPAT//RRM2//TSHZ1//HMG20A//KLF2//ZBTB18//CEBPB//ZNF268//TCERG1//WWP2//KLF12//OSR1//CRY2//GLIS3//ARX//DNMT3B//DR1//EDN1//EFNA1//CC2D1B//EN1//ESR1//FHL2//CPEB3//FOXF1//FOXC1//MYT1L//CIC//ZFPM2//DNAJB5//GATA6//CNOT7//HSBP1//AR//JARID2//JUN//ZNF568//MAF//MDM4//MYB//NFIA//NFIB//NR4A2//RLIM//KLF3//ZBTB7A//PIAS4//POU4F1//PPARG//CC2D1A//TSHZ3//ZBTB4//RARB//RBBP7//RBBP8//REST//RNF2//PRDM16//SOX17//SOX11//STAT3//ZEB1//TCF21//TFAP2A//TFAP2C//TGIF1//KLF10//TNF//UBE2I//VLDLR//WFS1//ZNF217//LRP8//BTG2//BHLHE41//TBL1XR1//NR4A3//HMGA2//ARID5B//BRMS1L//BHLHE40//RUNX3//URI1//PER2//SQSTM1//NOG//KLF4//HAND1//FNIP1//PHF14//USP3//GTF2E1//IRF9//KLF6//CREBRF//EGR2//ELK3//ERG//FOXJ3//FOXF2//FLI1//GRHL1//HIVEP2//IRF1//LHX1//MEF2D//MEOX2//MYBL1//NEUROD1//NHLH2//OTX2//PAX9//PBX3//POU3F2//RAD21//ARID4A//SHOX//BCL11B//BMPR2//SOX4//BTF3//TEF//TGFB1I1//THRA//TMF1//DEK//FOSL1//KLF7//CBFB//FUBP3//HAND2//CLOCK//POLR3G//SRSF2//PKD2//BAHD1//H2AFZ//KMT2B//DPY30//HIPK3//KHDRBS3//ZMYND11//NUP35//ZNF597//CTNND2//ZNF782//ZNF800//ELAVL2//ZBTB9//KDM2A//MYCBP2//FLII//CNOT6L//NR5A2//SERTAD3//NRBF2//HMGB3//ZBTB41//ZBTB34//ATXN3//MLLT6//RNF141//BNC2//PPP2CA//ZNF532//CDCA7L//LMO3//SLC2A4RG//PAK6//PHTF2//ZNF630//CNOT6//ZNF512B//ZBTB26//SNAPC1//BTG1//ZNF2//ZNF3//ZNF131//ZNF655//SNIP1//TCF7L1//WASL//RPS6KA4//RPS6KA5//ZBTB47//CDKN1A//TADA1//CRK//ZNF367//EMX2//ETS1//EPC2//LHX6//HBP1//ATAD2//MNX1//HOXA3//HOXD1//FOXN2//BOLA3//NR3C2//LHX8//NEUROG1//NFE2//ARID4B//PLEK//BRWD1//TRERF1//ZNFX1//MIER1//RBL2//DMRTA1//NKX2-4//RFX7//SMARCD2//BRD3//LCOR//BDP1//CDK8//E2F3//TEAD1//KAT2B//CCNC//G3BP2//EREG//TOPORS//MAP3K2//MAPRE3//BAMBI//RGMB//MDFIC//IGF1//LRP6//MAP3K5//NPAS2//MAPK1//ZNF287//RNF4//WNT7A//ACVR1//DKK3//NRG1//RASD1//ATXN1//TSG101//BTAF1//PPM1F//TOB1//SOSTDC1//TMEM100//RGMA//BMP3//TGFB2//SULF1//CTDSPL2//CMKLR1//EPHA5//INTS8//NABP1//EGLN1//FZD6//NKX2-3//E2F5//S1PR1//EZH1//DLL1//IL2//ITGA6//MET//PPP1R12A//NCK1//OSM//P2RY1//IER5//PPP3R1//DCAF6//CAPRIN2//STAT4//PCGF5//ST18//PTEN//MTPN//EGLN3//PRKAA1//FBXW7//NPNT// |
| GO:0045892 | negative regulation of transcription, DNA-templated | Biological process | 114 | 1176 | 934 | 17653 | 1.83218437267841 | 1.45934243514307e-10 | 1.73149699804782e-08 | 9.83584278881707 | 0.122055674518201 | TSHZ1//HMG20A//KLF2//ZBTB18//CEBPB//ZNF268//TCERG1//WWP2//KLF12//OSR1//CRY2//GLIS3//ARX//DNMT3B//DR1//E2F1//EDN1//EFNA1//CC2D1B//EN1//ESR1//FHL2//CPEB3//FOXF1//FOXC1//MYT1L//CIC//ZFPM2//DNAJB5//GATA6//CNOT7//HSBP1//AR//JARID2//JUN//ZNF568//MAF//MDM4//MYB//NFIA//NFIB//NR4A2//RLIM//KLF3//ZBTB7A//PIAS4//POU4F1//PPARG//CC2D1A//TSHZ3//ZBTB4//RARB//RBBP7//RBBP8//REST//RNF2//PRDM16//SOX17//SOX11//STAT3//ZEB1//TCF21//TFAP2A//TFAP2C//TGIF1//KLF10//TNF//UBE2I//VLDLR//WFS1//ZNF217//LRP8//BTG2//BHLHE41//TBL1XR1//NR4A3//HMGA2//ARID5B//BRMS1L//BHLHE40//RUNX3//URI1//PER2//SQSTM1//NOG//KLF4//HAND1//FNIP1//PHF14//USP3//BAHD1//H2AFZ//DPY30//THRA//ATAD2//MIER1//NCK1//ELK3//EREG//FOXF2//DKK3//MDFIC//NRG1//IRF1//LHX1//PAX9//RASD1//ARID4A//ATXN1//TSG101//BTAF1//RPS6KA5//CLOCK//PPM1F// |
| GO:0051174 | regulation of phosphorus metabolic process | Biological process | 157 | 1806 | 934 | 17653 | 1.64306048598415 | 1.5076868047161e-10 | 1.73421636291986e-08 | 9.8216888660991 | 0.168094218415418 | CDKN1A//GADD45A//PTEN//CCNE2//MINK1//TNF//MAP3K2//CRK//CRKL//MAP3K5//TAOK3//LAMTOR3//ADAM9//DUSP5//LPAR1//LPAR3//NRG1//IGF1//NTF3//PIK3CB//PRKAA1//MAPK1//MAP2K4//DUSP10//SPRED1//DUSP1//DUSP2//PPP2CA//RGS3//DRD1//FNIP1//FAM129A//IL2//LRP6//PPP1R15B//SLIT2//EFNA1//EFNA5//EPHA7//FMR1//NPTN//KDR//P2RY1//AKTIP//SQSTM1//CCND2//EREG//FBXW7//ERRFI1//TSG101//SOCS5//NUP35//NUP43//PPM1E//FLRT1//GNAQ//NCK1//NF2//ASPN//DUS2//YWHAG//CAMK2N2//PPM1F//COPS8//MDFIC//TGFB2//NPNT//CALM2//GDF11//INHBB//BMP3//BMPR2//ACVR1//LDLR//PLEK//MTMR9//PPP1R37//PPP1R12A//PPP1R12C//PLEKHF2//URI1//PKD2//TBL1XR1//ADRB1//PTHLH//EGLN1//CDK6//JUN//RAP2C//PAK6//TGFBR2//SLK//CRY2//MASTL//RPS6KA4//RPS6KA5//OSM//PFN2//CAPRIN2//IFNA1//EPHA2//EPHA4//EPHA5//DSTYK//MET//ADCY3//PRKACB//PRKAR2B//DLC1//CNEP1R1//NSMF//PTGES3//KCTD20//ITGA6//AR//BTBD10//CNOT7//STAT3//EDN1//GRB2//ITGAV//FZD10//HIPK3//AIDA//TWF1//NRBF2//RBL2//PIK3R3//KLF4//UBXN2B//PPP4R2//TSC1//LATS2//IPO5//KAT2B//CCNY//MAPRE3//CCNC//MMD//VLDLR//ZMYND11//WNT7A//ITGA5//LRP8//CHRM5//PMEPA1//LDLRAD4//NOG//IDH1//RANBP9//LMO3//DNAJC27//CCL2//CCL8//HAND2//ACSL1//FAM20C// |
| GO:0019220 | regulation of phosphate metabolic process | Biological process | 156 | 1791 | 934 | 17653 | 1.64626845863866 | 1.51291979021546e-10 | 1.73421636291986e-08 | 9.82018409616069 | 0.167023554603854 | CDKN1A//GADD45A//PTEN//CCNE2//MINK1//TNF//MAP3K2//CRK//CRKL//MAP3K5//TAOK3//LAMTOR3//ADAM9//DUSP5//LPAR1//LPAR3//NRG1//IGF1//NTF3//PIK3CB//PRKAA1//MAPK1//MAP2K4//DUSP10//SPRED1//DUSP1//DUSP2//PPP2CA//RGS3//DRD1//FNIP1//FAM129A//IL2//LRP6//PPP1R15B//SLIT2//EFNA1//EFNA5//EPHA7//FMR1//NPTN//KDR//P2RY1//AKTIP//SQSTM1//CCND2//EREG//FBXW7//ERRFI1//TSG101//SOCS5//NUP35//NUP43//PPM1E//FLRT1//GNAQ//NCK1//NF2//ASPN//DUS2//YWHAG//CAMK2N2//PPM1F//COPS8//MDFIC//TGFB2//NPNT//CALM2//GDF11//INHBB//BMP3//BMPR2//ACVR1//LDLR//PLEK//MTMR9//PPP1R37//PPP1R12A//PPP1R12C//PLEKHF2//URI1//PKD2//TBL1XR1//ADRB1//PTHLH//EGLN1//CDK6//JUN//RAP2C//PAK6//TGFBR2//SLK//CRY2//MASTL//RPS6KA4//RPS6KA5//OSM//PFN2//CAPRIN2//IFNA1//EPHA2//EPHA4//EPHA5//DSTYK//MET//ADCY3//PRKACB//PRKAR2B//DLC1//CNEP1R1//NSMF//PTGES3//KCTD20//ITGA6//AR//BTBD10//CNOT7//STAT3//EDN1//GRB2//ITGAV//FZD10//HIPK3//AIDA//TWF1//NRBF2//RBL2//PIK3R3//KLF4//UBXN2B//PPP4R2//TSC1//LATS2//IPO5//KAT2B//CCNY//MAPRE3//CCNC//MMD//VLDLR//ZMYND11//WNT7A//ITGA5//LRP8//CHRM5//PMEPA1//LDLRAD4//NOG//IDH1//RANBP9//LMO3//DNAJC27//CCL2//CCL8//HAND2//ACSL1// |
| GO:0052697 | xenobiotic glucuronidation | Biological process | 9 | 11 | 934 | 17653 | 15.463986762702 | 1.56401805809496e-10 | 1.74000777797723e-08 | 9.80575823689911 | 0.00963597430406852 | UGT1A10//UGT1A8//UGT1A7//UGT1A6//UGT1A5//UGT1A9//UGT1A4//UGT1A1//UGT1A3// |
| GO:0032502 | developmental process | Biological process | 423 | 6267 | 934 | 17653 | 1.27571105095212 | 1.56942886968226e-10 | 1.74000777797723e-08 | 9.80425836279574 | 0.452890792291221 | WNT7A//VASH2//FRY//KLF2//CDH20//NRG1//TGFB2//GDF11//CMKLR1//COL1A2//HAPLN1//EN1//FRZB//SERP1//GNAQ//IGF1//PTHLH//SHOX//BMP3//SOX4//SOX11//KLF10//PKDCC//NOG//THRA//S1PR1//EFNA1//EPHA2//ELK3//EREG//FOXC1//GAB1//CLIC4//HOXA3//ITGA5//ITGAV//JUN//KDR//MEOX2//MMP2//TMEM100//PTEN//CCL2//SOX17//VEGFB//HAND1//HAND2//CEBPB//INHBB//DMRTA1//IMMP2L//KMT2B//ESR1//PLEKHA1//YTHDF2//CRKL//FOXF1//SPHK2//TGFBR2//EDN1//PPP3R1//ACVR1//GJC1//ZFPM2//QKI//FHL2//GJA1//MEF2D//CBFB//SMOC1//OSR1//LHX1//RDH10//NPNT//RARB//TCF21//SLIT2//PKD2//TBC1D20//AGFG1//TMF1//BCL2L11//ADAM10//GATA6//EPN1//AR//ZNF568//MAN2A1//MBNL1//BTF3//FOSL1//LRP6//RNF2//DUSP1//DUSP2//DUSP5//NF2//BMPR2//NR4A3//DLL1//DSCAML1//STAT3//SEMA4B//DCX//GPM6A//LRP12//NR4A2//SPOCK1//UNK//SULF1//TFAP2A//TSC1//WFS1//BAG6//ARID5B//LATS2//RBBP8//FOXF2//HMGA2//DLC1//PRKACB//FZD6//MET//JARID2//UGT1A1//PPARG//APCDD1//SOS1//FBXW7//RECK//ARHGAP12//NFIB//RUNX3//ESCO2//PLEK//RBM47//REST//NKX2-3//FNIP1//MARCH7//NME1//ERG//POU4F1//CDK6//NEUROD1//PALLD//BCL11B//FRMD6//SLC17A7//BNC2//TSHZ1//EPHA4//EPHA5//EPHA7//PRR15//DZIP1//WDR47//WDFY3//FLII//LCLAT1//TNFRSF21//HIVEP2//HMGB3//SHISA2//NFE2//OSM//MINK1//KLF3//ZBTB7A//ZFR//BTBD7//TRERF1//TSHZ3//RBBP7//NKX2-4//TEF//TGIF1//ZNF3//ZNF217//PTP4A1//EVI5//VANGL1//ST6GAL2//ADAM18//TDRD5//EZH1//FOXJ3//DKK3//MNX1//NEUROG1//PBX3//SOSTDC1//ASPH//ZEB1//E2F5//STX2//FLI1//BHLHE41//NR5A2//AMER2//TRIM3//FUT9//CHRM2//SCRG1//RBFOX3//DPYSL2//EFNA5//MYT1L//FMR1//GLRB//MYLIP//ATXN3//NTF3//TAOK3//POU3F2//VLDLR//ZIC5//ITM2B//FEZ2//NTNG1//SLITRK3//NEFH//OGN//RANBP9//ARX//GRB2//KIF5A//OTX2//PIK3CB//ENAH//MAPK1//NTN4//KLF7//RPS6KA5//DRD1//NHLH2//NPAS2//SERPINI1//FXR1//B3GNT5//EGR2//RAB3GAP1//CIC//PTPRG//ATXN1//PAX9//PPP2CA//KLF4//ADIPOR2//KAT2B//SGCE//ZBTB18//CFL2//ADAM12//SLC32A1//NAPEPLD//GJB2//P2RY1//MORC3//CDC42EP3//WASF3//PALM2//FGD4//CRK//FGD5//LPAR1//EPS8//BAMBI//RHOC//RND3//MSN//BRWD1//WASL//CLDN11//GRHL1//LAMA3//RNF38//TFAP2C//IDH1//LHX8//ACO1//RAI2//EMX2//BTG2//AIDA//BNIP3//ITGA6//TGFB1I1//LDLRAD4//TOPORS//FJX1//SNAP25//ZNF804A//STMN2//NPTN//NCK1//TWF1//BAG5//KBTBD8//MTPN//FLRT1//STRN//SPRED1//DDX6//LRP8//HPRT1//CALM2//LHX6//ETS1//C21ORF91//TACC2//PRDM16//TCF7L1//KCNJ10//SH3D19//MKNK2//ZNF268//FOXN2//JAK1//MYB//MYBL1//RBL2//ARHGEF28//SQSTM1//FZD10//HOXD1//KLF6//IFNA1//DNAJB9//TSG101//ADAM9//IL2//TMOD3//TMOD1//ACTC1//ASPN//OSTM1//TNF//FAM20C//RAB21//CNN3//GSTA2//KIAA1109//E2F1//KRT27//CDKN1A//PSD//DSC2//PIKFYVE//MAF//LRIG1//SYCP3//SLC12A5//TBL1XR1//RAB38//HSBP1//ADRB1//PTGER4//SOBP//SBF2//IRF1//ARRDC3//ERRFI1//TMEFF2//TMEM64//DUSP10//PLEKHB2//ZADH2//CCDC71L//LMO3//BTG1//SOCS5//DPY30//CXCL14//MAP3K5//YWHAG//HMG20A//DNMT3B//MMD//TOB1//TOB2//EGLN1//F3//ITGB8//GOLGA4//LHFPL2//PHF14//TEAD1//PRKAA1//ULK2//LPAR3//RGMA//SLC35D1//RHEB//NSMF//HECW2//ARID4A//PER2//CAPRIN2//SLC2A4//MDM1//NFIA//SYBU//MFAP5//CTNND2//CPEB3//RAP1B//MAP2K4//NEFM//LDLR//KLHL3//SNX2//TMEM135//MIEF1//DDHD1//RAP2C//RAB23//ARID4B//NANOS1//DOCK5//NLN//PLAA//CLOCK// |
| GO:0048518 | positive regulation of biological process | Biological process | 399 | 5841 | 934 | 17653 | 1.29109242902659 | 1.7469945119285e-10 | 1.90563288454394e-08 | 9.75770845932326 | 0.427194860813705 | GADD45A//MINK1//TNF//MAP3K2//CRK//CRKL//MAP3K5//TAOK3//LAMTOR3//ADAM9//DUSP5//LPAR1//LPAR3//NRG1//IGF1//NTF3//PIK3CB//PRKAA1//MAPK1//MAP2K4//EREG//TMF1//EPS15//NSF//FAM129A//EFNA1//EFNA5//EPHA7//FMR1//NPTN//KDR//P2RY1//AKTIP//SQSTM1//CCND2//FNIP1//F3//JUN//BMPR2//VEGFB//VASH2//CDK6//ADRB1//ARL6IP1//FOXF1//TGFBR2//PRKACB//SKP1//RPS6KA5//BAG6//OSM//MIA3//CCL8//EDN1//GJA1//FBXW7//KMT2B//DLC1//EGLN3//PMAIP1//PPARG//CDKN1A//E2F1//CNOT6L//CNOT7//MDM4//CNOT6//RBL2//SOX4//BTG2//MTBP//KAT2B//COPS8//MDFIC//ADAM10//ZNF268//E2F3//ETS1//BAMBI//DLL1//HOXA3//IL2//AR//ITGAV//DERL2//POU3F2//CDCA7L//SPHK2//PTEN//PTHLH//RARB//SOX11//TGFB2//FOSL1//NPNT//PIGA//TOB1//EPS8//ARHGAP1//ARHGAP6//BNIP3//TRPC3//MTPN//PLEK//SULF1//WNT7A//PAN3//TWF1//NR4A3//HAND2//OSR1//ARX//DNMT3B//DRD1//RAB3GAP1//ITGB8//LDLR//MSN//POU4F1//STAT3//ACTC1//TFAP2A//HMGA2//FUBP3//ZNF804A//NOG//KLF4//QKI//PPM1F//DOCK5//TMED9//GRB2//FOXC1//TGFB1I1//CMKLR1//CALM2//ITGA6//ITGA5//SMOC2//THRA//GDF11//INHBB//BMP3//ACVR1//CNEP1R1//MTMR9//PCOLCE2//STMN2//NCK1//NME1//SERPINI1//SLC6A1//CPEB3//SERP1//PRR16//FXR1//ADIPOR2//SESN3//RAB12//SESN1//TSC1//TOPORS//KLF2//KLF6//EGR2//ESR1//FHL2//MAPRE3//FOXF2//FLI1//NR5A2//GATA6//RGMB//ATAD2//SERTAD3//FOXN2//IRF1//LHX1//LRP6//MYB//MYBL1//NEUROD1//NFE2//NPAS2//NPAT//OTX2//TRERF1//ZNF287//REST//RNF4//PRDM16//SOX17//TEAD1//TBL1XR1//RUNX3//CLOCK//DENND1B//TNFRSF21//ENPP4//SEMA4B//S1PR1//CD274//PTP4A1//FAM20C//PKDCC//CFL2//CDC42EP3//PFN2//MET//SLAIN2//CLIP1//PDCD1LG2//NDFIP2//WFS1//NEUROG1//PKD2//WASF3//MIER1//RAP2C//RRAGD//RHEB//PAK6//SLK//CCT6A//STIM2//RNF180//ANKIB1//LONRF3//LONRF1//SOCS5//BCL2L11//RHOC//KIF23//POLR3G//PPP1R15B//CEBPB//EPHA5//LRP8//RPS6KA4//CAPRIN2//IFNA1//PIAS4//EPHA2//EPHA4//DSTYK//ADCY3//PRKAR2B//IQGAP2//ARFIP1//NSMF//LMO3//PPP1R12A//SYBU//GAB1//WASL//NPLOC4//IPO5//PTGES3//KCTD20//BTBD10//FGD4//DUSP1//CDK19//FRZB//LATS2//USP27X//ARHGEF4//MOAP1//ZMAT3//UBE2Z//SOS1//SOS2//NSMAF//SLIT2//CC2D1A//SLC20A1//UBE2I//FZD10//SPRED1//TBC1D20//MMP2//DUSP10//NF2//CCDC71L//TMEM64//TMEM100//BTG1//MMD//ZEB1//KLF10//MID1IP1//WWP2//MYLIP//HECW2//CCNY//CCNC//ADAM12//GOLGA4//TOB2//DEK//VLDLR//ASPH//RAD21//CDK8//ZBTB18//CREBRF//NKX2-3//GLIS3//E2F5//ELK3//EN1//ERG//EZH1//FOXJ3//ZFPM2//GRHL1//H2AFZ//MAF//MEF2D//MEOX2//NFIA//NFIB//NHLH2//NR4A2//PAX9//PBX3//IER5//ARID4B//EGLN1//PPP3R1//DCAF6//RGMA//ARID4A//SHOX//BCL11B//STAT4//TCF21//TEF//TFAP2C//PCGF5//KLF7//CBFB//HAND1//ST18//RAET1E//HPRT1//RASGEF1A//CTDSPL2//RAB21//TSG101//PTGER4//MAN2A1//PLEKHA1//CCL2//SH3D19//ARID5B//ARRDC3//ARRDC4//GPM6A//PPM1E//JARID2//SLITRK3//FLRT1//TSHZ3//SLC24A2//SNAP25//C21ORF91//YTHDF2//DNAJC27//MCOLN2//ACSL1//PPP2CA//MIEF1//DDHD1//NANOS1//CD2AP//PAIP2//DYNLL2//YWHAG//CALCOCO2//FYCO1//TMOD3//CHMP3//SERINC3//FBXL5//PLAA//JAK1//ELMOD1//SNIP1//EDEM1//ATXN3//RNF128//CENPQ//LHFPL2//STK17B//GLRA1//CXCL14//GLRX//PIK3R3// |
| GO:0050794 | regulation of cellular process | Biological process | 674 | 11074 | 934 | 17653 | 1.15034212127177 | 2.64500492153596e-10 | 2.8393917911663e-08 | 9.57757351554036 | 0.721627408993576 | E2F1//USP28//CLOCK//CDKN1A//GADD45A//PTEN//CCNE2//NPAT//RRM2//TSHZ1//HMG20A//KLF2//ZBTB18//CEBPB//ZNF268//TCERG1//WWP2//KLF12//OSR1//CRY2//GLIS3//ARX//DNMT3B//DR1//EDN1//EFNA1//CC2D1B//EN1//ESR1//FHL2//CPEB3//FOXF1//FOXC1//MYT1L//CIC//ZFPM2//DNAJB5//GATA6//CNOT7//HSBP1//AR//JARID2//JUN//ZNF568//MAF//MDM4//MYB//NFIA//NFIB//NR4A2//RLIM//KLF3//ZBTB7A//PIAS4//POU4F1//PPARG//CC2D1A//TSHZ3//ZBTB4//RARB//RBBP7//RBBP8//REST//RNF2//PRDM16//SOX17//SOX11//STAT3//ZEB1//TCF21//TFAP2A//TFAP2C//TGIF1//KLF10//TNF//UBE2I//VLDLR//WFS1//ZNF217//LRP8//BTG2//BHLHE41//TBL1XR1//NR4A3//HMGA2//ARID5B//BRMS1L//BHLHE40//RUNX3//URI1//PER2//SQSTM1//NOG//KLF4//HAND1//FNIP1//PHF14//USP3//RANBP9//PPM1L//SPRED1//DUSP5//EREG//RASGEF1A//GRB2//NRG1//IL2//JAK1//MAP3K5//MET//MINK1//TAOK3//MAPK1//CCL2//SOS1//LAMTOR3//MAP3K2//CRK//CRKL//ADAM9//LPAR1//LPAR3//IGF1//NTF3//PIK3CB//PRKAA1//MAP2K4//DUSP10//DUSP1//DUSP2//PPP2CA//RGS3//RBFOX3//FMR1//MBNL1//RBM25//SRSF2//SECISBP2L//NANOS1//SPOCK1//TSG101//KIAA1109//PMAIP1//BCL2L11//MOAP1//EPS15//NSF//DRD1//FAM129A//LRP6//PPP1R15B//SLIT2//EFNA5//EPHA7//NPTN//KDR//P2RY1//AKTIP//CCND2//SULF1//GJA1//F3//BMPR2//VEGFB//VASH2//NF2//CBFB//TGFBR2//THRA//PRKACB//SKP1//RPS6KA5//BAG6//MIA3//CCL8//NME1//HAND2//S1PR1//GPR6//SPHK2//PKD2//E2F5//IRF1//TSC1//MASTL//CCNG2//EPS8//TWF1//APCDD1//CTNND2//AMER2//CCNY//SOSTDC1//HBP1//DKK3//STRN//TGFB1I1//WNT7A//TCF7L1//PPP3R1//FZD6//FAM13A//CNIH1//CDK6//DLC1//CDS1//CDC42EP3//GNB5//PTGES3//IQGAP2//ADCY3//RASSF8//ARAP2//TAGAP//CLCN6//DPYSL2//ELK3//STX2//ERG//EVI2A//LRCH1//CD2AP//APPL1//GLRA1//GLRB//RASSF3//RGMB//GRIA1//CD274//LRP12//HIVEP2//NR3C2//MPP1//PPP1R12A//RASD1//KCNK10//NDFIP2//PPP1R12C//PSD//ARRDC3//MIER1//RHEB//SMOC1//SMOC2//BCL11B//SLC20A1//RASL11B//DEK//PDCD1LG2//CAMLG//NSMAF//PPFIA1//RHPN2//OR6A2//ARRDC4//TRIP10//PLAA//ARHGAP12//CXCL14//FEZ2//CD69//ULK2//PTPRG//AKAP11//DGKH//DCX//PIKFYVE//DSTYK//LATS2//MKNK2//ARHGEF4//TREM1//PRKAR2B//ARHGEF28//STK33//DGKE//RPS6KA4//STK17B//PALM2//FBXW7//ERRFI1//SOCS5//NUP35//NUP43//NLN//DNAJC16//GLRX//NHLRC2//TMX3//ESCO2//BAHD1//H2AFZ//KMT2B//DPY30//HIPK3//KHDRBS3//ZMYND11//ZNF597//ZNF782//ZNF800//ELAVL2//ZBTB9//KDM2A//MYCBP2//FLII//CNOT6L//NR5A2//GTF2E1//SERTAD3//NRBF2//HMGB3//ZBTB41//ZBTB34//ATXN3//MLLT6//RNF141//BNC2//ZNF532//CDCA7L//LMO3//SLC2A4RG//PAK6//PHTF2//ZNF630//CNOT6//ZNF512B//ZBTB26//SNAPC1//SOX4//BTF3//BTG1//TMF1//ZNF2//ZNF3//ZNF131//ZNF655//SNIP1//WASL//ZBTB47//IRF9//TADA1//ZNF367//EMX2//ETS1//FOXJ3//FOXF2//FLI1//EPC2//LHX6//ATAD2//GRHL1//MNX1//HOXA3//HOXD1//FOXN2//BOLA3//LHX8//MYBL1//NEUROG1//NFE2//NHLH2//ARID4B//PLEK//BRWD1//POU3F2//TRERF1//ZNFX1//RAD21//ARID4A//RBL2//DMRTA1//NKX2-4//RFX7//SMARCD2//TEF//BRD3//FOSL1//LCOR//KLF7//POLR3G//BDP1//MTPN//DDX6//ACO1//KBTBD8//PPM1E//FLRT1//GNAQ//NCK1//ASPN//DUS2//YWHAG//CAMK2N2//PPM1F//EGLN3//STMN2//MID1IP1//MTBP//RRAGD//TGFB2//KAT2B//CDC23//NABP1//CDC14A//ADAM10//GAB1//COL1A2//ACVR1//ZFYVE9//PDE10A//RAPGEF4//FZD10//CHRM2//CHRM5//FGD4//CMKLR1//ADRB1//GPR180//FRZB//RHOC//GPRC5C//PTGER4//PTHLH//SOS2//CALM2//DLL1//EPN1//TMEM100//TMEM17//DZIP1//ITGA6//ITGA5//ITGAV//ITGB8//NKIRAS1//COPS8//G3BP2//MDFIC//STAT4//NEUROD1//RGL1//DOCK9//RAB38//RND3//ARHGAP1//RALGPS2//DOCK5//ARHGAP6//CDK8//CDK19//SLK//TRPC3//MARCKS//SNAP25//TOPORS//EPHA2//EPHA4//EPHA5//ARL6IP1//ZFAND6//BNIP3//ACTC1//GIT2//E2F3//BAMBI//OSM//DERL2//TOB1//GDF11//TOB2//BTG3//TES//ASPH//ST18//PIK3R3//WASF3//FGD5//MSN//ESRP1//OTX2//ADIPOR2//RGS17//NEDD1//HAUS8//CEP41//IFNA1//INHBB//OGN//BMP3//TTR//MTMR9//PPP4R2//PAN3//PFN2//TMED9//NPNT//LDLRAD4//C5ORF30//CHMP3//CNEP1R1//LDLR//PPP1R37//PLEKHF2//SERPINI1//RECK//PCOLCE2//ZNF804A//MYLIP//BAG5//PMEPA1//PLEKHA1//SERP1//PRR16//FXR1//SESN3//RAB12//SESN1//SNX5//TNFRSF21//ANP32E//KLF6//EGR2//MAPRE3//LHX1//NPAS2//ZNF287//RNF4//TEAD1//FUBP3//PAX9//ATXN1//BTAF1//CAPRIN2//RAB21//SYT6//TRPV6//MMP2//SH3D19//DENND1B//HECW2//RUFY2//SHISA2//LAMA3//SEMA4B//PTP4A1//TMEFF2//CLIC4//RAP2C//RGMA//CTDSPL2//EGLN1//LIMA1//CFL2//CREBRF//SLAIN2//CLIP1//TBC1D12//EVI5//TBC1D17//USP6NL//SPOPL//AGFG1//AGFG2//DCP2//CCT6A//USP46//SLC6A1//PKDCC//RNF180//ANKIB1//LONRF3//LONRF1//KIF13A//KIF23//RAB30//RAB9B//DNAJC27//RAB23//RAB22A//RAB28//RAP1B//MEMO1//FRMD6//UBA5//ACSL1//ARFIP1//CPEB4//NSMF//GSTA2//SYBU//DNAJB9//EDEM1//NPLOC4//FAM20C//RNF128//NKX2-3//IER5//MARCH7//KCTD20//BTBD10//ITM2B//LYPLA1//USP27X//ZMAT3//UBE2Z//PGRMC2//FASTK//AHNAK//YTHDF2//AIDA//UBXN2B//PEX5L//TMEM64//PLEKHB2//ZADH2//CCDC71L//MMD//CEP97//IPO5//CCNC//GOLGA4//UGT1A8//EZH1//MEF2D//MEOX2//PBX3//DCAF6//SHOX//PCGF5//PAIP2//HPRT1//MDM1//MORC3//KCNJ10//RAB3GAP1//MAN2A1//PXK//NOVA1//GPM6A//TMOD3//TMOD1//OTUD3//SLITRK3//VANGL1//SLC17A7//SLC24A2//IDH1//C21ORF91//QKI//FIBIN//SACS//TMEM135//MIEF1//DDHD1//SGPP1//DSC2//LYPD6//ORMDL3//CPEB2//DYNLL2//CALCOCO2//FYCO1//CRTAP//SERINC3//NEFH//TBC1D20//FBXL5//ELMOD1//FRY//CTSA//KLHL15//IER3IP1//CPEB1//UNK// |
| GO:0030182 | neuron differentiation | Biological process | 122 | 1303 | 934 | 17653 | 1.76964869408596 | 2.78212161830221e-10 | 2.93992007884029e-08 | 9.55562388908047 | 0.130620985010707 | STAT3//SOX4//SOX11//HAND2//NTNG1//SLITRK3//NEFH//OGN//POU4F1//DSCAML1//WNT7A//SLIT2//RANBP9//ARX//DPYSL2//EFNA1//EFNA5//EPHA5//GAB1//GRB2//KIF5A//OTX2//PIK3CB//ENAH//MAPK1//NTN4//SOS1//VLDLR//NR4A3//KLF7//RPS6KA5//FEZ2//EGR2//EPHA4//LHX1//NOG//PTEN//SNAP25//ZNF804A//STMN2//NPTN//NCK1//NME1//SERPINI1//TWF1//LPAR1//MYLIP//PTPRG//SPOCK1//TSC1//BAG5//GNAQ//FLRT1//STRN//MNX1//MTPN//BCL11B//DRD1//LHX6//LHX8//HPRT1//C21ORF91//NR4A2//BTG2//NFIB//RAB21//JUN//FRY//GPM6A//LRP12//PSD//EPHA7//TOPORS//NEUROD1//DLL1//NTF3//YWHAG//HMG20A//DDX6//REST//DNMT3B//MMD//NEUROG1//RARB//ZEB1//GOLGA4//NRG1//MYT1L//POU3F2//EN1//FMR1//PBX3//TGFB2//FXR1//UNK//ULK2//LPAR3//KLF4//RGMA//GJA1//MINK1//NSMF//HECW2//BMPR2//SEMA4B//RUNX3//CAPRIN2//SLC12A5//CTNND2//WASL//CPEB3//IL2//LRP8//NEFM//LRP6//NANOS1//PLAA//CEBPB//FZD10//S1PR1//EMX2//HOXD1// |
| GO:0010033 | response to organic substance | Biological process | 244 | 3218 | 934 | 17653 | 1.43309648750404 | 5.12091843428022e-10 | 5.32811867246725e-08 | 9.29065214153021 | 0.261241970021413 | CBFB//DRD1//HPRT1//NR4A2//THRA//DNAJB4//UBXN4//DNAJB5//JKAMP//HERPUD2//HSPA13//COL1A2//JUN//TGFB2//TGFBR2//KLF10//ADAM9//ACVR1//ZFYVE9//CHRM2//CHRM5//GNAQ//GAB1//APPL1//GRB2//SOS1//PIK3R3//SPRED1//FLRT1//ESRP1//MAPK1//FHL2//STEAP2//RAP1B//SLC6A1//GJA1//IGF1//NEUROD1//PTEN//NR5A2//LATS2//PPARG//RARB//ADIPOR2//SNIP1//CDKN1A//TNF//PIAS4//ACSL1//BTG2//NME1//DKK3//SOX11//ZEB1//CRK//CRKL//S1PR1//EREG//F3//IFNA1//IL2//JAK1//MMP2//OSM//PIK3CB//ASPN//CCL2//STAT3//STAT4//LRP8//SOCS5//GPR180//CEBPB//SESN3//CPEB3//NSMF//SESN1//GLRA1//IPO5//RRAGD//CPEB1//CPEB4//LAMTOR3//DNAJB9//DERL2//NPLOC4//WFS1//BAG6//EDEM1//TOB1//SOSTDC1//RGMB//TMEM100//RGMA//BMP3//BMPR2//NOG//NPNT//TGFB1I1//BAMBI//PMEPA1//PRDM16//RASL11B//LDLRAD4//SULF1//GATA6//CTDSPL2//ESR1//AR//RNF4//ITGA5//CREBRF//SERP1//DNMT3B//PRKAA1//DUSP1//ETS1//GJB2//RBBP8//WNT7A//DUSP10//EDN1//TNFRSF21//UGT1A1//PTGER4//FOSL1//EGR2//TSC1//CPEB2//INHBB//ERRFI1//SLC2A4//YWHAG//KAT2B//SLIT2//UBA5//SRSF2//TRPC3//ST18//CD274//CALCOCO2//ADAM10//IRF1//GSTA2//MSN//SYBU//KDR//DNAJB12//FOXC1//BRWD1//NTF3//OTX2//FAM20C//SHISA2//GLRB//PMAIP1//PGRMC2//NR3C2//NR4A3//LRP6//BTG1//DSTYK//LHX1//RPL15//ACTC1//EPS8//USP46//NPTN//NCK1//IDH1//RBBP7//EPHA2//P2RY1//IRF9//CNOT7//KLF4//TCF21//EFNA5//EPHA5//CMKLR1//CCL8//SKP1//SQSTM1//RPS6KA4//RPS6KA5//ATXN3//UBE2W//CDK19//PLSCR4//PDCD1LG2//PLAA//FZD10//OSR1//MYB//POU3F2//PKD2//HCN2//SOX4//FOXF1//POU4F1//PTPN4//RAB12//KLF2//MAP3K5//ZFAND6//PAX9//TGIF1//URI1//ADCY3//PRKACB//PRKAR2B//BCL2L11//REST//H2AFZ//E2F1//LDLR//ITGA6//TMF1//FIBIN//RUNX3//KLHL15//MAP2K4//FMR1//DLL1//KLF3//PPP1R15B//LPAR1//EPHA4//STMN2//DYNC1LI2//PCOLCE2//JARID2//NEFH//PFKP//PIGA//BCAT2//SOX17//ARID5B//GFPT2//LRCH1//CRY2//CLOCK//LMO3//PPP2CA// |
| GO:0051172 | negative regulation of nitrogen compound metabolic process | Biological process | 205 | 2591 | 934 | 17653 | 1.49540246794 | 6.15086158791832e-10 | 6.2715501409115e-08 | 9.2110640457416 | 0.21948608137045 | TSHZ1//HMG20A//KLF2//ZBTB18//CEBPB//ZNF268//TCERG1//WWP2//KLF12//OSR1//CRY2//GLIS3//ARX//DNMT3B//DR1//E2F1//EDN1//EFNA1//CC2D1B//EN1//ESR1//FHL2//CPEB3//FOXF1//FOXC1//MYT1L//CIC//ZFPM2//DNAJB5//GATA6//CNOT7//HSBP1//AR//JARID2//JUN//ZNF568//MAF//MDM4//MYB//NFIA//NFIB//NR4A2//RLIM//KLF3//ZBTB7A//PIAS4//POU4F1//PPARG//CC2D1A//TSHZ3//ZBTB4//RARB//RBBP7//RBBP8//REST//RNF2//PRDM16//SOX17//SOX11//STAT3//ZEB1//TCF21//TFAP2A//TFAP2C//TGIF1//KLF10//TNF//UBE2I//VLDLR//WFS1//ZNF217//LRP8//BTG2//BHLHE41//TBL1XR1//NR4A3//HMGA2//ARID5B//BRMS1L//BHLHE40//RUNX3//URI1//PER2//SQSTM1//NOG//KLF4//HAND1//FNIP1//PHF14//USP3//DUSP10//SPRED1//DUSP1//DUSP2//DUSP5//PPP2CA//RGS3//FAM129A//IL2//LRP6//PTEN//PPP1R15B//SLIT2//ERRFI1//TSG101//SOCS5//BAHD1//H2AFZ//DPY30//GADD45A//PPM1E//FLRT1//GNAQ//NCK1//NF2//TAOK3//ASPN//DUS2//YWHAG//CAMK2N2//PPM1F//SNIP1//CALM2//SERPINI1//SPOCK1//RECK//ELK3//EREG//FOXF2//DKK3//MDFIC//NRG1//IRF1//LHX1//PAX9//RASD1//ARID4A//ATXN1//THRA//BTAF1//RPS6KA5//CLOCK//TOB1//FMR1//NANOS1//CAPRIN2//TSC1//FXR1//EGLN1//SPOPL//SOX4//BAG5//CDK6//ATAD2//MIER1//DCP2//BAG6//PPP1R37//MASTL//FBXW7//RNF4//CNOT6//RNF128//ITM2B//ARL6IP1//HIPK3//AIDA//ITGAV//CDKN1A//LATS2//IPO5//KAT2B//PAIP2//PRKAA1//ZMYND11//INHBB//NTF3//LDLR//PMEPA1//LDLRAD4//NUP35//NUP43//CNOT6L//YTHDF2//QKI//RANBP9//LMO3//PLAA//ORMDL3//CPEB2//CRTAP//EPHA4//TOB2//NPAT//SRSF2//DERL2//FRY//CTSA//KLHL15//GJA1//BMPR2//PRKAR2B//CPEB1//CPEB4//UNK// |
| GO:0065009 | regulation of molecular function | Biological process | 253 | 3373 | 934 | 17653 | 1.41767220610072 | 6.21312818928095e-10 | 6.2715501409115e-08 | 9.20668968592929 | 0.270877944325482 | CDKN1A//GADD45A//PTEN//CCNE2//MINK1//TNF//MAP3K2//CRK//CRKL//MAP3K5//TAOK3//LAMTOR3//ADAM9//DUSP5//LPAR1//LPAR3//NRG1//IGF1//NTF3//PIK3CB//PRKAA1//MAPK1//MAP2K4//DUSP10//SPRED1//DUSP1//DUSP2//PPP2CA//RGS3//PKD2//THRA//EREG//FBXW7//ERRFI1//TSG101//SOCS5//PPM1E//FLRT1//GNAQ//LRP6//NCK1//NF2//ASPN//DUS2//YWHAG//CAMK2N2//PPM1F//DLC1//EGLN3//F3//PMAIP1//PPARG//EFNA5//ADCY3//ADRB1//DRD1//COPS8//G3BP2//MDFIC//GDF11//EDN1//IFNA1//IL2//INHBB//OGN//OSM//P2RY1//PTHLH//CCL2//CCL8//BMP3//TGFB2//TTR//VEGFB//WNT7A//CXCL14//NPNT//ESR1//MTMR9//PPP1R37//PPP1R12A//PPP1R12C//PLEKHF2//URI1//PLEK//SERPINI1//SPOCK1//RECK//PCOLCE2//IQGAP2//FZD10//CPEB2//LRCH1//IPO5//TSC1//SLIT2//GJA1//CDK6//CMKLR1//PIAS4//KLF4//BAMBI//AKTIP//CAPRIN2//PAK6//TGFBR2//SLK//STIM2//WWP2//NDFIP2//PLAA//CRY2//MASTL//CALM2//PPP1R15B//CNN3//PXK//DNAJB4//PFN2//EPHA5//LRP8//RPS6KA4//RPS6KA5//FGD4//FGD5//EPHA4//RAB3GAP1//ARHGAP1//SBF2//FAM13A//CDC42EP3//GNB5//ARAP2//TAGAP//TBC1D20//DENND1B//S1PR1//DOCK9//RGS17//AGFG1//AGFG2//ITGA6//JUN//ARHGAP6//ELMOD1//SOS1//EVI5//TRIP10//ARHGAP12//USP6NL//GIT2//EPHA2//EPHA7//DSTYK//KDR//MET//PRKACB//PRKAR2B//BTAF1//NR4A2//CTSA//NSMAF//FRY//UGT1A8//UGT1A7//UGT1A1//ANP32E//ARL6IP1//FNIP1//BCL2L11//REST//FOXC1//NME1//E2F1//HMGA2//HAND2//RLIM//EGLN1//RNF2//WFS1//FZD6//BHLHE40//HAND1//TMEM64//AIDA//HIPK3//NRBF2//RBL2//PIK3R3//UBXN2B//PPP4R2//LATS2//KAT2B//CCNY//MAPRE3//CCNC//CCND2//MMD//VLDLR//AR//LYPLA1//WASL//POU4F1//NEUROD1//NEUROG1//NHLH2//FOSL1//ARID5B//MTPN//CLOCK//MID1IP1//BAG5//TOPORS//ARRDC3//ARRDC4//PTGES3//RHOC//NOG//RAP2C//EFNA1//ACSL1//TBC1D12//TBC1D17//ASPH//LYPD6//AHNAK//FMR1//UBE2I//MTRR//STAT3//OSR1//CNOT6//DYNLL2//HECW2//GLRX//SOX11//PER2//CHCHD10//ST18//RANBP9//RAPGEF4//DENND5B//RASGEF1A//TRAPPC8//RGL1//GRB2//JAK1//ARHGEF4//RALGPS2//PSD//ARHGEF28//SOS2//DOCK5// |
| GO:0043412 | macromolecule modification | Biological process | 313 | 4389 | 934 | 17653 | 1.34787743155826 | 6.86174495488677e-10 | 6.82440898969106e-08 | 9.16356542832937 | 0.335117773019272 | CDKN1A//GADD45A//PTEN//CCNE2//RANBP9//PPM1L//SPRED1//DUSP5//EREG//RASGEF1A//GRB2//NRG1//IL2//JAK1//MAP3K5//MET//MINK1//TAOK3//MAPK1//CCL2//SOS1//TNF//LAMTOR3//MAP3K2//CRK//CRKL//ADAM9//LPAR1//LPAR3//IGF1//NTF3//PIK3CB//PRKAA1//MAP2K4//DUSP10//DUSP1//DUSP2//PPP2CA//RGS3//TOPORS//WWP2//HECTD2//RNF145//ZNRF2//FBXL5//RNF180//MYLIP//RLIM//ANKIB1//UBE2W//FBXW7//HECW2//SKP1//THOP1//LONRF3//FBXO30//CBFB//UBE3B//LONRF1//COPS8//DRD1//FNIP1//FAM129A//LRP6//PPP1R15B//SLIT2//EFNA1//EFNA5//EPHA7//FMR1//NPTN//KDR//P2RY1//AKTIP//SQSTM1//CCND2//LYPLA1//DUS2//ERRFI1//TSG101//SOCS5//TRIM3//KLHL2//RNF38//MYCBP2//FBXO33//KLHL3//SPOPL//MDM4//DCAF8//DCAF6//FEM1C//KLHL14//MARCH4//RNF4//MARCH7//UBE2Z//RNF128//KLHL15//KBTBD8//ARRDC4//ZFAND4//DNMT3B//MTRR//PPP4R2//SERP1//UEVLD//RIMKLB//UBE2I//HIPK3//ADAM10//CDK6//CDK8//FASTK//PDIK1L//ERG//CDK19//MORC3//MMD//PAN3//DSTYK//LATS2//MKNK2//CDK17//PRKACB//FAM20C//CCL8//TGFB2//TGFBR2//RUNX3//CCNC//RPS6KA4//ACVR1//RPS6KA5//STK17B//PPM1E//FLRT1//GNAQ//NCK1//NF2//ASPN//YWHAG//CAMK2N2//PPM1F//PTPN21//PPP1R12A//CTDSPL2//PPP3R1//PPP6C//PTPN4//KAT2B//CLOCK//NAA30//HS3ST5//PCMTD1//BTG1//BTG2//ASPH//FUT9//ST8SIA5//MAN1A1//MAN2A1//ST8SIA3//B3GNT5//B3GALT2//CCDC126//GFPT2//VEGFB//NTNG1//RECK//PTAR1//PIGA//PTGER4//MDFIC//CALM2//GDF11//INHBB//BMP3//BMPR2//ST3GAL3//RBM47//EPC2//ARID4B//MIER1//ARID4A//RBBP7//TBL1XR1//BRMS1L//ARID5B//USP3//DMWD//ESR1//OTUD3//USP24//AR//USP27X//ATXN3//USP28//USP46//USP45//WDR20//JOSD1//NUP35//EGR2//NUP43//PIAS4//VKORC1L1//CEP41//STK33//MASTL//OSR1//EPHA2//EPHA4//EPHA5//PKDCC//EGLN3//TMX3//ZDHHC1//ST6GALNAC3//ST6GALNAC6//ST6GAL2//ST3GAL5//MCFD2//CRY2//ZDHHC5//BAG6//CRTAP//EGLN1//PAK6//SLK//JARID2//SOX4//PER2//BAG5//NDFIP2//WFS1//PKD2//JUN//RAP2C//PPP1R37//URI1//OSM//PFN2//CAPRIN2//IFNA1//HMG20A//ADCY3//PRKAR2B//ESCO2//PRDM16//DLC1//CNEP1R1//NSMF//PTPDC1//PTPRG//PTP4A1//CDC14A//RNF2//HMGA2//PCGF5//EDEM1//CNOT7//STAT3//TDRD5//EDN1//ITGAV//FZD10//AIDA//TWF1//UBXN2B//TSC1//CKAP4//RAB38//MIA3//ANO8//TADA1//DR1//IPO5//CCNY//MAPRE3//VLDLR//ZMYND11//WNT7A//ULK2//ITGA5//LRP8//ARRDC3//DPY30//KMT2B//MYB//RNF141//PMEPA1//LDLRAD4//NOG//ATG16L1//LMO3//KLF4//NPNT//DNAJC27//HAND2//KDM2A//EZH1//REST//CDC23//UBA5//ACSL1//GLRX//CMTR2//PPT2//PLAA//RAB3GAP1//DERL2//FRY// |
| GO:0048869 | cellular developmental process | Biological process | 309 | 4323 | 934 | 17653 | 1.35096746103334 | 7.35297680656746e-10 | 7.18337366794821e-08 | 9.13353680391751 | 0.330835117773019 | FRY//KLF2//CDH20//NRG1//TGFB2//EREG//YTHDF2//GJC1//FOXF1//ZFPM2//ITGAV//KDR//TMEM100//SOX17//TGFBR2//QKI//EPHA2//FHL2//GJA1//MEF2D//BMP3//CBFB//NOG//TBC1D20//AGFG1//TMF1//DLL1//DSCAML1//STAT3//SEMA4B//ACVR1//DCX//GPM6A//LRP12//NR4A2//SPOCK1//UNK//LATS2//HAND1//FOXF2//PPP3R1//HMGA2//CLIC4//MET//OSR1//NFIB//WNT7A//RUNX3//SULF1//BMPR2//ESR1//RARB//PTHLH//ESCO2//PLEK//RBM47//REST//NKX2-3//FNIP1//SOX4//NME1//EFNA1//ERG//HAND2//GATA6//CDK6//NEUROD1//PALLD//BCL11B//SOX11//AR//FRMD6//DZIP1//RNF2//TDRD5//NTNG1//SLITRK3//NEFH//OGN//POU4F1//SLIT2//RANBP9//ARX//DPYSL2//EFNA5//EPHA5//GAB1//GRB2//KIF5A//OTX2//PIK3CB//ENAH//MAPK1//NTN4//SOS1//VLDLR//NR4A3//KLF7//RPS6KA5//FEZ2//KLF4//ADAM12//MORC3//EGR2//EPHA4//LHX1//P2RY1//DLC1//CDC42EP3//WASF3//PALM2//FGD4//CRK//FGD5//LPAR1//EPS8//BAMBI//RHOC//RND3//MSN//BRWD1//CCL2//WASL//KMT2B//SOSTDC1//ITGA6//FOXC1//TGFB1I1//PPP2CA//PTEN//LDLRAD4//FRZB//PPARG//THRA//BHLHE41//SNAP25//ZNF804A//STMN2//NPTN//NCK1//SERPINI1//TWF1//MYLIP//PTPRG//TSC1//BAG5//DRD1//POU3F2//NF2//LRP6//KBTBD8//RDH10//EDN1//IGF1//GNAQ//FLRT1//STRN//MNX1//MTPN//LHX6//LHX8//HPRT1//C21ORF91//BTG2//PRDM16//TCF7L1//KCNJ10//SH3D19//ZNF268//ELK3//EPHA7//STX2//ETS1//FOXJ3//FLI1//FOXN2//INHBB//JAK1//MYB//MYBL1//NHLH2//ZBTB7A//RBL2//SMOC1//ARHGEF28//ZNF3//BAG6//FXR1//ZIC5//ADAM18//SQSTM1//PKDCC//CEBPB//FZD10//S1PR1//EMX2//MYT1L//HOXD1//NEUROG1//KLF6//IFNA1//DNAJB9//TSG101//ADAM9//IL2//JUN//TMOD3//TMOD1//ACTC1//OSTM1//TNF//RAB21//CNN3//NR5A2//GSTA2//TCF21//KIAA1109//PSD//DSC2//KRT27//PIKFYVE//MAF//ZEB1//PLEKHA1//SYCP3//RAB38//TOPORS//HSBP1//ITGA5//LAMA3//MMP2//FAM20C//PTGER4//IRF1//APCDD1//TMEM64//CFL2//ARID5B//MBNL1//HMGB3//DUSP10//PLEKHB2//TFAP2A//GDF11//E2F1//ZADH2//CMKLR1//CCDC71L//LMO3//BTG1//GRHL1//ERRFI1//SOCS5//NFE2//DPY30//KAT2B//CXCL14//MAP3K5//NTF3//YWHAG//HMG20A//DDX6//DNMT3B//MMD//TOB1//NPNT//TOB2//KLF10//GOLGA4//EN1//FMR1//PBX3//ULK2//LPAR3//RGMA//BNIP3//TNFRSF21//RHEB//MINK1//NSMF//HECW2//ARID4A//JARID2//PER2//MAN2A1//CAPRIN2//TBL1XR1//ADRB1//SLC2A4//CDKN1A//SPRED1//SLC12A5//CTNND2//CPEB3//LRP8//RAP1B//MAP2K4//NEFM//LDLR//SNX2//RAP2C//ARID4B//NANOS1//CRKL//DOCK5//NLN//PLAA//CLOCK//FBXW7// |
| GO:0010558 | negative regulation of macromolecule biosynthetic process | Biological process | 148 | 1709 | 934 | 17653 | 1.63678372340412 | 7.43510508289775e-10 | 7.18337366794821e-08 | 9.12871288906534 | 0.158458244111349 | TSHZ1//HMG20A//KLF2//ZBTB18//CEBPB//ZNF268//TCERG1//WWP2//KLF12//OSR1//CRY2//GLIS3//ARX//DNMT3B//DR1//E2F1//EDN1//EFNA1//CC2D1B//EN1//ESR1//FHL2//CPEB3//FOXF1//FOXC1//MYT1L//CIC//ZFPM2//DNAJB5//GATA6//CNOT7//HSBP1//AR//JARID2//JUN//ZNF568//MAF//MDM4//MYB//NFIA//NFIB//NR4A2//RLIM//KLF3//ZBTB7A//PIAS4//POU4F1//PPARG//CC2D1A//TSHZ3//ZBTB4//RARB//RBBP7//RBBP8//REST//RNF2//PRDM16//SOX17//SOX11//STAT3//ZEB1//TCF21//TFAP2A//TFAP2C//TGIF1//KLF10//TNF//UBE2I//VLDLR//WFS1//ZNF217//LRP8//BTG2//BHLHE41//TBL1XR1//NR4A3//HMGA2//ARID5B//BRMS1L//BHLHE40//RUNX3//URI1//PER2//SQSTM1//NOG//KLF4//HAND1//FNIP1//PHF14//USP3//BAHD1//H2AFZ//DPY30//NF2//SNIP1//ELK3//EREG//FOXF2//DKK3//MDFIC//NRG1//IRF1//LHX1//PAX9//RASD1//ARID4A//ATXN1//THRA//TSG101//BTAF1//RPS6KA5//CLOCK//PPM1F//TOB1//FMR1//NANOS1//CAPRIN2//TSC1//FXR1//ATAD2//MIER1//DCP2//FBXW7//CNOT6//RNF128//ERRFI1//ITM2B//ITGAV//TMEFF2//PAIP2//INHBB//NUP35//NUP43//CNOT6L//YTHDF2//QKI//CPEB2//TOB2//ZMYND11//NPAT//SRSF2//NCK1//DUSP1//GJA1//BMPR2//CPEB1//CPEB4//UNK// |
| GO:0048598 | embryonic morphogenesis | Biological process | 68 | 586 | 934 | 17653 | 2.19322375777419 | 8.31258470172058e-10 | 7.91802962503328e-08 | 9.0802639163893 | 0.0728051391862955 | LHX1//LRP6//RNF2//ACVR1//DUSP1//DUSP2//DUSP5//SOX17//NOG//NF2//BMPR2//NR4A3//HAND1//LATS2//SOX4//SOX11//DLC1//PRKACB//TGFB2//TSC1//FZD6//GJA1//DLL1//PKD2//TGFBR2//HAND2//SOS1//TFAP2A//GATA6//KLF4//EFNA1//TGFB1I1//MBNL1//LRIG1//OSR1//RDH10//EN1//WNT7A//RECK//RARB//HSBP1//ITGA5//ITGAV//LAMA3//MMP2//HMGA2//NEUROG1//SOBP//MAPK1//TSHZ1//EDN1//GNAQ//FOXC1//FOXF1//TCF21//NEUROD1//FOXF2//ZEB1//HOXA3//DSCAML1//AR//ZNF568//GRB2//SULF1//OTX2//FRZB//BCL2L11//YTHDF2// |
| GO:0031324 | negative regulation of cellular metabolic process | Biological process | 214 | 2748 | 934 | 17653 | 1.47186741223518 | 9.25467590193935e-10 | 8.64228764648389e-08 | 9.03363878560518 | 0.229122055674518 | TSHZ1//HMG20A//KLF2//ZBTB18//CEBPB//ZNF268//TCERG1//WWP2//KLF12//OSR1//CRY2//GLIS3//ARX//DNMT3B//DR1//E2F1//EDN1//EFNA1//CC2D1B//EN1//ESR1//FHL2//CPEB3//FOXF1//FOXC1//MYT1L//CIC//ZFPM2//DNAJB5//GATA6//CNOT7//HSBP1//AR//JARID2//JUN//ZNF568//MAF//MDM4//MYB//NFIA//NFIB//NR4A2//RLIM//KLF3//ZBTB7A//PIAS4//POU4F1//PPARG//CC2D1A//TSHZ3//ZBTB4//RARB//RBBP7//RBBP8//REST//RNF2//PRDM16//SOX17//SOX11//STAT3//ZEB1//TCF21//TFAP2A//TFAP2C//TGIF1//KLF10//TNF//UBE2I//VLDLR//WFS1//ZNF217//LRP8//BTG2//BHLHE41//TBL1XR1//NR4A3//HMGA2//ARID5B//BRMS1L//BHLHE40//RUNX3//URI1//PER2//SQSTM1//NOG//KLF4//HAND1//FNIP1//PHF14//USP3//DUSP10//SPRED1//DUSP1//DUSP2//DUSP5//PPP2CA//RGS3//FAM129A//IL2//LRP6//PTEN//PPP1R15B//SLIT2//ERRFI1//TSG101//SOCS5//BAHD1//H2AFZ//DPY30//GADD45A//PPM1E//FLRT1//GNAQ//NCK1//NF2//TAOK3//ASPN//DUS2//YWHAG//CAMK2N2//PPM1F//SNIP1//MET//MTMR9//BNIP3//TGFB2//CALM2//FBXW7//PLEK//PPP1R37//PPP1R12A//PPP1R12C//PLEKHF2//SERPINI1//SPOCK1//RECK//TSC1//ELK3//EREG//FOXF2//DKK3//MDFIC//NRG1//IRF1//LHX1//PAX9//RASD1//ARID4A//ATXN1//THRA//BTAF1//RPS6KA5//CLOCK//TOB1//FMR1//NANOS1//CAPRIN2//FXR1//EGLN1//SPOPL//SOX4//BAG5//CDK6//ATAD2//MIER1//DCP2//BAG6//MASTL//RNF4//CNOT6//RNF128//CDKN1A//ITM2B//ARL6IP1//HIPK3//AIDA//ITGAV//LATS2//IPO5//KAT2B//UGT1A8//PAIP2//PRKAA1//ZMYND11//INHBB//NTF3//PMEPA1//LDLRAD4//NUP35//NUP43//CNOT6L//YTHDF2//QKI//RANBP9//LMO3//PLAA//ORMDL3//CPEB2//CRTAP//FEZ2//EPHA4//TOB2//NPAT//SRSF2//DERL2//FRY//CTSA//KLHL15//GJA1//BMPR2//PRKAR2B//CPEB1//CPEB4//UNK// |
| GO:0009887 | animal organ morphogenesis | Biological process | 99 | 1001 | 934 | 17653 | 1.86927312516178 | 9.32850803183978e-10 | 8.64228764648389e-08 | 9.03018781020335 | 0.105995717344754 | THRA//WNT7A//NPNT//LHX1//PKD2//TCF21//STAT3//GJA1//DLL1//SOX17//TGFBR2//HAND1//HAND2//FOXC1//MEF2D//SOX11//DLC1//TGFB2//ACVR1//ZFPM2//GATA6//RARB//BMPR2//NOG//JUN//TMEM100//EFNA1//ERG//SOS1//SOX4//FOXF1//NRG1//POU4F1//S1PR1//TFAP2A//HOXA3//TOPORS//FJX1//NKX2-3//MSN//LRIG1//OSR1//NEUROG1//LRP6//SOBP//FZD6//MAPK1//TSHZ1//EDN1//BCL2L11//SOSTDC1//LHX8//NF2//BCL11B//COL1A2//PAX9//ERRFI1//NEUROD1//GDF11//FOXF2//ZEB1//SERP1//ARID5B//AR//RDH10//DSCAML1//PLEKHA1//NR4A3//PTEN//EGLN1//ACTC1//MAN2A1//VANGL1//MMP2//FHL2//EPHA2//ESR1//NFIB//TNF//NTN4//SULF1//BTBD7//NR5A2//FAM20C//ASPN//TBC1D20//KLHL3//CEBPB//FRZB//RAB23//ITGA6//FBXW7//CRKL//E2F5//STX2//EREG//FLI1//CCL2//BHLHE41// |
| GO:0051247 | positive regulation of protein metabolic process | Biological process | 143 | 1641 | 934 | 17653 | 1.64702086652652 | 1.00270730438884e-09 | 9.16393175619152e-08 | 8.99882582135638 | 0.153104925053533 | GADD45A//MINK1//TNF//MAP3K2//CRK//CRKL//MAP3K5//TAOK3//LAMTOR3//ADAM9//DUSP5//LPAR1//LPAR3//NRG1//IGF1//NTF3//PIK3CB//PRKAA1//MAPK1//MAP2K4//FAM129A//EFNA1//EFNA5//EPHA7//FMR1//NPTN//KDR//P2RY1//AKTIP//SQSTM1//CCND2//FNIP1//EREG//FBXW7//DLC1//EGLN3//F3//PMAIP1//PPARG//COPS8//MDFIC//CALM2//GDF11//INHBB//BMP3//BMPR2//TGFB2//ACVR1//PCOLCE2//CPEB3//SERP1//PRR16//SOX4//FXR1//NDFIP2//WFS1//PKD2//RAP2C//PAK6//TGFBR2//SLK//WNT7A//KLF4//RNF180//ANKIB1//LONRF3//LONRF1//SOCS5//PPP1R15B//RPS6KA4//RPS6KA5//OSM//PFN2//CAPRIN2//IFNA1//PIAS4//ADCY3//PRKACB//PRKAR2B//CNEP1R1//NSMF//PPP1R12A//NCK1//IL2//STAT3//BCL2L11//REST//PPM1F//EDN1//EPHA2//EPHA4//EPHA5//AR//ITGAV//MET//FZD10//IRF1//WWP2//GJA1//MYLIP//NSF//HECW2//SOX17//CCNY//MAPRE3//CCNC//CDKN1A//MMD//VLDLR//ASPH//ITGA5//LRP8//SH3D19//TOPORS//ARRDC3//ARRDC4//DNMT3B//MYB//JARID2//NPNT//DSTYK//JUN//DNAJC27//PTEN//CCL2//CCL8//HAND2//ACSL1//PPP2CA//RAB3GAP1//FBXL5//MSN//YTHDF2//UBE2I//ESR1//EDEM1//ATXN3//BAG6//RNF128//LDLR//ST18//KLF2//MTPN// |
| GO:0051253 | negative regulation of RNA metabolic process | Biological process | 120 | 1306 | 934 | 17653 | 1.73663965686291 | 1.25638983122235e-09 | 1.13292859047423e-07 | 8.90087558728618 | 0.12847965738758 | TSHZ1//HMG20A//KLF2//ZBTB18//CEBPB//ZNF268//TCERG1//WWP2//KLF12//OSR1//CRY2//GLIS3//ARX//DNMT3B//DR1//E2F1//EDN1//EFNA1//CC2D1B//EN1//ESR1//FHL2//CPEB3//FOXF1//FOXC1//MYT1L//CIC//ZFPM2//DNAJB5//GATA6//CNOT7//HSBP1//AR//JARID2//JUN//ZNF568//MAF//MDM4//MYB//NFIA//NFIB//NR4A2//RLIM//KLF3//ZBTB7A//PIAS4//POU4F1//PPARG//CC2D1A//TSHZ3//ZBTB4//RARB//RBBP7//RBBP8//REST//RNF2//PRDM16//SOX17//SOX11//STAT3//ZEB1//TCF21//TFAP2A//TFAP2C//TGIF1//KLF10//TNF//UBE2I//VLDLR//WFS1//ZNF217//LRP8//BTG2//BHLHE41//TBL1XR1//NR4A3//HMGA2//ARID5B//BRMS1L//BHLHE40//RUNX3//URI1//PER2//SQSTM1//NOG//KLF4//HAND1//FNIP1//PHF14//USP3//BAHD1//H2AFZ//DPY30//ELK3//EREG//FOXF2//DKK3//MDFIC//NRG1//IRF1//LHX1//PAX9//RASD1//ARID4A//ATXN1//THRA//TSG101//BTAF1//RPS6KA5//CLOCK//PPM1F//ATAD2//MIER1//TOB1//FMR1//TOB2//ZMYND11//NPAT//SRSF2//NCK1// |
| GO:0034654 | nucleobase-containing compound biosynthetic process | Biological process | 317 | 4488 | 934 | 17653 | 1.33499014264067 | 1.51209334748789e-09 | 1.34556411961324e-07 | 8.82042139729545 | 0.339400428265525 | E2F1//NPAT//RRM2//TSHZ1//HMG20A//KLF2//ZBTB18//CEBPB//ZNF268//TCERG1//WWP2//KLF12//OSR1//CRY2//GLIS3//ARX//DNMT3B//DR1//EDN1//EFNA1//CC2D1B//EN1//ESR1//FHL2//CPEB3//FOXF1//FOXC1//MYT1L//CIC//ZFPM2//DNAJB5//GATA6//CNOT7//HSBP1//AR//JARID2//JUN//ZNF568//MAF//MDM4//MYB//NFIA//NFIB//NR4A2//RLIM//KLF3//ZBTB7A//PIAS4//POU4F1//PPARG//CC2D1A//TSHZ3//ZBTB4//RARB//RBBP7//RBBP8//REST//RNF2//PRDM16//SOX17//SOX11//STAT3//ZEB1//TCF21//TFAP2A//TFAP2C//TGIF1//KLF10//TNF//UBE2I//VLDLR//WFS1//ZNF217//LRP8//BTG2//BHLHE41//TBL1XR1//NR4A3//HMGA2//ARID5B//BRMS1L//BHLHE40//RUNX3//URI1//PER2//SQSTM1//NOG//KLF4//HAND1//FNIP1//PHF14//USP3//GTF2E1//IRF9//KLF6//CREBRF//EGR2//ELK3//ERG//FOXJ3//FOXF2//FLI1//GRHL1//HIVEP2//IRF1//LHX1//MEF2D//MEOX2//MYBL1//NEUROD1//NHLH2//OTX2//PAX9//PBX3//POU3F2//RAD21//ARID4A//SHOX//BCL11B//BMPR2//SOX4//BTF3//TEF//TGFB1I1//THRA//TMF1//DEK//FOSL1//KLF7//CBFB//FUBP3//HAND2//CLOCK//POLR3G//SRSF2//PKD2//NAGK//GFPT2//SLC35D1//UGP2//NUP35//NUP43//HPRT1//ADCY3//IMPDH1//NME1//PDE10A//CMPK1//DCTPP1//BAHD1//H2AFZ//KMT2B//DPY30//TOPORS//KHDRBS3//ZMYND11//TADA1//ZNF597//CTNND2//ZNF782//NKX2-3//ZNF800//E2F5//ZNF367//EZH1//ZBTB9//KDM2A//MYCBP2//FLII//CNOT6L//EPC2//LHX6//HBP1//ATAD2//SERTAD3//MDFIC//MNX1//HMGB3//HOXA3//HOXD1//FOXN2//ZBTB41//ZBTB34//ATXN3//LHX8//NEUROG1//NFE2//NPAS2//IER5//ARID4B//BRWD1//BNC2//ZNF532//CDCA7L//PRKAA1//TRERF1//LMO3//MAPK1//SLC2A4RG//PHTF2//ZNF630//ZNF287//CNOT6//ZNF512B//ZBTB26//MIER1//RBL2//RNF4//ATXN1//DMRTA1//SMARCD2//STAT4//ZNF2//ZNF3//ZNF131//ZNF655//BRD3//TCF7L1//PCGF5//LCOR//WASL//ZBTB47//ST18//HIPK3//ELAVL2//NR5A2//NRBF2//MLLT6//RNF141//PPP2CA//PAK6//SNAPC1//BTG1//SNIP1//RPS6KA4//RPS6KA5//CDKN1A//CRK//EMX2//ETS1//BOLA3//NR3C2//PLEK//ZNFX1//NKX2-4//RFX7//BDP1//CDK8//E2F3//TEAD1//KAT2B//CCNC//CHCHD10//PTGES3//G3BP2//EREG//ACOT7//PPCS//MAP3K2//MAPRE3//BAMBI//RGMB//IGF1//LRP6//MAP3K5//WNT7A//ACVR1//DKK3//NRG1//RASD1//TSG101//BTAF1//PPM1F//TOB1//SOSTDC1//TMEM100//RGMA//BMP3//TGFB2//SULF1//CTDSPL2//ADRB1//PTHLH//CALM2//EGLN1//CMKLR1//DCP2//CCT6A//EPHA5//UXS1//REV3L//INTS8//NABP1//FZD6//NPLOC4//S1PR1//DLL1//IL2//ITGA6//MET//PPP1R12A//NCK1//OSM//P2RY1//PPP3R1//DCAF6//CAPRIN2//PTEN//MTPN//EGLN3//PFKP//RAP2C//TOB2//DUSP1//GJA1//FBXW7//NPNT// |
| GO:0009790 | embryo development | Biological process | 95 | 959 | 934 | 17653 | 1.8723051983575 | 1.93616660164735e-09 | 1.70055775674559e-07 | 8.71305727561074 | 0.101713062098501 | WNT7A//BCL2L11//ADAM10//KLF2//RDH10//EDN1//FOXF1//FOXC1//ZFPM2//GATA6//GJA1//EPN1//AR//ZNF568//MAN2A1//MBNL1//TMEM100//BTF3//TGFBR2//FOSL1//ACVR1//NOG//HAND2//LHX1//LRP6//RNF2//DUSP1//DUSP2//DUSP5//SOX17//NF2//BMPR2//NR4A3//HAND1//DLL1//MEOX2//LATS2//RBBP8//SOX4//SOX11//DLC1//PRKACB//TGFB2//TSC1//FZD6//CEBPB//PKD2//SOS1//TFAP2A//NEUROG1//KLF4//RAI2//NR5A2//EFNA1//KBTBD8//TGFB1I1//TDRD5//EN1//LRIG1//OSR1//RECK//RARB//KDR//HSBP1//ITGA5//ITGAV//LAMA3//MMP2//HMGA2//PIK3CB//OTX2//SOBP//MAPK1//TSHZ1//GNAQ//TCF21//NEUROD1//FOXF2//TNF//PKDCC//TEAD1//ZEB1//YTHDF2//HOXA3//DSCAML1//SLC35D1//SULF1//HOXD1//GRB2//EGLN1//VASH2//FRZB//IGF1//NRG1//BAG6// |
| GO:0007417 | central nervous system development | Biological process | 93 | 932 | 934 | 17653 | 1.88598694065857 | 2.01214807069469e-09 | 1.74463556437284e-07 | 8.69634006341756 | 0.0995717344753747 | BCL2L11//DPYSL2//S1PR1//EGR2//EPHA7//RAB3GAP1//CIC//SPHK2//SLC17A7//DSCAML1//PTPRG//RARB//ATXN1//BMPR2//TGFBR2//THRA//BAG6//IMMP2L//B3GNT5//CDK6//DRD1//DLL1//POU3F2//WASF3//LPAR1//SOX11//PKD2//SOX4//NOG//GDF11//PBX3//LHX1//VLDLR//MNX1//POU4F1//LRP8//EMX2//NEUROD1//PTEN//BTG2//EN1//FOXC1//DLC1//LRP6//TFAP2A//MTPN//WNT7A//NFIB//HPRT1//ARX//CALM2//SLC32A1//EPHA5//EZH1//NF2//NME1//TSC1//NR4A3//BCL11B//LHX6//SLIT2//NRG1//ETS1//GNAQ//LHX8//C21ORF91//NR4A2//SPOCK1//EPHA4//TACC2//BNIP3//KCNJ10//E2F1//OTX2//FZD6//STAT3//DUSP10//TNFRSF21//PPARG//RHEB//MAPK1//LDLR//NANOS1//SOS1//HAPLN1//DCX//FMR1//JARID2//NHLH2//NPAS2//SERPINI1//ZEB1//FXR1// |
| GO:0032989 | cellular component morphogenesis | Biological process | 105 | 1101 | 934 | 17653 | 1.8024931588375 | 2.09313944813526e-09 | 1.7918863402201e-07 | 8.67920183733821 | 0.112419700214133 | FRY//KLF2//CDH20//NRG1//TGFB2//TBC1D20//AGFG1//TMF1//LATS2//SOX17//CLIC4//MET//PALLD//AR//BCL11B//FRMD6//NTNG1//SLITRK3//NEFH//OGN//POU4F1//DSCAML1//WNT7A//SLIT2//RANBP9//ARX//DPYSL2//EFNA1//EFNA5//EPHA5//GAB1//GRB2//KIF5A//OTX2//PIK3CB//ENAH//MAPK1//NTN4//SOS1//VLDLR//NR4A3//KLF7//RPS6KA5//FEZ2//EGR2//EPHA4//LHX1//NOG//DLC1//CDC42EP3//WASF3//PALM2//FGD4//CRK//FGD5//LPAR1//EPS8//BAMBI//KDR//RHOC//RND3//MSN//BRWD1//CCL2//WASL//NR4A2//PTEN//NFIB//SH3D19//TMOD3//TMOD1//ACTC1//RAB21//EPHA7//PIKFYVE//ITGAV//CFL2//GOLGA4//UNK//ULK2//LPAR3//GJA1//GPM6A//MNX1//NTF3//MINK1//HPRT1//FMR1//NSMF//HECW2//FXR1//BMPR2//SEMA4B//POU3F2//CAPRIN2//EDN1//CTNND2//LRP8//EPHA2//SNX2//YTHDF2//CRKL//DOCK5//PLAA//FLRT1// |
| GO:0030154 | cell differentiation | Biological process | 295 | 4124 | 934 | 17653 | 1.35199474741265 | 2.149195539206e-09 | 1.81687617895627e-07 | 8.66772406950862 | 0.315845824411135 | EREG//YTHDF2//GJC1//FOXF1//ZFPM2//ITGAV//KDR//TMEM100//SOX17//TGFBR2//QKI//EPHA2//FHL2//GJA1//MEF2D//BMP3//CBFB//NOG//TBC1D20//AGFG1//TMF1//DLL1//DSCAML1//STAT3//SEMA4B//ACVR1//DCX//GPM6A//LRP12//NR4A2//SPOCK1//UNK//LATS2//HAND1//FOXF2//PPP3R1//TGFB2//HMGA2//CLIC4//MET//OSR1//NFIB//WNT7A//RUNX3//SULF1//BMPR2//ESR1//RARB//PTHLH//ESCO2//PLEK//RBM47//REST//NKX2-3//FNIP1//SOX4//NME1//EFNA1//ERG//HAND2//GATA6//CDK6//NEUROD1//PALLD//BCL11B//SOX11//AR//FRMD6//DZIP1//RNF2//TDRD5//NTNG1//SLITRK3//NEFH//OGN//POU4F1//SLIT2//RANBP9//ARX//DPYSL2//EFNA5//EPHA5//GAB1//GRB2//KIF5A//OTX2//PIK3CB//ENAH//MAPK1//NTN4//SOS1//VLDLR//NR4A3//KLF7//RPS6KA5//FEZ2//KLF4//ADAM12//EGR2//EPHA4//LHX1//P2RY1//KMT2B//SOSTDC1//ITGA6//FOXC1//BAMBI//TGFB1I1//PPP2CA//PTEN//LDLRAD4//FRZB//PPARG//THRA//BHLHE41//SNAP25//ZNF804A//STMN2//NPTN//NCK1//SERPINI1//TWF1//LPAR1//MYLIP//PTPRG//TSC1//BAG5//DRD1//POU3F2//WASF3//NF2//LRP6//KBTBD8//RDH10//EDN1//NRG1//IGF1//GNAQ//FLRT1//STRN//MNX1//MTPN//LHX6//LHX8//HPRT1//C21ORF91//BTG2//PRDM16//TCF7L1//KCNJ10//CEBPB//FZD10//S1PR1//EMX2//MYT1L//HOXD1//NEUROG1//KLF6//IFNA1//DNAJB9//TSG101//ADAM9//IL2//JUN//TMOD3//TMOD1//ACTC1//OSTM1//TNF//RAB21//CNN3//NR5A2//GSTA2//TCF21//KIAA1109//FRY//PSD//EPHA7//DSC2//KRT27//MAF//PKDCC//ZEB1//PLEKHA1//SYCP3//RAB38//TOPORS//FLI1//FOXN2//HSBP1//ITGA5//LAMA3//MMP2//FAM20C//PTGER4//KLF2//IRF1//APCDD1//CCL2//TMEM64//MSN//CFL2//INHBB//ARID5B//MBNL1//HMGB3//DUSP10//PLEKHB2//TFAP2A//GDF11//ZNF268//E2F1//ZADH2//CMKLR1//CCDC71L//LMO3//BTG1//GRHL1//ERRFI1//MYB//SOCS5//ETS1//NFE2//DPY30//KAT2B//CXCL14//MAP3K5//NTF3//YWHAG//HMG20A//DDX6//DNMT3B//MMD//SMOC1//TOB1//NPNT//TOB2//KLF10//GOLGA4//EN1//FMR1//PBX3//FXR1//ULK2//LPAR3//RGMA//BNIP3//TNFRSF21//RHEB//MINK1//NSMF//HECW2//ARID4A//JARID2//PER2//MAN2A1//CAPRIN2//TBL1XR1//ADRB1//SLC2A4//CDKN1A//SPRED1//SLC12A5//CTNND2//WASL//CPEB3//LRP8//RAP1B//MAP2K4//NEFM//LDLR//RAP2C//ARID4B//NANOS1//CRK//CRKL//DOCK5//NLN//PLAA//CLOCK//FBXW7//ELK3//STX2//FOXJ3//JAK1//MYBL1//NHLH2//ZBTB7A//RBL2//ARHGEF28//ZNF3//BAG6//ZIC5//ADAM18//SQSTM1// |
| GO:0048666 | neuron development | Biological process | 102 | 1060 | 934 | 17653 | 1.81872045573916 | 2.25713252940339e-09 | 1.88456633288335e-07 | 8.64644294021386 | 0.109207708779443 | NTNG1//SLITRK3//NEFH//OGN//POU4F1//DSCAML1//WNT7A//SLIT2//RANBP9//ARX//DPYSL2//EFNA1//EFNA5//EPHA5//GAB1//GRB2//KIF5A//OTX2//PIK3CB//ENAH//MAPK1//NTN4//SOS1//VLDLR//NR4A3//KLF7//RPS6KA5//FEZ2//EGR2//EPHA4//LHX1//NOG//PTEN//SNAP25//ZNF804A//STMN2//NPTN//NCK1//NME1//SERPINI1//TWF1//LPAR1//MYLIP//PTPRG//SPOCK1//TSC1//BAG5//GNAQ//FLRT1//STRN//DRD1//LHX6//LHX8//NR4A2//HPRT1//BTG2//NFIB//RAB21//JUN//FRY//GPM6A//LRP12//PSD//EPHA7//TOPORS//GOLGA4//NRG1//UNK//ULK2//LPAR3//KLF4//RGMA//GJA1//MNX1//NTF3//MINK1//FMR1//NSMF//HECW2//FXR1//BMPR2//SEMA4B//RUNX3//HAND2//POU3F2//CAPRIN2//SLC12A5//CTNND2//WASL//CPEB3//IL2//C21ORF91//LRP8//NEFM//BCL11B//NANOS1//PLAA//EN1//MYT1L//NEUROD1//PBX3//TGFB2// |
| GO:0042325 | regulation of phosphorylation | Biological process | 135 | 1544 | 934 | 17653 | 1.65256335223175 | 2.59532812249015e-09 | 2.14051269419523e-07 | 8.58580772730363 | 0.144539614561028 | CDKN1A//GADD45A//PTEN//CCNE2//MINK1//TNF//MAP3K2//CRK//CRKL//MAP3K5//TAOK3//LAMTOR3//ADAM9//DUSP5//LPAR1//LPAR3//NRG1//IGF1//NTF3//PIK3CB//PRKAA1//MAPK1//MAP2K4//DUSP10//SPRED1//DUSP1//DUSP2//PPP2CA//RGS3//DRD1//FNIP1//FAM129A//IL2//LRP6//PPP1R15B//SLIT2//EFNA1//EFNA5//EPHA7//FMR1//NPTN//KDR//P2RY1//AKTIP//SQSTM1//CCND2//EREG//FBXW7//ERRFI1//TSG101//SOCS5//NUP35//NUP43//PPM1E//FLRT1//GNAQ//NCK1//NF2//ASPN//DUS2//YWHAG//CAMK2N2//PPM1F//COPS8//MDFIC//CALM2//GDF11//INHBB//BMP3//BMPR2//TGFB2//ACVR1//CDK6//PKD2//JUN//RAP2C//PAK6//TGFBR2//SLK//RPS6KA4//RPS6KA5//OSM//PFN2//CAPRIN2//IFNA1//EPHA2//EPHA4//EPHA5//DSTYK//MET//ADCY3//PRKACB//PRKAR2B//PTGES3//KCTD20//ITGA6//AR//BTBD10//CNOT7//STAT3//EDN1//GRB2//ITGAV//FZD10//HIPK3//AIDA//TWF1//NRBF2//RBL2//PIK3R3//KLF4//LATS2//IPO5//KAT2B//CCNY//MAPRE3//CCNC//TSC1//MMD//VLDLR//ZMYND11//WNT7A//ITGA5//LRP8//PMEPA1//LDLRAD4//NOG//RANBP9//LMO3//NPNT//DNAJC27//CCL2//CCL8//HAND2//ACSL1// |
| GO:0000902 | cell morphogenesis | Biological process | 97 | 997 | 934 | 17653 | 1.8388581161042 | 3.29685335970434e-09 | 2.6863396712868e-07 | 8.48190036935291 | 0.103854389721627 | LATS2//SOX17//CLIC4//MET//PALLD//AR//BCL11B//FRMD6//NTNG1//SLITRK3//NEFH//OGN//POU4F1//DSCAML1//WNT7A//SLIT2//RANBP9//ARX//DPYSL2//EFNA1//EFNA5//EPHA5//GAB1//GRB2//KIF5A//OTX2//PIK3CB//ENAH//MAPK1//NTN4//SOS1//VLDLR//NR4A3//KLF7//RPS6KA5//FEZ2//EGR2//EPHA4//LHX1//NOG//DLC1//CDC42EP3//WASF3//PALM2//FGD4//CRK//FGD5//LPAR1//EPS8//BAMBI//KDR//RHOC//RND3//MSN//BRWD1//CCL2//WASL//NR4A2//PTEN//NFIB//SH3D19//RAB21//EPHA7//ITGAV//GOLGA4//NRG1//UNK//ULK2//LPAR3//GJA1//GPM6A//MNX1//NTF3//MINK1//HPRT1//FMR1//NSMF//HECW2//FXR1//BMPR2//SEMA4B//POU3F2//CAPRIN2//CTNND2//LRP8//TBC1D20//EPHA2//SNX2//YTHDF2//CRKL//DOCK5//PLAA//FLRT1//FRY//KLF2//CDH20//TGFB2// |
| GO:0050790 | regulation of catalytic activity | Biological process | 183 | 2292 | 934 | 17653 | 1.5090656075877 | 3.42119301539907e-09 | 2.75446766227904e-07 | 8.46582242328408 | 0.19593147751606 | CDKN1A//GADD45A//PTEN//CCNE2//MINK1//TNF//MAP3K2//CRK//CRKL//MAP3K5//TAOK3//LAMTOR3//ADAM9//DUSP5//LPAR1//LPAR3//NRG1//IGF1//NTF3//PIK3CB//PRKAA1//MAPK1//MAP2K4//DUSP10//SPRED1//DUSP1//DUSP2//PPP2CA//RGS3//EREG//FBXW7//ERRFI1//TSG101//SOCS5//PPM1E//FLRT1//GNAQ//LRP6//NCK1//NF2//ASPN//DUS2//YWHAG//CAMK2N2//PPM1F//DLC1//EGLN3//F3//PMAIP1//PPARG//EFNA5//ADCY3//ADRB1//DRD1//COPS8//MDFIC//TGFB2//NPNT//ESR1//MTMR9//PPP1R37//PPP1R12A//PPP1R12C//PLEKHF2//URI1//PLEK//SERPINI1//SPOCK1//RECK//PCOLCE2//IQGAP2//FZD10//CPEB2//LRCH1//IPO5//TSC1//SLIT2//CDK6//PKD2//PAK6//TGFBR2//SLK//PLAA//CRY2//MASTL//CALM2//PPP1R15B//CNN3//PXK//DNAJB4//PFN2//FGD4//FGD5//EPHA4//EPHA5//RAB3GAP1//ARHGAP1//SBF2//FAM13A//CDC42EP3//GNB5//ARAP2//TAGAP//TBC1D20//DENND1B//S1PR1//DOCK9//RGS17//AGFG1//AGFG2//ITGA6//JUN//ARHGAP6//ELMOD1//CCL2//CCL8//SOS1//EVI5//TRIP10//ARHGAP12//USP6NL//GIT2//EPHA2//EPHA7//DSTYK//KDR//MET//PRKACB//PRKAR2B//NR4A2//CTSA//NSMAF//FRY//UGT1A8//UGT1A7//UGT1A1//ANP32E//ARL6IP1//KLF4//FNIP1//BCL2L11//REST//EDN1//TMEM64//HIPK3//AIDA//NRBF2//RBL2//PIK3R3//UBXN2B//PPP4R2//LATS2//KAT2B//CCNY//MAPRE3//CCNC//CCND2//MMD//VLDLR//LYPLA1//WASL//EGLN1//MID1IP1//BAG5//TOPORS//ARRDC3//ARRDC4//PTGES3//RHOC//RAP2C//EFNA1//LRP8//ACSL1//TBC1D12//TBC1D17//ASPH//UBE2I//MTRR//STAT3//DYNLL2//CHCHD10//ST18//AR// |
| GO:0009890 | negative regulation of biosynthetic process | Biological process | 152 | 1809 | 934 | 17653 | 1.58809568621324 | 3.47049771069197e-09 | 2.76129129616586e-07 | 8.45960823774316 | 0.162740899357602 | TSHZ1//HMG20A//KLF2//ZBTB18//CEBPB//ZNF268//TCERG1//WWP2//KLF12//OSR1//CRY2//GLIS3//ARX//DNMT3B//DR1//E2F1//EDN1//EFNA1//CC2D1B//EN1//ESR1//FHL2//CPEB3//FOXF1//FOXC1//MYT1L//CIC//ZFPM2//DNAJB5//GATA6//CNOT7//HSBP1//AR//JARID2//JUN//ZNF568//MAF//MDM4//MYB//NFIA//NFIB//NR4A2//RLIM//KLF3//ZBTB7A//PIAS4//POU4F1//PPARG//CC2D1A//TSHZ3//ZBTB4//RARB//RBBP7//RBBP8//REST//RNF2//PRDM16//SOX17//SOX11//STAT3//ZEB1//TCF21//TFAP2A//TFAP2C//TGIF1//KLF10//TNF//UBE2I//VLDLR//WFS1//ZNF217//LRP8//BTG2//BHLHE41//TBL1XR1//NR4A3//HMGA2//ARID5B//BRMS1L//BHLHE40//RUNX3//URI1//PER2//SQSTM1//NOG//KLF4//HAND1//FNIP1//PHF14//USP3//BAHD1//H2AFZ//DPY30//NF2//SNIP1//FBXW7//PLEK//ELK3//EREG//FOXF2//DKK3//MDFIC//NRG1//IRF1//LHX1//PAX9//RASD1//ARID4A//ATXN1//THRA//TSG101//BTAF1//RPS6KA5//CLOCK//PPM1F//TOB1//FMR1//NANOS1//CAPRIN2//TSC1//FXR1//EGLN1//ATAD2//MIER1//DCP2//ERRFI1//CNOT6//RNF128//ITM2B//ITGAV//TMEFF2//PAIP2//PRKAA1//INHBB//NUP35//NUP43//CNOT6L//YTHDF2//QKI//ORMDL3//CPEB2//TOB2//ZMYND11//NPAT//SRSF2//NCK1//DUSP1//GJA1//BMPR2//CPEB1//CPEB4//UNK// |
| GO:0044237 | cellular metabolic process | Biological process | 662 | 10957 | 934 | 17653 | 1.14192603009741 | 4.1270222111652e-09 | 3.22009269252392e-07 | 8.38436319379268 | 0.708779443254818 | ACOX1//UBXN2B//TRAPPC8//RAB23//ATG16L1//ULK2//IGF1//NFIA//NFIB//RBBP7//RBBP8//RRM2//CDKN1A//GADD45A//PTEN//CCNE2//E2F1//NPAT//MTRR//TSHZ1//HMG20A//KLF2//ZBTB18//CEBPB//ZNF268//TCERG1//WWP2//KLF12//OSR1//CRY2//GLIS3//ARX//DNMT3B//DR1//EDN1//EFNA1//CC2D1B//EN1//ESR1//FHL2//CPEB3//FOXF1//FOXC1//MYT1L//CIC//ZFPM2//DNAJB5//GATA6//CNOT7//HSBP1//AR//JARID2//JUN//ZNF568//MAF//MDM4//MYB//NR4A2//RLIM//KLF3//ZBTB7A//PIAS4//POU4F1//PPARG//CC2D1A//TSHZ3//ZBTB4//RARB//REST//RNF2//PRDM16//SOX17//SOX11//STAT3//ZEB1//TCF21//TFAP2A//TFAP2C//TGIF1//KLF10//TNF//UBE2I//VLDLR//WFS1//ZNF217//LRP8//BTG2//BHLHE41//TBL1XR1//NR4A3//HMGA2//ARID5B//BRMS1L//BHLHE40//RUNX3//URI1//PER2//SQSTM1//NOG//KLF4//HAND1//FNIP1//PHF14//USP3//RANBP9//PPM1L//SPRED1//DUSP5//EREG//RASGEF1A//GRB2//NRG1//IL2//JAK1//MAP3K5//MET//MINK1//TAOK3//MAPK1//CCL2//SOS1//LAMTOR3//DCP2//PPP2CA//RPL15//MAP3K2//CRK//CRKL//ADAM9//LPAR1//LPAR3//NTF3//PIK3CB//PRKAA1//MAP2K4//DUSP10//DUSP1//DUSP2//RGS3//TOPORS//HECTD2//RNF145//ZNRF2//FBXL5//RNF180//MYLIP//ANKIB1//UBE2W//FBXW7//HECW2//SKP1//THOP1//LONRF3//FBXO30//CBFB//UBE3B//LONRF1//ZFAND4//CNOT6L//PAN3//CNOT6//COPS8//RBFOX3//FMR1//MBNL1//RBM25//SRSF2//ELAVL2//NOVA1//BNIP3//PTGES3//NABP1//GTF2E1//SECISBP2L//RDH10//NAPEPLD//TTR//CHST7//CHST1//ITGB8//ST6GALNAC3//ST6GALNAC6//ST8SIA3//ST3GAL5//ACSL1//CAMLG//EPS15//NSF//DRD1//FAM129A//LRP6//PPP1R15B//SLIT2//EFNA5//EPHA7//NPTN//KDR//P2RY1//AKTIP//CCND2//LYPLA1//DUS2//IRF9//KLF6//CREBRF//EGR2//ELK3//ERG//FOXJ3//FOXF2//FLI1//GRHL1//HIVEP2//IRF1//LHX1//MEF2D//MEOX2//MYBL1//NEUROD1//NHLH2//OTX2//PAX9//PBX3//POU3F2//RAD21//ARID4A//SHOX//BCL11B//BMPR2//SOX4//BTF3//TEF//TGFB1I1//THRA//TMF1//DEK//FOSL1//KLF7//FUBP3//HAND2//CLOCK//POLR3G//PKD2//ERRFI1//TSG101//SOCS5//TRIM3//KLHL2//RNF38//MYCBP2//FBXO33//KLHL3//SPOPL//DCAF8//DCAF6//FEM1C//KLHL14//MARCH4//RNF4//MARCH7//UBE2Z//RNF128//KLHL15//KBTBD8//ARRDC4//UGP2//PFKP//GFPT2//NAGK//SLC35D1//IDH1//ACO1//NUP35//NUP43//NLN//IMMP2L//CHCHD10//COX8C//COX7A2L//COX7A2//GLRX//ASPH//GSTA2//DPYSL2//HPRT1//NME1//CMPK1//ADCY3//IMPDH1//PDE10A//DCTPP1//REV3L//GMNC//ESCO2//EPC2//USP28//USP45//ATXN3//KDM2A//HMGB3//BAHD1//H2AFZ//KMT2B//DPY30//ARID4B//KHDRBS3//ZMYND11//TADA1//ZNF597//CTNND2//ZNF782//NKX2-3//ZNF800//E2F5//ZNF367//EZH1//ZBTB9//FLII//LHX6//HBP1//ATAD2//SERTAD3//MDFIC//MNX1//HOXA3//HOXD1//FOXN2//ZBTB41//ZBTB34//LHX8//NEUROG1//NFE2//NPAS2//IER5//BRWD1//BNC2//ZNF532//CDCA7L//TRERF1//LMO3//SLC2A4RG//PHTF2//ZNF630//ZNF287//ZNF512B//ZBTB26//MIER1//RBL2//ATXN1//DMRTA1//SMARCD2//STAT4//ZNF2//ZNF3//ZNF131//ZNF655//BRD3//TCF7L1//PCGF5//LCOR//WASL//ZBTB47//ST18//HIPK3//NR5A2//NRBF2//MLLT6//RNF141//PAK6//SNAPC1//BTG1//SNIP1//RPS6KA4//RPS6KA5//EMX2//ETS1//BOLA3//NR3C2//PLEK//ZNFX1//NKX2-4//RFX7//BDP1//CDK8//E2F3//TEAD1//KAT2B//CCNC//CMTR2//ZFC3H1//RTCA//PPP4R2//ESRP1//CPEB1//QKI//CPEB2//CPEB4//MTPN//DDX6//MKNK2//TSC1//SERP1//UEVLD//RIMKLB//ADAM10//CDK6//FASTK//PDIK1L//CDK19//MORC3//MMD//DSTYK//LATS2//CDK17//PRKACB//FAM20C//CCL8//TGFB2//TGFBR2//ACVR1//STK17B//PPM1E//FLRT1//GNAQ//NCK1//NF2//ASPN//YWHAG//CAMK2N2//PPM1F//PTPN21//PPP1R12A//CTDSPL2//PPP3R1//PPP6C//PTPN4//NAA30//HS3ST5//PCMTD1//FUT9//ST8SIA5//MAN1A1//MAN2A1//B3GNT5//B3GALT2//CCDC126//EDEM1//VEGFB//NTNG1//RECK//PTAR1//PIGA//CTSA//USP24//USP27X//NPLOC4//USP46//BAG6//CDC23//SLC6A14//SLC6A6//GLUD2//LGSN//SLC25A32//BCAT2//SERINC3//PSAT1//ACOT7//UGT1A8//PRKAR2B//MSMO1//ELOVL4//SCD5//PLAA//LCLAT1//DDHD1//CDS1//ABHD3//SLC44A1//PIKFYVE//PLEKHA1//PLEKHA3//MTMR9//PIK3R3//KDELC1//SGPP1//SPHK2//NSMAF//ORMDL3//ZADH2//UGT1A4//UGT1A1//SLC20A1//SESN1//PMAIP1//UGT1A6//UGT1A9//RAB12//TBC1D17//TECPR2//DLC1//EGLN3//F3//DLL1//G3BP2//PTGER4//EPHA2//PTHLH//DGKE//ACBD5//PPCS//UGT1A7//RRAGD//NPNT//CALM2//GDF11//INHBB//BMP3//CNEP1R1//LDLR//PPP1R37//PPP1R12C//PLEKHF2//SERPINI1//SPOCK1//PCOLCE2//XYLT2//HS2ST1//PRR16//FXR1//INTS8//VPS37A//DYNLL2//CHMP3//SESN3//SNX5//RHEB//GNPTG//ST3GAL3//DGKH//HDHD2//RBM47//MAPRE3//BAMBI//RGMB//WNT7A//DKK3//RASD1//BTAF1//DMWD//OTUD3//WDR20//JOSD1//TOB1//NANOS1//CAPRIN2//VKORC1L1//CEP41//STK33//MASTL//EPHA4//EPHA5//PKDCC//OGN//TMX3//ZDHHC1//ST6GAL2//MCFD2//ZDHHC5//CRTAP//EGLN1//ELOVL7//PON2//ADIPOR2//SLK//SPTSSA//SULF1//DSEL//UBXN4//DNAJB9//DERL2//JKAMP//SOSTDC1//TMEM100//RGMA//ADRB1//BAG5//NDFIP2//GRIA1//RAP2C//CMKLR1//CCT6A//OSM//PFN2//IFNA1//UXS1//NSMF//PTPDC1//PTPRG//PTP4A1//CDC14A//WDFY3//GAB1//OSBPL5//DNAJB12//KCTD20//ITGA6//BTBD10//GNS//UGT1A3//ITM2B//TDRD5//ARL6IP1//CD2AP//BCL2L11//ITGAV//FZD6//AHNAK//YTHDF2//FZD10//AIDA//TWF1//CKAP4//RAB38//MIA3//ANO8//CCDC71L//FAM135A//MMP2//MID1IP1//IPO5//CCNY//S1PR1//PAIP2//MPP1//ENPP4//EFR3A//ZFYVE21//PPT2//ITGA5//SH3D19//ARRDC3//UGT1A10//UGT1A5//CHRM5//PMEPA1//LDLRAD4//DNAJC27//UBA5//REXO1//ZC3H12C//CALCOCO2//SNAP25//FYCO1//FEZ2//RAB3GAP1//MSN//TOB2//FRY//GJA1//UNK//TBC1D12// |
| GO:0001932 | regulation of protein phosphorylation | Biological process | 126 | 1421 | 934 | 17653 | 1.67590004324849 | 4.14236380673637e-09 | 3.22009269252392e-07 | 8.38275176147355 | 0.134903640256959 | CDKN1A//GADD45A//PTEN//CCNE2//MINK1//TNF//MAP3K2//CRK//CRKL//MAP3K5//TAOK3//LAMTOR3//ADAM9//DUSP5//LPAR1//LPAR3//NRG1//IGF1//NTF3//PIK3CB//PRKAA1//MAPK1//MAP2K4//DUSP10//SPRED1//DUSP1//DUSP2//PPP2CA//RGS3//FAM129A//IL2//LRP6//PPP1R15B//SLIT2//EFNA1//EFNA5//EPHA7//FMR1//NPTN//KDR//P2RY1//AKTIP//SQSTM1//CCND2//FNIP1//EREG//FBXW7//ERRFI1//TSG101//SOCS5//PPM1E//FLRT1//GNAQ//NCK1//NF2//ASPN//DUS2//YWHAG//CAMK2N2//PPM1F//COPS8//MDFIC//CALM2//GDF11//INHBB//BMP3//BMPR2//TGFB2//ACVR1//CDK6//PKD2//JUN//RAP2C//PAK6//TGFBR2//SLK//RPS6KA4//RPS6KA5//OSM//PFN2//CAPRIN2//IFNA1//ADCY3//PRKACB//PRKAR2B//CNOT7//STAT3//EDN1//GRB2//EPHA2//EPHA4//EPHA5//AR//ITGAV//MET//FZD10//HIPK3//AIDA//TWF1//LATS2//IPO5//KAT2B//CCNY//MAPRE3//CCNC//TSC1//MMD//VLDLR//ZMYND11//WNT7A//ITGA5//LRP8//PMEPA1//LDLRAD4//NOG//RANBP9//LMO3//KLF4//NPNT//DSTYK//DNAJC27//CCL2//CCL8//HAND2//ACSL1//DRD1// |
| GO:0018130 | heterocycle biosynthetic process | Biological process | 318 | 4550 | 934 | 17653 | 1.32095300844766 | 4.69319195006707e-09 | 3.60682467707995e-07 | 8.32853168291724 | 0.340471092077088 | E2F1//NPAT//RRM2//TSHZ1//HMG20A//KLF2//ZBTB18//CEBPB//ZNF268//TCERG1//WWP2//KLF12//OSR1//CRY2//GLIS3//ARX//DNMT3B//DR1//EDN1//EFNA1//CC2D1B//EN1//ESR1//FHL2//CPEB3//FOXF1//FOXC1//MYT1L//CIC//ZFPM2//DNAJB5//GATA6//CNOT7//HSBP1//AR//JARID2//JUN//ZNF568//MAF//MDM4//MYB//NFIA//NFIB//NR4A2//RLIM//KLF3//ZBTB7A//PIAS4//POU4F1//PPARG//CC2D1A//TSHZ3//ZBTB4//RARB//RBBP7//RBBP8//REST//RNF2//PRDM16//SOX17//SOX11//STAT3//ZEB1//TCF21//TFAP2A//TFAP2C//TGIF1//KLF10//TNF//UBE2I//VLDLR//WFS1//ZNF217//LRP8//BTG2//BHLHE41//TBL1XR1//NR4A3//HMGA2//ARID5B//BRMS1L//BHLHE40//RUNX3//URI1//PER2//SQSTM1//NOG//KLF4//HAND1//FNIP1//PHF14//USP3//GTF2E1//IRF9//KLF6//CREBRF//EGR2//ELK3//ERG//FOXJ3//FOXF2//FLI1//GRHL1//HIVEP2//IRF1//LHX1//MEF2D//MEOX2//MYBL1//NEUROD1//NHLH2//OTX2//PAX9//PBX3//POU3F2//RAD21//ARID4A//SHOX//BCL11B//BMPR2//SOX4//BTF3//TEF//TGFB1I1//THRA//TMF1//DEK//FOSL1//KLF7//CBFB//FUBP3//HAND2//CLOCK//POLR3G//SRSF2//PKD2//NAGK//GFPT2//SLC35D1//UGP2//NUP35//NUP43//HPRT1//ADCY3//IMPDH1//NME1//PDE10A//CMPK1//DCTPP1//BAHD1//H2AFZ//KMT2B//DPY30//TOPORS//KHDRBS3//ZMYND11//TADA1//ZNF597//CTNND2//ZNF782//NKX2-3//ZNF800//E2F5//ZNF367//EZH1//ZBTB9//KDM2A//MYCBP2//FLII//CNOT6L//EPC2//LHX6//HBP1//ATAD2//SERTAD3//MDFIC//MNX1//HMGB3//HOXA3//HOXD1//FOXN2//ZBTB41//ZBTB34//ATXN3//LHX8//NEUROG1//NFE2//NPAS2//IER5//ARID4B//BRWD1//BNC2//ZNF532//CDCA7L//PRKAA1//TRERF1//LMO3//MAPK1//SLC2A4RG//PHTF2//ZNF630//ZNF287//CNOT6//ZNF512B//ZBTB26//MIER1//RBL2//RNF4//ATXN1//DMRTA1//SMARCD2//STAT4//ZNF2//ZNF3//ZNF131//ZNF655//BRD3//TCF7L1//PCGF5//LCOR//WASL//ZBTB47//ST18//HIPK3//ELAVL2//NR5A2//NRBF2//MLLT6//RNF141//PPP2CA//PAK6//SNAPC1//BTG1//SNIP1//RPS6KA4//RPS6KA5//CDKN1A//CRK//EMX2//ETS1//BOLA3//NR3C2//PLEK//ZNFX1//NKX2-4//RFX7//BDP1//CDK8//E2F3//TEAD1//KAT2B//CCNC//CHCHD10//PTGES3//G3BP2//PSAT1//EREG//ACOT7//PPCS//MAP3K2//MAPRE3//BAMBI//RGMB//IGF1//LRP6//MAP3K5//WNT7A//ACVR1//DKK3//NRG1//RASD1//TSG101//BTAF1//PPM1F//TOB1//SOSTDC1//TMEM100//RGMA//BMP3//TGFB2//SULF1//CTDSPL2//ADRB1//PTHLH//CALM2//EGLN1//CMKLR1//DCP2//CCT6A//EPHA5//UXS1//REV3L//INTS8//NABP1//FZD6//NPLOC4//S1PR1//DLL1//IL2//ITGA6//MET//PPP1R12A//NCK1//OSM//P2RY1//PPP3R1//DCAF6//CAPRIN2//PTEN//MTPN//EGLN3//PFKP//RAP2C//TOB2//DUSP1//GJA1//FBXW7//NPNT// |
| GO:0031327 | negative regulation of cellular biosynthetic process | Biological process | 150 | 1789 | 934 | 17653 | 1.58472008933968 | 5.19764917952069e-09 | 3.91753587274999e-07 | 8.28419303698199 | 0.160599571734475 | TSHZ1//HMG20A//KLF2//ZBTB18//CEBPB//ZNF268//TCERG1//WWP2//KLF12//OSR1//CRY2//GLIS3//ARX//DNMT3B//DR1//E2F1//EDN1//EFNA1//CC2D1B//EN1//ESR1//FHL2//CPEB3//FOXF1//FOXC1//MYT1L//CIC//ZFPM2//DNAJB5//GATA6//CNOT7//HSBP1//AR//JARID2//JUN//ZNF568//MAF//MDM4//MYB//NFIA//NFIB//NR4A2//RLIM//KLF3//ZBTB7A//PIAS4//POU4F1//PPARG//CC2D1A//TSHZ3//ZBTB4//RARB//RBBP7//RBBP8//REST//RNF2//PRDM16//SOX17//SOX11//STAT3//ZEB1//TCF21//TFAP2A//TFAP2C//TGIF1//KLF10//TNF//UBE2I//VLDLR//WFS1//ZNF217//LRP8//BTG2//BHLHE41//TBL1XR1//NR4A3//HMGA2//ARID5B//BRMS1L//BHLHE40//RUNX3//URI1//PER2//SQSTM1//NOG//KLF4//HAND1//FNIP1//PHF14//USP3//BAHD1//H2AFZ//DPY30//NF2//SNIP1//FBXW7//PLEK//ELK3//EREG//FOXF2//DKK3//MDFIC//NRG1//IRF1//LHX1//PAX9//RASD1//ARID4A//ATXN1//THRA//TSG101//BTAF1//RPS6KA5//CLOCK//PPM1F//TOB1//FMR1//NANOS1//CAPRIN2//TSC1//FXR1//EGLN1//ATAD2//MIER1//DCP2//CNOT6//RNF128//ERRFI1//ITM2B//PAIP2//PRKAA1//INHBB//NUP35//NUP43//CNOT6L//YTHDF2//QKI//ORMDL3//CPEB2//TOB2//ZMYND11//NPAT//SRSF2//NCK1//DUSP1//GJA1//BMPR2//CPEB1//CPEB4//UNK// |
| GO:0032270 | positive regulation of cellular protein metabolic process | Biological process | 134 | 1546 | 934 | 17653 | 1.63820012133266 | 5.21334065573708e-09 | 3.91753587274999e-07 | 8.28288389600658 | 0.143468950749465 | GADD45A//MINK1//TNF//MAP3K2//CRK//CRKL//MAP3K5//TAOK3//LAMTOR3//ADAM9//DUSP5//LPAR1//LPAR3//NRG1//IGF1//NTF3//PIK3CB//PRKAA1//MAPK1//MAP2K4//FAM129A//EFNA1//EFNA5//EPHA7//FMR1//NPTN//KDR//P2RY1//AKTIP//SQSTM1//CCND2//FNIP1//EREG//FBXW7//DLC1//EGLN3//F3//PMAIP1//PPARG//COPS8//MDFIC//CALM2//GDF11//INHBB//BMP3//BMPR2//TGFB2//ACVR1//PCOLCE2//CPEB3//SERP1//PRR16//SOX4//FXR1//NDFIP2//WFS1//PKD2//RAP2C//PAK6//TGFBR2//SLK//RNF180//ANKIB1//LONRF3//LONRF1//SOCS5//PPP1R15B//RPS6KA4//RPS6KA5//OSM//PFN2//CAPRIN2//IFNA1//PIAS4//ADCY3//PRKACB//PRKAR2B//CNEP1R1//NSMF//PPP1R12A//NCK1//IL2//STAT3//BCL2L11//REST//PPM1F//EDN1//EPHA2//EPHA4//EPHA5//AR//ITGAV//MET//FZD10//CCNY//MAPRE3//CCNC//CDKN1A//MMD//VLDLR//ASPH//WNT7A//ITGA5//LRP8//SH3D19//TOPORS//ARRDC3//ARRDC4//DNMT3B//MYB//JARID2//NPNT//DSTYK//JUN//DNAJC27//PTEN//CCL2//CCL8//HAND2//ACSL1//PPP2CA//RAB3GAP1//FBXL5//MSN//YTHDF2//UBE2I//ESR1//EDEM1//ATXN3//BAG6//RNF128//LDLR//ST18//KLF4// |
| GO:0045934 | negative regulation of nucleobase-containing compound metabolic process | Biological process | 128 | 1457 | 934 | 17653 | 1.66043570211884 | 5.39543207982276e-09 | 4.00981397316938e-07 | 8.26797377018981 | 0.137044967880086 | TSHZ1//HMG20A//KLF2//ZBTB18//CEBPB//ZNF268//TCERG1//WWP2//KLF12//OSR1//CRY2//GLIS3//ARX//DNMT3B//DR1//E2F1//EDN1//EFNA1//CC2D1B//EN1//ESR1//FHL2//CPEB3//FOXF1//FOXC1//MYT1L//CIC//ZFPM2//DNAJB5//GATA6//CNOT7//HSBP1//AR//JARID2//JUN//ZNF568//MAF//MDM4//MYB//NFIA//NFIB//NR4A2//RLIM//KLF3//ZBTB7A//PIAS4//POU4F1//PPARG//CC2D1A//TSHZ3//ZBTB4//RARB//RBBP7//RBBP8//REST//RNF2//PRDM16//SOX17//SOX11//STAT3//ZEB1//TCF21//TFAP2A//TFAP2C//TGIF1//KLF10//TNF//UBE2I//VLDLR//WFS1//ZNF217//LRP8//BTG2//BHLHE41//TBL1XR1//NR4A3//HMGA2//ARID5B//BRMS1L//BHLHE40//RUNX3//URI1//PER2//SQSTM1//NOG//KLF4//HAND1//FNIP1//PHF14//USP3//BAHD1//H2AFZ//DPY30//NF2//ELK3//EREG//FOXF2//DKK3//MDFIC//NRG1//IRF1//LHX1//PAX9//RASD1//ARID4A//ATXN1//THRA//TSG101//BTAF1//RPS6KA5//CLOCK//PPM1F//EGLN1//ATAD2//MIER1//DCP2//FBXW7//TOB1//FMR1//TOB2//ZMYND11//NPAT//SRSF2//NCK1//KLHL15//DUSP1//GJA1//BMPR2// |
| GO:0019438 | aromatic compound biosynthetic process | Biological process | 318 | 4558 | 934 | 17653 | 1.31863453015288 | 5.72235454489911e-09 | 4.17990510218883e-07 | 8.24242523773608 | 0.340471092077088 | E2F1//NPAT//RRM2//TSHZ1//HMG20A//KLF2//ZBTB18//CEBPB//ZNF268//TCERG1//WWP2//KLF12//OSR1//CRY2//GLIS3//ARX//DNMT3B//DR1//EDN1//EFNA1//CC2D1B//EN1//ESR1//FHL2//CPEB3//FOXF1//FOXC1//MYT1L//CIC//ZFPM2//DNAJB5//GATA6//CNOT7//HSBP1//AR//JARID2//JUN//ZNF568//MAF//MDM4//MYB//NFIA//NFIB//NR4A2//RLIM//KLF3//ZBTB7A//PIAS4//POU4F1//PPARG//CC2D1A//TSHZ3//ZBTB4//RARB//RBBP7//RBBP8//REST//RNF2//PRDM16//SOX17//SOX11//STAT3//ZEB1//TCF21//TFAP2A//TFAP2C//TGIF1//KLF10//TNF//UBE2I//VLDLR//WFS1//ZNF217//LRP8//BTG2//BHLHE41//TBL1XR1//NR4A3//HMGA2//ARID5B//BRMS1L//BHLHE40//RUNX3//URI1//PER2//SQSTM1//NOG//KLF4//HAND1//FNIP1//PHF14//USP3//GTF2E1//IRF9//KLF6//CREBRF//EGR2//ELK3//ERG//FOXJ3//FOXF2//FLI1//GRHL1//HIVEP2//IRF1//LHX1//MEF2D//MEOX2//MYBL1//NEUROD1//NHLH2//OTX2//PAX9//PBX3//POU3F2//RAD21//ARID4A//SHOX//BCL11B//BMPR2//SOX4//BTF3//TEF//TGFB1I1//THRA//TMF1//DEK//FOSL1//KLF7//CBFB//FUBP3//HAND2//CLOCK//POLR3G//SRSF2//PKD2//NAGK//GFPT2//SLC35D1//UGP2//NUP35//NUP43//HPRT1//ADCY3//IMPDH1//NME1//PDE10A//CMPK1//DCTPP1//BAHD1//H2AFZ//KMT2B//DPY30//TOPORS//KHDRBS3//ZMYND11//TADA1//ZNF597//CTNND2//ZNF782//NKX2-3//ZNF800//E2F5//ZNF367//EZH1//ZBTB9//KDM2A//MYCBP2//FLII//CNOT6L//EPC2//LHX6//HBP1//ATAD2//SERTAD3//MDFIC//MNX1//HMGB3//HOXA3//HOXD1//FOXN2//ZBTB41//ZBTB34//ATXN3//LHX8//NEUROG1//NFE2//NPAS2//IER5//ARID4B//BRWD1//BNC2//ZNF532//CDCA7L//PRKAA1//TRERF1//LMO3//MAPK1//SLC2A4RG//PHTF2//ZNF630//ZNF287//CNOT6//ZNF512B//ZBTB26//MIER1//RBL2//RNF4//ATXN1//DMRTA1//SMARCD2//STAT4//ZNF2//ZNF3//ZNF131//ZNF655//BRD3//TCF7L1//PCGF5//LCOR//WASL//ZBTB47//ST18//HIPK3//ELAVL2//NR5A2//NRBF2//MLLT6//RNF141//PPP2CA//PAK6//SNAPC1//BTG1//SNIP1//RPS6KA4//RPS6KA5//CDKN1A//CRK//EMX2//ETS1//BOLA3//NR3C2//PLEK//ZNFX1//NKX2-4//RFX7//BDP1//CDK8//E2F3//TEAD1//KAT2B//CCNC//CHCHD10//PTGES3//G3BP2//PSAT1//EREG//ACOT7//PPCS//MAP3K2//MAPRE3//BAMBI//RGMB//IGF1//LRP6//MAP3K5//WNT7A//ACVR1//DKK3//NRG1//RASD1//TSG101//BTAF1//PPM1F//TOB1//SOSTDC1//TMEM100//RGMA//BMP3//TGFB2//SULF1//CTDSPL2//ADRB1//PTHLH//CALM2//EGLN1//CMKLR1//DCP2//CCT6A//EPHA5//UXS1//REV3L//INTS8//NABP1//FZD6//NPLOC4//S1PR1//DLL1//IL2//ITGA6//MET//PPP1R12A//NCK1//OSM//P2RY1//PPP3R1//DCAF6//CAPRIN2//PTEN//MTPN//EGLN3//PFKP//RAP2C//TOB2//DUSP1//GJA1//FBXW7//NPNT// |
| GO:0045595 | regulation of cell differentiation | Biological process | 148 | 1761 | 934 | 17653 | 1.58845166569997 | 5.74791031352301e-09 | 4.17990510218883e-07 | 8.24049001692019 | 0.158458244111349 | NME1//SOSTDC1//FOXC1//BAMBI//TGFB1I1//TGFB2//TGFBR2//EFNA1//PPP2CA//PTEN//LDLRAD4//FRZB//ITGAV//PPARG//THRA//BHLHE41//SNAP25//ZNF804A//STMN2//NPTN//NCK1//SERPINI1//TWF1//LPAR1//EPHA4//MYLIP//PTPRG//SPOCK1//TSC1//BAG5//NF2//DPYSL2//RAB21//KIAA1109//OSR1//DLL1//WNT7A//MAF//PTHLH//RARB//PKDCC//SOS1//ZEB1//SOX17//HMGB3//IL2//CBFB//IRF1//DUSP10//CDK6//GDF11//MAPK1//ZNF268//AR//JUN//NEUROD1//E2F1//ZFPM2//ZADH2//TNF//CEBPB//CMKLR1//CCDC71L//TMEM64//LMO3//TMEM100//BTG1//GRHL1//ERRFI1//MYB//SOCS5//GNAQ//ETS1//STAT3//NFE2//NR4A3//DPY30//KAT2B//KMT2B//CXCL14//MAP3K5//NTF3//BCL11B//YWHAG//HMG20A//DDX6//REST//DNMT3B//MMD//NEUROG1//SOX11//SMOC1//TOB1//NOG//HAND2//NPNT//GJA1//IGF1//FAM20C//BMPR2//ACVR1//TOB2//POU4F1//KLF10//GOLGA4//NRG1//EPHA7//ULK2//EFNA5//LPAR3//KLF4//RGMA//TNFRSF21//RHEB//FMR1//NSMF//HECW2//FXR1//SEMA4B//PER2//MAN2A1//POU3F2//SLIT2//CAPRIN2//FOXF1//EREG//GATA6//EDN1//CPEB3//C21ORF91//LRP8//LDLR//PRDM16//VLDLR//CRK//CRKL//DOCK5//YTHDF2//NLN//PLAA//CLOCK//LHX1//FNIP1//FBXW7//KDR//PLEKHB2//TFAP2A//RUNX3// |
| GO:0010605 | negative regulation of macromolecule metabolic process | Biological process | 216 | 2842 | 934 | 17653 | 1.43648575135585 | 6.27789184725863e-09 | 4.51674282585214e-07 | 8.20218617040459 | 0.231263383297645 | TSHZ1//HMG20A//KLF2//ZBTB18//CEBPB//ZNF268//TCERG1//WWP2//KLF12//OSR1//CRY2//GLIS3//ARX//DNMT3B//DR1//E2F1//EDN1//EFNA1//CC2D1B//EN1//ESR1//FHL2//CPEB3//FOXF1//FOXC1//MYT1L//CIC//ZFPM2//DNAJB5//GATA6//CNOT7//HSBP1//AR//JARID2//JUN//ZNF568//MAF//MDM4//MYB//NFIA//NFIB//NR4A2//RLIM//KLF3//ZBTB7A//PIAS4//POU4F1//PPARG//CC2D1A//TSHZ3//ZBTB4//RARB//RBBP7//RBBP8//REST//RNF2//PRDM16//SOX17//SOX11//STAT3//ZEB1//TCF21//TFAP2A//TFAP2C//TGIF1//KLF10//TNF//UBE2I//VLDLR//WFS1//ZNF217//LRP8//BTG2//BHLHE41//TBL1XR1//NR4A3//HMGA2//ARID5B//BRMS1L//BHLHE40//RUNX3//URI1//PER2//SQSTM1//NOG//KLF4//HAND1//FNIP1//PHF14//USP3//DCP2//PPP2CA//RPL15//DUSP10//SPRED1//DUSP1//DUSP2//DUSP5//RGS3//CNOT6L//PAN3//CNOT6//FAM129A//IL2//LRP6//PTEN//PPP1R15B//SLIT2//ERRFI1//TSG101//SOCS5//BAHD1//H2AFZ//DPY30//GADD45A//PPM1E//FLRT1//GNAQ//NCK1//NF2//TAOK3//ASPN//DUS2//YWHAG//CAMK2N2//PPM1F//SNIP1//CDKN1A//GJA1//ITGB8//KDR//LDLR//NME1//POU3F2//NDFIP2//RBL2//TGFB2//TMF1//HAND2//CALM2//SERPINI1//SPOCK1//RECK//KMT2B//ELK3//EREG//FOXF2//DKK3//MDFIC//NRG1//IRF1//LHX1//PAX9//RASD1//ARID4A//ATXN1//THRA//BTAF1//RPS6KA5//CLOCK//TOB1//FMR1//NANOS1//CAPRIN2//TSC1//FXR1//SPOPL//SOX4//BAG5//CDK6//ATAD2//MIER1//BAG6//PPP1R37//MASTL//FBXW7//RNF4//RNF128//ITM2B//ARL6IP1//YTHDF2//HIPK3//AIDA//DDX6//ITGAV//TMEFF2//LATS2//IPO5//KAT2B//PAIP2//ZMYND11//INHBB//NTF3//PMEPA1//LDLRAD4//NUP35//NUP43//QKI//RANBP9//LMO3//PLAA//CPEB2//CRTAP//EPHA4//TOB2//NPAT//SRSF2//DERL2//FRY//CTSA//KLHL15//BMPR2//PRKAR2B//CPEB1//CPEB4//UNK// |
| GO:0051338 | regulation of transferase activity | Biological process | 95 | 988 | 934 | 17653 | 1.81734887168506 | 8.77493872990839e-09 | 6.24683269793373e-07 | 8.05675590726733 | 0.101713062098501 | CDKN1A//GADD45A//PTEN//CCNE2//MINK1//TNF//MAP3K2//CRK//CRKL//MAP3K5//TAOK3//LAMTOR3//ADAM9//DUSP5//LPAR1//LPAR3//NRG1//IGF1//NTF3//PIK3CB//PRKAA1//MAPK1//MAP2K4//DUSP10//SPRED1//DUSP1//DUSP2//PPP2CA//RGS3//EREG//FBXW7//ERRFI1//TSG101//SOCS5//PPM1E//FLRT1//GNAQ//LRP6//NCK1//NF2//ASPN//DUS2//YWHAG//CAMK2N2//PPM1F//EFNA5//COPS8//MDFIC//CDK6//PKD2//PAK6//TGFB2//TGFBR2//SLK//EPHA2//EPHA4//EPHA5//EPHA7//DSTYK//KDR//MET//ADCY3//PRKACB//PRKAR2B//EDN1//FZD10//HIPK3//AIDA//NRBF2//RBL2//PIK3R3//KLF4//LATS2//IPO5//KAT2B//CCNY//MAPRE3//CCNC//CCND2//TSC1//MMD//VLDLR//BAG5//TOPORS//ARRDC3//ARRDC4//PTGES3//PPARG//RAP2C//EFNA1//LRP8//ACSL1//CALM2//UBE2I//CHCHD10// |
| GO:2000113 | negative regulation of cellular macromolecule biosynthetic process | Biological process | 139 | 1636 | 934 | 17653 | 1.6058432328288 | 9.22767034083862e-09 | 6.50070151198871e-07 | 8.03490792907422 | 0.148822269807281 | TSHZ1//HMG20A//KLF2//ZBTB18//CEBPB//ZNF268//TCERG1//WWP2//KLF12//OSR1//CRY2//GLIS3//ARX//DNMT3B//DR1//E2F1//EDN1//EFNA1//CC2D1B//EN1//ESR1//FHL2//CPEB3//FOXF1//FOXC1//MYT1L//CIC//ZFPM2//DNAJB5//GATA6//CNOT7//HSBP1//AR//JARID2//JUN//ZNF568//MAF//MDM4//MYB//NFIA//NFIB//NR4A2//RLIM//KLF3//ZBTB7A//PIAS4//POU4F1//PPARG//CC2D1A//TSHZ3//ZBTB4//RARB//RBBP7//RBBP8//REST//RNF2//PRDM16//SOX17//SOX11//STAT3//ZEB1//TCF21//TFAP2A//TFAP2C//TGIF1//KLF10//TNF//UBE2I//VLDLR//WFS1//ZNF217//LRP8//BTG2//BHLHE41//TBL1XR1//NR4A3//HMGA2//ARID5B//BRMS1L//BHLHE40//RUNX3//URI1//PER2//SQSTM1//NOG//KLF4//HAND1//FNIP1//PHF14//USP3//BAHD1//H2AFZ//DPY30//NF2//SNIP1//ELK3//EREG//FOXF2//DKK3//MDFIC//NRG1//IRF1//LHX1//PAX9//RASD1//ARID4A//ATXN1//THRA//TSG101//BTAF1//RPS6KA5//CLOCK//PPM1F//TOB1//FMR1//NANOS1//CAPRIN2//TSC1//FXR1//ATAD2//MIER1//DCP2//FBXW7//CNOT6//ITM2B//PAIP2//NUP35//NUP43//CNOT6L//YTHDF2//QKI//CPEB2//NCK1//DUSP1//GJA1//BMPR2//CPEB1//CPEB4//UNK// |
| GO:0050789 | regulation of biological process | Biological process | 697 | 11709 | 934 | 17653 | 1.1250831412649 | 1.08237365161229e-08 | 7.54648763490095e-07 | 7.9656227883789 | 0.746252676659529 | E2F1//USP28//CLOCK//CDKN1A//GADD45A//PTEN//CCNE2//NPAT//RRM2//TSHZ1//HMG20A//KLF2//ZBTB18//CEBPB//ZNF268//TCERG1//WWP2//KLF12//OSR1//CRY2//GLIS3//ARX//DNMT3B//DR1//EDN1//EFNA1//CC2D1B//EN1//ESR1//FHL2//CPEB3//FOXF1//FOXC1//MYT1L//CIC//ZFPM2//DNAJB5//GATA6//CNOT7//HSBP1//AR//JARID2//JUN//ZNF568//MAF//MDM4//MYB//NFIA//NFIB//NR4A2//RLIM//KLF3//ZBTB7A//PIAS4//POU4F1//PPARG//CC2D1A//TSHZ3//ZBTB4//RARB//RBBP7//RBBP8//REST//RNF2//PRDM16//SOX17//SOX11//STAT3//ZEB1//TCF21//TFAP2A//TFAP2C//TGIF1//KLF10//TNF//UBE2I//VLDLR//WFS1//ZNF217//LRP8//BTG2//BHLHE41//TBL1XR1//NR4A3//HMGA2//ARID5B//BRMS1L//BHLHE40//RUNX3//URI1//PER2//SQSTM1//NOG//KLF4//HAND1//FNIP1//PHF14//USP3//RANBP9//PPM1L//SPRED1//DUSP5//EREG//RASGEF1A//GRB2//NRG1//IL2//JAK1//MAP3K5//MET//MINK1//TAOK3//MAPK1//CCL2//SOS1//LAMTOR3//DCP2//PPP2CA//RPL15//MAP3K2//CRK//CRKL//ADAM9//LPAR1//LPAR3//IGF1//NTF3//PIK3CB//PRKAA1//MAP2K4//DUSP10//DUSP1//DUSP2//RGS3//CNOT6L//PAN3//CNOT6//RBFOX3//FMR1//MBNL1//RBM25//SRSF2//SECISBP2L//NANOS1//SPOCK1//TSG101//KIAA1109//RPS6KA4//RPS6KA5//TMF1//PMAIP1//BCL2L11//MOAP1//CAMLG//EPS15//NSF//DRD1//FAM129A//LRP6//PPP1R15B//SLIT2//EFNA5//EPHA7//NPTN//KDR//P2RY1//AKTIP//CCND2//SULF1//GJA1//F3//BMPR2//VEGFB//VASH2//TSC1//NF2//CDK6//CBFB//ADRB1//CALM2//ARL6IP1//TGFBR2//GLRA1//PBX3//THRA//PRKACB//SKP1//BAG6//MARCH7//OSM//MIA3//CCL8//NME1//IRF1//HAND2//S1PR1//GPR6//SPHK2//PKD2//E2F5//MASTL//CCNG2//EPS8//TWF1//APCDD1//CTNND2//AMER2//CCNY//SOSTDC1//HBP1//DKK3//STRN//TGFB1I1//WNT7A//TCF7L1//PPP3R1//FZD6//FAM13A//CNIH1//DLC1//CDS1//CDC42EP3//GNB5//PTGES3//IQGAP2//ADCY3//RASSF8//ARAP2//TAGAP//CLCN6//DPYSL2//ELK3//STX2//ERG//EVI2A//LRCH1//CD2AP//APPL1//GLRB//RASSF3//RGMB//GRIA1//CD274//LRP12//HIVEP2//NR3C2//MPP1//PPP1R12A//RASD1//KCNK10//NDFIP2//PPP1R12C//PSD//ARRDC3//MIER1//RHEB//SMOC1//SMOC2//BCL11B//SLC20A1//RASL11B//DEK//PDCD1LG2//NSMAF//PPFIA1//RHPN2//OR6A2//ARRDC4//TRIP10//PLAA//ARHGAP12//CXCL14//FEZ2//CD69//ULK2//PTPRG//AKAP11//DGKH//DCX//PIKFYVE//DSTYK//LATS2//MKNK2//ARHGEF4//TREM1//PRKAR2B//ARHGEF28//STK33//DGKE//STK17B//PALM2//FBXW7//ERRFI1//SOCS5//NUP35//NUP43//NLN//DNAJC16//GLRX//NHLRC2//TMX3//ESCO2//BAHD1//H2AFZ//KMT2B//DPY30//ARID4B//ARID4A//HIPK3//KHDRBS3//ZMYND11//ZNF597//ZNF782//ZNF800//ELAVL2//ZBTB9//KDM2A//MYCBP2//FLII//NR5A2//GTF2E1//SERTAD3//NRBF2//HMGB3//ZBTB41//ZBTB34//ATXN3//MLLT6//RNF141//BNC2//ZNF532//CDCA7L//LMO3//SLC2A4RG//PAK6//PHTF2//ZNF630//ZNF512B//ZBTB26//SNAPC1//SOX4//BTF3//BTG1//ZNF2//ZNF3//ZNF131//ZNF655//SNIP1//WASL//ZBTB47//IRF9//TADA1//ZNF367//EMX2//ETS1//FOXJ3//FOXF2//FLI1//EPC2//LHX6//ATAD2//GRHL1//MNX1//HOXA3//HOXD1//FOXN2//BOLA3//LHX8//MYBL1//NEUROG1//NFE2//NHLH2//PLEK//BRWD1//POU3F2//TRERF1//ZNFX1//RAD21//RBL2//DMRTA1//NKX2-4//RFX7//SMARCD2//TEF//BRD3//FOSL1//LCOR//KLF7//POLR3G//BDP1//MTPN//DDX6//ACO1//KBTBD8//PPM1E//FLRT1//GNAQ//NCK1//ASPN//DUS2//YWHAG//CAMK2N2//PPM1F//EGLN3//CHRM2//STMN2//MID1IP1//MTBP//RRAGD//TGFB2//KAT2B//CDC23//NABP1//CDC14A//ADAM10//GAB1//COL1A2//ACVR1//ZFYVE9//PDE10A//RAPGEF4//FZD10//CHRM5//FGD4//CMKLR1//GPR180//FRZB//RHOC//GPRC5C//PTGER4//PTHLH//SOS2//DLL1//EPN1//TMEM100//TMEM17//DZIP1//ITGA6//ITGA5//ITGAV//ITGB8//NKIRAS1//COPS8//G3BP2//MDFIC//STAT4//NEUROD1//RGL1//DOCK9//RAB38//RND3//ARHGAP1//RALGPS2//DOCK5//ARHGAP6//CDK8//CDK19//SLK//TRPC3//MARCKS//SNAP25//TOPORS//EPHA2//EPHA4//EPHA5//ZFAND6//BNIP3//ACTC1//GIT2//E2F3//BAMBI//DERL2//TOB1//GDF11//TOB2//BTG3//TES//ASPH//ST18//PIK3R3//WASF3//FGD5//MSN//HS3ST5//ESRP1//OTX2//NPNT//ADIPOR2//PIGA//RGS17//NEDD1//HAUS8//CEP41//LHX1//IFNA1//INHBB//OGN//BMP3//TTR//MTMR9//PPP4R2//RAB3GAP1//LDLR//FUBP3//ZNF804A//QKI//PFN2//TMED9//LDLRAD4//C5ORF30//CHMP3//CNEP1R1//PPP1R37//PLEKHF2//SERPINI1//RECK//PCOLCE2//MYLIP//BAG5//PMEPA1//SLC6A1//PLEKHA1//SERP1//PRR16//FXR1//SESN3//RAB12//SESN1//SNX5//CPEB2//IPO5//TNFRSF21//ANP32E//KLF6//EGR2//MAPRE3//NPAS2//ZNF287//RNF4//TEAD1//PAX9//ATXN1//BTAF1//CAPRIN2//RAB21//SYT6//TRPV6//ACSL1//ACOX1//UGT1A9//MMP2//SH3D19//DENND1B//HECW2//RUFY2//LAMA3//SPOPL//SHISA2//ENPP4//SEMA4B//PTP4A1//TMEFF2//CLIC4//RAP2C//FAM20C//PKDCC//RGMA//CTDSPL2//EGLN1//LIMA1//CFL2//CREBRF//SLAIN2//CLIP1//TBC1D12//EVI5//TBC1D17//USP6NL//AGFG1//AGFG2//CCT6A//USP46//STIM2//RNF180//ANKIB1//LONRF3//LONRF1//KIF13A//KIF23//NPLOC4//RAB30//RAB9B//DNAJC27//RAB23//RAB22A//RAB28//RAP1B//STX6//MMD//MEMO1//FRMD6//UBA5//ARFIP1//KCNA4//KCNJ3//KCNJ10//HCN2//SCN1A//CPEB4//NSMF//GSTA2//SYBU//DNAJB9//EDEM1//ZMAT3//RNF128//NKX2-3//IER5//KCTD20//BTBD10//ITM2B//LYPLA1//USP27X//UBE2Z//PXK//PGRMC2//FASTK//AHNAK//YTHDF2//AIDA//UBXN2B//PEX5L//TMEM64//TBC1D20//PLEKHB2//ZADH2//CCDC71L//CEP97//CCNC//ADAM12//GOLGA4//UGT1A8//UGT1A1//EZH1//MEF2D//MEOX2//DCAF6//SHOX//PCGF5//PAIP2//RAET1E//HPRT1//MDM1//MORC3//MAN2A1//NOVA1//GPM6A//TMOD3//TMOD1//OTUD3//SLITRK3//VANGL1//SLC17A7//SLC24A2//NTN4//BTBD7//IDH1//C21ORF91//FIBIN//MCOLN2//GJC1//DSC2//SACS//TMEM135//MIEF1//DDHD1//SGPP1//LYPD6//ORMDL3//DYNLL2//CALCOCO2//FYCO1//CRTAP//SERINC3//NEFH//FBXL5//ELMOD1//NAPEPLD//FRY//CTSA//SESTD1//CENPQ//LHFPL2//KLHL15//IER3IP1//CPEB1//UNK// |
| GO:0009892 | negative regulation of metabolic process | Biological process | 229 | 3075 | 934 | 17653 | 1.40754408871712 | 1.10903773907732e-08 | 7.65349207079583e-07 | 7.95505367513653 | 0.245182012847966 | TSHZ1//HMG20A//KLF2//ZBTB18//CEBPB//ZNF268//TCERG1//WWP2//KLF12//OSR1//CRY2//GLIS3//ARX//DNMT3B//DR1//E2F1//EDN1//EFNA1//CC2D1B//EN1//ESR1//FHL2//CPEB3//FOXF1//FOXC1//MYT1L//CIC//ZFPM2//DNAJB5//GATA6//CNOT7//HSBP1//AR//JARID2//JUN//ZNF568//MAF//MDM4//MYB//NFIA//NFIB//NR4A2//RLIM//KLF3//ZBTB7A//PIAS4//POU4F1//PPARG//CC2D1A//TSHZ3//ZBTB4//RARB//RBBP7//RBBP8//REST//RNF2//PRDM16//SOX17//SOX11//STAT3//ZEB1//TCF21//TFAP2A//TFAP2C//TGIF1//KLF10//TNF//UBE2I//VLDLR//WFS1//ZNF217//LRP8//BTG2//BHLHE41//TBL1XR1//NR4A3//HMGA2//ARID5B//BRMS1L//BHLHE40//RUNX3//URI1//PER2//SQSTM1//NOG//KLF4//HAND1//FNIP1//PHF14//USP3//DCP2//PPP2CA//RPL15//DUSP10//SPRED1//DUSP1//DUSP2//DUSP5//RGS3//CNOT6L//PAN3//CNOT6//FAM129A//IL2//LRP6//PTEN//PPP1R15B//SLIT2//ERRFI1//TSG101//SOCS5//BAHD1//H2AFZ//DPY30//GADD45A//PPM1E//FLRT1//GNAQ//NCK1//NF2//TAOK3//ASPN//DUS2//YWHAG//CAMK2N2//PPM1F//SNIP1//MET//MTMR9//CDKN1A//GJA1//ITGB8//KDR//LDLR//NME1//POU3F2//NDFIP2//RBL2//TGFB2//TMF1//HAND2//BNIP3//CALM2//FBXW7//PLEK//PPP1R37//PPP1R12A//PPP1R12C//PLEKHF2//SERPINI1//SPOCK1//RECK//TSC1//KMT2B//ELK3//EREG//FOXF2//DKK3//MDFIC//NRG1//IRF1//LHX1//PAX9//RASD1//ARID4A//ATXN1//THRA//BTAF1//RPS6KA5//CLOCK//TOB1//FMR1//NANOS1//CAPRIN2//FXR1//EGLN1//SPOPL//SOX4//BAG5//CDK6//ATAD2//MIER1//BAG6//MASTL//RNF4//RNF128//ITM2B//ARL6IP1//YTHDF2//HIPK3//AIDA//DDX6//ITGAV//TMEFF2//LATS2//IPO5//KAT2B//UGT1A8//UGT1A1//PAIP2//PRKAA1//ZMYND11//INHBB//NTF3//PMEPA1//LDLRAD4//NUP35//NUP43//QKI//RANBP9//LMO3//PLAA//ORMDL3//CPEB2//CRTAP//FEZ2//EPHA4//TOB2//NPAT//SRSF2//DERL2//FRY//CTSA//KLHL15//BMPR2//PRKAR2B//CPEB1//CPEB4//UNK// |
| GO:0009812 | flavonoid metabolic process | Biological process | 9 | 15 | 934 | 17653 | 11.3402569593148 | 1.17343118447077e-08 | 8.01607585916749e-07 | 7.9305423743917 | 0.00963597430406852 | UGT1A10//UGT1A8//UGT1A7//UGT1A9//UGT1A1//UGT1A6//UGT1A5//UGT1A4//UGT1A3// |
| GO:0009888 | tissue development | Biological process | 160 | 1969 | 934 | 17653 | 1.53583977779784 | 1.2953583653361e-08 | 8.76050862476804e-07 | 7.88761006590137 | 0.171306209850107 | THRA//WNT7A//EDN1//PPP3R1//TGFBR2//ACVR1//GDF11//OSR1//FOXC1//NPNT//LHX1//RARB//TCF21//SLIT2//PKD2//DUSP1//DUSP2//DUSP5//SOX17//NOG//NF2//BMPR2//NR4A3//HAND1//SEMA4B//FOXF1//DLL1//MEOX2//FOXF2//TGFB2//HMGA2//SOX4//SOX11//DLC1//LRP6//PRKACB//TSC1//FZD6//CLIC4//MET//APCDD1//SOS1//GJA1//HAND2//ARHGAP12//MEF2D//NFIB//RUNX3//SULF1//ESR1//KDR//NRG1//ERG//TMEM100//EFNA1//ZFPM2//POU4F1//S1PR1//GATA6//CDK6//NEUROD1//PALLD//BCL11B//AR//FRMD6//TFAP2A//STX2//AMER2//PAX9//PPP2CA//KLF4//ZBTB18//CFL2//GRHL1//LAMA3//POU3F2//PTHLH//ITGA6//BAMBI//TGFB1I1//PTEN//LDLRAD4//KBTBD8//RDH10//FRZB//IGF1//MTPN//EPHA2//EREG//LATS2//TSG101//ADAM9//ASPN//KLF10//PKDCC//FAM20C//CNN3//NR5A2//GSTA2//PPARG//KIAA1109//EN1//SOSTDC1//KRT27//DSC2//MAF//CEBPB//MAPK1//JUN//ITGA5//EGR2//FOXN2//BTG2//HSBP1//ITGAV//MMP2//TMEFF2//RHOC//MSN//ZEB1//BTG1//ERRFI1//CBFB//GJB2//NKX2-3//PRKAA1//GJC1//HOXA3//ITGB8//BMP3//TNF//ACTC1//EGLN1//FHL2//JARID2//VANGL1//BNC2//KLF2//CDKN1A//ARID5B//ARRDC3//TBL1XR1//NTN4//GRB2//BTBD7//RAP1B//MAP2K4//TMOD1//TBC1D20//ARX//KLHL3//EPHA4//EPHA7//RAP2C//ARID4B//ARID4A//NLN//DUSP10//RHEB//CLOCK// |
| GO:0032268 | regulation of cellular protein metabolic process | Biological process | 211 | 2792 | 934 | 17653 | 1.42836331089746 | 1.61017039696781e-08 | 1.07817647472211e-06 | 7.79312816213769 | 0.225910064239829 | CDKN1A//GADD45A//PTEN//CCNE2//MINK1//TNF//MAP3K2//CRK//CRKL//MAP3K5//TAOK3//LAMTOR3//ADAM9//DUSP5//LPAR1//LPAR3//NRG1//IGF1//NTF3//PIK3CB//PRKAA1//MAPK1//MAP2K4//DUSP10//SPRED1//DUSP1//DUSP2//PPP2CA//RGS3//SECISBP2L//DRD1//FNIP1//FAM129A//IL2//LRP6//PPP1R15B//SLIT2//EFNA1//EFNA5//EPHA7//FMR1//NPTN//KDR//P2RY1//AKTIP//SQSTM1//CCND2//EREG//FBXW7//ERRFI1//TSG101//SOCS5//MTPN//DDX6//MKNK2//ACO1//TSC1//KBTBD8//PPM1E//FLRT1//GNAQ//NCK1//NF2//ASPN//DUS2//YWHAG//CAMK2N2//PPM1F//DLC1//EGLN3//F3//PMAIP1//PPARG//COPS8//MDFIC//TMF1//SNIP1//CALM2//KAT2B//GDF11//INHBB//BMP3//BMPR2//TGFB2//ACVR1//SERPINI1//SPOCK1//RECK//PCOLCE2//CPEB3//SERP1//PRR16//SOX4//FXR1//TOB1//CNOT7//NANOS1//CAPRIN2//BTG2//JARID2//SPOPL//PER2//BAG5//NDFIP2//WFS1//CDK6//PKD2//JUN//RAP2C//PAK6//TGFBR2//SLK//EDN1//WNT7A//KLF4//BAG6//RNF180//ANKIB1//LONRF3//LONRF1//CRY2//PPP1R37//MASTL//URI1//MYLIP//RPS6KA4//RPS6KA5//OSM//PFN2//IFNA1//HMG20A//RNF4//PIAS4//ADCY3//PRKACB//PRKAR2B//CNOT6//STAT3//CNEP1R1//NSMF//PPP1R12A//ARL6IP1//BCL2L11//REST//GRB2//EPHA2//EPHA4//EPHA5//AR//ITGAV//MET//FZD10//HIPK3//AIDA//TWF1//UBXN2B//PPP4R2//LATS2//IPO5//CCNY//MAPRE3//CCNC//MMD//VLDLR//ASPH//PAIP2//ZMYND11//ITGA5//LRP8//SH3D19//TOPORS//ARRDC3//ARRDC4//KMT2B//DNMT3B//MYB//PMEPA1//LDLRAD4//NOG//NUP35//NUP43//CNOT6L//YTHDF2//QKI//RANBP9//LMO3//NPNT//DSTYK//DNAJC27//CCL2//CCL8//HAND2//ACSL1//PLAA//CPEB2//CRTAP//RAB3GAP1//FBXL5//MSN//UBE2I//ESR1//DERL2//EDEM1//ATXN3//RNF128//FRY//LDLR//CPEB1//CPEB4//UNK//ST18// |
| GO:0043549 | regulation of kinase activity | Biological process | 87 | 889 | 934 | 17653 | 1.84964821046191 | 1.73371287301354e-08 | 1.1495196235481e-06 | 7.76102282611649 | 0.0931477516059957 | CDKN1A//GADD45A//PTEN//CCNE2//MINK1//TNF//MAP3K2//CRK//CRKL//MAP3K5//TAOK3//LAMTOR3//ADAM9//DUSP5//LPAR1//LPAR3//NRG1//IGF1//NTF3//PIK3CB//PRKAA1//MAPK1//MAP2K4//DUSP10//SPRED1//DUSP1//DUSP2//PPP2CA//RGS3//EREG//FBXW7//ERRFI1//TSG101//SOCS5//PPM1E//FLRT1//GNAQ//LRP6//NCK1//NF2//ASPN//DUS2//YWHAG//CAMK2N2//PPM1F//EFNA5//COPS8//MDFIC//CDK6//PKD2//PAK6//TGFB2//TGFBR2//SLK//EPHA2//EPHA4//EPHA5//EPHA7//DSTYK//KDR//MET//ADCY3//PRKACB//PRKAR2B//EDN1//FZD10//HIPK3//AIDA//NRBF2//RBL2//PIK3R3//KLF4//LATS2//IPO5//KAT2B//CCNY//MAPRE3//CCNC//CCND2//TSC1//MMD//VLDLR//RAP2C//EFNA1//LRP8//ACSL1//CALM2// |
| GO:0060021 | roof of mouth development | Biological process | 20 | 87 | 934 | 17653 | 4.34492603805164 | 1.96218539628644e-08 | 1.28837474127041e-06 | 7.70725996088051 | 0.0214132762312634 | SOX11//TSHZ1//HAND2//GDF11//OSR1//FOXF2//LRP6//MEOX2//ASPH//BNC2//PLEKHA1//PRDM16//SOS1//TCF21//TFAP2A//TGFB2//TGFBR2//WNT7A//ARID5B//PKDCC// |
| GO:0010468 | regulation of gene expression | Biological process | 333 | 4873 | 934 | 17653 | 1.29157451516924 | 2.0615753900528e-08 | 1.34061868874299e-06 | 7.68580077879362 | 0.356531049250535 | E2F1//NPAT//RRM2//TSHZ1//HMG20A//KLF2//ZBTB18//CEBPB//ZNF268//TCERG1//WWP2//KLF12//OSR1//CRY2//GLIS3//ARX//DNMT3B//DR1//EDN1//EFNA1//CC2D1B//EN1//ESR1//FHL2//CPEB3//FOXF1//FOXC1//MYT1L//CIC//ZFPM2//DNAJB5//GATA6//CNOT7//HSBP1//AR//JARID2//JUN//ZNF568//MAF//MDM4//MYB//NFIA//NFIB//NR4A2//RLIM//KLF3//ZBTB7A//PIAS4//POU4F1//PPARG//CC2D1A//TSHZ3//ZBTB4//RARB//RBBP7//RBBP8//REST//RNF2//PRDM16//SOX17//SOX11//STAT3//ZEB1//TCF21//TFAP2A//TFAP2C//TGIF1//KLF10//TNF//UBE2I//VLDLR//WFS1//ZNF217//LRP8//BTG2//BHLHE41//TBL1XR1//NR4A3//HMGA2//ARID5B//BRMS1L//BHLHE40//RUNX3//URI1//PER2//SQSTM1//NOG//KLF4//HAND1//FNIP1//PHF14//USP3//DCP2//PPP2CA//RPL15//CNOT6L//PAN3//CNOT6//RBFOX3//FMR1//MBNL1//RBM25//SRSF2//SECISBP2L//PKD2//THRA//BAHD1//H2AFZ//KMT2B//DPY30//ARID4B//ARID4A//HIPK3//KHDRBS3//ZMYND11//NUP35//ZNF597//CTNND2//ZNF782//ZNF800//ELAVL2//ZBTB9//KDM2A//MYCBP2//FLII//NR5A2//GTF2E1//SERTAD3//NRBF2//HIVEP2//HMGB3//ZBTB41//ZBTB34//ATXN3//MLLT6//RNF141//BNC2//ZNF532//CDCA7L//LMO3//SLC2A4RG//PAK6//PHTF2//ZNF630//ZNF512B//ZBTB26//BCL11B//SNAPC1//SOX4//BTF3//BTG1//TMF1//ZNF2//ZNF3//ZNF131//ZNF655//SNIP1//TCF7L1//WASL//RPS6KA4//RPS6KA5//ZBTB47//CLOCK//CDKN1A//IRF9//TADA1//CRK//ZNF367//ELK3//EMX2//ERG//ETS1//FOXJ3//FOXF2//FLI1//EPC2//LHX6//HBP1//ATAD2//GRHL1//MNX1//HOXA3//HOXD1//FOXN2//BOLA3//NR3C2//LHX8//MYBL1//NEUROG1//NFE2//NHLH2//PLEK//BRWD1//POU3F2//TRERF1//ZNFX1//MIER1//RAD21//RBL2//DMRTA1//NKX2-4//RFX7//SMARCD2//TEF//DEK//BRD3//FOSL1//LCOR//KLF7//CBFB//POLR3G//BDP1//MTPN//DDX6//MKNK2//ACO1//TSC1//KBTBD8//KAT2B//G3BP2//NANOS1//DRD1//RAB3GAP1//GJA1//IGF1//ITGB8//LDLR//MSN//PIK3CB//PRKAA1//MAPK1//PTEN//ACTC1//FUBP3//ZNF804A//QKI//HAND2//PPM1F//KDR//NME1//NDFIP2//TGFB2//FAM129A//SERP1//PRR16//FXR1//TOPORS//MAP3K2//KLF6//EGR2//MAPRE3//BAMBI//RGMB//MDFIC//IRF1//LHX1//LRP6//MAP3K5//NEUROD1//NPAS2//OTX2//ZNF287//RNF4//TEAD1//TGFB1I1//WNT7A//ACVR1//EREG//DKK3//NRG1//PAX9//RASD1//ATXN1//TSG101//BTAF1//TOB1//CAPRIN2//P2RY1//SOSTDC1//TMEM100//RGMA//BMP3//BMPR2//SULF1//CTDSPL2//CMKLR1//EPHA5//NCK1//EGLN1//FZD6//FASTK//ESRP1//AHNAK//YTHDF2//CDK8//CREBRF//NKX2-3//E2F3//E2F5//S1PR1//EZH1//DLL1//IL2//ITGA6//MEF2D//MEOX2//MET//PPP1R12A//OSM//PBX3//IER5//PPP3R1//DCAF6//SHOX//STAT4//PCGF5//CCNC//ST18//PAIP2//NUP43//EGLN3//PRKACB//CPEB2//PPP1R15B//FBXW7//NPNT//CPEB1//CPEB4//UNK//CDK6//TOB2//PTHLH//TGFBR2// |
| GO:0007610 | behavior | Biological process | 64 | 583 | 934 | 17653 | 2.07483260547783 | 2.3157834500629e-08 | 1.48339898742247e-06 | 7.63530205411548 | 0.0685224839400428 | DRD1//USP46//DERL2//POU4F1//HAND2//EGR2//LHX8//MAPK1//PTEN//THRA//CIC//SLC24A2//JUN//PRKAR2B//SLC12A5//ATXN1//SLC6A1//FOSL1//CEBPB//ITGA5//NTF3//PAIP2//KCNK10//VLDLR//KMT2B//CPEB3//GRIA1//LDLR//SLC17A7//CCND2//NHLH2//HPRT1//NRG1//NOVA1//SOBP//SNAP25//STRN//EPHA4//GLRA1//GLRB//KCNJ10//SCN1A//RNF180//NR4A3//BTG2//EN1//EPS8//NR4A2//PBX3//TMOD1//TSC1//ADCY3//NPTN//NEUROG1//WFS1//CREBRF//GNAQ//P2RY1//STAT3//DMRTA1//ARRDC3//NAPEPLD//GJA1//ADRB1// |
| GO:0035295 | tube development | Biological process | 99 | 1065 | 934 | 17653 | 1.75694121904877 | 2.32500802405414e-08 | 1.48339898742247e-06 | 7.63357554393686 | 0.105995717344754 | S1PR1//EFNA1//EPHA2//ELK3//EREG//FOXC1//GAB1//CLIC4//HOXA3//ITGA5//ITGAV//JUN//KDR//MEOX2//MMP2//TMEM100//PTEN//CCL2//SOX17//TGFB2//VEGFB//WNT7A//HAND1//HAND2//EDN1//PPP3R1//TGFBR2//ACVR1//GJC1//FOXF1//ZFPM2//QKI//GDF11//OSR1//NPNT//LHX1//RARB//TCF21//SLIT2//PKD2//SOX4//SOX11//DLC1//LRP6//PRKACB//TSC1//FZD6//NOG//GJA1//DLL1//SPRED1//SULF1//PPARG//KLF4//BAG6//EN1//BCL2L11//GATA6//ETS1//EGLN1//VASH2//F3//ITGB8//STAT3//BTG1//ADAM12//HMGA2//CIC//MAN2A1//ERRFI1//BMPR2//PKDCC//PHF14//SOS1//ITGA6//FOXF2//TNF//NKX2-3//ZEB1//NR4A3//MET//PIK3CB//MAPK1//RDH10//NFIB//KLF2//THRA//CDKN1A//AR//SOSTDC1//ESR1//ADIPOR2//KLHL3//EPHA4//EPHA7//NFIA//TBC1D20//EPN1//JAK1// |
| GO:0044093 | positive regulation of molecular function | Biological process | 144 | 1742 | 934 | 17653 | 1.56237753744865 | 2.77615667138442e-08 | 1.75468668865167e-06 | 7.55655602827563 | 0.154175588865096 | GADD45A//MINK1//TNF//MAP3K2//CRK//CRKL//MAP3K5//TAOK3//LAMTOR3//ADAM9//DUSP5//LPAR1//LPAR3//NRG1//IGF1//NTF3//PIK3CB//PRKAA1//MAPK1//MAP2K4//EREG//FBXW7//DLC1//EGLN3//F3//PMAIP1//PPARG//EFNA5//GNAQ//COPS8//MDFIC//NPNT//ESR1//MTMR9//PLEK//PCOLCE2//PKD2//BAMBI//AKTIP//CAPRIN2//PAK6//TGFB2//TGFBR2//SLK//STIM2//PLAA//CALM2//PPP1R15B//DNAJB4//PFN2//EPHA5//LRP8//RPS6KA4//RPS6KA5//FAM13A//CDC42EP3//GNB5//FZD10//ARAP2//TAGAP//TBC1D20//ADRB1//DENND1B//S1PR1//RAB3GAP1//DOCK9//RGS17//AGFG1//AGFG2//ITGA6//JUN//ARHGAP1//ARHGAP6//ERRFI1//ELMOD1//RGS3//CCL2//CCL8//SOS1//EVI5//TRIP10//ARHGAP12//USP6NL//GIT2//EPHA2//EPHA4//EPHA7//DSTYK//KDR//MET//ADCY3//PRKACB//PRKAR2B//PPP1R12A//NR4A2//CTSA//NSMAF//BCL2L11//REST//PPM1F//FOXC1//NME1//EDN1//CCNY//MAPRE3//CCNC//CCND2//CDKN1A//MMD//VLDLR//LRP6//NEUROD1//NEUROG1//NHLH2//PTEN//FOSL1//ARID5B//MTPN//AR//CLOCK//MID1IP1//TOPORS//ARRDC3//ARRDC4//PTGES3//KLF4//RHOC//EFNA1//ACSL1//PPP2CA//TBC1D12//TSC1//TBC1D17//ASPH//FMR1//UBE2I//STAT3//CNOT6//DYNLL2//GLRX//POU4F1//HMGA2//HAND2//ST18// |
| GO:0009059 | macromolecule biosynthetic process | Biological process | 369 | 5526 | 934 | 17653 | 1.26208071479888 | 3.00364696708908e-08 | 1.88089485540958e-06 | 7.52235111336395 | 0.395074946466809 | IGF1//NFIA//NFIB//RBBP7//RBBP8//RRM2//E2F1//NPAT//TSHZ1//HMG20A//KLF2//ZBTB18//CEBPB//ZNF268//TCERG1//WWP2//KLF12//OSR1//CRY2//GLIS3//ARX//DNMT3B//DR1//EDN1//EFNA1//CC2D1B//EN1//ESR1//FHL2//CPEB3//FOXF1//FOXC1//MYT1L//CIC//ZFPM2//DNAJB5//GATA6//CNOT7//HSBP1//AR//JARID2//JUN//ZNF568//MAF//MDM4//MYB//NR4A2//RLIM//KLF3//ZBTB7A//PIAS4//POU4F1//PPARG//CC2D1A//TSHZ3//ZBTB4//RARB//REST//RNF2//PRDM16//SOX17//SOX11//STAT3//ZEB1//TCF21//TFAP2A//TFAP2C//TGIF1//KLF10//TNF//UBE2I//VLDLR//WFS1//ZNF217//LRP8//BTG2//BHLHE41//TBL1XR1//NR4A3//HMGA2//ARID5B//BRMS1L//BHLHE40//RUNX3//URI1//PER2//SQSTM1//NOG//KLF4//HAND1//FNIP1//PHF14//USP3//GTF2E1//SECISBP2L//RPL15//IRF9//KLF6//CREBRF//EGR2//ELK3//ERG//FOXJ3//FOXF2//FLI1//GRHL1//HIVEP2//IRF1//LHX1//MEF2D//MEOX2//MYBL1//NEUROD1//NHLH2//OTX2//PAX9//PBX3//POU3F2//RAD21//ARID4A//SHOX//BCL11B//BMPR2//SOX4//BTF3//TEF//TGFB1I1//THRA//TMF1//DEK//FOSL1//KLF7//CBFB//FUBP3//HAND2//CLOCK//POLR3G//SRSF2//PKD2//UGP2//HS3ST5//XYLT2//HS2ST1//HS3ST3A1//HS3ST1//REV3L//GMNC//CCNE2//ESCO2//PPP2CA//BAHD1//H2AFZ//KMT2B//DPY30//TOPORS//KHDRBS3//ZMYND11//TADA1//ZNF597//CTNND2//ZNF782//NKX2-3//ZNF800//E2F5//ZNF367//EZH1//ZBTB9//KDM2A//MYCBP2//FLII//CNOT6L//EPC2//LHX6//HBP1//ATAD2//SERTAD3//MDFIC//MNX1//HMGB3//HOXA3//HOXD1//FOXN2//ZBTB41//ZBTB34//ATXN3//LHX8//NEUROG1//NFE2//NPAS2//IER5//ARID4B//BRWD1//BNC2//ZNF532//CDCA7L//PRKAA1//TRERF1//LMO3//MAPK1//SLC2A4RG//PHTF2//ZNF630//ZNF287//CNOT6//ZNF512B//ZBTB26//MIER1//RBL2//RNF4//ATXN1//DMRTA1//SMARCD2//STAT4//ZNF2//ZNF3//ZNF131//ZNF655//BRD3//TCF7L1//PCGF5//LCOR//WASL//ZBTB47//ST18//HIPK3//NUP35//ELAVL2//NR5A2//NRBF2//MLLT6//RNF141//PAK6//SNAPC1//BTG1//SNIP1//RPS6KA4//RPS6KA5//CDKN1A//CRK//EMX2//ETS1//BOLA3//NR3C2//PLEK//ZNFX1//NKX2-4//RFX7//BDP1//CDK8//E2F3//TEAD1//KAT2B//CCNC//CPEB2//CPEB1//CPEB4//MTPN//DDX6//MKNK2//ACO1//TSC1//KBTBD8//FUT9//SERP1//ST8SIA5//MAN1A1//MAN2A1//ST8SIA3//B3GNT5//B3GALT2//CCDC126//GFPT2//VEGFB//NTNG1//RECK//PIGA//PTGES3//G3BP2//NF2//EREG//SLC2A4//FAM129A//FMR1//PRR16//FXR1//ST3GAL3//MAP3K2//MAPRE3//BAMBI//RGMB//LRP6//MAP3K5//WNT7A//ACVR1//DKK3//NRG1//RASD1//TSG101//BTAF1//PPM1F//TOB1//NANOS1//CAPRIN2//OGN//CHST1//ZDHHC1//ST6GALNAC3//ST6GALNAC6//ST6GAL2//ST3GAL5//MCFD2//ZDHHC5//SLC35D1//CHST7//DSEL//SOSTDC1//TMEM100//RGMA//BMP3//TGFB2//SULF1//CTDSPL2//CMKLR1//DCP2//CCT6A//EPHA5//FBXW7//NCK1//RNF128//ERRFI1//INTS8//NABP1//ITM2B//EGLN1//FZD6//NPLOC4//ITGAV//TMEFF2//MAP2K4//S1PR1//DLL1//IL2//ITGA6//MET//PPP1R12A//OSM//P2RY1//PPP3R1//DCAF6//PAIP2//INHBB//PTEN//KDR//CCL2//NUP43//YTHDF2//QKI//EGLN3//ATG16L1//ORMDL3//RAB3GAP1//RAP2C//TOB2//PPP1R15B//DUSP1//GJA1//NSMAF//NPNT//UNK// |
| GO:0051128 | regulation of cellular component organization | Biological process | 185 | 2403 | 934 | 17653 | 1.45508915069582 | 4.34167516181392e-08 | 2.69383019443555e-06 | 7.36234267298412 | 0.198072805139186 | NANOS1//SPOCK1//TSG101//KIAA1109//URI1//FMR1//NTF3//EPS8//TWF1//STMN2//MID1IP1//CDC23//CDC14A//DLC1//CDC42EP3//WASF3//PALM2//FGD4//CRK//FGD5//LPAR1//BAMBI//KDR//RHOC//RND3//MSN//BRWD1//CCL2//WASL//EPHA2//SLIT2//CNOT6L//PAN3//CNOT6//BNIP3//TMED9//ETS1//SQSTM1//CHMP3//PTEN//SNAP25//ZNF804A//NPTN//NCK1//NME1//SERPINI1//EPHA4//MYLIP//PTPRG//TSC1//BAG5//PMEPA1//LDLRAD4//THRA//SH3D19//EFNA5//HECW2//RAB21//RUFY2//ADAM10//MTPN//IL2//DERL2//TGFB2//CDKN1A//FRZB//GJA1//SERTAD3//PPARG//PPP2CA//RBBP7//SOX17//BMPR2//CAPRIN2//BTG1//ADIPOR2//DPYSL2//PFN2//LIMA1//CFL2//PLEK//GRB2//JARID2//MET//SLAIN2//CLIP1//WNT7A//TNF//FNIP1//TBC1D12//EVI5//TBC1D17//USP6NL//NEUROG1//ATAD2//MIER1//DCP2//MAPK1//CCT6A//PMAIP1//BCL2L11//FZD10//EPHA5//RPS6KA4//RPS6KA5//PTGER4//ADAM9//IQGAP2//ARFIP1//CEP97//TMEFF2//AR//GOLGA4//NRG1//DLL1//LDLR//MTBP//EDN1//EREG//IGF1//RAD21//ESR1//MDM1//KAT2B//CD2AP//EPHA7//ULK2//LPAR3//KLF4//RGMA//NSMF//FXR1//SEMA4B//ITGAV//EFNA1//POU3F2//GPM6A//CLIC4//PPM1E//NF2//PPM1F//S1PR1//ARHGAP6//PPFIA1//KMT2B//DNMT3B//MYB//TMOD3//TMOD1//SLK//SLITRK3//FLRT1//CPEB3//C21ORF91//LRP8//SACS//TMEM135//MIEF1//DDHD1//RNF4//MOAP1//DUSP1//VLDLR//CRKL//DOCK5//DYNLL2//E2F1//PPP3R1//YWHAG//CALCOCO2//FYCO1//ASPH//RAP1B//FEZ2//FBXW7//RAB3GAP1//PLAA//ELMOD1//PRKAA1//SNIP1//JUN//FOSL1//PIK3CB//PIKFYVE//CBFB// |
| GO:2000026 | regulation of multicellular organismal development | Biological process | 160 | 2008 | 934 | 17653 | 1.50601022036053 | 4.8848744833045e-08 | 3.0033096482353e-06 | 7.31114659098483 | 0.171306209850107 | MARCH7//TGFBR2//NME1//BMPR2//HAND2//SOX17//FOXC1//BAMBI//TGFB1I1//TGFB2//EFNA1//PPP2CA//PTEN//LDLRAD4//SNAP25//ZNF804A//STMN2//NPTN//NCK1//SERPINI1//TWF1//LPAR1//EPHA4//MYLIP//PTPRG//SPOCK1//TSC1//BAG5//NF2//DLL1//MTPN//SPRED1//SULF1//PPARG//KLF4//LHX1//S1PR1//GJA1//OSR1//FAM20C//TFAP2A//ACVR1//PKDCC//DPYSL2//RAB21//KIAA1109//WNT7A//RARB//TNFRSF21//WASF3//NRG1//MAF//PTHLH//SOS1//ZEB1//OTX2//PAX9//EDN1//HMGB3//IL2//CBFB//IRF1//DUSP10//TMEM100//BTG1//GRHL1//ERRFI1//MYB//SOCS5//CDK6//ETS1//STAT3//NFE2//NR4A3//DPY30//KAT2B//KMT2B//JUN//NTF3//BCL11B//YWHAG//HMG20A//DDX6//REST//DNMT3B//MMD//NEUROD1//NEUROG1//SOX11//CEBPB//TOB2//TMEM64//POU4F1//KLF10//TNF//EPHA2//EGLN1//VASH2//F3//GAB1//GATA6//ITGB8//KDR//VEGFB//ADAM12//HMGA2//GOLGA4//LAMA3//PRKAA1//EPHA7//ULK2//EFNA5//LPAR3//RGMA//NOG//RHEB//FMR1//NSMF//HECW2//FXR1//SEMA4B//PER2//MAN2A1//POU3F2//SLIT2//CAPRIN2//SLITRK3//FLRT1//JARID2//ZFPM2//VANGL1//FZD6//AR//NTN4//ESR1//BTBD7//CPEB3//C21ORF91//LRP8//FRZB//IGF1//LDLR//ASPN//MEOX2//VLDLR//PRKACB//YTHDF2//NLN//KLF2//EPN1//ITGA5//JAK1//PLAA//SOSTDC1//LRP6//CLOCK//FBXW7//NFIB//PHF14//FNIP1// |
| GO:1901362 | organic cyclic compound biosynthetic process | Biological process | 321 | 4704 | 934 | 17653 | 1.28976136760914 | 5.30012652036649e-08 | 3.22925726641789e-06 | 7.27571376314616 | 0.343683083511777 | E2F1//NPAT//RRM2//TSHZ1//HMG20A//KLF2//ZBTB18//CEBPB//ZNF268//TCERG1//WWP2//KLF12//OSR1//CRY2//GLIS3//ARX//DNMT3B//DR1//EDN1//EFNA1//CC2D1B//EN1//ESR1//FHL2//CPEB3//FOXF1//FOXC1//MYT1L//CIC//ZFPM2//DNAJB5//GATA6//CNOT7//HSBP1//AR//JARID2//JUN//ZNF568//MAF//MDM4//MYB//NFIA//NFIB//NR4A2//RLIM//KLF3//ZBTB7A//PIAS4//POU4F1//PPARG//CC2D1A//TSHZ3//ZBTB4//RARB//RBBP7//RBBP8//REST//RNF2//PRDM16//SOX17//SOX11//STAT3//ZEB1//TCF21//TFAP2A//TFAP2C//TGIF1//KLF10//TNF//UBE2I//VLDLR//WFS1//ZNF217//LRP8//BTG2//BHLHE41//TBL1XR1//NR4A3//HMGA2//ARID5B//BRMS1L//BHLHE40//RUNX3//URI1//PER2//SQSTM1//NOG//KLF4//HAND1//FNIP1//PHF14//USP3//GTF2E1//IRF9//KLF6//CREBRF//EGR2//ELK3//ERG//FOXJ3//FOXF2//FLI1//GRHL1//HIVEP2//IRF1//LHX1//MEF2D//MEOX2//MYBL1//NEUROD1//NHLH2//OTX2//PAX9//PBX3//POU3F2//RAD21//ARID4A//SHOX//BCL11B//BMPR2//SOX4//BTF3//TEF//TGFB1I1//THRA//TMF1//DEK//FOSL1//KLF7//CBFB//FUBP3//HAND2//CLOCK//POLR3G//SRSF2//PKD2//NAGK//GFPT2//SLC35D1//UGP2//NUP35//NUP43//HPRT1//ADCY3//IMPDH1//NME1//PDE10A//CMPK1//DCTPP1//BAHD1//H2AFZ//KMT2B//DPY30//TOPORS//KHDRBS3//ZMYND11//TADA1//ZNF597//CTNND2//ZNF782//NKX2-3//ZNF800//E2F5//ZNF367//EZH1//ZBTB9//KDM2A//MYCBP2//FLII//CNOT6L//EPC2//LHX6//HBP1//ATAD2//SERTAD3//MDFIC//MNX1//HMGB3//HOXA3//HOXD1//FOXN2//ZBTB41//ZBTB34//ATXN3//LHX8//NEUROG1//NFE2//NPAS2//IER5//ARID4B//BRWD1//BNC2//ZNF532//CDCA7L//PRKAA1//TRERF1//LMO3//MAPK1//SLC2A4RG//PHTF2//ZNF630//ZNF287//CNOT6//ZNF512B//ZBTB26//MIER1//RBL2//RNF4//ATXN1//DMRTA1//SMARCD2//STAT4//ZNF2//ZNF3//ZNF131//ZNF655//BRD3//TCF7L1//PCGF5//LCOR//WASL//ZBTB47//ST18//HIPK3//ELAVL2//NR5A2//NRBF2//MLLT6//RNF141//PPP2CA//PAK6//SNAPC1//BTG1//SNIP1//RPS6KA4//RPS6KA5//CDKN1A//CRK//EMX2//ETS1//BOLA3//NR3C2//PLEK//ZNFX1//NKX2-4//RFX7//BDP1//CDK8//E2F3//TEAD1//KAT2B//CCNC//ACBD3//LBR//MSMO1//CHCHD10//PTGES3//G3BP2//PSAT1//EREG//ACOT7//PPCS//MAP3K2//MAPRE3//BAMBI//RGMB//IGF1//LRP6//MAP3K5//WNT7A//ACVR1//DKK3//NRG1//RASD1//TSG101//BTAF1//PPM1F//TOB1//SOSTDC1//TMEM100//RGMA//BMP3//TGFB2//SULF1//CTDSPL2//ADRB1//PTHLH//CALM2//EGLN1//CMKLR1//DCP2//CCT6A//EPHA5//UXS1//REV3L//INTS8//NABP1//FZD6//NPLOC4//S1PR1//DLL1//IL2//ITGA6//MET//PPP1R12A//NCK1//OSM//P2RY1//PPP3R1//DCAF6//CAPRIN2//PTEN//MTPN//EGLN3//PFKP//RAP2C//TOB2//DUSP1//GJA1//FBXW7//NPNT// |
| GO:0051246 | regulation of protein metabolic process | Biological process | 223 | 3033 | 934 | 17653 | 1.38964573135905 | 5.48223850627806e-08 | 3.3103909837463e-06 | 7.2610420742859 | 0.238758029978587 | CDKN1A//GADD45A//PTEN//CCNE2//MINK1//TNF//MAP3K2//CRK//CRKL//MAP3K5//TAOK3//LAMTOR3//ADAM9//DUSP5//LPAR1//LPAR3//NRG1//IGF1//NTF3//PIK3CB//PRKAA1//MAPK1//MAP2K4//DUSP10//SPRED1//DUSP1//DUSP2//PPP2CA//RGS3//SECISBP2L//DRD1//FNIP1//FAM129A//IL2//LRP6//PPP1R15B//SLIT2//EFNA1//EFNA5//EPHA7//FMR1//NPTN//KDR//P2RY1//AKTIP//SQSTM1//CCND2//EREG//FBXW7//ERRFI1//TSG101//SOCS5//MTPN//DDX6//MKNK2//ACO1//TSC1//KBTBD8//PPM1E//FLRT1//GNAQ//NCK1//NF2//ASPN//DUS2//YWHAG//CAMK2N2//PPM1F//DLC1//EGLN3//F3//PMAIP1//PPARG//COPS8//MDFIC//TMF1//SNIP1//CALM2//KAT2B//GDF11//INHBB//BMP3//BMPR2//TGFB2//ACVR1//SERPINI1//SPOCK1//RECK//PCOLCE2//CPEB3//SERP1//PRR16//SOX4//FXR1//TOB1//CNOT7//NANOS1//CAPRIN2//BTG2//SPOPL//JARID2//PER2//BAG5//NDFIP2//WFS1//CDK6//PKD2//JUN//RAP2C//PAK6//TGFBR2//SLK//EDN1//WNT7A//KLF4//BAG6//RNF180//ANKIB1//LONRF3//LONRF1//CRY2//PPP1R37//MASTL//URI1//MYLIP//RPS6KA4//RPS6KA5//OSM//PFN2//IFNA1//HMG20A//RNF4//PIAS4//ADCY3//PRKACB//PRKAR2B//CNOT6//STAT3//CNEP1R1//NSMF//PPP1R12A//ZNF287//RNF128//MDM4//ITM2B//ARL6IP1//BCL2L11//REST//GRB2//EPHA2//EPHA4//EPHA5//AR//ITGAV//MET//FZD10//HIPK3//AIDA//TWF1//UBXN2B//PPP4R2//IRF1//CEBPB//WWP2//GJA1//NSF//HECW2//SOX17//LATS2//IPO5//CCNY//MAPRE3//CCNC//MMD//VLDLR//ASPH//PAIP2//ZMYND11//ITGA5//LRP8//SH3D19//KLF2//LDLR//TOPORS//ARRDC3//ARRDC4//KMT2B//DNMT3B//MYB//PMEPA1//LDLRAD4//NOG//NUP35//NUP43//CNOT6L//YTHDF2//QKI//RANBP9//LMO3//NPNT//DSTYK//DNAJC27//CCL2//CCL8//HAND2//ACSL1//PLAA//CPEB2//CRTAP//RAB3GAP1//FBXL5//MSN//UBE2I//ESR1//DERL2//EDEM1//ATXN3//FRY//CTSA//CPEB1//CPEB4//UNK//ST18// |
| GO:0048729 | tissue morphogenesis | Biological process | 67 | 639 | 934 | 17653 | 1.98173504505501 | 6.44695056278022e-08 | 3.85847138549404e-06 | 7.19064566008113 | 0.0717344753747323 | EDN1//PPP3R1//TGFBR2//ACVR1//NPNT//LHX1//PKD2//TCF21//NF2//BMPR2//NR4A3//NOG//HAND1//SOX4//SOX11//DLC1//LRP6//PRKACB//TGFB2//TSC1//FZD6//GJA1//DLL1//SOX17//HAND2//ARHGAP12//TMEM100//ZFPM2//NRG1//POU4F1//S1PR1//SOS1//TFAP2A//KLF4//TGFB1I1//SOSTDC1//KRT27//ITGA5//WNT7A//FOXF2//TMEFF2//RHOC//FOXF1//MSN//HMGA2//FOXC1//CLIC4//MET//SLIT2//EGLN1//ACTC1//FRZB//VANGL1//RDH10//EPHA2//AR//ESR1//NTN4//GRB2//SULF1//BTBD7//TNF//OSR1//KLHL3//EPHA4//EPHA7//PTEN// |
| GO:0043085 | positive regulation of catalytic activity | Biological process | 121 | 1414 | 934 | 17653 | 1.61736338057177 | 6.61530251282065e-08 | 3.92449920124615e-06 | 7.17945029106156 | 0.129550321199143 | GADD45A//MINK1//TNF//MAP3K2//CRK//CRKL//MAP3K5//TAOK3//LAMTOR3//ADAM9//DUSP5//LPAR1//LPAR3//NRG1//IGF1//NTF3//PIK3CB//PRKAA1//MAPK1//MAP2K4//EREG//FBXW7//DLC1//EGLN3//F3//PMAIP1//PPARG//EFNA5//GNAQ//COPS8//MDFIC//NPNT//ESR1//MTMR9//PLEK//PCOLCE2//PKD2//PAK6//TGFB2//TGFBR2//SLK//PLAA//CALM2//PPP1R15B//DNAJB4//PFN2//FAM13A//CDC42EP3//GNB5//FZD10//ARAP2//TAGAP//TBC1D20//ADRB1//DENND1B//S1PR1//RAB3GAP1//DOCK9//RGS17//AGFG1//AGFG2//ITGA6//JUN//ARHGAP1//ARHGAP6//ERRFI1//ELMOD1//RGS3//CCL2//CCL8//SOS1//EVI5//TRIP10//ARHGAP12//USP6NL//GIT2//EPHA2//EPHA4//EPHA5//EPHA7//DSTYK//KDR//MET//ADCY3//PRKACB//PRKAR2B//PPP1R12A//BCL2L11//REST//PPM1F//EDN1//CCNY//MAPRE3//CCNC//CCND2//CDKN1A//MMD//VLDLR//MID1IP1//TOPORS//ARRDC3//ARRDC4//PTGES3//KLF4//RHOC//EFNA1//LRP8//ACSL1//PPP2CA//TBC1D12//TSC1//TBC1D17//ASPH//UBE2I//PTEN//STAT3//DYNLL2//ST18//NR4A2//CTSA//NSMAF// |
| GO:0065007 | biological regulation | Biological process | 724 | 12351 | 934 | 17653 | 1.10791920202735 | 6.9531805422387e-08 | 4.08907478323133e-06 | 7.15781649383025 | 0.775160599571734 | E2F1//USP28//CLOCK//CDKN1A//GADD45A//PTEN//CCNE2//NPAT//RRM2//TSHZ1//HMG20A//KLF2//ZBTB18//CEBPB//ZNF268//TCERG1//WWP2//KLF12//OSR1//CRY2//GLIS3//ARX//DNMT3B//DR1//EDN1//EFNA1//CC2D1B//EN1//ESR1//FHL2//CPEB3//FOXF1//FOXC1//MYT1L//CIC//ZFPM2//DNAJB5//GATA6//CNOT7//HSBP1//AR//JARID2//JUN//ZNF568//MAF//MDM4//MYB//NFIA//NFIB//NR4A2//RLIM//KLF3//ZBTB7A//PIAS4//POU4F1//PPARG//CC2D1A//TSHZ3//ZBTB4//RARB//RBBP7//RBBP8//REST//RNF2//PRDM16//SOX17//SOX11//STAT3//ZEB1//TCF21//TFAP2A//TFAP2C//TGIF1//KLF10//TNF//UBE2I//VLDLR//WFS1//ZNF217//LRP8//BTG2//BHLHE41//TBL1XR1//NR4A3//HMGA2//ARID5B//BRMS1L//BHLHE40//RUNX3//URI1//PER2//SQSTM1//NOG//KLF4//HAND1//FNIP1//PHF14//USP3//RANBP9//PPM1L//SPRED1//DUSP5//EREG//RASGEF1A//GRB2//NRG1//IL2//JAK1//MAP3K5//MET//MINK1//TAOK3//MAPK1//CCL2//SOS1//LAMTOR3//DCP2//PPP2CA//RPL15//MAP3K2//CRK//CRKL//ADAM9//LPAR1//LPAR3//IGF1//NTF3//PIK3CB//PRKAA1//MAP2K4//DUSP10//DUSP1//DUSP2//RGS3//CNOT6L//PAN3//CNOT6//RBFOX3//FMR1//MBNL1//RBM25//SRSF2//PTGES3//SNAP25//SLC17A7//GLRA1//GNAQ//SECISBP2L//NANOS1//SPOCK1//TSG101//KIAA1109//ADRB1//DRD1//ARRDC3//NKX2-3//BCL2L11//RPS6KA4//RPS6KA5//TMF1//PMAIP1//MOAP1//CAMLG//EPS15//NSF//FAM129A//LRP6//PPP1R15B//SLIT2//EFNA5//EPHA7//NPTN//KDR//P2RY1//AKTIP//CCND2//SULF1//GJA1//F3//BMPR2//VEGFB//VASH2//TSC1//NF2//CDK6//CBFB//CALM2//ARL6IP1//TGFBR2//PBX3//RDH10//THRA//PRKACB//SKP1//BAG6//MARCH7//OSM//MIA3//CCL8//NME1//IRF1//ADCY3//PRKAR2B//HAND2//S1PR1//GPR6//SPHK2//PKD2//E2F5//MASTL//CCNG2//EPS8//TWF1//COL1A2//ENPP4//DOCK9//IFNA1//NFE2//APCDD1//CTNND2//AMER2//CCNY//SOSTDC1//HBP1//DKK3//STRN//TGFB1I1//WNT7A//TCF7L1//PPP3R1//FZD6//FAM13A//CNIH1//DLC1//CDS1//CDC42EP3//GNB5//IQGAP2//RASSF8//ARAP2//TAGAP//CLCN6//DPYSL2//ELK3//STX2//ERG//EVI2A//LRCH1//CD2AP//APPL1//GLRB//RASSF3//RGMB//GRIA1//CD274//LRP12//HIVEP2//NR3C2//MPP1//PPP1R12A//RASD1//KCNK10//NDFIP2//PPP1R12C//PSD//MIER1//RHEB//SMOC1//SMOC2//BCL11B//SLC20A1//RASL11B//DEK//PDCD1LG2//NSMAF//PPFIA1//RHPN2//OR6A2//ARRDC4//TRIP10//PLAA//ARHGAP12//CXCL14//FEZ2//CD69//ULK2//PTPRG//AKAP11//DGKH//DCX//PIKFYVE//DSTYK//LATS2//MKNK2//ARHGEF4//TREM1//ARHGEF28//STK33//DGKE//STK17B//PALM2//FBXW7//ERRFI1//SOCS5//NUP35//NUP43//NLN//DNAJC16//GLRX//NHLRC2//TMX3//ESCO2//BAHD1//H2AFZ//KMT2B//DPY30//ARID4B//ARID4A//HIPK3//KHDRBS3//ZMYND11//ZNF597//ZNF782//ZNF800//ELAVL2//ZBTB9//KDM2A//MYCBP2//FLII//NR5A2//GTF2E1//SERTAD3//NRBF2//HMGB3//ZBTB41//ZBTB34//ATXN3//MLLT6//RNF141//BNC2//ZNF532//CDCA7L//LMO3//SLC2A4RG//PAK6//PHTF2//ZNF630//ZNF512B//ZBTB26//SNAPC1//SOX4//BTF3//BTG1//ZNF2//ZNF3//ZNF131//ZNF655//SNIP1//WASL//ZBTB47//IRF9//TADA1//ZNF367//EMX2//ETS1//FOXJ3//FOXF2//FLI1//EPC2//LHX6//ATAD2//GRHL1//MNX1//HOXA3//HOXD1//FOXN2//BOLA3//LHX8//MYBL1//NEUROG1//NHLH2//PLEK//BRWD1//POU3F2//TRERF1//ZNFX1//RAD21//RBL2//DMRTA1//NKX2-4//RFX7//SMARCD2//TEF//BRD3//FOSL1//LCOR//KLF7//POLR3G//BDP1//MTPN//DDX6//ACO1//KBTBD8//PPM1E//FLRT1//NCK1//ASPN//DUS2//YWHAG//CAMK2N2//PPM1F//SLC25A32//SLC12A5//SLC24A2//SLC24A3//STIM2//FBXL5//EGLN1//CYBRD1//EGLN3//CHRM2//STMN2//MID1IP1//CLIC4//MTBP//RRAGD//TGFB2//KAT2B//CDC23//NABP1//CDC14A//ADAM10//GAB1//ACVR1//ZFYVE9//PDE10A//RAPGEF4//FZD10//CHRM5//FGD4//CMKLR1//GPR180//FRZB//RHOC//GPRC5C//PTGER4//PTHLH//SOS2//ITGAV//DLL1//EPN1//TMEM100//TMEM17//DZIP1//ITGA6//ITGA5//ITGB8//NKIRAS1//COPS8//G3BP2//MDFIC//STAT4//NEUROD1//RGL1//RAB38//RND3//ARHGAP1//RALGPS2//DOCK5//ARHGAP6//SLC32A1//CDK8//CDK19//SLK//TRPC3//MARCKS//TOPORS//EPHA2//EPHA4//EPHA5//ZFAND6//BNIP3//ACTC1//PLEKHA1//UGT1A1//GIT2//E2F3//BAMBI//DERL2//TOB1//GDF11//TOB2//BTG3//TES//ASPH//ST18//PIK3R3//WASF3//FGD5//MSN//HS3ST5//PLSCR4//ESRP1//OTX2//NPNT//ADIPOR2//PIGA//RGS17//NEDD1//HAUS8//CEP41//LHX1//INHBB//OGN//BMP3//TTR//MTMR9//PPP4R2//RAB3GAP1//LDLR//FUBP3//ZNF804A//QKI//PFN2//TMED9//LDLRAD4//C5ORF30//BCAT2//CHMP3//CNEP1R1//PPP1R37//PLEKHF2//SERPINI1//RECK//PCOLCE2//SLC41A1//MYLIP//BAG5//PMEPA1//SLC6A1//SERP1//PRR16//FXR1//SESN3//RAB12//SESN1//SNX5//CPEB2//IPO5//TNFRSF21//ANP32E//KLF6//EGR2//MAPRE3//NPAS2//ZNF287//RNF4//TEAD1//PAX9//ATXN1//BTAF1//CRTAP//OTUD3//MORC3//USP27X//CDC37L1//CCT6A//CAPRIN2//RAB21//SYT6//TRPV6//ACSL1//ACOX1//UGT1A9//MMP2//SCN1A//SH3D19//DENND1B//HECW2//RUFY2//LAMA3//SPOPL//SHISA2//SEMA4B//PTP4A1//TMEFF2//RAP2C//FAM20C//PKDCC//RGMA//CTDSPL2//LIMA1//CFL2//CREBRF//SLAIN2//CLIP1//TBC1D12//EVI5//TBC1D17//USP6NL//CTSA//ELMOD1//RNF128//AGFG1//AGFG2//USP46//RNF180//ANKIB1//LONRF3//LONRF1//KIF13A//KIF23//NPLOC4//RAB30//RAB9B//DNAJC27//RAB23//RAB22A//RAB28//RAP1B//CNN3//PXK//DNAJB4//STX6//MMD//MEMO1//FRMD6//SBF2//TBC1D20//UBA5//ARFIP1//KCNA4//KCNJ3//KCNJ10//HCN2//CPEB4//NSMF//GSTA2//SYBU//DNAJB9//EDEM1//ZMAT3//IER5//KCTD20//BTBD10//SLC44A1//UGT1A8//UGT1A7//UGT1A3//SLC2A4//ITM2B//LYPLA1//UBE2Z//FRY//PGRMC2//TMEM64//FASTK//AHNAK//YTHDF2//AIDA//UBXN2B//PEX5L//PLEKHB2//ZADH2//CCDC71L//CEP97//CCNC//ADAM12//GOLGA4//EZH1//MEF2D//MEOX2//DCAF6//SHOX//PCGF5//PAIP2//RAET1E//HPRT1//MDM1//KLHL3//TMOD3//MAN2A1//SLC17A6//NOVA1//SLC4A4//GPM6A//TMOD1//SLITRK3//STEAP2//VANGL1//NTN4//BTBD7//IDH1//C21ORF91//DENND5B//TRAPPC8//FIBIN//XKR8//MCOLN2//GJC1//DSC2//SACS//TMEM135//MIEF1//DDHD1//ORMDL3//SGPP1//FAM155A//LYPD6//DYNLL2//CALCOCO2//FYCO1//SERINC3//NEFH//NAPEPLD//MTRR//SESTD1//CENPQ//LHFPL2//KLHL15//IER3IP1//CPEB1//UNK//CHCHD10// |
| GO:0048513 | animal organ development | Biological process | 246 | 3428 | 934 | 17653 | 1.35633178334862 | 7.18288067540748e-08 | 4.18774327653283e-06 | 7.14370134816263 | 0.26338329764454 | THRA//WNT7A//CEBPB//FOXC1//INHBB//DMRTA1//IMMP2L//KMT2B//ESR1//EREG//PLEKHA1//SMOC1//TGFB2//GDF11//RDH10//OSR1//NPNT//LHX1//RARB//TCF21//SLIT2//PKD2//STAT3//SEMA4B//ACVR1//BCL2L11//SULF1//SOX11//TFAP2A//TSC1//WFS1//BAG6//ARID5B//FOXF2//PPP3R1//HMGA2//NOG//GATA6//JARID2//JUN//MAN2A1//MET//UGT1A1//PPARG//BMPR2//APCDD1//SOS1//FZD6//GJA1//DLL1//SOX17//TGFBR2//HAND1//HAND2//MEF2D//NFIB//RUNX3//ESCO2//PLEK//RBM47//REST//NKX2-3//FNIP1//SOX4//NME1//DLC1//ZFPM2//NRG1//EFNA1//ERG//FOXF1//TMEM100//POU4F1//S1PR1//CDK6//NEUROD1//PALLD//BCL11B//LRP6//GPM6A//SLC17A7//CRKL//E2F5//STX2//FLI1//CCL2//BHLHE41//DPYSL2//EGR2//EPHA7//RAB3GAP1//CIC//SPHK2//DSCAML1//PTPRG//ATXN1//B3GNT5//EDN1//GNAQ//PTEN//ADIPOR2//KAT2B//IGF1//FXR1//SGCE//ZBTB18//CFL2//MEOX2//RNF38//TFAP2C//IDH1//LHX8//HOXA3//BAMBI//TGFB1I1//PPP2CA//LDLRAD4//TOPORS//FJX1//KBTBD8//FRZB//MTPN//PBX3//VLDLR//MNX1//LRP8//DRD1//EMX2//BTG2//LPAR1//EN1//HPRT1//ARX//CALM2//SLC32A1//EPHA5//EZH1//NF2//NR4A3//LHX6//POU3F2//ETS1//C21ORF91//NR4A2//TACC2//BNIP3//MSN//MKNK2//CBFB//KLF6//IFNA1//DNAJB9//EPHA2//CLIC4//LATS2//TSG101//ADAM9//IL2//ASPN//PTHLH//KLF10//PKDCC//OSTM1//TNF//DKK3//FAM20C//AR//MAPK1//E2F1//OTX2//SOSTDC1//KRT27//KLF4//CDKN1A//DSC2//MAF//LRIG1//ZEB1//TMF1//NEUROG1//KDR//FOXN2//PTGER4//SOBP//TSHZ1//COL1A2//PAX9//KLF2//IRF1//BNC2//PRDM16//ITGA6//ARRDC3//ERRFI1//HMGB3//DUSP10//GRHL1//MYB//SOCS5//NFE2//DPY30//TOB2//TMEM64//GJB2//PHF14//EPN1//TEAD1//PRKAA1//SERP1//GJC1//TMOD3//ARID4A//CXCL14//ITGB8//BMP3//ACTC1//EGLN1//FHL2//MDM1//VANGL1//MFAP5//MMP2//TBL1XR1//NTN4//ZNF568//GRB2//FOSL1//BTBD7//VASH2//VEGFB//SPRED1//MAP2K4//NR5A2//TMOD1//TBC1D20//KLHL3//EPHA4//NFIA//RAB23//ARID4B//NANOS1//YTHDF2//NLN//RHEB//CLOCK//FBXW7// |
| GO:0031175 | neuron projection development | Biological process | 88 | 936 | 934 | 17653 | 1.77696334120317 | 9.00302751800647e-08 | 5.20405770122032e-06 | 7.04561142242017 | 0.0942184154175589 | NTNG1//SLITRK3//NEFH//OGN//POU4F1//DSCAML1//WNT7A//SLIT2//RANBP9//ARX//DPYSL2//EFNA1//EFNA5//EPHA5//GAB1//GRB2//KIF5A//OTX2//PIK3CB//ENAH//MAPK1//NTN4//SOS1//VLDLR//NR4A3//KLF7//RPS6KA5//FEZ2//EGR2//EPHA4//LHX1//NOG//PTEN//SNAP25//ZNF804A//STMN2//NPTN//NCK1//NME1//SERPINI1//TWF1//LPAR1//MYLIP//PTPRG//SPOCK1//TSC1//BAG5//FLRT1//STRN//NR4A2//NFIB//RAB21//JUN//EPHA7//GOLGA4//NRG1//ULK2//LPAR3//KLF4//RGMA//GJA1//GPM6A//MNX1//NTF3//MINK1//HPRT1//FMR1//NSMF//HECW2//FXR1//BMPR2//SEMA4B//POU3F2//CAPRIN2//SLC12A5//CTNND2//WASL//CPEB3//IL2//C21ORF91//LRP8//NEFM//BCL11B//PLAA//FRY//LRP12//PSD//BTG2// |
| GO:0044267 | cellular protein metabolic process | Biological process | 359 | 5421 | 934 | 17653 | 1.25166090155384 | 1.44612937447e-07 | 8.28828216910221e-06 | 6.83979285219706 | 0.384368308351178 | CDKN1A//GADD45A//PTEN//CCNE2//RANBP9//PPM1L//SPRED1//DUSP5//EREG//RASGEF1A//GRB2//NRG1//IL2//JAK1//MAP3K5//MET//MINK1//TAOK3//MAPK1//CCL2//SOS1//TNF//LAMTOR3//MAP3K2//CRK//CRKL//ADAM9//LPAR1//LPAR3//IGF1//NTF3//PIK3CB//PRKAA1//MAP2K4//DUSP10//DUSP1//DUSP2//PPP2CA//RGS3//TOPORS//WWP2//HECTD2//RNF145//ZNRF2//FBXL5//RNF180//MYLIP//RLIM//ANKIB1//UBE2W//FBXW7//HECW2//SKP1//THOP1//LONRF3//FBXO30//CBFB//UBE3B//LONRF1//ZFAND4//COPS8//SECISBP2L//DRD1//FNIP1//FAM129A//LRP6//PPP1R15B//SLIT2//EFNA1//EFNA5//EPHA7//FMR1//NPTN//KDR//P2RY1//AKTIP//SQSTM1//CCND2//LYPLA1//RPL15//ERRFI1//TSG101//SOCS5//TRIM3//KLHL2//RNF38//MYCBP2//FBXO33//KLHL3//SPOPL//MDM4//DCAF8//DCAF6//FEM1C//KLHL14//MARCH4//RNF4//MARCH7//UBE2Z//RNF128//KLHL15//KBTBD8//ARRDC4//CPEB2//CPEB3//CPEB1//SOX11//CPEB4//MTPN//DDX6//MKNK2//ACO1//TSC1//PPP4R2//SERP1//UEVLD//RIMKLB//UBE2I//IMMP2L//HIPK3//ADAM10//CDK6//CDK8//FASTK//PDIK1L//ERG//CDK19//MORC3//MMD//PAN3//DSTYK//LATS2//CDK17//PRKACB//FAM20C//CCL8//TGFB2//TGFBR2//RUNX3//CCNC//RPS6KA4//ACVR1//RPS6KA5//STK17B//PPM1E//FLRT1//GNAQ//NCK1//NF2//ASPN//DUS2//YWHAG//CAMK2N2//PPM1F//PTPN21//PPP1R12A//CTDSPL2//PPP3R1//PPP6C//PTPN4//KAT2B//CLOCK//NAA30//HS3ST5//PCMTD1//BTG1//BTG2//ASPH//FUT9//ST8SIA5//MAN1A1//MAN2A1//ST8SIA3//B3GNT5//B3GALT2//CCDC126//GFPT2//VEGFB//NTNG1//RECK//PTAR1//PIGA//CTSA//USP24//USP27X//ATXN3//NPLOC4//USP28//USP46//BAG6//USP45//CDC23//USP3//DLC1//EGLN3//F3//PMAIP1//PPARG//DLL1//PTGER4//MDFIC//TMF1//CALM2//GDF11//INHBB//BMP3//BMPR2//SERPINI1//SPOCK1//PCOLCE2//PRR16//SOX4//FXR1//ST3GAL3//EPC2//ARID4B//MIER1//ARID4A//RBBP7//TBL1XR1//BRMS1L//ARID5B//DMWD//ESR1//OTUD3//AR//WDR20//JOSD1//NUP35//EGR2//NUP43//PIAS4//TOB1//CNOT7//NANOS1//CAPRIN2//VKORC1L1//CEP41//STK33//MASTL//OSR1//EPHA2//EPHA4//EPHA5//PKDCC//TMX3//ZDHHC1//ST6GALNAC3//ST6GALNAC6//ST6GAL2//ST3GAL5//MCFD2//CRY2//ZDHHC5//CRTAP//EGLN1//PAK6//SLK//UBXN4//DNAJB9//DERL2//JKAMP//WFS1//EDEM1//TGFB1I1//JARID2//PER2//BAG5//NDFIP2//PKD2//JUN//RAP2C//EDN1//WNT7A//KLF4//PPP1R37//URI1//VLDLR//OSM//PFN2//IFNA1//HMG20A//ADCY3//PRKAR2B//ESCO2//PRDM16//CNOT6//STAT3//CNEP1R1//NSMF//PTPDC1//PTPRG//PTP4A1//CDC14A//RNF2//BNIP3//HMGA2//PCGF5//DNAJB12//ARL6IP1//UBXN2B//CD2AP//PLAA//VPS37A//BCL2L11//REST//ITGAV//FZD10//AIDA//TWF1//CKAP4//RAB38//MIA3//ANO8//TADA1//DR1//RAB12//IPO5//CCNY//MAPRE3//PAIP2//ZMYND11//ULK2//ITGA5//LRP8//SH3D19//ARRDC3//DPY30//KMT2B//DNMT3B//MYB//RNF141//PMEPA1//LDLRAD4//NOG//CNOT6L//YTHDF2//QKI//ATG16L1//LMO3//NPNT//DNAJC27//HAND2//KDM2A//EZH1//UBA5//ACSL1//GLRX//RAB3GAP1//MSN//FRY//LDLR//UNK//ST18//GATA6//MMP2//TTR//ITM2B// |
| GO:0031400 | negative regulation of protein modification process | Biological process | 63 | 600 | 934 | 17653 | 1.98454496788009 | 1.53165496028803e-07 | 8.70469117346886e-06 | 6.8148390582805 | 0.0674518201284797 | DUSP10//SPRED1//DUSP1//DUSP2//DUSP5//PPP2CA//RGS3//FAM129A//IL2//LRP6//PTEN//PPP1R15B//SLIT2//ERRFI1//TSG101//SOCS5//GADD45A//PPM1E//FLRT1//GNAQ//NCK1//NF2//TAOK3//ASPN//DUS2//YWHAG//CAMK2N2//PPM1F//CALM2//JARID2//SPOPL//SOX4//PER2//BAG5//CDK6//JUN//CRY2//PPP1R37//MASTL//URI1//HMG20A//RNF4//TNF//EFNA1//HIPK3//AIDA//CDKN1A//LATS2//IPO5//KAT2B//ZMYND11//NTF3//DNMT3B//PMEPA1//LDLRAD4//NOG//RANBP9//LMO3//KLF4//PLAA//CRTAP//FRY//PRKAR2B// |
| GO:0009725 | response to hormone | Biological process | 90 | 977 | 934 | 17653 | 1.74108346355908 | 1.60447353046056e-07 | 9.04254540542064e-06 | 6.79466744321151 | 0.0963597430406852 | THRA//CHRM2//CHRM5//GNAQ//GAB1//APPL1//GRB2//SOS1//PIK3R3//NR5A2//LATS2//PPARG//RARB//ADIPOR2//ESR1//FHL2//AR//PMEPA1//RNF4//TGFB1I1//DNMT3B//DUSP1//ETS1//GJB2//PTEN//RBBP8//SLC6A1//STAT3//WNT7A//TGFB2//FOSL1//SESN3//EGR2//IGF1//TSC1//CPEB2//INHBB//ERRFI1//CPEB1//SLC2A4//YWHAG//KAT2B//JUN//SLIT2//UBA5//CBFB//ACSL1//EDN1//NME1//PGRMC2//NR3C2//NR4A2//NR4A3//EREG//GJA1//LRP6//BTG1//BTG2//NCK1//IDH1//RBBP7//TGFBR2//TNF//ADAM9//CDKN1A//MAPK1//TCF21//CRK//NSMF//GATA6//EFNA5//ADCY3//PRKACB//PRKAR2B//PRKAA1//PTGER4//URI1//BCL2L11//UGT1A1//REST//H2AFZ//POU4F1//MSN//FIBIN//RAP1B//CREBRF//CRY2//CLOCK//LMO3//STEAP2// |
| GO:0052695 | cellular glucuronidation | Biological process | 9 | 19 | 934 | 17653 | 8.9528344415643 | 1.78631347639646e-07 | 9.98416367013988e-06 | 6.74804232533385 | 0.00963597430406852 | UGT1A10//UGT1A8//UGT1A7//UGT1A6//UGT1A5//UGT1A9//UGT1A4//UGT1A1//UGT1A3// |
| GO:0032870 | cellular response to hormone stimulus | Biological process | 70 | 698 | 934 | 17653 | 1.89545842204402 | 1.84214764701564e-07 | 1.02118397842351e-05 | 6.73467556430507 | 0.0749464668094218 | THRA//CHRM2//CHRM5//GNAQ//GAB1//APPL1//GRB2//SOS1//PIK3R3//NR5A2//LATS2//PPARG//RARB//ADIPOR2//ESR1//FHL2//AR//PMEPA1//RNF4//TGFB1I1//CPEB2//INHBB//ERRFI1//PTEN//CPEB1//SLC2A4//YWHAG//KAT2B//UBA5//CBFB//STAT3//ACSL1//PGRMC2//NR3C2//NR4A2//NR4A3//SESN3//NCK1//TSC1//IGF1//MAPK1//TCF21//NSMF//GATA6//EFNA5//GJA1//EDN1//ADCY3//GJB2//PRKACB//PRKAR2B//PRKAA1//PTGER4//URI1//BCL2L11//UGT1A1//REST//H2AFZ//POU4F1//MSN//DNMT3B//RAP1B//CREBRF//CRK//CRY2//CLOCK//LMO3//DUSP1//JUN//SLIT2// |
| GO:0045859 | regulation of protein kinase activity | Biological process | 77 | 797 | 934 | 17653 | 1.8260137722025 | 2.03647152956493e-07 | 1.11972820767867e-05 | 6.69112165709248 | 0.082441113490364 | CDKN1A//GADD45A//PTEN//CCNE2//MINK1//TNF//MAP3K2//CRK//CRKL//MAP3K5//TAOK3//LAMTOR3//ADAM9//DUSP5//LPAR1//LPAR3//NRG1//IGF1//NTF3//PIK3CB//PRKAA1//MAPK1//MAP2K4//DUSP10//SPRED1//DUSP1//DUSP2//PPP2CA//RGS3//EREG//FBXW7//ERRFI1//TSG101//SOCS5//PPM1E//FLRT1//GNAQ//LRP6//NCK1//NF2//ASPN//DUS2//YWHAG//CAMK2N2//PPM1F//EFNA5//COPS8//MDFIC//CDK6//PKD2//PAK6//TGFB2//TGFBR2//SLK//ADCY3//PRKACB//PRKAR2B//EDN1//FZD10//EPHA4//HIPK3//AIDA//LATS2//IPO5//KAT2B//CCNY//MAPRE3//CCNC//CCND2//MMD//VLDLR//RAP2C//EFNA1//LRP8//ACSL1//CALM2//TSC1// |
| GO:0000904 | cell morphogenesis involved in differentiation | Biological process | 70 | 701 | 934 | 17653 | 1.88734661709946 | 2.16923419881916e-07 | 1.18275149916563e-05 | 6.66369355736153 | 0.0749464668094218 | LATS2//SOX17//CLIC4//MET//PALLD//AR//BCL11B//FRMD6//NTNG1//SLITRK3//NEFH//OGN//POU4F1//DSCAML1//WNT7A//SLIT2//RANBP9//ARX//DPYSL2//EFNA1//EFNA5//EPHA5//GAB1//GRB2//KIF5A//OTX2//PIK3CB//ENAH//MAPK1//NTN4//SOS1//VLDLR//NR4A3//KLF7//RPS6KA5//FEZ2//EGR2//EPHA4//LHX1//NOG//NR4A2//PTEN//NFIB//RAB21//EPHA7//ITGAV//GOLGA4//NRG1//UNK//ULK2//LPAR3//HPRT1//MINK1//FMR1//NSMF//HECW2//FXR1//BMPR2//SEMA4B//POU3F2//CAPRIN2//CTNND2//WASL//LRP8//TBC1D20//EPHA2//YTHDF2//CRK//CRKL//DOCK5// |
| GO:0016043 | cellular component organization | Biological process | 405 | 6269 | 934 | 17653 | 1.22103580276559 | 2.18607034445814e-07 | 1.18275149916563e-05 | 6.66033586721776 | 0.433618843683084 | KIF23//UBXN2B//TRAPPC8//RAB23//ATG16L1//ULK2//WASL//TACC2//DYNC1LI2//ATXN3//NEFH//MAP7D1//MIEF1//BNIP3//SQSTM1//COPS8//PTGES3//CCNE2//FRY//KLF2//CDH20//NRG1//TGFB2//GTF2E1//NANOS1//SPOCK1//TSG101//KIAA1109//URI1//CLIP1//TBC1D20//AGFG1//TMF1//LATS2//SOX17//JUN//PMAIP1//BCL2L11//MOAP1//CLIC4//MET//FMR1//NTF3//GRHL1//PALLD//AR//BCL11B//FRMD6//NAP1L3//ANP32E//EPS8//RHOC//RND3//MARCKS//MYO1B//LIMA1//TWF1//CFL2//CORO2B//DLC1//FGD4//FGD5//FLII//NF2//PFN2//PAK6//TRIP10//WASF3//SH3D19//DPYSL2//MSN//BRWD1//CCL2//CCDC6//MAN2A1//STX6//SYT6//STX2//SNAP25//ZFAND6//HMG20A//ZMYND11//ATAD2//H2AFZ//JARID2//RSBN1//RBL2//BAG6//BRD3//HMGA2//TCF7L1//NFE2//SMARCD2//ESR1//HMGB3//MYB//DEK//KAT2B//BAHD1//KMT2B//DPY30//MTX3//IMMP2L//CLCN6//SLC12A5//ARHGAP12//CD2AP//NCK1//REEP3//NUP35//PIKFYVE//RAB38//CHCHD10//SERP1//PALM2//TMOD3//TMOD1//STMN2//RANBP9//SLAIN2//VBP1//MID1IP1//GOLGA8A//RAB30//TMED9//NPLOC4//BAG5//USP6NL//TMEM135//RAB22A//AKTIP//FAM160A2//GJC1//ITGA6//ITGA5//DYNLL2//NUP43//CENPQ//FBXW7//RAD21//CENPO//CNEP1R1//CDC23//PPP2CA//CDC14A//GADD45A//PPP1R12A//HAUS8//MYBL1//SYCP3//EREG//MASTL//EFNA5//ZFYVE9//SOS1//FOSL1//NTNG1//SLITRK3//OGN//POU4F1//DSCAML1//WNT7A//SLIT2//ARX//EFNA1//EPHA5//GAB1//GRB2//KIF5A//OTX2//PIK3CB//ENAH//MAPK1//NTN4//VLDLR//NR4A3//KLF7//RPS6KA5//FEZ2//DRD1//GPM6A//PTEN//EGR2//EPHA4//LHX1//NOG//CDC42EP3//CRK//LPAR1//BAMBI//KDR//MTPN//PLSCR4//ARHGAP6//PTPDC1//TMEM17//DZIP1//GMNC//C5ORF30//CEP41//SKP1//FCHO2//EPHA2//CNOT6L//PAN3//CNOT6//ETS1//CHMP3//ZNF804A//NPTN//NME1//SERPINI1//MYLIP//PTPRG//TSC1//PMEPA1//LDLRAD4//EPS15//SNX5//SNX2//GJA1//GJB2//FLRT1//STRN//PEX5L//RPS6KA4//EPC2//CLOCK//ARID4B//MIER1//ARID4A//RBBP7//TBL1XR1//BRMS1L//PPM1F//ARID5B//USP3//THRA//NR4A2//NFIB//ADAM10//LAMA3//MMP2//E2F5//PLEK//S1PR1//ARHGEF4//HECW2//RAB21//RUFY2//COL1A2//HAPLN1//FOXF1//FOXF2//NPNT//ITGAV//ITGB8//SMOC2//TNF//TTR//ADAM12//MFAP5//RECK//FOXC1//ACTC1//IL2//DERL2//CDKN1A//FRZB//SERTAD3//PPARG//BMPR2//CAPRIN2//BTG1//ADIPOR2//FAM118B//TDRD5//CNN3//SLK//LRP12//PSD//BTG2//EPHA7//FNIP1//TBC1D12//EVI5//TBC1D17//PLEKHA1//MINK1//NEUROG1//LPAR3//DCP2//CCT6A//KIF13A//SULF1//FZD10//PTGER4//NEFM//DDX6//CNOT7//G3BP2//ADAM9//IQGAP2//ARFIP1//CADM2//RASSF8//PRKACB//RAB3GAP1//DNAJB12//PRDM16//PRKAA1//MIA3//NSF//RNF2//TOPORS//VPS37A//PCGF5//APOO//GLRA1//KCNA4//SLC6A1//XKR8//TADA1//DR1//VASH2//CEP97//TMEFF2//GOLGA4//EDN1//PRR16//DLL1//LDLR//MTBP//IGF1//MDM1//STAT3//CNIH1//SEC23A//GRIA1//SAR1B//PPP6C//MCFD2//UNK//KLF4//RGMA//MNX1//HPRT1//NSMF//FXR1//SEMA4B//POU3F2//CTNND2//UXS1//SBF2//KHDRBS3//DGKH//MCOLN2//AHNAK//PKD2//DCTPP1//GLRB//MZT1//PPM1E//PPFIA1//DNMT3B//NFIA//SYBU//CPEB3//C21ORF91//LRP8//CHRM2//NECAP1//EPN1//YWHAG//SGCE//MDM4//TEAD1//KDM2A//EZH1//BDP1//PER2//REST//RTN3//ARL6IP1//SACS//DDHD1//RNF4//DUSP1//SLC17A7//NEDD1//PRKAR2B//YTHDF2//CRKL//DOCK5//E2F1//PPP3R1//CALCOCO2//FYCO1//ASPH//RAP1B//PLAA//ELMOD1//SNIP1//LRP6//CBFB// |
| GO:0071229 | cellular response to acid chemical | Biological process | 30 | 200 | 934 | 17653 | 2.83506423982869 | 2.34033360427466e-07 | 1.25616477505631e-05 | 6.63072223140654 | 0.0321199143468951 | CEBPB//COL1A2//SESN3//CPEB3//NSMF//SESN1//GLRA1//IPO5//MMP2//RRAGD//CPEB1//ZEB1//TNF//CPEB4//LAMTOR3//FZD10//OSR1//MYB//PPARG//RARB//KLF4//PRKAA1//PTGER4//E2F1//EDN1//LDLR//NME1//PKD2//KLF2//KDR// |
| GO:0008285 | negative regulation of cell proliferation | Biological process | 72 | 732 | 934 | 17653 | 1.85905851792045 | 2.59875104906964e-07 | 1.38076569620977e-05 | 6.58523532234347 | 0.0770877944325482 | SULF1//GJA1//TNF//BMPR2//TNFRSF21//CEBPB//CD274//MARCH7//PDCD1LG2//MORC3//OGN//PPARG//KLF4//IL2//SOX11//CDK6//DUSP10//EREG//AR//PTEN//TGFB2//RUNX3//JARID2//TGFBR2//NOG//E2F1//E2F3//PER2//CCL8//BTG2//FBXW7//NFIB//PHF14//TOB1//GDF11//CDKN1A//DLC1//TOB2//ZNF268//COPS8//BTG3//ETS1//FRZB//TES//MTBP//DLL1//CNOT7//IRF1//JUN//MDM4//ASPH//NF2//NME1//OSM//PKD2//PTHLH//RARB//REST//BCL11B//SOX4//STAT3//STRN//ZEB1//BTG1//TFAP2A//TGFB1I1//KLF10//TSC1//TSG101//FOSL1//KAT2B//ST18// |
| GO:0035239 | tube morphogenesis | Biological process | 83 | 887 | 934 | 17653 | 1.76858573397807 | 2.61330783845706e-07 | 1.38076569620977e-05 | 6.58280942889929 | 0.088865096359743 | S1PR1//EFNA1//EPHA2//ELK3//EREG//FOXC1//GAB1//CLIC4//HOXA3//ITGA5//ITGAV//JUN//KDR//MEOX2//MMP2//TMEM100//PTEN//CCL2//SOX17//TGFB2//VEGFB//WNT7A//HAND1//HAND2//EDN1//PPP3R1//TGFBR2//ACVR1//GJC1//FOXF1//ZFPM2//QKI//NPNT//LHX1//PKD2//TCF21//SOX4//SOX11//DLC1//LRP6//PRKACB//TSC1//FZD6//NOG//GJA1//DLL1//SLIT2//SPRED1//SULF1//PPARG//KLF4//BCL2L11//ETS1//EGLN1//VASH2//F3//GATA6//ITGB8//STAT3//BTG1//ADAM12//HMGA2//SOS1//NKX2-3//ZEB1//NR4A3//MET//PIK3CB//RDH10//AR//SOSTDC1//NFIB//ESR1//ADIPOR2//TNF//BMPR2//KLHL3//OSR1//EPHA4//EPHA7//KLF2//EPN1//JAK1// |
| GO:1901576 | organic substance biosynthetic process | Biological process | 426 | 6665 | 934 | 17653 | 1.20803937601103 | 2.82652518155192e-07 | 1.48184417076245e-05 | 6.54874714105108 | 0.45610278372591 | IGF1//NFIA//NFIB//RBBP7//RBBP8//RRM2//E2F1//NPAT//TSHZ1//HMG20A//KLF2//ZBTB18//CEBPB//ZNF268//TCERG1//WWP2//KLF12//OSR1//CRY2//GLIS3//ARX//DNMT3B//DR1//EDN1//EFNA1//CC2D1B//EN1//ESR1//FHL2//CPEB3//FOXF1//FOXC1//MYT1L//CIC//ZFPM2//DNAJB5//GATA6//CNOT7//HSBP1//AR//JARID2//JUN//ZNF568//MAF//MDM4//MYB//NR4A2//RLIM//KLF3//ZBTB7A//PIAS4//POU4F1//PPARG//CC2D1A//TSHZ3//ZBTB4//RARB//REST//RNF2//PRDM16//SOX17//SOX11//STAT3//ZEB1//TCF21//TFAP2A//TFAP2C//TGIF1//KLF10//TNF//UBE2I//VLDLR//WFS1//ZNF217//LRP8//BTG2//BHLHE41//TBL1XR1//NR4A3//HMGA2//ARID5B//BRMS1L//BHLHE40//RUNX3//URI1//PER2//SQSTM1//NOG//KLF4//HAND1//FNIP1//PHF14//USP3//GTF2E1//SECISBP2L//PTGES3//ST6GALNAC3//ST6GALNAC6//ST8SIA3//ST3GAL5//RDH10//RPL15//IRF9//KLF6//CREBRF//EGR2//ELK3//ERG//FOXJ3//FOXF2//FLI1//GRHL1//HIVEP2//IRF1//LHX1//MEF2D//MEOX2//MYBL1//NEUROD1//NHLH2//OTX2//PAX9//PBX3//POU3F2//RAD21//ARID4A//SHOX//BCL11B//BMPR2//SOX4//BTF3//TEF//TGFB1I1//THRA//TMF1//DEK//FOSL1//KLF7//CBFB//FUBP3//HAND2//CLOCK//POLR3G//SRSF2//PKD2//UGP2//HS3ST5//XYLT2//HS2ST1//HS3ST3A1//HS3ST1//NAGK//GFPT2//SLC35D1//NUP35//NUP43//NLN//HPRT1//ADCY3//IMPDH1//NME1//PDE10A//CMPK1//DCTPP1//REV3L//GMNC//CCNE2//ESCO2//PPP2CA//BAHD1//H2AFZ//KMT2B//DPY30//TOPORS//KHDRBS3//ZMYND11//TADA1//ZNF597//CTNND2//ZNF782//NKX2-3//ZNF800//E2F5//ZNF367//EZH1//ZBTB9//KDM2A//MYCBP2//FLII//CNOT6L//EPC2//LHX6//HBP1//ATAD2//SERTAD3//MDFIC//MNX1//HMGB3//HOXA3//HOXD1//FOXN2//ZBTB41//ZBTB34//ATXN3//LHX8//NEUROG1//NFE2//NPAS2//IER5//ARID4B//BRWD1//BNC2//ZNF532//CDCA7L//PRKAA1//TRERF1//LMO3//MAPK1//SLC2A4RG//PHTF2//ZNF630//ZNF287//CNOT6//ZNF512B//ZBTB26//MIER1//RBL2//RNF4//ATXN1//DMRTA1//SMARCD2//STAT4//ZNF2//ZNF3//ZNF131//ZNF655//BRD3//TCF7L1//PCGF5//LCOR//WASL//ZBTB47//ST18//HIPK3//ELAVL2//NR5A2//NRBF2//MLLT6//RNF141//PAK6//SNAPC1//BTG1//SNIP1//RPS6KA4//RPS6KA5//CDKN1A//CRK//EMX2//ETS1//BOLA3//NR3C2//PLEK//ZNFX1//NKX2-4//RFX7//BDP1//CDK8//E2F3//TEAD1//KAT2B//CCNC//CPEB2//CPEB1//CPEB4//MTPN//DDX6//MKNK2//ACO1//TSC1//KBTBD8//FUT9//SERP1//ST8SIA5//MAN1A1//MAN2A1//B3GNT5//B3GALT2//CCDC126//VEGFB//NTNG1//RECK//PIGA//GLUD2//LGSN//SERINC3//PSAT1//ELOVL4//SCD5//LCLAT1//DDHD1//CDS1//ABHD3//SLC44A1//PIKFYVE//PIK3CB//PTEN//PLEKHA1//PLEKHA3//MTMR9//PIK3R3//SPHK2//ACBD3//LBR//MSMO1//CHCHD10//G3BP2//NF2//ACSL1//RIMKLB//DGKE//BCAT2//MTRR//EREG//SLC2A4//CNEP1R1//LDLR//FBXW7//ACOT7//PPCS//FAM129A//FMR1//PRR16//FXR1//ST3GAL3//MAP3K2//MAPRE3//BAMBI//RGMB//LRP6//MAP3K5//WNT7A//ACVR1//DKK3//NRG1//RASD1//TSG101//BTAF1//PPM1F//TOB1//NANOS1//CAPRIN2//OGN//CHST1//ZDHHC1//ST6GAL2//MCFD2//ZDHHC5//ELOVL7//PPM1L//SPTSSA//SGPP1//CHST7//DSEL//SOSTDC1//TMEM100//RGMA//BMP3//TGFB2//SULF1//CTDSPL2//ADRB1//PTHLH//CALM2//EGLN1//CMKLR1//DCP2//CCT6A//EPHA5//P2RY1//UXS1//GAB1//GRB2//NCK1//RNF128//ADIPOR2//ERRFI1//QKI//INTS8//NABP1//ITM2B//FZD6//NPLOC4//ITGAV//TMEFF2//MID1IP1//MAP2K4//S1PR1//DLL1//IL2//ITGA6//MET//PPP1R12A//OSM//PPP3R1//DCAF6//PAIP2//PPT2//INHBB//KDR//CCL2//YTHDF2//EGLN3//PFKP//ATG16L1//IDH1//ORMDL3//GSTA2//RAB3GAP1//RAP2C//TOB2//PPP1R15B//DUSP1//GJA1//NSMAF//NPNT//UNK// |
| GO:0060485 | mesenchyme development | Biological process | 35 | 258 | 934 | 17653 | 2.56401158640838 | 2.95749358155599e-07 | 1.53857916092794e-05 | 6.52907618921832 | 0.0374732334047109 | SEMA4B//ACVR1//FOXF2//PPP3R1//TGFB2//HMGA2//NOG//ERG//FOXF1//BMPR2//TMEM100//EFNA1//HAND2//TGFBR2//ZFPM2//FOXC1//BAMBI//TGFB1I1//PPP2CA//PTEN//LDLRAD4//LRP6//KBTBD8//RDH10//EDN1//NRG1//SOX11//FRZB//MAPK1//OSR1//PKD2//TCF21//ACTC1//BNC2//HAND1// |
| GO:0043170 | macromolecule metabolic process | Biological process | 583 | 9622 | 934 | 17653 | 1.14518288077332 | 3.04913053381064e-07 | 1.57414273283674e-05 | 6.51582398301403 | 0.624197002141328 | IGF1//NFIA//NFIB//RBBP7//RBBP8//RRM2//CDKN1A//GADD45A//PTEN//CCNE2//E2F1//NPAT//TSHZ1//HMG20A//KLF2//ZBTB18//CEBPB//ZNF268//TCERG1//WWP2//KLF12//OSR1//CRY2//GLIS3//ARX//DNMT3B//DR1//EDN1//EFNA1//CC2D1B//EN1//ESR1//FHL2//CPEB3//FOXF1//FOXC1//MYT1L//CIC//ZFPM2//DNAJB5//GATA6//CNOT7//HSBP1//AR//JARID2//JUN//ZNF568//MAF//MDM4//MYB//NR4A2//RLIM//KLF3//ZBTB7A//PIAS4//POU4F1//PPARG//CC2D1A//TSHZ3//ZBTB4//RARB//REST//RNF2//PRDM16//SOX17//SOX11//STAT3//ZEB1//TCF21//TFAP2A//TFAP2C//TGIF1//KLF10//TNF//UBE2I//VLDLR//WFS1//ZNF217//LRP8//BTG2//BHLHE41//TBL1XR1//NR4A3//HMGA2//ARID5B//BRMS1L//BHLHE40//RUNX3//URI1//PER2//SQSTM1//NOG//KLF4//HAND1//FNIP1//PHF14//USP3//RANBP9//PPM1L//SPRED1//DUSP5//EREG//RASGEF1A//GRB2//NRG1//IL2//JAK1//MAP3K5//MET//MINK1//TAOK3//MAPK1//CCL2//SOS1//LAMTOR3//DCP2//PPP2CA//RPL15//MAP3K2//CRK//CRKL//ADAM9//LPAR1//LPAR3//NTF3//PIK3CB//PRKAA1//MAP2K4//DUSP10//DUSP1//DUSP2//RGS3//TOPORS//HECTD2//RNF145//ZNRF2//FBXL5//RNF180//MYLIP//ANKIB1//UBE2W//FBXW7//HECW2//SKP1//THOP1//LONRF3//FBXO30//CBFB//UBE3B//LONRF1//ZFAND4//CNOT6L//PAN3//CNOT6//COPS8//RBFOX3//FMR1//MBNL1//RBM25//SRSF2//ELAVL2//NOVA1//PTGES3//NABP1//GTF2E1//SECISBP2L//CAMLG//EPS15//NSF//DRD1//FAM129A//LRP6//PPP1R15B//SLIT2//EFNA5//EPHA7//NPTN//KDR//P2RY1//AKTIP//CCND2//LYPLA1//F3//DUS2//IRF9//KLF6//CREBRF//EGR2//ELK3//ERG//FOXJ3//FOXF2//FLI1//GRHL1//HIVEP2//IRF1//LHX1//MEF2D//MEOX2//MYBL1//NEUROD1//NHLH2//OTX2//PAX9//PBX3//POU3F2//RAD21//ARID4A//SHOX//BCL11B//BMPR2//SOX4//BTF3//TEF//TGFB1I1//THRA//TMF1//DEK//FOSL1//KLF7//FUBP3//HAND2//CLOCK//POLR3G//PKD2//ERRFI1//TSG101//SOCS5//TRIM3//KLHL2//RNF38//MYCBP2//FBXO33//KLHL3//SPOPL//DCAF8//DCAF6//FEM1C//KLHL14//MARCH4//RNF4//MARCH7//UBE2Z//RNF128//KLHL15//KBTBD8//ARRDC4//CHST7//CHST1//UGP2//HS3ST5//XYLT2//HS2ST1//HS3ST3A1//HS3ST1//GNS//NME1//REV3L//GMNC//ESCO2//EPC2//USP28//USP45//ATXN3//KDM2A//MTRR//HMGB3//BAHD1//H2AFZ//KMT2B//DPY30//ARID4B//KHDRBS3//ZMYND11//TADA1//ZNF597//CTNND2//ZNF782//NKX2-3//ZNF800//E2F5//ZNF367//EZH1//ZBTB9//FLII//LHX6//HBP1//ATAD2//SERTAD3//MDFIC//MNX1//HOXA3//HOXD1//FOXN2//ZBTB41//ZBTB34//LHX8//NEUROG1//NFE2//NPAS2//IER5//BRWD1//BNC2//ZNF532//CDCA7L//TRERF1//LMO3//SLC2A4RG//PHTF2//ZNF630//ZNF287//ZNF512B//ZBTB26//MIER1//RBL2//ATXN1//DMRTA1//SMARCD2//STAT4//ZNF2//ZNF3//ZNF131//ZNF655//BRD3//TCF7L1//PCGF5//LCOR//WASL//ZBTB47//ST18//HIPK3//NUP35//NR5A2//NRBF2//MLLT6//RNF141//PAK6//SNAPC1//BTG1//SNIP1//RPS6KA4//RPS6KA5//EMX2//ETS1//BOLA3//NR3C2//PLEK//ZNFX1//NKX2-4//RFX7//BDP1//CDK8//E2F3//TEAD1//KAT2B//CCNC//CMTR2//ZFC3H1//RTCA//PPP4R2//ESRP1//CPEB1//QKI//AGFG1//NUP43//CPEB2//CPEB4//MTPN//DDX6//MKNK2//ACO1//TSC1//SERP1//UEVLD//RIMKLB//IMMP2L//ADAM10//CDK6//FASTK//PDIK1L//CDK19//MORC3//MMD//DSTYK//LATS2//CDK17//PRKACB//FAM20C//CCL8//TGFB2//TGFBR2//ACVR1//STK17B//PPM1E//FLRT1//GNAQ//NCK1//NF2//ASPN//YWHAG//CAMK2N2//PPM1F//PTPN21//PPP1R12A//CTDSPL2//PPP3R1//PPP6C//PTPN4//NAA30//PCMTD1//ASPH//FUT9//ST8SIA5//MAN1A1//MAN2A1//ST8SIA3//B3GNT5//B3GALT2//CCDC126//GFPT2//EDEM1//VEGFB//NTNG1//RECK//PTAR1//PIGA//MMP2//CTSA//NLN//NPEPL1//ADAM12//ADAM18//LMLN//ZFYVE9//USP24//USP27X//NPLOC4//USP46//BAG6//CDC23//DLC1//EGLN3//PMAIP1//DLL1//G3BP2//PTGER4//ST6GALNAC3//ST6GALNAC6//SLC2A4//TOB1//TOB2//PTHLH//NANOS1//RAB3GAP1//GJA1//ITGB8//LDLR//MSN//ACTC1//ZNF804A//NDFIP2//CALM2//GDF11//INHBB//BMP3//SERPINI1//SPOCK1//PCOLCE2//PRR16//FXR1//INTS8//GNPTG//ST3GAL3//RBM47//MAPRE3//BAMBI//RGMB//WNT7A//DKK3//RASD1//BTAF1//DMWD//OTUD3//WDR20//JOSD1//CAPRIN2//VKORC1L1//CEP41//STK33//MASTL//EPHA2//EPHA4//EPHA5//PKDCC//OGN//TMX3//ZDHHC1//ST6GAL2//ST3GAL5//MCFD2//ZDHHC5//CRTAP//EGLN1//SLK//SULF1//DSEL//SLC35D1//UBXN4//DNAJB9//DERL2//JKAMP//SOSTDC1//TMEM100//RGMA//BAG5//GRIA1//RAP2C//CMKLR1//CCT6A//PPP1R37//OSM//PFN2//IFNA1//ADCY3//PRKAR2B//CNEP1R1//NSMF//PTPDC1//PTPRG//PTP4A1//CDC14A//BNIP3//DNAJB12//DCTPP1//ITM2B//TDRD5//ARL6IP1//UBXN2B//CD2AP//PLAA//VPS37A//BCL2L11//ITGAV//FZD6//AHNAK//YTHDF2//FZD10//AIDA//TWF1//CKAP4//RAB38//MIA3//ANO8//RAB12//TTR//TMEFF2//IPO5//CCNY//S1PR1//ITGA6//PAIP2//ULK2//ITGA5//SH3D19//ARRDC3//CHRM5//MTMR9//PMEPA1//LDLRAD4//ATG16L1//NPNT//DNAJC27//UBA5//ACSL1//GLRX//REXO1//ZC3H12C//PPT2//SNAP25//ORMDL3//FRY//NSMAF//UNK// |
| GO:0010562 | positive regulation of phosphorus metabolic process | Biological process | 99 | 1126 | 934 | 17653 | 1.66176056686229 | 3.42417756957688e-07 | 1.74118142128184e-05 | 6.46544372194822 | 0.105995717344754 | GADD45A//MINK1//TNF//MAP3K2//CRK//CRKL//MAP3K5//TAOK3//LAMTOR3//ADAM9//DUSP5//LPAR1//LPAR3//NRG1//IGF1//NTF3//PIK3CB//PRKAA1//MAPK1//MAP2K4//FAM129A//EFNA1//EFNA5//EPHA7//FMR1//NPTN//KDR//P2RY1//AKTIP//SQSTM1//CCND2//FNIP1//EREG//FBXW7//COPS8//MDFIC//NPNT//CALM2//GDF11//INHBB//BMP3//BMPR2//TGFB2//ACVR1//MTMR9//PLEK//ADRB1//PTHLH//PKD2//RAP2C//PAK6//TGFBR2//SLK//PPP1R15B//RPS6KA4//RPS6KA5//OSM//PFN2//CAPRIN2//IFNA1//EPHA2//EPHA4//EPHA5//DSTYK//MET//ADCY3//PRKACB//PRKAR2B//DLC1//CNEP1R1//NSMF//PPP1R12A//PTGES3//KCTD20//ITGA6//AR//BTBD10//IL2//STAT3//EDN1//ITGAV//FZD10//CCNY//MAPRE3//CCNC//CDKN1A//MMD//VLDLR//WNT7A//ITGA5//LRP8//JUN//DNAJC27//PTEN//CCL2//CCL8//HAND2//ACSL1//PPP2CA// |
| GO:0045937 | positive regulation of phosphate metabolic process | Biological process | 99 | 1126 | 934 | 17653 | 1.66176056686229 | 3.42417756957688e-07 | 1.74118142128184e-05 | 6.46544372194822 | 0.105995717344754 | GADD45A//MINK1//TNF//MAP3K2//CRK//CRKL//MAP3K5//TAOK3//LAMTOR3//ADAM9//DUSP5//LPAR1//LPAR3//NRG1//IGF1//NTF3//PIK3CB//PRKAA1//MAPK1//MAP2K4//FAM129A//EFNA1//EFNA5//EPHA7//FMR1//NPTN//KDR//P2RY1//AKTIP//SQSTM1//CCND2//FNIP1//EREG//FBXW7//COPS8//MDFIC//NPNT//CALM2//GDF11//INHBB//BMP3//BMPR2//TGFB2//ACVR1//MTMR9//PLEK//ADRB1//PTHLH//PKD2//RAP2C//PAK6//TGFBR2//SLK//PPP1R15B//RPS6KA4//RPS6KA5//OSM//PFN2//CAPRIN2//IFNA1//EPHA2//EPHA4//EPHA5//DSTYK//MET//ADCY3//PRKACB//PRKAR2B//DLC1//CNEP1R1//NSMF//PPP1R12A//PTGES3//KCTD20//ITGA6//AR//BTBD10//IL2//STAT3//EDN1//ITGAV//FZD10//CCNY//MAPRE3//CCNC//CDKN1A//MMD//VLDLR//WNT7A//ITGA5//LRP8//JUN//DNAJC27//PTEN//CCL2//CCL8//HAND2//ACSL1//PPP2CA// |
| GO:0006796 | phosphate-containing compound metabolic process | Biological process | 232 | 3255 | 934 | 17653 | 1.34712729880237 | 3.49819669214862e-07 | 1.7655450917165e-05 | 6.45615577521377 | 0.248394004282655 | CDKN1A//GADD45A//PTEN//CCNE2//RANBP9//PPM1L//SPRED1//DUSP5//EREG//RASGEF1A//GRB2//NRG1//IL2//JAK1//MAP3K5//MET//MINK1//TAOK3//MAPK1//CCL2//SOS1//TNF//LAMTOR3//MAP3K2//CRK//CRKL//ADAM9//LPAR1//LPAR3//IGF1//NTF3//PIK3CB//PRKAA1//MAP2K4//DUSP10//DUSP1//DUSP2//PPP2CA//RGS3//DRD1//FNIP1//FAM129A//LRP6//PPP1R15B//SLIT2//EFNA1//EFNA5//EPHA7//FMR1//NPTN//KDR//P2RY1//AKTIP//SQSTM1//CCND2//FBXW7//ERRFI1//TSG101//SOCS5//PFKP//GFPT2//NUP35//NUP43//CHCHD10//COX8C//COX7A2L//HPRT1//NME1//CMPK1//ADCY3//IMPDH1//PDE10A//DCTPP1//HIPK3//ADAM10//CDK6//CDK8//FASTK//PDIK1L//ERG//CDK19//MORC3//MMD//PAN3//DSTYK//LATS2//MKNK2//CDK17//PRKACB//FAM20C//CCL8//TGFB2//TGFBR2//RUNX3//CCNC//RPS6KA4//ACVR1//RPS6KA5//STK17B//PPM1E//FLRT1//GNAQ//NCK1//NF2//ASPN//DUS2//YWHAG//CAMK2N2//PPM1F//PTPN21//PPP4R2//PPP1R12A//CTDSPL2//PPP3R1//PPP6C//PTPN4//PIGA//GATA6//PLAA//LCLAT1//DDHD1//CDS1//ABHD3//SLC44A1//SERINC3//PIKFYVE//PLEKHA1//PLEKHA3//MTMR9//PIK3R3//SGPP1//SPHK2//IDH1//COPS8//PTGER4//MDFIC//EPHA2//PTHLH//DGKE//RRM2//NAPEPLD//NPNT//CALM2//GDF11//INHBB//BMP3//BMPR2//LDLR//PLEK//PPP1R37//PPP1R12C//PLEKHF2//URI1//ACOT7//PPCS//DGKH//STAT3//HDHD2//STK33//MASTL//OSR1//EPHA4//EPHA5//PKDCC//UGP2//PAK6//SLK//PKD2//TBL1XR1//ADRB1//EGLN1//JUN//RAP2C//CRY2//OSM//PFN2//CAPRIN2//IFNA1//PRKAR2B//DLC1//CNEP1R1//NSMF//PTPDC1//PTPRG//PTP4A1//CDC14A//HMGA2//GAB1//OSBPL5//PTGES3//KCTD20//ITGA6//AR//BTBD10//CNOT7//EDN1//ITGAV//FZD10//AIDA//TWF1//NRBF2//RBL2//KLF4//UBXN2B//TSC1//IPO5//KAT2B//CCNY//MAPRE3//VLDLR//MPP1//RAB23//ZMYND11//WNT7A//ULK2//NAGK//GNPTG//ESR1//EFR3A//ZFYVE21//ITGA5//LRP8//SKP1//CHRM5//PMEPA1//LDLRAD4//NOG//LMO3//DNAJC27//HAND2//ACSL1//SLC20A1// |
| GO:0031401 | positive regulation of protein modification process | Biological process | 103 | 1188 | 934 | 17653 | 1.63867349440155 | 3.79370948338641e-07 | 1.90050794341795e-05 | 6.42093592979765 | 0.110278372591006 | GADD45A//MINK1//TNF//MAP3K2//CRK//CRKL//MAP3K5//TAOK3//LAMTOR3//ADAM9//DUSP5//LPAR1//LPAR3//NRG1//IGF1//NTF3//PIK3CB//PRKAA1//MAPK1//MAP2K4//FAM129A//EFNA1//EFNA5//EPHA7//FMR1//NPTN//KDR//P2RY1//AKTIP//SQSTM1//CCND2//FNIP1//EREG//FBXW7//COPS8//MDFIC//CALM2//GDF11//INHBB//BMP3//BMPR2//TGFB2//ACVR1//NDFIP2//WFS1//PKD2//RAP2C//PAK6//TGFBR2//SLK//PPP1R15B//RPS6KA4//RPS6KA5//OSM//PFN2//CAPRIN2//IFNA1//PIAS4//ADCY3//PRKACB//PRKAR2B//DLC1//CNEP1R1//NSMF//PPP1R12A//IL2//STAT3//EDN1//EPHA2//EPHA4//EPHA5//AR//ITGAV//MET//FZD10//CCNY//MAPRE3//CCNC//CDKN1A//MMD//VLDLR//WNT7A//ITGA5//LRP8//TOPORS//ARRDC3//ARRDC4//DNMT3B//MYB//JARID2//NPNT//DSTYK//JUN//DNAJC27//PTEN//CCL2//CCL8//HAND2//ACSL1//PPP2CA//RAB3GAP1//UBE2I//SOX4// |
| GO:0006793 | phosphorus metabolic process | Biological process | 237 | 3352 | 934 | 17653 | 1.33633696268775 | 4.74967476049988e-07 | 2.36191547097505e-05 | 6.32333612817644 | 0.253747323340471 | CDKN1A//GADD45A//PTEN//CCNE2//RANBP9//PPM1L//SPRED1//DUSP5//EREG//RASGEF1A//GRB2//NRG1//IL2//JAK1//MAP3K5//MET//MINK1//TAOK3//MAPK1//CCL2//SOS1//TNF//LAMTOR3//MAP3K2//CRK//CRKL//ADAM9//LPAR1//LPAR3//IGF1//NTF3//PIK3CB//PRKAA1//MAP2K4//DUSP10//DUSP1//DUSP2//PPP2CA//RGS3//DRD1//FNIP1//FAM129A//LRP6//PPP1R15B//SLIT2//EFNA1//EFNA5//EPHA7//FMR1//NPTN//KDR//P2RY1//AKTIP//SQSTM1//CCND2//FBXW7//ERRFI1//TSG101//SOCS5//PFKP//GFPT2//UGP2//NAGK//SLC35D1//NUP35//NUP43//CHCHD10//COX8C//COX7A2L//HPRT1//NME1//CMPK1//ADCY3//IMPDH1//PDE10A//DCTPP1//HIPK3//ADAM10//CDK6//CDK8//FASTK//PDIK1L//ERG//CDK19//MORC3//MMD//PAN3//DSTYK//LATS2//MKNK2//CDK17//PRKACB//FAM20C//CCL8//TGFB2//TGFBR2//RUNX3//CCNC//RPS6KA4//ACVR1//RPS6KA5//STK17B//PPM1E//FLRT1//GNAQ//NCK1//NF2//ASPN//DUS2//YWHAG//CAMK2N2//PPM1F//PTPN21//PPP4R2//PPP1R12A//CTDSPL2//PPP3R1//PPP6C//PTPN4//PIGA//ACOT7//GATA6//PLAA//LCLAT1//DDHD1//CDS1//ABHD3//SLC44A1//SERINC3//PIKFYVE//PLEKHA1//PLEKHA3//MTMR9//PIK3R3//SGPP1//SPHK2//IDH1//SLC20A1//COPS8//PTGER4//MDFIC//EPHA2//PTHLH//DGKE//RRM2//NAPEPLD//NPNT//CALM2//GDF11//INHBB//BMP3//BMPR2//LDLR//PLEK//PPP1R37//PPP1R12C//PLEKHF2//URI1//PPCS//DGKH//STAT3//HDHD2//STK33//MASTL//OSR1//EPHA4//EPHA5//PKDCC//PAK6//SLK//PKD2//TBL1XR1//ADRB1//EGLN1//JUN//RAP2C//CRY2//OSM//PFN2//CAPRIN2//IFNA1//UXS1//PRKAR2B//DLC1//CNEP1R1//NSMF//PTPDC1//PTPRG//PTP4A1//CDC14A//ACSL1//ELOVL7//HMGA2//GAB1//OSBPL5//PTGES3//KCTD20//ITGA6//AR//BTBD10//CNOT7//EDN1//ITGAV//FZD10//AIDA//TWF1//NRBF2//RBL2//KLF4//UBXN2B//TSC1//IPO5//KAT2B//CCNY//MAPRE3//VLDLR//MPP1//RAB23//ZMYND11//WNT7A//ULK2//GNPTG//ESR1//EFR3A//ZFYVE21//SCD5//PPT2//ITGA5//LRP8//SKP1//CHRM5//PMEPA1//LDLRAD4//NOG//LMO3//DNAJC27//HAND2// |
| GO:0006468 | protein phosphorylation | Biological process | 151 | 1946 | 934 | 17653 | 1.46657999388192 | 6.38684163475194e-07 | 3.15286204203119e-05 | 6.19471385227884 | 0.161670235546039 | CDKN1A//GADD45A//PTEN//CCNE2//RANBP9//PPM1L//SPRED1//DUSP5//EREG//RASGEF1A//GRB2//NRG1//IL2//JAK1//MAP3K5//MET//MINK1//TAOK3//MAPK1//CCL2//SOS1//TNF//LAMTOR3//MAP3K2//CRK//CRKL//ADAM9//LPAR1//LPAR3//IGF1//NTF3//PIK3CB//PRKAA1//MAP2K4//DUSP10//DUSP1//DUSP2//PPP2CA//RGS3//DRD1//FNIP1//FAM129A//LRP6//PPP1R15B//SLIT2//EFNA1//EFNA5//EPHA7//FMR1//NPTN//KDR//P2RY1//AKTIP//SQSTM1//CCND2//FBXW7//ERRFI1//TSG101//SOCS5//PPM1E//FLRT1//GNAQ//NCK1//NF2//ASPN//DUS2//YWHAG//CAMK2N2//PPM1F//TGFBR2//COPS8//PTGER4//MDFIC//CALM2//GDF11//INHBB//BMP3//BMPR2//TGFB2//ACVR1//RPS6KA4//RPS6KA5//HIPK3//MORC3//LATS2//MKNK2//STK33//MASTL//OSR1//EPHA2//EPHA4//EPHA5//DSTYK//PKDCC//PAK6//SLK//CDK6//PKD2//JUN//RAP2C//OSM//PFN2//CAPRIN2//IFNA1//ADCY3//PRKACB//PRKAR2B//HMGA2//CNOT7//STAT3//EDN1//AR//ITGAV//FZD10//AIDA//TWF1//IPO5//KAT2B//CCNY//MAPRE3//CCNC//TSC1//MMD//VLDLR//ZMYND11//WNT7A//STK17B//ULK2//ITGA5//LRP8//SKP1//PMEPA1//LDLRAD4//NOG//LMO3//KLF4//NPNT//DNAJC27//CCL8//HAND2//ACSL1//ADAM10//CDK8//FASTK//PDIK1L//ERG//CDK19//PAN3//CDK17//FAM20C//RUNX3// |
| GO:0060429 | epithelium development | Biological process | 107 | 1264 | 934 | 17653 | 1.59995713956577 | 7.03503451355629e-07 | 3.44767669675226e-05 | 6.15273376759522 | 0.114561027837259 | EDN1//PPP3R1//TGFBR2//ACVR1//GDF11//OSR1//FOXC1//NPNT//LHX1//RARB//TCF21//SLIT2//PKD2//FOXF1//DLL1//MEOX2//NOG//SOX4//SOX11//DLC1//LRP6//PRKACB//TGFB2//TSC1//FZD6//CLIC4//MET//APCDD1//SOS1//GJA1//SOX17//HAND1//HAND2//ARHGAP12//ESR1//KDR//GATA6//CDK6//NEUROD1//PALLD//BCL11B//AR//FRMD6//TFAP2A//KLF4//TGFB1I1//WNT7A//EPHA2//EREG//LATS2//TSG101//ADAM9//CNN3//NR5A2//GSTA2//PPARG//KIAA1109//EN1//SOSTDC1//KRT27//DSC2//CEBPB//MAPK1//JUN//ITGA5//FOXF2//TMEFF2//RHOC//MSN//S1PR1//ZEB1//TMEM100//BTG1//GRHL1//ERRFI1//CBFB//TNF//FRZB//VANGL1//RDH10//NFIB//KLF2//THRA//CDKN1A//NTN4//GRB2//SULF1//BTBD7//BMPR2//NRG1//RAP1B//MAF//NF2//TMOD1//TBC1D20//FAM20C//ARX//KLHL3//EPHA4//EPHA7//RAP2C//ARID4B//ARID4A//PTEN//DUSP10//RHEB//CLOCK// |
| GO:0071230 | cellular response to amino acid stimulus | Biological process | 15 | 63 | 934 | 17653 | 4.50010196798205 | 7.13639728863901e-07 | 3.47219099734285e-05 | 6.14652098045637 | 0.0160599571734475 | SESN3//SESN1//RRAGD//CEBPB//COL1A2//CPEB3//NSMF//GLRA1//IPO5//MMP2//CPEB1//ZEB1//TNF//CPEB4//LAMTOR3// |
| GO:0001501 | skeletal system development | Biological process | 53 | 493 | 934 | 17653 | 2.03189188250062 | 7.32356102189654e-07 | 3.53780308507759e-05 | 6.13527769543009 | 0.056745182012848 | THRA//WNT7A//FOXC1//MEF2D//OSR1//NFIB//HMGA2//RUNX3//SULF1//BMPR2//RARB//TGFBR2//MAF//PTHLH//PKDCC//IGF1//FLI1//FAM20C//TFAP2A//RDH10//LHX1//HOXA3//DSCAML1//SOX11//ZEB1//PLEKHA1//ARID5B//SLC35D1//HOXD1//NOG//EDN1//ITGB8//BMP3//MMP2//PTGER4//LRP6//HAND1//HAND2//FRZB//NEUROG1//RAB23//FBXW7//GDF11//CMKLR1//COL1A2//HAPLN1//EN1//SERP1//GNAQ//SHOX//SOX4//TGFB2//KLF10// |
| GO:0007167 | enzyme linked receptor protein signaling pathway | Biological process | 91 | 1028 | 934 | 17653 | 1.67309238537232 | 7.92207639149616e-07 | 3.79978742097082e-05 | 6.10116097406941 | 0.0974304068522484 | GRB2//JAK1//KDR//NTF3//PIK3CB//PTPRG//PALM2//PPM1L//BMPR2//EREG//FBXW7//ERRFI1//TSG101//SOCS5//EFNA5//NRG1//EPS8//EPS15//GAB1//SOS1//CAMLG//RPS6KA5//COL1A2//JUN//TGFB2//TGFBR2//KLF10//ADAM9//ACVR1//ZFYVE9//APPL1//PIK3R3//SPRED1//FLRT1//ESRP1//MAPK1//F3//GDF11//INHBB//BMP3//PMEPA1//LDLRAD4//DKK3//SOX11//ZEB1//TOB1//SOSTDC1//RGMB//TMEM100//RGMA//NOG//NPNT//TGFB1I1//BAMBI//ASPN//PRDM16//RASL11B//SULF1//GATA6//CTDSPL2//ITGA5//FOXC1//OTX2//FAM20C//SHISA2//EPN1//IGF1//AR//DSTYK//NPTN//SESN3//NCK1//TSC1//PTEN//PLEKHA1//ARID5B//CRK//ITGAV//VEGFB//MET//EFNA1//EPHA2//EPHA4//EPHA5//EPHA7//MMP2//ARHGEF28//WASL//STAT3//DLL1//PHF14// |
| GO:0048646 | anatomical structure formation involved in morphogenesis | Biological process | 98 | 1133 | 934 | 17653 | 1.63481197707097 | 8.30646014101364e-07 | 3.92886314452881e-05 | 6.0805840145736 | 0.104925053533191 | VASH2//S1PR1//EFNA1//EPHA2//ELK3//EREG//FOXC1//GAB1//CLIC4//HOXA3//ITGA5//ITGAV//JUN//KDR//MEOX2//MMP2//TMEM100//PTEN//CCL2//SOX17//TGFB2//VEGFB//WNT7A//HAND1//HAND2//EDN1//PPP3R1//TGFBR2//ACVR1//TBC1D20//AGFG1//TMF1//LHX1//DUSP1//DUSP2//DUSP5//NOG//NF2//BMPR2//NR4A3//FOXF1//DLL1//LATS2//SOX4//SOX11//DLC1//LRP6//PRKACB//TSC1//FZD6//SLIT2//ERG//TFAP2A//GATA6//KLF4//ADAM12//TOPORS//FJX1//KBTBD8//SPRED1//SULF1//PPARG//NEUROG1//EGR2//MTPN//TMOD3//TMOD1//ACTC1//PIKFYVE//ADAM9//BCL2L11//HSBP1//LAMA3//HMGA2//CFL2//ETS1//EGLN1//F3//ITGB8//STAT3//BTG1//AR//PIK3CB//FHL2//RDH10//MAPK1//NFIB//ADIPOR2//FAM20C//OSR1//TCF21//OTX2//ITGA6//KLF2//EPN1//JAK1//TNF//GRB2// |
| GO:0048608 | reproductive structure development | Biological process | 48 | 430 | 934 | 17653 | 2.10981524824461 | 8.37076640828109e-07 | 3.92886314452881e-05 | 6.07723477717406 | 0.0513918629550321 | CEBPB//FOXC1//INHBB//DMRTA1//IMMP2L//KMT2B//ESR1//EREG//PLEKHA1//PPARG//PKD2//BMPR2//ZFPM2//OSR1//RDH10//BCL2L11//RNF38//GATA6//TFAP2C//TGFB2//ARID5B//IDH1//LHX8//AR//TMF1//NEUROG1//LRP6//LHX1//GJA1//GJB2//FOXF2//TCF21//WNT7A//NOG//ZNF568//GRB2//ITGB8//FOSL1//SULF1//HAND1//EGLN1//MAPK1//VASH2//PTEN//SLIT2//TBC1D20//ARID4B//ARID4A// |
| GO:0060322 | head development | Biological process | 71 | 741 | 934 | 17653 | 1.81097220897739 | 8.41491694048509e-07 | 3.92886314452881e-05 | 6.07495016637467 | 0.076017130620985 | BCL2L11//DPYSL2//S1PR1//EGR2//EPHA7//RAB3GAP1//CIC//SPHK2//SLC17A7//DSCAML1//PTPRG//RARB//ATXN1//BMPR2//TGFBR2//THRA//BAG6//IMMP2L//B3GNT5//NOG//POU4F1//LHX1//LRP8//CDK6//DRD1//EMX2//NEUROD1//PTEN//BTG2//LPAR1//EN1//FOXC1//DLC1//LRP6//DLL1//MTPN//WNT7A//NFIB//HPRT1//ARX//CALM2//SLC32A1//EPHA5//EZH1//NF2//NME1//TSC1//NR4A3//BCL11B//LHX6//POU3F2//SLIT2//NRG1//ETS1//GNAQ//LHX8//C21ORF91//NR4A2//TACC2//BNIP3//E2F1//OTX2//FZD6//MAPK1//MMP2//ASPH//PAX9//PLEKHA1//ARID5B//NANOS1//SOS1// |
| GO:0035556 | intracellular signal transduction | Biological process | 207 | 2871 | 934 | 17653 | 1.36272680284347 | 8.43415796711678e-07 | 3.92886314452881e-05 | 6.07395826914939 | 0.221627408993576 | RANBP9//PPM1L//SPRED1//DUSP5//EREG//RASGEF1A//GRB2//NRG1//IL2//JAK1//MAP3K5//MET//MINK1//TAOK3//MAPK1//CCL2//SOS1//TNF//LAMTOR3//GADD45A//MAP3K2//CRK//CRKL//ADAM9//LPAR1//LPAR3//IGF1//NTF3//PIK3CB//PRKAA1//MAP2K4//DUSP10//DUSP1//DUSP2//PPP2CA//RGS3//CDKN1A//E2F1//CNOT6L//CNOT7//MDM4//CNOT6//RBL2//SOX4//BTG2//ADCY3//DRD1//GNAQ//PTGER4//PTHLH//EDN1//NKIRAS1//SNIP1//COPS8//MDFIC//PKD2//STAT3//STAT4//SOCS5//NEUROD1//RASD1//RAPGEF4//RGL1//DOCK9//RAB38//RHOC//RND3//ARHGAP1//RALGPS2//SOS2//DOCK5//JUN//USP28//G3BP2//CDC42EP3//COL1A2//EPS8//ARHGAP6//PAK6//BCL2L11//TOPORS//EPHA2//MOAP1//PDE10A//AKAP11//CALM2//PLEKHA1//PTEN//KLF4//KDR//TGFB2//EPHA5//SLK//FNIP1//TSC1//RRAGD//RHEB//ARAP2//APPL1//AGFG1//AGFG2//PSD//GIT2//RAB12//RAB21//RAB30//RAB9B//DNAJC27//RAB23//RAB22A//RAB28//RAP2C//RAP1B//IFNA1//PPP3R1//FGD4//FGD5//ARHGEF4//ARHGEF28//DLC1//LATS2//TEAD1//NF2//SOX11//SKP1//SESN3//NPLOC4//OSM//BAG6//ZFAND6//SQSTM1//GJA1//NDFIP2//CC2D1A//MIER1//SLC20A1//UBE2I//ZMYND11//ESR1//TSG101//GDF11//INHBB//BMP3//EFNA1//EPHA4//EPHA7//AR//ITGAV//TMEM100//FZD10//HIPK3//AIDA//PMAIP1//PEX5L//ADRB1//WNT7A//FLRT1//ASPN//CMKLR1//PLEK//FAM13A//TAGAP//TRIP10//ARHGAP12//CD2AP//SLIT2//F3//GAB1//OTUD3//CEBPB//ERRFI1//LMO3//NPNT//DSTYK//NPTN//P2RY1//FBXW7//CCL8//HAND2//FIBIN//POU4F1//ZMAT3//SGPP1//PPM1F//NR5A2//RBBP7//RBBP8//BAG5//PIAS4//WFS1//SERINC3//NCK1//SESN1//BNIP3//URI1//DGKH//DCX//PIKFYVE//MKNK2//TREM1//PRKAR2B//STK33//MASTL//DGKE//RPS6KA4//RPS6KA5//STK17B// |
| GO:0034645 | cellular macromolecule biosynthetic process | Biological process | 353 | 5401 | 934 | 17653 | 1.23529923675804 | 8.48165043769342e-07 | 3.92886314452881e-05 | 6.071519630498 | 0.377944325481799 | IGF1//NFIA//NFIB//RBBP7//RBBP8//RRM2//E2F1//NPAT//TSHZ1//HMG20A//KLF2//ZBTB18//CEBPB//ZNF268//TCERG1//WWP2//KLF12//OSR1//CRY2//GLIS3//ARX//DNMT3B//DR1//EDN1//EFNA1//CC2D1B//EN1//ESR1//FHL2//CPEB3//FOXF1//FOXC1//MYT1L//CIC//ZFPM2//DNAJB5//GATA6//CNOT7//HSBP1//AR//JARID2//JUN//ZNF568//MAF//MDM4//MYB//NR4A2//RLIM//KLF3//ZBTB7A//PIAS4//POU4F1//PPARG//CC2D1A//TSHZ3//ZBTB4//RARB//REST//RNF2//PRDM16//SOX17//SOX11//STAT3//ZEB1//TCF21//TFAP2A//TFAP2C//TGIF1//KLF10//TNF//UBE2I//VLDLR//WFS1//ZNF217//LRP8//BTG2//BHLHE41//TBL1XR1//NR4A3//HMGA2//ARID5B//BRMS1L//BHLHE40//RUNX3//URI1//PER2//SQSTM1//NOG//KLF4//HAND1//FNIP1//PHF14//USP3//GTF2E1//SECISBP2L//RPL15//IRF9//KLF6//CREBRF//EGR2//ELK3//ERG//FOXJ3//FOXF2//FLI1//GRHL1//HIVEP2//IRF1//LHX1//MEF2D//MEOX2//MYBL1//NEUROD1//NHLH2//OTX2//PAX9//PBX3//POU3F2//RAD21//ARID4A//SHOX//BCL11B//BMPR2//SOX4//BTF3//TEF//TGFB1I1//THRA//TMF1//DEK//FOSL1//KLF7//CBFB//FUBP3//HAND2//CLOCK//POLR3G//SRSF2//PKD2//UGP2//REV3L//GMNC//CCNE2//ESCO2//PPP2CA//BAHD1//H2AFZ//KMT2B//DPY30//TOPORS//KHDRBS3//ZMYND11//TADA1//ZNF597//CTNND2//ZNF782//NKX2-3//ZNF800//E2F5//ZNF367//EZH1//ZBTB9//KDM2A//MYCBP2//FLII//CNOT6L//EPC2//LHX6//HBP1//ATAD2//SERTAD3//MDFIC//MNX1//HMGB3//HOXA3//HOXD1//FOXN2//ZBTB41//ZBTB34//ATXN3//LHX8//NEUROG1//NFE2//NPAS2//IER5//ARID4B//BRWD1//BNC2//ZNF532//CDCA7L//PRKAA1//TRERF1//LMO3//MAPK1//SLC2A4RG//PHTF2//ZNF630//ZNF287//CNOT6//ZNF512B//ZBTB26//MIER1//RBL2//RNF4//ATXN1//DMRTA1//SMARCD2//STAT4//ZNF2//ZNF3//ZNF131//ZNF655//BRD3//TCF7L1//PCGF5//LCOR//WASL//ZBTB47//ST18//HIPK3//NUP35//ELAVL2//NR5A2//NRBF2//MLLT6//RNF141//PAK6//SNAPC1//BTG1//SNIP1//RPS6KA4//RPS6KA5//CDKN1A//CRK//EMX2//ETS1//BOLA3//NR3C2//PLEK//ZNFX1//NKX2-4//RFX7//BDP1//CDK8//E2F3//TEAD1//KAT2B//CCNC//CPEB2//CPEB1//CPEB4//MTPN//DDX6//MKNK2//ACO1//TSC1//KBTBD8//FUT9//SERP1//ST8SIA5//MAN1A1//MAN2A1//ST8SIA3//B3GNT5//B3GALT2//CCDC126//GFPT2//VEGFB//NTNG1//RECK//PIGA//PTGES3//G3BP2//NF2//EREG//XYLT2//HS2ST1//HS3ST5//FAM129A//FMR1//PRR16//FXR1//ST3GAL3//MAP3K2//MAPRE3//BAMBI//RGMB//LRP6//MAP3K5//WNT7A//ACVR1//DKK3//NRG1//RASD1//TSG101//BTAF1//PPM1F//TOB1//NANOS1//CAPRIN2//ZDHHC1//ST6GALNAC3//ST6GALNAC6//ST6GAL2//ST3GAL5//MCFD2//ZDHHC5//SLC35D1//CHST7//DSEL//SOSTDC1//TMEM100//RGMA//BMP3//TGFB2//SULF1//CTDSPL2//CMKLR1//DCP2//CCT6A//EPHA5//FBXW7//NCK1//INTS8//NABP1//ITM2B//EGLN1//FZD6//NPLOC4//MAP2K4//S1PR1//DLL1//IL2//ITGA6//MET//PPP1R12A//OSM//P2RY1//PPP3R1//DCAF6//PAIP2//PTEN//NUP43//YTHDF2//QKI//EGLN3//ATG16L1//RAB3GAP1//PPP1R15B//DUSP1//GJA1//NPNT//UNK// |
| GO:0060284 | regulation of cell development | Biological process | 79 | 858 | 934 | 17653 | 1.74024922258327 | 9.77326834479467e-07 | 4.49636828679227e-05 | 6.00996017663504 | 0.0845824411134904 | FRZB//PTEN//SNAP25//ZNF804A//STMN2//NPTN//NCK1//NME1//SERPINI1//TWF1//LPAR1//EPHA4//MYLIP//PTPRG//SPOCK1//TSC1//BAG5//NF2//DPYSL2//RAB21//WNT7A//DLL1//NTF3//BCL11B//YWHAG//HMG20A//DDX6//REST//DNMT3B//MMD//NEUROD1//NEUROG1//RARB//SOX11//ZEB1//GOLGA4//NRG1//EPHA7//ULK2//EFNA5//LPAR3//KLF4//RGMA//NOG//TNFRSF21//PPARG//RHEB//DUSP10//FMR1//NSMF//HECW2//FXR1//BMPR2//SEMA4B//POU4F1//PER2//MAN2A1//EFNA1//POU3F2//SLIT2//CAPRIN2//MYB//IGF1//EDN1//CPEB3//IL2//C21ORF91//LRP8//LDLR//VLDLR//CRK//CRKL//DOCK5//TNF//YTHDF2//PLAA//CLOCK//FBXW7//STAT3// |
| GO:1901701 | cellular response to oxygen-containing compound | Biological process | 92 | 1048 | 934 | 17653 | 1.65919790117201 | 9.84255760306495e-07 | 4.49764980199515e-05 | 6.00689203484405 | 0.0985010706638116 | CHRM2//CHRM5//GNAQ//GAB1//APPL1//GRB2//SOS1//PIK3R3//CEBPB//COL1A2//SESN3//CPEB3//NSMF//SESN1//GLRA1//IPO5//MMP2//RRAGD//CPEB1//ZEB1//TNF//CPEB4//LAMTOR3//MAPK1//CCL2//CPEB2//INHBB//ERRFI1//PPARG//PTEN//SLC2A4//YWHAG//KAT2B//JUN//PKD2//SYBU//NCK1//TSC1//IGF1//STAT3//EFNA5//EPHA5//KLF2//ETS1//MAP3K5//MYB//PRKAA1//PLEKHA1//BNIP3//KLF4//CDK19//CD274//PLSCR4//PDCD1LG2//ADAM9//PLAA//FZD10//OSR1//RARB//RAP1B//HCN2//NEUROD1//NME1//SOX4//UGT1A1//EDN1//NR4A2//NR4A3//ADCY3//GJB2//PRKACB//PRKAR2B//PTGER4//ESR1//H2AFZ//POU4F1//AR//MSN//LRP6//LRP8//E2F1//LDLR//SLIT2//DNMT3B//CRK//DRD1//MAP2K4//MET//KLF3//KLF10//LPAR1//EPHA4// |
| GO:0042981 | regulation of apoptotic process | Biological process | 126 | 1566 | 934 | 17653 | 1.52072411331807 | 1.0060309609218e-06 | 4.56630026088197e-05 | 5.99738865352404 | 0.134903640256959 | PTEN//IL2//FNIP1//DLC1//EGLN3//F3//PMAIP1//PPARG//TNF//HIPK3//TOPORS//CDKN1A//ZNF268//OSR1//DUSP1//EPHA2//EPHA4//EPHA5//EPHA7//FHL2//ARL6IP1//DSTYK//GATA6//IGF1//KDR//MDM4//MET//ZFAND6//PRKAA1//SPHK2//RARB//BCL11B//BNIP3//STAT3//ACTC1//TFAP2A//TMF1//WNT7A//BAG6//HMGA2//SQSTM1//CCND2//MAP3K5//HAND2//PIK3CB//THRA//DLL1//CCL2//EDN1//LRP6//MAP2K4//BCL2L11//FGD4//GADD45A//E2F1//LPAR1//CDK19//FRZB//LATS2//ITGA6//JUN//USP27X//NEUROD1//ARHGEF4//POU4F1//REST//MOAP1//ZMAT3//UBE2Z//SOS1//SOS2//SOX4//FOSL1//NSMAF//SLIT2//KLF4//PPM1F//NSMF//CEBPB//EN1//NTF3//NR4A2//WFS1//BTG2//NR4A3//CPEB4//MYB//TGFB2//EFNA1//DYNLL2//PPP3R1//YWHAG//PIAS4//BAG5//SERINC3//NCK1//FOXC1//FBXW7//CD274//E2F3//IER3IP1//BTG1//STK17B//ITGA5//NOG//INHBB//ZMYND11//AR//ITGAV//ACVR1//URI1//ST18//GDF11//MAP3K2//ESR1//TNFRSF21//RASSF3//NF2//NME1//MINK1//TAOK3//PAK6//RBM25//BMP3//ANP32E//SLK// |
| GO:0061458 | reproductive system development | Biological process | 48 | 433 | 934 | 17653 | 2.09519759063552 | 1.02292233460433e-06 | 4.61201583261939e-05 | 5.99015733885207 | 0.0513918629550321 | CEBPB//FOXC1//INHBB//DMRTA1//IMMP2L//KMT2B//ESR1//EREG//PLEKHA1//PPARG//PKD2//BMPR2//ZFPM2//OSR1//RDH10//BCL2L11//RNF38//GATA6//TFAP2C//TGFB2//ARID5B//IDH1//LHX8//AR//TMF1//NEUROG1//LRP6//LHX1//GJA1//GJB2//TCF21//FOXF2//WNT7A//NOG//ZNF568//GRB2//ITGB8//FOSL1//SULF1//HAND1//EGLN1//MAPK1//VASH2//PTEN//SLIT2//TBC1D20//ARID4B//ARID4A// |
| GO:0016310 | phosphorylation | Biological process | 176 | 2367 | 934 | 17653 | 1.40535503790973 | 1.04659496706926e-06 | 4.6874978558208e-05 | 5.98022135805355 | 0.188436830835118 | CDKN1A//GADD45A//PTEN//CCNE2//RANBP9//PPM1L//SPRED1//DUSP5//EREG//RASGEF1A//GRB2//NRG1//IL2//JAK1//MAP3K5//MET//MINK1//TAOK3//MAPK1//CCL2//SOS1//TNF//LAMTOR3//MAP3K2//CRK//CRKL//ADAM9//LPAR1//LPAR3//IGF1//NTF3//PIK3CB//PRKAA1//MAP2K4//DUSP10//DUSP1//DUSP2//PPP2CA//RGS3//DRD1//FNIP1//FAM129A//LRP6//PPP1R15B//SLIT2//EFNA1//EFNA5//EPHA7//FMR1//NPTN//KDR//P2RY1//AKTIP//SQSTM1//CCND2//FBXW7//ERRFI1//TSG101//SOCS5//NUP35//NUP43//CHCHD10//COX8C//COX7A2L//NME1//CMPK1//HIPK3//ADAM10//CDK6//CDK8//FASTK//PDIK1L//ERG//CDK19//MORC3//MMD//PAN3//DSTYK//LATS2//MKNK2//CDK17//PRKACB//FAM20C//CCL8//TGFB2//TGFBR2//RUNX3//CCNC//RPS6KA4//ACVR1//RPS6KA5//STK17B//PPM1E//FLRT1//GNAQ//NCK1//NF2//ASPN//DUS2//YWHAG//CAMK2N2//PPM1F//COPS8//PTGER4//MDFIC//CALM2//GDF11//INHBB//BMP3//BMPR2//STK33//MASTL//OSR1//EPHA2//EPHA4//EPHA5//PKDCC//PAK6//SLK//PKD2//JUN//RAP2C//OSM//PFN2//CAPRIN2//IFNA1//ADCY3//PRKAR2B//HMGA2//PTGES3//KCTD20//ITGA6//AR//BTBD10//CNOT7//STAT3//EDN1//ITGAV//FZD10//AIDA//TWF1//NRBF2//RBL2//PIK3R3//KLF4//IPO5//KAT2B//CCNY//MAPRE3//TSC1//VLDLR//ZMYND11//WNT7A//ULK2//SPHK2//DGKE//NAGK//GNPTG//PIKFYVE//ESR1//EFR3A//GAB1//ZFYVE21//ITGA5//LRP8//SKP1//PMEPA1//LDLRAD4//NOG//PFKP//LMO3//NPNT//DNAJC27//HAND2//ACSL1//DGKH// |
| GO:0009058 | biosynthetic process | Biological process | 426 | 6735 | 934 | 17653 | 1.1954836586657 | 1.05361664264141e-06 | 4.68790089091043e-05 | 5.97731737799479 | 0.45610278372591 | IGF1//NFIA//NFIB//RBBP7//RBBP8//RRM2//E2F1//NPAT//TSHZ1//HMG20A//KLF2//ZBTB18//CEBPB//ZNF268//TCERG1//WWP2//KLF12//OSR1//CRY2//GLIS3//ARX//DNMT3B//DR1//EDN1//EFNA1//CC2D1B//EN1//ESR1//FHL2//CPEB3//FOXF1//FOXC1//MYT1L//CIC//ZFPM2//DNAJB5//GATA6//CNOT7//HSBP1//AR//JARID2//JUN//ZNF568//MAF//MDM4//MYB//NR4A2//RLIM//KLF3//ZBTB7A//PIAS4//POU4F1//PPARG//CC2D1A//TSHZ3//ZBTB4//RARB//REST//RNF2//PRDM16//SOX17//SOX11//STAT3//ZEB1//TCF21//TFAP2A//TFAP2C//TGIF1//KLF10//TNF//UBE2I//VLDLR//WFS1//ZNF217//LRP8//BTG2//BHLHE41//TBL1XR1//NR4A3//HMGA2//ARID5B//BRMS1L//BHLHE40//RUNX3//URI1//PER2//SQSTM1//NOG//KLF4//HAND1//FNIP1//PHF14//USP3//GTF2E1//SECISBP2L//PTGES3//ST6GALNAC3//ST6GALNAC6//ST8SIA3//ST3GAL5//RDH10//RPL15//IRF9//KLF6//CREBRF//EGR2//ELK3//ERG//FOXJ3//FOXF2//FLI1//GRHL1//HIVEP2//IRF1//LHX1//MEF2D//MEOX2//MYBL1//NEUROD1//NHLH2//OTX2//PAX9//PBX3//POU3F2//RAD21//ARID4A//SHOX//BCL11B//BMPR2//SOX4//BTF3//TEF//TGFB1I1//THRA//TMF1//DEK//FOSL1//KLF7//CBFB//FUBP3//HAND2//CLOCK//POLR3G//SRSF2//PKD2//UGP2//HS3ST5//XYLT2//HS2ST1//HS3ST3A1//HS3ST1//NAGK//GFPT2//SLC35D1//NUP35//NUP43//NLN//HPRT1//ADCY3//IMPDH1//NME1//PDE10A//CMPK1//DCTPP1//REV3L//GMNC//CCNE2//ESCO2//PPP2CA//BAHD1//H2AFZ//KMT2B//DPY30//TOPORS//KHDRBS3//ZMYND11//TADA1//ZNF597//CTNND2//ZNF782//NKX2-3//ZNF800//E2F5//ZNF367//EZH1//ZBTB9//KDM2A//MYCBP2//FLII//CNOT6L//EPC2//LHX6//HBP1//ATAD2//SERTAD3//MDFIC//MNX1//HMGB3//HOXA3//HOXD1//FOXN2//ZBTB41//ZBTB34//ATXN3//LHX8//NEUROG1//NFE2//NPAS2//IER5//ARID4B//BRWD1//BNC2//ZNF532//CDCA7L//PRKAA1//TRERF1//LMO3//MAPK1//SLC2A4RG//PHTF2//ZNF630//ZNF287//CNOT6//ZNF512B//ZBTB26//MIER1//RBL2//RNF4//ATXN1//DMRTA1//SMARCD2//STAT4//ZNF2//ZNF3//ZNF131//ZNF655//BRD3//TCF7L1//PCGF5//LCOR//WASL//ZBTB47//ST18//HIPK3//ELAVL2//NR5A2//NRBF2//MLLT6//RNF141//PAK6//SNAPC1//BTG1//SNIP1//RPS6KA4//RPS6KA5//CDKN1A//CRK//EMX2//ETS1//BOLA3//NR3C2//PLEK//ZNFX1//NKX2-4//RFX7//BDP1//CDK8//E2F3//TEAD1//KAT2B//CCNC//CPEB2//CPEB1//CPEB4//MTPN//DDX6//MKNK2//ACO1//TSC1//KBTBD8//FUT9//SERP1//ST8SIA5//MAN1A1//MAN2A1//B3GNT5//B3GALT2//CCDC126//VEGFB//NTNG1//RECK//PIGA//GLUD2//LGSN//SERINC3//PSAT1//ELOVL4//SCD5//LCLAT1//DDHD1//CDS1//ABHD3//SLC44A1//PIKFYVE//PIK3CB//PTEN//PLEKHA1//PLEKHA3//MTMR9//PIK3R3//SPHK2//ACBD3//LBR//MSMO1//CHCHD10//G3BP2//NF2//ACSL1//RIMKLB//DGKE//BCAT2//MTRR//PPCS//EREG//SLC2A4//CNEP1R1//LDLR//FBXW7//ACOT7//FAM129A//FMR1//PRR16//FXR1//ST3GAL3//MAP3K2//MAPRE3//BAMBI//RGMB//LRP6//MAP3K5//WNT7A//ACVR1//DKK3//NRG1//RASD1//TSG101//BTAF1//PPM1F//TOB1//NANOS1//CAPRIN2//OGN//CHST1//ZDHHC1//ST6GAL2//MCFD2//ZDHHC5//ELOVL7//PPM1L//SPTSSA//SGPP1//CHST7//DSEL//SOSTDC1//TMEM100//RGMA//BMP3//TGFB2//SULF1//CTDSPL2//ADRB1//PTHLH//CALM2//EGLN1//CMKLR1//DCP2//CCT6A//EPHA5//P2RY1//ERRFI1//UXS1//GAB1//GRB2//NCK1//RNF128//ADIPOR2//QKI//INTS8//NABP1//ITM2B//FZD6//NPLOC4//ITGAV//TMEFF2//MID1IP1//MAP2K4//S1PR1//DLL1//IL2//ITGA6//MET//PPP1R12A//OSM//PPP3R1//DCAF6//PAIP2//PPT2//INHBB//KDR//CCL2//YTHDF2//EGLN3//PFKP//ATG16L1//IDH1//ORMDL3//GSTA2//RAB3GAP1//RAP2C//TOB2//PPP1R15B//DUSP1//GJA1//NSMAF//NPNT//UNK// |
| GO:0002009 | morphogenesis of an epithelium | Biological process | 55 | 528 | 934 | 17653 | 1.96879461099215 | 1.22401626988376e-06 | 5.41047191713978e-05 | 5.91221280941776 | 0.0588865096359743 | EDN1//PPP3R1//TGFBR2//ACVR1//NPNT//LHX1//PKD2//TCF21//NOG//SOX4//SOX11//DLC1//LRP6//PRKACB//TGFB2//TSC1//FZD6//GJA1//DLL1//SOX17//HAND1//HAND2//ARHGAP12//SOS1//TFAP2A//TGFB1I1//SOSTDC1//KRT27//ITGA5//WNT7A//FOXF2//TMEFF2//RHOC//FOXF1//MSN//KLF4//CLIC4//MET//SLIT2//FRZB//VANGL1//RDH10//EPHA2//AR//ESR1//NTN4//GRB2//SULF1//BTBD7//TNF//KLHL3//OSR1//EPHA4//EPHA7//PTEN// |
| GO:0007611 | learning or memory | Biological process | 32 | 240 | 934 | 17653 | 2.52005710206995 | 1.38923238977512e-06 | 6.10089522860334e-05 | 5.85722509972305 | 0.0342612419700214 | DRD1//CIC//SLC24A2//JUN//PRKAR2B//SLC12A5//ATXN1//SLC6A1//FOSL1//CEBPB//ITGA5//NTF3//PAIP2//KCNK10//PTEN//VLDLR//KMT2B//CPEB3//GRIA1//LDLR//SLC17A7//CCND2//SNAP25//BTG2//ADCY3//NPTN//EN1//NEUROG1//EGR2//LHX8//MAPK1//THRA// |
| GO:0044271 | cellular nitrogen compound biosynthetic process | Biological process | 348 | 5342 | 934 | 17653 | 1.23125215956619 | 1.53162607733236e-06 | 6.68283042645081e-05 | 5.81484724800133 | 0.372591006423983 | E2F1//NPAT//RRM2//TSHZ1//HMG20A//KLF2//ZBTB18//CEBPB//ZNF268//TCERG1//WWP2//KLF12//OSR1//CRY2//GLIS3//ARX//DNMT3B//DR1//EDN1//EFNA1//CC2D1B//EN1//ESR1//FHL2//CPEB3//FOXF1//FOXC1//MYT1L//CIC//ZFPM2//DNAJB5//GATA6//CNOT7//HSBP1//AR//JARID2//JUN//ZNF568//MAF//MDM4//MYB//NFIA//NFIB//NR4A2//RLIM//KLF3//ZBTB7A//PIAS4//POU4F1//PPARG//CC2D1A//TSHZ3//ZBTB4//RARB//RBBP7//RBBP8//REST//RNF2//PRDM16//SOX17//SOX11//STAT3//ZEB1//TCF21//TFAP2A//TFAP2C//TGIF1//KLF10//TNF//UBE2I//VLDLR//WFS1//ZNF217//LRP8//BTG2//BHLHE41//TBL1XR1//NR4A3//HMGA2//ARID5B//BRMS1L//BHLHE40//RUNX3//URI1//PER2//SQSTM1//NOG//KLF4//HAND1//FNIP1//PHF14//USP3//GTF2E1//SECISBP2L//ST6GALNAC3//ST6GALNAC6//ST8SIA3//ST3GAL5//RPL15//IRF9//KLF6//CREBRF//EGR2//ELK3//ERG//FOXJ3//FOXF2//FLI1//GRHL1//HIVEP2//IRF1//LHX1//MEF2D//MEOX2//MYBL1//NEUROD1//NHLH2//OTX2//PAX9//PBX3//POU3F2//RAD21//ARID4A//SHOX//BCL11B//BMPR2//SOX4//BTF3//TEF//TGFB1I1//THRA//TMF1//DEK//FOSL1//KLF7//CBFB//FUBP3//HAND2//CLOCK//POLR3G//SRSF2//PKD2//NAGK//GFPT2//SLC35D1//UGP2//NUP35//NUP43//HPRT1//ADCY3//IMPDH1//NME1//PDE10A//CMPK1//DCTPP1//BAHD1//H2AFZ//KMT2B//DPY30//TOPORS//KHDRBS3//ZMYND11//TADA1//ZNF597//CTNND2//ZNF782//NKX2-3//ZNF800//E2F5//ZNF367//EZH1//ZBTB9//KDM2A//MYCBP2//FLII//CNOT6L//EPC2//LHX6//HBP1//ATAD2//SERTAD3//MDFIC//MNX1//HMGB3//HOXA3//HOXD1//FOXN2//ZBTB41//ZBTB34//ATXN3//LHX8//NEUROG1//NFE2//NPAS2//IER5//ARID4B//BRWD1//BNC2//ZNF532//CDCA7L//PRKAA1//TRERF1//LMO3//MAPK1//SLC2A4RG//PHTF2//ZNF630//ZNF287//CNOT6//ZNF512B//ZBTB26//MIER1//RBL2//RNF4//ATXN1//DMRTA1//SMARCD2//STAT4//ZNF2//ZNF3//ZNF131//ZNF655//BRD3//TCF7L1//PCGF5//LCOR//WASL//ZBTB47//ST18//HIPK3//ELAVL2//NR5A2//NRBF2//MLLT6//RNF141//PPP2CA//PAK6//SNAPC1//BTG1//SNIP1//RPS6KA4//RPS6KA5//CDKN1A//CRK//EMX2//ETS1//BOLA3//NR3C2//PLEK//ZNFX1//NKX2-4//RFX7//BDP1//CDK8//E2F3//TEAD1//KAT2B//CCNC//CPEB2//CPEB1//CPEB4//MTPN//DDX6//MKNK2//ACO1//TSC1//KBTBD8//B3GALT2//CHCHD10//PTGES3//G3BP2//PSAT1//EREG//ACOT7//PPCS//FAM129A//FMR1//SERP1//PRR16//FXR1//MAP3K2//MAPRE3//BAMBI//RGMB//IGF1//LRP6//MAP3K5//WNT7A//ACVR1//DKK3//NRG1//RASD1//TSG101//BTAF1//PPM1F//TOB1//NANOS1//CAPRIN2//SOSTDC1//TMEM100//RGMA//BMP3//TGFB2//SULF1//CTDSPL2//ADRB1//PTHLH//CALM2//EGLN1//CMKLR1//DCP2//CCT6A//EPHA5//UXS1//NCK1//REV3L//INTS8//NABP1//FZD6//NPLOC4//S1PR1//DLL1//IL2//ITGA6//MET//PPP1R12A//OSM//P2RY1//PPP3R1//DCAF6//PAIP2//SPHK2//SPTSSA//PTEN//YTHDF2//QKI//EGLN3//PFKP//ORMDL3//RAP2C//TOB2//PPP1R15B//DUSP1//GJA1//NSMAF//FBXW7//NPNT//UNK// |
| GO:0043067 | regulation of programmed cell death | Biological process | 126 | 1579 | 934 | 17653 | 1.50820390212546 | 1.54530997360105e-06 | 6.69931496888712e-05 | 5.81098439240622 | 0.134903640256959 | PTEN//IL2//FNIP1//DLC1//EGLN3//F3//PMAIP1//PPARG//TNF//HIPK3//TOPORS//CDKN1A//ZNF268//OSR1//DUSP1//EPHA2//EPHA4//EPHA5//EPHA7//FHL2//ARL6IP1//DSTYK//GATA6//IGF1//KDR//MDM4//MET//ZFAND6//PRKAA1//SPHK2//RARB//BCL11B//BNIP3//STAT3//ACTC1//TFAP2A//TMF1//WNT7A//BAG6//HMGA2//SQSTM1//CCND2//MAP3K5//HAND2//BCL2L11//GDF11//MAP3K2//ESR1//TNFRSF21//RASSF3//INHBB//NF2//NME1//NTF3//MINK1//TAOK3//PAK6//RBM25//MOAP1//MAP2K4//BMP3//TGFB2//ANP32E//SLK//PIK3CB//THRA//DLL1//CCL2//EDN1//LRP6//FGD4//GADD45A//E2F1//LPAR1//CDK19//FRZB//LATS2//ITGA6//JUN//USP27X//NEUROD1//ARHGEF4//POU4F1//REST//ZMAT3//UBE2Z//SOS1//SOS2//SOX4//FOSL1//NSMAF//SLIT2//WFS1//KLF4//PPM1F//NSMF//CEBPB//EN1//NR4A2//BTG2//NR4A3//CPEB4//MYB//EFNA1//DYNLL2//PPP3R1//YWHAG//PIAS4//BAG5//SERINC3//NCK1//FOXC1//FBXW7//CD274//E2F3//IER3IP1//BTG1//STK17B//ITGA5//NOG//ZMYND11//AR//ITGAV//ACVR1//URI1//ST18// |
| GO:0042221 | response to chemical | Biological process | 309 | 4655 | 934 | 17653 | 1.25461489453214 | 1.69529843288173e-06 | 7.30274095641983e-05 | 5.77075383944008 | 0.330835117773019 | CBFB//DRD1//HPRT1//NR4A2//ARL6IP1//THRA//CCL2//CCL8//SLIT2//DNAJB4//UBXN4//DNAJB5//JKAMP//HERPUD2//HSPA13//CALM2//PTGES3//UGT1A6//UGT1A9//SLC17A7//SLC17A6//CMKLR1//S1PR1//PIK3CB//MAPK1//FOSL1//CXCL14//COL1A2//JUN//TGFB2//TGFBR2//KLF10//ADAM9//ACVR1//ZFYVE9//CHRM2//CHRM5//GNAQ//RANBP9//ARX//DPYSL2//EFNA1//EFNA5//EPHA5//GAB1//GRB2//KIF5A//OTX2//ENAH//NTN4//SOS1//VLDLR//NR4A3//KLF7//RPS6KA5//FEZ2//ACSL1//UGT1A1//PPARG//ADIPOR2//EGR2//EPHA4//LHX1//NOG//APPL1//PIK3R3//ADCY3//SPRED1//FLRT1//ESRP1//MAP3K5//CDKN1A//EMX2//GATA6//NEUROD1//SLC12A5//TGIF1//VEGFB//LRP8//SLC6A14//DNMT3B//PON2//SLC6A1//SNN//FHL2//STEAP2//RAP1B//GJA1//IGF1//PTEN//NR5A2//LATS2//RARB//PPP2CA//CYBRD1//ACO1//FIBIN//EDN1//SNIP1//TNF//PIAS4//ADAM10//BTG2//NME1//SLC32A1//SLC25A32//DKK3//SOX11//ZEB1//RNF4//GJB2//JAK1//CRK//CRKL//EREG//F3//IFNA1//IL2//MMP2//OSM//ASPN//STAT3//STAT4//SOCS5//GPR180//NRG1//CEBPB//SESN3//CPEB3//NSMF//SESN1//GLRA1//IPO5//RRAGD//CPEB1//CPEB4//LAMTOR3//DNAJB9//DERL2//NPLOC4//WFS1//BAG6//EDEM1//TOB1//SOSTDC1//RGMB//TMEM100//RGMA//BMP3//BMPR2//NPNT//TGFB1I1//BAMBI//PMEPA1//PRDM16//RASL11B//LDLRAD4//SULF1//CTDSPL2//ESR1//AR//ITGA5//CREBRF//SERP1//PRKAA1//EPHA7//TMEM135//DUSP1//ETS1//RBBP8//WNT7A//DUSP10//TNFRSF21//PTGER4//TSC1//CPEB2//INHBB//ERRFI1//SLC2A4//YWHAG//KAT2B//UBA5//STX2//SRSF2//TRPC3//ST18//CD274//CALCOCO2//VKORC1L1//PKD2//IRF1//PPM1E//PPP1R12A//REST//PPM1F//CD69//GSTA2//MSN//SYBU//KDR//DNAJB12//FOXC1//BRWD1//NTF3//FAM20C//SHISA2//PPP1R15B//GLRB//PMAIP1//PGRMC2//NR3C2//LRP6//BTG1//DSTYK//RPL15//ACTC1//EPS8//USP46//NPTN//NCK1//IDH1//RBBP7//SEMA4B//OR6A2//MET//ITGAV//TRPV6//EPHA2//P2RY1//KCNJ10//PER2//UGT1A10//UGT1A8//UGT1A7//UGT1A5//UGT1A4//UGT1A3//LPAR1//IRF9//CNOT7//KLF4//TCF21//EGLN3//EGLN1//KLF2//MYB//PLEKHA1//BNIP3//SKP1//SQSTM1//RPS6KA4//ATXN3//UBE2W//CDK19//PLSCR4//PDCD1LG2//PLAA//MKNK2//CLIC4//TFAP2A//SLC41A1//FZD10//OSR1//POU3F2//HCN2//SOX4//FOXF1//POU4F1//PTPN4//RAB12//ZFAND6//PAX9//URI1//PRKACB//PRKAR2B//BCL2L11//H2AFZ//E2F1//LDLR//ITGA6//TMF1//MDM4//RUNX3//KLHL15//BCL11B//NFIB//MAP2K4//FMR1//MPP1//DLL1//KLF3//BAG5//FBXW7//STMN2//DYNC1LI2//PCOLCE2//JARID2//NEFH//PFKP//PIGA//BCAT2//SOX17//ARID5B//GFPT2//LRCH1//CRY2//CLOCK//LMO3//HMGA2// |
| GO:0042327 | positive regulation of phosphorylation | Biological process | 90 | 1032 | 934 | 17653 | 1.6482931626911 | 1.71590321342796e-06 | 7.34186668779312e-05 | 5.7655072124428 | 0.0963597430406852 | GADD45A//MINK1//TNF//MAP3K2//CRK//CRKL//MAP3K5//TAOK3//LAMTOR3//ADAM9//DUSP5//LPAR1//LPAR3//NRG1//IGF1//NTF3//PIK3CB//PRKAA1//MAPK1//MAP2K4//FAM129A//EFNA1//EFNA5//EPHA7//FMR1//NPTN//KDR//P2RY1//AKTIP//SQSTM1//CCND2//FNIP1//EREG//FBXW7//COPS8//MDFIC//CALM2//GDF11//INHBB//BMP3//BMPR2//TGFB2//ACVR1//PKD2//RAP2C//PAK6//TGFBR2//SLK//RPS6KA4//RPS6KA5//OSM//PFN2//CAPRIN2//IFNA1//EPHA2//EPHA4//EPHA5//DSTYK//MET//ADCY3//PRKACB//PRKAR2B//IL2//STAT3//EDN1//AR//ITGAV//FZD10//CCNY//MAPRE3//CCNC//CDKN1A//MMD//VLDLR//WNT7A//ITGA5//LRP8//NPNT//JUN//DNAJC27//PTEN//CCL2//CCL8//HAND2//ACSL1//PPP2CA//PTGES3//KCTD20//ITGA6//BTBD10// |
| GO:0048568 | embryonic organ development | Biological process | 47 | 428 | 934 | 17653 | 2.0755143188777 | 1.72609315889266e-06 | 7.34186668779312e-05 | 5.76293576870122 | 0.0503211991434689 | CEBPB//PKD2//GJA1//DLL1//SOX17//TGFBR2//HAND1//HAND2//ACVR1//TFAP2A//EFNA1//RDH10//LRIG1//FOXC1//OSR1//KDR//NEUROG1//SOBP//FZD6//MAPK1//TSHZ1//EDN1//NOG//RARB//FOXF1//SOX11//TCF21//NEUROD1//FOXF2//TGFB2//TNF//PKDCC//LRP6//ZEB1//LHX1//HOXA3//DSCAML1//NR4A3//ZNF568//GRB2//EGLN1//VASH2//FRZB//EN1//ZFPM2//EPN1//TEAD1// |
| GO:0022603 | regulation of anatomical structure morphogenesis | Biological process | 96 | 1123 | 934 | 17653 | 1.61570891673229 | 1.81358853944062e-06 | 7.66581205764807e-05 | 5.74146123729785 | 0.102783725910064 | BMPR2//HAND2//DLC1//CDC42EP3//WASF3//PALM2//FGD4//CRK//FGD5//LPAR1//EPS8//BAMBI//KDR//RHOC//RND3//MSN//BRWD1//CCL2//WASL//BNIP3//DLL1//SPRED1//FOXC1//SULF1//PPARG//TGFB2//KLF4//SH3D19//EFNA5//DPYSL2//RAB21//EPHA4//WNT7A//ADAM9//PAX9//EDN1//SOX17//EFNA1//EPHA2//ETS1//EGLN1//VASH2//F3//GAB1//GATA6//ITGB8//STAT3//BTG1//TGFBR2//VEGFB//ADAM12//HMGA2//GOLGA4//NRG1//EPHA7//ULK2//LPAR3//FMR1//NSMF//HECW2//FXR1//SEMA4B//POU3F2//PTEN//SLIT2//CAPRIN2//VANGL1//FZD6//AR//NTN4//ESR1//BTBD7//TNF//LRP8//ASPN//TFAP2A//MEOX2//TMEM135//MIEF1//DDHD1//LHX1//NOG//CRKL//DOCK5//KLF2//EPN1//ITGA5//JAK1//PLAA//ACVR1//FBXW7//ERG//OSR1//OTX2//NFIB//TMEM100// |
| GO:0120036 | plasma membrane bounded cell projection organization | Biological process | 117 | 1444 | 934 | 17653 | 1.53140589132021 | 1.83560429800691e-06 | 7.71067817852219e-05 | 5.73622093408999 | 0.125267665952891 | CD2AP//NCK1//NTNG1//SLITRK3//NEFH//OGN//POU4F1//DSCAML1//WNT7A//SLIT2//RANBP9//ARX//DPYSL2//EFNA1//EFNA5//EPHA5//GAB1//GRB2//KIF5A//OTX2//PIK3CB//ENAH//MAPK1//NTN4//SOS1//VLDLR//NR4A3//KLF7//RPS6KA5//FEZ2//EGR2//EPHA4//LHX1//NOG//PTPDC1//DYNLL2//TMEM17//DZIP1//RAB23//GMNC//CDC14A//C5ORF30//CEP41//EPHA2//TWF1//PTEN//SNAP25//ZNF804A//STMN2//NPTN//NME1//SERPINI1//LPAR1//MYLIP//PTPRG//SPOCK1//TSC1//BAG5//FLRT1//STRN//NR4A2//NFIB//WASF3//S1PR1//ARHGEF4//RAB21//JUN//FRY//GPM6A//LRP12//PSD//BTG2//CDC42EP3//EPHA7//LIMA1//PLEK//PLEKHA1//LPAR3//CEP97//GOLGA4//NRG1//FGD4//FGD5//ITGA6//ULK2//KLF4//RGMA//GJA1//MNX1//NTF3//MINK1//HPRT1//FMR1//NSMF//HECW2//FXR1//BMPR2//SEMA4B//POU3F2//CAPRIN2//WASL//SLC12A5//CTNND2//CPEB3//IL2//C21ORF91//LRP8//NEFM//BCL11B//SNX2//NEDD1//PRKAR2B//YWHAG//HAUS8//PFN2//EPS8//PLAA// |
| GO:0010941 | regulation of cell death | Biological process | 133 | 1696 | 934 | 17653 | 1.4821680184639 | 1.92636220542166e-06 | 8.04196765139919e-05 | 5.7152620510503 | 0.142398286937901 | PTEN//IL2//FNIP1//DLC1//EGLN3//F3//PMAIP1//PPARG//TNF//HIPK3//TOPORS//CDKN1A//ZNF268//OSR1//DUSP1//EPHA2//EPHA4//EPHA5//EPHA7//FHL2//ARL6IP1//DSTYK//GATA6//IGF1//KDR//MDM4//MET//ZFAND6//PRKAA1//SPHK2//RARB//BCL11B//BNIP3//STAT3//ACTC1//TFAP2A//TMF1//WNT7A//BAG6//HMGA2//SQSTM1//CCND2//MAP3K5//HAND2//BCL2L11//GDF11//MAP3K2//ESR1//TNFRSF21//RASSF3//INHBB//NF2//NME1//NTF3//MINK1//TAOK3//PAK6//RBM25//MOAP1//MAP2K4//BMP3//TGFB2//ANP32E//SLK//PIK3CB//THRA//DLL1//CCL2//EDN1//LRP6//FGD4//GADD45A//E2F1//LPAR1//CDK19//FRZB//LATS2//ITGA6//JUN//USP27X//NEUROD1//ARHGEF4//POU4F1//REST//ZMAT3//UBE2Z//SOS1//SOS2//SOX4//FOSL1//NSMAF//SLIT2//WFS1//KLF4//PPM1F//NSMF//CEBPB//EN1//NR4A2//BTG2//NR4A3//CPEB4//MYB//ZFPM2//NCK1//NPAS2//DUS2//SOX11//EFNA1//DYNLL2//PPP3R1//YWHAG//EGLN1//TSC1//BTBD10//PIAS4//BAG5//SERINC3//FOXC1//FBXW7//CD274//E2F3//IER3IP1//BTG1//STK17B//ITGA5//NOG//ZMYND11//AR//ITGAV//ACVR1//URI1//ST18// |
| GO:0006063 | uronic acid metabolic process | Biological process | 9 | 24 | 934 | 17653 | 7.08766059957173 | 1.9883212569404e-06 | 8.19940040285849e-05 | 5.70151344446355 | 0.00963597430406852 | UGT1A10//UGT1A7//UGT1A6//UGT1A9//UGT1A4//UGT1A1//UGT1A3//UGT1A8//UGT1A5// |
| GO:0019585 | glucuronate metabolic process | Biological process | 9 | 24 | 934 | 17653 | 7.08766059957173 | 1.9883212569404e-06 | 8.19940040285849e-05 | 5.70151344446355 | 0.00963597430406852 | UGT1A10//UGT1A7//UGT1A6//UGT1A9//UGT1A4//UGT1A1//UGT1A3//UGT1A8//UGT1A5// |
| GO:0050767 | regulation of neurogenesis | Biological process | 69 | 732 | 934 | 17653 | 1.78159774634044 | 2.15570032649113e-06 | 8.83575836852092e-05 | 5.6664116125006 | 0.0738758029978587 | PTEN//SNAP25//ZNF804A//STMN2//NPTN//NCK1//NME1//SERPINI1//TWF1//LPAR1//EPHA4//MYLIP//PTPRG//SPOCK1//TSC1//BAG5//NF2//DPYSL2//RAB21//WNT7A//DLL1//NTF3//BCL11B//YWHAG//HMG20A//DDX6//REST//DNMT3B//MMD//NEUROD1//NEUROG1//RARB//SOX11//ZEB1//GOLGA4//NRG1//EPHA7//ULK2//EFNA5//LPAR3//KLF4//RGMA//NOG//TNFRSF21//PPARG//RHEB//DUSP10//FMR1//NSMF//HECW2//FXR1//BMPR2//SEMA4B//MAN2A1//EFNA1//POU3F2//SLIT2//CAPRIN2//MYB//CPEB3//IL2//C21ORF91//LRP8//LDLR//VLDLR//PLAA//STAT3//POU4F1//PER2// |
| GO:0044238 | primary metabolic process | Biological process | 643 | 10905 | 934 | 17653 | 1.11444065793052 | 2.2085539595004e-06 | 8.95463099162822e-05 | 5.65589198557154 | 0.688436830835118 | ACOX1//IGF1//NFIA//NFIB//RBBP7//RBBP8//RRM2//CDKN1A//GADD45A//PTEN//CCNE2//E2F1//NPAT//MTRR//TSHZ1//HMG20A//KLF2//ZBTB18//CEBPB//ZNF268//TCERG1//WWP2//KLF12//OSR1//CRY2//GLIS3//ARX//DNMT3B//DR1//EDN1//EFNA1//CC2D1B//EN1//ESR1//FHL2//CPEB3//FOXF1//FOXC1//MYT1L//CIC//ZFPM2//DNAJB5//GATA6//CNOT7//HSBP1//AR//JARID2//JUN//ZNF568//MAF//MDM4//MYB//NR4A2//RLIM//KLF3//ZBTB7A//PIAS4//POU4F1//PPARG//CC2D1A//TSHZ3//ZBTB4//RARB//REST//RNF2//PRDM16//SOX17//SOX11//STAT3//ZEB1//TCF21//TFAP2A//TFAP2C//TGIF1//KLF10//TNF//UBE2I//VLDLR//WFS1//ZNF217//LRP8//BTG2//BHLHE41//TBL1XR1//NR4A3//HMGA2//ARID5B//BRMS1L//BHLHE40//RUNX3//URI1//PER2//SQSTM1//NOG//KLF4//HAND1//FNIP1//PHF14//USP3//RANBP9//PPM1L//SPRED1//DUSP5//EREG//RASGEF1A//GRB2//NRG1//IL2//JAK1//MAP3K5//MET//MINK1//TAOK3//MAPK1//CCL2//SOS1//LAMTOR3//DCP2//PPP2CA//RPL15//MAP3K2//CRK//CRKL//ADAM9//LPAR1//LPAR3//NTF3//PIK3CB//PRKAA1//MAP2K4//DUSP10//DUSP1//DUSP2//RGS3//TOPORS//HECTD2//RNF145//ZNRF2//FBXL5//RNF180//MYLIP//ANKIB1//UBE2W//FBXW7//HECW2//SKP1//THOP1//LONRF3//FBXO30//CBFB//UBE3B//LONRF1//ZFAND4//CNOT6L//PAN3//CNOT6//COPS8//RBFOX3//FMR1//MBNL1//RBM25//SRSF2//ELAVL2//NOVA1//PTGES3//NABP1//GTF2E1//SECISBP2L//RDH10//NAPEPLD//TTR//ITGB8//ST6GALNAC3//ST6GALNAC6//ST8SIA3//ST3GAL5//ACSL1//DRD1//FAM129A//LRP6//PPP1R15B//SLIT2//EFNA5//EPHA7//NPTN//KDR//P2RY1//AKTIP//CCND2//LYPLA1//F3//DUS2//IRF9//KLF6//CREBRF//EGR2//ELK3//ERG//FOXJ3//FOXF2//FLI1//GRHL1//HIVEP2//IRF1//LHX1//MEF2D//MEOX2//MYBL1//NEUROD1//NHLH2//OTX2//PAX9//PBX3//POU3F2//RAD21//ARID4A//SHOX//BCL11B//BMPR2//SOX4//BTF3//TEF//TGFB1I1//THRA//TMF1//DEK//FOSL1//KLF7//FUBP3//HAND2//CLOCK//POLR3G//PKD2//ERRFI1//TSG101//SOCS5//TRIM3//KLHL2//RNF38//MYCBP2//FBXO33//KLHL3//SPOPL//DCAF8//DCAF6//FEM1C//KLHL14//MARCH4//RNF4//MARCH7//UBE2Z//RNF128//KLHL15//KBTBD8//ARRDC4//FUT9//ST8SIA5//UEVLD//CASD1//SLC2A4//CHST7//CHST1//UGP2//SERP1//MAN2A1//GFPT2//NAGK//SLC35D1//IDH1//ACO1//NUP35//NUP43//NLN//CHCHD10//COX8C//COX7A2L//DPYSL2//HPRT1//NME1//CMPK1//ADCY3//IMPDH1//PDE10A//DCTPP1//REV3L//GMNC//ESCO2//EPC2//USP28//USP45//ATXN3//KDM2A//HMGB3//BAHD1//H2AFZ//KMT2B//DPY30//ARID4B//KHDRBS3//ZMYND11//TADA1//ZNF597//CTNND2//ZNF782//NKX2-3//ZNF800//E2F5//ZNF367//EZH1//ZBTB9//FLII//LHX6//HBP1//ATAD2//SERTAD3//MDFIC//MNX1//HOXA3//HOXD1//FOXN2//ZBTB41//ZBTB34//LHX8//NEUROG1//NFE2//NPAS2//IER5//BRWD1//BNC2//ZNF532//CDCA7L//TRERF1//LMO3//SLC2A4RG//PHTF2//ZNF630//ZNF287//ZNF512B//ZBTB26//MIER1//RBL2//ATXN1//DMRTA1//SMARCD2//STAT4//ZNF2//ZNF3//ZNF131//ZNF655//BRD3//TCF7L1//PCGF5//LCOR//WASL//ZBTB47//ST18//HIPK3//NR5A2//NRBF2//MLLT6//RNF141//PAK6//SNAPC1//BTG1//SNIP1//RPS6KA4//RPS6KA5//EMX2//ETS1//BOLA3//NR3C2//PLEK//ZNFX1//NKX2-4//RFX7//BDP1//CDK8//E2F3//TEAD1//KAT2B//CCNC//CMTR2//ZFC3H1//RTCA//PPP4R2//ESRP1//CPEB1//QKI//CPEB2//CPEB4//MTPN//DDX6//MKNK2//TSC1//RIMKLB//IMMP2L//ADAM10//CDK6//FASTK//PDIK1L//CDK19//MORC3//MMD//DSTYK//LATS2//CDK17//PRKACB//FAM20C//CCL8//TGFB2//TGFBR2//ACVR1//STK17B//PPM1E//FLRT1//GNAQ//NCK1//NF2//ASPN//YWHAG//CAMK2N2//PPM1F//PTPN21//PPP1R12A//CTDSPL2//PPP3R1//PPP6C//PTPN4//NAA30//HS3ST5//PCMTD1//ASPH//MAN1A1//B3GNT5//B3GALT2//CCDC126//EDEM1//VEGFB//NTNG1//RECK//PTAR1//PIGA//MMP2//CTSA//NPEPL1//ADAM12//ADAM18//LMLN//ZFYVE9//USP24//USP27X//NPLOC4//USP46//BAG6//CDC23//SLC6A14//SLC6A6//GLUD2//LGSN//SLC25A32//BCAT2//SERINC3//PSAT1//CNEP1R1//LDLR//MID1IP1//MSMO1//ACOT7//UGT1A8//PRKAR2B//ELOVL4//SCD5//PLAA//LCLAT1//DDHD1//CDS1//ABHD3//SLC44A1//PIKFYVE//PLEKHA1//PLEKHA3//MTMR9//PIK3R3//KDELC1//SGPP1//SPHK2//NSMAF//ORMDL3//ZADH2//ACBD3//LBR//DLC1//EGLN3//PMAIP1//DLL1//G3BP2//PTGER4//UGT1A1//OSBPL5//EPHA2//PTHLH//DGKE//ACBD5//ST3GAL3//ST6GAL2//CALM2//GDF11//INHBB//BMP3//SERPINI1//SPOCK1//PCOLCE2//XYLT2//HS2ST1//PPCS//GLRX//PRR16//FXR1//INTS8//GNPTG//RBM47//MAPRE3//BAMBI//RGMB//WNT7A//DKK3//RASD1//BTAF1//DMWD//OTUD3//WDR20//JOSD1//TOB1//NANOS1//CAPRIN2//VKORC1L1//CEP41//STK33//MASTL//EPHA4//EPHA5//PKDCC//TMX3//ZDHHC1//MCFD2//ZDHHC5//CRTAP//EGLN1//RGL1//TNFRSF21//UGT1A9//ELOVL7//PON2//ADIPOR2//SLK//SPTSSA//SULF1//DSEL//UBXN4//DNAJB9//DERL2//JKAMP//SOSTDC1//TMEM100//RGMA//ADRB1//BAG5//NDFIP2//RAP2C//CMKLR1//CCT6A//PPP1R37//OSM//PFN2//IFNA1//UXS1//NSMF//PTPDC1//PTPRG//PTP4A1//CDC14A//BNIP3//GAB1//DNAJB12//UGT1A7//UGT1A3//ITM2B//TDRD5//ARL6IP1//UBXN2B//CD2AP//VPS37A//BCL2L11//ITGAV//FZD6//AHNAK//YTHDF2//FZD10//AIDA//TWF1//CKAP4//RAB38//MIA3//ANO8//CCDC71L//FAM135A//RAB12//GJA1//NSF//IPO5//CCNY//S1PR1//ITGA6//PAIP2//MPP1//RAB23//ENPP4//DGKH//ULK2//EFR3A//ZFYVE21//ITGA5//SH3D19//ARRDC3//UGT1A10//UGT1A6//UGT1A4//UGT1A5//CHRM5//PMEPA1//LDLRAD4//PFKP//ATG16L1//NPNT//DNAJC27//UBA5//REXO1//ZC3H12C//RAB3GAP1//MSN//TOB2//FRY//UNK// |
| GO:0009896 | positive regulation of catabolic process | Biological process | 44 | 393 | 934 | 17653 | 2.11607848265416 | 2.2111834623716e-06 | 8.95463099162822e-05 | 5.65537522241896 | 0.0471092077087794 | PIK3CB//PRKAA1//BNIP3//SESN3//RAB12//SESN1//KDR//TSC1//RNF180//ANKIB1//LONRF3//LONRF1//SOCS5//WWP2//GJA1//MYLIP//NSF//HECW2//SOX17//TNF//IGF1//SH3D19//ADAM9//TOB1//CPEB3//CNOT7//BTG2//CNOT6L//YTHDF2//QKI//NANOS1//CALCOCO2//FYCO1//FMR1//FBXW7//FBXL5//MSN//EDEM1//ATXN3//BAG6//RNF128//LDLR//PTEN//RAB3GAP1// |
| GO:0010646 | regulation of cell communication | Biological process | 243 | 3522 | 934 | 17653 | 1.30403295528747 | 2.27648993781696e-06 | 9.16422705324768e-05 | 5.64273426495176 | 0.26017130620985 | GADD45A//MINK1//TNF//MAP3K2//CRK//CRKL//MAP3K5//TAOK3//LAMTOR3//ADAM9//DUSP5//LPAR1//LPAR3//NRG1//IGF1//NTF3//PIK3CB//PRKAA1//MAPK1//MAP2K4//DUSP10//SPRED1//DUSP1//DUSP2//PPP2CA//RGS3//CBFB//THRA//SOX17//EREG//FBXW7//ERRFI1//TSG101//SOCS5//NCK1//COPS8//MDFIC//RAPGEF4//MARCKS//NEUROD1//SNAP25//PER2//CLOCK//GIT2//OTX2//YWHAG//TOB1//EPS8//ARHGAP1//ARHGAP6//EPHA2//EPHA4//EPHA5//EPHA7//RGS17//KDR//MET//ACVR1//GDF11//EDN1//IFNA1//IL2//INHBB//OGN//OSM//P2RY1//PTHLH//CCL2//CCL8//BMP3//TGFB2//TTR//VEGFB//WNT7A//CXCL14//F3//GJA1//PDE10A//AKAP11//PIAS4//BMPR2//PMEPA1//LDLRAD4//PTEN//KLF4//PLEK//DKK3//SOX11//ZEB1//ESR1//TCF7L1//SULF1//SOX4//APCDD1//FRZB//SOSTDC1//SHISA2//NPNT//TGFB1I1//BAMBI//ASPN//PRDM16//RASL11B//TGFBR2//GATA6//CTDSPL2//NOG//ITGA5//PKD2//TSC1//FNIP1//RRAGD//RHEB//ARAP2//APPL1//AGFG1//AGFG2//PSD//SERP1//USP46//SLC6A1//RALGPS2//UBA5//AR//IRF1//FGD4//FGD5//ARHGEF4//ARHGEF28//SOS1//SOS2//DLC1//NF2//LMO3//SYBU//NPLOC4//FAM20C//EPS15//GRB2//EPN1//CNOT7//STAT3//ZFAND6//SQSTM1//RHOC//NDFIP2//CC2D1A//MIER1//SLC20A1//UBE2I//ZMYND11//PAK6//EFNA1//ITGAV//FZD10//HIPK3//AIDA//PMAIP1//PEX5L//ADRB1//DSTYK//NPTN//WWP2//YTHDF2//DLL1//KAT2B//FLRT1//RASGEF1A//SESN3//REST//FMR1//CPEB3//EGR2//NSMF//KCNJ10//RAB3GAP1//GRIA1//PXK//LRP8//CMKLR1//TMEM100//FAM13A//TAGAP//TRIP10//ARHGAP12//CD2AP//SLIT2//DRD1//GAB1//OTUD3//PLEKHA1//TSHZ3//GLRA1//SLC24A2//CALM2//JAK1//PPARG//TCF21//CTNND2//AMER2//CCNY//LRP6//EFNA5//RANBP9//JUN//DNAJC27//HAND2//ARRDC3//TMEM64//LATS2//FZD6//BCL2L11//MOAP1//BNIP3//CAPRIN2//TBL1XR1//FXR1//LYPD6//CREBRF//PAIP2//DYNLL2//E2F1//PPP3R1//PRKACB//MDM4//POU4F1//RBBP7//RBBP8//BAG5//WFS1//SERINC3//DNAJB9//PPP1R15B//SESN1//PFN2//CRY2//PHF14//MYB//TMF1//NR4A2//ITGA6//URI1//ST18// |
| GO:0023051 | regulation of signaling | Biological process | 246 | 3575 | 934 | 17653 | 1.30056093799134 | 2.35705599799332e-06 | 9.4069370506532e-05 | 5.62763009955858 | 0.26338329764454 | GADD45A//MINK1//TNF//MAP3K2//CRK//CRKL//MAP3K5//TAOK3//LAMTOR3//ADAM9//DUSP5//LPAR1//LPAR3//NRG1//IGF1//NTF3//PIK3CB//PRKAA1//MAPK1//MAP2K4//DUSP10//SPRED1//DUSP1//DUSP2//PPP2CA//RGS3//CAMLG//EPS15//NSF//CBFB//THRA//SOX17//EREG//FBXW7//ERRFI1//TSG101//SOCS5//NCK1//COPS8//MDFIC//RAPGEF4//MARCKS//NEUROD1//SNAP25//PER2//CLOCK//GIT2//OTX2//YWHAG//TOB1//EPS8//ARHGAP1//ARHGAP6//EPHA2//EPHA4//EPHA5//EPHA7//RGS17//KDR//MET//ACVR1//GDF11//EDN1//IFNA1//IL2//INHBB//OGN//OSM//P2RY1//PTHLH//CCL2//CCL8//BMP3//TGFB2//TTR//VEGFB//WNT7A//CXCL14//F3//PDE10A//AKAP11//PIAS4//BMPR2//PMEPA1//LDLRAD4//PTEN//KLF4//PLEK//DKK3//SOX11//ZEB1//ESR1//TCF7L1//SULF1//SOX4//APCDD1//FRZB//SOSTDC1//SHISA2//NPNT//TGFB1I1//BAMBI//ASPN//PRDM16//RASL11B//TGFBR2//GATA6//CTDSPL2//NOG//ITGA5//PKD2//TSC1//FNIP1//RRAGD//RHEB//ARAP2//APPL1//AGFG1//AGFG2//PSD//GJA1//SERP1//USP46//SLC6A1//RALGPS2//UBA5//AR//IRF1//FGD4//FGD5//ARHGEF4//ARHGEF28//SOS1//SOS2//DLC1//NF2//LMO3//SYBU//NPLOC4//FAM20C//GRB2//EPN1//CNOT7//STAT3//ZFAND6//SQSTM1//RHOC//NDFIP2//CC2D1A//MIER1//SLC20A1//UBE2I//ZMYND11//PAK6//EFNA1//ITGAV//FZD10//HIPK3//AIDA//PMAIP1//PEX5L//ADRB1//DSTYK//NPTN//WWP2//YTHDF2//DLL1//KAT2B//FLRT1//RASGEF1A//SESN3//REST//FMR1//CPEB3//EGR2//NSMF//KCNJ10//RAB3GAP1//GRIA1//PXK//LRP8//CMKLR1//TMEM100//FAM13A//TAGAP//TRIP10//ARHGAP12//CD2AP//SLIT2//DRD1//GAB1//OTUD3//PLEKHA1//TSHZ3//GLRA1//SLC24A2//CALM2//JAK1//PPARG//TCF21//CTNND2//AMER2//CCNY//LRP6//EFNA5//RANBP9//JUN//DNAJC27//HAND2//ARRDC3//TMEM64//LATS2//FZD6//BCL2L11//MOAP1//BNIP3//CAPRIN2//TBL1XR1//FXR1//LYPD6//CREBRF//PAIP2//DYNLL2//E2F1//PPP3R1//PRKACB//MDM4//POU4F1//RBBP7//RBBP8//BAG5//WFS1//SERINC3//ASPH//DNAJB9//PPP1R15B//SESN1//PFN2//CRY2//PHF14//MYB//TMF1//NR4A2//ITGA6//URI1//ST18// |
| GO:0001704 | formation of primary germ layer | Biological process | 21 | 125 | 934 | 17653 | 3.17527194860814 | 2.36460047110904e-06 | 9.4069370506532e-05 | 5.62624222822448 | 0.0224839400428266 | LHX1//DUSP1//DUSP2//DUSP5//SOX17//NOG//NF2//BMPR2//NR4A3//ACVR1//HAND1//GATA6//KLF4//HSBP1//ITGA5//ITGAV//LAMA3//MMP2//HMGA2//FOXC1//FOXF1// |
| GO:0044249 | cellular biosynthetic process | Biological process | 415 | 6579 | 934 | 17653 | 1.19222947715348 | 2.40133782807876e-06 | 9.49722089549512e-05 | 5.61954673767323 | 0.444325481798715 | IGF1//NFIA//NFIB//RBBP7//RBBP8//RRM2//E2F1//NPAT//TSHZ1//HMG20A//KLF2//ZBTB18//CEBPB//ZNF268//TCERG1//WWP2//KLF12//OSR1//CRY2//GLIS3//ARX//DNMT3B//DR1//EDN1//EFNA1//CC2D1B//EN1//ESR1//FHL2//CPEB3//FOXF1//FOXC1//MYT1L//CIC//ZFPM2//DNAJB5//GATA6//CNOT7//HSBP1//AR//JARID2//JUN//ZNF568//MAF//MDM4//MYB//NR4A2//RLIM//KLF3//ZBTB7A//PIAS4//POU4F1//PPARG//CC2D1A//TSHZ3//ZBTB4//RARB//REST//RNF2//PRDM16//SOX17//SOX11//STAT3//ZEB1//TCF21//TFAP2A//TFAP2C//TGIF1//KLF10//TNF//UBE2I//VLDLR//WFS1//ZNF217//LRP8//BTG2//BHLHE41//TBL1XR1//NR4A3//HMGA2//ARID5B//BRMS1L//BHLHE40//RUNX3//URI1//PER2//SQSTM1//NOG//KLF4//HAND1//FNIP1//PHF14//USP3//GTF2E1//SECISBP2L//PTGES3//ST6GALNAC3//ST6GALNAC6//ST8SIA3//ST3GAL5//RDH10//RPL15//IRF9//KLF6//CREBRF//EGR2//ELK3//ERG//FOXJ3//FOXF2//FLI1//GRHL1//HIVEP2//IRF1//LHX1//MEF2D//MEOX2//MYBL1//NEUROD1//NHLH2//OTX2//PAX9//PBX3//POU3F2//RAD21//ARID4A//SHOX//BCL11B//BMPR2//SOX4//BTF3//TEF//TGFB1I1//THRA//TMF1//DEK//FOSL1//KLF7//CBFB//FUBP3//HAND2//CLOCK//POLR3G//SRSF2//PKD2//UGP2//NAGK//GFPT2//SLC35D1//NUP35//NUP43//HPRT1//ADCY3//IMPDH1//NME1//PDE10A//CMPK1//DCTPP1//REV3L//GMNC//CCNE2//ESCO2//PPP2CA//BAHD1//H2AFZ//KMT2B//DPY30//TOPORS//KHDRBS3//ZMYND11//TADA1//ZNF597//CTNND2//ZNF782//NKX2-3//ZNF800//E2F5//ZNF367//EZH1//ZBTB9//KDM2A//MYCBP2//FLII//CNOT6L//EPC2//LHX6//HBP1//ATAD2//SERTAD3//MDFIC//MNX1//HMGB3//HOXA3//HOXD1//FOXN2//ZBTB41//ZBTB34//ATXN3//LHX8//NEUROG1//NFE2//NPAS2//IER5//ARID4B//BRWD1//BNC2//ZNF532//CDCA7L//PRKAA1//TRERF1//LMO3//MAPK1//SLC2A4RG//PHTF2//ZNF630//ZNF287//CNOT6//ZNF512B//ZBTB26//MIER1//RBL2//RNF4//ATXN1//DMRTA1//SMARCD2//STAT4//ZNF2//ZNF3//ZNF131//ZNF655//BRD3//TCF7L1//PCGF5//LCOR//WASL//ZBTB47//ST18//HIPK3//ELAVL2//NR5A2//NRBF2//MLLT6//RNF141//PAK6//SNAPC1//BTG1//SNIP1//RPS6KA4//RPS6KA5//CDKN1A//CRK//EMX2//ETS1//BOLA3//NR3C2//PLEK//ZNFX1//NKX2-4//RFX7//BDP1//CDK8//E2F3//TEAD1//KAT2B//CCNC//CPEB2//CPEB1//CPEB4//MTPN//DDX6//MKNK2//ACO1//TSC1//KBTBD8//FUT9//SERP1//ST8SIA5//MAN1A1//MAN2A1//B3GNT5//B3GALT2//CCDC126//VEGFB//NTNG1//RECK//PIGA//GLUD2//LGSN//SERINC3//PSAT1//ELOVL4//SCD5//LCLAT1//DDHD1//CDS1//ABHD3//SLC44A1//PIKFYVE//PIK3CB//PTEN//PLEKHA1//PLEKHA3//MTMR9//PIK3R3//SPHK2//CHCHD10//G3BP2//NF2//RIMKLB//DGKE//BCAT2//MTRR//PPCS//EREG//CNEP1R1//LDLR//FBXW7//XYLT2//HS2ST1//HS3ST5//ACOT7//FAM129A//FMR1//PRR16//FXR1//ST3GAL3//MAP3K2//MAPRE3//BAMBI//RGMB//LRP6//MAP3K5//WNT7A//ACVR1//DKK3//NRG1//RASD1//TSG101//BTAF1//PPM1F//TOB1//NANOS1//CAPRIN2//OGN//CHST1//ZDHHC1//ST6GAL2//MCFD2//ZDHHC5//ELOVL7//PPM1L//SPTSSA//SGPP1//CHST7//DSEL//SOSTDC1//TMEM100//RGMA//BMP3//TGFB2//SULF1//CTDSPL2//ADRB1//PTHLH//CALM2//EGLN1//CMKLR1//DCP2//CCT6A//EPHA5//P2RY1//UXS1//ACSL1//GAB1//GRB2//NCK1//RNF128//ADIPOR2//ERRFI1//QKI//INTS8//NABP1//ITM2B//FZD6//NPLOC4//MID1IP1//MAP2K4//S1PR1//DLL1//IL2//ITGA6//MET//PPP1R12A//OSM//PPP3R1//DCAF6//PAIP2//PPT2//INHBB//YTHDF2//EGLN3//PFKP//ATG16L1//IDH1//ORMDL3//GSTA2//RAB3GAP1//RAP2C//TOB2//PPP1R15B//DUSP1//GJA1//NSMAF//NPNT//UNK// |
| GO:0008152 | metabolic process | Biological process | 685 | 11746 | 934 | 17653 | 1.10222998143065 | 2.44245059314923e-06 | 9.60365893108619e-05 | 5.61217421259985 | 0.733404710920771 | ACOX1//UBXN2B//TRAPPC8//RAB23//ATG16L1//ULK2//IGF1//NFIA//NFIB//RBBP7//RBBP8//RRM2//CDKN1A//GADD45A//PTEN//CCNE2//E2F1//NPAT//MTRR//TSHZ1//HMG20A//KLF2//ZBTB18//CEBPB//ZNF268//TCERG1//WWP2//KLF12//OSR1//CRY2//GLIS3//ARX//DNMT3B//DR1//EDN1//EFNA1//CC2D1B//EN1//ESR1//FHL2//CPEB3//FOXF1//FOXC1//MYT1L//CIC//ZFPM2//DNAJB5//GATA6//CNOT7//HSBP1//AR//JARID2//JUN//ZNF568//MAF//MDM4//MYB//NR4A2//RLIM//KLF3//ZBTB7A//PIAS4//POU4F1//PPARG//CC2D1A//TSHZ3//ZBTB4//RARB//REST//RNF2//PRDM16//SOX17//SOX11//STAT3//ZEB1//TCF21//TFAP2A//TFAP2C//TGIF1//KLF10//TNF//UBE2I//VLDLR//WFS1//ZNF217//LRP8//BTG2//BHLHE41//TBL1XR1//NR4A3//HMGA2//ARID5B//BRMS1L//BHLHE40//RUNX3//URI1//PER2//SQSTM1//NOG//KLF4//HAND1//FNIP1//PHF14//USP3//RANBP9//PPM1L//SPRED1//DUSP5//EREG//RASGEF1A//GRB2//NRG1//IL2//JAK1//MAP3K5//MET//MINK1//TAOK3//MAPK1//CCL2//SOS1//LAMTOR3//DCP2//PPP2CA//RPL15//MAP3K2//CRK//CRKL//ADAM9//LPAR1//LPAR3//NTF3//PIK3CB//PRKAA1//MAP2K4//DUSP10//DUSP1//DUSP2//RGS3//TOPORS//HECTD2//RNF145//ZNRF2//FBXL5//RNF180//MYLIP//ANKIB1//UBE2W//FBXW7//HECW2//SKP1//THOP1//LONRF3//FBXO30//CBFB//UBE3B//LONRF1//ZFAND4//CNOT6L//PAN3//CNOT6//COPS8//RBFOX3//FMR1//MBNL1//RBM25//SRSF2//ELAVL2//NOVA1//BNIP3//PTGES3//NABP1//GTF2E1//SECISBP2L//RDH10//NAPEPLD//TTR//CHST7//CHST1//ITGB8//ST6GALNAC3//ST6GALNAC6//ST8SIA3//ST3GAL5//ACSL1//CAMLG//EPS15//NSF//DRD1//FAM129A//LRP6//PPP1R15B//SLIT2//EFNA5//EPHA7//NPTN//KDR//P2RY1//AKTIP//CCND2//ADRB1//LYPLA1//F3//DUS2//IRF9//KLF6//CREBRF//EGR2//ELK3//ERG//FOXJ3//FOXF2//FLI1//GRHL1//HIVEP2//IRF1//LHX1//MEF2D//MEOX2//MYBL1//NEUROD1//NHLH2//OTX2//PAX9//PBX3//POU3F2//RAD21//ARID4A//SHOX//BCL11B//BMPR2//SOX4//BTF3//TEF//TGFB1I1//THRA//TMF1//DEK//FOSL1//KLF7//FUBP3//HAND2//CLOCK//POLR3G//PKD2//ERRFI1//TSG101//SOCS5//TRIM3//KLHL2//RNF38//MYCBP2//FBXO33//KLHL3//SPOPL//DCAF8//DCAF6//FEM1C//KLHL14//MARCH4//RNF4//MARCH7//UBE2Z//RNF128//KLHL15//KBTBD8//ARRDC4//FUT9//ST8SIA5//UEVLD//CASD1//SLC2A4//UGP2//PFKP//GFPT2//SERP1//MAN2A1//HS3ST5//XYLT2//HS2ST1//HS3ST3A1//HS3ST1//GNS//NAGK//SLC35D1//IDH1//ACO1//NUP35//NUP43//NLN//EGLN3//SESN3//VKORC1L1//KDM2A//STEAP2//SESN1//GLUD2//ZADH2//IMPDH1//LBR//EGLN1//RSBN1//MSMO1//CYBRD1//SCD5//GLYR1//IMMP2L//CHCHD10//COX8C//COX7A2L//COX7A2//GLRX//ASPH//GSTA2//DPYSL2//HPRT1//NME1//CMPK1//ADCY3//PDE10A//DCTPP1//REV3L//GMNC//ESCO2//EPC2//USP28//USP45//ATXN3//HMGB3//BAHD1//H2AFZ//KMT2B//DPY30//ARID4B//KHDRBS3//ZMYND11//TADA1//ZNF597//CTNND2//ZNF782//NKX2-3//ZNF800//E2F5//ZNF367//EZH1//ZBTB9//FLII//LHX6//HBP1//ATAD2//SERTAD3//MDFIC//MNX1//HOXA3//HOXD1//FOXN2//ZBTB41//ZBTB34//LHX8//NEUROG1//NFE2//NPAS2//IER5//BRWD1//BNC2//ZNF532//CDCA7L//TRERF1//LMO3//SLC2A4RG//PHTF2//ZNF630//ZNF287//ZNF512B//ZBTB26//MIER1//RBL2//ATXN1//DMRTA1//SMARCD2//STAT4//ZNF2//ZNF3//ZNF131//ZNF655//BRD3//TCF7L1//PCGF5//LCOR//WASL//ZBTB47//ST18//HIPK3//NR5A2//NRBF2//MLLT6//RNF141//PAK6//SNAPC1//BTG1//SNIP1//RPS6KA4//RPS6KA5//EMX2//ETS1//BOLA3//NR3C2//PLEK//ZNFX1//NKX2-4//RFX7//BDP1//CDK8//E2F3//TEAD1//KAT2B//CCNC//CMTR2//ZFC3H1//RTCA//PPP4R2//ESRP1//CPEB1//QKI//AGFG1//CPEB2//CPEB4//MTPN//DDX6//MKNK2//TSC1//RIMKLB//ADAM10//CDK6//FASTK//PDIK1L//CDK19//MORC3//MMD//DSTYK//LATS2//CDK17//PRKACB//FAM20C//CCL8//TGFB2//TGFBR2//ACVR1//STK17B//PPM1E//FLRT1//GNAQ//NCK1//NF2//ASPN//YWHAG//CAMK2N2//PPM1F//PTPN21//PPP1R12A//CTDSPL2//PPP3R1//PPP6C//PTPN4//NAA30//PCMTD1//MAN1A1//B3GNT5//B3GALT2//CCDC126//EDEM1//VEGFB//NTNG1//RECK//PTAR1//PIGA//MMP2//CTSA//NPEPL1//ADAM12//ADAM18//LMLN//ZFYVE9//USP24//USP27X//NPLOC4//USP46//BAG6//CDC23//SLC6A14//SLC6A6//LGSN//SLC25A32//BCAT2//SERINC3//PSAT1//CNEP1R1//LDLR//MID1IP1//ACOT7//UGT1A8//PRKAR2B//ELOVL4//PLAA//LCLAT1//DDHD1//CDS1//ABHD3//SLC44A1//PIKFYVE//PLEKHA1//PLEKHA3//MTMR9//PIK3R3//KDELC1//SGPP1//SPHK2//NSMAF//ORMDL3//ACBD3//UGT1A4//UGT1A1//SLC20A1//PMAIP1//UGT1A6//UGT1A9//RAB12//TBC1D17//TECPR2//DLC1//DLL1//G3BP2//PTGER4//OSBPL5//EPHA2//PTHLH//DGKE//ACBD5//PPCS//ST3GAL3//ST6GAL2//UGT1A7//TOB1//TOB2//RRAGD//NANOS1//RAB3GAP1//GJA1//MSN//ACTC1//ZNF804A//NDFIP2//NPNT//CALM2//GDF11//INHBB//BMP3//PPP1R37//PPP1R12C//PLEKHF2//SERPINI1//SPOCK1//PCOLCE2//PRR16//FXR1//INTS8//VPS37A//DYNLL2//CHMP3//SNX5//RHEB//GNPTG//DGKH//HDHD2//RBM47//MAPRE3//BAMBI//RGMB//WNT7A//DKK3//RASD1//BTAF1//DMWD//OTUD3//WDR20//JOSD1//CAPRIN2//CEP41//STK33//MASTL//EPHA4//EPHA5//PKDCC//OGN//TMX3//ZDHHC1//MCFD2//ZDHHC5//CRTAP//RGL1//TNFRSF21//ELOVL7//PON2//ADIPOR2//SLK//SPTSSA//SULF1//DSEL//UBXN4//DNAJB9//DERL2//JKAMP//SOSTDC1//TMEM100//RGMA//COL1A2//BAG5//GRIA1//RAP2C//CMKLR1//CCT6A//OSM//PFN2//IFNA1//UXS1//NSMF//PTPDC1//PTPRG//PTP4A1//CDC14A//WDFY3//GAB1//DNAJB12//KCTD20//ITGA6//BTBD10//UGT1A3//ITM2B//TDRD5//ARL6IP1//CD2AP//BCL2L11//ITGAV//FZD6//AHNAK//YTHDF2//FZD10//AIDA//TWF1//CKAP4//RAB38//MIA3//ANO8//CCDC71L//FAM135A//TMEFF2//IPO5//CCNY//S1PR1//PAIP2//MPP1//ENPP4//EFR3A//ZFYVE21//PPT2//ITGA5//SH3D19//ARRDC3//UGT1A10//UGT1A5//CHRM5//PMEPA1//LDLRAD4//DNAJC27//UBA5//REXO1//ZC3H12C//CALCOCO2//SNAP25//FYCO1//FEZ2//FRY//UNK//TBC1D12//ENPP5// |
| GO:0040007 | growth | Biological process | 84 | 954 | 934 | 17653 | 1.66418865231034 | 2.66617647506557e-06 | 0.000104227465322939 | 5.57411110788141 | 0.0899357601713062 | ESR1//EREG//AR//SOCS5//NANOS1//SPOCK1//TSG101//KIAA1109//URI1//S1PR1//BNC2//RARB//TGFBR2//IGF1//WNT7A//ADAM10//MTPN//IL2//DERL2//TGFB2//CDKN1A//FRZB//GJA1//SERTAD3//PPARG//PPP2CA//RBBP7//SOX17//BMPR2//CAPRIN2//BTG1//ADIPOR2//SLIT2//DPYSL2//RAB21//EPHA4//TNF//SQSTM1//KLF2//EN1//CLIC4//SLC12A5//PLEKHA1//SOS1//TBL1XR1//ARID5B//PKDCC//DLL1//LHX1//LRP12//OSM//ZMAT3//HMGA2//BRMS1L//STAT3//ADRB1//POU3F2//GOLGA4//NRG1//WFS1//PPM1F//BCL2L11//FOXC1//LATS2//PTEN//ARX//SERP1//EFNA5//EPHA7//ULK2//LPAR3//SEMA4B//JARID2//NOG//ZFPM2//GATA6//RDH10//MAP2K4//EDN1//LRP6//PLAA//DUSP10//FLRT1//ST6GAL2// |
| GO:0001933 | negative regulation of protein phosphorylation | Biological process | 47 | 435 | 934 | 17653 | 2.04211523788427 | 2.70626080761972e-06 | 0.000105186447367426 | 5.56763035191725 | 0.0503211991434689 | DUSP10//SPRED1//DUSP1//DUSP2//DUSP5//PPP2CA//RGS3//ERRFI1//TSG101//SOCS5//GADD45A//PPM1E//FLRT1//GNAQ//LRP6//NCK1//NF2//TAOK3//ASPN//DUS2//YWHAG//CAMK2N2//PPM1F//CALM2//CDK6//PTEN//JUN//EFNA1//HIPK3//AIDA//CDKN1A//LATS2//IPO5//KAT2B//ZMYND11//NTF3//PMEPA1//LDLRAD4//NOG//RANBP9//LMO3//KLF4//PPP1R15B//PRKAR2B//FAM129A//IL2//SLIT2// |
| GO:0048762 | mesenchymal cell differentiation | Biological process | 28 | 203 | 934 | 17653 | 2.60695562283098 | 3.217373806579e-06 | 0.000124272903830066 | 5.49249847820172 | 0.0299785867237687 | SEMA4B//ACVR1//FOXF2//PPP3R1//TGFB2//HMGA2//NOG//TMEM100//EFNA1//ERG//HAND2//FOXC1//BAMBI//TGFB1I1//TGFBR2//PPP2CA//PTEN//LDLRAD4//LRP6//KBTBD8//RDH10//EDN1//NRG1//SOX11//FRZB//MAPK1//OSR1//TCF21// |
| GO:0071840 | cellular component organization or biogenesis | Biological process | 409 | 6486 | 934 | 17653 | 1.19184014193641 | 3.23407231614544e-06 | 0.000124272903830066 | 5.49025027318912 | 0.437901498929336 | KIF23//UBXN2B//TRAPPC8//RAB23//ATG16L1//ULK2//WASL//TACC2//DYNC1LI2//ATXN3//NEFH//MAP7D1//MIEF1//BNIP3//SQSTM1//COPS8//PTGES3//CCNE2//FRY//KLF2//CDH20//NRG1//TGFB2//GTF2E1//NANOS1//SPOCK1//TSG101//KIAA1109//URI1//CLIP1//TBC1D20//AGFG1//TMF1//LATS2//SOX17//JUN//PMAIP1//BCL2L11//MOAP1//CLIC4//MET//FMR1//NTF3//GRHL1//PALLD//AR//BCL11B//FRMD6//NAP1L3//ANP32E//EPS8//RHOC//RND3//MARCKS//MYO1B//LIMA1//TWF1//CFL2//CORO2B//DLC1//FGD4//FGD5//FLII//NF2//PFN2//PAK6//TRIP10//WASF3//SH3D19//DPYSL2//MSN//BRWD1//CCL2//CCDC6//MAN2A1//STX6//SYT6//STX2//SNAP25//ZFAND6//HMG20A//ZMYND11//ATAD2//H2AFZ//JARID2//RSBN1//RBL2//BAG6//BRD3//HMGA2//TCF7L1//NFE2//SMARCD2//ESR1//HMGB3//MYB//DEK//KAT2B//BAHD1//KMT2B//DPY30//RPL15//TSC1//MTX3//IMMP2L//CLCN6//SLC12A5//ARHGAP12//CD2AP//NCK1//REEP3//NUP35//PIKFYVE//RAB38//CHCHD10//SERP1//PALM2//TMOD3//TMOD1//STMN2//RANBP9//SLAIN2//VBP1//MID1IP1//GOLGA8A//RAB30//TMED9//NPLOC4//BAG5//USP6NL//TMEM135//RAB22A//AKTIP//FAM160A2//GJC1//ITGA6//ITGA5//DYNLL2//NUP43//CENPQ//FBXW7//RAD21//CENPO//CNEP1R1//CDC23//PPP2CA//CDC14A//GADD45A//PPP1R12A//HAUS8//MYBL1//SYCP3//EREG//MASTL//EFNA5//ZFYVE9//SOS1//FOSL1//NTNG1//SLITRK3//OGN//POU4F1//DSCAML1//WNT7A//SLIT2//ARX//EFNA1//EPHA5//GAB1//GRB2//KIF5A//OTX2//PIK3CB//ENAH//MAPK1//NTN4//VLDLR//NR4A3//KLF7//RPS6KA5//FEZ2//DRD1//GPM6A//PTEN//EGR2//EPHA4//LHX1//NOG//CDC42EP3//CRK//LPAR1//BAMBI//KDR//MTPN//PLSCR4//ARHGAP6//PTPDC1//TMEM17//DZIP1//GMNC//C5ORF30//CEP41//SKP1//FCHO2//EPHA2//CNOT6L//PAN3//CNOT6//ETS1//CHMP3//ZNF804A//NPTN//NME1//SERPINI1//MYLIP//PTPRG//PMEPA1//LDLRAD4//EPS15//SNX5//SNX2//GJA1//GJB2//FLRT1//STRN//PEX5L//RPS6KA4//EPC2//CLOCK//ARID4B//MIER1//ARID4A//RBBP7//TBL1XR1//BRMS1L//PPM1F//ARID5B//USP3//THRA//NR4A2//NFIB//ADAM10//LAMA3//MMP2//E2F5//PLEK//S1PR1//ARHGEF4//HECW2//RAB21//RUFY2//COL1A2//HAPLN1//FOXF1//FOXF2//NPNT//ITGAV//ITGB8//SMOC2//TNF//TTR//ADAM12//MFAP5//RECK//FOXC1//ACTC1//IL2//DERL2//CDKN1A//FRZB//SERTAD3//PPARG//BMPR2//CAPRIN2//BTG1//ADIPOR2//FAM118B//TDRD5//CNN3//SLK//LRP12//PSD//BTG2//EPHA7//FNIP1//TBC1D12//EVI5//TBC1D17//PLEKHA1//MINK1//NEUROG1//LPAR3//DCP2//CCT6A//KIF13A//SULF1//FZD10//PTGER4//NEFM//DDX6//CNOT7//G3BP2//ADAM9//IQGAP2//ARFIP1//CADM2//RASSF8//PRKACB//RAB3GAP1//DNAJB12//PRDM16//PRKAA1//MIA3//NSF//RNF2//TOPORS//VPS37A//PCGF5//APOO//GLRA1//KCNA4//SLC6A1//XKR8//TADA1//DR1//VASH2//CEP97//TMEFF2//GOLGA4//EDN1//PRR16//DLL1//LDLR//MTBP//IGF1//MDM1//STAT3//CNIH1//SEC23A//GRIA1//SAR1B//PPP6C//MCFD2//UNK//KLF4//RGMA//MNX1//HPRT1//NSMF//FXR1//SEMA4B//POU3F2//CTNND2//UXS1//SBF2//KHDRBS3//DGKH//MCOLN2//AHNAK//PKD2//DCTPP1//GLRB//MZT1//PPM1E//PPFIA1//DNMT3B//NFIA//SYBU//CPEB3//C21ORF91//LRP8//CHRM2//NECAP1//EPN1//YWHAG//SGCE//MDM4//TEAD1//KDM2A//EZH1//BDP1//PER2//REST//RTN3//ARL6IP1//SACS//DDHD1//RNF4//DUSP1//SLC17A7//NEDD1//PRKAR2B//YTHDF2//ARRDC4//CRKL//DOCK5//ORMDL3//E2F1//PPP3R1//CALCOCO2//FYCO1//ASPH//RAP1B//PLAA//ELMOD1//SNIP1//LRP6//NSMAF//CBFB// |
| GO:0030030 | cell projection organization | Biological process | 118 | 1477 | 934 | 17653 | 1.50998682148403 | 3.27836226557042e-06 | 0.000124284417596731 | 5.48434305770717 | 0.126338329764454 | CD2AP//NCK1//NTNG1//SLITRK3//NEFH//OGN//POU4F1//DSCAML1//WNT7A//SLIT2//RANBP9//ARX//DPYSL2//EFNA1//EFNA5//EPHA5//GAB1//GRB2//KIF5A//OTX2//PIK3CB//ENAH//MAPK1//NTN4//SOS1//VLDLR//NR4A3//KLF7//RPS6KA5//FEZ2//EGR2//EPHA4//LHX1//NOG//PTPDC1//DYNLL2//TMEM17//DZIP1//RAB23//GMNC//CDC14A//C5ORF30//CEP41//EPHA2//TWF1//PTEN//SNAP25//ZNF804A//STMN2//NPTN//NME1//SERPINI1//LPAR1//MYLIP//PTPRG//SPOCK1//TSC1//BAG5//FLRT1//STRN//NR4A2//NFIB//WASF3//S1PR1//ARHGEF4//RAB21//JUN//FRY//GPM6A//LRP12//PSD//BTG2//CDC42EP3//EPHA7//LIMA1//PLEK//PLEKHA1//LPAR3//CEP97//GOLGA4//NRG1//FGD4//FGD5//ITGA6//ULK2//KLF4//RGMA//GJA1//MNX1//NTF3//MINK1//HPRT1//FMR1//NSMF//HECW2//FXR1//BMPR2//SEMA4B//POU3F2//CAPRIN2//WASL//SLC12A5//CTNND2//CPEB3//IL2//C21ORF91//LRP8//NEFM//BCL11B//SNX2//NEDD1//PRKAR2B//YWHAG//HAUS8//PFN2//EPS8//PLAA//E2F5// |
| GO:0048812 | neuron projection morphogenesis | Biological process | 61 | 628 | 934 | 17653 | 1.83586962451752 | 3.28221025645532e-06 | 0.000124284417596731 | 5.48383360174315 | 0.0653104925053533 | NTNG1//SLITRK3//NEFH//OGN//POU4F1//DSCAML1//WNT7A//SLIT2//RANBP9//ARX//DPYSL2//EFNA1//EFNA5//EPHA5//GAB1//GRB2//KIF5A//OTX2//PIK3CB//ENAH//MAPK1//NTN4//SOS1//VLDLR//NR4A3//KLF7//RPS6KA5//FEZ2//EGR2//EPHA4//LHX1//NOG//NR4A2//PTEN//NFIB//RAB21//EPHA7//GOLGA4//NRG1//ULK2//LPAR3//HPRT1//MINK1//FMR1//NSMF//HECW2//FXR1//BMPR2//SEMA4B//POU3F2//CAPRIN2//CTNND2//WASL//LRP8//BCL11B//PLAA//FLRT1//GJA1//GPM6A//MNX1//NTF3// |
| GO:0120039 | plasma membrane bounded cell projection morphogenesis | Biological process | 62 | 642 | 934 | 17653 | 1.82527500383571 | 3.2895032899327e-06 | 0.000124284417596731 | 5.4828696749154 | 0.0663811563169165 | NTNG1//SLITRK3//NEFH//OGN//POU4F1//DSCAML1//WNT7A//SLIT2//RANBP9//ARX//DPYSL2//EFNA1//EFNA5//EPHA5//GAB1//GRB2//KIF5A//OTX2//PIK3CB//ENAH//MAPK1//NTN4//SOS1//VLDLR//NR4A3//KLF7//RPS6KA5//FEZ2//EGR2//EPHA4//LHX1//NOG//NR4A2//PTEN//NFIB//RAB21//EPHA7//GOLGA4//NRG1//ULK2//LPAR3//GJA1//GPM6A//MNX1//NTF3//MINK1//HPRT1//FMR1//NSMF//HECW2//FXR1//BMPR2//SEMA4B//POU3F2//CAPRIN2//CTNND2//WASL//LRP8//BCL11B//SNX2//PLAA//FLRT1// |
| GO:0001934 | positive regulation of protein phosphorylation | Biological process | 86 | 990 | 934 | 17653 | 1.64185538468194 | 3.45516347603317e-06 | 0.000129818169935624 | 5.46153139979208 | 0.0920770877944325 | GADD45A//MINK1//TNF//MAP3K2//CRK//CRKL//MAP3K5//TAOK3//LAMTOR3//ADAM9//DUSP5//LPAR1//LPAR3//NRG1//IGF1//NTF3//PIK3CB//PRKAA1//MAPK1//MAP2K4//EREG//FBXW7//EFNA5//COPS8//MDFIC//CALM2//GDF11//INHBB//BMP3//BMPR2//TGFB2//ACVR1//PKD2//RAP2C//PAK6//TGFBR2//SLK//FMR1//RPS6KA4//RPS6KA5//OSM//PFN2//CAPRIN2//FNIP1//IFNA1//ADCY3//PRKACB//PRKAR2B//IL2//STAT3//EDN1//EFNA1//EPHA2//EPHA4//EPHA5//EPHA7//AR//ITGAV//KDR//MET//FZD10//CCNY//MAPRE3//CCNC//CCND2//CDKN1A//MMD//VLDLR//WNT7A//ITGA5//LRP8//NPNT//DSTYK//NPTN//JUN//P2RY1//DNAJC27//PTEN//CCL2//CCL8//HAND2//ACSL1//PPP2CA//FAM129A//AKTIP//SQSTM1// |
| GO:0048858 | cell projection morphogenesis | Biological process | 62 | 644 | 934 | 17653 | 1.81960644792318 | 3.63801749710858e-06 | 0.00013593321730909 | 5.43913521652063 | 0.0663811563169165 | NTNG1//SLITRK3//NEFH//OGN//POU4F1//DSCAML1//WNT7A//SLIT2//RANBP9//ARX//DPYSL2//EFNA1//EFNA5//EPHA5//GAB1//GRB2//KIF5A//OTX2//PIK3CB//ENAH//MAPK1//NTN4//SOS1//VLDLR//NR4A3//KLF7//RPS6KA5//FEZ2//EGR2//EPHA4//LHX1//NOG//NR4A2//PTEN//NFIB//RAB21//EPHA7//GOLGA4//NRG1//ULK2//LPAR3//GJA1//GPM6A//MNX1//NTF3//MINK1//HPRT1//FMR1//NSMF//HECW2//FXR1//BMPR2//SEMA4B//POU3F2//CAPRIN2//CTNND2//WASL//LRP8//BCL11B//SNX2//PLAA//FLRT1// |
| GO:0070848 | response to growth factor | Biological process | 66 | 701 | 934 | 17653 | 1.77949823897949 | 3.75011666135822e-06 | 0.000139351862531679 | 5.42595522171333 | 0.0706638115631692 | COL1A2//JUN//TGFB2//TGFBR2//KLF10//ADAM9//ACVR1//ZFYVE9//SPRED1//FLRT1//GRB2//ESRP1//MAPK1//DKK3//SOX11//ZEB1//TOB1//SOSTDC1//RGMB//TMEM100//RGMA//BMP3//BMPR2//NOG//NPNT//TGFB1I1//BAMBI//ASPN//PMEPA1//PRDM16//RASL11B//LDLRAD4//SULF1//GATA6//CTDSPL2//ITGA5//CRK//GAB1//KDR//ERRFI1//FOXC1//NTF3//OTX2//FAM20C//SHISA2//DSTYK//LHX1//CCL2//NPTN//SOS1//PAX9//TGIF1//LRP8//URI1//KLF4//RUNX3//EDN1//WNT7A//SLIT2//DLL1//STMN2//DYNC1LI2//E2F1//PTEN//EPHA2//P2RY1// |
| GO:0007423 | sensory organ development | Biological process | 53 | 521 | 934 | 17653 | 1.92269231875778 | 3.86086679392964e-06 | 0.000142683290313367 | 5.41331518197069 | 0.056745182012848 | FOXC1//SMOC1//TGFB2//STAT3//GJA1//TGFBR2//SOX11//TFAP2A//RARB//GPM6A//SLC17A7//TOPORS//FJX1//LHX1//RDH10//BCL11B//KLF4//LRIG1//NEUROD1//NEUROG1//SOBP//FZD6//MAPK1//TSHZ1//OSR1//EDN1//NOG//RAB3GAP1//BCL2L11//BNC2//PRDM16//HAND2//DLL1//GDF11//FOXF2//LRP6//ZEB1//NR4A3//GJB2//MAF//CXCL14//MDM1//MAN2A1//JUN//SOS1//BMPR2//CLIC4//NF2//TMOD1//WNT7A//TBC1D20//EPHA2//FRZB// |
| GO:0051239 | regulation of multicellular organismal process | Biological process | 209 | 2969 | 934 | 17653 | 1.33047810963107 | 4.01200841388392e-06 | 0.000147463113603788 | 5.39663816485912 | 0.223768736616702 | RPS6KA4//RPS6KA5//EREG//TMF1//TNF//ADRB1//CALM2//GLRA1//PBX3//TSHZ3//F3//MARCH7//TGFBR2//NME1//EDN1//GJA1//BMPR2//HAND2//SOX17//CHRM2//THRA//HS3ST5//NPNT//PLEK//SULF1//ETS1//KDR//WNT7A//SLIT2//MTPN//IGF1//TWF1//NR4A3//JARID2//DUSP10//PFN2//PTPRG//JUN//TGFB2//DOCK5//PPM1F//FOXC1//BAMBI//TGFB1I1//EFNA1//PPP2CA//PTEN//LDLRAD4//SNAP25//ZNF804A//STMN2//NPTN//NCK1//SERPINI1//LPAR1//EPHA4//MYLIP//SPOCK1//TSC1//BAG5//LDLR//NF2//KLF4//DLL1//SPRED1//PPARG//INHBB//PER2//LHX1//ENPP4//EGR2//LRP6//MAPK1//PTGER4//ACVR1//S1PR1//OSR1//FAM20C//TFAP2A//PKDCC//DPYSL2//RAB21//KIAA1109//RARB//TNFRSF21//WASF3//NRG1//ARRDC3//MAF//PTHLH//NPLOC4//POLR3G//IRF1//CD274//PDCD1LG2//ERRFI1//CMKLR1//KLF2//SOCS5//IL2//CEBPB//CD2AP//GATA6//SOS1//ZEB1//STAT3//POU3F2//OTX2//ZNF287//RNF128//PAX9//CLOCK//EPHA2//NR4A2//HMGB3//CBFB//TMEM100//BTG1//GRHL1//MYB//CDK6//NFE2//DPY30//KAT2B//KMT2B//NTF3//BCL11B//YWHAG//HMG20A//DDX6//REST//DNMT3B//MMD//NEUROD1//NEUROG1//SOX11//SMOC1//TOB1//TMEM64//NOG//TOB2//POU4F1//KLF10//EGLN1//VASH2//GAB1//ITGB8//VEGFB//ADAM12//HMGA2//GOLGA4//LAMA3//BCL2L11//LATS2//ARX//SERP1//PRKAA1//EPHA7//ULK2//EFNA5//LPAR3//RGMA//RHEB//FMR1//NSMF//HECW2//FXR1//SEMA4B//MAN2A1//CAPRIN2//ADAM9//SLITRK3//FLRT1//ZFPM2//VANGL1//FZD6//P2RY1//AR//NTN4//ESR1//BTBD7//CPEB3//C21ORF91//LRP8//FRZB//ASPN//MCOLN2//GJC1//DSC2//KCNJ3//MEOX2//VLDLR//C5ORF30//PRKACB//YTHDF2//NLN//TRPC3//EPN1//ITGA5//JAK1//ASPH//PLAA//SOSTDC1//FBXW7//ERG//NFIB//PHF14//FNIP1//MET// |
| GO:0051094 | positive regulation of developmental process | Biological process | 108 | 1329 | 934 | 17653 | 1.53592645047153 | 4.18575692593084e-06 | 0.000153017697784164 | 5.37822599548899 | 0.115631691648822 | TGFBR2//FOXC1//BAMBI//TGFB1I1//TGFB2//THRA//STMN2//NPTN//NCK1//NME1//SERPINI1//TWF1//ZNF804A//LHX1//OSR1//FAM20C//BMPR2//TFAP2A//ACVR1//PKDCC//WASF3//NRG1//ADAM9//POU3F2//OTX2//EDN1//DUSP10//IL2//ZNF268//AR//JUN//NEUROD1//NF2//SOX17//CEBPB//CMKLR1//CCDC71L//TMEM64//FRZB//PPARG//LMO3//TMEM100//BTG1//MYB//SOCS5//ETS1//STAT3//MAP3K5//DNMT3B//MMD//NEUROG1//PTEN//RARB//SOX11//ZEB1//NPNT//GJA1//IGF1//POU4F1//KLF10//TNF//F3//GAB1//GATA6//ITGB8//KDR//VEGFB//VASH2//ADAM12//HMGA2//GOLGA4//ARX//SERP1//DLL1//PRKAA1//EFNA5//LPAR3//RHEB//MAN2A1//SLIT2//EPHA4//RAB21//CAPRIN2//SLITRK3//FLRT1//WNT7A//ZFPM2//LPAR1//CPEB3//FMR1//C21ORF91//LRP8//MIEF1//BNIP3//DDHD1//NOG//PRDM16//VLDLR//CRK//CRKL//DOCK5//ITGA5//JAK1//KLF4//PLAA//LRP6//HAND2//NSMF// |
| GO:0043068 | positive regulation of programmed cell death | Biological process | 61 | 633 | 934 | 17653 | 1.82136828467141 | 4.22938463011199e-06 | 0.000153781334695954 | 5.373722817303 | 0.0653104925053533 | FNIP1//DLC1//EGLN3//F3//PMAIP1//PPARG//TNF//MAP3K5//PTEN//BNIP3//PIK3CB//MAP2K4//BCL2L11//ZNF268//FGD4//GADD45A//DUSP1//E2F1//LPAR1//CDK19//FRZB//LATS2//ITGA6//JUN//USP27X//NEUROD1//ARHGEF4//POU4F1//RARB//REST//MOAP1//ZMAT3//UBE2Z//SOS1//SOS2//SOX4//FOSL1//HMGA2//NSMAF//SQSTM1//SLIT2//PPM1F//EPHA7//MYB//TFAP2A//TGFB2//DYNLL2//PPP3R1//YWHAG//PIAS4//SERINC3//NCK1//FBXW7//CD274//E2F3//NR4A3//BTG1//STK17B//INHBB//ST18//CDKN1A// |
| GO:0001706 | endoderm formation | Biological process | 13 | 55 | 934 | 17653 | 4.46737395366946 | 4.27577494200117e-06 | 0.000154280214043731 | 5.36898516221426 | 0.0139186295503212 | GATA6//SOX17//HSBP1//ITGA5//ITGAV//LAMA3//MMP2//HMGA2//DUSP1//DUSP2//DUSP5//LHX1//NOG// |
| GO:0001944 | vasculature development | Biological process | 70 | 761 | 934 | 17653 | 1.73854136476573 | 4.2887298891352e-06 | 0.000154280214043731 | 5.36767130544013 | 0.0749464668094218 | S1PR1//EFNA1//EPHA2//ELK3//EREG//FOXC1//GAB1//CLIC4//HOXA3//ITGA5//ITGAV//JUN//KDR//MEOX2//MMP2//TMEM100//PTEN//CCL2//SOX17//TGFB2//VEGFB//WNT7A//HAND1//HAND2//COL1A2//CRKL//FOXF1//SPHK2//TGFBR2//EDN1//PPP3R1//ACVR1//GJC1//ZFPM2//QKI//BMPR2//RECK//SLIT2//SPRED1//SULF1//PPARG//KLF4//PKD2//SOX4//ETS1//EGLN1//VASH2//F3//GATA6//ITGB8//STAT3//BTG1//ADAM12//HMGA2//GJA1//SOS1//PIK3CB//ERRFI1//TCF21//FOSL1//MAPK1//DLL1//ADIPOR2//IMMP2L//NOG//OSR1//KLF2//EPN1//JAK1//FBXW7// |
| GO:0007264 | small GTPase mediated signal transduction | Biological process | 57 | 578 | 934 | 17653 | 1.86388306424724 | 4.36381042560645e-06 | 0.000155328683728297 | 5.36013412446805 | 0.0610278372591006 | CDKN1A//CRKL//GRB2//IGF1//JUN//USP28//SOS1//G3BP2//CDC42EP3//COL1A2//EPS8//RHOC//RND3//ARHGAP1//ARHGAP6//PAK6//ARAP2//APPL1//AGFG1//AGFG2//PSD//GIT2//RAB12//RAB21//RAB38//RAB30//RAB9B//DNAJC27//RAB23//RAB22A//RAB28//RALGPS2//RAP2C//RAP1B//CRK//FGD4//FGD5//ARHGEF4//ARHGEF28//SOS2//DLC1//MET//LPAR1//SQSTM1//RASGEF1A//NRG1//TGFB2//FAM13A//TAGAP//TRIP10//ARHGAP12//CD2AP//SLIT2//RAPGEF4//RGL1//DOCK9//DOCK5// |
| GO:0009792 | embryo development ending in birth or egg hatching | Biological process | 57 | 578 | 934 | 17653 | 1.86388306424724 | 4.36381042560645e-06 | 0.000155328683728297 | 5.36013412446805 | 0.0610278372591006 | BCL2L11//ADAM10//KLF2//RDH10//EDN1//FOXF1//FOXC1//ZFPM2//GATA6//GJA1//EPN1//AR//ZNF568//MAN2A1//MBNL1//TMEM100//BTF3//TGFBR2//FOSL1//ACVR1//NOG//HAND2//DLL1//MEOX2//HAND1//LATS2//SOX17//RBBP8//SOX4//SOX11//DLC1//LRP6//PRKACB//TGFB2//TSC1//FZD6//CEBPB//PKD2//KBTBD8//EN1//LHX1//OSR1//TFAP2A//HOXA3//DSCAML1//ZEB1//SLC35D1//SULF1//HOXD1//GRB2//EGLN1//MAPK1//VASH2//BMPR2//IGF1//RAI2//NR5A2// |
| GO:0040008 | regulation of growth | Biological process | 63 | 662 | 934 | 17653 | 1.79868123977047 | 4.42508869668064e-06 | 0.000156685208668331 | 5.35407801986041 | 0.0674518201284797 | NANOS1//SPOCK1//TSG101//KIAA1109//URI1//ADAM10//MTPN//IL2//DERL2//TGFB2//CDKN1A//FRZB//GJA1//SERTAD3//PPARG//PPP2CA//RBBP7//SOX17//BMPR2//CAPRIN2//BTG1//ADIPOR2//SLIT2//DPYSL2//RAB21//EPHA4//TNF//SQSTM1//IGF1//STAT3//ADRB1//POU3F2//TGFBR2//GOLGA4//NRG1//WFS1//PPM1F//BCL2L11//FOXC1//LATS2//PTEN//ARX//SERP1//DLL1//AR//EPHA7//ULK2//EFNA5//LPAR3//SEMA4B//JARID2//NOG//ZFPM2//GATA6//EDN1//PLAA//DUSP10//LRP12//OSM//ZMAT3//HMGA2//BRMS1L//SOCS5// |
| GO:0051960 | regulation of nervous system development | Biological process | 74 | 820 | 934 | 17653 | 1.70564840444978 | 4.5202227295941e-06 | 0.000159220137084609 | 5.3448401652217 | 0.0792291220556745 | PTEN//SNAP25//ZNF804A//STMN2//NPTN//NCK1//NME1//SERPINI1//TWF1//LPAR1//EPHA4//MYLIP//PTPRG//SPOCK1//TSC1//BAG5//NF2//LHX1//DPYSL2//RAB21//WNT7A//RARB//TNFRSF21//WASF3//NRG1//DLL1//NTF3//BCL11B//YWHAG//HMG20A//DDX6//REST//DNMT3B//MMD//NEUROD1//NEUROG1//SOX11//ZEB1//GOLGA4//EPHA7//ULK2//EFNA5//LPAR3//KLF4//RGMA//NOG//PPARG//RHEB//DUSP10//FMR1//NSMF//HECW2//FXR1//BMPR2//SEMA4B//POU4F1//PER2//MAN2A1//EFNA1//POU3F2//SLIT2//CAPRIN2//SLITRK3//FLRT1//MYB//CPEB3//IL2//C21ORF91//LRP8//LDLR//VLDLR//PRKACB//PLAA//STAT3// |
| GO:0010648 | negative regulation of cell communication | Biological process | 111 | 1380 | 934 | 17653 | 1.52025183874872 | 4.87058823899783e-06 | 0.000170672478032862 | 5.31241858426315 | 0.118843683083512 | DUSP10//SPRED1//DUSP1//DUSP2//DUSP5//PPP2CA//RGS3//SOX17//ERRFI1//TSG101//SOCS5//EPHA2//EPHA4//EPHA5//EPHA7//RGS17//KDR//MET//ACVR1//PIAS4//PMEPA1//LDLRAD4//PTEN//KLF4//PLEK//INHBB//APCDD1//FRZB//SOSTDC1//SHISA2//BAMBI//ASPN//PRDM16//RASL11B//TGFB1I1//TGFBR2//TOB1//CTDSPL2//NOG//PRKAA1//TSC1//FNIP1//SLC6A1//DLC1//NPLOC4//SULF1//EPS15//GRB2//EPN1//NF2//ZMYND11//ESR1//EFNA1//HIPK3//AIDA//LPAR1//WWP2//YTHDF2//FBXW7//TAOK3//FLRT1//NEUROD1//TGFB2//NCK1//REST//EDN1//OSM//CD2AP//SLIT2//ARHGAP12//OTUD3//PLEKHA1//GLRA1//DRD1//SLC24A2//GRIA1//PKD2//CALM2//PPARG//CNOT7//TCF21//RANBP9//LMO3//TMEM64//AMER2//LATS2//DKK3//LRP6//FZD6//IGF1//CREBRF//FMR1//EREG//NRG1//PRKACB//BAG5//WFS1//DNAJB9//PPP1R15B//SESN3//SESN1//LYPD6//CRY2//CLOCK//PHF14//NR4A2//ITGA6//AR//ITGAV//TNF//URI1// |
| GO:0048667 | cell morphogenesis involved in neuron differentiation | Biological process | 56 | 567 | 934 | 17653 | 1.86670896449626 | 5.05991898404462e-06 | 0.000176392948912854 | 5.29585643673017 | 0.0599571734475375 | NTNG1//SLITRK3//NEFH//OGN//POU4F1//DSCAML1//WNT7A//SLIT2//RANBP9//ARX//DPYSL2//EFNA1//EFNA5//EPHA5//GAB1//GRB2//KIF5A//OTX2//PIK3CB//ENAH//MAPK1//NTN4//SOS1//VLDLR//NR4A3//KLF7//RPS6KA5//FEZ2//EGR2//EPHA4//LHX1//NOG//NR4A2//PTEN//NFIB//RAB21//EPHA7//GOLGA4//NRG1//ULK2//LPAR3//HPRT1//MINK1//FMR1//NSMF//HECW2//FXR1//BMPR2//SEMA4B//POU3F2//CAPRIN2//CTNND2//WASL//LRP8//BCL11B//UNK// |
| GO:0023057 | negative regulation of signaling | Biological process | 111 | 1384 | 934 | 17653 | 1.51585804730725 | 5.55796863536378e-06 | 0.000191088902848482 | 5.25508390835513 | 0.118843683083512 | DUSP10//SPRED1//DUSP1//DUSP2//DUSP5//PPP2CA//RGS3//SOX17//ERRFI1//TSG101//SOCS5//EPHA2//EPHA4//EPHA5//EPHA7//RGS17//KDR//MET//ACVR1//PIAS4//PMEPA1//LDLRAD4//PTEN//KLF4//PLEK//INHBB//APCDD1//FRZB//SOSTDC1//SHISA2//BAMBI//ASPN//PRDM16//RASL11B//TGFB1I1//TGFBR2//TOB1//CTDSPL2//NOG//PRKAA1//TSC1//FNIP1//SLC6A1//DLC1//NPLOC4//SULF1//EPS15//GRB2//EPN1//NF2//ZMYND11//ESR1//EFNA1//HIPK3//AIDA//LPAR1//WWP2//YTHDF2//FBXW7//TAOK3//FLRT1//NEUROD1//TGFB2//NCK1//REST//EDN1//OSM//CD2AP//SLIT2//ARHGAP12//OTUD3//PLEKHA1//GLRA1//DRD1//SLC24A2//GRIA1//PKD2//CALM2//PPARG//CNOT7//TCF21//RANBP9//LMO3//TMEM64//AMER2//LATS2//DKK3//LRP6//FZD6//IGF1//CREBRF//FMR1//EREG//NRG1//PRKACB//BAG5//WFS1//DNAJB9//PPP1R15B//SESN3//SESN1//LYPD6//CRY2//CLOCK//PHF14//NR4A2//ITGA6//AR//ITGAV//TNF//URI1// |
| GO:0007369 | gastrulation | Biological process | 26 | 186 | 934 | 17653 | 2.6419953489443 | 5.59108738556159e-06 | 0.000191088902848482 | 5.25250371990899 | 0.0278372591006424 | LHX1//LRP6//RNF2//ACVR1//DUSP1//DUSP2//DUSP5//SOX17//NOG//NF2//BMPR2//NR4A3//HAND1//GATA6//KLF4//HSBP1//ITGA5//ITGAV//LAMA3//MMP2//HMGA2//FOXC1//FOXF1//OTX2//OSR1//TGFBR2// |
| GO:0044092 | negative regulation of molecular function | Biological process | 95 | 1138 | 934 | 17653 | 1.57780376557543 | 5.59798131060212e-06 | 0.000191088902848482 | 5.25196855580125 | 0.101713062098501 | DUSP10//SPRED1//DUSP1//DUSP2//DUSP5//PPP2CA//RGS3//PKD2//THRA//ERRFI1//TSG101//SOCS5//GADD45A//PPM1E//FLRT1//GNAQ//LRP6//NCK1//NF2//TAOK3//ASPN//DUS2//YWHAG//CAMK2N2//PPM1F//G3BP2//TGFB2//TNF//PPP1R37//PPP1R12A//PPP1R12C//PLEKHF2//URI1//SERPINI1//SPOCK1//RECK//IQGAP2//FZD10//CPEB2//LRCH1//IPO5//TSC1//SLIT2//CDK6//PTEN//CMKLR1//PIAS4//KLF4//WWP2//NDFIP2//CRY2//MASTL//CNN3//PXK//BTAF1//FRY//UGT1A8//UGT1A7//UGT1A1//ANP32E//ARL6IP1//FNIP1//E2F1//JUN//HMGA2//HAND2//ESR1//RLIM//EGLN1//RNF2//WFS1//FZD6//BHLHE40//HAND1//HIPK3//AIDA//CDKN1A//LATS2//KAT2B//P2RY1//BAG5//PPARG//NOG//CALM2//GNB5//FMR1//MTRR//MET//OSR1//LYPD6//PRKAR2B//HECW2//SOX11//PER2//CHCHD10// |
| GO:0010629 | negative regulation of gene expression | Biological process | 150 | 2005 | 934 | 17653 | 1.41399712709661 | 5.61650874820284e-06 | 0.000191088902848482 | 5.25053356032802 | 0.160599571734475 | TSHZ1//HMG20A//KLF2//ZBTB18//CEBPB//ZNF268//TCERG1//WWP2//KLF12//OSR1//CRY2//GLIS3//ARX//DNMT3B//DR1//E2F1//EDN1//EFNA1//CC2D1B//EN1//ESR1//FHL2//CPEB3//FOXF1//FOXC1//MYT1L//CIC//ZFPM2//DNAJB5//GATA6//CNOT7//HSBP1//AR//JARID2//JUN//ZNF568//MAF//MDM4//MYB//NFIA//NFIB//NR4A2//RLIM//KLF3//ZBTB7A//PIAS4//POU4F1//PPARG//CC2D1A//TSHZ3//ZBTB4//RARB//RBBP7//RBBP8//REST//RNF2//PRDM16//SOX17//SOX11//STAT3//ZEB1//TCF21//TFAP2A//TFAP2C//TGIF1//KLF10//TNF//UBE2I//VLDLR//WFS1//ZNF217//LRP8//BTG2//BHLHE41//TBL1XR1//NR4A3//HMGA2//ARID5B//BRMS1L//BHLHE40//RUNX3//URI1//PER2//SQSTM1//NOG//KLF4//HAND1//FNIP1//PHF14//USP3//DCP2//PPP2CA//RPL15//CNOT6L//PAN3//CNOT6//BAHD1//H2AFZ//DPY30//SNIP1//KMT2B//ELK3//EREG//FOXF2//DKK3//MDFIC//NRG1//IRF1//LHX1//PAX9//RASD1//ARID4A//ATXN1//THRA//TSG101//BTAF1//RPS6KA5//CLOCK//PPM1F//TOB1//FMR1//NANOS1//CAPRIN2//TSC1//FXR1//ATAD2//MIER1//YTHDF2//DDX6//PAIP2//NUP35//NUP43//QKI//CPEB2//NCK1//CPEB1//CPEB4//UNK//CDKN1A//GJA1//ITGB8//KDR//LDLR//NME1//POU3F2//NDFIP2//RBL2//TGFB2//TMF1//HAND2// |
| GO:0071396 | cellular response to lipid | Biological process | 56 | 569 | 934 | 17653 | 1.86014759730998 | 5.62275494112788e-06 | 0.000191088902848482 | 5.25005084411555 | 0.0599571734475375 | ESR1//FHL2//AR//PMEPA1//RNF4//TGFB1I1//MAPK1//CCL2//TNF//UBA5//CBFB//PGRMC2//NR5A2//NR3C2//NR4A2//PPARG//RARB//THRA//NR4A3//TCF21//CEBPB//CDK19//CD274//PLSCR4//PDCD1LG2//ADAM9//PLAA//FZD10//OSR1//MYB//KLF4//PRKAA1//PTGER4//URI1//BCL2L11//EDN1//UGT1A1//REST//H2AFZ//POU4F1//MSN//LRP6//LRP8//E2F1//LDLR//NME1//DNMT3B//GJB2//ERRFI1//CREBRF//ADCY3//EFNA5//LPAR1//CRY2//CLOCK//LMO3// |
| GO:0006915 | apoptotic process | Biological process | 147 | 1958 | 934 | 17653 | 1.41898005874981 | 5.87202503178964e-06 | 0.000198562526449967 | 5.23121210173896 | 0.157387580299786 | BCL2L11//TNFRSF21//JUN//PMAIP1//MOAP1//PTEN//IL2//FNIP1//HAND2//DLC1//EGLN3//F3//PPARG//TNF//HIPK3//TOPORS//CDKN1A//ZNF268//OSR1//DUSP1//EPHA2//EPHA4//EPHA5//EPHA7//FHL2//ARL6IP1//DSTYK//GATA6//IGF1//KDR//MDM4//MET//ZFAND6//PRKAA1//SPHK2//RARB//BCL11B//BNIP3//STAT3//ACTC1//TFAP2A//TMF1//WNT7A//BAG6//HMGA2//SQSTM1//CCND2//E2F1//MAP3K5//GDF11//MAP3K2//ESR1//RASSF3//INHBB//NF2//NME1//NTF3//MINK1//TAOK3//PAK6//RBM25//MAP2K4//BMP3//TGFB2//ANP32E//SLK//PIK3CB//THRA//DLL1//CCL2//EDN1//LRP6//USP28//FGD4//GADD45A//LPAR1//CDK19//FRZB//LATS2//ITGA6//USP27X//NEUROD1//ARHGEF4//POU4F1//REST//ZMAT3//UBE2Z//SOS1//SOS2//SOX4//FOSL1//NSMAF//SLIT2//KLF4//PPM1F//NSMF//CEBPB//EN1//NR4A2//WFS1//BTG2//NR4A3//CPEB4//MYB//EFNA1//XKR8//BMPR2//FASTK//SGPP1//APPL1//MKNK2//ITGAV//ITM2B//DYNLL2//PPP3R1//YWHAG//PIAS4//BAG5//SERINC3//NCK1//FOXC1//FBXW7//CD274//E2F3//TGFBR2//IER3IP1//BTG1//STK17B//ITGA5//NOG//ZMYND11//AR//ACVR1//URI1//ST18//RTN3//SULF1//GJA1//IRF1//C6ORF120//MEF2D//CHMP3//PPP2CA//MAPK1//RAD21//AKTIP//FXR1// |
| GO:0072358 | cardiovascular system development | Biological process | 70 | 769 | 934 | 17653 | 1.72045510869535 | 6.14493006606382e-06 | 0.000206757025058655 | 5.21148305534798 | 0.0749464668094218 | S1PR1//EFNA1//EPHA2//ELK3//EREG//FOXC1//GAB1//CLIC4//HOXA3//ITGA5//ITGAV//JUN//KDR//MEOX2//MMP2//TMEM100//PTEN//CCL2//SOX17//TGFB2//VEGFB//WNT7A//HAND1//HAND2//COL1A2//CRKL//FOXF1//SPHK2//TGFBR2//EDN1//PPP3R1//ACVR1//GJC1//ZFPM2//QKI//FBXW7//TCF21//BMPR2//RECK//SLIT2//SPRED1//SULF1//PPARG//KLF4//PKD2//SOX4//ETS1//EGLN1//VASH2//F3//GATA6//ITGB8//STAT3//BTG1//ADAM12//HMGA2//GJA1//SOS1//PIK3CB//ERRFI1//FOSL1//MAPK1//DLL1//ADIPOR2//IMMP2L//NOG//OSR1//KLF2//EPN1//JAK1// |
| GO:1901698 | response to nitrogen compound | Biological process | 90 | 1065 | 934 | 17653 | 1.59721929004433 | 6.19103164968072e-06 | 0.000207276965578172 | 5.20823697578898 | 0.0963597430406852 | DRD1//HPRT1//NR4A2//CHRM2//CHRM5//GNAQ//GAB1//APPL1//GRB2//SOS1//PIK3R3//SNIP1//CDKN1A//SLC6A1//NME1//CEBPB//COL1A2//SESN3//CPEB3//NSMF//SESN1//GLRA1//IPO5//MMP2//RRAGD//CPEB1//ZEB1//TNF//CPEB4//LAMTOR3//UBXN4//DNAJB9//DERL2//JKAMP//NPLOC4//WFS1//BAG6//EDEM1//DNMT3B//PPARG//PRKAA1//EGR2//IGF1//TSC1//CPEB2//INHBB//ERRFI1//PTEN//SLC2A4//YWHAG//KAT2B//TRPC3//DNAJB12//EDN1//GLRB//IFNA1//MAPK1//PMAIP1//EREG//GJA1//LRP6//BTG1//BTG2//NCK1//DUSP1//JUN//FOSL1//STAT3//CRK//PKD2//RAP1B//HCN2//NR4A3//ADCY3//GJB2//PRKACB//PRKAR2B//LDLR//SOCS5//KLF2//KLF4//SLIT2//EGLN1//FMR1//KLF3//KLF10//MAP3K5//ESR1//ATXN3//EPHA4// |
| GO:0043065 | positive regulation of apoptotic process | Biological process | 60 | 627 | 934 | 17653 | 1.80865342253824 | 6.31084275219475e-06 | 0.000210247436123611 | 5.19991264104293 | 0.0642398286937901 | FNIP1//DLC1//EGLN3//F3//PMAIP1//PPARG//TNF//MAP3K5//PTEN//BNIP3//PIK3CB//MAP2K4//BCL2L11//REST//PPM1F//EPHA7//JUN//MYB//TFAP2A//TGFB2//MOAP1//DYNLL2//E2F1//PPP3R1//YWHAG//PIAS4//SERINC3//NCK1//FBXW7//CD274//E2F3//NR4A3//BTG1//STK17B//INHBB//ST18//ZNF268//FGD4//GADD45A//DUSP1//LPAR1//CDK19//FRZB//LATS2//ITGA6//USP27X//NEUROD1//ARHGEF4//POU4F1//RARB//ZMAT3//UBE2Z//SOS1//SOS2//SOX4//FOSL1//HMGA2//NSMAF//SQSTM1//SLIT2// |
| GO:0045936 | negative regulation of phosphate metabolic process | Biological process | 59 | 614 | 934 | 17653 | 1.81616493105204 | 6.6417323084866e-06 | 0.000220186449030857 | 5.17771863239465 | 0.063169164882227 | DUSP10//SPRED1//DUSP1//DUSP2//DUSP5//PPP2CA//RGS3//FAM129A//IL2//LRP6//PTEN//PPP1R15B//SLIT2//ERRFI1//TSG101//SOCS5//GADD45A//PPM1E//FLRT1//GNAQ//NCK1//NF2//TAOK3//ASPN//DUS2//YWHAG//CAMK2N2//PPM1F//TGFB2//TNF//CALM2//PLEK//PPP1R37//PPP1R12A//PPP1R12C//PLEKHF2//URI1//EGLN1//CDK6//JUN//CRY2//MASTL//CDKN1A//EFNA1//HIPK3//AIDA//LATS2//IPO5//KAT2B//STAT3//ZMYND11//NTF3//PMEPA1//LDLRAD4//NOG//RANBP9//LMO3//KLF4//PRKAR2B// |
| GO:0071704 | organic substance metabolic process | Biological process | 657 | 11249 | 934 | 17653 | 1.10388313365185 | 6.90713167216103e-06 | 0.000227867958530854 | 5.16070226486429 | 0.703426124197002 | ACOX1//IGF1//NFIA//NFIB//RBBP7//RBBP8//RRM2//CDKN1A//GADD45A//PTEN//CCNE2//E2F1//NPAT//MTRR//TSHZ1//HMG20A//KLF2//ZBTB18//CEBPB//ZNF268//TCERG1//WWP2//KLF12//OSR1//CRY2//GLIS3//ARX//DNMT3B//DR1//EDN1//EFNA1//CC2D1B//EN1//ESR1//FHL2//CPEB3//FOXF1//FOXC1//MYT1L//CIC//ZFPM2//DNAJB5//GATA6//CNOT7//HSBP1//AR//JARID2//JUN//ZNF568//MAF//MDM4//MYB//NR4A2//RLIM//KLF3//ZBTB7A//PIAS4//POU4F1//PPARG//CC2D1A//TSHZ3//ZBTB4//RARB//REST//RNF2//PRDM16//SOX17//SOX11//STAT3//ZEB1//TCF21//TFAP2A//TFAP2C//TGIF1//KLF10//TNF//UBE2I//VLDLR//WFS1//ZNF217//LRP8//BTG2//BHLHE41//TBL1XR1//NR4A3//HMGA2//ARID5B//BRMS1L//BHLHE40//RUNX3//URI1//PER2//SQSTM1//NOG//KLF4//HAND1//FNIP1//PHF14//USP3//RANBP9//PPM1L//SPRED1//DUSP5//EREG//RASGEF1A//GRB2//NRG1//IL2//JAK1//MAP3K5//MET//MINK1//TAOK3//MAPK1//CCL2//SOS1//LAMTOR3//DCP2//PPP2CA//RPL15//MAP3K2//CRK//CRKL//ADAM9//LPAR1//LPAR3//NTF3//PIK3CB//PRKAA1//MAP2K4//DUSP10//DUSP1//DUSP2//RGS3//TOPORS//HECTD2//RNF145//ZNRF2//FBXL5//RNF180//MYLIP//ANKIB1//UBE2W//FBXW7//HECW2//SKP1//THOP1//LONRF3//FBXO30//CBFB//UBE3B//LONRF1//ZFAND4//CNOT6L//PAN3//CNOT6//COPS8//RBFOX3//FMR1//MBNL1//RBM25//SRSF2//ELAVL2//NOVA1//PTGES3//NABP1//GTF2E1//SECISBP2L//RDH10//NAPEPLD//TTR//ITGB8//ST6GALNAC3//ST6GALNAC6//ST8SIA3//ST3GAL5//ACSL1//CAMLG//EPS15//NSF//DRD1//FAM129A//LRP6//PPP1R15B//SLIT2//EFNA5//EPHA7//NPTN//KDR//P2RY1//AKTIP//CCND2//LYPLA1//F3//DUS2//IRF9//KLF6//CREBRF//EGR2//ELK3//ERG//FOXJ3//FOXF2//FLI1//GRHL1//HIVEP2//IRF1//LHX1//MEF2D//MEOX2//MYBL1//NEUROD1//NHLH2//OTX2//PAX9//PBX3//POU3F2//RAD21//ARID4A//SHOX//BCL11B//BMPR2//SOX4//BTF3//TEF//TGFB1I1//THRA//TMF1//DEK//FOSL1//KLF7//FUBP3//HAND2//CLOCK//POLR3G//PKD2//ERRFI1//TSG101//SOCS5//TRIM3//KLHL2//RNF38//MYCBP2//FBXO33//KLHL3//SPOPL//DCAF8//DCAF6//FEM1C//KLHL14//MARCH4//RNF4//MARCH7//UBE2Z//RNF128//KLHL15//KBTBD8//ARRDC4//FUT9//ST8SIA5//UEVLD//CASD1//SLC2A4//CHST7//CHST1//UGP2//PFKP//GFPT2//SERP1//MAN2A1//HS3ST5//XYLT2//HS2ST1//HS3ST3A1//HS3ST1//GNS//NAGK//SLC35D1//IDH1//ACO1//NUP35//NUP43//NLN//CHCHD10//COX8C//COX7A2L//GSTA2//DPYSL2//HPRT1//NME1//CMPK1//ADCY3//IMPDH1//PDE10A//DCTPP1//REV3L//GMNC//ESCO2//EPC2//USP28//USP45//ATXN3//KDM2A//HMGB3//BAHD1//H2AFZ//KMT2B//DPY30//ARID4B//KHDRBS3//ZMYND11//TADA1//ZNF597//CTNND2//ZNF782//NKX2-3//ZNF800//E2F5//ZNF367//EZH1//ZBTB9//FLII//LHX6//HBP1//ATAD2//SERTAD3//MDFIC//MNX1//HOXA3//HOXD1//FOXN2//ZBTB41//ZBTB34//LHX8//NEUROG1//NFE2//NPAS2//IER5//BRWD1//BNC2//ZNF532//CDCA7L//TRERF1//LMO3//SLC2A4RG//PHTF2//ZNF630//ZNF287//ZNF512B//ZBTB26//MIER1//RBL2//ATXN1//DMRTA1//SMARCD2//STAT4//ZNF2//ZNF3//ZNF131//ZNF655//BRD3//TCF7L1//PCGF5//LCOR//WASL//ZBTB47//ST18//HIPK3//NR5A2//NRBF2//MLLT6//RNF141//PAK6//SNAPC1//BTG1//SNIP1//RPS6KA4//RPS6KA5//EMX2//ETS1//BOLA3//NR3C2//PLEK//ZNFX1//NKX2-4//RFX7//BDP1//CDK8//E2F3//TEAD1//KAT2B//CCNC//CMTR2//ZFC3H1//RTCA//PPP4R2//ESRP1//CPEB1//QKI//AGFG1//CPEB2//CPEB4//MTPN//DDX6//MKNK2//TSC1//RIMKLB//IMMP2L//ADAM10//CDK6//FASTK//PDIK1L//CDK19//MORC3//MMD//DSTYK//LATS2//CDK17//PRKACB//FAM20C//CCL8//TGFB2//TGFBR2//ACVR1//STK17B//PPM1E//FLRT1//GNAQ//NCK1//NF2//ASPN//YWHAG//CAMK2N2//PPM1F//PTPN21//PPP1R12A//CTDSPL2//PPP3R1//PPP6C//PTPN4//NAA30//PCMTD1//ASPH//MAN1A1//B3GNT5//B3GALT2//CCDC126//EDEM1//VEGFB//NTNG1//RECK//PTAR1//PIGA//MMP2//CTSA//NPEPL1//ADAM12//ADAM18//LMLN//ZFYVE9//USP24//USP27X//NPLOC4//USP46//BAG6//CDC23//SLC6A14//SLC6A6//GLUD2//LGSN//SLC25A32//BCAT2//SERINC3//PSAT1//CNEP1R1//LDLR//MID1IP1//MSMO1//ACOT7//UGT1A8//PRKAR2B//ELOVL4//SCD5//PLAA//LCLAT1//DDHD1//CDS1//ABHD3//SLC44A1//PIKFYVE//PLEKHA1//PLEKHA3//MTMR9//PIK3R3//KDELC1//SGPP1//SPHK2//NSMAF//ORMDL3//ZADH2//ACBD3//LBR//UGT1A4//UGT1A1//DLC1//EGLN3//PMAIP1//DLL1//G3BP2//PTGER4//OSBPL5//EPHA2//PTHLH//DGKE//ACBD5//ST3GAL3//ST6GAL2//UGT1A7//TOB1//TOB2//NANOS1//RAB3GAP1//GJA1//MSN//ACTC1//ZNF804A//NDFIP2//CALM2//GDF11//INHBB//BMP3//SERPINI1//SPOCK1//PCOLCE2//PPCS//GLRX//PRR16//FXR1//INTS8//GNPTG//RBM47//MAPRE3//BAMBI//RGMB//WNT7A//DKK3//RASD1//BTAF1//DMWD//OTUD3//WDR20//JOSD1//CAPRIN2//VKORC1L1//CEP41//STK33//MASTL//EPHA4//EPHA5//PKDCC//OGN//TMX3//ZDHHC1//MCFD2//ZDHHC5//CRTAP//EGLN1//RGL1//TNFRSF21//UGT1A9//ELOVL7//PON2//ADIPOR2//SLK//SPTSSA//SULF1//DSEL//UBXN4//DNAJB9//DERL2//JKAMP//SOSTDC1//TMEM100//RGMA//ADRB1//BAG5//GRIA1//RAP2C//CMKLR1//CCT6A//PPP1R37//OSM//PFN2//IFNA1//UXS1//NSMF//PTPDC1//PTPRG//PTP4A1//CDC14A//BNIP3//GAB1//DNAJB12//UGT1A3//ITM2B//TDRD5//ARL6IP1//UBXN2B//CD2AP//VPS37A//BCL2L11//ITGAV//FZD6//AHNAK//YTHDF2//FZD10//AIDA//TWF1//CKAP4//RAB38//MIA3//ANO8//CCDC71L//FAM135A//RAB12//TMEFF2//IPO5//CCNY//S1PR1//ITGA6//PAIP2//MPP1//RAB23//ENPP4//DGKH//ULK2//EFR3A//ZFYVE21//PPT2//ITGA5//SH3D19//ARRDC3//UGT1A10//UGT1A6//UGT1A5//CHRM5//PMEPA1//LDLRAD4//ATG16L1//NPNT//DNAJC27//UBA5//REXO1//ZC3H12C//SNAP25//FRY//UNK// |
| GO:0010563 | negative regulation of phosphorus metabolic process | Biological process | 59 | 615 | 934 | 17653 | 1.81321181734301 | 6.98146766639815e-06 | 0.000228652597288548 | 5.1560532690079 | 0.063169164882227 | DUSP10//SPRED1//DUSP1//DUSP2//DUSP5//PPP2CA//RGS3//FAM129A//IL2//LRP6//PTEN//PPP1R15B//SLIT2//ERRFI1//TSG101//SOCS5//GADD45A//PPM1E//FLRT1//GNAQ//NCK1//NF2//TAOK3//ASPN//DUS2//YWHAG//CAMK2N2//PPM1F//TGFB2//TNF//CALM2//PLEK//PPP1R37//PPP1R12A//PPP1R12C//PLEKHF2//URI1//EGLN1//CDK6//JUN//CRY2//MASTL//CDKN1A//EFNA1//HIPK3//AIDA//LATS2//IPO5//KAT2B//STAT3//ZMYND11//NTF3//PMEPA1//LDLRAD4//NOG//RANBP9//LMO3//KLF4//PRKAR2B// |
| GO:0007420 | brain development | Biological process | 65 | 700 | 934 | 17653 | 1.755039767513 | 6.99853432481582e-06 | 0.000228652597288548 | 5.15499290302768 | 0.069593147751606 | NOG//POU4F1//LHX1//LRP8//CDK6//DRD1//EMX2//NEUROD1//PTEN//BTG2//LPAR1//EN1//FOXC1//DLC1//LRP6//EGR2//DLL1//MTPN//WNT7A//NFIB//HPRT1//RARB//ARX//CALM2//SLC32A1//EPHA5//EZH1//NF2//NME1//TSC1//NR4A3//BCL11B//LHX6//POU3F2//SLIT2//NRG1//ETS1//RAB3GAP1//GNAQ//LHX8//C21ORF91//NR4A2//TACC2//BNIP3//E2F1//OTX2//FZD6//NANOS1//SOS1//BCL2L11//DPYSL2//S1PR1//EPHA7//CIC//SPHK2//SLC17A7//DSCAML1//PTPRG//ATXN1//BMPR2//TGFBR2//THRA//BAG6//IMMP2L//B3GNT5// |
| GO:0043009 | chordate embryonic development | Biological process | 55 | 560 | 934 | 17653 | 1.8562920617926 | 7.24204424498514e-06 | 0.000235127486121209 | 5.14013882620581 | 0.0588865096359743 | BCL2L11//ADAM10//KLF2//RDH10//EDN1//FOXF1//FOXC1//ZFPM2//GATA6//GJA1//EPN1//AR//ZNF568//MAN2A1//MBNL1//TMEM100//BTF3//TGFBR2//FOSL1//ACVR1//NOG//HAND2//DLL1//MEOX2//HAND1//LATS2//SOX17//RBBP8//SOX4//SOX11//DLC1//LRP6//PRKACB//TGFB2//TSC1//FZD6//CEBPB//PKD2//KBTBD8//EN1//LHX1//OSR1//TFAP2A//HOXA3//DSCAML1//ZEB1//SLC35D1//SULF1//HOXD1//GRB2//EGLN1//MAPK1//VASH2//BMPR2//IGF1// |
| GO:1902531 | regulation of intracellular signal transduction | Biological process | 143 | 1901 | 934 | 17653 | 1.42175762334036 | 7.26624938626831e-06 | 0.000235127486121209 | 5.13868970071752 | 0.153104925053533 | GADD45A//MINK1//TNF//MAP3K2//CRK//CRKL//MAP3K5//TAOK3//LAMTOR3//ADAM9//DUSP5//LPAR1//LPAR3//NRG1//IGF1//NTF3//PIK3CB//PRKAA1//MAPK1//MAP2K4//DUSP10//SPRED1//DUSP1//DUSP2//PPP2CA//RGS3//COPS8//MDFIC//PDE10A//AKAP11//PTEN//KLF4//KDR//TGFB2//PKD2//TSC1//FNIP1//RRAGD//RHEB//ARAP2//APPL1//AGFG1//AGFG2//PSD//GIT2//RALGPS2//IFNA1//FGD4//FGD5//EPS8//ARHGEF4//ARHGEF28//SOS1//SOS2//DLC1//MET//NF2//SOX11//NPLOC4//CNOT7//IL2//OSM//STAT3//ZFAND6//SQSTM1//GJA1//RHOC//NDFIP2//CC2D1A//MIER1//SLC20A1//UBE2I//ZMYND11//ESR1//TSG101//EDN1//GDF11//GRB2//INHBB//PAK6//BMP3//EFNA1//EPHA2//EPHA4//EPHA5//EPHA7//AR//ITGAV//FZD10//HIPK3//AIDA//PMAIP1//PEX5L//ADRB1//WNT7A//FLRT1//NEUROD1//ASPN//SOCS5//RASGEF1A//CMKLR1//TMEM100//PLEK//FAM13A//TAGAP//ARHGAP1//ARHGAP6//TRIP10//ARHGAP12//CD2AP//SLIT2//SESN3//EREG//F3//GAB1//OTUD3//PLEKHA1//CALM2//RANBP9//ERRFI1//LMO3//NPNT//DSTYK//NPTN//JUN//P2RY1//DNAJC27//FBXW7//CCL2//CCL8//HAND2//MDM4//POU4F1//RBBP7//RBBP8//BAG5//PIAS4//WFS1//BCL2L11//SERINC3//NCK1//SESN1//URI1// |
| GO:0033043 | regulation of organelle organization | Biological process | 102 | 1253 | 934 | 17653 | 1.53858234882962 | 7.3695869376519e-06 | 0.000237335792663523 | 5.13255685348773 | 0.109207708779443 | EPS8//TWF1//STMN2//MID1IP1//CDC23//CDC14A//CNOT6L//PAN3//CNOT6//BNIP3//TMED9//SQSTM1//CHMP3//HECW2//PFN2//LIMA1//CFL2//PLEK//SLIT2//CDC42EP3//GRB2//NCK1//JARID2//MET//SLAIN2//CLIP1//TBC1D12//EVI5//TBC1D17//USP6NL//NEUROG1//ATAD2//MIER1//DCP2//MAPK1//CCT6A//DLC1//FZD10//CRK//EFNA5//EPHA5//RHOC//RND3//TSC1//FMR1//RPS6KA4//RPS6KA5//IQGAP2//ARFIP1//CEP97//MTBP//EDN1//EREG//IGF1//RAD21//MDM1//KAT2B//WASL//PTGER4//CLIC4//LPAR1//PPM1E//NF2//PPM1F//S1PR1//TMEFF2//ARHGAP6//PPFIA1//KMT2B//DNMT3B//MYB//TMOD3//TMOD1//MSN//TNF//TMEM135//KDR//MIEF1//DDHD1//RNF4//BCL2L11//PMAIP1//MOAP1//DUSP1//DYNLL2//E2F1//PPP3R1//YWHAG//CALCOCO2//FYCO1//FEZ2//FBXW7//RAB3GAP1//TSG101//NRG1//ELMOD1//PRKAA1//SNIP1//CD2AP//NTF3//PIKFYVE//MTPN// |
| GO:0071363 | cellular response to growth factor stimulus | Biological process | 63 | 674 | 934 | 17653 | 1.76665724143628 | 7.87380760739504e-06 | 0.00025237232629769 | 5.10381520118758 | 0.0674518201284797 | COL1A2//JUN//TGFB2//TGFBR2//KLF10//ADAM9//ACVR1//ZFYVE9//SPRED1//FLRT1//GRB2//ESRP1//MAPK1//DKK3//SOX11//ZEB1//TOB1//SOSTDC1//RGMB//TMEM100//RGMA//BMP3//BMPR2//NOG//NPNT//TGFB1I1//BAMBI//ASPN//PMEPA1//PRDM16//RASL11B//LDLRAD4//SULF1//GATA6//CTDSPL2//ITGA5//KDR//ERRFI1//FOXC1//GAB1//NTF3//OTX2//FAM20C//SHISA2//DSTYK//LHX1//CCL2//NPTN//SOS1//CRK//EDN1//WNT7A//SLIT2//DLL1//STMN2//DYNC1LI2//E2F1//PTEN//PAX9//TGIF1//LRP8//URI1//KLF4// |
| GO:0031331 | positive regulation of cellular catabolic process | Biological process | 38 | 335 | 934 | 17653 | 2.14392917638787 | 7.95626363692439e-06 | 0.000253812315926979 | 5.09929083462697 | 0.0406852248394004 | PIK3CB//PRKAA1//BNIP3//SESN3//RAB12//SESN1//KDR//TSC1//RNF180//ANKIB1//LONRF3//LONRF1//SOCS5//IGF1//SH3D19//TNF//ADAM9//TOB1//CPEB3//CNOT7//BTG2//CNOT6L//YTHDF2//QKI//NANOS1//CALCOCO2//FYCO1//FMR1//FBXW7//FBXL5//MSN//EDEM1//ATXN3//BAG6//RNF128//LDLR//PTEN//RAB3GAP1// |
| GO:0007548 | sex differentiation | Biological process | 33 | 273 | 934 | 17653 | 2.2846671529755 | 8.26809239577723e-06 | 0.000262521637899725 | 5.08259467878495 | 0.0353319057815846 | CEBPB//FOXC1//INHBB//DMRTA1//IMMP2L//KMT2B//ESR1//EREG//PLEKHA1//ZFPM2//OSR1//RDH10//BCL2L11//RNF38//GATA6//TFAP2C//TGFB2//ARID5B//IDH1//LHX8//AR//TMF1//NEUROG1//LRP6//LHFPL2//FOXF2//TCF21//LHX1//SLIT2//TBC1D20//ARID4B//ARID4A//WNT7A// |
| GO:0071900 | regulation of protein serine/threonine kinase activity | Biological process | 51 | 508 | 934 | 17653 | 1.89748394004283 | 8.4979315685423e-06 | 0.000268558463542297 | 5.07068677048399 | 0.0546038543897216 | CDKN1A//GADD45A//PTEN//CCNE2//MINK1//TNF//MAP3K2//CRK//CRKL//MAP3K5//TAOK3//LAMTOR3//ADAM9//DUSP5//LPAR1//LPAR3//NRG1//IGF1//NTF3//PIK3CB//PRKAA1//MAPK1//MAP2K4//DUSP10//SPRED1//DUSP1//DUSP2//PPP2CA//RGS3//COPS8//MDFIC//CDK6//PKD2//TSG101//EDN1//FZD10//EPHA4//HIPK3//AIDA//LATS2//IPO5//KAT2B//CCNY//MAPRE3//CCNC//CCND2//LRP6//YWHAG//ACSL1//CALM2//PRKAR2B// |
| GO:0043087 | regulation of GTPase activity | Biological process | 49 | 482 | 934 | 17653 | 1.92141283197242 | 9.09158718980151e-06 | 0.000285983275184314 | 5.04136029195954 | 0.0524625267665953 | IQGAP2//FZD10//CPEB2//LRCH1//IPO5//TSC1//SLIT2//FAM13A//DLC1//CDC42EP3//GNB5//ARAP2//TAGAP//TBC1D20//ADRB1//DENND1B//S1PR1//RAB3GAP1//DOCK9//RGS17//GNAQ//AGFG1//AGFG2//ITGA6//JUN//ARHGAP1//ARHGAP6//ERRFI1//ELMOD1//RGS3//CCL2//CCL8//SOS1//EVI5//TRIP10//ARHGAP12//USP6NL//GIT2//EPHA2//TBC1D12//NTF3//TBC1D17//FGD4//CRK//FGD5//EFNA5//EPHA4//EPHA5//SBF2// |
| GO:0032990 | cell part morphogenesis | Biological process | 62 | 663 | 934 | 17653 | 1.76746086344273 | 9.16752539193249e-06 | 0.000287036917711294 | 5.03774787847007 | 0.0663811563169165 | NTNG1//SLITRK3//NEFH//OGN//POU4F1//DSCAML1//WNT7A//SLIT2//RANBP9//ARX//DPYSL2//EFNA1//EFNA5//EPHA5//GAB1//GRB2//KIF5A//OTX2//PIK3CB//ENAH//MAPK1//NTN4//SOS1//VLDLR//NR4A3//KLF7//RPS6KA5//FEZ2//EGR2//EPHA4//LHX1//NOG//NR4A2//PTEN//NFIB//RAB21//EPHA7//GOLGA4//NRG1//ULK2//LPAR3//GJA1//GPM6A//MNX1//NTF3//MINK1//HPRT1//FMR1//NSMF//HECW2//FXR1//BMPR2//SEMA4B//POU3F2//CAPRIN2//CTNND2//WASL//LRP8//BCL11B//SNX2//PLAA//FLRT1// |
| GO:0001568 | blood vessel development | Biological process | 67 | 736 | 934 | 17653 | 1.72055529047575 | 9.73287125131583e-06 | 0.000303333678675802 | 5.01175902153502 | 0.0717344753747323 | S1PR1//EFNA1//EPHA2//ELK3//EREG//FOXC1//GAB1//CLIC4//HOXA3//ITGA5//ITGAV//JUN//KDR//MEOX2//MMP2//TMEM100//PTEN//CCL2//SOX17//TGFB2//VEGFB//WNT7A//HAND1//HAND2//EDN1//PPP3R1//TGFBR2//ACVR1//GJC1//FOXF1//ZFPM2//QKI//RECK//SLIT2//SPRED1//SULF1//PPARG//KLF4//PKD2//SOX4//ETS1//EGLN1//VASH2//F3//GATA6//ITGB8//STAT3//BTG1//ADAM12//HMGA2//GJA1//SOS1//PIK3CB//FOSL1//MAPK1//BMPR2//DLL1//ADIPOR2//NOG//OSR1//TCF21//KLF2//EPN1//JAK1//COL1A2//CRKL//SPHK2// |
| GO:0006469 | negative regulation of protein kinase activity | Biological process | 32 | 263 | 934 | 17653 | 2.29967188021592 | 9.85531413496835e-06 | 0.000305740777499041 | 5.00632952816698 | 0.0342612419700214 | DUSP10//SPRED1//DUSP1//DUSP2//DUSP5//PPP2CA//RGS3//ERRFI1//TSG101//SOCS5//CDK6//PTEN//HIPK3//AIDA//PPM1F//CDKN1A//LATS2//IPO5//KAT2B//LRP6//YWHAG//PRKAR2B//GADD45A//PPM1E//FLRT1//GNAQ//NCK1//NF2//TAOK3//ASPN//DUS2//CAMK2N2// |
| GO:0016070 | RNA metabolic process | Biological process | 313 | 4821 | 934 | 17653 | 1.2270968776414 | 1.05288261721401e-05 | 0.0003251436137086 | 4.97762004433004 | 0.335117773019272 | E2F1//NPAT//RRM2//TSHZ1//HMG20A//KLF2//ZBTB18//CEBPB//ZNF268//TCERG1//WWP2//KLF12//OSR1//CRY2//GLIS3//ARX//DNMT3B//DR1//EDN1//EFNA1//CC2D1B//EN1//ESR1//FHL2//CPEB3//FOXF1//FOXC1//MYT1L//CIC//ZFPM2//DNAJB5//GATA6//CNOT7//HSBP1//AR//JARID2//JUN//ZNF568//MAF//MDM4//MYB//NFIA//NFIB//NR4A2//RLIM//KLF3//ZBTB7A//PIAS4//POU4F1//PPARG//CC2D1A//TSHZ3//ZBTB4//RARB//RBBP7//RBBP8//REST//RNF2//PRDM16//SOX17//SOX11//STAT3//ZEB1//TCF21//TFAP2A//TFAP2C//TGIF1//KLF10//TNF//UBE2I//VLDLR//WFS1//ZNF217//LRP8//BTG2//BHLHE41//TBL1XR1//NR4A3//HMGA2//ARID5B//BRMS1L//BHLHE40//RUNX3//URI1//PER2//SQSTM1//NOG//KLF4//HAND1//FNIP1//PHF14//USP3//DCP2//PPP2CA//RPL15//CNOT6L//PAN3//CNOT6//RBFOX3//FMR1//MBNL1//RBM25//SRSF2//ELAVL2//NOVA1//GTF2E1//DUS2//IRF9//KLF6//CREBRF//EGR2//ELK3//ERG//FOXJ3//FOXF2//FLI1//GRHL1//HIVEP2//IRF1//LHX1//MEF2D//MEOX2//MYBL1//NEUROD1//NHLH2//OTX2//PAX9//PBX3//POU3F2//RAD21//ARID4A//SHOX//BCL11B//BMPR2//SOX4//BTF3//TEF//TGFB1I1//THRA//TMF1//DEK//FOSL1//KLF7//CBFB//FUBP3//HAND2//CLOCK//POLR3G//PKD2//BAHD1//H2AFZ//KMT2B//DPY30//TOPORS//KHDRBS3//ZMYND11//TADA1//ZNF597//CTNND2//ZNF782//NKX2-3//ZNF800//E2F5//ZNF367//EZH1//ZBTB9//KDM2A//MYCBP2//FLII//EPC2//LHX6//HBP1//ATAD2//SERTAD3//MDFIC//MNX1//HMGB3//HOXA3//HOXD1//FOXN2//ZBTB41//ZBTB34//ATXN3//LHX8//NEUROG1//NFE2//NPAS2//IER5//ARID4B//BRWD1//BNC2//ZNF532//CDCA7L//PRKAA1//TRERF1//LMO3//MAPK1//SLC2A4RG//PHTF2//ZNF630//ZNF287//ZNF512B//ZBTB26//MIER1//RBL2//RNF4//ATXN1//DMRTA1//SMARCD2//STAT4//ZNF2//ZNF3//ZNF131//ZNF655//BRD3//TCF7L1//PCGF5//LCOR//WASL//ZBTB47//ST18//HIPK3//NUP35//NR5A2//NRBF2//MLLT6//RNF141//PAK6//SNAPC1//BTG1//SNIP1//RPS6KA4//RPS6KA5//CDKN1A//CRK//EMX2//ETS1//BOLA3//NR3C2//PLEK//ZNFX1//NKX2-4//RFX7//BDP1//CDK8//E2F3//TEAD1//KAT2B//CCNC//CMTR2//ZFC3H1//RTCA//PPP4R2//ESRP1//CPEB1//QKI//G3BP2//EREG//DDX6//INTS8//RBM47//MAP3K2//MAPRE3//BAMBI//RGMB//IGF1//LRP6//MAP3K5//WNT7A//ACVR1//DKK3//NRG1//RASD1//TSG101//BTAF1//PPM1F//TOB1//SOSTDC1//TMEM100//RGMA//BMP3//TGFB2//SULF1//CTDSPL2//CMKLR1//EPHA5//NABP1//EGLN1//FZD6//FASTK//AHNAK//YTHDF2//FXR1//S1PR1//DLL1//IL2//ITGA6//MET//PPP1R12A//NCK1//OSM//P2RY1//PPP3R1//DCAF6//CAPRIN2//PTEN//MTPN//EGLN3//NANOS1//RAP2C//TOB2//FBXW7//NPNT// |
| GO:0050890 | cognition | Biological process | 33 | 277 | 934 | 17653 | 2.25167556953904 | 1.12444199502995e-05 | 0.000345663691472161 | 4.9490629429746 | 0.0353319057815846 | DRD1//EGR2//LHX8//MAPK1//PTEN//THRA//CIC//SLC24A2//JUN//PRKAR2B//SLC12A5//ATXN1//SLC6A1//FOSL1//CEBPB//ITGA5//NTF3//PAIP2//KCNK10//VLDLR//KMT2B//CPEB3//GRIA1//LDLR//SLC17A7//CCND2//SNAP25//BTG2//ADCY3//NPTN//EN1//NEUROG1//SOBP// |
| GO:0030900 | forebrain development | Biological process | 40 | 366 | 934 | 17653 | 2.06562057546717 | 1.13206191555788e-05 | 0.000346431435969138 | 4.94612981974688 | 0.0428265524625268 | LHX1//LRP8//CDK6//DRD1//EMX2//NEUROD1//PTEN//BTG2//HPRT1//RARB//ARX//SLC32A1//EPHA5//EZH1//NF2//NME1//TSC1//NR4A3//BCL11B//LRP6//LHX6//POU3F2//SLIT2//NRG1//WNT7A//ETS1//RAB3GAP1//GNAQ//LHX8//C21ORF91//NFIB//NOG//NR4A2//POU4F1//TACC2//BNIP3//LPAR1//DLC1//E2F1//OTX2// |
| GO:1901700 | response to oxygen-containing compound | Biological process | 121 | 1565 | 934 | 17653 | 1.46131106717475 | 1.16819614912401e-05 | 0.000354526869859614 | 4.93248422971501 | 0.129550321199143 | CHRM2//CHRM5//GNAQ//GAB1//APPL1//GRB2//SOS1//PIK3R3//DRD1//RAP1B//SLC6A1//GJA1//IGF1//NEUROD1//PTEN//TGFBR2//EDN1//CEBPB//COL1A2//SESN3//CPEB3//NSMF//SESN1//GLRA1//IPO5//MMP2//RRAGD//CPEB1//ZEB1//TNF//CPEB4//LAMTOR3//MAPK1//CCL2//DNMT3B//DUSP1//ESR1//ETS1//GJB2//RBBP8//STAT3//WNT7A//DUSP10//TNFRSF21//JUN//UGT1A1//PTGER4//PPARG//RARB//TGFB2//FOSL1//EGR2//TSC1//CPEB2//INHBB//ERRFI1//SLC2A4//YWHAG//KAT2B//STX2//SRSF2//TRPC3//NME1//ACSL1//PKD2//SYBU//CRK//NR4A3//PPP1R15B//ADAM9//GLRB//EREG//LRP6//BTG1//BTG2//IL2//RPL15//ACTC1//EPS8//USP46//NCK1//CDKN1A//SLIT2//EFNA5//EPHA5//KLF2//MAP3K5//MYB//PRKAA1//PLEKHA1//BNIP3//KLF4//CDK19//CD274//PLSCR4//PDCD1LG2//PLAA//FZD10//OSR1//HCN2//SOX4//NR4A2//ADCY3//PRKACB//PRKAR2B//H2AFZ//POU4F1//AR//MSN//LRP8//E2F1//LDLR//FIBIN//EGLN1//MAP2K4//EDEM1//MET//KLF3//KLF10//LPAR1//EPHA4// |
| GO:0031647 | regulation of protein stability | Biological process | 31 | 253 | 934 | 17653 | 2.31586275190223 | 1.17270345436264e-05 | 0.000354526869859614 | 4.93081179557085 | 0.0331905781584582 | CDKN1A//CRTAP//PTGES3//EFNA1//EPHA4//OTUD3//MORC3//GNAQ//IGF1//USP27X//MDM4//PFN2//FBXW7//CDC37L1//PTEN//SOX17//MTMR9//SOX4//TSC1//WFS1//BAG6//CCT6A//MYLIP//ASPH//NF2//CTSA//ELMOD1//MAPK1//USP28//RNF128//USP3// |
| GO:0042326 | negative regulation of phosphorylation | Biological process | 48 | 473 | 934 | 17653 | 1.91801386204055 | 1.17424247890808e-05 | 0.000354526869859614 | 4.93024221281742 | 0.0513918629550321 | DUSP10//SPRED1//DUSP1//DUSP2//DUSP5//PPP2CA//RGS3//FAM129A//IL2//LRP6//PTEN//PPP1R15B//SLIT2//ERRFI1//TSG101//SOCS5//GADD45A//PPM1E//FLRT1//GNAQ//NCK1//NF2//TAOK3//ASPN//DUS2//YWHAG//CAMK2N2//PPM1F//CALM2//CDK6//JUN//EFNA1//HIPK3//AIDA//CDKN1A//LATS2//IPO5//KAT2B//STAT3//ZMYND11//NTF3//PMEPA1//LDLRAD4//NOG//RANBP9//LMO3//KLF4//PRKAR2B// |
| GO:0051130 | positive regulation of cellular component organization | Biological process | 99 | 1221 | 934 | 17653 | 1.53246715666416 | 1.19633989614363e-05 | 0.00035959318744975 | 4.92214541395303 | 0.105995717344754 | FMR1//NTF3//CNOT6L//PAN3//CNOT6//TMED9//STMN2//NPTN//NCK1//NME1//SERPINI1//TWF1//ZNF804A//CFL2//PLEK//CDC42EP3//GRB2//PFN2//MET//SLAIN2//CLIP1//TNF//FNIP1//NEUROG1//MIER1//MAPK1//CCT6A//PMAIP1//BCL2L11//RHOC//RPS6KA4//RPS6KA5//ADAM9//IQGAP2//ARFIP1//BNIP3//AR//TGFB2//GOLGA4//NRG1//DLL1//LDLR//EDN1//EREG//IGF1//RAD21//ESR1//RAB21//EFNA5//LPAR3//BMPR2//SLIT2//EPHA4//CAPRIN2//WASL//GPM6A//LPAR1//PPM1E//NF2//TSC1//PPM1F//DNMT3B//MYB//JARID2//KDR//SLITRK3//FLRT1//WNT7A//ITGAV//PPARG//CPEB3//IL2//C21ORF91//LRP8//MSN//MIEF1//DDHD1//MOAP1//VLDLR//CRK//CRKL//DOCK5//EPS8//DYNLL2//E2F1//PPP3R1//YWHAG//CALCOCO2//FYCO1//RAB3GAP1//PLAA//FBXW7//ELMOD1//PRKAA1//SNIP1//JUN//FOSL1//TSG101//CCL2// |
| GO:0060562 | epithelial tube morphogenesis | Biological process | 36 | 316 | 934 | 17653 | 2.15321334670534 | 1.24465711690502e-05 | 0.000372460888567639 | 4.90495027326356 | 0.0385438972162741 | EDN1//PPP3R1//TGFBR2//ACVR1//NPNT//LHX1//PKD2//TCF21//SOX4//SOX11//DLC1//LRP6//PRKACB//TGFB2//TSC1//FZD6//NOG//GJA1//DLL1//SOX17//HAND1//HAND2//CLIC4//MET//SLIT2//RDH10//FOXF1//EPHA2//AR//SOSTDC1//ESR1//TNF//KLHL3//OSR1//EPHA4//EPHA7// |
| GO:0045664 | regulation of neuron differentiation | Biological process | 57 | 600 | 934 | 17653 | 1.79554068522484 | 1.33242359084506e-05 | 0.000396968314752649 | 4.87535768663597 | 0.0610278372591006 | PTEN//SNAP25//ZNF804A//STMN2//NPTN//NCK1//NME1//SERPINI1//TWF1//LPAR1//EPHA4//MYLIP//PTPRG//SPOCK1//TSC1//BAG5//DPYSL2//RAB21//WNT7A//DLL1//HMG20A//DDX6//REST//DNMT3B//MMD//NEUROD1//NEUROG1//RARB//SOX11//ZEB1//GOLGA4//NRG1//EPHA7//ULK2//EFNA5//LPAR3//KLF4//RGMA//FMR1//NSMF//HECW2//FXR1//BMPR2//SEMA4B//EFNA1//POU3F2//SLIT2//CAPRIN2//CPEB3//IL2//C21ORF91//LRP8//VLDLR//PLAA//NTF3//BCL11B//YWHAG// |
| GO:0003007 | heart morphogenesis | Biological process | 30 | 243 | 934 | 17653 | 2.33338620562032 | 1.39276744672119e-05 | 0.000413126589569097 | 4.85612139257609 | 0.0321199143468951 | GJA1//DLL1//PKD2//SOX17//TGFBR2//HAND1//HAND2//ACVR1//ZFPM2//GATA6//RARB//BMPR2//TGFB2//NOG//JUN//SOX11//TMEM100//EFNA1//ERG//SOS1//SOX4//FOXF1//NRG1//POU4F1//S1PR1//EGLN1//ACTC1//FOXC1//FHL2//DLC1// |
| GO:0007265 | Ras protein signal transduction | Biological process | 47 | 463 | 934 | 17653 | 1.91861798807701 | 1.43382524777465e-05 | 0.000423448041515282 | 4.84350377652868 | 0.0503211991434689 | CDC42EP3//COL1A2//EPS8//RHOC//RND3//ARHGAP1//ARHGAP6//PAK6//ARAP2//APPL1//AGFG1//AGFG2//PSD//GIT2//RAB12//RAB21//RAB38//RAB30//RAB9B//DNAJC27//RAB23//RAB22A//RAB28//RALGPS2//RAP2C//RAP1B//CRK//FGD4//FGD5//ARHGEF4//ARHGEF28//SOS1//SOS2//DLC1//MET//LPAR1//SQSTM1//RASGEF1A//NRG1//IGF1//TGFB2//CDKN1A//CRKL//GRB2//JUN//USP28//G3BP2// |
| GO:0010243 | response to organonitrogen compound | Biological process | 80 | 937 | 934 | 17653 | 1.61369718382281 | 1.44282620239273e-05 | 0.00042425363507748 | 4.84078597929293 | 0.0856531049250535 | DRD1//HPRT1//NR4A2//GAB1//APPL1//GRB2//SOS1//PIK3R3//SLC6A1//NME1//CEBPB//COL1A2//SESN3//CPEB3//NSMF//SESN1//GLRA1//IPO5//MMP2//RRAGD//CPEB1//ZEB1//TNF//CPEB4//LAMTOR3//UBXN4//DNAJB9//DERL2//JKAMP//NPLOC4//WFS1//BAG6//EDEM1//DNMT3B//PPARG//PRKAA1//EGR2//IGF1//TSC1//CPEB2//INHBB//ERRFI1//PTEN//SLC2A4//YWHAG//KAT2B//TRPC3//DNAJB12//EDN1//GLRB//EREG//GJA1//LRP6//BTG1//BTG2//NCK1//DUSP1//JUN//FOSL1//STAT3//MAPK1//CRK//PKD2//RAP1B//HCN2//NR4A3//ADCY3//GJB2//PRKACB//PRKAR2B//LDLR//SOCS5//KLF2//KLF4//SLIT2//KLF3//KLF10//ATXN3//EPHA4//CDKN1A// |
| GO:0032956 | regulation of actin cytoskeleton organization | Biological process | 37 | 331 | 934 | 17653 | 2.11273669433357 | 1.45143092889329e-05 | 0.000424936249874689 | 4.83820362666954 | 0.0396145610278373 | EPS8//TWF1//PFN2//LIMA1//CFL2//PLEK//SLIT2//CDC42EP3//GRB2//NCK1//IQGAP2//ARFIP1//WASL//PTGER4//TSC1//LPAR1//PPM1E//NF2//PPM1F//DLC1//S1PR1//TMEFF2//ARHGAP6//MET//PPFIA1//TMOD3//TMOD1//EDN1//CD2AP//NTF3//MTPN//FZD10//CRK//EFNA5//EPHA5//RHOC//RND3// |
| GO:0030855 | epithelial cell differentiation | Biological process | 68 | 760 | 934 | 17653 | 1.69109095007326 | 1.47105819726771e-05 | 0.000428826146039721 | 4.8323701455918 | 0.0728051391862955 | CLIC4//MET//ESR1//RARB//GJA1//GATA6//CDK6//DLL1//NEUROD1//PALLD//BCL11B//AR//FRMD6//KLF4//WNT7A//EPHA2//EREG//LATS2//TSG101//ADAM9//KIAA1109//OSR1//DSC2//KRT27//JUN//FOXF1//MSN//S1PR1//ZEB1//TMEM100//BTG1//GRHL1//ERRFI1//CBFB//FOXC1//TCF21//NFIB//PPP3R1//KLF2//THRA//SOX4//SOX11//CDKN1A//CEBPB//BMPR2//NRG1//SOX17//RAP1B//ACVR1//MAF//NF2//TMOD1//TBC1D20//FRZB//FAM20C//ARX//RAP2C//ARID4B//ARID4A//TNF//RHEB//CLOCK//LHX1//CNN3//NR5A2//GSTA2//PPARG//TGFB1I1// |
| GO:0030278 | regulation of ossification | Biological process | 25 | 185 | 934 | 17653 | 2.5541119277736 | 1.52901414762136e-05 | 0.000443807840358938 | 4.81558849614068 | 0.0267665952890792 | S1PR1//GJA1//OSR1//FAM20C//BMPR2//TFAP2A//ACVR1//PKDCC//SMOC1//TOB1//CDK6//TMEM64//TNF//NOG//HAND2//CEBPB//NPNT//IGF1//SOX11//TOB2//TGFB2//EGR2//LRP6//MAPK1//PTGER4// |
| GO:0045137 | development of primary sexual characteristics | Biological process | 28 | 221 | 934 | 17653 | 2.39462439563208 | 1.64990272892113e-05 | 0.000476850092123658 | 4.78254165914249 | 0.0299785867237687 | CEBPB//FOXC1//INHBB//DMRTA1//IMMP2L//KMT2B//ESR1//EREG//PLEKHA1//ZFPM2//OSR1//RDH10//BCL2L11//RNF38//GATA6//TFAP2C//TGFB2//ARID5B//IDH1//LHX8//AR//TMF1//LHFPL2//TCF21//SLIT2//TBC1D20//ARID4B//ARID4A// |
| GO:0060996 | dendritic spine development | Biological process | 16 | 90 | 934 | 17653 | 3.36007613609327 | 1.77822211824443e-05 | 0.000509673173433638 | 4.75001399212682 | 0.0171306209850107 | CTNND2//PTEN//WNT7A//WASL//CPEB3//FMR1//FXR1//LPAR1//NRG1//IL2//C21ORF91//EPHA4//EFNA1//CAPRIN2//LRP8//SLC12A5// |
| GO:0045444 | fat cell differentiation | Biological process | 27 | 210 | 934 | 17653 | 2.43005506271031 | 1.77928985575046e-05 | 0.000509673173433638 | 4.74975329727924 | 0.0289079229122056 | E2F1//ZFPM2//ZADH2//TGFB1I1//TNF//CEBPB//CMKLR1//CCDC71L//TMEM64//FRZB//PPARG//LMO3//PRDM16//TBL1XR1//PER2//ADRB1//ITGA6//SLC2A4//BNIP3//DUSP10//EGR2//INHBB//NR4A2//NR4A3//HMGA2//ARID5B//KLF4// |
| GO:0030334 | regulation of cell migration | Biological process | 74 | 853 | 934 | 17653 | 1.6396620066223 | 1.78607928587568e-05 | 0.000509673173433638 | 4.74809926624589 | 0.0792291220556745 | MIA3//CCL8//SLIT2//EDN1//ETS1//KDR//BMPR2//WNT7A//DUSP10//PFN2//PTPRG//JUN//TGFB2//TGFBR2//DOCK5//PPM1F//CMKLR1//C5ORF30//ADAM10//CRK//IGF1//NRG1//SEMA4B//ZNF268//DRD1//S1PR1//F3//FOXF1//CD274//ITGA6//ITGA5//ITGAV//NTF3//MAPK1//PTP4A1//ACVR1//DLC1//SULF1//TMEFF2//CLIC4//NF2//PTEN//RAP2C//LDLRAD4//RECK//NOG//GAB1//EFNA1//EPHA2//PPARG//ADAM9//VEGFB//LPAR1//TNF//MPP1//SPRED1//MEOX2//KLF4//MSN//WASL//LRCH1//PTGER4//CCL2//CXCL14//MET//STAT3//NSMF//PLAA//RHOC//RND3//LAMA3//NCK1//MINK1//SLK// |
| GO:0051348 | negative regulation of transferase activity | Biological process | 35 | 309 | 934 | 17653 | 2.14082520806913 | 1.85273376608299e-05 | 0.000526001419547716 | 4.7321869833946 | 0.0374732334047109 | DUSP10//SPRED1//DUSP1//DUSP2//DUSP5//PPP2CA//RGS3//ERRFI1//TSG101//SOCS5//GADD45A//PPM1E//FLRT1//GNAQ//LRP6//NCK1//NF2//TAOK3//ASPN//DUS2//YWHAG//CAMK2N2//PPM1F//CDK6//PTEN//HIPK3//AIDA//CDKN1A//LATS2//IPO5//KAT2B//BAG5//PPARG//PRKAR2B//CHCHD10// |
| GO:0051347 | positive regulation of transferase activity | Biological process | 61 | 664 | 934 | 17653 | 1.73633452439308 | 1.85885463953725e-05 | 0.000526001419547716 | 4.73075457026851 | 0.0653104925053533 | GADD45A//MINK1//TNF//MAP3K2//CRK//CRKL//MAP3K5//TAOK3//LAMTOR3//ADAM9//DUSP5//LPAR1//LPAR3//NRG1//IGF1//NTF3//PIK3CB//PRKAA1//MAPK1//MAP2K4//EREG//FBXW7//EFNA5//COPS8//MDFIC//PKD2//PAK6//TGFB2//TGFBR2//SLK//EPHA2//EPHA4//EPHA5//EPHA7//DSTYK//KDR//MET//ADCY3//PRKACB//PRKAR2B//EDN1//FZD10//CCNY//MAPRE3//CCNC//CCND2//CDKN1A//MMD//VLDLR//TOPORS//ARRDC3//ARRDC4//PTGES3//KLF4//EFNA1//LRP8//ACSL1//PPP2CA//CALM2//UBE2I//PTEN// |
| GO:0071417 | cellular response to organonitrogen compound | Biological process | 52 | 537 | 934 | 17653 | 1.83020906854242 | 1.89449766293487e-05 | 0.000533853653934522 | 4.72250592613727 | 0.0556745182012848 | GAB1//APPL1//GRB2//SOS1//PIK3R3//CEBPB//COL1A2//SESN3//CPEB3//NSMF//SESN1//GLRA1//IPO5//MMP2//RRAGD//CPEB1//ZEB1//TNF//CPEB4//LAMTOR3//CPEB2//INHBB//ERRFI1//PPARG//PTEN//SLC2A4//YWHAG//KAT2B//NCK1//TSC1//IGF1//STAT3//MAPK1//PKD2//RAP1B//HCN2//EDN1//NR4A2//NR4A3//ADCY3//GJB2//PRKACB//PRKAR2B//KLF2//KLF4//SLIT2//DRD1//KLF3//KLF10//EPHA4//CRK//PRKAA1// |
| GO:0006807 | nitrogen compound metabolic process | Biological process | 610 | 10390 | 934 | 17653 | 1.10964978267276 | 1.90582589252862e-05 | 0.000534817448596309 | 4.71991677702815 | 0.653104925053533 | IGF1//NFIA//NFIB//RBBP7//RBBP8//RRM2//CDKN1A//GADD45A//PTEN//CCNE2//E2F1//NPAT//MTRR//TSHZ1//HMG20A//KLF2//ZBTB18//CEBPB//ZNF268//TCERG1//WWP2//KLF12//OSR1//CRY2//GLIS3//ARX//DNMT3B//DR1//EDN1//EFNA1//CC2D1B//EN1//ESR1//FHL2//CPEB3//FOXF1//FOXC1//MYT1L//CIC//ZFPM2//DNAJB5//GATA6//CNOT7//HSBP1//AR//JARID2//JUN//ZNF568//MAF//MDM4//MYB//NR4A2//RLIM//KLF3//ZBTB7A//PIAS4//POU4F1//PPARG//CC2D1A//TSHZ3//ZBTB4//RARB//REST//RNF2//PRDM16//SOX17//SOX11//STAT3//ZEB1//TCF21//TFAP2A//TFAP2C//TGIF1//KLF10//TNF//UBE2I//VLDLR//WFS1//ZNF217//LRP8//BTG2//BHLHE41//TBL1XR1//NR4A3//HMGA2//ARID5B//BRMS1L//BHLHE40//RUNX3//URI1//PER2//SQSTM1//NOG//KLF4//HAND1//FNIP1//PHF14//USP3//RANBP9//PPM1L//SPRED1//DUSP5//EREG//RASGEF1A//GRB2//NRG1//IL2//JAK1//MAP3K5//MET//MINK1//TAOK3//MAPK1//CCL2//SOS1//LAMTOR3//DCP2//PPP2CA//RPL15//MAP3K2//CRK//CRKL//ADAM9//LPAR1//LPAR3//NTF3//PIK3CB//PRKAA1//MAP2K4//DUSP10//DUSP1//DUSP2//RGS3//TOPORS//HECTD2//RNF145//ZNRF2//FBXL5//RNF180//MYLIP//ANKIB1//UBE2W//FBXW7//HECW2//SKP1//THOP1//LONRF3//FBXO30//CBFB//UBE3B//LONRF1//ZFAND4//CNOT6L//PAN3//CNOT6//COPS8//RBFOX3//FMR1//MBNL1//RBM25//SRSF2//ELAVL2//NOVA1//PTGES3//NABP1//GTF2E1//SECISBP2L//ITGB8//ST6GALNAC3//ST6GALNAC6//ST8SIA3//ST3GAL5//DRD1//FAM129A//LRP6//PPP1R15B//SLIT2//EFNA5//EPHA7//NPTN//KDR//P2RY1//AKTIP//CCND2//LYPLA1//F3//DUS2//IRF9//KLF6//CREBRF//EGR2//ELK3//ERG//FOXJ3//FOXF2//FLI1//GRHL1//HIVEP2//IRF1//LHX1//MEF2D//MEOX2//MYBL1//NEUROD1//NHLH2//OTX2//PAX9//PBX3//POU3F2//RAD21//ARID4A//SHOX//BCL11B//BMPR2//SOX4//BTF3//TEF//TGFB1I1//THRA//TMF1//DEK//FOSL1//KLF7//FUBP3//HAND2//CLOCK//POLR3G//PKD2//ERRFI1//TSG101//SOCS5//TRIM3//KLHL2//RNF38//MYCBP2//FBXO33//KLHL3//SPOPL//DCAF8//DCAF6//FEM1C//KLHL14//MARCH4//RNF4//MARCH7//UBE2Z//RNF128//KLHL15//KBTBD8//ARRDC4//UGP2//HS3ST5//XYLT2//HS2ST1//HS3ST3A1//HS3ST1//GNS//GFPT2//NAGK//SLC35D1//NUP35//NUP43//CHCHD10//COX8C//COX7A2L//GSTA2//IDH1//DPYSL2//TTR//HPRT1//NME1//CMPK1//ADCY3//IMPDH1//PDE10A//DCTPP1//REV3L//GMNC//ESCO2//EPC2//USP28//USP45//ATXN3//KDM2A//HMGB3//BAHD1//H2AFZ//KMT2B//DPY30//ARID4B//KHDRBS3//ZMYND11//TADA1//ZNF597//CTNND2//ZNF782//NKX2-3//ZNF800//E2F5//ZNF367//EZH1//ZBTB9//FLII//LHX6//HBP1//ATAD2//SERTAD3//MDFIC//MNX1//HOXA3//HOXD1//FOXN2//ZBTB41//ZBTB34//LHX8//NEUROG1//NFE2//NPAS2//IER5//BRWD1//BNC2//ZNF532//CDCA7L//TRERF1//LMO3//SLC2A4RG//PHTF2//ZNF630//ZNF287//ZNF512B//ZBTB26//MIER1//RBL2//ATXN1//DMRTA1//SMARCD2//STAT4//ZNF2//ZNF3//ZNF131//ZNF655//BRD3//TCF7L1//PCGF5//LCOR//WASL//ZBTB47//ST18//HIPK3//NR5A2//NRBF2//MLLT6//RNF141//PAK6//SNAPC1//BTG1//SNIP1//RPS6KA4//RPS6KA5//EMX2//ETS1//BOLA3//NR3C2//PLEK//ZNFX1//NKX2-4//RFX7//BDP1//CDK8//E2F3//TEAD1//KAT2B//CCNC//CMTR2//ZFC3H1//RTCA//PPP4R2//ESRP1//CPEB1//QKI//CPEB2//CPEB4//MTPN//DDX6//MKNK2//ACO1//TSC1//SERP1//UEVLD//RIMKLB//IMMP2L//ADAM10//CDK6//FASTK//PDIK1L//CDK19//MORC3//MMD//DSTYK//LATS2//CDK17//PRKACB//FAM20C//CCL8//TGFB2//TGFBR2//ACVR1//STK17B//PPM1E//FLRT1//GNAQ//NCK1//NF2//ASPN//YWHAG//CAMK2N2//PPM1F//PTPN21//PPP1R12A//CTDSPL2//PPP3R1//PPP6C//PTPN4//NAA30//PCMTD1//ASPH//FUT9//ST8SIA5//MAN1A1//MAN2A1//B3GNT5//B3GALT2//CCDC126//EDEM1//VEGFB//NTNG1//RECK//PTAR1//PIGA//MMP2//CTSA//NLN//NPEPL1//ADAM12//ADAM18//LMLN//ZFYVE9//USP24//USP27X//NPLOC4//USP46//BAG6//CDC23//SLC6A14//SLC6A6//GLUD2//LGSN//SLC25A32//BCAT2//SERINC3//PSAT1//ABHD3//SLC44A1//CDS1//SGPP1//SPHK2//NSMAF//ORMDL3//UGT1A4//UGT1A1//DLC1//EGLN3//PMAIP1//DLL1//G3BP2//PTGER4//EPHA2//PTHLH//CALM2//GDF11//INHBB//BMP3//LDLR//SERPINI1//SPOCK1//PCOLCE2//ACOT7//PPCS//GLRX//PRR16//FXR1//INTS8//GNPTG//ST3GAL3//RBM47//MAPRE3//BAMBI//RGMB//WNT7A//DKK3//RASD1//BTAF1//DMWD//OTUD3//WDR20//JOSD1//TOB1//NANOS1//CAPRIN2//VKORC1L1//CEP41//STK33//MASTL//EPHA4//EPHA5//PKDCC//OGN//CHST1//TMX3//ZDHHC1//ST6GAL2//MCFD2//ZDHHC5//CRTAP//EGLN1//SLK//SPTSSA//ELOVL4//ELOVL7//SULF1//DSEL//CHST7//UBXN4//DNAJB9//DERL2//JKAMP//SOSTDC1//TMEM100//RGMA//ADRB1//BAG5//NDFIP2//RAP2C//CMKLR1//CCT6A//PPP1R37//OSM//PFN2//IFNA1//UXS1//PRKAR2B//CNEP1R1//NSMF//PTPDC1//PTPRG//PTP4A1//CDC14A//BNIP3//OSBPL5//DNAJB12//ITM2B//TDRD5//ARL6IP1//UBXN2B//CD2AP//PLAA//VPS37A//BCL2L11//ITGAV//FZD6//AHNAK//YTHDF2//FZD10//AIDA//TWF1//CKAP4//RAB38//MIA3//ANO8//RAB12//GJA1//NSF//IPO5//CCNY//S1PR1//ITGA6//PAIP2//MPP1//RAB23//ENPP4//ULK2//ITGA5//SH3D19//ARRDC3//PMEPA1//LDLRAD4//PFKP//ATG16L1//NAPEPLD//NPNT//DNAJC27//UBA5//ACSL1//REXO1//ZC3H12C//RAB3GAP1//MSN//TOB2//FRY//UNK// |
| GO:0048736 | appendage development | Biological process | 24 | 176 | 934 | 17653 | 2.57733112711699 | 1.93154871499504e-05 | 0.000537574648539566 | 4.71409433417371 | 0.0256959314775161 | LRP6//MBNL1//TGFB2//ASPH//OSR1//RDH10//EN1//TFAP2A//WNT7A//RECK//RARB//ITGA6//FZD6//GJA1//GNAQ//NOG//HAND2//MEOX2//SMOC1//BMPR2//KAT2B//SOX4//SOX11//BCL2L11// |
| GO:0060173 | limb development | Biological process | 24 | 176 | 934 | 17653 | 2.57733112711699 | 1.93154871499504e-05 | 0.000537574648539566 | 4.71409433417371 | 0.0256959314775161 | LRP6//MBNL1//TGFB2//ASPH//OSR1//RDH10//EN1//TFAP2A//WNT7A//RECK//RARB//ITGA6//FZD6//GJA1//GNAQ//NOG//HAND2//SOX4//SOX11//BCL2L11//MEOX2//SMOC1//BMPR2//KAT2B// |
| GO:0043010 | camera-type eye development | Biological process | 34 | 297 | 934 | 17653 | 2.16368539066612 | 1.94513399513103e-05 | 0.000539136934797998 | 4.71105047591073 | 0.0364025695931477 | GJA1//TGFBR2//SOX11//TFAP2A//RARB//GPM6A//SLC17A7//TGFB2//TOPORS//FJX1//LHX1//RDH10//BCL11B//KLF4//NEUROD1//GDF11//FOXF2//LRP6//ZEB1//DLL1//MDM1//MAN2A1//JUN//SOS1//BMPR2//CLIC4//MAF//NF2//TMOD1//WNT7A//TBC1D20//EPHA2//RAB3GAP1//FOXC1// |
| GO:1901699 | cellular response to nitrogen compound | Biological process | 59 | 637 | 934 | 17653 | 1.750589117215 | 2.00483443369272e-05 | 0.000553416133676076 | 4.69792148713488 | 0.063169164882227 | CHRM2//CHRM5//GNAQ//GAB1//APPL1//GRB2//SOS1//PIK3R3//SNIP1//CEBPB//COL1A2//SESN3//CPEB3//NSMF//SESN1//GLRA1//IPO5//MMP2//RRAGD//CPEB1//ZEB1//TNF//CPEB4//LAMTOR3//CPEB2//INHBB//ERRFI1//PPARG//PTEN//SLC2A4//YWHAG//KAT2B//NCK1//TSC1//IGF1//STAT3//MAPK1//PKD2//RAP1B//HCN2//EDN1//NR4A2//NR4A3//ADCY3//GJB2//PRKACB//PRKAR2B//KLF2//KLF4//PRKAA1//SLIT2//CRK//DRD1//FMR1//KLF3//KLF10//MAP3K5//ESR1//EPHA4// |
| GO:2000765 | regulation of cytoplasmic translation | Biological process | 8 | 24 | 934 | 17653 | 6.30014275517488 | 2.05100403422998e-05 | 0.000563221140548503 | 4.68803348539365 | 0.00856531049250535 | CPEB2//CPEB3//NCK1//YTHDF2//FMR1//CPEB1//CPEB4//UNK// |
| GO:0061564 | axon development | Biological process | 49 | 497 | 934 | 17653 | 1.86342250505172 | 2.05701052366524e-05 | 0.000563221140548503 | 4.68676348644915 | 0.0524625267665953 | NTNG1//SLITRK3//NEFH//OGN//POU4F1//DSCAML1//WNT7A//SLIT2//RANBP9//ARX//DPYSL2//EFNA1//EFNA5//EPHA5//GAB1//GRB2//KIF5A//OTX2//PIK3CB//ENAH//MAPK1//NTN4//SOS1//VLDLR//NR4A3//KLF7//RPS6KA5//FEZ2//EGR2//EPHA4//LHX1//NOG//NR4A2//PTEN//NFIB//RAB21//JUN//EPHA7//GOLGA4//NRG1//ULK2//LPAR3//KLF4//RGMA//BMPR2//SEMA4B//POU3F2//BCL11B//NEFM// |
| GO:0007411 | axon guidance | Biological process | 30 | 248 | 934 | 17653 | 2.28634212889411 | 2.07204153005951e-05 | 0.000565049067249696 | 4.68360154424779 | 0.0321199143468951 | EGR2//EPHA4//LHX1//NOG//SLIT2//EFNA5//EPHA7//BMPR2//SEMA4B//BCL11B//NFIB//RANBP9//ARX//DPYSL2//EFNA1//EPHA5//GAB1//GRB2//KIF5A//OTX2//PIK3CB//ENAH//MAPK1//NTN4//SOS1//VLDLR//NR4A3//KLF7//RPS6KA5//FEZ2// |
| GO:0048732 | gland development | Biological process | 44 | 430 | 934 | 17653 | 1.93399731089089 | 2.2019759071295e-05 | 0.000598004780338955 | 4.6571874371617 | 0.0471092077087794 | GATA6//JARID2//JUN//MAN2A1//MET//PKD2//UGT1A1//RARB//ARID5B//TGFB2//NME1//ETS1//NOG//POU3F2//NKX2-3//MSN//DKK3//AR//HOXA3//MAPK1//THRA//BCL2L11//NRG1//FOXC1//CEBPB//EPHA2//CRKL//BCL11B//HAND2//GJA1//TGFBR2//LRP6//ESR1//SOSTDC1//NFIB//TNF//NTN4//SULF1//BTBD7//PTEN//FOXF1//FRZB//FBXW7//TCF21// |
| GO:0006928 | movement of cell or subcellular component | Biological process | 152 | 2090 | 934 | 17653 | 1.37457660112906 | 2.21057511584709e-05 | 0.000598004780338955 | 4.65549472308173 | 0.162740899357602 | SEMA4B//ACVR1//DCX//GJA1//GPM6A//LRP12//NR4A2//SPOCK1//UNK//KDR//SLIT2//CCL2//CCL8//MIA3//HAND2//FRMD6//CD2AP//NCK1//DYNC1LI2//KIF5A//MYO1B//KIF13A//KIF23//RANBP9//ARX//DPYSL2//EFNA1//EFNA5//EPHA5//GAB1//GRB2//OTX2//PIK3CB//ENAH//MAPK1//NTN4//SOS1//VLDLR//NR4A3//KLF7//RPS6KA5//FEZ2//EGR2//EPHA4//LHX1//NOG//RAB21//FMR1//FXR1//P2RY1//TGFB2//FOXC1//ACTC1//WASL//EDN1//ETS1//BMPR2//WNT7A//NANOS1//DUSP10//PFN2//PTPRG//JUN//TGFBR2//DOCK5//PPM1F//CMKLR1//ARID5B//C5ORF30//ADAM10//DYNLL2//CRK//IGF1//S1PR1//EPHA2//ERG//RASGEF1A//PALLD//BAMBI//JAK1//RHOC//RND3//PAK6//PTEN//BTG1//ADAM9//SYBU//POU4F1//LHX6//POU3F2//NRG1//DRD1//EMX2//TMOD1//ADCY3//TMF1//LAMA3//MINK1//SLK//ZNF268//F3//FOXF1//CD274//ITGA6//ITGA5//ITGAV//NTF3//PTP4A1//DLC1//SULF1//TMEFF2//CLIC4//NF2//RAP2C//LDLRAD4//RECK//EPHA7//BCL11B//EPS8//SOX17//PPARG//APCDD1//COL1A2//NKX2-3//MSN//TREM1//TNF//LPAR1//CXCL14//VEGFB//NFIB//FYCO1//SCN1A//GJC1//MPP1//SPRED1//MEOX2//KLF4//DSC2//KCNJ3//NEFH//MCOLN2//CDK6//EREG//MEMO1//LRCH1//PTGER4//MET//STAT3//NSMF//PLAA//AKAP11// |
| GO:0040011 | locomotion | Biological process | 137 | 1845 | 934 | 17653 | 1.40344643489261 | 2.2240338965393e-05 | 0.000599248655071525 | 4.65285859794979 | 0.146680942184154 | SEMA4B//ACVR1//DCX//GJA1//GPM6A//LRP12//NR4A2//SPOCK1//UNK//KDR//SLIT2//CCL2//CCL8//MIA3//HAND2//CD2AP//NCK1//CMKLR1//S1PR1//PIK3CB//MAPK1//FOSL1//CXCL14//RANBP9//ARX//DPYSL2//EFNA1//EFNA5//EPHA5//GAB1//GRB2//KIF5A//OTX2//ENAH//NTN4//SOS1//VLDLR//NR4A3//KLF7//RPS6KA5//FEZ2//EGR2//EPHA4//LHX1//NOG//P2RY1//TGFB2//FOXC1//EDN1//ETS1//BMPR2//WNT7A//NANOS1//DUSP10//PFN2//PTPRG//JUN//TGFBR2//DOCK5//PPM1F//ARID5B//C5ORF30//ADAM10//CRK//IGF1//EPHA2//ERG//RASGEF1A//PALLD//BAMBI//JAK1//RHOC//RND3//PAK6//PTEN//BTG1//ADAM9//POU4F1//LHX6//POU3F2//NRG1//DRD1//EMX2//ADCY3//TMF1//LAMA3//MINK1//SLK//ZNF268//F3//FOXF1//CD274//ITGA6//ITGA5//ITGAV//NTF3//PTP4A1//DLC1//SULF1//TMEFF2//CLIC4//NF2//RAP2C//LDLRAD4//RECK//EPHA7//BCL11B//EPS8//SOX17//PPARG//APCDD1//COL1A2//NKX2-3//MSN//TREM1//TNF//MET//VEGFB//LPAR1//NFIB//MPP1//SPRED1//MEOX2//KLF4//ARRDC3//CHMP3//TSG101//MCOLN2//CDK6//EREG//MEMO1//WASL//LRCH1//PTGER4//STAT3//NSMF//PLAA// |
| GO:2000145 | regulation of cell motility | Biological process | 77 | 904 | 934 | 17653 | 1.60988161111216 | 2.27702317225999e-05 | 0.000611091575952155 | 4.64263254973908 | 0.082441113490364 | MIA3//CCL8//SLIT2//EDN1//ETS1//KDR//BMPR2//WNT7A//DUSP10//PFN2//PTPRG//JUN//TGFB2//TGFBR2//DOCK5//PPM1F//CMKLR1//C5ORF30//ADAM10//CRK//IGF1//NRG1//RHOC//RND3//LAMA3//NCK1//MINK1//SLK//SEMA4B//ZNF268//DRD1//S1PR1//F3//FOXF1//CD274//ITGA6//ITGA5//ITGAV//NTF3//MAPK1//PTP4A1//ACVR1//DLC1//SULF1//TMEFF2//CLIC4//NF2//PTEN//RAP2C//LDLRAD4//RECK//NOG//GAB1//EFNA1//EPHA2//PPARG//ADAM9//VEGFB//LPAR1//TNF//MPP1//SPRED1//MEOX2//KLF4//MSN//WASL//LRCH1//PTGER4//CCL2//CXCL14//MET//STAT3//NSMF//PLAA//CDK6//EREG//MEMO1// |
| GO:0021761 | limbic system development | Biological process | 17 | 102 | 934 | 17653 | 3.15007137758744 | 2.3635072432945e-05 | 0.000631794446102795 | 4.62644306240358 | 0.0182012847965739 | LRP8//CDK6//DRD1//EMX2//NEUROD1//PTEN//BTG2//SLC32A1//EPHA5//EZH1//NF2//NME1//TSC1//NR4A3//ETS1//RAB3GAP1//POU3F2// |
| GO:0007050 | cell cycle arrest | Biological process | 30 | 250 | 934 | 17653 | 2.26805139186296 | 2.41948421938958e-05 | 0.000641685167675754 | 4.61627720614654 | 0.0321199143468951 | CDKN1A//GADD45A//E2F1//CNOT6L//CNOT7//MDM4//CNOT6//RBL2//SOX4//BTG2//NEUROD1//ZNF268//GATA6//PKD2//POU4F1//HMGA2//DUSP1//CDC14A//CDK6//HBP1//MTBP//IRF1//PRKAA1//RRAGD//RHEB//TGFB2//TSC1//TSG101//LAMTOR3//KAT2B// |
| GO:0097485 | neuron projection guidance | Biological process | 30 | 250 | 934 | 17653 | 2.26805139186296 | 2.41948421938958e-05 | 0.000641685167675754 | 4.61627720614654 | 0.0321199143468951 | RANBP9//ARX//DPYSL2//EFNA1//EFNA5//EPHA5//GAB1//GRB2//KIF5A//OTX2//PIK3CB//ENAH//MAPK1//NTN4//SOS1//VLDLR//NR4A3//KLF7//RPS6KA5//SLIT2//FEZ2//EGR2//EPHA4//LHX1//NOG//EPHA7//BMPR2//SEMA4B//BCL11B//NFIB// |
| GO:0042476 | odontogenesis | Biological process | 19 | 123 | 934 | 17653 | 2.91957834995909 | 2.43624842462013e-05 | 0.000643607347488513 | 4.61327842870716 | 0.0203426124197002 | BCL2L11//NKX2-3//FOXC1//SOSTDC1//LRP6//LHX8//NF2//BCL11B//HAND1//HAND2//PAX9//EDN1//FAM20C//ASPN//TFAP2A//ITGA6//COL1A2//OSR1//TGFB2// |
| GO:0048514 | blood vessel morphogenesis | Biological process | 60 | 656 | 934 | 17653 | 1.72869770721262 | 2.47809316846532e-05 | 0.00065211455635529 | 4.60588236955409 | 0.0642398286937901 | S1PR1//EFNA1//EPHA2//ELK3//EREG//FOXC1//GAB1//CLIC4//HOXA3//ITGA5//ITGAV//JUN//KDR//MEOX2//MMP2//TMEM100//PTEN//CCL2//SOX17//TGFB2//VEGFB//WNT7A//HAND1//HAND2//EDN1//PPP3R1//TGFBR2//ACVR1//GJC1//FOXF1//ZFPM2//QKI//SLIT2//SPRED1//SULF1//PPARG//KLF4//SOX4//ETS1//EGLN1//VASH2//F3//GATA6//ITGB8//STAT3//BTG1//ADAM12//HMGA2//PIK3CB//ADIPOR2//PKD2//BMPR2//NOG//TCF21//DLL1//KLF2//EPN1//JAK1//GJA1//SOS1// |
| GO:0048562 | embryonic organ morphogenesis | Biological process | 33 | 288 | 934 | 17653 | 2.16567407209136 | 2.51838503165967e-05 | 0.000655526283444565 | 4.5988778705976 | 0.0353319057815846 | GJA1//DLL1//PKD2//SOX17//TGFBR2//HAND1//HAND2//ACVR1//TFAP2A//EFNA1//LRIG1//OSR1//NEUROG1//SOBP//FZD6//MAPK1//TSHZ1//EDN1//NOG//RARB//FOXF1//SOX11//TCF21//FOXF2//LRP6//ZEB1//RDH10//LHX1//HOXA3//DSCAML1//NR4A3//FRZB//NEUROD1// |
| GO:0030036 | actin cytoskeleton organization | Biological process | 59 | 642 | 934 | 17653 | 1.7369552455856 | 2.5200901389911e-05 | 0.000655526283444565 | 4.59858392502547 | 0.063169164882227 | WASL//KIF23//EPS8//RHOC//RND3//MARCKS//MYO1B//LIMA1//TWF1//CFL2//PALM2//CD2AP//TMOD3//NCK1//TMOD1//ARHGAP12//WASF3//ARHGAP6//ACTC1//PFN2//PLEK//SLIT2//CDC42EP3//GRB2//TNF//FRMD6//CNN3//MYLIP//S1PR1//GAB1//MINK1//DLC1//FZD10//CRK//EFNA5//EPHA5//TSC1//IQGAP2//ARFIP1//PTGER4//LPAR1//PPM1E//NF2//PPM1F//TMEFF2//MET//PPFIA1//EDN1//FRY//NTF3//MTPN//CORO2B//FGD4//FGD5//PALLD//FLII//ATXN3//PAK6//TRIP10// |
| GO:0051726 | regulation of cell cycle | Biological process | 96 | 1197 | 934 | 17653 | 1.51582382079395 | 2.52013653253862e-05 | 0.000655526283444565 | 4.59857592996374 | 0.102783725910064 | E2F1//USP28//CLOCK//CDKN1A//GADD45A//PTEN//CCNE2//CNOT6L//CNOT7//MDM4//CNOT6//RBL2//SOX4//BTG2//CDK6//HBP1//MTBP//IRF1//PKD2//PRKAA1//RRAGD//RHEB//TGFB2//TSC1//TSG101//LAMTOR3//KAT2B//CDC23//NABP1//TAOK3//HMGA2//CDC14A//CDK8//MAP3K2//ZNF268//CDK19//MINK1//PAK6//MAP2K4//SLK//NEDD1//PRKAR2B//YWHAG//HAUS8//CEP41//CHMP3//RAD21//SKP1//HECW2//NEUROG1//RBBP8//KIF13A//CALM2//KIF23//FBXW7//LATS2//IPO5//CCNY//MAPRE3//CCNC//CCND2//RUNX3//BCL2L11//LRP6//NR4A3//FOSL1//EDN1//EREG//IGF1//BTG3//FOXC1//BTG1//TNF//MDM1//DUSP1//PRKACB//RBBP7//RNF2//RRM2//NEUROD1//GATA6//POU4F1//SOX11//RNF4//CEP97//TMOD3//YTHDF2//ZNF655//KLF4//E2F5//JUN//NF2//STAT3//MASTL//PER2//CCNG2// |
| GO:0009968 | negative regulation of signal transduction | Biological process | 101 | 1276 | 934 | 17653 | 1.49603703355642 | 2.6110229384379e-05 | 0.000676565062553851 | 4.58318931278324 | 0.10813704496788 | DUSP10//SPRED1//DUSP1//DUSP2//DUSP5//PPP2CA//RGS3//SOX17//ERRFI1//TSG101//SOCS5//PIAS4//PMEPA1//LDLRAD4//PTEN//KLF4//PLEK//APCDD1//FRZB//SOSTDC1//SHISA2//BAMBI//ASPN//PRDM16//RASL11B//TGFB1I1//TGFBR2//TOB1//CTDSPL2//NOG//PRKAA1//TSC1//FNIP1//ACVR1//DLC1//MET//NPLOC4//SULF1//EPS15//GRB2//EPN1//NF2//ZMYND11//ESR1//EFNA1//HIPK3//AIDA//LPAR1//WWP2//YTHDF2//FBXW7//TAOK3//FLRT1//NEUROD1//TGFB2//NCK1//CD2AP//SLIT2//ARHGAP12//EPHA2//OTUD3//PLEKHA1//PKD2//CALM2//PPARG//CNOT7//TCF21//RANBP9//LMO3//TMEM64//AMER2//LATS2//DKK3//LRP6//FZD6//IGF1//CREBRF//EREG//NRG1//PRKACB//BAG5//WFS1//DNAJB9//PPP1R15B//SESN3//SESN1//LYPD6//CRY2//CLOCK//PHF14//NR4A2//ITGA6//AR//ITGAV//TNF//URI1//EPHA4//EPHA5//EPHA7//RGS17//KDR// |
| GO:0035690 | cellular response to drug | Biological process | 37 | 340 | 934 | 17653 | 2.05681131124827 | 2.62621921131129e-05 | 0.000677905363591536 | 4.58066902604187 | 0.0396145610278373 | CHRM2//CHRM5//GNAQ//KLF2//ETS1//MAP3K5//MYB//PRKAA1//PLEKHA1//BNIP3//KLF4//TNF//GLRA1//UGT1A1//PTEN//SLIT2//DNMT3B//GJB2//ERRFI1//CRK//FMR1//MET//NR4A3//DRD1//MAPK1//ADCY3//EFNA5//KDR//HMGA2//EDN1//PPM1E//PPP1R12A//NME1//RAP1B//REST//PPM1F//CD69// |
| GO:0008406 | gonad development | Biological process | 27 | 215 | 934 | 17653 | 2.37354215427519 | 2.72258750369981e-05 | 0.000700108718156723 | 4.56501815316511 | 0.0289079229122056 | CEBPB//FOXC1//INHBB//DMRTA1//IMMP2L//KMT2B//ESR1//EREG//PLEKHA1//ZFPM2//BCL2L11//RNF38//GATA6//TFAP2C//TGFB2//ARID5B//IDH1//LHX8//AR//TMF1//TCF21//SLIT2//TBC1D20//ARID4B//ARID4A//OSR1//RDH10// |
| GO:0003206 | cardiac chamber morphogenesis | Biological process | 19 | 124 | 934 | 17653 | 2.89603336326587 | 2.73697854185872e-05 | 0.000701143404492065 | 4.56272860748169 | 0.0203426124197002 | ZFPM2//GATA6//RARB//BMPR2//TGFB2//TGFBR2//NOG//SOS1//SOX4//SOX11//FOXF1//HAND1//HAND2//NRG1//POU4F1//ACVR1//FOXC1//FHL2//EGLN1// |
| GO:0045597 | positive regulation of cell differentiation | Biological process | 78 | 926 | 934 | 17653 | 1.59204471351071 | 2.95638351515498e-05 | 0.000754491385395967 | 4.5292392280202 | 0.0835117773019272 | FOXC1//BAMBI//TGFB1I1//TGFB2//TGFBR2//THRA//STMN2//NPTN//NCK1//NME1//SERPINI1//TWF1//ZNF804A//PKDCC//DUSP10//IL2//CEBPB//CMKLR1//CCDC71L//TMEM64//FRZB//PPARG//LMO3//TMEM100//BTG1//MYB//SOCS5//ETS1//STAT3//JUN//MAP3K5//DNMT3B//MMD//NEUROD1//NEUROG1//PTEN//RARB//SOX11//ZEB1//NPNT//GJA1//IGF1//FAM20C//BMPR2//ACVR1//POU4F1//KLF10//TNF//GOLGA4//NRG1//EFNA5//LPAR3//RHEB//MAN2A1//SLIT2//EPHA4//RAB21//CAPRIN2//GATA6//EDN1//LPAR1//CPEB3//FMR1//C21ORF91//LRP8//PRDM16//VLDLR//CRK//CRKL//DOCK5//PLAA//LHX1//KDR//NSMF//ZNF268//AR//NF2//SOX17// |
| GO:0021766 | hippocampus development | Biological process | 14 | 75 | 934 | 17653 | 3.52807994289793 | 3.34248679935979e-05 | 0.000849820985867303 | 4.4759302992422 | 0.0149892933618844 | LRP8//CDK6//DRD1//EMX2//NEUROD1//PTEN//BTG2//SLC32A1//EPHA5//EZH1//NF2//NME1//TSC1//NR4A3// |
| GO:0050821 | protein stabilization | Biological process | 22 | 159 | 934 | 17653 | 2.61515359648768 | 3.37721374619918e-05 | 0.000854519425792816 | 4.4714414516959 | 0.0235546038543897 | CDKN1A//CRTAP//PTGES3//EFNA1//EPHA4//OTUD3//MORC3//GNAQ//IGF1//USP27X//MDM4//PFN2//FBXW7//CDC37L1//PTEN//SOX17//MTMR9//SOX4//TSC1//WFS1//BAG6//CCT6A// |
| GO:0019538 | protein metabolic process | Biological process | 379 | 6078 | 934 | 17653 | 1.17855582636292 | 3.38623696750665e-05 | 0.000854519425792816 | 4.47028265340974 | 0.405781584582441 | CDKN1A//GADD45A//PTEN//CCNE2//RANBP9//PPM1L//SPRED1//DUSP5//EREG//RASGEF1A//GRB2//NRG1//IL2//JAK1//MAP3K5//MET//MINK1//TAOK3//MAPK1//CCL2//SOS1//TNF//LAMTOR3//MAP3K2//CRK//CRKL//ADAM9//LPAR1//LPAR3//IGF1//NTF3//PIK3CB//PRKAA1//MAP2K4//DUSP10//DUSP1//DUSP2//PPP2CA//RGS3//TOPORS//WWP2//HECTD2//RNF145//ZNRF2//FBXL5//RNF180//MYLIP//RLIM//ANKIB1//UBE2W//FBXW7//HECW2//SKP1//THOP1//LONRF3//FBXO30//CBFB//UBE3B//LONRF1//ZFAND4//COPS8//SECISBP2L//DRD1//FNIP1//FAM129A//LRP6//PPP1R15B//SLIT2//EFNA1//EFNA5//EPHA7//FMR1//NPTN//KDR//P2RY1//AKTIP//SQSTM1//CCND2//LYPLA1//RPL15//F3//ERRFI1//TSG101//SOCS5//TRIM3//KLHL2//RNF38//MYCBP2//FBXO33//KLHL3//SPOPL//MDM4//DCAF8//DCAF6//FEM1C//KLHL14//MARCH4//RNF4//MARCH7//UBE2Z//RNF128//KLHL15//KBTBD8//ARRDC4//CPEB2//CPEB3//CPEB1//SOX11//CPEB4//MTPN//DDX6//MKNK2//ACO1//TSC1//PPP4R2//SERP1//UEVLD//RIMKLB//UBE2I//IMMP2L//HIPK3//ADAM10//CDK6//CDK8//FASTK//PDIK1L//ERG//CDK19//MORC3//MMD//PAN3//DSTYK//LATS2//CDK17//PRKACB//FAM20C//CCL8//TGFB2//TGFBR2//RUNX3//CCNC//RPS6KA4//ACVR1//RPS6KA5//STK17B//PPM1E//FLRT1//GNAQ//NCK1//NF2//ASPN//DUS2//YWHAG//CAMK2N2//PPM1F//PTPN21//PPP1R12A//CTDSPL2//PPP3R1//PPP6C//PTPN4//KAT2B//CLOCK//NAA30//HS3ST5//PCMTD1//BTG1//BTG2//ASPH//FUT9//ST8SIA5//MAN1A1//MAN2A1//ST8SIA3//B3GNT5//B3GALT2//CCDC126//GFPT2//EDEM1//VEGFB//NTNG1//RECK//PTAR1//PIGA//MMP2//CTSA//NLN//LRP8//NPEPL1//ADAM12//ADAM18//LMLN//ZFYVE9//USP24//USP27X//ATXN3//NPLOC4//USP28//USP46//BAG6//USP45//CDC23//USP3//DLC1//EGLN3//PMAIP1//PPARG//DLL1//PTGER4//MDFIC//TMF1//ST6GALNAC3//ST6GALNAC6//CALM2//GDF11//INHBB//BMP3//BMPR2//SERPINI1//SPOCK1//PCOLCE2//XYLT2//HS2ST1//PRR16//SOX4//FXR1//GNPTG//ST3GAL3//EPC2//ARID4B//MIER1//ARID4A//RBBP7//TBL1XR1//BRMS1L//ARID5B//DMWD//ESR1//OTUD3//AR//WDR20//JOSD1//NUP35//EGR2//NUP43//PIAS4//TOB1//CNOT7//NANOS1//CAPRIN2//VKORC1L1//CEP41//STK33//MASTL//OSR1//EPHA2//EPHA4//EPHA5//PKDCC//TMX3//ZDHHC1//ST6GAL2//ST3GAL5//MCFD2//CRY2//ZDHHC5//CRTAP//EGLN1//WFS1//PAK6//SLK//SULF1//DSEL//SLC35D1//CHST7//UBXN4//DNAJB9//DERL2//JKAMP//TGFB1I1//JARID2//PER2//BAG5//NDFIP2//PKD2//JUN//RAP2C//EDN1//WNT7A//KLF4//PPP1R37//URI1//VLDLR//OSM//PFN2//IFNA1//HMG20A//ADCY3//PRKAR2B//ESCO2//PRDM16//CNOT6//STAT3//CNEP1R1//NSMF//PTPDC1//PTPRG//PTP4A1//CDC14A//RNF2//BNIP3//HMGA2//PCGF5//DNAJB12//ZNF287//LDLR//ITM2B//ARL6IP1//UBXN2B//CD2AP//PLAA//VPS37A//BCL2L11//REST//ITGAV//FZD10//AIDA//TWF1//CKAP4//RAB38//MIA3//ANO8//TADA1//DR1//RAB12//GATA6//TTR//IRF1//CEBPB//GJA1//NSF//SOX17//IPO5//CCNY//MAPRE3//PAIP2//ZMYND11//ULK2//ITGA5//SH3D19//KLF2//ARRDC3//DPY30//KMT2B//DNMT3B//MYB//RNF141//PMEPA1//LDLRAD4//NOG//CNOT6L//YTHDF2//QKI//ATG16L1//LMO3//NPNT//DNAJC27//HAND2//KDM2A//EZH1//UBA5//ACSL1//GLRX//RAB3GAP1//MSN//FRY//UNK//ST18// |
| GO:0033993 | response to lipid | Biological process | 76 | 900 | 934 | 17653 | 1.5960361646443 | 3.44727690280114e-05 | 0.000866688984893833 | 4.46252383045089 | 0.0813704496788009 | ESR1//FHL2//AR//PMEPA1//RNF4//TGFB1I1//MAPK1//CCL2//TNF//DNMT3B//DUSP1//ETS1//GJB2//PTEN//RBBP8//SLC6A1//STAT3//WNT7A//DUSP10//EDN1//GJA1//TNFRSF21//JUN//UGT1A1//PTGER4//PPARG//RARB//TGFB2//FOSL1//UBA5//CBFB//NME1//ACSL1//PGRMC2//NR5A2//NR3C2//NR4A2//THRA//NR4A3//IDH1//RBBP7//TGFBR2//ADAM9//CDKN1A//SLIT2//TCF21//CEBPB//CDK19//CD274//PLSCR4//PDCD1LG2//PLAA//FZD10//OSR1//MYB//KLF4//PRKAA1//URI1//BCL2L11//REST//H2AFZ//POU4F1//MSN//LRP6//LRP8//E2F1//LDLR//FIBIN//ERRFI1//CREBRF//ADCY3//EFNA5//LPAR1//CRY2//CLOCK//LMO3// |
| GO:0042127 | regulation of cell proliferation | Biological process | 125 | 1668 | 934 | 17653 | 1.41639900071378 | 3.53893738362641e-05 | 0.000886438278720941 | 4.45112712150662 | 0.133832976445396 | SULF1//GJA1//TNF//F3//JUN//KDR//BMPR2//VEGFB//VASH2//FOXF1//TGFBR2//HAND2//ADAM10//ZNF268//CRKL//E2F3//EDN1//EREG//ETS1//CNOT6L//BAMBI//DLL1//CNOT7//NRG1//HOXA3//IGF1//IL2//AR//ITGAV//NTF3//OSM//DERL2//POU3F2//CDCA7L//PRKAA1//MAPK1//SPHK2//PTEN//PTHLH//CNOT6//RARB//SOX4//SOX11//TGFB2//FOSL1//CCND2//TOB1//CDK6//GDF11//CDKN1A//DLC1//TOB2//COPS8//BTG3//FRZB//TES//MTBP//IRF1//MDM4//ASPH//NF2//NME1//PKD2//REST//BCL11B//STAT3//STRN//ZEB1//BTG1//TFAP2A//TGFB1I1//KLF10//TSC1//TSG101//BTG2//KAT2B//KLF4//ST18//LHX1//TNFRSF21//CD274//NCK1//PDCD1LG2//SOS1//CEBPB//MARCH7//LRP6//E2F1//ESR1//MORC3//NR4A3//S1PR1//MYB//OGN//PPARG//OSR1//NOG//DUSP10//RUNX3//JARID2//ZFPM2//GATA6//WNT7A//ERRFI1//PER2//CCL8//HMGA2//SOX17//MEF2D//MAP3K5//MMP2//FBXW7//NFIB//PHF14//TOPORS//EGLN3//NKX2-3//ARX//EPS15//NR5A2//GRB2//JAK1//IER5//TFAP2C//BAG6// |
| GO:0021953 | central nervous system neuron differentiation | Biological process | 23 | 171 | 934 | 17653 | 2.5421628661232 | 3.56812244706866e-05 | 0.000890450631347799 | 4.4475602503795 | 0.0246252676659529 | MNX1//SOX4//LHX1//MTPN//WNT7A//BCL11B//ARX//SLIT2//DRD1//LHX6//GNAQ//LHX8//HPRT1//C21ORF91//NR4A2//BTG2//PTEN//EPHA4//NFIB//NANOS1//LRP6//POU4F1//SPOCK1// |
| GO:0008283 | cell proliferation | Biological process | 150 | 2078 | 934 | 17653 | 1.36432350328619 | 3.70911762760164e-05 | 0.000922233916009922 | 4.4307293936306 | 0.160599571734475 | PIK3CB//BMPR2//SULF1//GJA1//TNF//F3//JUN//KDR//VEGFB//VASH2//FOXF1//TGFBR2//HAND2//ADAM10//ZNF268//CRKL//E2F3//EDN1//EREG//ETS1//CNOT6L//BAMBI//DLL1//CNOT7//NRG1//HOXA3//IGF1//IL2//AR//ITGAV//NTF3//OSM//DERL2//POU3F2//CDCA7L//PRKAA1//MAPK1//SPHK2//PTEN//PTHLH//CNOT6//RARB//SOX4//SOX11//TGFB2//FOSL1//CCND2//TOB1//CDK6//GDF11//CDKN1A//DLC1//TOB2//COPS8//BTG3//FRZB//TES//MTBP//IRF1//MDM4//ASPH//NF2//NME1//PKD2//REST//BCL11B//STAT3//STRN//ZEB1//BTG1//TFAP2A//TGFB1I1//KLF10//TSC1//TSG101//BTG2//KAT2B//KLF4//ST18//ARX//EMX2//WNT7A//TNFRSF21//FZD6//CEBPB//EPHA2//NR4A3//HMGA2//MSN//IFNA1//CD274//NCK1//PDCD1LG2//TOPORS//EGLN3//NKX2-3//EPS15//NR5A2//GRB2//JAK1//IER5//TFAP2C//BAG6//SOS1//MARCH7//LRP6//BTBD10//HPRT1//IMPDH1//E2F1//ESR1//MORC3//S1PR1//MYB//OGN//PPARG//OSR1//NOG//DUSP10//RUNX3//FOXC1//JARID2//ZFPM2//GATA6//ERRFI1//PER2//CCL8//SOX17//MEF2D//MAP3K5//MMP2//FBXW7//NFIB//PHF14//TACC2//POLR3G//ZMYND11//CHRM5//EPS8//ERG//GAB1//APPL1//MET//USP28//RBBP7//GMNC//EVI5//BHLHE41//CDC14A// |
| GO:0010638 | positive regulation of organelle organization | Biological process | 58 | 637 | 934 | 17653 | 1.7209181152283 | 3.84069749191125e-05 | 0.000951451909809369 | 4.41558989818953 | 0.0620985010706638 | CNOT6L//PAN3//CNOT6//CFL2//PLEK//CDC42EP3//GRB2//NCK1//PFN2//MET//SLAIN2//CLIP1//STMN2//NEUROG1//MIER1//MAPK1//CCT6A//FMR1//RPS6KA4//RPS6KA5//IQGAP2//ARFIP1//EDN1//EREG//IGF1//RAD21//WASL//LPAR1//PPM1E//NF2//TSC1//PPM1F//DNMT3B//MYB//JARID2//MSN//TNF//KDR//MIEF1//BNIP3//DDHD1//BCL2L11//PMAIP1//MOAP1//DYNLL2//E2F1//PPP3R1//YWHAG//CALCOCO2//FYCO1//RAB3GAP1//NRG1//FBXW7//ELMOD1//PRKAA1//SNIP1//NTF3//TMED9// |
| GO:0010942 | positive regulation of cell death | Biological process | 61 | 681 | 934 | 17653 | 1.69298990337298 | 3.9302955904006e-05 | 0.000970094491893404 | 4.40557478589623 | 0.0653104925053533 | FNIP1//DLC1//EGLN3//F3//PMAIP1//PPARG//TNF//MAP3K5//PTEN//BNIP3//PIK3CB//MAP2K4//BCL2L11//ZNF268//FGD4//GADD45A//DUSP1//E2F1//LPAR1//CDK19//FRZB//LATS2//ITGA6//JUN//USP27X//NEUROD1//ARHGEF4//POU4F1//RARB//REST//MOAP1//ZMAT3//UBE2Z//SOS1//SOS2//SOX4//FOSL1//HMGA2//NSMAF//SQSTM1//SLIT2//CDKN1A//PPM1F//EPHA7//MYB//TFAP2A//TGFB2//DYNLL2//PPP3R1//YWHAG//PIAS4//SERINC3//NCK1//FBXW7//CD274//E2F3//NR4A3//BTG1//STK17B//INHBB//ST18// |
| GO:0051216 | cartilage development | Biological process | 24 | 184 | 934 | 17653 | 2.46527325202495 | 4.04494935486087e-05 | 0.000994763363160875 | 4.39308691163939 | 0.0256959314775161 | THRA//WNT7A//OSR1//MEF2D//NFIB//HMGA2//RUNX3//SULF1//BMPR2//RARB//TGFBR2//MAF//PTHLH//PKDCC//LRP6//HAND1//HAND2//FRZB//NOG//EDN1//HOXA3//ITGB8//BMP3//ZEB1// |
| GO:0045596 | negative regulation of cell differentiation | Biological process | 61 | 682 | 934 | 17653 | 1.69050751348534 | 4.10185034198289e-05 | 0.0010042903621909 | 4.3870201891253 | 0.0653104925053533 | NME1//SOSTDC1//EFNA1//PPP2CA//PTEN//LDLRAD4//FRZB//ITGAV//PPARG//BHLHE41//STMN2//LPAR1//EPHA4//MYLIP//PTPRG//SPOCK1//TSC1//BAG5//OSR1//DLL1//PTHLH//RARB//SOX17//HMGB3//IRF1//E2F1//ZFPM2//ZADH2//TGFB1I1//TNF//ZEB1//SOCS5//CDK6//CXCL14//HMG20A//DDX6//REST//TOB1//TMEM64//NOG//HAND2//TOB2//EPHA7//ULK2//RGMA//DUSP10//SEMA4B//WNT7A//EREG//SOX11//IGF1//LDLR//SLIT2//EFNA5//TGFB2//IL2//STAT3//FBXW7//NRG1//GDF11//MAPK1// |
| GO:0090304 | nucleic acid metabolic process | Biological process | 337 | 5329 | 934 | 17653 | 1.19524194510824 | 4.11338799832737e-05 | 0.0010042903621909 | 4.38580032341445 | 0.360813704496788 | IGF1//NFIA//NFIB//RBBP7//RBBP8//RRM2//E2F1//NPAT//TSHZ1//HMG20A//KLF2//ZBTB18//CEBPB//ZNF268//TCERG1//WWP2//KLF12//OSR1//CRY2//GLIS3//ARX//DNMT3B//DR1//EDN1//EFNA1//CC2D1B//EN1//ESR1//FHL2//CPEB3//FOXF1//FOXC1//MYT1L//CIC//ZFPM2//DNAJB5//GATA6//CNOT7//HSBP1//AR//JARID2//JUN//ZNF568//MAF//MDM4//MYB//NR4A2//RLIM//KLF3//ZBTB7A//PIAS4//POU4F1//PPARG//CC2D1A//TSHZ3//ZBTB4//RARB//REST//RNF2//PRDM16//SOX17//SOX11//STAT3//ZEB1//TCF21//TFAP2A//TFAP2C//TGIF1//KLF10//TNF//UBE2I//VLDLR//WFS1//ZNF217//LRP8//BTG2//BHLHE41//TBL1XR1//NR4A3//HMGA2//ARID5B//BRMS1L//BHLHE40//RUNX3//URI1//PER2//SQSTM1//NOG//KLF4//HAND1//FNIP1//PHF14//USP3//DCP2//PPP2CA//RPL15//CNOT6L//PAN3//CNOT6//RBFOX3//FMR1//MBNL1//RBM25//SRSF2//ELAVL2//NOVA1//COPS8//PTGES3//CCNE2//NABP1//GTF2E1//DUS2//IRF9//KLF6//CREBRF//EGR2//ELK3//ERG//FOXJ3//FOXF2//FLI1//GRHL1//HIVEP2//IRF1//LHX1//MEF2D//MEOX2//MYBL1//NEUROD1//NHLH2//OTX2//PAX9//PBX3//POU3F2//RAD21//ARID4A//SHOX//BCL11B//BMPR2//SOX4//BTF3//TEF//TGFB1I1//THRA//TMF1//DEK//FOSL1//KLF7//CBFB//FUBP3//HAND2//CLOCK//POLR3G//PKD2//NME1//REV3L//GMNC//ESCO2//GADD45A//EPC2//TAOK3//UBE2W//USP28//USP45//ATXN3//KDM2A//MTRR//HMGB3//BAHD1//H2AFZ//KMT2B//DPY30//TOPORS//KHDRBS3//ZMYND11//TADA1//ZNF597//CTNND2//ZNF782//NKX2-3//ZNF800//E2F5//ZNF367//EZH1//ZBTB9//MYCBP2//FLII//LHX6//HBP1//ATAD2//SERTAD3//MDFIC//MNX1//HOXA3//HOXD1//FOXN2//ZBTB41//ZBTB34//LHX8//NEUROG1//NFE2//NPAS2//IER5//ARID4B//BRWD1//BNC2//ZNF532//CDCA7L//PRKAA1//TRERF1//LMO3//MAPK1//SLC2A4RG//PHTF2//ZNF630//ZNF287//ZNF512B//ZBTB26//MIER1//RBL2//RNF4//ATXN1//DMRTA1//SMARCD2//STAT4//ZNF2//ZNF3//ZNF131//ZNF655//BRD3//TCF7L1//PCGF5//LCOR//WASL//ZBTB47//ST18//HIPK3//NUP35//NR5A2//NRBF2//MLLT6//RNF141//PAK6//SNAPC1//BTG1//SNIP1//RPS6KA4//RPS6KA5//CDKN1A//CRK//EMX2//ETS1//BOLA3//NR3C2//PLEK//ZNFX1//NKX2-4//RFX7//BDP1//CDK8//E2F3//TEAD1//KAT2B//CCNC//CMTR2//ZFC3H1//RTCA//PPP4R2//ESRP1//CPEB1//QKI//G3BP2//NF2//EREG//DDX6//INTS8//RBM47//MAP3K2//MAPRE3//BAMBI//RGMB//LRP6//MAP3K5//WNT7A//ACVR1//DKK3//NRG1//RASD1//TSG101//BTAF1//PPM1F//TOB1//SOSTDC1//TMEM100//RGMA//BMP3//TGFB2//SULF1//CTDSPL2//CMKLR1//CCT6A//EPHA5//FBXW7//DCTPP1//TDRD5//EGLN1//FZD6//FASTK//AHNAK//YTHDF2//FXR1//NPLOC4//MAP2K4//S1PR1//DLL1//IL2//ITGA6//MET//PPP1R12A//NCK1//OSM//P2RY1//PPP3R1//DCAF6//CAPRIN2//PTEN//MTPN//EGLN3//REXO1//ZC3H12C//NANOS1//RAP2C//TOB2//KLHL15//DUSP1//GJA1//NPNT// |
| GO:0033673 | negative regulation of kinase activity | Biological process | 32 | 283 | 934 | 17653 | 2.13715089928194 | 4.31481453342973e-05 | 0.0010496795212081 | 4.3650378671189 | 0.0342612419700214 | DUSP10//SPRED1//DUSP1//DUSP2//DUSP5//PPP2CA//RGS3//ERRFI1//TSG101//SOCS5//GADD45A//PPM1E//FLRT1//GNAQ//LRP6//NCK1//NF2//TAOK3//ASPN//DUS2//YWHAG//CAMK2N2//PPM1F//CDK6//PTEN//HIPK3//AIDA//CDKN1A//LATS2//IPO5//KAT2B//PRKAR2B// |
| GO:0007409 | axonogenesis | Biological process | 45 | 456 | 934 | 17653 | 1.86517384199256 | 4.33929310721983e-05 | 0.00105185087039884 | 4.3625810134917 | 0.0481798715203426 | RANBP9//ARX//DPYSL2//EFNA1//EFNA5//EPHA5//GAB1//GRB2//KIF5A//OTX2//PIK3CB//ENAH//MAPK1//NTN4//SOS1//VLDLR//NR4A3//KLF7//RPS6KA5//SLIT2//FEZ2//EGR2//EPHA4//LHX1//NOG//NR4A2//PTEN//NFIB//RAB21//WNT7A//EPHA7//GOLGA4//NRG1//ULK2//LPAR3//BMPR2//SEMA4B//NEFH//POU3F2//BCL11B//NTNG1//SLITRK3//OGN//POU4F1//DSCAML1// |
| GO:0061061 | muscle structure development | Biological process | 58 | 640 | 934 | 17653 | 1.71285131156317 | 4.38509148374018e-05 | 0.00105558674994663 | 4.35802134175767 | 0.0620985010706638 | ZFPM2//NRG1//TGFB2//POU4F1//NOG//S1PR1//IGF1//MEF2D//FXR1//SGCE//ZBTB18//CFL2//MEOX2//ADAM12//THRA//BHLHE41//TCF21//MTPN//TMOD3//TMOD1//ACTC1//EGR2//FOXN2//SOX11//BTG2//QKI//MBNL1//SOSTDC1//DLL1//TNF//CXCL14//MAP3K5//BTG1//GJA1//NEUROG1//PRKAA1//SOX17//SERP1//ARID5B//FOXF1//GATA6//ACVR1//ZEB1//EREG//TSC1//EGLN1//FOXC1//HAND1//RARB//FHL2//JARID2//PTEN//TGFBR2//EDN1//REST//MAP2K4//NLN//NPNT// |
| GO:0043408 | regulation of MAPK cascade | Biological process | 66 | 757 | 934 | 17653 | 1.64785768233108 | 4.38592158413429e-05 | 0.00105558674994663 | 4.35793913733752 | 0.0706638115631692 | GADD45A//MINK1//TNF//MAP3K2//CRK//CRKL//MAP3K5//TAOK3//LAMTOR3//ADAM9//DUSP5//LPAR1//LPAR3//NRG1//IGF1//NTF3//PIK3CB//PRKAA1//MAPK1//MAP2K4//DUSP10//SPRED1//DUSP1//DUSP2//PPP2CA//RGS3//MDFIC//TGFB2//TSG101//EDN1//EFNA1//NF2//EPHA2//EPHA4//EPHA5//EPHA7//AR//ITGAV//KDR//MET//OSM//FZD10//HIPK3//AIDA//ZMYND11//WNT7A//RANBP9//ERRFI1//LMO3//PTEN//KLF4//NPNT//DSTYK//NPTN//JUN//P2RY1//DNAJC27//FBXW7//CCL2//CCL8//HAND2//GDF11//GRB2//INHBB//PAK6//BMP3// |
| GO:0014070 | response to organic cyclic compound | Biological process | 78 | 937 | 934 | 17653 | 1.57335475422724 | 4.41431160358451e-05 | 0.00105865210549794 | 4.35513701350766 | 0.0835117773019272 | DRD1//SNIP1//SLC6A1//ESR1//FHL2//AR//PMEPA1//RNF4//TGFB1I1//DNMT3B//PPARG//PRKAA1//DUSP1//ETS1//GJB2//PTEN//RBBP8//STAT3//WNT7A//TGFB2//FOSL1//UBA5//CBFB//SRSF2//TRPC3//EDN1//NME1//IFNA1//MAPK1//PMAIP1//PGRMC2//NR5A2//NR3C2//NR4A2//RARB//THRA//NR4A3//IDH1//RBBP7//TGFBR2//TNF//ADAM9//CDKN1A//SLIT2//JUN//TCF21//PKD2//RAP1B//HCN2//URI1//BCL2L11//UGT1A1//REST//H2AFZ//POU4F1//MSN//LRP6//LRP8//CEBPB//FOXF1//ITGA6//P2RY1//CCL2//TMF1//KLF2//KLF4//FIBIN//ERRFI1//PRKACB//PRKAR2B//CREBRF//ADCY3//EFNA5//CRY2//CLOCK//LMO3//ACSL1//BTG2// |
| GO:0051093 | negative regulation of developmental process | Biological process | 80 | 968 | 934 | 17653 | 1.56201886491939 | 4.50714207032086e-05 | 0.00107709547072721 | 4.34609875224987 | 0.0856531049250535 | NME1//BMPR2//SOX17//SOSTDC1//BNIP3//EFNA1//PPP2CA//PTEN//LDLRAD4//FRZB//ITGAV//PPARG//BHLHE41//STMN2//LPAR1//EPHA4//MYLIP//PTPRG//SPOCK1//TSC1//BAG5//SPRED1//FOXC1//SULF1//TGFB2//KLF4//OSR1//DLL1//TNFRSF21//PTHLH//RARB//ADRB1//HMGB3//IRF1//CDK6//GDF11//MAPK1//E2F1//ZFPM2//ZADH2//TGFB1I1//TNF//ZEB1//SOCS5//CXCL14//HMG20A//DDX6//REST//TOB1//TMEM64//NOG//HAND2//TOB2//EPHA7//ULK2//RGMA//DUSP10//SEMA4B//WNT7A//EREG//GJA1//JARID2//TGFBR2//SOX11//IGF1//LDLR//ASPN//MEOX2//SLIT2//EFNA5//PRKACB//KLF2//EPN1//IL2//FBXW7//STAT3//HMGA2//NFIB//PHF14//NRG1// |
| GO:0032970 | regulation of actin filament-based process | Biological process | 39 | 377 | 934 | 17653 | 1.95521671712324 | 5.00881960321305e-05 | 0.00119276925973697 | 4.30026460950433 | 0.0417558886509636 | EPS8//TWF1//PFN2//LIMA1//CFL2//PLEK//SLIT2//CDC42EP3//GRB2//NCK1//DLC1//FZD10//CRK//EFNA5//EPHA5//RHOC//RND3//TSC1//IQGAP2//ARFIP1//WASL//PTGER4//LPAR1//PPM1E//NF2//PPM1F//S1PR1//TMEFF2//ARHGAP6//MET//PPFIA1//TMOD3//TMOD1//EDN1//DSC2//CD2AP//NTF3//MTPN//FRMD6// |
| GO:0042063 | gliogenesis | Biological process | 30 | 260 | 934 | 17653 | 2.18081864602207 | 5.08862003519085e-05 | 0.00120752060694722 | 4.29339997647601 | 0.0321199143468951 | EPHA4//P2RY1//TGFB2//NFIB//CDK6//DRD1//DLL1//POU3F2//WASF3//LPAR1//SOX11//SOX4//NF2//EGR2//NRG1//KCNJ10//PPP3R1//PTEN//APCDD1//CCL2//STAT3//DUSP10//BNIP3//NOG//TNFRSF21//PPARG//RHEB//MAPK1//MYB//LDLR// |
| GO:0048545 | response to steroid hormone | Biological process | 40 | 391 | 934 | 17653 | 1.93354764864702 | 5.16953337694678e-05 | 0.00122243196602416 | 4.28654865631862 | 0.0428265524625268 | ESR1//FHL2//AR//PMEPA1//RNF4//TGFB1I1//GJB2//TGFB2//FOSL1//UBA5//CBFB//PGRMC2//NR5A2//NR3C2//NR4A2//PPARG//RARB//THRA//NR4A3//DUSP1//TNF//ADAM9//CDKN1A//SLIT2//TCF21//URI1//BCL2L11//EDN1//UGT1A1//REST//FIBIN//DNMT3B//ERRFI1//CREBRF//CRY2//CLOCK//LMO3//IDH1//RBBP7//TGFBR2// |
| GO:0044087 | regulation of cellular component biogenesis | Biological process | 74 | 881 | 934 | 17653 | 1.58755016078186 | 5.20573456513209e-05 | 0.00122670323567904 | 4.2835179798759 | 0.0792291220556745 | EPS8//TWF1//EPHA2//SLIT2//CNOT6L//PAN3//CNOT6//PMEPA1//LDLRAD4//THRA//PFN2//CDC42EP3//GRB2//NCK1//STMN2//MET//SLAIN2//CLIP1//TNF//FNIP1//PLEK//PMAIP1//BCL2L11//RHOC//PTGER4//IQGAP2//ARFIP1//CEP97//ESR1//PRKAA1//MDM1//KAT2B//WASL//FMR1//GPM6A//TSC1//LPAR1//PPM1E//NF2//PPM1F//DLC1//S1PR1//TMEFF2//ARHGAP6//PPFIA1//TMOD3//TMOD1//EFNA5//SLK//KDR//PTEN//EPHA7//SLITRK3//FLRT1//WNT7A//EDN1//MSN//BAG5//SACS//RNF4//ORMDL3//RAP1B//FEZ2//TSG101//JUN//FOSL1//NSMAF//REST//PIKFYVE//TBC1D12//RAB3GAP1//GJA1//CBFB//MTPN// |
| GO:0016477 | cell migration | Biological process | 112 | 1474 | 934 | 17653 | 1.43612480714977 | 5.33831633710301e-05 | 0.00125357754818846 | 4.27259569443243 | 0.119914346895075 | SEMA4B//ACVR1//DCX//GJA1//GPM6A//LRP12//NR4A2//SPOCK1//UNK//KDR//SLIT2//CCL2//CCL8//MIA3//HAND2//CD2AP//NCK1//EPHA4//P2RY1//TGFB2//FOXC1//EDN1//ETS1//BMPR2//WNT7A//NANOS1//DUSP10//PFN2//PTPRG//JUN//TGFBR2//DOCK5//PPM1F//CMKLR1//ARID5B//C5ORF30//ADAM10//CRK//IGF1//POU4F1//LHX6//POU3F2//ARX//NRG1//DRD1//EMX2//RHOC//RND3//LAMA3//MINK1//SLK//ZNF268//S1PR1//F3//FOXF1//CD274//ITGA6//ITGA5//ITGAV//NTF3//MAPK1//PTP4A1//DLC1//SULF1//TMEFF2//CLIC4//NF2//PTEN//RAP2C//LDLRAD4//RECK//NOG//BCL11B//EPS8//GAB1//SOX17//EFNA1//EPHA2//PPARG//APCDD1//COL1A2//NKX2-3//GRB2//MSN//PIK3CB//TREM1//SOS1//TNF//ADAM9//LPAR1//CXCL14//VEGFB//MPP1//SPRED1//MEOX2//KLF4//LHX1//MCOLN2//WASL//LRCH1//PTGER4//MET//STAT3//NSMF//PLAA//ERG//RASGEF1A//PALLD//BAMBI//JAK1//PAK6//BTG1// |
| GO:0040012 | regulation of locomotion | Biological process | 80 | 973 | 934 | 17653 | 1.5539920464974 | 5.36971274389502e-05 | 0.00125658710335509 | 4.27004894653101 | 0.0856531049250535 | MIA3//CCL8//SLIT2//EDN1//ETS1//KDR//BMPR2//WNT7A//DUSP10//PFN2//PTPRG//JUN//TGFB2//TGFBR2//DOCK5//PPM1F//CMKLR1//C5ORF30//ADAM10//CRK//IGF1//NRG1//RHOC//RND3//LAMA3//NCK1//MINK1//SLK//SEMA4B//ZNF268//DRD1//S1PR1//F3//FOXF1//CD274//ITGA6//ITGA5//ITGAV//NTF3//MAPK1//PTP4A1//ACVR1//DLC1//SULF1//TMEFF2//CLIC4//NF2//PTEN//RAP2C//LDLRAD4//RECK//NOG//GAB1//EFNA1//EPHA2//PPARG//VEGFB//ADAM9//LPAR1//TNF//MPP1//SPRED1//MEOX2//KLF4//ARRDC3//CHMP3//TSG101//CDK6//EREG//MEMO1//MSN//WASL//LRCH1//PTGER4//CCL2//CXCL14//MET//STAT3//NSMF//PLAA// |
| GO:0043200 | response to amino acid | Biological process | 17 | 109 | 934 | 17653 | 2.94777321572402 | 5.67789633818453e-05 | 0.00132412458397041 | 4.24581254068748 | 0.0182012847965739 | CEBPB//COL1A2//SESN3//CPEB3//NSMF//SESN1//GLRA1//IPO5//MMP2//RRAGD//CPEB1//ZEB1//TNF//CPEB4//LAMTOR3//EDN1//GLRB// |
| GO:0021954 | central nervous system neuron development | Biological process | 13 | 69 | 934 | 17653 | 3.56095025292493 | 5.72897560892307e-05 | 0.00133144543103597 | 4.24192302675242 | 0.0139186295503212 | ARX//SLIT2//DRD1//LHX6//GNAQ//LHX8//NR4A2//PTEN//EPHA4//NFIB//NANOS1//HPRT1//BTG2// |
| GO:0051179 | localization | Biological process | 400 | 6497 | 934 | 17653 | 1.16364034265197 | 5.82585566970149e-05 | 0.0013493240374723 | 4.23464027882758 | 0.428265524625268 | RANBP6//IPO5//TNF//DRD1//STAT3//SNAP25//SLC17A7//CHRM5//SEMA4B//ACVR1//DCX//GJA1//GPM6A//LRP12//NR4A2//SPOCK1//UNK//EDN1//BCL2L11//PMAIP1//MOAP1//ARL6IP1//KDR//SLIT2//FMR1//NTF3//STIM2//CCL2//CCL8//LHFPL2//IGF1//NHLRC2//PLEK//TMX3//CDC37L1//TGFB2//VEGFB//MIA3//HAND2//SLC6A14//SLC32A1//SLC6A6//PKD2//THRA//TWF1//RTN3//ARAP2//RAB38//NECAP1//APPL1//GOLGA4//AGFG1//AGFG2//KIF5A//NSF//HSPA13//AP1S2//GIT2//CEP128//MAPRE3//SQSTM1//STX6//SYT6//STX2//ZFAND6//SRSF2//NUP35//NUP43//TSC1//PAN3//YWHAG//EGR2//ZDHHC1//RPL15//MTX3//IMMP2L//SLC6A1//SLC24A2//GLRA1//GLRB//TMEM63B//SLC17A6//SLC24A3//SLC12A5//SLC20A1//KCNA4//KCNJ3//KCNJ10//SCN1A//SLC4A4//TRPV6//TRPC3//CLCN6//CLIC4//ANO8//NDFIP2//ARMC1//SLC14A1//SLC25A32//MICU3//MID1IP1//WWP2//TSG101//VLDLR//APOO//SEC23A//RAB12//RAB21//TBC1D12//SNX5//ARFIP1//RAB30//SAR1B//RAB9B//DNAJC27//RAB23//TMED9//CTSA//RAB22A//KIF13A//SNX2//EVI5//TBC1D17//RAB28//ZFYVE9//USP6NL//EXOC5//CNIH1//DCTN6//TBC1D20//DYNLL2//DYNC1LI2//TRAPPC8//GRIA1//IER3IP1//MCFD2//ERGIC2//KIF23//GOLGA3//MYO1B//OSBPL5//STEAP2//EPS15//DENND1B//DPYSL2//EPN1//LDLR//NME1//LRP8//WASL//TRIP10//LMBR1L//TGFBR2//ARHGAP12//NEUROD1//ANP32E//CD2AP//NCK1//CDC23//G3BP2//WNT7A//PPFIA1//RAPGEF4//MARCKS//PER2//CLOCK//FXR1//AKTIP//FAM160A2//EPHA4//P2RY1//FOXC1//SLC35D1//CBLN4//ETS1//BMPR2//NANOS1//DUSP10//PFN2//PTPRG//JUN//DOCK5//PPM1F//CMKLR1//ARID5B//C5ORF30//ADAM10//NR4A3//CALM2//FBXW7//PPARG//ITGAV//CRK//TRIM3//VPS37A//LCA5//SERP1//CHMP3//ATG16L1//UEVLD//ANKRD50//ZMAT3//BTF3//PLEKHF2//PKDCC//ARRDC4//CEP41//SLC41A1//MMGT1//SLC16A6//SERINC3//SLC44A1//ADIPOR2//ARHGAP1//DPY30//GNPTG//S1PR1//EFNA1//EPHA2//ERG//RASGEF1A//PALLD//BAMBI//GRB2//JAK1//RHOC//RND3//PIK3CB//PAK6//PTEN//BTG1//ADAM9//PEX5L//INHBB//SOX11//SYBU//POU4F1//LHX6//POU3F2//ARX//NRG1//EMX2//RUFY2//ADCY3//TMF1//LAMA3//MINK1//SLK//ZNF268//F3//FOXF1//CD274//ITGA6//ITGA5//MAPK1//PTP4A1//DLC1//SULF1//TMEFF2//NF2//RAP2C//LDLRAD4//RECK//NOG//DERL2//NPLOC4//SOX4//MMD//CDKN1A//MZT1//GJC1//CLCN5//OSTM1//ASPH//MTBP//TOPORS//PIKFYVE//CNEP1R1//FRMD6//LATS2//RRAGD//LAMTOR3//KCNK10//HCN2//BCL11B//LHX1//MCOLN2//EPS8//GAB1//SOX17//TMEM87A//ZFYVE21//MDFIC//LYPLA1//C16ORF70//PXK//IQGAP2//CKAP4//ENPP4//GNS//IDH1//IMPDH1//C6ORF120//RAP1B//TTR//ORMDL3//APCDD1//XKR8//SLC2A4//ACSL1//CLIP1//DZIP1//NPNT//PLEKHA1//DLL1//REST//PPP1R12A//CTDSPL2//OSM//BNIP3//PPP6C//FCHO2//PTGER4//ACO1//COL1A2//NKX2-3//MSN//TREM1//SOS1//IL2//QKI//ATXN1//CENPQ//MORC3//SKP1//LPAR3//WFS1//GJB2//SLC46A3//LPAR1//CXCL14//PRKAA1//EFNA5//EPHA5//RNF128//RAB3GAP1//KLHL3//DENND5B//ESCO2//ESR1//RAD21//NEDD1//BAG6//FYCO1//EFR3A//LRP6//MPP1//SPRED1//MEOX2//KLF4//MIEF1//PRKAR2B//CEP97//HAUS8//FAM155A//DSC2//E2F1//PPP3R1//AHNAK//GNB5//NEFH//COX7A2//COX8C//COX7A2L//NPTN//AR//ARL5A//PLAA//CCNE2//ELMOD1//SNIP1//EDEM1//ATXN3//CCT6A//DCP2//SESTD1//OSR1//NEUROG1//CRY2//CDK6//EREG//MEMO1//LRCH1//HECW2//GLRX//TNFRSF21//MYB//MET//NSMF//PIK3R3// |
| GO:0021537 | telencephalon development | Biological process | 28 | 237 | 934 | 17653 | 2.23296198917591 | 5.90297617803579e-05 | 0.00136251972327836 | 4.22892896940772 | 0.0299785867237687 | LRP8//CDK6//DRD1//EMX2//NEUROD1//PTEN//BTG2//HPRT1//RARB//SLC32A1//EPHA5//EZH1//NF2//NME1//TSC1//NR4A3//BCL11B//LHX6//POU3F2//ARX//SLIT2//NRG1//NFIB//TACC2//LRP6//BNIP3//LPAR1//LHX1// |
| GO:0007492 | endoderm development | Biological process | 14 | 79 | 934 | 17653 | 3.34944298376386 | 6.09281514357151e-05 | 0.00140155472163177 | 4.21518199819995 | 0.0149892933618844 | DUSP1//DUSP2//DUSP5//LHX1//SOX17//NOG//GATA6//HSBP1//ITGA5//ITGAV//LAMA3//MMP2//HMGA2//PAX9// |
| GO:0033674 | positive regulation of kinase activity | Biological process | 54 | 590 | 934 | 17653 | 1.72986970565819 | 6.16488624023722e-05 | 0.00141332629297371 | 4.21007493296818 | 0.0578158458244111 | GADD45A//MINK1//TNF//MAP3K2//CRK//CRKL//MAP3K5//TAOK3//LAMTOR3//ADAM9//DUSP5//LPAR1//LPAR3//NRG1//IGF1//NTF3//PIK3CB//PRKAA1//MAPK1//MAP2K4//EREG//FBXW7//EFNA5//COPS8//MDFIC//PKD2//PAK6//TGFB2//TGFBR2//SLK//ADCY3//PRKACB//PRKAR2B//EDN1//FZD10//EPHA4//CCNY//MAPRE3//CCNC//CCND2//CDKN1A//MMD//VLDLR//EFNA1//LRP8//ACSL1//PPP2CA//CALM2//EPHA2//EPHA5//EPHA7//DSTYK//KDR//MET// |
| GO:0008219 | cell death | Biological process | 157 | 2216 | 934 | 17653 | 1.33906463794556 | 6.22617891408622e-05 | 0.00142255567553936 | 4.20577840368499 | 0.168094218415418 | BCL2L11//TNFRSF21//JUN//PMAIP1//MOAP1//PTEN//IL2//FNIP1//HAND2//HIPK3//RTN3//DLC1//EGLN3//GADD45A//EPHA7//ARL6IP1//SULF1//GJA1//IRF1//C6ORF120//MEF2D//CHMP3//ZFAND6//PPP2CA//MAPK1//PAK6//RAD21//MAP2K4//AKTIP//UBE2Z//BNIP3//TGFBR2//BAG6//FXR1//SQSTM1//STK17B//SLK//F3//PPARG//TNF//TOPORS//CDKN1A//ZNF268//OSR1//DUSP1//EPHA2//EPHA4//EPHA5//FHL2//DSTYK//GATA6//IGF1//KDR//MDM4//MET//PRKAA1//SPHK2//RARB//BCL11B//STAT3//ACTC1//TFAP2A//TMF1//WNT7A//HMGA2//CCND2//E2F1//MAP3K5//GDF11//MAP3K2//ESR1//RASSF3//INHBB//NF2//NME1//NTF3//MINK1//TAOK3//RBM25//BMP3//TGFB2//ANP32E//PIK3CB//THRA//DLL1//CCL2//EDN1//LRP6//USP28//FGD4//LPAR1//CDK19//FRZB//LATS2//ITGA6//USP27X//NEUROD1//ARHGEF4//POU4F1//REST//ZMAT3//SOS1//SOS2//SOX4//FOSL1//NSMAF//SLIT2//WFS1//KLF4//PPM1F//NSMF//CEBPB//EN1//NR4A2//BTG2//NR4A3//CPEB4//MYB//ZFPM2//NCK1//NPAS2//DUS2//SOX11//EFNA1//DSC2//KRT27//XKR8//MEOX2//BAG5//BMPR2//FASTK//SGPP1//APPL1//MKNK2//ITGAV//ITM2B//DYNLL2//PPP3R1//YWHAG//EGLN1//TSC1//BTBD10//PIAS4//SERINC3//FOXC1//FBXW7//CD274//E2F3//IER3IP1//BTG1//ITGA5//NOG//ZMYND11//AR//ACVR1//URI1//ST18// |
| GO:0007613 | memory | Biological process | 17 | 110 | 934 | 17653 | 2.92097527739926 | 6.39062341081218e-05 | 0.00145521165411861 | 4.19445677397468 | 0.0182012847965739 | CPEB3//GRIA1//LDLR//SLC17A7//CCND2//CEBPB//DRD1//CIC//SLC24A2//ITGA5//NTF3//PAIP2//KCNK10//PTEN//ATXN1//VLDLR//KMT2B// |
| GO:0051493 | regulation of cytoskeleton organization | Biological process | 48 | 506 | 934 | 17653 | 1.79292600147269 | 6.57879936303277e-05 | 0.00149303423128156 | 4.18185335829903 | 0.0513918629550321 | EPS8//TWF1//STMN2//MID1IP1//CHMP3//PFN2//LIMA1//CFL2//PLEK//SLIT2//CDC42EP3//GRB2//NCK1//MET//SLAIN2//CLIP1//DLC1//FZD10//CRK//EFNA5//EPHA5//RHOC//RND3//TSC1//IQGAP2//ARFIP1//MDM1//KAT2B//WASL//PTGER4//LPAR1//PPM1E//NF2//PPM1F//S1PR1//TMEFF2//ARHGAP6//PPFIA1//TMOD3//TMOD1//EDN1//RNF4//CEP97//CD2AP//NTF3//MTPN//CLIC4//MAPK1// |
| GO:0001101 | response to acid chemical | Biological process | 35 | 329 | 934 | 17653 | 2.01068385803453 | 6.84537912873465e-05 | 0.00154615455822263 | 4.16460249362821 | 0.0374732334047109 | EDN1//CEBPB//COL1A2//SESN3//CPEB3//NSMF//SESN1//GLRA1//IPO5//MMP2//RRAGD//CPEB1//ZEB1//TNF//CPEB4//LAMTOR3//DUSP1//GJA1//GJB2//PPARG//RARB//ACSL1//GLRB//FZD10//OSR1//MYB//KLF4//PRKAA1//PTGER4//E2F1//LDLR//NME1//PKD2//KLF2//KDR// |
| GO:0097503 | sialylation | Biological process | 7 | 21 | 934 | 17653 | 6.30014275517487 | 6.85858890236268e-05 | 0.00154615455822263 | 4.16376522758123 | 0.00749464668094218 | ST8SIA3//ST6GALNAC3//ST8SIA5//ST6GALNAC6//ST3GAL3//ST6GAL2//ST3GAL5// |
| GO:0006470 | protein dephosphorylation | Biological process | 33 | 303 | 934 | 17653 | 2.05846248436407 | 6.93925332330772e-05 | 0.00155914186795781 | 4.15868725793319 | 0.0353319057815846 | PPM1F//CRY2//PPP1R37//MASTL//URI1//CALM2//PPP1R15B//DLC1//CNEP1R1//NSMF//PTPN21//PTPDC1//DUSP1//DUSP2//DUSP5//PTEN//PTPN4//PTPRG//PTP4A1//CDC14A//PPP1R12A//TNF//DUSP10//PPM1E//PPP2CA//UBXN2B//PPP4R2//TSC1//NCK1//PPM1L//CTDSPL2//PPP3R1//PPP6C// |
| GO:0030029 | actin filament-based process | Biological process | 64 | 739 | 934 | 17653 | 1.63684358456506 | 6.96666664230291e-05 | 0.00155916861998631 | 4.15697497012742 | 0.0685224839400428 | WASL//KIF23//FRMD6//EPS8//RHOC//RND3//MARCKS//MYO1B//LIMA1//TWF1//FLII//CFL2//CORO2B//DLC1//FGD4//FGD5//PALLD//ATXN3//NF2//PFN2//PAK6//TRIP10//PALM2//CD2AP//TMOD3//NCK1//TMOD1//ARHGAP12//ACTC1//WASF3//ARHGAP6//PLEK//SLIT2//CDC42EP3//GRB2//TNF//CNN3//MYLIP//S1PR1//GAB1//MINK1//FZD10//CRK//EFNA5//EPHA5//TSC1//IQGAP2//ARFIP1//PTGER4//LPAR1//PPM1E//PPM1F//TMEFF2//MET//PPFIA1//EDN1//SCN1A//GJC1//GJA1//FRY//DSC2//KCNJ3//NTF3//MTPN// |
| GO:0012501 | programmed cell death | Biological process | 149 | 2088 | 934 | 17653 | 1.3487374576452 | 6.99558690391676e-05 | 0.00155916861998631 | 4.15517584393984 | 0.159528907922912 | BCL2L11//TNFRSF21//JUN//PMAIP1//MOAP1//PTEN//IL2//FNIP1//HAND2//HIPK3//RTN3//DLC1//EGLN3//GADD45A//EPHA7//ARL6IP1//SULF1//GJA1//IRF1//C6ORF120//MEF2D//CHMP3//ZFAND6//PPP2CA//MAPK1//PAK6//RAD21//MAP2K4//AKTIP//UBE2Z//BNIP3//TGFBR2//BAG6//FXR1//SQSTM1//STK17B//SLK//F3//PPARG//TNF//TOPORS//CDKN1A//ZNF268//OSR1//DUSP1//EPHA2//EPHA4//EPHA5//FHL2//DSTYK//GATA6//IGF1//KDR//MDM4//MET//PRKAA1//SPHK2//RARB//BCL11B//STAT3//ACTC1//TFAP2A//TMF1//WNT7A//HMGA2//CCND2//E2F1//MAP3K5//GDF11//MAP3K2//ESR1//RASSF3//INHBB//NF2//NME1//NTF3//MINK1//TAOK3//RBM25//BMP3//TGFB2//ANP32E//PIK3CB//THRA//DLL1//CCL2//EDN1//LRP6//USP28//FGD4//LPAR1//CDK19//FRZB//LATS2//ITGA6//USP27X//NEUROD1//ARHGEF4//POU4F1//REST//ZMAT3//SOS1//SOS2//SOX4//FOSL1//NSMAF//SLIT2//WFS1//KLF4//PPM1F//NSMF//CEBPB//EN1//NR4A2//BTG2//NR4A3//CPEB4//MYB//EFNA1//DSC2//KRT27//XKR8//BMPR2//FASTK//SGPP1//APPL1//MKNK2//ITGAV//ITM2B//DYNLL2//PPP3R1//YWHAG//PIAS4//BAG5//SERINC3//NCK1//FOXC1//FBXW7//CD274//E2F3//IER3IP1//BTG1//ITGA5//NOG//ZMYND11//AR//ACVR1//URI1//ST18// |
| GO:0051345 | positive regulation of hydrolase activity | Biological process | 65 | 754 | 934 | 17653 | 1.62934726426936 | 7.0085355681774e-05 | 0.00155916861998631 | 4.15437271828163 | 0.069593147751606 | DLC1//EGLN3//F3//PMAIP1//PPARG//TNF//LPAR1//GNAQ//NPNT//ESR1//MTMR9//PLEK//PCOLCE2//PLAA//CALM2//PPP1R15B//DNAJB4//PFN2//FAM13A//CDC42EP3//GNB5//FZD10//ARAP2//TAGAP//TBC1D20//ADRB1//DENND1B//S1PR1//RAB3GAP1//DOCK9//RGS17//AGFG1//AGFG2//ITGA6//JUN//ARHGAP1//ARHGAP6//ERRFI1//ELMOD1//RGS3//CCL2//CCL8//SOS1//EVI5//TRIP10//ARHGAP12//USP6NL//GIT2//PPP1R12A//BCL2L11//MAP3K5//REST//PPM1F//RHOC//EPHA2//TBC1D12//NTF3//TSC1//TBC1D17//ASPH//EFNA1//EPHA4//STAT3//DYNLL2//ST18// |
| GO:0021542 | dentate gyrus development | Biological process | 6 | 15 | 934 | 17653 | 7.56017130620985 | 7.15130139690623e-05 | 0.00158571315892711 | 4.14561491791007 | 0.00642398286937901 | CDK6//DRD1//EMX2//NEUROD1//PTEN//BTG2// |
| GO:0051017 | actin filament bundle assembly | Biological process | 20 | 144 | 934 | 17653 | 2.62505948132286 | 7.17497919381741e-05 | 0.00158576419241134 | 4.14417935397005 | 0.0214132762312634 | PFN2//PLEK//PTGER4//TSC1//LPAR1//PPM1E//NF2//PPM1F//DLC1//S1PR1//TMEFF2//ARHGAP6//MET//PPFIA1//EPS8//RHOC//RND3//MARCKS//MYO1B//LIMA1// |
| GO:0048589 | developmental growth | Biological process | 55 | 609 | 934 | 17653 | 1.70693522923457 | 7.54899700900231e-05 | 0.00166299240299292 | 4.12211074670085 | 0.0588865096359743 | ESR1//EREG//S1PR1//BNC2//RARB//TGFBR2//IGF1//WNT7A//DPYSL2//RAB21//EPHA4//KLF2//EN1//CLIC4//AR//SLC12A5//PLEKHA1//SOS1//TBL1XR1//ARID5B//PKDCC//DLL1//LHX1//STAT3//ADRB1//POU3F2//MTPN//GJA1//GOLGA4//NRG1//BCL2L11//FOXC1//LATS2//PTEN//ARX//SERP1//EFNA5//EPHA7//ULK2//LPAR3//BMPR2//SEMA4B//SLIT2//TGFB2//JARID2//NOG//ZFPM2//GATA6//RDH10//MAP2K4//EDN1//LRP6//PLAA//DUSP10//FLRT1// |
| GO:0009987 | cellular process | Biological process | 885 | 16158 | 934 | 17653 | 1.03520726667838 | 7.57467791687499e-05 | 0.00166323203739693 | 4.12063582908279 | 0.947537473233405 | KIF23//ACOX1//UBXN2B//TRAPPC8//RAB23//ATG16L1//ULK2//IGF1//NFIA//NFIB//RBBP7//RBBP8//RRM2//ESCO2//UBE2I//VBP1//E2F1//USP28//CLOCK//CDKN1A//GADD45A//PTEN//CCNE2//CDK6//LATS2//PPP6C//ACVR1//NPAT//NEDD1//CCNY//PPP1R12A//PRKAR2B//SKP1//YWHAG//MASTL//HAUS8//CEP41//MTRR//TSHZ1//HMG20A//KLF2//ZBTB18//CEBPB//ZNF268//TCERG1//WWP2//KLF12//OSR1//CRY2//GLIS3//ARX//DNMT3B//DR1//EDN1//EFNA1//CC2D1B//EN1//ESR1//FHL2//CPEB3//FOXF1//FOXC1//MYT1L//CIC//ZFPM2//DNAJB5//GATA6//CNOT7//HSBP1//AR//JARID2//JUN//ZNF568//MAF//MDM4//MYB//NR4A2//RLIM//KLF3//ZBTB7A//PIAS4//POU4F1//PPARG//CC2D1A//TSHZ3//ZBTB4//RARB//REST//RNF2//PRDM16//SOX17//SOX11//STAT3//ZEB1//TCF21//TFAP2A//TFAP2C//TGIF1//KLF10//TNF//VLDLR//WFS1//ZNF217//LRP8//BTG2//BHLHE41//TBL1XR1//NR4A3//HMGA2//ARID5B//BRMS1L//BHLHE40//RUNX3//URI1//PER2//SQSTM1//NOG//KLF4//HAND1//FNIP1//PHF14//USP3//WASL//RANBP9//PPM1L//SPRED1//DUSP5//EREG//RASGEF1A//GRB2//NRG1//IL2//JAK1//MAP3K5//MET//MINK1//TAOK3//MAPK1//CCL2//SOS1//LAMTOR3//DCP2//PPP2CA//RPL15//MAP3K2//CRK//CRKL//ADAM9//LPAR1//LPAR3//NTF3//PIK3CB//PRKAA1//MAP2K4//DUSP10//DUSP1//DUSP2//RGS3//TOPORS//HECTD2//RNF145//ZNRF2//FBXL5//RNF180//MYLIP//ANKIB1//UBE2W//FBXW7//HECW2//THOP1//LONRF3//FBXO30//CBFB//UBE3B//LONRF1//ZFAND4//TACC2//DYNC1LI2//ATXN3//NEFH//MAP7D1//MIEF1//MYBL1//CLIP1//SRSF2//CDC23//RHOC//CNOT6L//PAN3//CNOT6//COPS8//RBFOX3//FMR1//MBNL1//RBM25//ELAVL2//NOVA1//BNIP3//PTGES3//NABP1//VASH2//FRY//CDH20//TGFB2//RND3//PAK6//CHMP3//GTF2E1//SECISBP2L//RDH10//NAPEPLD//TTR//CHST7//CHST1//YTHDF2//SOCS5//NANOS1//SPOCK1//TSG101//KIAA1109//GJC1//ITGAV//KDR//TMEM100//TGFBR2//QKI//ITGB8//ST6GALNAC3//ST6GALNAC6//ST8SIA3//ST3GAL5//EPHA2//GJA1//MEF2D//BMP3//TBC1D20//AGFG1//TMF1//ACSL1//DLL1//DSCAML1//SEMA4B//DCX//GPM6A//LRP12//UNK//MSN//BCL2L11//TNFRSF21//PMAIP1//FOXF2//PPP3R1//MOAP1//CAMLG//CLIC4//EPS15//NSF//DRD1//FAM129A//LRP6//PPP1R15B//SLIT2//EFNA5//EPHA7//NPTN//P2RY1//AKTIP//CCND2//SULF1//F3//BMPR2//VEGFB//NF2//WNT7A//PTHLH//LYPLA1//THRA//PRKACB//RPS6KA5//PLEK//RBM47//IFNA1//NKX2-3//SOX4//BAG6//CCL8//LHFPL2//NHLRC2//TMX3//CDC37L1//MIA3//NME1//GRHL1//DUS2//ADAM10//ADRB1//FJX1//GJB2//LHX1//HCN2//CXCL14//ERG//HAND2//NEUROD1//PALLD//BCL11B//S1PR1//GPR6//SPHK2//FRMD6//IRF9//KLF6//CREBRF//EGR2//ELK3//FOXJ3//FLI1//HIVEP2//IRF1//MEOX2//NHLH2//OTX2//PAX9//PBX3//POU3F2//RAD21//ARID4A//SHOX//BTF3//TEF//TGFB1I1//DEK//FOSL1//KLF7//FUBP3//POLR3G//PKD2//E2F5//TSC1//CCNG2//GNB5//DNAJB4//SACS//CCT6A//BAG5//NAP1L3//ANP32E//HSPA13//EPS8//MARCKS//MYO1B//LIMA1//TWF1//FLII//CFL2//CORO2B//DLC1//FGD4//FGD5//PFN2//TRIP10//WASF3//SH3D19//DPYSL2//BRWD1//CCDC6//HIPK3//RTN3//EGLN3//ARL6IP1//C6ORF120//ZFAND6//UBE2Z//FXR1//STK17B//SLK//APCDD1//CTNND2//AMER2//SOSTDC1//HBP1//DKK3//STRN//TCF7L1//FZD6//FAM13A//CNIH1//CDS1//CDC42EP3//IQGAP2//ADCY3//RASSF8//ARAP2//TAGAP//CLCN6//STX2//EVI2A//LRCH1//CD2AP//APPL1//GLRA1//GLRB//RASSF3//RGMB//GRIA1//CD274//NR3C2//MPP1//RASD1//KCNK10//NDFIP2//PPP1R12C//PSD//ARRDC3//MIER1//RHEB//SMOC1//SMOC2//SLC20A1//RASL11B//PDCD1LG2//NSMAF//PPFIA1//RHPN2//OR6A2//ARRDC4//PLAA//ARHGAP12//FEZ2//CD69//PTPRG//AKAP11//DGKH//PIKFYVE//DSTYK//MKNK2//ARHGEF4//TREM1//ARHGEF28//STK33//DGKE//RPS6KA4//PALM2//ERRFI1//MAN2A1//STX6//SYT6//SNAP25//TRIM3//KLHL2//RNF38//MYCBP2//FBXO33//KLHL3//SPOPL//DCAF8//DCAF6//FEM1C//KLHL14//MARCH4//RNF4//MARCH7//RNF128//KLHL15//KBTBD8//UGP2//PFKP//GFPT2//NAGK//SLC35D1//IDH1//ACO1//NUP35//NUP43//NLN//IMMP2L//CHCHD10//COX8C//COX7A2L//DNAJC16//GLRX//COX7A2//ASPH//GSTA2//HPRT1//CMPK1//IMPDH1//PDE10A//DCTPP1//REV3L//GMNC//EPC2//USP45//KDM2A//HMGB3//ZMYND11//ATAD2//H2AFZ//RSBN1//RBL2//BRD3//NFE2//SMARCD2//KAT2B//BAHD1//KMT2B//DPY30//ARID4B//KHDRBS3//TADA1//ZNF597//ZNF782//ZNF800//ZNF367//EZH1//ZBTB9//LHX6//SERTAD3//MDFIC//MNX1//HOXA3//HOXD1//FOXN2//ZBTB41//ZBTB34//LHX8//NEUROG1//NPAS2//IER5//BNC2//ZNF532//CDCA7L//TRERF1//LMO3//SLC2A4RG//PHTF2//ZNF630//ZNF287//ZNF512B//ZBTB26//ATXN1//DMRTA1//STAT4//ZNF2//ZNF3//ZNF131//ZNF655//PCGF5//LCOR//ZBTB47//ST18//NR5A2//NRBF2//MLLT6//RNF141//SNAPC1//BTG1//SNIP1//EMX2//ETS1//BOLA3//ZNFX1//NKX2-4//RFX7//BDP1//CDK8//E2F3//TEAD1//CCNC//CMTR2//ZFC3H1//RTCA//PPP4R2//ESRP1//CPEB1//CPEB2//CPEB4//MTPN//DDX6//FYCO1//SERP1//UEVLD//RIMKLB//FASTK//PDIK1L//CDK19//MORC3//MMD//CDK17//FAM20C//PPM1E//FLRT1//GNAQ//NCK1//ASPN//CAMK2N2//PPM1F//PTPN21//CTDSPL2//PTPN4//NAA30//HS3ST5//PCMTD1//FUT9//ST8SIA5//MAN1A1//B3GNT5//B3GALT2//CCDC126//EDEM1//NTNG1//RECK//PTAR1//PIGA//CTSA//USP24//USP27X//NPLOC4//USP46//SLC6A14//SLC6A6//GLUD2//LGSN//SLC25A32//BCAT2//SERINC3//PSAT1//MTX3//ACOT7//UGT1A8//MSMO1//ELOVL4//SCD5//LCLAT1//DDHD1//ABHD3//SLC44A1//PLEKHA1//PLEKHA3//MTMR9//PIK3R3//KDELC1//SGPP1//ORMDL3//ZADH2//UGT1A4//UGT1A1//SESN1//UGT1A6//UGT1A9//SLC12A5//SLC24A2//SLC24A3//STIM2//EGLN1//CYBRD1//EXOC5//RAB12//TBC1D17//TECPR2//ZMAT3//REEP3//RAB38//TMOD3//TMOD1//KIF5A//KIF13A//STMN2//SLAIN2//MID1IP1//GOLGA8A//RAB30//TMED9//USP6NL//TMEM135//RAB22A//FAM160A2//ITGA6//ITGA5//CETN1//MAPRE3//PTP4A1//EVI5//LMLN//MTBP//RRAGD//DYNLL2//CENPQ//CENPO//CNEP1R1//CDC14A//SYCP3//SNTG1//ENPP5//GAB1//COL1A2//ZFYVE9//RAPGEF4//FZD10//CHRM2//CHRM5//CMKLR1//GPR180//FRZB//GPRC5C//PTGER4//SOS2//CALM2//EPN1//TMEM17//DZIP1//NKIRAS1//G3BP2//RGL1//DOCK9//ARHGAP1//RALGPS2//DOCK5//ARHGAP6//SLC6A1//SLC32A1//TDRD5//SLITRK3//OGN//EPHA5//ENAH//NTN4//ADAM12//TRPC3//CADM2//EPHA4//RAB21//ACTC1//GIT2//BAMBI//OSM//DERL2//TOB1//GDF11//TOB2//BTG3//TES//CLDN11//PLSCR4//ACBD5//PPCS//DSC2//INHBB//PLEKHF2//PTPDC1//C5ORF30//CBLN4//ADIPOR2//UGT1A7//RGS17//KCNJ3//KCNJ10//FCHO2//NPNT//LDLRAD4//LDLR//PPP1R37//SERPINI1//PCOLCE2//SLC41A1//ZNF804A//PMEPA1//SLC17A7//XYLT2//HS2ST1//SNX5//SNX2//PRR16//INTS8//VPS37A//SESN3//GNPTG//ST3GAL3//HDHD2//PEX5L//BTAF1//DMWD//OTUD3//WDR20//JOSD1//CAPRIN2//TRPV6//VKORC1L1//PKDCC//ZDHHC1//ST6GAL2//MCFD2//ZDHHC5//CRTAP//MMP2//SCN1A//ELOVL7//PON2//SYBU//C21ORF91//LAMA3//NSMF//IPO5//DENND1B//RUFY2//SPTSSA//ZIC5//ADAM18//SHISA2//DNAJB9//HAPLN1//MFAP5//DSEL//OSTM1//TMEFF2//RAP2C//UBXN4//JKAMP//RGMA//FAM118B//CNN3//TBC1D12//KRT27//AGFG2//RAB9B//DNAJC27//RAB28//RAP1B//SLC2A4//MEMO1//UBA5//UXS1//NEFM//ARFIP1//RAB3GAP1//DNAJB12//SLC17A6//MCOLN2//WDFY3//OSBPL5//KCTD20//BTBD10//GNS//APOO//SBF2//UGT1A3//KCNA4//ITM2B//CKAP4//ENPP4//PGRMC2//AHNAK//AIDA//XKR8//ANO8//CCDC71L//FAM135A//TMEM64//STEAP2//PLEKHB2//CEP97//GOLGA4//PAIP2//MDM1//EFR3A//ZFYVE21//PPT2//SEC23A//SAR1B//PXK//MZT1//SLC4A4//UGT1A10//UGT1A5//VANGL1//NECAP1//SGCE//FIBIN//REXO1//ZC3H12C//FAM155A//CALCOCO2//LYPD6//ELMOD1//IER3IP1// |
| GO:0001654 | eye development | Biological process | 36 | 344 | 934 | 17653 | 1.97795179522932 | 7.60831670874575e-05 | 0.00166521184146432 | 4.11871141746945 | 0.0385438972162741 | STAT3//GJA1//TGFBR2//SOX11//TFAP2A//RARB//GPM6A//SLC17A7//TGFB2//TOPORS//FJX1//LHX1//RDH10//BCL11B//KLF4//NEUROD1//RAB3GAP1//FOXC1//GDF11//FOXF2//LRP6//ZEB1//DLL1//MDM1//MAN2A1//JUN//SOS1//BMPR2//CLIC4//MAF//NF2//TMOD1//WNT7A//TBC1D20//EPHA2//SMOC1// |
| GO:0030148 | sphingolipid biosynthetic process | Biological process | 16 | 101 | 934 | 17653 | 2.9941272499841 | 7.68216395445643e-05 | 0.00167595080077383 | 4.11451642826224 | 0.0171306209850107 | ST6GALNAC3//ST6GALNAC6//ST8SIA3//ST3GAL5//SPHK2//B3GALT2//ST8SIA5//PRKAA1//SPTSSA//ORMDL3//TNF//NSMAF//PPM1L//ELOVL4//ELOVL7//SGPP1// |
| GO:0003158 | endothelium development | Biological process | 18 | 123 | 934 | 17653 | 2.76591633154019 | 8.19148745867308e-05 | 0.00178131928241177 | 4.08663722932158 | 0.019271948608137 | CLIC4//MET//WNT7A//S1PR1//ZEB1//TMEM100//BTG1//BMPR2//DLL1//NRG1//SOX17//MSN//RAP1B//ACVR1//RAP2C//TNF//GJA1//KDR// |
| GO:0009966 | regulation of signal transduction | Biological process | 215 | 3215 | 934 | 17653 | 1.26394776892311 | 8.31352658997935e-05 | 0.00180206347205225 | 4.08021470982901 | 0.230192719486081 | GADD45A//MINK1//TNF//MAP3K2//CRK//CRKL//MAP3K5//TAOK3//LAMTOR3//ADAM9//DUSP5//LPAR1//LPAR3//NRG1//IGF1//NTF3//PIK3CB//PRKAA1//MAPK1//MAP2K4//DUSP10//SPRED1//DUSP1//DUSP2//PPP2CA//RGS3//CBFB//THRA//SOX17//EREG//FBXW7//ERRFI1//TSG101//SOCS5//NCK1//COPS8//MDFIC//GIT2//OTX2//TOB1//EPS8//ARHGAP1//ARHGAP6//EPHA2//EPHA4//EPHA5//EPHA7//RGS17//KDR//MET//ACVR1//GDF11//EDN1//IFNA1//IL2//INHBB//OGN//OSM//P2RY1//PTHLH//CCL2//CCL8//BMP3//TGFB2//TTR//VEGFB//WNT7A//CXCL14//F3//PDE10A//AKAP11//PIAS4//BMPR2//PMEPA1//LDLRAD4//PTEN//KLF4//PLEK//DKK3//SOX11//ZEB1//ESR1//TCF7L1//SULF1//SOX4//APCDD1//FRZB//SOSTDC1//SHISA2//NPNT//TGFB1I1//BAMBI//ASPN//PRDM16//RASL11B//TGFBR2//GATA6//CTDSPL2//NOG//ITGA5//PKD2//TSC1//FNIP1//RRAGD//RHEB//ARAP2//APPL1//AGFG1//AGFG2//PSD//RALGPS2//UBA5//AR//IRF1//FGD4//FGD5//ARHGEF4//ARHGEF28//SOS1//SOS2//DLC1//NF2//LMO3//NPLOC4//FAM20C//EPS15//GRB2//EPN1//CNOT7//STAT3//ZFAND6//SQSTM1//GJA1//RHOC//NDFIP2//CC2D1A//MIER1//SLC20A1//UBE2I//ZMYND11//PAK6//EFNA1//ITGAV//FZD10//HIPK3//AIDA//PMAIP1//PEX5L//ADRB1//DSTYK//NPTN//WWP2//YTHDF2//DLL1//KAT2B//FLRT1//NEUROD1//RASGEF1A//SESN3//CMKLR1//TMEM100//FAM13A//TAGAP//TRIP10//ARHGAP12//CD2AP//SLIT2//GAB1//OTUD3//PLEKHA1//CALM2//JAK1//PPARG//TCF21//CTNND2//AMER2//CCNY//LRP6//RANBP9//JUN//DNAJC27//HAND2//ARRDC3//TMEM64//LATS2//FZD6//BCL2L11//MOAP1//BNIP3//CAPRIN2//TBL1XR1//LYPD6//CREBRF//DYNLL2//E2F1//PPP3R1//YWHAG//PRKACB//MDM4//POU4F1//RBBP7//RBBP8//BAG5//WFS1//SERINC3//DNAJB9//PPP1R15B//SESN1//CRY2//CLOCK//PHF14//NR4A2//ITGA6//URI1//ST18// |
| GO:0044257 | cellular protein catabolic process | Biological process | 63 | 730 | 934 | 17653 | 1.6311328503124 | 8.76812955031881e-05 | 0.0018945322731248 | 4.05709304205166 | 0.0674518201284797 | ZFAND4//CTSA//TOPORS//USP24//KLHL3//MYLIP//USP27X//ATXN3//RLIM//NPLOC4//USP28//USP46//UBE2I//BAG6//RNF128//KLHL15//USP45//CDC23//SQSTM1//USP3//UBE2W//TMF1//PMAIP1//UBXN4//DNAJB9//DERL2//JKAMP//WFS1//EDEM1//TGFB1I1//FBXL5//FBXW7//SKP1//BAG5//RNF180//ANKIB1//LONRF3//LONRF1//SOCS5//VLDLR//BNIP3//DNAJB12//WWP2//UBXN2B//RNF38//RNF145//ZNRF2//CD2AP//SPOPL//HECW2//RNF4//TBL1XR1//PLAA//CLOCK//VPS37A//TSG101//FMR1//EFNA1//EPHA4//MSN//LDLR//PTEN//RAB12// |
| GO:0023014 | signal transduction by protein phosphorylation | Biological process | 79 | 973 | 934 | 17653 | 1.53456714591618 | 9.0732366163874e-05 | 0.00195421335148497 | 4.04223776322705 | 0.0845824411134904 | RANBP9//PPM1L//SPRED1//DUSP5//EREG//RASGEF1A//GRB2//NRG1//IL2//JAK1//MAP3K5//MET//MINK1//TAOK3//MAPK1//CCL2//SOS1//TNF//LAMTOR3//GADD45A//MAP3K2//CRK//CRKL//ADAM9//LPAR1//LPAR3//IGF1//NTF3//PIK3CB//PRKAA1//MAP2K4//DUSP10//DUSP1//DUSP2//PPP2CA//RGS3//PTGER4//MDFIC//OSR1//TGFB2//TSG101//EDN1//GDF11//INHBB//PAK6//BMP3//EFNA1//NF2//EPHA2//EPHA4//EPHA5//EPHA7//AR//ITGAV//KDR//OSM//FZD10//HIPK3//AIDA//ZMYND11//WNT7A//SKP1//ERRFI1//LMO3//PTEN//KLF4//NPNT//DSTYK//NPTN//JUN//P2RY1//DNAJC27//FBXW7//CCL8//HAND2//BMPR2//TGFBR2//ACVR1//SLK// |
| GO:0071407 | cellular response to organic cyclic compound | Biological process | 52 | 572 | 934 | 17653 | 1.71822075141133 | 9.97922812568898e-05 | 0.00214252443854078 | 4.00090304926554 | 0.0556745182012848 | SNIP1//ESR1//FHL2//AR//PMEPA1//RNF4//TGFB1I1//UBA5//CBFB//PGRMC2//NR5A2//NR3C2//NR4A2//PPARG//RARB//THRA//NR4A3//TCF21//PKD2//RAP1B//HCN2//URI1//BCL2L11//EDN1//UGT1A1//REST//H2AFZ//POU4F1//MSN//LRP6//LRP8//KLF2//KLF4//DNMT3B//GJB2//ERRFI1//DRD1//CREBRF//MAPK1//ADCY3//EFNA5//CRY2//CLOCK//LMO3//CEBPB//FOXF1//ITGA6//P2RY1//CCL2//STAT3//TMF1//TNF// |
| GO:0048286 | lung alveolus development | Biological process | 9 | 37 | 934 | 17653 | 4.59740146999248 | 0.000101596253635879 | 0.00217435273208687 | 3.99312230637555 | 0.00963597430406852 | GATA6//FOXF1//CIC//MAN2A1//ERRFI1//BMPR2//TCF21//PKDCC//PHF14// |
| GO:0061572 | actin filament bundle organization | Biological process | 20 | 148 | 934 | 17653 | 2.5541119277736 | 0.000105706176590304 | 0.00225517625324992 | 3.97589963539159 | 0.0214132762312634 | EPS8//RHOC//RND3//MARCKS//MYO1B//LIMA1//PFN2//PLEK//PTGER4//TSC1//LPAR1//PPM1E//NF2//PPM1F//DLC1//S1PR1//TMEFF2//ARHGAP6//MET//PPFIA1// |
| GO:0051716 | cellular response to stimulus | Biological process | 455 | 7556 | 934 | 17653 | 1.13812795934538 | 0.000106237664799644 | 0.00225938782088048 | 3.97372148405828 | 0.487152034261242 | E2F1//USP28//CLOCK//RANBP9//PPM1L//SPRED1//DUSP5//EREG//RASGEF1A//GRB2//NRG1//IL2//JAK1//MAP3K5//MET//MINK1//TAOK3//MAPK1//CCL2//SOS1//TNF//LAMTOR3//GADD45A//MAP3K2//CRK//CRKL//ADAM9//LPAR1//LPAR3//IGF1//NTF3//PIK3CB//PRKAA1//MAP2K4//DUSP10//DUSP1//DUSP2//PPP2CA//RGS3//COPS8//RBBP8//NABP1//JUN//PMAIP1//BCL2L11//MOAP1//CBFB//P2RY1//THRA//PRKACB//SKP1//RPS6KA5//BAG6//CCL8//SLIT2//SOX17//S1PR1//GPR6//SPHK2//APCDD1//CTNND2//AMER2//CCNY//SOSTDC1//HBP1//DKK3//LRP6//PIAS4//STRN//TGFB1I1//WNT7A//TCF7L1//PPP3R1//FZD6//FAM13A//CNIH1//CDK6//DLC1//CDS1//CDC42EP3//GNB5//PTGES3//IQGAP2//ADCY3//RASSF8//ARAP2//TAGAP//CLCN6//DPYSL2//ELK3//STX2//EPS8//ERG//ESR1//EVI2A//LRCH1//CD2AP//APPL1//GJA1//GLRA1//GLRB//RASSF3//RGMB//GRIA1//CD274//CNOT7//LRP12//HIVEP2//AR//NR3C2//MPP1//PPP1R12A//NR4A2//RASD1//KCNK10//NDFIP2//PPARG//PPP1R12C//CC2D1A//PSD//ARRDC3//MIER1//RARB//RHEB//SMOC1//SMOC2//BCL11B//SLC20A1//RASL11B//SPOCK1//STAT3//VLDLR//ZNF217//LRP8//DEK//PDCD1LG2//CAMLG//NSMAF//PPFIA1//RHPN2//OR6A2//ARRDC4//TRIP10//PLAA//ARHGAP12//CXCL14//FEZ2//CD69//ULK2//KDR//PTPRG//AKAP11//DGKH//DCX//PIKFYVE//DSTYK//LATS2//MKNK2//ARHGEF4//TREM1//PRKAR2B//ARHGEF28//STK33//MASTL//DGKE//SQSTM1//RPS6KA4//STK17B//PALM2//BMPR2//FBXW7//ERRFI1//TSG101//SOCS5//EPC2//UBE2W//BTG2//USP45//USP3//HMGA2//ATXN3//ESCO2//RAD21//KDM2A//UGT1A6//UGT1A9//TOPORS//CDKN1A//EGLN3//FMR1//NPAS2//ZBTB4//ZMAT3//IMMP2L//CNOT6L//MDM4//CNOT6//RBL2//SOX4//WFS1//PPP1R15B//IRF9//EDN1//EFNA5//NCK1//EPS15//GAB1//COL1A2//TGFB2//TGFBR2//KLF10//ACVR1//ZFYVE9//PDE10A//RAPGEF4//FZD10//CHRM2//CHRM5//FGD4//CMKLR1//ADRB1//GPR180//DRD1//FRZB//GNAQ//RHOC//GPRC5C//PTGER4//PTHLH//SOS2//CALM2//ADAM10//FOXC1//DLL1//EPN1//TMEM100//KAT2B//TMEM17//DZIP1//FOXF1//ITGA6//ITGA5//ITGAV//ITGB8//PLEK//OSR1//NKIRAS1//SNIP1//MDFIC//PKD2//STAT4//NEUROD1//RGL1//DOCK9//RAB38//RND3//ARHGAP1//RALGPS2//DOCK5//G3BP2//ARHGAP6//PAK6//TRPC3//GIT2//PIK3R3//FLRT1//ESRP1//OTX2//BNIP3//EPHA2//DSC2//INHBB//RRAGD//PLEKHF2//FNIP1//NR5A2//ADIPOR2//CRY2//YWHAG//TOB1//EPHA4//EPHA5//EPHA7//RGS17//GDF11//IFNA1//OGN//OSM//BMP3//TTR//VEGFB//PPP4R2//F3//PMEPA1//LDLRAD4//PLEKHA1//PTEN//KLF4//SOX11//ZEB1//MMP2//ASPN//CEBPB//SESN3//CPEB3//NSMF//SESN1//IPO5//CPEB1//CPEB4//DENND1B//TNFRSF21//SLK//MTPN//IRF1//SULF1//SHISA2//UBXN4//DNAJB9//DERL2//JKAMP//NPLOC4//EDEM1//RGMA//NOG//NPNT//BAMBI//PRDM16//GATA6//CTDSPL2//FHL2//RNF4//NR4A3//CREBRF//SERP1//FOSL1//TSC1//AGFG1//AGFG2//RAB12//RAB21//RAB30//RAB9B//DNAJC27//RAB23//RAB22A//RAB28//RAP2C//RAP1B//CPEB2//SLC2A4//UBA5//ST18//ACSL1//VKORC1L1//GJB2//CETN1//IER5//HSPA13//FAM129A//NRBF2//TMX3//FGD5//TEAD1//NF2//LMO3//PPM1E//NME1//REST//PPM1F//GSTA2//MSN//SYBU//KLF2//DNAJB12//WASL//BRWD1//FAM20C//DCTPP1//REV3L//ZFAND6//UBE2I//ZMYND11//PGRMC2//EFNA1//HIPK3//AIDA//PEX5L//TMEM64//LHX1//NPTN//WWP2//YTHDF2//ARID5B//OTUD3//UGT1A10//UGT1A8//UGT1A7//UGT1A5//UGT1A4//UGT1A1//UGT1A3//VANGL1//RAB3GAP1//SLC17A7//TCF21//EGLN1//ETS1//MYB//HSBP1//RBBP7//HAND2//FIBIN//CDK19//PLSCR4//CLIC4//TFAP2A//SLC41A1//EGR2//POU3F2//HCN2//POU4F1//PTPN4//PAX9//TGIF1//URI1//H2AFZ//LDLR//TMF1//DNMT3B//CCND2//SEMA4B//KLHL15//CAPRIN2//TBL1XR1//FASTK//SGPP1//ITM2B//LYPD6//NUP35//NUP43//BAG5//DYNLL2//KLF3//SERINC3//STMN2//DYNC1LI2//PCOLCE2//JARID2//NEFH//PFKP//PIGA//BCAT2//GFPT2//PHF14// |
| GO:0035970 | peptidyl-threonine dephosphorylation | Biological process | 6 | 16 | 934 | 17653 | 7.08766059957173 | 0.000109308390240381 | 0.00231740640500218 | 3.96134650140574 | 0.00642398286937901 | DUSP10//DUSP1//DUSP5//PPM1E//PPP2CA//PPM1F// |
| GO:0035265 | organ growth | Biological process | 22 | 172 | 934 | 17653 | 2.41749663861362 | 0.000111884092114786 | 0.00236460035928843 | 3.95123165786745 | 0.0235546038543897 | S1PR1//BNC2//RARB//TGFBR2//BCL2L11//FOXC1//LATS2//PTEN//ARX//SERP1//TGFB2//GJA1//JARID2//NOG//ZFPM2//GATA6//ESR1//AR//MAP2K4//EDN1//IGF1//DLL1// |
| GO:0007154 | cell communication | Biological process | 411 | 6744 | 934 | 17653 | 1.15184994322815 | 0.000113014139142854 | 0.00238104243932437 | 3.94686721875035 | 0.440042826552463 | RANBP9//PPM1L//SPRED1//DUSP5//EREG//RASGEF1A//GRB2//NRG1//IL2//JAK1//MAP3K5//MET//MINK1//TAOK3//MAPK1//CCL2//SOS1//TNF//LAMTOR3//GADD45A//MAP3K2//CRK//CRKL//ADAM9//LPAR1//LPAR3//IGF1//NTF3//PIK3CB//PRKAA1//MAP2K4//DUSP10//DUSP1//DUSP2//PPP2CA//RGS3//JUN//PMAIP1//BCL2L11//MOAP1//CBFB//DRD1//P2RY1//THRA//PRKACB//SKP1//RPS6KA5//BAG6//ADAM10//ADRB1//EDN1//EFNA1//FJX1//GJA1//GJB2//AR//LHX1//PTHLH//HCN2//CCL8//BMP3//TFAP2C//TGFB2//KLF10//CXCL14//HMGA2//SOX17//S1PR1//GPR6//SPHK2//APCDD1//CTNND2//AMER2//CCNY//SOSTDC1//HBP1//DKK3//LRP6//PIAS4//STRN//TGFB1I1//WNT7A//TCF7L1//PPP3R1//FZD6//FAM13A//CNIH1//CDK6//DLC1//CDS1//CDC42EP3//GNB5//PTGES3//IQGAP2//ADCY3//RASSF8//ARAP2//TAGAP//CLCN6//DPYSL2//ELK3//STX2//EPS8//ERG//ESR1//EVI2A//LRCH1//CD2AP//APPL1//GLRA1//GLRB//RASSF3//RGMB//GRIA1//CD274//CNOT7//LRP12//HIVEP2//NR3C2//MPP1//PPP1R12A//NR4A2//RASD1//KCNK10//NDFIP2//PPARG//PPP1R12C//CC2D1A//PSD//ARRDC3//MIER1//RARB//RHEB//SMOC1//SMOC2//BCL11B//SLC20A1//RASL11B//SPOCK1//STAT3//VLDLR//ZNF217//LRP8//DEK//PDCD1LG2//CAMLG//NSMAF//PPFIA1//RHPN2//OR6A2//ARRDC4//TRIP10//PLAA//ARHGAP12//CLOCK//FEZ2//CD69//ULK2//KDR//PTPRG//AKAP11//DGKH//DCX//PIKFYVE//DSTYK//LATS2//MKNK2//ARHGEF4//TREM1//PRKAR2B//ARHGEF28//STK33//MASTL//DGKE//SQSTM1//RPS6KA4//STK17B//PALM2//BMPR2//FBXW7//ERRFI1//TSG101//SOCS5//CDKN1A//E2F1//CNOT6L//MDM4//CNOT6//RBL2//SOX4//BTG2//WFS1//PPP1R15B//IRF9//EFNA5//NCK1//EPS15//GAB1//COL1A2//TGFBR2//ACVR1//ZFYVE9//PDE10A//RAPGEF4//FZD10//CHRM2//CHRM5//FGD4//CMKLR1//GPR180//FRZB//GNAQ//RHOC//GPRC5C//PTGER4//SOS2//CALM2//FMR1//FOXC1//DLL1//EPN1//TMEM100//KAT2B//TMEM17//DZIP1//FOXF1//ITGA6//ITGA5//ITGAV//ITGB8//PLEK//OSR1//NKIRAS1//SNIP1//COPS8//MDFIC//PKD2//STAT4//NEUROD1//RGL1//DOCK9//RAB38//RND3//ARHGAP1//RALGPS2//DOCK5//USP28//G3BP2//ARHGAP6//PAK6//GJC1//KIF5A//ATXN3//NOVA1//SLC12A5//SLC6A1//SNAP25//SLC32A1//PTEN//TRPC3//MARCKS//PER2//GIT2//PIK3R3//FLRT1//ESRP1//OTX2//BNIP3//TOPORS//EPHA2//DSC2//INHBB//RRAGD//PLEKHF2//FNIP1//NR5A2//ADIPOR2//CRY2//YWHAG//TOB1//EPHA4//EPHA5//EPHA7//RGS17//GDF11//IFNA1//OGN//OSM//TTR//VEGFB//F3//PMEPA1//LDLRAD4//PLEKHA1//KLF4//SOX11//ZEB1//MMP2//ASPN//SCN1A//DENND1B//TNFRSF21//SLK//SULF1//SHISA2//RGMA//NOG//NPNT//BAMBI//PRDM16//GATA6//CTDSPL2//FHL2//RNF4//NR4A3//CREBRF//SERP1//DERL2//FOSL1//TSC1//AGFG1//AGFG2//USP46//TMF1//RAB12//RAB21//RAB30//RAB9B//DNAJC27//RAB23//RAB22A//RAB28//RAP2C//RAP1B//UBA5//ST18//ACSL1//IRF1//SESN3//SESN1//FGD5//CPEB4//SLC17A7//SLC17A6//TEAD1//NF2//LMO3//SLIT2//GSTA2//MSN//SYBU//DNAJB9//EDEM1//WASL//BRWD1//NPLOC4//FAM20C//DCTPP1//ZFAND6//UBE2I//ZMYND11//PGRMC2//HIPK3//AIDA//PEX5L//TMEM64//NPTN//WWP2//YTHDF2//REST//ARID5B//CPEB3//EGR2//NSMF//KCNJ10//RAB3GAP1//SYT6//PXK//OTUD3//TSHZ3//VANGL1//SLC24A2//NTN4//TCF21//CEBPB//HAND2//FIBIN//SEMA4B//POU4F1//ZMAT3//CAPRIN2//TBL1XR1//FASTK//SGPP1//ITM2B//PPM1F//KCNJ3//FXR1//LYPD6//PAIP2//DYNLL2//RBBP7//RBBP8//BAG5//SERINC3//PFN2//PHF14//MYB//URI1//SNTG1//ENPP5// |
| GO:0048585 | negative regulation of response to stimulus | Biological process | 119 | 1615 | 934 | 17653 | 1.39266313535445 | 0.00011376655630435 | 0.00238945099467801 | 3.9439853877399 | 0.127408993576017 | DUSP10//SPRED1//DUSP1//DUSP2//DUSP5//PPP2CA//RGS3//PPARG//SLIT2//TNF//SOX17//ERRFI1//TSG101//SOCS5//EPHA2//EPHA4//EPHA5//EPHA7//RGS17//KDR//MET//ACVR1//PIAS4//TGFB2//PMEPA1//LDLRAD4//PTEN//KLF4//PLEK//APCDD1//FRZB//SOSTDC1//SHISA2//EDN1//BAMBI//ASPN//PRDM16//RASL11B//TGFB1I1//TGFBR2//TOB1//CTDSPL2//NOG//PRKAA1//TSC1//FNIP1//DLC1//NPLOC4//SULF1//EPS15//GRB2//EPN1//NF2//ZMYND11//ESR1//FOXF1//EFNA1//HIPK3//AIDA//LPAR1//WWP2//YTHDF2//FBXW7//CRK//TAOK3//FLRT1//NEUROD1//NCK1//RGMA//SEMA4B//ETS1//IL2//PTGER4//CD2AP//ARHGAP12//OTUD3//PLEKHA1//DRD1//PKD2//CALM2//CNOT7//TCF21//GJA1//RANBP9//LMO3//TMEM64//AMER2//LATS2//DKK3//LRP6//FZD6//IGF1//C5ORF30//CREBRF//EREG//NRG1//PRKACB//BAG5//WFS1//NR4A3//DNAJB9//PPP1R15B//DERL2//SESN3//SESN1//OSR1//KLHL15//LYPD6//CRY2//CLOCK//CCL2//PHF14//CDK6//HMGA2//NR4A2//ITGA6//AR//ITGAV//URI1// |
| GO:0051248 | negative regulation of protein metabolic process | Biological process | 104 | 1374 | 934 | 17653 | 1.43060010161176 | 0.000116776178914392 | 0.00244409289586344 | 3.93264573959633 | 0.11134903640257 | DUSP10//SPRED1//DUSP1//DUSP2//DUSP5//PPP2CA//RGS3//FAM129A//IL2//LRP6//PTEN//PPP1R15B//SLIT2//ERRFI1//TSG101//SOCS5//GADD45A//PPM1E//FLRT1//GNAQ//NCK1//NF2//TAOK3//ASPN//DUS2//YWHAG//CAMK2N2//PPM1F//SNIP1//CALM2//SERPINI1//SPOCK1//RECK//TOB1//CPEB3//FMR1//CNOT7//NANOS1//CAPRIN2//TSC1//BTG2//FXR1//JARID2//SPOPL//SOX4//PER2//BAG5//CDK6//JUN//EDN1//BAG6//CRY2//PPP1R37//MASTL//URI1//HMG20A//RNF4//CNOT6//STAT3//TNF//RNF128//NRG1//MDM4//ITM2B//ARL6IP1//KLF4//FNIP1//EFNA1//HIPK3//AIDA//ITGAV//CDKN1A//LATS2//IPO5//KAT2B//PAIP2//ZMYND11//INHBB//NTF3//DNMT3B//PMEPA1//LDLRAD4//NOG//NUP35//NUP43//PPARG//CNOT6L//YTHDF2//QKI//RANBP9//LMO3//PLAA//CPEB2//CRTAP//EPHA4//ESR1//DERL2//FRY//CTSA//LDLR//PRKAR2B//CPEB1//CPEB4//UNK// |
| GO:0072359 | circulatory system development | Biological process | 87 | 1105 | 934 | 17653 | 1.48808801728565 | 0.000117090950504178 | 0.00244409289586344 | 3.93147666853112 | 0.0931477516059957 | S1PR1//EFNA1//EPHA2//ELK3//EREG//FOXC1//GAB1//CLIC4//HOXA3//ITGA5//ITGAV//JUN//KDR//MEOX2//MMP2//TMEM100//PTEN//CCL2//SOX17//TGFB2//VEGFB//WNT7A//HAND1//HAND2//COL1A2//CRKL//FOXF1//SPHK2//TGFBR2//EDN1//PPP3R1//ACVR1//GJC1//ZFPM2//QKI//FBXW7//TCF21//BMPR2//GJA1//DLL1//PKD2//RECK//SLIT2//DLC1//GATA6//RARB//NOG//SOX11//NRG1//SOX4//ERG//SOS1//POU4F1//LRP6//OSR1//GNAQ//MEF2D//PPARG//ADIPOR2//KAT2B//SPRED1//SULF1//KLF4//ETS1//EGLN1//VASH2//F3//ITGB8//STAT3//BTG1//ADAM12//HMGA2//ACTC1//TSC1//FHL2//JARID2//PIK3CB//REST//ERRFI1//FOSL1//MAPK1//MAP2K4//IGF1//IMMP2L//KLF2//EPN1//JAK1// |
| GO:0042493 | response to drug | Biological process | 78 | 966 | 934 | 17653 | 1.52612153696783 | 0.000120117557524558 | 0.00249955397396488 | 3.92039350734544 | 0.0835117773019272 | DRD1//HPRT1//NR4A2//ARL6IP1//SLC17A7//SLC17A6//CHRM2//CHRM5//GNAQ//EDN1//SLC32A1//GJA1//SLC25A32//DNMT3B//PPARG//PRKAA1//GJB2//TGFB2//FOSL1//PTEN//TRPC3//MAPK1//PPM1E//PPP1R12A//NME1//RAP1B//REST//PPM1F//CD69//SLC6A1//CRK//DUSP1//JUN//NR4A3//PPP1R15B//ADAM9//IL2//RPL15//SLC2A4//STAT3//ACTC1//EPS8//USP46//SLIT2//KCNJ10//PER2//KLF2//ETS1//MAP3K5//MYB//PLEKHA1//BNIP3//KLF4//TNF//GLRA1//UGT1A1//FIBIN//ERRFI1//EGLN1//FMR1//PRKACB//PRKAR2B//MET//ADCY3//EFNA5//KDR//OSR1//HMGA2//CDKN1A//EMX2//ACSL1//GATA6//NEUROD1//SLC12A5//TGFBR2//TGIF1//VEGFB//LRP8// |
| GO:0010001 | glial cell differentiation | Biological process | 24 | 197 | 934 | 17653 | 2.30259024554615 | 0.000120695773013723 | 0.00250388194138592 | 3.91830793942179 | 0.0256959314775161 | CDK6//DRD1//DLL1//POU3F2//WASF3//LPAR1//SOX11//EGR2//NRG1//SOX4//KCNJ10//PPP3R1//PTEN//STAT3//DUSP10//BNIP3//EPHA4//NOG//TNFRSF21//PPARG//RHEB//MAPK1//LDLR//NFIB// |
| GO:0001701 | in utero embryonic development | Biological process | 33 | 312 | 934 | 17653 | 1.99908375885357 | 0.000122023258343109 | 0.00251643418748518 | 3.9135573823797 | 0.0353319057815846 | HAND1//LATS2//SOX17//RBBP8//CEBPB//PKD2//ZNF568//GRB2//EGLN1//MAPK1//VASH2//IGF1//BCL2L11//ADAM10//KLF2//RDH10//EDN1//FOXF1//FOXC1//ZFPM2//GATA6//GJA1//EPN1//AR//MAN2A1//MBNL1//TMEM100//BTF3//TGFBR2//FOSL1//ACVR1//NOG//HAND2// |
| GO:0061138 | morphogenesis of a branching epithelium | Biological process | 23 | 185 | 934 | 17653 | 2.34978297355171 | 0.000122045011606556 | 0.00251643418748518 | 3.91347996697207 | 0.0246252676659529 | EDN1//PPP3R1//TGFBR2//ACVR1//NPNT//LHX1//PKD2//TCF21//CLIC4//MET//SLIT2//RDH10//FOXF1//EPHA2//LRP6//NOG//AR//ESR1//NTN4//GRB2//SULF1//BTBD7//TNF// |
| GO:0060325 | face morphogenesis | Biological process | 8 | 30 | 934 | 17653 | 5.0401142041399 | 0.000122948246229542 | 0.00252178043142989 | 3.91027766178712 | 0.00856531049250535 | RAB3GAP1//LRP6//MMP2//ASPH//PAX9//PLEKHA1//ARID5B//NOG// |
| GO:0045446 | endothelial cell differentiation | Biological process | 16 | 105 | 934 | 17653 | 2.88006525950851 | 0.000123411930866436 | 0.00252178043142989 | 3.90864285279072 | 0.0171306209850107 | CLIC4//MET//WNT7A//ZEB1//TMEM100//BTG1//BMPR2//DLL1//NRG1//SOX17//MSN//RAP1B//ACVR1//RAP2C//TNF//S1PR1// |
| GO:0071383 | cellular response to steroid hormone stimulus | Biological process | 29 | 260 | 934 | 17653 | 2.10812469115467 | 0.000123422936981117 | 0.00252178043142989 | 3.90860412329583 | 0.0310492505353319 | ESR1//FHL2//AR//PMEPA1//RNF4//TGFB1I1//UBA5//CBFB//PGRMC2//NR5A2//NR3C2//NR4A2//PPARG//RARB//THRA//NR4A3//TCF21//BCL2L11//EDN1//UGT1A1//REST//DNMT3B//GJB2//ERRFI1//CREBRF//CRY2//CLOCK//LMO3//URI1// |
| GO:0030163 | protein catabolic process | Biological process | 72 | 875 | 934 | 17653 | 1.5552352401346 | 0.00012435504607914 | 0.00253317221877477 | 3.90533658723472 | 0.0770877944325482 | ZFAND4//LYPLA1//CTSA//TOPORS//USP24//KLHL3//MYLIP//USP27X//ATXN3//RLIM//NPLOC4//USP28//USP46//UBE2I//BAG6//RNF128//KLHL15//USP45//CDC23//SQSTM1//USP3//UBE2W//TMF1//PMAIP1//UBXN4//DNAJB9//DERL2//JKAMP//WFS1//EDEM1//TGFB1I1//FBXL5//FBXW7//SKP1//BAG5//RNF180//ANKIB1//LONRF3//LONRF1//SOCS5//VLDLR//BNIP3//DNAJB12//LDLR//NRG1//MDM4//WWP2//UBXN2B//RNF38//RNF145//ZNRF2//CD2AP//SPOPL//HECW2//RNF4//TBL1XR1//PLAA//CLOCK//VPS37A//TSG101//RAB12//GJA1//NSF//SOX17//TNF//SH3D19//ADAM9//FMR1//EFNA1//EPHA4//MSN//PTEN// |
| GO:0061614 | pri-miRNA transcription by RNA polymerase II | Biological process | 9 | 38 | 934 | 17653 | 4.47641722078215 | 0.000126933465182385 | 0.0025779310060915 | 3.89642386389415 | 0.00963597430406852 | NFIB//JUN//STAT3//TEAD1//TGFB2//ETS1//PPARG//FOSL1//KLF4// |
| GO:0001763 | morphogenesis of a branching structure | Biological process | 24 | 198 | 934 | 17653 | 2.29096100188177 | 0.000130618922028178 | 0.00264483763376218 | 3.88399390470591 | 0.0256959314775161 | EDN1//PPP3R1//TGFBR2//ACVR1//NPNT//LHX1//PKD2//TCF21//CLIC4//MET//SLIT2//EPHA7//RDH10//FOXF1//EPHA2//LRP6//NOG//AR//ESR1//NTN4//GRB2//SULF1//BTBD7//TNF// |
| GO:2000679 | positive regulation of transcription regulatory region DNA binding | Biological process | 7 | 23 | 934 | 17653 | 5.75230425472489 | 0.000131753576438222 | 0.00265984906701999 | 3.88023758707326 | 0.00749464668094218 | FOXC1//KLF4//IGF1//NEUROD1//POU4F1//HMGA2//HAND2// |
| GO:0010975 | regulation of neuron projection development | Biological process | 43 | 450 | 934 | 17653 | 1.80604092315013 | 0.000132393557077223 | 0.00266481436462279 | 3.87813314929129 | 0.0460385438972163 | STMN2//NPTN//NCK1//NME1//SERPINI1//TWF1//ZNF804A//LPAR1//EPHA4//MYLIP//PTPRG//SPOCK1//TSC1//BAG5//DPYSL2//RAB21//WNT7A//GOLGA4//NRG1//EPHA7//ULK2//EFNA5//LPAR3//KLF4//RGMA//PTEN//FMR1//NSMF//HECW2//FXR1//BMPR2//SEMA4B//EFNA1//POU3F2//SLIT2//CAPRIN2//CPEB3//IL2//C21ORF91//LRP8//VLDLR//PLAA//SNAP25// |
| GO:0051270 | regulation of cellular component movement | Biological process | 79 | 985 | 934 | 17653 | 1.51587191165121 | 0.00013499991343449 | 0.00269710207368719 | 3.869666509986 | 0.0845824411134904 | MIA3//CCL8//SLIT2//EDN1//ETS1//KDR//BMPR2//WNT7A//DUSP10//PFN2//PTPRG//JUN//TGFB2//TGFBR2//DOCK5//PPM1F//CMKLR1//C5ORF30//ADAM10//CRK//IGF1//NRG1//RHOC//RND3//LAMA3//NCK1//MINK1//SLK//SEMA4B//ZNF268//DRD1//S1PR1//F3//FOXF1//CD274//ITGA6//ITGA5//ITGAV//NTF3//MAPK1//PTP4A1//ACVR1//DLC1//SULF1//TMEFF2//CLIC4//NF2//PTEN//RAP2C//LDLRAD4//RECK//NOG//GAB1//EFNA1//EPHA2//PPARG//ADAM9//VEGFB//LPAR1//TNF//MPP1//SPRED1//MEOX2//KLF4//DSC2//NEFH//CDK6//EREG//MEMO1//MSN//WASL//LRCH1//PTGER4//CCL2//CXCL14//MET//STAT3//NSMF//PLAA// |
| GO:0007507 | heart development | Biological process | 50 | 550 | 934 | 17653 | 1.71822075141133 | 0.000135097228279042 | 0.00269710207368719 | 3.86935356108504 | 0.0535331905781585 | GJA1//DLL1//PKD2//SOX17//TGFBR2//HAND1//HAND2//DLC1//TGFB2//ACVR1//ZFPM2//GATA6//RARB//BMPR2//NOG//JUN//SOX11//NRG1//EFNA1//SOX4//ERG//FOXF1//TMEM100//SOS1//POU4F1//S1PR1//LRP6//MEF2D//FOXC1//GJC1//PTEN//ACTC1//TSC1//EGLN1//FHL2//JARID2//REST//VEGFB//SPRED1//MAP2K4//EDN1//IGF1//MAPK1//OSR1//CRKL//GNAQ//PPARG//PPP3R1//ADIPOR2//KAT2B// |
| GO:0044255 | cellular lipid metabolic process | Biological process | 86 | 1094 | 934 | 17653 | 1.48577406840504 | 0.000135194085905657 | 0.00269710207368719 | 3.8690423062843 | 0.0920770877944325 | ACOX1//PTGES3//EDN1//RDH10//NAPEPLD//TTR//LRP8//ITGB8//ST6GALNAC3//ST6GALNAC6//ST8SIA3//ST3GAL5//ACSL1//PIGA//LYPLA1//ACOT7//UGT1A8//PRKAR2B//MSMO1//PER2//PRKAA1//ELOVL4//SCD5//NKX2-3//GATA6//PLAA//LCLAT1//DDHD1//CDS1//ABHD3//SLC44A1//SERINC3//PIKFYVE//PIK3CB//PTEN//PLEKHA1//PLEKHA3//MTMR9//PIK3R3//KDELC1//SGPP1//SPHK2//PPP2CA//NSMAF//ORMDL3//B3GALT2//CTSA//ST8SIA5//ZADH2//DGKE//ACBD5//B3GNT5//CNEP1R1//LDLR//FBXW7//ELOVL7//PON2//PPARG//ADIPOR2//PPM1L//SPTSSA//GAB1//GRB2//OSBPL5//UGT1A7//UGT1A9//UGT1A1//UGT1A3//QKI//KLF4//MID1IP1//NR4A3//DGKH//PLEK//EREG//ESR1//EFR3A//NRG1//MET//ZFYVE21//CHRM5//IDH1//TBL1XR1//TNF//CCDC71L//FAM135A// |
| GO:0045860 | positive regulation of protein kinase activity | Biological process | 48 | 522 | 934 | 17653 | 1.73797041522065 | 0.000139665309765447 | 0.00277810732336388 | 3.85491145106775 | 0.0513918629550321 | GADD45A//MINK1//TNF//MAP3K2//CRK//CRKL//MAP3K5//TAOK3//LAMTOR3//ADAM9//DUSP5//LPAR1//LPAR3//NRG1//IGF1//NTF3//PIK3CB//PRKAA1//MAPK1//MAP2K4//EREG//FBXW7//EFNA5//COPS8//MDFIC//PKD2//PAK6//TGFB2//TGFBR2//SLK//ADCY3//PRKACB//PRKAR2B//EDN1//FZD10//EPHA4//CCNY//MAPRE3//CCNC//CCND2//EFNA1//LRP8//ACSL1//PPP2CA//CALM2//CDKN1A//MMD//VLDLR// |
| GO:0051336 | regulation of hydrolase activity | Biological process | 97 | 1269 | 934 | 17653 | 1.44471358688407 | 0.000140924888615229 | 0.00279494141262403 | 3.85101229976703 | 0.103854389721627 | DLC1//EGLN3//F3//PMAIP1//PPARG//TNF//LPAR1//GNAQ//TGFB2//NPNT//ESR1//MTMR9//SPRED1//PPP1R37//PPP1R12A//PPP1R12C//PLEKHF2//URI1//PLEK//SERPINI1//SPOCK1//RECK//PCOLCE2//IQGAP2//FZD10//CPEB2//LRCH1//IPO5//TSC1//SLIT2//PLAA//CRY2//MASTL//CALM2//PPP1R15B//CNN3//PXK//DNAJB4//PFN2//FGD4//CRK//FGD5//EFNA5//EPHA4//EPHA5//RAB3GAP1//ARHGAP1//SBF2//FAM13A//CDC42EP3//GNB5//ARAP2//TAGAP//TBC1D20//ADRB1//DENND1B//S1PR1//DOCK9//RGS17//AGFG1//AGFG2//ITGA6//JUN//ARHGAP6//ERRFI1//ELMOD1//RGS3//CCL2//CCL8//SOS1//EVI5//TRIP10//ARHGAP12//USP6NL//GIT2//ARL6IP1//KLF4//FNIP1//BCL2L11//MAP3K5//REST//PPM1F//EPHA7//TMEM64//UBXN2B//PPP4R2//EGLN1//RHOC//EPHA2//TBC1D12//NTF3//TBC1D17//ASPH//EFNA1//STAT3//DYNLL2//ST18// |
| GO:0021543 | pallium development | Biological process | 21 | 163 | 934 | 17653 | 2.43502450046636 | 0.000143272150257544 | 0.00283318582512213 | 3.84383822108157 | 0.0224839400428266 | LRP8//CDK6//DRD1//EMX2//NEUROD1//PTEN//BTG2//SLC32A1//EPHA5//EZH1//NF2//NME1//TSC1//NR4A3//LHX6//POU3F2//ARX//SLIT2//TACC2//LRP6//BNIP3// |
| GO:0060537 | muscle tissue development | Biological process | 39 | 396 | 934 | 17653 | 1.86140581402894 | 0.000143709349785658 | 0.00283354615918485 | 3.84251497558029 | 0.0417558886509636 | NRG1//ZFPM2//TGFB2//POU4F1//NOG//S1PR1//ZBTB18//CFL2//MEOX2//TCF21//MTPN//EGR2//FOXN2//MEF2D//SOX11//BTG2//GJA1//DLL1//PRKAA1//SOX17//GJC1//PTEN//ACTC1//GATA6//TSC1//EGLN1//FOXC1//HAND1//RARB//FHL2//JARID2//TGFBR2//ACVR1//MAP2K4//EDN1//IGF1//OSR1//PKD2//NLN// |
| GO:0023052 | signaling | Biological process | 409 | 6724 | 934 | 17653 | 1.14965424756091 | 0.000144194796321311 | 0.00283485292884019 | 3.84105041208343 | 0.437901498929336 | RANBP9//PPM1L//SPRED1//DUSP5//EREG//RASGEF1A//GRB2//NRG1//IL2//JAK1//MAP3K5//MET//MINK1//TAOK3//MAPK1//CCL2//SOS1//TNF//LAMTOR3//GADD45A//MAP3K2//CRK//CRKL//ADAM9//LPAR1//LPAR3//IGF1//NTF3//PIK3CB//PRKAA1//MAP2K4//DUSP10//DUSP1//DUSP2//PPP2CA//RGS3//JUN//PMAIP1//BCL2L11//MOAP1//EPS15//NSF//CBFB//DRD1//P2RY1//THRA//PRKACB//SKP1//RPS6KA5//BAG6//ADAM10//ADRB1//EDN1//EFNA1//FJX1//GJA1//GJB2//AR//LHX1//PTHLH//HCN2//CCL8//BMP3//TFAP2C//TGFB2//KLF10//CXCL14//HMGA2//SOX17//S1PR1//GPR6//SPHK2//APCDD1//CTNND2//AMER2//CCNY//SOSTDC1//HBP1//DKK3//LRP6//PIAS4//STRN//TGFB1I1//WNT7A//TCF7L1//PPP3R1//FZD6//FAM13A//CNIH1//CDK6//DLC1//CDS1//CDC42EP3//GNB5//PTGES3//IQGAP2//ADCY3//RASSF8//ARAP2//TAGAP//CLCN6//DPYSL2//ELK3//STX2//EPS8//ERG//ESR1//EVI2A//LRCH1//CD2AP//APPL1//GLRA1//GLRB//RASSF3//RGMB//GRIA1//CD274//CNOT7//LRP12//HIVEP2//NR3C2//MPP1//PPP1R12A//NR4A2//RASD1//KCNK10//NDFIP2//PPARG//PPP1R12C//CC2D1A//PSD//ARRDC3//MIER1//RARB//RHEB//SMOC1//SMOC2//BCL11B//SLC20A1//RASL11B//SPOCK1//STAT3//VLDLR//ZNF217//LRP8//DEK//PDCD1LG2//CAMLG//NSMAF//PPFIA1//RHPN2//OR6A2//ARRDC4//TRIP10//PLAA//ARHGAP12//CLOCK//FEZ2//CD69//ULK2//KDR//PTPRG//AKAP11//DGKH//DCX//PIKFYVE//DSTYK//LATS2//MKNK2//ARHGEF4//TREM1//PRKAR2B//ARHGEF28//STK33//MASTL//DGKE//SQSTM1//RPS6KA4//STK17B//PALM2//BMPR2//FBXW7//ERRFI1//TSG101//SOCS5//CDKN1A//E2F1//CNOT6L//MDM4//CNOT6//RBL2//SOX4//BTG2//WFS1//PPP1R15B//IRF9//EFNA5//NCK1//GAB1//COL1A2//TGFBR2//ACVR1//ZFYVE9//PDE10A//RAPGEF4//FZD10//CHRM2//CHRM5//FGD4//CMKLR1//GPR180//FRZB//GNAQ//RHOC//GPRC5C//PTGER4//SOS2//CALM2//FMR1//FOXC1//DLL1//EPN1//TMEM100//KAT2B//TMEM17//DZIP1//FOXF1//ITGA6//ITGA5//ITGAV//ITGB8//PLEK//OSR1//NKIRAS1//SNIP1//COPS8//MDFIC//PKD2//STAT4//NEUROD1//RGL1//DOCK9//RAB38//RND3//ARHGAP1//RALGPS2//DOCK5//USP28//G3BP2//ARHGAP6//PAK6//GJC1//KIF5A//ATXN3//NOVA1//SLC12A5//SLC6A1//SNAP25//SLC32A1//PTEN//TRPC3//MARCKS//PER2//GIT2//PIK3R3//FLRT1//ESRP1//OTX2//BNIP3//TOPORS//EPHA2//NR5A2//ADIPOR2//CRY2//YWHAG//TOB1//EPHA4//EPHA5//EPHA7//RGS17//GDF11//IFNA1//INHBB//OGN//OSM//TTR//VEGFB//F3//PMEPA1//LDLRAD4//PLEKHA1//KLF4//SOX11//ZEB1//MMP2//ASPN//SCN1A//DENND1B//TNFRSF21//SLK//SULF1//SHISA2//RGMA//NOG//NPNT//BAMBI//PRDM16//GATA6//CTDSPL2//FHL2//RNF4//NR4A3//CREBRF//SERP1//DERL2//FNIP1//TSC1//RRAGD//AGFG1//AGFG2//USP46//TMF1//RAB12//RAB21//RAB30//RAB9B//DNAJC27//RAB23//RAB22A//RAB28//RAP2C//RAP1B//UBA5//ST18//ACSL1//IRF1//FGD5//CPEB4//SLC17A7//SLC17A6//TEAD1//NF2//LMO3//SLIT2//GSTA2//MSN//SYBU//DNAJB9//EDEM1//WASL//BRWD1//SESN3//NPLOC4//FAM20C//ZFAND6//UBE2I//ZMYND11//PGRMC2//HIPK3//AIDA//PEX5L//TMEM64//NPTN//WWP2//YTHDF2//REST//ARID5B//CPEB3//EGR2//NSMF//KCNJ10//RAB3GAP1//SYT6//PXK//OTUD3//TSHZ3//VANGL1//SLC24A2//NTN4//TCF21//CEBPB//HAND2//FIBIN//SEMA4B//POU4F1//ZMAT3//DSC2//KCNJ3//CAPRIN2//TBL1XR1//FASTK//SGPP1//ITM2B//PPM1F//FXR1//LYPD6//PAIP2//DYNLL2//RBBP7//RBBP8//BAG5//SERINC3//ASPH//SESN1//PFN2//PHF14//MYB//URI1//TOB2// |
| GO:0003006 | developmental process involved in reproduction | Biological process | 58 | 669 | 934 | 17653 | 1.63860215157015 | 0.000147250808357344 | 0.00288654265774121 | 3.83194231240388 | 0.0620985010706638 | CEBPB//FOXC1//INHBB//DMRTA1//IMMP2L//KMT2B//ESR1//EREG//PLEKHA1//YTHDF2//TBC1D20//AGFG1//TMF1//PPARG//PKD2//BMPR2//DZIP1//RNF2//ACVR1//TDRD5//QKI//ZFPM2//TCF21//AR//WNT7A//OSR1//RDH10//BCL2L11//RNF38//GATA6//TFAP2C//TGFB2//ARID5B//IDH1//LHX8//SYCP3//NEUROG1//LRP6//LHX1//LHFPL2//GJA1//GJB2//FOXF2//IGF1//NOG//ZNF568//GRB2//ITGB8//FOSL1//SULF1//HAND1//EGLN1//MAPK1//VASH2//PTEN//SLIT2//ARID4B//ARID4A// |
| GO:0001655 | urogenital system development | Biological process | 33 | 316 | 934 | 17653 | 1.97377890114656 | 0.000155279085006363 | 0.00303512269334692 | 3.80888703672491 | 0.0353319057815846 | GDF11//RDH10//OSR1//FOXC1//NPNT//LHX1//RARB//TCF21//SLIT2//PKD2//BCL2L11//SULF1//SOX11//TFAP2A//TGFB2//TSC1//WFS1//BAG6//ARID5B//AR//NOG//ESR1//PTEN//SOX4//EMX2//FOXF1//ITGA6//SOX17//DLL1//KLHL3//EPHA4//EPHA7//NFIA// |
| GO:0001503 | ossification | Biological process | 37 | 371 | 934 | 17653 | 1.88494837149437 | 0.000163943743638954 | 0.00319524938971252 | 3.7853051520652 | 0.0396145610278373 | EPHA2//FHL2//GJA1//MEF2D//BMP3//CBFB//NOG//MMP2//FOXC1//PTHLH//EGR2//LRP6//MAPK1//PTGER4//ACVR1//ASPN//KLF10//PKDCC//S1PR1//OSR1//FAM20C//BMPR2//TFAP2A//IGF1//TMEM64//SMOC1//TOB1//CDK6//TNF//HAND2//CEBPB//NPNT//SOX11//TOB2//TGFB2//THRA//RUNX3// |
| GO:0002064 | epithelial cell development | Biological process | 24 | 201 | 934 | 17653 | 2.25676755409249 | 0.000164881781165264 | 0.00320429737362265 | 3.78282732965881 | 0.0256959314775161 | CLIC4//MET//RARB//GJA1//CDK6//DLL1//PALLD//BCL11B//AR//FRMD6//WNT7A//CDKN1A//MSN//RAP1B//TMOD1//TBC1D20//EPHA2//RAP2C//ARID4B//ARID4A//TNF//RHEB//CLOCK//ESR1// |
| GO:0032535 | regulation of cellular component size | Biological process | 36 | 358 | 934 | 17653 | 1.90060172502482 | 0.000169873941621118 | 0.00329185520682986 | 3.76987323606984 | 0.0385438972162741 | EPS8//TWF1//CLCN6//SLC12A5//MTPN//MSN//DPYSL2//RAB21//EPHA4//PFN2//LIMA1//CFL2//PLEK//SLIT2//CDC42EP3//GRB2//NCK1//WNT7A//IQGAP2//ARFIP1//GOLGA4//NRG1//PTEN//TSC1//EDN1//PRR16//EPHA7//ULK2//EFNA5//LPAR3//BMPR2//SEMA4B//WASL//TMOD3//TMOD1//RAB22A// |
| GO:2000677 | regulation of transcription regulatory region DNA binding | Biological process | 10 | 48 | 934 | 17653 | 3.9375892219843 | 0.000170657059752468 | 0.00329758198601697 | 3.76787574109104 | 0.0107066381156317 | HAND1//FOXC1//KLF4//SOX11//PER2//IGF1//NEUROD1//POU4F1//HMGA2//HAND2// |
| GO:0022612 | gland morphogenesis | Biological process | 17 | 119 | 934 | 17653 | 2.70006118078923 | 0.000172739561116036 | 0.00332831239836966 | 3.76260818816033 | 0.0182012847965739 | TGFB2//TGFBR2//EPHA2//LRP6//NOG//AR//ESR1//SOSTDC1//NFIB//TNF//NTN4//SULF1//BTBD7//CEBPB//FBXW7//NKX2-3//MSN// |
| GO:0006688 | glycosphingolipid biosynthetic process | Biological process | 7 | 24 | 934 | 17653 | 5.51262491077802 | 0.000177559738360072 | 0.00341146735945786 | 3.75065550359295 | 0.00749464668094218 | ST6GALNAC3//ST6GALNAC6//ST8SIA3//ST3GAL5//B3GALT2//PRKAA1//ST8SIA5// |
| GO:0061448 | connective tissue development | Biological process | 27 | 240 | 934 | 17653 | 2.12629817987152 | 0.000181022031780643 | 0.00346813597997872 | 3.74226856491828 | 0.0289079229122056 | THRA//WNT7A//OSR1//MEF2D//NFIB//HMGA2//RUNX3//SULF1//BMPR2//RARB//TGFBR2//MAF//PTHLH//PKDCC//EDN1//HOXA3//ITGB8//BMP3//ZEB1//NOG//LRP6//HAND1//HAND2//ARID5B//ARRDC3//TBL1XR1//FRZB// |
| GO:0006996 | organelle organization | Biological process | 251 | 3888 | 934 | 17653 | 1.22016653668896 | 0.000185636294332979 | 0.00353993358592764 | 3.73133710954196 | 0.268736616702355 | KIF23//UBXN2B//TRAPPC8//RAB23//ATG16L1//ULK2//WASL//TACC2//DYNC1LI2//ATXN3//NEFH//MAP7D1//MIEF1//BNIP3//SQSTM1//COPS8//PTGES3//CCNE2//CLIP1//TBC1D20//AGFG1//TMF1//JUN//PMAIP1//BCL2L11//MOAP1//NAP1L3//ANP32E//EPS8//RHOC//RND3//MARCKS//MYO1B//LIMA1//TWF1//CFL2//CORO2B//DLC1//FGD4//FGD5//PALLD//FLII//NF2//PFN2//PAK6//TRIP10//WASF3//SH3D19//DPYSL2//MSN//BRWD1//CCL2//CCDC6//MAN2A1//STX6//SYT6//STX2//SNAP25//ZFAND6//HMG20A//ZMYND11//ATAD2//H2AFZ//JARID2//RSBN1//RBL2//BAG6//BRD3//HMGA2//TCF7L1//NFE2//SMARCD2//ESR1//HMGB3//MYB//DEK//KAT2B//BAHD1//KMT2B//DPY30//MTX3//IMMP2L//REEP3//NUP35//PIKFYVE//RAB38//CHCHD10//PALM2//CD2AP//TMOD3//NCK1//TMOD1//ARHGAP12//STMN2//RANBP9//SLAIN2//MID1IP1//GOLGA8A//RAB30//TMED9//NPLOC4//BAG5//USP6NL//TMEM135//RAB22A//AKTIP//FAM160A2//DYNLL2//NUP43//CENPQ//FBXW7//RAD21//CENPO//CNEP1R1//CDC23//PPP2CA//CDC14A//GADD45A//PPP1R12A//HAUS8//MYBL1//SYCP3//EREG//MASTL//ARHGAP6//PTPDC1//TMEM17//DZIP1//GMNC//C5ORF30//CEP41//CNOT6L//PAN3//CNOT6//CHMP3//EPS15//SNX5//SNX2//PEX5L//RPS6KA4//RPS6KA5//EPC2//CLOCK//ARID4B//MIER1//ARID4A//RBBP7//TBL1XR1//BRMS1L//PPM1F//ARID5B//USP3//HECW2//ACTC1//FAM118B//ETS1//TDRD5//PLEK//SLIT2//CDC42EP3//GRB2//TNF//FRMD6//CNN3//MYLIP//MET//SLK//TBC1D12//EVI5//TBC1D17//S1PR1//GAB1//MINK1//NEUROG1//DCP2//MAPK1//CCT6A//KIF13A//FZD10//CRK//EFNA5//EPHA5//TSC1//FMR1//NEFM//DDX6//CNOT7//G3BP2//IQGAP2//ARFIP1//RAB3GAP1//PRDM16//PRKAA1//RNF2//SKP1//VPS37A//TSG101//PCGF5//APOO//TADA1//DR1//DRD1//CEP97//MTBP//EDN1//IGF1//MDM1//STAT3//CNIH1//SEC23A//GRIA1//NSF//SAR1B//PPP6C//MCFD2//PKD2//MZT1//PTGER4//CLIC4//LPAR1//PPM1E//TMEFF2//PPFIA1//DNMT3B//KDM2A//EZH1//PER2//REST//RTN3//ARL6IP1//MIA3//KDR//DDHD1//RNF4//DUSP1//FRY//PTEN//SLC17A7//NEDD1//PRKAR2B//YWHAG//E2F1//PPP3R1//CALCOCO2//FYCO1//FEZ2//NRG1//ELMOD1//SNIP1//NTF3//MTPN// |
| GO:1901564 | organonitrogen compound metabolic process | Biological process | 429 | 7112 | 934 | 17653 | 1.14008488834506 | 0.000185816416236036 | 0.00353993358592764 | 3.73091592023591 | 0.4593147751606 | CDKN1A//GADD45A//PTEN//CCNE2//MTRR//RANBP9//PPM1L//SPRED1//DUSP5//EREG//RASGEF1A//GRB2//NRG1//IL2//JAK1//MAP3K5//MET//MINK1//TAOK3//MAPK1//CCL2//SOS1//TNF//LAMTOR3//MAP3K2//CRK//CRKL//ADAM9//LPAR1//LPAR3//IGF1//NTF3//PIK3CB//PRKAA1//MAP2K4//DUSP10//DUSP1//DUSP2//PPP2CA//RGS3//TOPORS//WWP2//HECTD2//RNF145//ZNRF2//FBXL5//RNF180//MYLIP//RLIM//ANKIB1//UBE2W//FBXW7//HECW2//SKP1//THOP1//LONRF3//FBXO30//CBFB//UBE3B//LONRF1//ZFAND4//COPS8//SECISBP2L//ITGB8//ST6GALNAC3//ST6GALNAC6//ST8SIA3//ST3GAL5//DRD1//FNIP1//FAM129A//LRP6//PPP1R15B//SLIT2//EFNA1//EFNA5//EPHA7//FMR1//NPTN//KDR//P2RY1//AKTIP//SQSTM1//CCND2//LYPLA1//RPL15//F3//ERRFI1//TSG101//SOCS5//TRIM3//KLHL2//RNF38//MYCBP2//FBXO33//KLHL3//SPOPL//MDM4//DCAF8//DCAF6//FEM1C//KLHL14//MARCH4//RNF4//MARCH7//UBE2Z//RNF128//KLHL15//KBTBD8//ARRDC4//HS3ST5//XYLT2//HS2ST1//HS3ST3A1//HS3ST1//GNS//NUP35//NUP43//CHCHD10//COX8C//COX7A2L//GSTA2//IDH1//TTR//HPRT1//ADCY3//IMPDH1//NME1//PDE10A//CMPK1//DCTPP1//CPEB2//CPEB3//CPEB1//SOX11//CPEB4//MTPN//DDX6//MKNK2//ACO1//TSC1//PPP4R2//SERP1//UEVLD//RIMKLB//UBE2I//IMMP2L//HIPK3//ADAM10//CDK6//CDK8//FASTK//PDIK1L//ERG//CDK19//MORC3//MMD//PAN3//DSTYK//LATS2//CDK17//PRKACB//FAM20C//CCL8//TGFB2//TGFBR2//RUNX3//CCNC//RPS6KA4//ACVR1//RPS6KA5//STK17B//PPM1E//FLRT1//GNAQ//NCK1//NF2//ASPN//DUS2//YWHAG//CAMK2N2//PPM1F//PTPN21//PPP1R12A//CTDSPL2//PPP3R1//PPP6C//PTPN4//KAT2B//CLOCK//NAA30//PCMTD1//BTG1//BTG2//ASPH//FUT9//ST8SIA5//MAN1A1//MAN2A1//B3GNT5//B3GALT2//CCDC126//GFPT2//EDEM1//VEGFB//NTNG1//RECK//PTAR1//PIGA//MMP2//CTSA//NLN//LRP8//NPEPL1//ADAM12//ADAM18//LMLN//ZFYVE9//USP24//USP27X//ATXN3//NPLOC4//USP28//USP46//BAG6//USP45//CDC23//USP3//SLC6A14//SLC6A6//GLUD2//LGSN//SLC25A32//BCAT2//SERINC3//PSAT1//ABHD3//SLC44A1//CDS1//SGPP1//SPHK2//NSMAF//ORMDL3//UGT1A4//UGT1A1//DLC1//EGLN3//PMAIP1//PPARG//DLL1//PTGER4//MDFIC//EPHA2//PTHLH//TMF1//CALM2//GDF11//INHBB//BMP3//BMPR2//LDLR//SERPINI1//SPOCK1//PCOLCE2//ACOT7//PPCS//PRR16//SOX4//FXR1//GNPTG//ST3GAL3//EPC2//ARID4B//MIER1//ARID4A//RBBP7//TBL1XR1//BRMS1L//ARID5B//DMWD//ESR1//OTUD3//AR//WDR20//JOSD1//EGR2//PIAS4//TOB1//CNOT7//NANOS1//CAPRIN2//VKORC1L1//CEP41//STK33//MASTL//OSR1//EPHA4//EPHA5//PKDCC//OGN//CHST1//TMX3//ZDHHC1//ST6GAL2//MCFD2//CRY2//ZDHHC5//CRTAP//EGLN1//WFS1//PAK6//SLK//SPTSSA//ELOVL4//ELOVL7//SULF1//FOXC1//DSEL//SLC35D1//CHST7//UBXN4//DNAJB9//DERL2//JKAMP//TGFB1I1//PKD2//ADRB1//JARID2//PER2//BAG5//NDFIP2//JUN//RAP2C//EDN1//WNT7A//KLF4//PPP1R37//URI1//VLDLR//OSM//PFN2//IFNA1//HMG20A//PRKAR2B//ESCO2//PRDM16//CNOT6//STAT3//CNEP1R1//NSMF//PTPDC1//PTPRG//PTP4A1//CDC14A//RNF2//BNIP3//HMGA2//OSBPL5//PCGF5//DNAJB12//ZNF287//NR4A2//ITM2B//ARL6IP1//UBXN2B//CD2AP//PLAA//VPS37A//BCL2L11//REST//ITGAV//FZD10//AIDA//TWF1//CKAP4//RAB38//MIA3//ANO8//TADA1//DR1//RAB12//GATA6//IRF1//CEBPB//GJA1//NSF//SOX17//IPO5//CCNY//MAPRE3//PAIP2//MPP1//RAB23//ENPP4//ZMYND11//ULK2//ITGA5//SH3D19//KLF2//ARRDC3//DPY30//KMT2B//DNMT3B//MYB//RNF141//PMEPA1//LDLRAD4//NOG//CNOT6L//YTHDF2//QKI//PFKP//ATG16L1//NAPEPLD//LMO3//NPNT//DNAJC27//HAND2//KDM2A//EZH1//UBA5//ACSL1//GLRX//RAB3GAP1//MSN//FRY//UNK//ST18// |
| GO:0060412 | ventricular septum morphogenesis | Biological process | 9 | 40 | 934 | 17653 | 4.25259635974304 | 0.000193588789200019 | 0.00367764320606665 | 3.71311979645709 | 0.00963597430406852 | TGFB2//TGFBR2//NOG//ZFPM2//EGLN1//BMPR2//SOX4//SOX11//ACVR1// |
| GO:0072001 | renal system development | Biological process | 30 | 280 | 934 | 17653 | 2.02504588559192 | 0.000194717247205351 | 0.00368133872601984 | 3.71059557886327 | 0.0321199143468951 | GDF11//RDH10//OSR1//FOXC1//NPNT//LHX1//RARB//TCF21//SLIT2//PKD2//BCL2L11//SULF1//SOX11//TFAP2A//TGFB2//TSC1//WFS1//BAG6//ARID5B//NOG//SOX4//DLL1//KLHL3//EPHA4//EPHA7//FOXF1//NFIA//EMX2//ITGA6//SOX17// |
| GO:0007015 | actin filament organization | Biological process | 38 | 388 | 934 | 17653 | 1.85107287136581 | 0.000194871989341284 | 0.00368133872601984 | 3.71025058134803 | 0.0406852248394004 | EPS8//RHOC//RND3//MARCKS//MYO1B//LIMA1//TWF1//CFL2//WASL//WASF3//ARHGAP6//ACTC1//PFN2//PLEK//SLIT2//CDC42EP3//GRB2//NCK1//IQGAP2//ARFIP1//PTGER4//TSC1//LPAR1//PPM1E//NF2//PPM1F//DLC1//S1PR1//TMEFF2//MET//PPFIA1//TMOD3//TMOD1//FRY//MTPN//PALM2//CD2AP//ARHGAP12// |
| GO:0110053 | regulation of actin filament organization | Biological process | 28 | 254 | 934 | 17653 | 2.08351177730193 | 0.000195573189308655 | 0.00368429381419062 | 3.70869068192765 | 0.0299785867237687 | EPS8//TWF1//PFN2//LIMA1//CFL2//PLEK//SLIT2//CDC42EP3//GRB2//NCK1//IQGAP2//ARFIP1//WASL//PTGER4//TSC1//LPAR1//PPM1E//NF2//PPM1F//DLC1//S1PR1//TMEFF2//ARHGAP6//MET//PPFIA1//TMOD3//TMOD1//MTPN// |
| GO:0016358 | dendrite development | Biological process | 25 | 216 | 934 | 17653 | 2.18754956776905 | 0.000199191550991926 | 0.00374203460932888 | 3.70072908677278 | 0.0267665952890792 | HPRT1//MINK1//VLDLR//KLF7//FMR1//NSMF//HECW2//FXR1//EPHA4//RAB21//CAPRIN2//SLC12A5//CTNND2//PTEN//WNT7A//WASL//CPEB3//LPAR1//NRG1//IL2//C21ORF91//EFNA1//LRP8//FLRT1//STRN// |
| GO:0030324 | lung development | Biological process | 21 | 167 | 934 | 17653 | 2.37670056033543 | 0.000201893272951388 | 0.00376395335273653 | 3.69487815143161 | 0.0224839400428266 | FOXF1//CIC//MAN2A1//ERRFI1//BMPR2//TCF21//PKDCC//PHF14//MAPK1//SOX11//NOG//GATA6//RDH10//TGFBR2//NFIB//PPP3R1//KLF2//THRA//TNF//ZFPM2//BAG6// |
| GO:0071158 | positive regulation of cell cycle arrest | Biological process | 14 | 88 | 934 | 17653 | 3.00688631496983 | 0.00020247454373698 | 0.00376395335273653 | 3.6936295710148 | 0.0149892933618844 | CDKN1A//GADD45A//E2F1//CNOT6L//CNOT7//MDM4//CNOT6//RBL2//SOX4//BTG2//GATA6//PKD2//POU4F1//HMGA2// |
| GO:0110020 | regulation of actomyosin structure organization | Biological process | 14 | 88 | 934 | 17653 | 3.00688631496983 | 0.00020247454373698 | 0.00376395335273653 | 3.6936295710148 | 0.0149892933618844 | PTGER4//TSC1//LPAR1//PPM1E//NF2//PFN2//PPM1F//DLC1//S1PR1//TMEFF2//ARHGAP6//MET//PPFIA1//EDN1// |
| GO:0065008 | regulation of biological quality | Biological process | 244 | 3770 | 934 | 17653 | 1.22326379225146 | 0.000202584506934215 | 0.00376395335273653 | 3.69339377126726 | 0.261241970021413 | PTGES3//CCNE2//SNAP25//SLC17A7//GLRA1//GNAQ//ADRB1//DRD1//ARRDC3//STAT3//NKX2-3//BCL2L11//SOS1//PMAIP1//MOAP1//NANOS1//CALM2//ARL6IP1//RDH10//F3//AR//EDN1//BMPR2//ADCY3//PRKACB//PRKAR2B//WFS1//GJA1//PKD2//THRA//EPS8//TWF1//COL1A2//ENPP4//DOCK9//ZFPM2//GATA6//IFNA1//IRF1//NFE2//DNAJC16//GLRX//NHLRC2//TMX3//SLC25A32//SLC12A5//SLC24A2//PIK3CB//SLC24A3//STIM2//CCL8//FBXL5//ACO1//EGLN1//SKP1//CYBRD1//CLCN6//CLIC4//LPAR1//ESR1//LPAR3//NPTN//GPR6//IL2//ITGAV//LRP6//P2RY1//PTGER4//G3BP2//SLC32A1//WNT7A//PPFIA1//NME1//FLI1//RAPGEF4//MARCKS//NEUROD1//TNF//PER2//CLOCK//PLEKHA1//UGT1A1//DLL1//PPARG//DLC1//CDC42EP3//WASF3//PALM2//FGD4//CRK//FGD5//BAMBI//KDR//RHOC//RND3//MSN//BRWD1//CCL2//WASL//MTPN//PLSCR4//TRPC3//PLEK//BCAT2//FBXW7//BNIP3//SLC41A1//PLAA//INHBB//CDKN1A//CRTAP//EFNA1//EPHA4//OTUD3//MORC3//IGF1//USP27X//MDM4//PFN2//CDC37L1//PTEN//SOX17//MTMR9//SOX4//TSC1//BAG6//CCT6A//SOX11//SCN1A//DGKH//MAPK1//FZD6//DGKE//KCNK10//DPYSL2//RAB21//LIMA1//CFL2//SLIT2//GRB2//NCK1//STX2//ASPH//NF2//CTSA//ELMOD1//USP28//RNF128//USP3//MYLIP//SERP1//DCP2//TMF1//DKK3//REST//IQGAP2//ARFIP1//SYBU//FOXC1//WWP2//GLRB//PXK//HCN2//SLC44A1//RNF180//TTR//UGT1A8//UGT1A7//UGT1A9//UGT1A3//NR5A2//TRERF1//CRY2//SESN3//PRKAA1//SLC2A4//ADIPOR2//LDLR//KLF2//FMR1//YTHDF2//FXR1//S1PR1//KLF4//CDK6//ETS1//GOLGA4//NRG1//TMEM64//PRR16//TBC1D20//OSM//KLHL3//CPEB3//YWHAG//EGR2//NSMF//KCNJ10//RAB3GAP1//E2F1//EPHA7//ULK2//EFNA5//SYT6//TMOD3//ARID4A//SEMA4B//SLC17A6//DZIP1//SLC4A4//TOPORS//TMOD1//JUN//SLITRK3//FLRT1//STEAP2//TRPV6//ACOX1//GRIA1//TSHZ3//CAPRIN2//LRP8//CNOT6L//QKI//EPHA5//GRHL1//MET//SQSTM1//XKR8//GJC1//DSC2//KCNJ3//ORMDL3//RAB38//NR4A3//RAB22A//FAM155A//PAIP2//DYNLL2//PPP3R1//ZNF804A//DOCK5//NEUROG1//MYB// |
| GO:0060998 | regulation of dendritic spine development | Biological process | 12 | 68 | 934 | 17653 | 3.33536969391611 | 0.000210184981597818 | 0.00388941599449926 | 3.67739831895579 | 0.012847965738758 | LPAR1//CPEB3//FMR1//NRG1//IL2//C21ORF91//EPHA4//EFNA1//PTEN//CAPRIN2//LRP8//FXR1// |
| GO:0051492 | regulation of stress fiber assembly | Biological process | 13 | 78 | 934 | 17653 | 3.15007137758744 | 0.000210487395236837 | 0.00388941599449926 | 3.67677390620832 | 0.0139186295503212 | LPAR1//PPM1E//NF2//PFN2//TSC1//PPM1F//DLC1//S1PR1//TMEFF2//ARHGAP6//MET//PPFIA1//PTGER4// |
| GO:0051603 | proteolysis involved in cellular protein catabolic process | Biological process | 58 | 679 | 934 | 17653 | 1.6144695720183 | 0.000217396546641276 | 0.00400613854205708 | 3.6627473589919 | 0.0620985010706638 | ZFAND4//TOPORS//USP24//KLHL3//MYLIP//USP27X//ATXN3//RLIM//NPLOC4//USP28//USP46//UBE2I//BAG6//RNF128//KLHL15//USP45//CDC23//SQSTM1//USP3//UBE2W//TMF1//PMAIP1//UBXN4//DNAJB9//DERL2//JKAMP//WFS1//EDEM1//TGFB1I1//FBXL5//FBXW7//SKP1//BAG5//RNF180//ANKIB1//LONRF3//LONRF1//SOCS5//DNAJB12//WWP2//UBXN2B//RNF38//RNF145//ZNRF2//CD2AP//SPOPL//HECW2//RNF4//TBL1XR1//PLAA//CLOCK//VPS37A//TSG101//FMR1//EFNA1//EPHA4//PTEN//CTSA// |
| GO:0030155 | regulation of cell adhesion | Biological process | 56 | 650 | 934 | 17653 | 1.62834458902981 | 0.000224000948732686 | 0.00411662613119336 | 3.6497501422529 | 0.0599571734475375 | MINK1//PIK3CB//TSC1//NF2//CDK6//ADAM10//MIA3//ATXN3//FOXF1//NPNT//ITGA6//ITGA5//SMOC2//PPM1F//SPOCK1//EFNA5//EPHA7//GRB2//CD274//PDCD1LG2//EFNA1//EPHA2//TGFB2//ADAM9//KLF4//TNF//IL2//NCK1//IGF1//CEBPB//TNFRSF21//MARCH7//IRF1//DUSP10//MYB//SOCS5//ITGAV//CCL2//TGFBR2//SLK//KDR//DLC1//ARHGAP6//PTEN//YTHDF2//CRK//CRKL//DOCK5//NR4A3//ETS1//S1PR1//DLL1//LAMA3//PPP1R12A//SERPINI1//PPP2CA// |
| GO:0016311 | dephosphorylation | Biological process | 43 | 461 | 934 | 17653 | 1.76294667118776 | 0.000225065439252862 | 0.00412497985275638 | 3.64769118955806 | 0.0460385438972163 | PTPN21//DUSP10//PPM1L//PPP4R2//DUSP1//DUSP2//DUSP5//PPP1R12A//CTDSPL2//PPP2CA//PPP3R1//PPP6C//PTEN//PTPN4//TGFB2//TNF//NPNT//MTMR9//SPRED1//PPP1R37//PPP1R12C//PLEKHF2//URI1//PLEK//PPM1F//CRY2//MASTL//CALM2//PPP1R15B//DLC1//CNEP1R1//NSMF//PTPDC1//PTPRG//PTP4A1//CDC14A//PPM1E//UBXN2B//TSC1//CHRM5//NCK1//SGPP1//HDHD2// |
| GO:0007178 | transmembrane receptor protein serine/threonine kinase signaling pathway | Biological process | 34 | 336 | 934 | 17653 | 1.91254333639237 | 0.000226568642521321 | 0.0041301448231043 | 3.64480019739547 | 0.0364025695931477 | COL1A2//JUN//TGFB2//TGFBR2//KLF10//ADAM9//ACVR1//ZFYVE9//GDF11//INHBB//BMP3//BMPR2//PMEPA1//LDLRAD4//DKK3//SOX11//ZEB1//TOB1//SOSTDC1//RGMB//TMEM100//RGMA//NOG//NPNT//TGFB1I1//BAMBI//ASPN//PRDM16//RASL11B//SULF1//GATA6//CTDSPL2//PALM2//PPM1L// |
| GO:1902903 | regulation of supramolecular fiber organization | Biological process | 34 | 336 | 934 | 17653 | 1.91254333639237 | 0.000226568642521321 | 0.0041301448231043 | 3.64480019739547 | 0.0364025695931477 | EPS8//TWF1//STMN2//MID1IP1//PFN2//LIMA1//CFL2//PLEK//SLIT2//CDC42EP3//GRB2//NCK1//MET//SLAIN2//CLIP1//IQGAP2//ARFIP1//WASL//PTGER4//TSC1//LPAR1//PPM1E//NF2//PPM1F//DLC1//S1PR1//TMEFF2//ARHGAP6//PPFIA1//TMOD3//TMOD1//EDN1//LDLR//MTPN// |
| GO:0006139 | nucleobase-containing compound metabolic process | Biological process | 366 | 5967 | 934 | 17653 | 1.15930228677426 | 0.00022918711704257 | 0.00416664643160995 | 3.63980979837381 | 0.39186295503212 | IGF1//NFIA//NFIB//RBBP7//RBBP8//RRM2//E2F1//NPAT//TSHZ1//HMG20A//KLF2//ZBTB18//CEBPB//ZNF268//TCERG1//WWP2//KLF12//OSR1//CRY2//GLIS3//ARX//DNMT3B//DR1//EDN1//EFNA1//CC2D1B//EN1//ESR1//FHL2//CPEB3//FOXF1//FOXC1//MYT1L//CIC//ZFPM2//DNAJB5//GATA6//CNOT7//HSBP1//AR//JARID2//JUN//ZNF568//MAF//MDM4//MYB//NR4A2//RLIM//KLF3//ZBTB7A//PIAS4//POU4F1//PPARG//CC2D1A//TSHZ3//ZBTB4//RARB//REST//RNF2//PRDM16//SOX17//SOX11//STAT3//ZEB1//TCF21//TFAP2A//TFAP2C//TGIF1//KLF10//TNF//UBE2I//VLDLR//WFS1//ZNF217//LRP8//BTG2//BHLHE41//TBL1XR1//NR4A3//HMGA2//ARID5B//BRMS1L//BHLHE40//RUNX3//URI1//PER2//SQSTM1//NOG//KLF4//HAND1//FNIP1//PHF14//USP3//DCP2//PPP2CA//RPL15//CNOT6L//PAN3//CNOT6//RBFOX3//FMR1//MBNL1//RBM25//SRSF2//ELAVL2//NOVA1//COPS8//PTGES3//CCNE2//NABP1//GTF2E1//DUS2//IRF9//KLF6//CREBRF//EGR2//ELK3//ERG//FOXJ3//FOXF2//FLI1//GRHL1//HIVEP2//IRF1//LHX1//MEF2D//MEOX2//MYBL1//NEUROD1//NHLH2//OTX2//PAX9//PBX3//POU3F2//RAD21//ARID4A//SHOX//BCL11B//BMPR2//SOX4//BTF3//TEF//TGFB1I1//THRA//TMF1//DEK//FOSL1//KLF7//CBFB//FUBP3//HAND2//CLOCK//POLR3G//PKD2//UGP2//GFPT2//NAGK//SLC35D1//NUP35//NUP43//CHCHD10//COX8C//COX7A2L//TTR//HPRT1//NME1//CMPK1//ADCY3//IMPDH1//PDE10A//DCTPP1//REV3L//GMNC//ESCO2//GADD45A//EPC2//TAOK3//UBE2W//USP28//USP45//ATXN3//KDM2A//MTRR//HMGB3//BAHD1//H2AFZ//KMT2B//DPY30//ARID4B//TOPORS//KHDRBS3//ZMYND11//TADA1//ZNF597//CTNND2//ZNF782//NKX2-3//ZNF800//E2F5//ZNF367//EZH1//ZBTB9//MYCBP2//FLII//LHX6//HBP1//ATAD2//SERTAD3//MDFIC//MNX1//HOXA3//HOXD1//FOXN2//ZBTB41//ZBTB34//LHX8//NEUROG1//NFE2//NPAS2//IER5//BRWD1//BNC2//ZNF532//CDCA7L//PRKAA1//TRERF1//LMO3//MAPK1//SLC2A4RG//PHTF2//ZNF630//ZNF287//ZNF512B//ZBTB26//MIER1//RBL2//RNF4//ATXN1//DMRTA1//SMARCD2//STAT4//ZNF2//ZNF3//ZNF131//ZNF655//BRD3//TCF7L1//PCGF5//LCOR//WASL//ZBTB47//ST18//HIPK3//NR5A2//NRBF2//MLLT6//RNF141//PAK6//SNAPC1//BTG1//SNIP1//RPS6KA4//RPS6KA5//CDKN1A//CRK//EMX2//ETS1//BOLA3//NR3C2//PLEK//ZNFX1//NKX2-4//RFX7//BDP1//CDK8//E2F3//TEAD1//KAT2B//CCNC//CMTR2//ZFC3H1//RTCA//PPP4R2//ESRP1//CPEB1//QKI//IDH1//G3BP2//NF2//EPHA2//PTHLH//EREG//DDX6//ACOT7//PPCS//GLRX//INTS8//RBM47//MAP3K2//MAPRE3//BAMBI//RGMB//LRP6//MAP3K5//WNT7A//ACVR1//DKK3//NRG1//RASD1//TSG101//BTAF1//PPM1F//TOB1//SOSTDC1//TMEM100//RGMA//BMP3//TGFB2//SULF1//CTDSPL2//CALM2//ADRB1//EGLN1//CMKLR1//CCT6A//EPHA5//FBXW7//UXS1//TDRD5//FZD6//FASTK//AHNAK//YTHDF2//FXR1//NPLOC4//MAP2K4//S1PR1//DLL1//IL2//ITGA6//MET//PPP1R12A//NCK1//OSM//P2RY1//PPP3R1//DCAF6//CAPRIN2//FAM20C//MPP1//RAB23//ENPP4//PTEN//MTPN//EGLN3//PFKP//REXO1//ZC3H12C//NANOS1//RAP2C//TOB2//KLHL15//DUSP1//GJA1//NPNT//DPYSL2// |
| GO:0007169 | transmembrane receptor protein tyrosine kinase signaling pathway | Biological process | 60 | 711 | 934 | 17653 | 1.59497284941136 | 0.000234887975228323 | 0.00425884015139182 | 3.62913921577096 | 0.0642398286937901 | EREG//FBXW7//ERRFI1//TSG101//SOCS5//EFNA5//NRG1//EPS8//EPS15//GAB1//GRB2//SOS1//CAMLG//RPS6KA5//APPL1//PIK3R3//SPRED1//FLRT1//ESRP1//MAPK1//F3//ITGA5//SULF1//KDR//FOXC1//OTX2//FAM20C//SHISA2//EPN1//IGF1//AR//DSTYK//NPTN//SESN3//NCK1//TSC1//PTEN//PLEKHA1//ARID5B//CRK//ITGAV//PIK3CB//BMPR2//VEGFB//MET//EFNA1//EPHA2//EPHA4//EPHA5//EPHA7//MMP2//ARHGEF28//WASL//STAT3//NOG//DLL1//PHF14//JAK1//NTF3//PTPRG// |
| GO:0035404 | histone-serine phosphorylation | Biological process | 5 | 12 | 934 | 17653 | 7.87517844396859 | 0.000237749914316625 | 0.00429920500139929 | 3.62387963090716 | 0.00535331905781585 | HMGA2//DRD1//RPS6KA4//RPS6KA5//PRKAA1// |
| GO:0008344 | adult locomotory behavior | Biological process | 13 | 79 | 934 | 17653 | 3.11019705635215 | 0.00023994603416736 | 0.00432734674419695 | 3.61988642369974 | 0.0139186295503212 | DRD1//EPHA4//GLRA1//GLRB//KCNJ10//SCN1A//EN1//EPS8//NR4A2//PBX3//TMOD1//TSC1//CCND2// |
| GO:0060411 | cardiac septum morphogenesis | Biological process | 12 | 69 | 934 | 17653 | 3.28703100269993 | 0.000242373450125942 | 0.00435949905106847 | 3.61551495503094 | 0.012847965738758 | ZFPM2//GATA6//RARB//BMPR2//TGFB2//TGFBR2//NOG//SOX4//ACVR1//EGLN1//SOX11//HAND1// |
| GO:0014706 | striated muscle tissue development | Biological process | 37 | 379 | 934 | 17653 | 1.84516054307232 | 0.000250618745393299 | 0.00449584767929677 | 3.60098644844002 | 0.0396145610278373 | NRG1//ZFPM2//TGFB2//POU4F1//NOG//S1PR1//ZBTB18//CFL2//MEOX2//TCF21//MTPN//EGR2//FOXN2//MEF2D//SOX11//BTG2//GJA1//DLL1//PRKAA1//SOX17//GJC1//PTEN//ACTC1//GATA6//TSC1//EGLN1//FOXC1//HAND1//RARB//FHL2//JARID2//TGFBR2//ACVR1//MAP2K4//EDN1//IGF1//NLN// |
| GO:0033554 | cellular response to stress | Biological process | 138 | 1962 | 934 | 17653 | 1.32938792081672 | 0.000256019401582978 | 0.00457830174639616 | 3.59172712187115 | 0.147751605995717 | E2F1//USP28//CLOCK//COPS8//RBBP8//NABP1//GADD45A//EPC2//TAOK3//UBE2W//BTG2//USP45//USP3//HMGA2//ATXN3//ESCO2//RAD21//KDM2A//PIAS4//TOPORS//CDKN1A//EGLN3//FMR1//NPAS2//PMAIP1//MAPK1//ZBTB4//ZMAT3//IMMP2L//MASTL//CNOT6L//CNOT7//MDM4//CNOT6//RBL2//SOX4//WFS1//PPP1R15B//OSR1//CRKL//MAP3K5//MINK1//PTGER4//MAP2K4//TNF//MAP3K2//MDFIC//BCL2L11//EPHA2//MOAP1//DSC2//INHBB//RRAGD//BMPR2//KLF10//PLEKHF2//FNIP1//PPP4R2//UBXN4//DNAJB9//DERL2//JKAMP//NPLOC4//BAG6//EDEM1//CREBRF//SERP1//PAK6//SLK//JUN//TGFB2//SESN3//SESN1//CPEB2//VKORC1L1//GJB2//NR4A2//CETN1//IER5//HSPA13//PKD2//CEBPB//FAM129A//NRBF2//TMX3//UBA5//KLF2//NCK1//CCL2//DNAJB12//PRKAA1//CPEB4//DCTPP1//REV3L//GRB2//FZD10//EDN1//EPHA4//HIPK3//DUSP10//AIDA//SPRED1//ZMYND11//WNT7A//KLF4//RGMA//PTEN//SKP1//EGLN1//ETS1//MYB//PLEKHA1//BNIP3//HSBP1//RBBP7//UBE2I//DNMT3B//PPARG//GATA6//CPEB1//SLC2A4//ERRFI1//KLHL15//CRK//SOCS5//SQSTM1//PTGES3//NUP35//NUP43//BAG5//HAND2//MET//SERINC3//TSC1//NR4A3//FBXW7//CDK6//DEK// |
| GO:0048870 | cell motility | Biological process | 117 | 1615 | 934 | 17653 | 1.36925703223925 | 0.000257245994918016 | 0.00457830174639616 | 3.58965137807591 | 0.125267665952891 | SEMA4B//ACVR1//DCX//GJA1//GPM6A//LRP12//NR4A2//SPOCK1//UNK//KDR//SLIT2//CCL2//CCL8//MIA3//HAND2//CD2AP//NCK1//EPHA4//P2RY1//TGFB2//FOXC1//EDN1//ETS1//BMPR2//WNT7A//NANOS1//DUSP10//PFN2//PTPRG//JUN//TGFBR2//DOCK5//PPM1F//CMKLR1//ARID5B//C5ORF30//ADAM10//CRK//IGF1//S1PR1//EFNA1//EPHA2//ERG//RASGEF1A//PALLD//BAMBI//GRB2//JAK1//RHOC//RND3//PIK3CB//PAK6//PTEN//BTG1//ADAM9//POU4F1//LHX6//POU3F2//ARX//NRG1//DRD1//EMX2//ADCY3//TMF1//LAMA3//MINK1//SLK//ZNF268//F3//FOXF1//CD274//ITGA6//ITGA5//ITGAV//NTF3//MAPK1//PTP4A1//DLC1//SULF1//TMEFF2//CLIC4//NF2//RAP2C//LDLRAD4//RECK//NOG//BCL11B//EPS8//GAB1//SOX17//PPARG//APCDD1//COL1A2//NKX2-3//MSN//TREM1//SOS1//TNF//LPAR1//CXCL14//VEGFB//MPP1//SPRED1//MEOX2//KLF4//LHX1//MCOLN2//CDK6//EREG//MEMO1//WASL//LRCH1//PTGER4//MET//STAT3//NSMF//PLAA// |
| GO:0051674 | localization of cell | Biological process | 117 | 1615 | 934 | 17653 | 1.36925703223925 | 0.000257245994918016 | 0.00457830174639616 | 3.58965137807591 | 0.125267665952891 | SEMA4B//ACVR1//DCX//GJA1//GPM6A//LRP12//NR4A2//SPOCK1//UNK//KDR//SLIT2//CCL2//CCL8//MIA3//HAND2//CD2AP//NCK1//EPHA4//P2RY1//TGFB2//FOXC1//EDN1//ETS1//BMPR2//WNT7A//NANOS1//DUSP10//PFN2//PTPRG//JUN//TGFBR2//DOCK5//PPM1F//CMKLR1//ARID5B//C5ORF30//ADAM10//CRK//IGF1//S1PR1//EFNA1//EPHA2//ERG//RASGEF1A//PALLD//BAMBI//GRB2//JAK1//RHOC//RND3//PIK3CB//PAK6//PTEN//BTG1//ADAM9//POU4F1//LHX6//POU3F2//ARX//NRG1//DRD1//EMX2//ADCY3//TMF1//LAMA3//MINK1//SLK//ZNF268//F3//FOXF1//CD274//ITGA6//ITGA5//ITGAV//NTF3//MAPK1//PTP4A1//DLC1//SULF1//TMEFF2//CLIC4//NF2//RAP2C//LDLRAD4//RECK//NOG//BCL11B//LHX1//EPS8//GAB1//SOX17//PPARG//APCDD1//COL1A2//NKX2-3//MSN//TREM1//SOS1//TNF//LPAR1//CXCL14//VEGFB//MPP1//SPRED1//MEOX2//KLF4//MCOLN2//CDK6//EREG//MEMO1//WASL//LRCH1//PTGER4//MET//STAT3//NSMF//PLAA// |
| GO:0032231 | regulation of actin filament bundle assembly | Biological process | 14 | 90 | 934 | 17653 | 2.94006661908161 | 0.00025796353207984 | 0.00457902196182666 | 3.58844168526128 | 0.0149892933618844 | PFN2//PLEK//PTGER4//TSC1//LPAR1//PPM1E//NF2//PPM1F//DLC1//S1PR1//TMEFF2//ARHGAP6//MET//PPFIA1// |
| GO:0046545 | development of primary female sexual characteristics | Biological process | 15 | 101 | 934 | 17653 | 2.80699429686009 | 0.00026580979673122 | 0.00470594674160534 | 3.57542901666679 | 0.0160599571734475 | CEBPB//FOXC1//INHBB//DMRTA1//IMMP2L//KMT2B//ESR1//EREG//PLEKHA1//IDH1//LHX8//ARID5B//SLIT2//ZFPM2//LHFPL2// |
| GO:0007049 | cell cycle | Biological process | 134 | 1898 | 934 | 17653 | 1.33438218523725 | 0.000271351162531551 | 0.00479150890914068 | 3.56646831346185 | 0.143468950749465 | KIF23//E2F1//USP28//CLOCK//CDKN1A//GADD45A//PTEN//CCNE2//CDK6//LATS2//PPP6C//RBBP8//ACVR1//NPAT//RRM2//NEDD1//CCNY//PPP1R12A//PRKAR2B//SKP1//YWHAG//MASTL//HAUS8//CEP41//MYB//MYBL1//RNF2//CLIP1//SRSF2//CDC23//USP3//RHOC//RND3//PAK6//E2F5//IRF1//JUN//NF2//STAT3//TSC1//PER2//CCNG2//CNOT6L//CNOT7//MDM4//CNOT6//RBL2//SOX4//BTG2//HBP1//MTBP//PKD2//PRKAA1//RRAGD//RHEB//TGFB2//TSG101//LAMTOR3//KAT2B//DYNLL2//DYNC1LI2//NUP43//CENPQ//FBXW7//RAD21//CENPO//NUP35//CNEP1R1//REEP3//PPP2CA//NABP1//TAOK3//HMGA2//CDC14A//BCL2L11//BAG6//SYCP3//EREG//CDK8//MAP3K2//ZNF268//CDK19//MINK1//MAP2K4//SLK//EPS8//CHMP3//HECW2//NEUROG1//KIF13A//CALM2//IPO5//MAPRE3//CCNC//CCND2//RUNX3//LRP6//NR4A3//FOSL1//EDN1//IGF1//BTG3//FOXC1//BTG1//TNF//MDM1//GOLGA8A//DUSP1//PRKACB//WASL//RBBP7//NEUROD1//GATA6//POU4F1//RNF4//MZT1//CEP97//TMOD3//YTHDF2//ZNF655//KLF4//CETN1//ZMYND11//ESCO2//E2F3//CD2AP//APPL1//MAPK1//GMNC//UBE2I//PTP4A1//EVI5//LMLN// |
| GO:1901653 | cellular response to peptide | Biological process | 35 | 353 | 934 | 17653 | 1.87398013964125 | 0.00027224676242832 | 0.00479480430808002 | 3.56503727619248 | 0.0374732334047109 | GAB1//APPL1//GRB2//SOS1//PIK3R3//CPEB2//INHBB//ERRFI1//PPARG//PTEN//CPEB1//SLC2A4//YWHAG//KAT2B//SESN3//NCK1//TSC1//IGF1//STAT3//MAPK1//EDN1//NR4A2//NR4A3//ADCY3//GJB2//PRKACB//PRKAR2B//RAP1B//EPHA4//CRK//PRKAA1//KLF2//KLF3//KLF10//KLF4// |
| GO:0001525 | angiogenesis | Biological process | 50 | 567 | 934 | 17653 | 1.66670443258595 | 0.000279726977134065 | 0.00491374947105891 | 3.55326564783837 | 0.0535331905781585 | EDN1//PPP3R1//TGFBR2//ACVR1//KDR//SLIT2//SPRED1//FOXC1//SULF1//PPARG//TGFB2//KLF4//EFNA1//EPHA2//ETS1//EGLN1//VASH2//F3//GAB1//GATA6//ITGB8//STAT3//BTG1//VEGFB//ADAM12//HMGA2//PIK3CB//ADIPOR2//TCF21//MEOX2//DLL1//KLF2//EPN1//ITGA5//JAK1//S1PR1//ELK3//EREG//CLIC4//HOXA3//ITGAV//JUN//MMP2//TMEM100//PTEN//CCL2//SOX17//WNT7A//HAND1//HAND2// |
| GO:0030323 | respiratory tube development | Biological process | 21 | 171 | 934 | 17653 | 2.32110522559074 | 0.000280667622154904 | 0.00491750033324771 | 3.55180768473107 | 0.0224839400428266 | FOXF1//ZFPM2//BAG6//CIC//MAN2A1//ERRFI1//BMPR2//TCF21//PKDCC//PHF14//MAPK1//SOX11//NOG//GATA6//RDH10//TGFBR2//NFIB//PPP3R1//KLF2//THRA//TNF// |
| GO:0071156 | regulation of cell cycle arrest | Biological process | 16 | 113 | 934 | 17653 | 2.67616683405658 | 0.000294394339277352 | 0.00514467420292695 | 3.53107054502831 | 0.0171306209850107 | CDKN1A//GADD45A//E2F1//CNOT6L//CNOT7//MDM4//CNOT6//RBL2//SOX4//BTG2//ZNF268//GATA6//PKD2//POU4F1//HMGA2//NEUROD1// |
| GO:0000188 | inactivation of MAPK activity | Biological process | 7 | 26 | 934 | 17653 | 5.08857684071817 | 0.000307555288439967 | 0.00534793687030141 | 3.51207680084459 | 0.00749464668094218 | DUSP10//SPRED1//DUSP1//DUSP2//DUSP5//PPP2CA//RGS3// |
| GO:0051240 | positive regulation of multicellular organismal process | Biological process | 116 | 1606 | 934 | 17653 | 1.36516169290215 | 0.000307607192451168 | 0.00534793687030141 | 3.51200351410684 | 0.124197002141328 | EREG//TMF1//TNF//ADRB1//F3//TGFBR2//EDN1//GJA1//NPNT//PLEK//SULF1//ETS1//KDR//BMPR2//WNT7A//MTPN//IGF1//TWF1//NR4A3//HAND2//JUN//TGFB2//DOCK5//PPM1F//FOXC1//BAMBI//TGFB1I1//STMN2//NPTN//NCK1//NME1//SERPINI1//ZNF804A//LDLR//INHBB//LHX1//ENPP4//OSR1//FAM20C//TFAP2A//ACVR1//PKDCC//WASF3//NRG1//POLR3G//IRF1//IL2//CEBPB//NPLOC4//POU3F2//OTX2//DUSP10//MYB//SOCS5//STAT3//DNMT3B//MMD//NEUROD1//NEUROG1//PTEN//RARB//SOX11//ZEB1//TMEM64//POU4F1//KLF10//GAB1//GATA6//ITGB8//BTG1//VEGFB//VASH2//ADAM12//HMGA2//GOLGA4//TOB2//ARX//SERP1//DLL1//PRKAA1//SOX17//EFNA5//LPAR3//PPARG//RHEB//PTGER4//MAN2A1//SLIT2//EPHA4//RAB21//CAPRIN2//ADAM9//SLITRK3//FLRT1//ZFPM2//P2RY1//AR//LPAR1//CPEB3//FMR1//C21ORF91//LRP8//MCOLN2//NOG//VLDLR//TRPC3//ITGA5//JAK1//KLF4//PLAA//LRP6//ERG//MET//CD274//TMEM100//NSMF// |
| GO:0022604 | regulation of cell morphogenesis | Biological process | 43 | 468 | 934 | 17653 | 1.73657781072128 | 0.000311085148806687 | 0.00539453554199904 | 3.50712072159866 | 0.0460385438972163 | DLC1//CDC42EP3//WASF3//PALM2//FGD4//CRK//FGD5//LPAR1//EPS8//BAMBI//KDR//RHOC//RND3//MSN//BRWD1//CCL2//WASL//DPYSL2//RAB21//EPHA4//WNT7A//GOLGA4//NRG1//EPHA7//ULK2//EFNA5//LPAR3//FMR1//NSMF//HECW2//FXR1//BMPR2//SEMA4B//EFNA1//POU3F2//PTEN//SLIT2//CAPRIN2//LRP8//CRKL//DOCK5//PLAA//SH3D19// |
| GO:0035270 | endocrine system development | Biological process | 17 | 125 | 934 | 17653 | 2.57045824411135 | 0.000314649862965527 | 0.00544239647886409 | 3.50217245305785 | 0.0182012847965739 | GATA6//CDK6//DLL1//NEUROD1//ETS1//NOG//POU3F2//DKK3//ARID5B//HOXA3//MAPK1//THRA//MNX1//SOX4//CRKL//RHEB//CLOCK// |
| GO:0060004 | reflex | Biological process | 6 | 19 | 934 | 17653 | 5.9685562943762 | 0.000322984884520765 | 0.00557231319901514 | 3.49081780188818 | 0.00642398286937901 | NR4A3//GLRA1//GLRB//USP46//GJA1//NPNT// |
| GO:0001837 | epithelial to mesenchymal transition | Biological process | 18 | 137 | 934 | 17653 | 2.48326794729521 | 0.000327268043052001 | 0.00561754765269209 | 3.48509640044746 | 0.019271948608137 | TMEM100//EFNA1//ERG//FOXC1//BAMBI//TGFB1I1//TGFB2//TGFBR2//PPP2CA//PTEN//LDLRAD4//LRP6//KBTBD8//NOG//ACVR1//FOXF2//PPP3R1//HMGA2// |
| GO:0051494 | negative regulation of cytoskeleton organization | Biological process | 18 | 137 | 934 | 17653 | 2.48326794729521 | 0.000327268043052001 | 0.00561754765269209 | 3.48509640044746 | 0.019271948608137 | EPS8//TWF1//STMN2//MID1IP1//LIMA1//PFN2//SLIT2//MDM1//KAT2B//DLC1//S1PR1//TMEFF2//ARHGAP6//MET//PPFIA1//TMOD3//TMOD1//MTPN// |
| GO:0035107 | appendage morphogenesis | Biological process | 19 | 149 | 934 | 17653 | 2.41012172513401 | 0.000333216652176157 | 0.00569076822895795 | 3.47727330328113 | 0.0203426124197002 | LRP6//MBNL1//TGFB2//ASPH//OSR1//RDH10//EN1//TFAP2A//WNT7A//RECK//RARB//FZD6//GJA1//GNAQ//NOG//HAND2//SOX4//SOX11//BCL2L11// |
| GO:0035108 | limb morphogenesis | Biological process | 19 | 149 | 934 | 17653 | 2.41012172513401 | 0.000333216652176157 | 0.00569076822895795 | 3.47727330328113 | 0.0203426124197002 | LRP6//MBNL1//TGFB2//OSR1//RDH10//EN1//TFAP2A//WNT7A//RECK//RARB//FZD6//GJA1//GNAQ//NOG//HAND2//SOX4//SOX11//BCL2L11//ASPH// |
| GO:0033002 | muscle cell proliferation | Biological process | 24 | 211 | 934 | 17653 | 2.14981174584166 | 0.000343631129193965 | 0.00585384717062666 | 3.46390750076396 | 0.0256959314775161 | NR4A3//S1PR1//EDN1//EREG//IGF1//MYB//TGFBR2//TNF//OGN//PPARG//KLF4//FOXC1//TGFB2//GJA1//JARID2//PTEN//NOG//ZFPM2//GATA6//STAT3//JUN//MEF2D//MAP3K5//MMP2// |
| GO:0022414 | reproductive process | Biological process | 104 | 1417 | 934 | 17653 | 1.38718739563483 | 0.000364974352850131 | 0.00620181293549104 | 3.437737652823 | 0.11134903640257 | CEBPB//FOXC1//INHBB//DMRTA1//IMMP2L//KMT2B//ESR1//EREG//PLEKHA1//YTHDF2//TBC1D20//AGFG1//TMF1//PPARG//PKD2//BMPR2//BCL2L11//CCNE2//BAG6//RAD21//MYBL1//SYCP3//MASTL//DZIP1//RNF2//ACVR1//CRTAP//DDX6//E2F1//GOLGA3//AR//CLDN11//ACOX1//PAIP2//ARID4B//ARID4A//HERPUD2//SOX17//BTG1//ADAM18//CLOCK//TDRD5//QKI//TOB2//SOS1//FOSL1//LHFPL2//ADCY3//TRPC3//STX2//GLRA1//GLRB//ZFPM2//TCF21//WNT7A//ETS1//EPN1//SPHK2//PTHLH//ADIPOR2//MMP2//TGFBR2//RECK//DRD1//NHLH2//THRA//OSR1//RDH10//RNF38//GATA6//TFAP2C//TGFB2//ARID5B//IDH1//LHX8//CLIC4//STAT3//NEUROG1//LRP6//LHX1//CREBRF//GNAQ//PTEN//GJA1//GJB2//FOXF2//PPP2CA//RBBP8//DUSP1//PRKACB//EDN1//IGF1//P2RY1//SYT6//NOG//ZNF568//GRB2//ITGB8//SULF1//HAND1//EGLN1//MAPK1//VASH2//SLIT2// |
| GO:0045732 | positive regulation of protein catabolic process | Biological process | 24 | 212 | 934 | 17653 | 2.13967112439901 | 0.00036856179857654 | 0.00624707630018331 | 3.43348968139907 | 0.0256959314775161 | RNF180//ANKIB1//LONRF3//LONRF1//SOCS5//SH3D19//TNF//ADAM9//FMR1//FBXW7//FBXL5//MSN//EDEM1//ATXN3//BAG6//RNF128//LDLR//PTEN//WWP2//GJA1//MYLIP//NSF//HECW2//SOX17// |
| GO:0045786 | negative regulation of cell cycle | Biological process | 54 | 634 | 934 | 17653 | 1.60981565668506 | 0.000381168596594078 | 0.00644460804691437 | 3.41888288685769 | 0.0578158458244111 | E2F1//USP28//CLOCK//CDKN1A//GADD45A//CNOT6L//CNOT7//MDM4//CNOT6//RBL2//SOX4//BTG2//CDK6//HBP1//MTBP//IRF1//PKD2//PRKAA1//RRAGD//RHEB//TGFB2//TSC1//TSG101//LAMTOR3//KAT2B//NABP1//TAOK3//HMGA2//RAD21//SKP1//RBBP8//PTEN//FBXW7//LATS2//IPO5//BTG3//FOXC1//BTG1//TNF//MDM1//DUSP1//PRKACB//RBBP7//RNF2//RRM2//NEUROD1//ZNF268//GATA6//POU4F1//CDC14A//SOX11//ZNF655//KLF4//RUNX3// |
| GO:0048566 | embryonic digestive tract development | Biological process | 8 | 35 | 934 | 17653 | 4.32009788926277 | 0.000390775904614039 | 0.00659056718928864 | 3.40807222288514 | 0.00856531049250535 | FOXF1//SOX11//TCF21//FOXF2//RARB//TGFB2//TNF//PKDCC// |
| GO:0000003 | reproduction | Biological process | 104 | 1420 | 934 | 17653 | 1.38425671803842 | 0.000393593361906189 | 0.00662157190689442 | 3.40495223471179 | 0.11134903640257 | CEBPB//FOXC1//INHBB//DMRTA1//IMMP2L//KMT2B//ESR1//EREG//PLEKHA1//YTHDF2//TBC1D20//AGFG1//TMF1//PPARG//PKD2//BMPR2//BCL2L11//CCNE2//BAG6//RAD21//MYBL1//SYCP3//MASTL//DZIP1//RNF2//ACVR1//CRTAP//DDX6//E2F1//GOLGA3//AR//CLDN11//ACOX1//PAIP2//ARID4B//ARID4A//HERPUD2//SOX17//BTG1//ADAM18//CLOCK//TDRD5//QKI//TOB2//SOS1//FOSL1//LHFPL2//ADCY3//TRPC3//STX2//GLRA1//GLRB//ZFPM2//TCF21//WNT7A//ETS1//EPN1//SPHK2//PTHLH//ADIPOR2//MMP2//TGFBR2//RECK//DRD1//NHLH2//THRA//OSR1//RDH10//RNF38//GATA6//TFAP2C//TGFB2//ARID5B//IDH1//LHX8//CLIC4//STAT3//NEUROG1//LRP6//LHX1//CREBRF//GNAQ//PTEN//GJA1//GJB2//FOXF2//PPP2CA//RBBP8//DUSP1//PRKACB//EDN1//IGF1//P2RY1//SYT6//NOG//ZNF568//GRB2//ITGB8//SULF1//HAND1//EGLN1//MAPK1//VASH2//SLIT2// |
| GO:0046660 | female sex differentiation | Biological process | 16 | 116 | 934 | 17653 | 2.60695562283098 | 0.000398013802767042 | 0.00667932344445038 | 3.40010186671621 | 0.0171306209850107 | CEBPB//FOXC1//INHBB//DMRTA1//IMMP2L//KMT2B//ESR1//EREG//PLEKHA1//IDH1//LHX8//ARID5B//LHFPL2//LHX1//SLIT2//ZFPM2// |
| GO:0031032 | actomyosin structure organization | Biological process | 22 | 188 | 934 | 17653 | 2.21175224383799 | 0.000402825281694737 | 0.00674333509926115 | 3.39488328053162 | 0.0235546038543897 | KIF23//TMOD3//TMOD1//ACTC1//CFL2//PTGER4//TSC1//LPAR1//PPM1E//NF2//PFN2//PPM1F//DLC1//S1PR1//TMEFF2//ARHGAP6//MET//PPFIA1//EDN1//FRMD6//CNN3//MYLIP// |
| GO:0046483 | heterocycle metabolic process | Biological process | 373 | 6136 | 934 | 17653 | 1.14893411718394 | 0.000405865630744079 | 0.00677745496474619 | 3.39161772377552 | 0.399357601713062 | IGF1//NFIA//NFIB//RBBP7//RBBP8//RRM2//E2F1//NPAT//TSHZ1//HMG20A//KLF2//ZBTB18//CEBPB//ZNF268//TCERG1//WWP2//KLF12//OSR1//CRY2//GLIS3//ARX//DNMT3B//DR1//EDN1//EFNA1//CC2D1B//EN1//ESR1//FHL2//CPEB3//FOXF1//FOXC1//MYT1L//CIC//ZFPM2//DNAJB5//GATA6//CNOT7//HSBP1//AR//JARID2//JUN//ZNF568//MAF//MDM4//MYB//NR4A2//RLIM//KLF3//ZBTB7A//PIAS4//POU4F1//PPARG//CC2D1A//TSHZ3//ZBTB4//RARB//REST//RNF2//PRDM16//SOX17//SOX11//STAT3//ZEB1//TCF21//TFAP2A//TFAP2C//TGIF1//KLF10//TNF//UBE2I//VLDLR//WFS1//ZNF217//LRP8//BTG2//BHLHE41//TBL1XR1//NR4A3//HMGA2//ARID5B//BRMS1L//BHLHE40//RUNX3//URI1//PER2//SQSTM1//NOG//KLF4//HAND1//FNIP1//PHF14//USP3//DCP2//PPP2CA//RPL15//CNOT6L//PAN3//CNOT6//RBFOX3//FMR1//MBNL1//RBM25//SRSF2//ELAVL2//NOVA1//COPS8//PTGES3//CCNE2//NABP1//GTF2E1//DUS2//IRF9//KLF6//CREBRF//EGR2//ELK3//ERG//FOXJ3//FOXF2//FLI1//GRHL1//HIVEP2//IRF1//LHX1//MEF2D//MEOX2//MYBL1//NEUROD1//NHLH2//OTX2//PAX9//PBX3//POU3F2//RAD21//ARID4A//SHOX//BCL11B//BMPR2//SOX4//BTF3//TEF//TGFB1I1//THRA//TMF1//DEK//FOSL1//KLF7//CBFB//FUBP3//HAND2//CLOCK//POLR3G//PKD2//UGP2//GFPT2//NAGK//SLC35D1//NUP35//NUP43//CHCHD10//COX8C//COX7A2L//DPYSL2//TTR//HPRT1//NME1//CMPK1//ADCY3//IMPDH1//PDE10A//DCTPP1//REV3L//GMNC//ESCO2//GADD45A//EPC2//TAOK3//UBE2W//USP28//USP45//ATXN3//KDM2A//MTRR//HMGB3//BAHD1//H2AFZ//KMT2B//DPY30//ARID4B//TOPORS//KHDRBS3//ZMYND11//TADA1//ZNF597//CTNND2//ZNF782//NKX2-3//ZNF800//E2F5//ZNF367//EZH1//ZBTB9//MYCBP2//FLII//LHX6//HBP1//ATAD2//SERTAD3//MDFIC//MNX1//HOXA3//HOXD1//FOXN2//ZBTB41//ZBTB34//LHX8//NEUROG1//NFE2//NPAS2//IER5//BRWD1//BNC2//ZNF532//CDCA7L//PRKAA1//TRERF1//LMO3//MAPK1//SLC2A4RG//PHTF2//ZNF630//ZNF287//ZNF512B//ZBTB26//MIER1//RBL2//RNF4//ATXN1//DMRTA1//SMARCD2//STAT4//ZNF2//ZNF3//ZNF131//ZNF655//BRD3//TCF7L1//PCGF5//LCOR//WASL//ZBTB47//ST18//HIPK3//NR5A2//NRBF2//MLLT6//RNF141//PAK6//SNAPC1//BTG1//SNIP1//RPS6KA4//RPS6KA5//CDKN1A//CRK//EMX2//ETS1//BOLA3//NR3C2//PLEK//ZNFX1//NKX2-4//RFX7//BDP1//CDK8//E2F3//TEAD1//KAT2B//CCNC//CMTR2//ZFC3H1//RTCA//PPP4R2//ESRP1//CPEB1//QKI//IDH1//UGT1A4//UGT1A1//G3BP2//NF2//EPHA2//PTHLH//PSAT1//EREG//UGT1A8//UGT1A7//DDX6//ACOT7//PPCS//GLRX//INTS8//RBM47//MAP3K2//MAPRE3//BAMBI//RGMB//LRP6//MAP3K5//WNT7A//ACVR1//DKK3//NRG1//RASD1//TSG101//BTAF1//PPM1F//TOB1//SOSTDC1//TMEM100//RGMA//BMP3//TGFB2//SULF1//CTDSPL2//CALM2//ADRB1//EGLN1//CMKLR1//CCT6A//EPHA5//FBXW7//UXS1//RNF180//TDRD5//FZD6//FASTK//AHNAK//YTHDF2//FXR1//NPLOC4//MAP2K4//S1PR1//DLL1//IL2//ITGA6//MET//PPP1R12A//NCK1//OSM//P2RY1//PPP3R1//DCAF6//CAPRIN2//FAM20C//MPP1//RAB23//ENPP4//SLC25A32//PTEN//MTPN//EGLN3//PFKP//REXO1//ZC3H12C//NANOS1//RAP2C//TOB2//KLHL15//DUSP1//GJA1//NPNT// |
| GO:0043244 | regulation of protein complex disassembly | Biological process | 15 | 105 | 934 | 17653 | 2.70006118078923 | 0.000408532066948166 | 0.00680517824820307 | 3.38877384867365 | 0.0160599571734475 | EPS8//TWF1//STMN2//MID1IP1//LIMA1//CFL2//PLEK//TNF//BNIP3//TMOD3//TMOD1//CALCOCO2//FYCO1//ASPH//MTPN// |
| GO:0003208 | cardiac ventricle morphogenesis | Biological process | 12 | 73 | 934 | 17653 | 3.10691971488076 | 0.000416349184782693 | 0.00691835266998858 | 3.38054228135929 | 0.012847965738758 | SOX4//SOX11//FOXF1//TGFBR2//TGFB2//HAND1//HAND2//ZFPM2//NRG1//POU4F1//NOG//FOXC1// |
| GO:0016236 | macroautophagy | Biological process | 30 | 293 | 934 | 17653 | 1.93519743333017 | 0.000424696974417601 | 0.00701829537253874 | 3.37192083295427 | 0.0321199143468951 | UBXN2B//TRAPPC8//RAB23//ATG16L1//ULK2//SQSTM1//SESN3//RAB12//SESN1//KDR//BNIP3//TSC1//SNX5//PRKAA1//RRAGD//RHEB//LAMTOR3//WDFY3//CHMP3//TSG101//CALCOCO2//FYCO1//FEZ2//PIKFYVE//TBC1D12//RAB3GAP1//VPS37A//DYNLL2//PLEKHF2//PLAA// |
| GO:0006665 | sphingolipid metabolic process | Biological process | 20 | 164 | 934 | 17653 | 2.30493027628349 | 0.000425477022436919 | 0.00701829537253874 | 3.37112388868939 | 0.0214132762312634 | ITGB8//ST6GALNAC3//ST6GALNAC6//ST8SIA3//ST3GAL5//SGPP1//SPHK2//PPP2CA//NSMAF//ORMDL3//B3GALT2//CTSA//ST8SIA5//PPM1L//SPTSSA//ELOVL4//ELOVL7//PRKAA1//TNF//SERINC3// |
| GO:0008361 | regulation of cell size | Biological process | 20 | 164 | 934 | 17653 | 2.30493027628349 | 0.000425477022436919 | 0.00701829537253874 | 3.37112388868939 | 0.0214132762312634 | CLCN6//SLC12A5//DPYSL2//RAB21//EPHA4//GOLGA4//NRG1//PTEN//TSC1//EDN1//PRR16//EPHA7//ULK2//EFNA5//LPAR3//BMPR2//SEMA4B//PLEK//MTPN//MSN// |
| GO:0000165 | MAPK cascade | Biological process | 74 | 944 | 934 | 17653 | 1.48160136827206 | 0.000431567558316018 | 0.00710143892187647 | 3.36495120943371 | 0.0792291220556745 | GADD45A//MINK1//TNF//MAP3K2//CRK//CRKL//MAP3K5//TAOK3//LAMTOR3//ADAM9//DUSP5//LPAR1//LPAR3//NRG1//IGF1//NTF3//PIK3CB//PRKAA1//MAPK1//MAP2K4//DUSP10//SPRED1//DUSP1//DUSP2//PPP2CA//RGS3//PTGER4//MDFIC//TGFB2//TSG101//EDN1//GDF11//GRB2//INHBB//PAK6//BMP3//EFNA1//NF2//EPHA2//EPHA4//EPHA5//EPHA7//AR//ITGAV//KDR//MET//OSM//FZD10//HIPK3//AIDA//ZMYND11//WNT7A//SKP1//RANBP9//ERRFI1//LMO3//PTEN//KLF4//NPNT//DSTYK//NPTN//JUN//P2RY1//DNAJC27//FBXW7//CCL2//CCL8//HAND2//PPM1L//EREG//RASGEF1A//IL2//JAK1//SOS1// |
| GO:0032879 | regulation of localization | Biological process | 180 | 2700 | 934 | 17653 | 1.26002855103498 | 0.000433144139199326 | 0.00711008207137146 | 3.36336755759099 | 0.19271948608137 | ARL6IP1//FMR1//NTF3//MIA3//CCL8//SLIT2//TNF//EDN1//RAPGEF4//MARCKS//NEUROD1//SNAP25//PER2//CLOCK//TRPC3//ETS1//KDR//BMPR2//WNT7A//DUSP10//PFN2//PTPRG//JUN//TGFB2//TGFBR2//DOCK5//PPM1F//P2RY1//CMKLR1//C5ORF30//ADAM10//NR4A3//CALM2//FBXW7//PPARG//ITGAV//SLC6A1//CRK//IGF1//ADIPOR2//INHBB//SOX11//RAB21//NSF//SYT6//TRPV6//NRG1//GJA1//RUFY2//RHOC//RND3//LAMA3//NCK1//MINK1//SLK//SEMA4B//ZNF268//DRD1//S1PR1//F3//FOXF1//CD274//ITGA6//ITGA5//MAPK1//PTP4A1//ACVR1//DLC1//SULF1//TMEFF2//CLIC4//NF2//PTEN//RAP2C//LDLRAD4//RECK//NOG//TBC1D12//EVI5//TBC1D17//USP6NL//PKD2//SERP1//SOX4//STIM2//WWP2//NDFIP2//STX6//MMD//WASL//CDKN1A//KCNA4//KCNJ3//KCNJ10//KCNK10//HCN2//SCN1A//SYBU//GAB1//IPO5//MDFIC//LYPLA1//PKDCC//PXK//EFNA1//EPHA2//DLL1//LDLR//REST//APPL1//PPP1R12A//CTDSPL2//OSM//CD2AP//ARFIP1//PTGER4//ADAM9//IL2//LPAR3//WFS1//PRKAA1//VEGFB//EFNA5//EPHA5//RAB3GAP1//LPAR1//DNAJC27//MPP1//SPRED1//MEOX2//KLF4//CCL2//MIEF1//DSC2//BCL2L11//DYNLL2//E2F1//PMAIP1//PPP3R1//YWHAG//AHNAK//GNB5//NEFH//NPTN//TBC1D20//MSN//AR//PPFIA1//SQSTM1//PLAA//TSG101//CCNE2//ELMOD1//SNIP1//DERL2//EDEM1//CCT6A//DCP2//SESTD1//CENPQ//OSR1//NEUROG1//CRY2//CDK6//EREG//MEMO1//PIK3CB//LRCH1//CXCL14//CHMP3//HECW2//GLRX//TNFRSF21//MYB//TMF1//MET//ARHGAP1//STAT3//NSMF//PIK3R3// |
| GO:0006725 | cellular aromatic compound metabolic process | Biological process | 375 | 6178 | 934 | 17653 | 1.14724192288309 | 0.000436301012076473 | 0.00714456112511667 | 3.36021377959004 | 0.401498929336188 | IGF1//NFIA//NFIB//RBBP7//RBBP8//RRM2//E2F1//NPAT//TSHZ1//HMG20A//KLF2//ZBTB18//CEBPB//ZNF268//TCERG1//WWP2//KLF12//OSR1//CRY2//GLIS3//ARX//DNMT3B//DR1//EDN1//EFNA1//CC2D1B//EN1//ESR1//FHL2//CPEB3//FOXF1//FOXC1//MYT1L//CIC//ZFPM2//DNAJB5//GATA6//CNOT7//HSBP1//AR//JARID2//JUN//ZNF568//MAF//MDM4//MYB//NR4A2//RLIM//KLF3//ZBTB7A//PIAS4//POU4F1//PPARG//CC2D1A//TSHZ3//ZBTB4//RARB//REST//RNF2//PRDM16//SOX17//SOX11//STAT3//ZEB1//TCF21//TFAP2A//TFAP2C//TGIF1//KLF10//TNF//UBE2I//VLDLR//WFS1//ZNF217//LRP8//BTG2//BHLHE41//TBL1XR1//NR4A3//HMGA2//ARID5B//BRMS1L//BHLHE40//RUNX3//URI1//PER2//SQSTM1//NOG//KLF4//HAND1//FNIP1//PHF14//USP3//DCP2//PPP2CA//RPL15//CNOT6L//PAN3//CNOT6//RBFOX3//FMR1//MBNL1//RBM25//SRSF2//ELAVL2//NOVA1//COPS8//PTGES3//CCNE2//NABP1//GTF2E1//DUS2//IRF9//KLF6//CREBRF//EGR2//ELK3//ERG//FOXJ3//FOXF2//FLI1//GRHL1//HIVEP2//IRF1//LHX1//MEF2D//MEOX2//MYBL1//NEUROD1//NHLH2//OTX2//PAX9//PBX3//POU3F2//RAD21//ARID4A//SHOX//BCL11B//BMPR2//SOX4//BTF3//TEF//TGFB1I1//THRA//TMF1//DEK//FOSL1//KLF7//CBFB//FUBP3//HAND2//CLOCK//POLR3G//PKD2//UGP2//GFPT2//NAGK//SLC35D1//NUP35//NUP43//CHCHD10//COX8C//COX7A2L//DPYSL2//TTR//HPRT1//NME1//CMPK1//ADCY3//IMPDH1//PDE10A//DCTPP1//REV3L//GMNC//ESCO2//GADD45A//EPC2//TAOK3//UBE2W//USP28//USP45//ATXN3//KDM2A//MTRR//HMGB3//BAHD1//H2AFZ//KMT2B//DPY30//ARID4B//TOPORS//KHDRBS3//ZMYND11//TADA1//ZNF597//CTNND2//ZNF782//NKX2-3//ZNF800//E2F5//ZNF367//EZH1//ZBTB9//MYCBP2//FLII//LHX6//HBP1//ATAD2//SERTAD3//MDFIC//MNX1//HOXA3//HOXD1//FOXN2//ZBTB41//ZBTB34//LHX8//NEUROG1//NFE2//NPAS2//IER5//BRWD1//BNC2//ZNF532//CDCA7L//PRKAA1//TRERF1//LMO3//MAPK1//SLC2A4RG//PHTF2//ZNF630//ZNF287//ZNF512B//ZBTB26//MIER1//RBL2//RNF4//ATXN1//DMRTA1//SMARCD2//STAT4//ZNF2//ZNF3//ZNF131//ZNF655//BRD3//TCF7L1//PCGF5//LCOR//WASL//ZBTB47//ST18//HIPK3//NR5A2//NRBF2//MLLT6//RNF141//PAK6//SNAPC1//BTG1//SNIP1//RPS6KA4//RPS6KA5//CDKN1A//CRK//EMX2//ETS1//BOLA3//NR3C2//PLEK//ZNFX1//NKX2-4//RFX7//BDP1//CDK8//E2F3//TEAD1//KAT2B//CCNC//CMTR2//ZFC3H1//RTCA//PPP4R2//ESRP1//CPEB1//QKI//MTPN//IDH1//UGT1A4//UGT1A1//G3BP2//NF2//EPHA2//PTHLH//PSAT1//EREG//UGT1A8//UGT1A7//DDX6//ACOT7//PPCS//GLRX//INTS8//RBM47//MAP3K2//MAPRE3//BAMBI//RGMB//LRP6//MAP3K5//WNT7A//ACVR1//DKK3//NRG1//RASD1//TSG101//BTAF1//PPM1F//PON2//TOB1//SOSTDC1//TMEM100//RGMA//BMP3//TGFB2//SULF1//CTDSPL2//CALM2//ADRB1//EGLN1//CMKLR1//CCT6A//EPHA5//FBXW7//UXS1//DRD1//RNF180//TDRD5//FZD6//FASTK//AHNAK//YTHDF2//FXR1//NPLOC4//MAP2K4//S1PR1//DLL1//IL2//ITGA6//MET//PPP1R12A//NCK1//OSM//P2RY1//PPP3R1//DCAF6//CAPRIN2//FAM20C//MPP1//RAB23//ENPP4//SLC25A32//PTEN//EGLN3//PFKP//REXO1//ZC3H12C//NANOS1//RAP2C//TOB2//KLHL15//DUSP1//GJA1//NPNT// |
| GO:0048754 | branching morphogenesis of an epithelial tube | Biological process | 19 | 153 | 934 | 17653 | 2.34711200682986 | 0.00046693036379632 | 0.00762765712646018 | 3.33074788361809 | 0.0203426124197002 | EDN1//PPP3R1//TGFBR2//ACVR1//NPNT//LHX1//PKD2//TCF21//RDH10//FOXF1//EPHA2//LRP6//AR//ESR1//TNF//NOG//CLIC4//MET//SLIT2// |
| GO:0060323 | head morphogenesis | Biological process | 8 | 36 | 934 | 17653 | 4.20009517011658 | 0.00047938707844162 | 0.0078122766542185 | 3.31931367626217 | 0.00856531049250535 | RAB3GAP1//LRP6//MMP2//ASPH//PAX9//PLEKHA1//ARID5B//NOG// |
| GO:0007267 | cell-cell signaling | Biological process | 118 | 1658 | 934 | 17653 | 1.34514507559222 | 0.00048117330002971 | 0.00782253612524262 | 3.31769847936103 | 0.126338329764454 | DRD1//HMGA2//SOX17//APCDD1//CTNND2//AMER2//CCNY//SOSTDC1//HBP1//DKK3//LRP6//PIAS4//PRKAA1//SKP1//STRN//TGFB1I1//WNT7A//TCF7L1//PPP3R1//FZD6//GJC1//LPAR3//GLRA1//GLRB//GRIA1//KIF5A//ATXN3//NOVA1//MINK1//MAPK1//SLC12A5//SLC6A1//SNAP25//SLC32A1//PPFIA1//PTEN//CHRM2//CHRM5//RAPGEF4//MARCKS//NEUROD1//TNF//PER2//CLOCK//INHBB//EDN1//P2RY1//SOX11//LHX1//ESR1//MDFIC//PPP2CA//CBFB//SULF1//SOX4//FRZB//SHISA2//STX2//GJA1//SERP1//USP46//TMF1//SLC17A7//SLC17A6//FZD10//SYBU//TMEM64//DLL1//REST//OSM//FMR1//CPEB3//YWHAG//EGR2//NSMF//KCNJ10//NPTN//RAB3GAP1//SYT6//NTF3//PXK//LRP8//TSHZ3//NR4A2//KLF4//VANGL1//SLC24A2//EFNA5//EPHA5//LATS2//NOG//BAMBI//CAPRIN2//TBL1XR1//KCNJ3//FXR1//PAIP2//EPHA4//SQSTM1//PFN2//MYB//ADAM10//ADRB1//EFNA1//EREG//FJX1//GJB2//GRB2//IL2//AR//PTHLH//HCN2//CCL8//BMP3//TFAP2C//TGFB2//KLF10//CXCL14// |
| GO:0097435 | supramolecular fiber organization | Biological process | 54 | 641 | 934 | 17653 | 1.59223576651846 | 0.000495683422176711 | 0.00802339393753187 | 3.30479560556039 | 0.0578158458244111 | EPS8//RHOC//RND3//MARCKS//MYO1B//LIMA1//TWF1//CFL2//PALM2//CD2AP//TMOD3//NCK1//TMOD1//ARHGAP12//STMN2//RANBP9//SLAIN2//MID1IP1//WASL//WASF3//ARHGAP6//COL1A2//FOXC1//TGFB2//ACTC1//PFN2//PLEK//SLIT2//CDC42EP3//GRB2//MET//CLIP1//SLK//NEFM//NEFH//IQGAP2//ARFIP1//AGFG1//MZT1//PTGER4//TSC1//LPAR1//PPM1E//NF2//PPM1F//DLC1//S1PR1//TMEFF2//PPFIA1//EDN1//FRY//LDLR//MTPN//MFAP5// |
| GO:0048638 | regulation of developmental growth | Biological process | 32 | 323 | 934 | 17653 | 1.87248824921606 | 0.000495901029999752 | 0.00802339393753187 | 3.30460498966666 | 0.0342612419700214 | DPYSL2//RAB21//EPHA4//IGF1//STAT3//ADRB1//POU3F2//TGFBR2//GOLGA4//NRG1//BCL2L11//FOXC1//LATS2//PTEN//ARX//SERP1//DLL1//EPHA7//ULK2//EFNA5//LPAR3//BMPR2//SEMA4B//GJA1//JARID2//NOG//ZFPM2//GATA6//EDN1//PLAA//DUSP10//AR// |
| GO:1902904 | negative regulation of supramolecular fiber organization | Biological process | 17 | 130 | 934 | 17653 | 2.47159446549168 | 0.000501174205826671 | 0.00805853626460221 | 3.30001128914519 | 0.0182012847965739 | EPS8//TWF1//STMN2//MID1IP1//LIMA1//PFN2//SLIT2//DLC1//S1PR1//TMEFF2//ARHGAP6//MET//PPFIA1//TMOD3//TMOD1//LDLR//MTPN// |
| GO:0045778 | positive regulation of ossification | Biological process | 13 | 85 | 934 | 17653 | 2.8906537347273 | 0.000501353836054674 | 0.00805853626460221 | 3.29985565775259 | 0.0139186295503212 | OSR1//FAM20C//BMPR2//TFAP2A//ACVR1//PKDCC//CEBPB//NPNT//GJA1//IGF1//SOX11//TOB2//TGFB2// |
| GO:0010810 | regulation of cell-substrate adhesion | Biological process | 22 | 191 | 934 | 17653 | 2.17701267979865 | 0.000501647755049169 | 0.00805853626460221 | 3.29960112694781 | 0.0235546038543897 | MINK1//PIK3CB//TSC1//NF2//CDK6//FOXF1//NPNT//ITGA6//ITGA5//SMOC2//PPM1F//SPOCK1//EFNA5//SLK//KDR//DLC1//ARHGAP6//PTEN//CRK//CRKL//DOCK5//ATXN3// |
| GO:0071901 | negative regulation of protein serine/threonine kinase activity | Biological process | 18 | 142 | 934 | 17653 | 2.3958289350665 | 0.000508384940638885 | 0.00814044197514868 | 3.29380732258361 | 0.019271948608137 | DUSP10//SPRED1//DUSP1//DUSP2//DUSP5//PPP2CA//RGS3//CDK6//PTEN//HIPK3//AIDA//CDKN1A//LATS2//IPO5//KAT2B//PRKAR2B//LRP6//YWHAG// |
| GO:0008585 | female gonad development | Biological process | 14 | 96 | 934 | 17653 | 2.75631245538901 | 0.000509153771327501 | 0.00814044197514868 | 3.29315103504349 | 0.0149892933618844 | CEBPB//FOXC1//INHBB//DMRTA1//IMMP2L//KMT2B//ESR1//EREG//PLEKHA1//SLIT2//ZFPM2//IDH1//LHX8//ARID5B// |
| GO:0019941 | modification-dependent protein catabolic process | Biological process | 51 | 597 | 934 | 17653 | 1.61460944981869 | 0.000512767840922557 | 0.00817888893433786 | 3.29007922012698 | 0.0546038543897216 | TOPORS//USP24//KLHL3//MYLIP//USP27X//ATXN3//RLIM//NPLOC4//USP28//USP46//UBE2I//BAG6//RNF128//KLHL15//USP45//CDC23//SQSTM1//USP3//UBXN4//DNAJB9//DERL2//JKAMP//WFS1//EDEM1//TGFB1I1//FBXL5//FBXW7//SKP1//BAG5//RNF180//ANKIB1//LONRF3//LONRF1//SOCS5//WWP2//UBXN2B//RNF38//RNF145//ZNRF2//CD2AP//SPOPL//UBE2W//HECW2//RNF4//TBL1XR1//PLAA//CLOCK//VPS37A//TSG101//PTEN//ZFAND4// |
| GO:0003151 | outflow tract morphogenesis | Biological process | 12 | 75 | 934 | 17653 | 3.02406852248394 | 0.000536947363513305 | 0.00854441181044819 | 3.27006828572715 | 0.012847965738758 | ZFPM2//GATA6//RARB//BMPR2//TGFB2//TGFBR2//HAND2//FOXC1//JUN//SOX17//SOX11//NOG// |
| GO:0006629 | lipid metabolic process | Biological process | 104 | 1433 | 934 | 17653 | 1.37169891110576 | 0.000542711186826953 | 0.00861585858335841 | 3.26543122627911 | 0.11134903640257 | ACOX1//PTGES3//EDN1//RDH10//NAPEPLD//TTR//LRP8//ITGB8//ST6GALNAC3//ST6GALNAC6//ST8SIA3//ST3GAL5//ACSL1//PIGA//LYPLA1//ACOT7//UGT1A8//PRKAR2B//MSMO1//PER2//PRKAA1//ELOVL4//SCD5//NKX2-3//GATA6//PLAA//LCLAT1//DDHD1//CDS1//ABHD3//SLC44A1//SERINC3//PIKFYVE//PIK3CB//PTEN//PLEKHA1//PLEKHA3//MTMR9//PIK3R3//KDELC1//SGPP1//SPHK2//PPP2CA//NSMAF//ORMDL3//B3GALT2//CTSA//ST8SIA5//ZADH2//TRERF1//ACBD3//LBR//UGT1A1//OSBPL5//LDLR//VLDLR//NR5A2//ESR1//DGKE//ACBD5//B3GNT5//CNEP1R1//FBXW7//TBL1XR1//FHL2//RGL1//TNFRSF21//GRHL1//NPAS2//UGT1A9//PPARG//BCL11B//ELOVL7//PON2//ADIPOR2//PPM1L//SPTSSA//DKK3//REST//GAB1//GRB2//PIAS4//UBE2I//UGT1A7//UGT1A3//QKI//NRBF2//RBL2//KLF4//CCDC71L//FAM135A//MID1IP1//NR4A3//DGKH//PLEK//EREG//EFR3A//NRG1//MET//ZFYVE21//THRA//TNF//CHRM5//IDH1// |
| GO:0046513 | ceramide biosynthetic process | Biological process | 10 | 55 | 934 | 17653 | 3.43644150282266 | 0.000545751607935344 | 0.00864383635706495 | 3.26300497606515 | 0.0107066381156317 | ST6GALNAC3//ST6GALNAC6//ST8SIA3//ST3GAL5//B3GALT2//PRKAA1//ORMDL3//TNF//NSMAF//SPTSSA// |
| GO:0009952 | anterior/posterior pattern specification | Biological process | 23 | 205 | 934 | 17653 | 2.12053585418081 | 0.00054942732561545 | 0.00866996364421505 | 3.26008974486287 | 0.0246252676659529 | FOXF1//FOXC1//DLL1//MEOX2//NEUROG1//PBX3//LHX1//RNF2//GDF11//SOX17//TDRD5//EN1//LRP6//NOG//OSR1//OTX2//TSHZ1//CRKL//EMX2//HOXA3//NEUROD1//BMPR2//BTG2// |
| GO:0045926 | negative regulation of growth | Biological process | 26 | 244 | 934 | 17653 | 2.01398006108049 | 0.00054996516388707 | 0.00866996364421505 | 3.25966481888896 | 0.0278372591006424 | CDKN1A//FRZB//GJA1//SERTAD3//PPARG//PPP2CA//RBBP7//SOX17//BMPR2//CAPRIN2//BTG1//TGFB2//ADIPOR2//SLIT2//EPHA4//TNF//SQSTM1//ADRB1//PTEN//EPHA7//ULK2//SEMA4B//JARID2//TGFBR2//NOG//DUSP10// |
| GO:0007389 | pattern specification process | Biological process | 39 | 424 | 934 | 17653 | 1.7384827885742 | 0.000569184986618496 | 0.00895208852209509 | 3.24474656381392 | 0.0417558886509636 | WNT7A//FOXF1//FOXC1//DLL1//MEOX2//GJA1//PKD2//SOX17//TGFBR2//HAND1//HAND2//LHX1//LRP6//NEUROG1//ACVR1//PBX3//RNF2//TSHZ1//CRKL//EMX2//HOXA3//NEUROD1//BMPR2//BTG2//EDN1//EN1//DSCAML1//AIDA//NOG//GDF11//TDRD5//EGR2//AR//OSR1//OTX2//PRKACB//SOSTDC1//ASPH//ZEB1// |
| GO:0048583 | regulation of response to stimulus | Biological process | 267 | 4243 | 934 | 17653 | 1.18935054133751 | 0.000575970983322564 | 0.00903779990768097 | 3.23959939522318 | 0.285867237687366 | GADD45A//MINK1//TNF//MAP3K2//CRK//CRKL//MAP3K5//TAOK3//LAMTOR3//ADAM9//DUSP5//LPAR1//LPAR3//NRG1//IGF1//NTF3//PIK3CB//PRKAA1//MAPK1//MAP2K4//DUSP10//SPRED1//DUSP1//DUSP2//PPP2CA//RGS3//CBFB//ARL6IP1//THRA//PRKACB//SKP1//RPS6KA5//BAG6//F3//PPARG//OSM//SLIT2//IRF1//SOX17//EREG//FBXW7//ERRFI1//TSG101//SOCS5//NCK1//COPS8//MDFIC//GIT2//OTX2//YWHAG//TOB1//EPS8//ARHGAP1//ARHGAP6//EPHA2//EPHA4//EPHA5//EPHA7//RGS17//KDR//MET//ACVR1//GDF11//EDN1//IFNA1//IL2//INHBB//OGN//P2RY1//PTHLH//CCL2//CCL8//BMP3//TGFB2//TTR//VEGFB//WNT7A//CXCL14//PPP4R2//PLEK//MTPN//TWF1//NR4A3//HAND2//JARID2//PDE10A//AKAP11//CMKLR1//PIAS4//ADAM10//BMPR2//PMEPA1//LDLRAD4//PTEN//KLF4//DKK3//SOX11//ZEB1//DENND1B//TNFRSF21//ESR1//TCF7L1//SULF1//SOX4//APCDD1//FRZB//SOSTDC1//SHISA2//ENPP4//NPNT//TGFB1I1//BAMBI//ASPN//PRDM16//RASL11B//TGFBR2//GATA6//CTDSPL2//NOG//ITGA5//PKD2//TSC1//FNIP1//RRAGD//RHEB//ARAP2//APPL1//AGFG1//AGFG2//PSD//RALGPS2//UBA5//AR//FGD4//FGD5//ARHGEF4//ARHGEF28//SOS1//SOS2//DLC1//NF2//LMO3//SYBU//GRB2//JUN//PPP3R1//WASL//NPLOC4//FAM20C//EPS15//EPN1//CNOT7//STAT3//ZFAND6//SQSTM1//GJA1//RHOC//NDFIP2//CC2D1A//MIER1//SLC20A1//UBE2I//ZMYND11//FOXF1//PAK6//EFNA1//ITGAV//FZD10//HIPK3//AIDA//PMAIP1//PEX5L//ADRB1//POLR3G//MMP2//MYB//NPAS2//DSTYK//NPTN//WWP2//YTHDF2//DLL1//KAT2B//RAET1E//FLRT1//NEUROD1//RASGEF1A//SESN3//RGMA//SEMA4B//ETS1//PTGER4//CDK19//LDLR//CLOCK//COL1A2//TREM1//TMEM100//PLEKHA1//PPM1F//S1PR1//FAM13A//TAGAP//TRIP10//ARHGAP12//CD2AP//DRD1//GAB1//OTUD3//PER2//CALM2//JAK1//TCF21//CTNND2//AMER2//CCNY//LRP6//EFNA5//RANBP9//DNAJC27//ARRDC3//MPP1//TMEM64//LATS2//FZD6//BCL2L11//MOAP1//BNIP3//CAPRIN2//TBL1XR1//LYPD6//C5ORF30//PTGES3//NUP35//HSBP1//NUP43//BAG5//IER5//CREBRF//DYNLL2//E2F1//SESN1//MDM4//POU4F1//RBBP7//RBBP8//WFS1//SERINC3//TRPC3//DNAJB9//PPP1R15B//DERL2//EDEM1//ATXN3//OSR1//KLHL15//CRY2//PHF14//HMGA2//CDK6//DEK//FMR1//NR4A2//ITGA6//URI1//ST18//PIK3R3// |
| GO:0060541 | respiratory system development | Biological process | 22 | 193 | 934 | 17653 | 2.15445296290954 | 0.000578710651703135 | 0.00905976883673218 | 3.23753852397251 | 0.0235546038543897 | FOXF1//ZFPM2//BAG6//RDH10//CIC//MAN2A1//ERRFI1//BMPR2//TCF21//PKDCC//PHF14//MAPK1//SOX11//NOG//GATA6//TGFBR2//NFIB//PPP3R1//KLF2//THRA//LRP6//TNF// |
| GO:0072132 | mesenchyme morphogenesis | Biological process | 9 | 46 | 934 | 17653 | 3.69790987803743 | 0.000586648971386226 | 0.00916283370319872 | 3.23162168647123 | 0.00963597430406852 | TMEM100//TGFB2//ACVR1//NOG//TGFBR2//FOXC1//FOXF1//OSR1//ACTC1// |
| GO:0003181 | atrioventricular valve morphogenesis | Biological process | 6 | 21 | 934 | 17653 | 5.40012236157846 | 0.000589792826123745 | 0.00919071171215412 | 3.22930051424237 | 0.00642398286937901 | EFNA1//BMPR2//SOX4//ACVR1//TGFBR2//TGFB2// |
| GO:0051241 | negative regulation of multicellular organismal process | Biological process | 87 | 1162 | 934 | 17653 | 1.41509230559436 | 0.000606504032646853 | 0.00942297753032134 | 3.2171663071627 | 0.0931477516059957 | RPS6KA4//RPS6KA5//TNF//NME1//BMPR2//SOX17//HS3ST5//SLIT2//JARID2//DUSP10//PFN2//PTPRG//EFNA1//PPP2CA//PTEN//LDLRAD4//TGFB2//STMN2//LPAR1//EPHA4//MYLIP//SPOCK1//TSC1//BAG5//KLF4//SPRED1//FOXC1//SULF1//PPARG//INHBB//EDN1//TNFRSF21//ARRDC3//PTHLH//RARB//NPLOC4//CD274//PDCD1LG2//ERRFI1//DLL1//CMKLR1//KLF2//SOCS5//CD2AP//GATA6//ADRB1//RNF128//HMGB3//IRF1//CDK6//HMG20A//DDX6//REST//TOB1//TMEM64//NOG//HAND2//TOB2//IL2//EPHA7//ULK2//RGMA//SEMA4B//PTGER4//WNT7A//GLRA1//GJA1//TGFBR2//SOX11//IGF1//FRZB//LDLR//ASPN//OSR1//MEOX2//C5ORF30//NEUROG1//PRKACB//EPN1//DOCK5//SOSTDC1//ZFPM2//FBXW7//NFIB//PHF14//NRG1//STAT3// |
| GO:0031016 | pancreas development | Biological process | 12 | 76 | 934 | 17653 | 2.9842781471881 | 0.000607484578326202 | 0.00942297753032134 | 3.21646474263968 | 0.012847965738758 | GATA6//CDK6//DLL1//NEUROD1//MNX1//SOX4//NR5A2//RHEB//CLOCK//GDF11//FOXF1//WFS1// |
| GO:0006511 | ubiquitin-dependent protein catabolic process | Biological process | 50 | 587 | 934 | 17653 | 1.60991722874997 | 0.000618795105791308 | 0.009576456065141 | 3.20845312990517 | 0.0535331905781585 | UBXN4//DNAJB9//DERL2//JKAMP//NPLOC4//WFS1//BAG6//EDEM1//TGFB1I1//CDC23//FBXL5//FBXW7//SKP1//BAG5//RNF180//ANKIB1//LONRF3//LONRF1//SOCS5//TOPORS//WWP2//UBXN2B//RNF38//RNF145//ZNRF2//CD2AP//SPOPL//ATXN3//RLIM//UBE2W//HECW2//RNF4//TBL1XR1//PLAA//CLOCK//VPS37A//TSG101//KLHL15//PTEN//USP24//KLHL3//MYLIP//USP27X//USP28//USP46//UBE2I//RNF128//USP45//SQSTM1//USP3// |
| GO:0019098 | reproductive behavior | Biological process | 7 | 29 | 934 | 17653 | 4.56217233995422 | 0.000635097416942312 | 0.00980631011593803 | 3.19715965361265 | 0.00749464668094218 | DRD1//NHLH2//THRA//CREBRF//GNAQ//PTEN//DMRTA1// |
| GO:0010256 | endomembrane system organization | Biological process | 39 | 427 | 934 | 17653 | 1.72626862378328 | 0.000652133180585162 | 0.0100464161737983 | 3.18566370229455 | 0.0417558886509636 | TBC1D20//AGFG1//TMF1//REEP3//SERP1//UBXN2B//TRAPPC8//GOLGA8A//RAB30//TMED9//NPLOC4//BAG5//USP6NL//STX6//RAB22A//AKTIP//FAM160A2//SQSTM1//NUP35//CNEP1R1//NUP43//PPP2CA//PLSCR4//PTEN//VPS37A//CHMP3//TSG101//TMEFF2//AR//TGFB2//RAB38//XKR8//RTN3//ARL6IP1//BAG6//MAPK1//RAB3GAP1//WASL//STX2// |
| GO:0046467 | membrane lipid biosynthetic process | Biological process | 18 | 145 | 934 | 17653 | 2.34626006054788 | 0.000654158139634819 | 0.0100547079507961 | 3.18431725034749 | 0.019271948608137 | ST6GALNAC3//ST6GALNAC6//ST8SIA3//ST3GAL5//PIGA//SPHK2//B3GALT2//ST8SIA5//B3GNT5//PPM1L//SPTSSA//ELOVL4//ELOVL7//SGPP1//PRKAA1//ORMDL3//TNF//NSMAF// |
| GO:0010721 | negative regulation of cell development | Biological process | 30 | 301 | 934 | 17653 | 1.88376361450412 | 0.000664265532956189 | 0.0101869111097114 | 3.17765828140652 | 0.0321199143468951 | STMN2//LPAR1//EPHA4//MYLIP//PTPRG//SPOCK1//TSC1//BAG5//DLL1//HMG20A//DDX6//REST//EPHA7//ULK2//RGMA//PTEN//NOG//DUSP10//SEMA4B//WNT7A//SOX11//IGF1//EFNA1//LDLR//SLIT2//EFNA5//FBXW7//NRG1//STAT3//FRZB// |
| GO:0048738 | cardiac muscle tissue development | Biological process | 24 | 221 | 934 | 17653 | 2.05253519625607 | 0.000674539646268911 | 0.0103210670310331 | 3.17099251945419 | 0.0256959314775161 | NRG1//ZFPM2//TGFB2//POU4F1//NOG//S1PR1//ACTC1//GATA6//TSC1//EGLN1//FOXC1//HAND1//RARB//FHL2//GJA1//JARID2//PTEN//TGFBR2//ACVR1//MAP2K4//EDN1//IGF1//DLL1//GJC1// |
| GO:0003205 | cardiac chamber development | Biological process | 19 | 158 | 934 | 17653 | 2.27283631041119 | 0.000697521432997978 | 0.010648617271705 | 3.15644244312283 | 0.0203426124197002 | ZFPM2//GATA6//RARB//BMPR2//TGFB2//TGFBR2//NOG//SOS1//SOX4//SOX11//FOXF1//HAND1//HAND2//NRG1//POU4F1//ACVR1//FOXC1//FHL2//EGLN1// |
| GO:0000079 | regulation of cyclin-dependent protein serine/threonine kinase activity | Biological process | 13 | 88 | 934 | 17653 | 2.79210872104341 | 0.000704091191742137 | 0.0107247043462885 | 3.15237108871926 | 0.0139186295503212 | CDK6//PTEN//PKD2//CDKN1A//LATS2//IPO5//KAT2B//CCNY//MAPRE3//CCNC//CCND2//GADD45A//CCNE2// |
| GO:0048813 | dendrite morphogenesis | Biological process | 17 | 134 | 934 | 17653 | 2.39781552622327 | 0.000712504808043558 | 0.010828471947862 | 3.14721220065315 | 0.0182012847965739 | FMR1//NSMF//HECW2//FXR1//EPHA4//RAB21//CAPRIN2//CTNND2//PTEN//WNT7A//WASL//EFNA1//LRP8//HPRT1//MINK1//VLDLR//KLF7// |
| GO:0043405 | regulation of MAP kinase activity | Biological process | 33 | 344 | 934 | 17653 | 1.81312247896021 | 0.000718751443163447 | 0.0108877167851778 | 3.14342127029217 | 0.0353319057815846 | GADD45A//MINK1//TNF//MAP3K2//CRK//CRKL//MAP3K5//TAOK3//LAMTOR3//ADAM9//DUSP5//LPAR1//LPAR3//NRG1//IGF1//NTF3//PIK3CB//PRKAA1//MAPK1//MAP2K4//DUSP10//SPRED1//DUSP1//DUSP2//PPP2CA//RGS3//MDFIC//EDN1//FZD10//EPHA4//HIPK3//AIDA//TSG101// |
| GO:0043632 | modification-dependent macromolecule catabolic process | Biological process | 51 | 606 | 934 | 17653 | 1.59063010155405 | 0.000719622860117474 | 0.0108877167851778 | 3.1428950489602 | 0.0546038543897216 | ZFAND4//TOPORS//USP24//KLHL3//MYLIP//USP27X//ATXN3//RLIM//NPLOC4//USP28//USP46//UBE2I//BAG6//RNF128//KLHL15//USP45//CDC23//SQSTM1//USP3//UBXN4//DNAJB9//DERL2//JKAMP//WFS1//EDEM1//TGFB1I1//FBXL5//FBXW7//SKP1//BAG5//RNF180//ANKIB1//LONRF3//LONRF1//SOCS5//WWP2//UBXN2B//RNF38//RNF145//ZNRF2//CD2AP//SPOPL//UBE2W//HECW2//RNF4//TBL1XR1//PLAA//CLOCK//VPS37A//TSG101//PTEN// |
| GO:0009894 | regulation of catabolic process | Biological process | 71 | 914 | 934 | 17653 | 1.46819519349261 | 0.000721987675886146 | 0.0108991130625402 | 3.1414702156441 | 0.076017130620985 | NUP35//NUP43//TMF1//RRAGD//ULK2//MET//MTMR9//PIK3CB//PRKAA1//BNIP3//LDLR//SESN3//RAB12//SESN1//KDR//TSC1//SNX5//RHEB//LAMTOR3//EGLN1//BAG6//BAG5//RNF180//ANKIB1//LONRF3//LONRF1//SOCS5//MYLIP//NRG1//MDM4//DCP2//FMR1//YTHDF2//FXR1//WWP2//GJA1//NSF//HECW2//SOX17//TNF//STAT3//IGF1//E2F1//THRA//SH3D19//ADAM9//TOB1//CPEB3//CNOT7//BTG2//IDH1//CNOT6L//QKI//NANOS1//CALCOCO2//FYCO1//FBXW7//FEZ2//EFNA1//EPHA4//FBXL5//MSN//DERL2//EDEM1//ATXN3//RNF128//CTSA//PTEN//PIKFYVE//TBC1D12//RAB3GAP1// |
| GO:0097306 | cellular response to alcohol | Biological process | 10 | 57 | 934 | 17653 | 3.31586460798678 | 0.000732318647381094 | 0.0110304476887268 | 3.13529990707358 | 0.0107066381156317 | GLRA1//UGT1A1//PRKAA1//PTEN//PPARG//PTGER4//LRP6//LRP8//KLF2//KLF4// |
| GO:0032269 | negative regulation of cellular protein metabolic process | Biological process | 96 | 1316 | 934 | 17653 | 1.3787546455094 | 0.000755073407570463 | 0.0113479143453312 | 3.12201082459196 | 0.102783725910064 | DUSP10//SPRED1//DUSP1//DUSP2//DUSP5//PPP2CA//RGS3//FAM129A//IL2//LRP6//PTEN//PPP1R15B//SLIT2//ERRFI1//TSG101//SOCS5//GADD45A//PPM1E//FLRT1//GNAQ//NCK1//NF2//TAOK3//ASPN//DUS2//YWHAG//CAMK2N2//PPM1F//SNIP1//CALM2//SERPINI1//SPOCK1//RECK//TOB1//CPEB3//FMR1//CNOT7//NANOS1//CAPRIN2//TSC1//BTG2//FXR1//JARID2//SPOPL//SOX4//PER2//BAG5//CDK6//JUN//BAG6//CRY2//PPP1R37//MASTL//URI1//HMG20A//RNF4//CNOT6//STAT3//TNF//ARL6IP1//KLF4//FNIP1//EFNA1//HIPK3//AIDA//CDKN1A//LATS2//IPO5//KAT2B//PAIP2//ZMYND11//NTF3//DNMT3B//PMEPA1//LDLRAD4//NOG//NUP35//NUP43//PPARG//CNOT6L//YTHDF2//QKI//RANBP9//LMO3//PLAA//CPEB2//CRTAP//EPHA4//ESR1//DERL2//FRY//PRKAR2B//CPEB1//CPEB4//UNK//EDN1// |
| GO:0008104 | protein localization | Biological process | 174 | 2628 | 934 | 17653 | 1.25139821849364 | 0.000763371978030557 | 0.0114471944288706 | 3.11726378623924 | 0.186295503211991 | RANBP6//IPO5//TNF//DRD1//STAT3//BCL2L11//PMAIP1//MOAP1//PKD2//THRA//TWF1//ZFAND6//NUP35//AGFG1//NUP43//SRSF2//TSC1//PAN3//YWHAG//EGR2//ZDHHC1//ARL6IP1//RPL15//MTX3//IMMP2L//STX6//SEC23A//RAB12//STX2//RAB21//TBC1D12//RAB38//SNX5//ARFIP1//RAB30//NSF//SAR1B//RAB9B//DNAJC27//RAB23//TMED9//CTSA//RAB22A//KIF13A//SNX2//EVI5//TBC1D17//AP1S2//RAB28//ZFYVE9//USP6NL//G3BP2//RAPGEF4//MARCKS//NEUROD1//SNAP25//PER2//CLOCK//CBLN4//MIA3//TRIM3//EXOC5//VPS37A//DENND1B//LCA5//EPS15//NECAP1//SERP1//CHMP3//ATG16L1//UEVLD//ANKRD50//ZMAT3//AKTIP//BTF3//TSG101//PLEKHF2//FAM160A2//C5ORF30//MCFD2//PKDCC//ARRDC4//CEP41//GNPTG//PEX5L//DERL2//NPLOC4//GJA1//SOX4//TRAPPC8//MMD//FBXW7//WASL//CDKN1A//IGF1//MAPK1//MZT1//ARHGAP1//VLDLR//MTBP//TOPORS//PIKFYVE//CNEP1R1//FRMD6//LATS2//RRAGD//LAMTOR3//SYBU//MCOLN2//ITGAV//MDFIC//PPARG//LYPLA1//GOLGA4//C16ORF70//CLIP1//WNT7A//DZIP1//NPNT//PLEKHA1//INHBB//REST//CTDSPL2//PTGER4//TGFB2//ADAM9//IL2//WWP2//NDFIP2//MORC3//SKP1//EFNA5//EPHA5//RNF128//PLEK//ESCO2//ESR1//RAD21//NEDD1//BAG6//FCHO2//EFR3A//LRP6//P2RY1//MIEF1//RAB3GAP1//GLRB//NF2//CD2AP//DYNLL2//E2F1//PPP3R1//MSN//APPL1//AR//PPFIA1//EPHA2//SQSTM1//ARL5A//CCNE2//NPTN//NRG1//ELMOD1//PRKAA1//SNIP1//EDEM1//ATXN3//CCT6A//CENPQ//TNFRSF21//CD274//CEP128//MAPRE3//KIF5A// |
| GO:0014033 | neural crest cell differentiation | Biological process | 12 | 78 | 934 | 17653 | 2.9077581946961 | 0.000772022730599046 | 0.0115513047058437 | 3.11236991258083 | 0.012847965738758 | SEMA4B//ACVR1//HAND2//RDH10//EDN1//FOXC1//NRG1//SOX11//KBTBD8//LRP6//MAPK1//FRZB// |
| GO:0051497 | negative regulation of stress fiber assembly | Biological process | 6 | 22 | 934 | 17653 | 5.15466225423399 | 0.000774958448514575 | 0.0115696335260576 | 3.11072158273834 | 0.00642398286937901 | DLC1//S1PR1//TMEFF2//ARHGAP6//MET//PPFIA1// |
| GO:0030038 | contractile actin filament bundle assembly | Biological process | 13 | 89 | 934 | 17653 | 2.7607367129418 | 0.00078539506859216 | 0.0116739051623929 | 3.10491182994354 | 0.0139186295503212 | PTGER4//TSC1//LPAR1//PPM1E//NF2//PFN2//PPM1F//DLC1//S1PR1//TMEFF2//ARHGAP6//MET//PPFIA1// |
| GO:0043149 | stress fiber assembly | Biological process | 13 | 89 | 934 | 17653 | 2.7607367129418 | 0.00078539506859216 | 0.0116739051623929 | 3.10491182994354 | 0.0139186295503212 | PTGER4//TSC1//LPAR1//PPM1E//NF2//PFN2//PPM1F//DLC1//S1PR1//TMEFF2//ARHGAP6//MET//PPFIA1// |
| GO:0072176 | nephric duct development | Biological process | 5 | 15 | 934 | 17653 | 6.30014275517487 | 0.000789096271959939 | 0.0117031975597918 | 3.10287000841242 | 0.00535331905781585 | LHX1//PKD2//EPHA4//EPHA7//OSR1// |
| GO:0006935 | chemotaxis | Biological process | 50 | 594 | 934 | 17653 | 1.59094514019568 | 0.000804811642146446 | 0.0119101556582854 | 3.09430574987837 | 0.0535331905781585 | CCL2//CCL8//SLIT2//RANBP9//ARX//DPYSL2//EFNA1//EFNA5//EPHA5//GAB1//GRB2//KIF5A//OTX2//PIK3CB//ENAH//MAPK1//NTN4//SOS1//VLDLR//NR4A3//KLF7//RPS6KA5//FEZ2//EGR2//EPHA4//LHX1//NOG//CMKLR1//ADAM10//NRG1//TGFB2//S1PR1//EPHA7//KDR//BMPR2//SEMA4B//MET//NTF3//ITGAV//PPM1F//F3//VEGFB//LPAR1//EPHA2//CXCL14//BCL11B//NFIB//MPP1//EDN1//FOSL1// |
| GO:0060324 | face development | Biological process | 9 | 48 | 934 | 17653 | 3.54383029978587 | 0.000812335349648087 | 0.0119952488420743 | 3.09026464756208 | 0.00963597430406852 | RAB3GAP1//LRP6//MMP2//ASPH//PAX9//PLEKHA1//ARID5B//NOG//MAPK1// |
| GO:0003002 | regionalization | Biological process | 32 | 333 | 934 | 17653 | 1.81625737086123 | 0.000834510047518261 | 0.0122776530027063 | 3.0785684300387 | 0.0342612419700214 | FOXF1//FOXC1//DLL1//MEOX2//NEUROG1//PBX3//LHX1//RNF2//TSHZ1//CRKL//EMX2//HOXA3//LRP6//NEUROD1//BMPR2//BTG2//EDN1//EN1//DSCAML1//AIDA//WNT7A//NOG//GDF11//SOX17//TDRD5//EGR2//AR//OSR1//OTX2//PRKACB//SOSTDC1//ACVR1// |
| GO:0042330 | taxis | Biological process | 50 | 595 | 934 | 17653 | 1.5882712828172 | 0.000835090992347316 | 0.0122776530027063 | 3.07826620077828 | 0.0535331905781585 | CCL2//CCL8//SLIT2//CMKLR1//S1PR1//PIK3CB//MAPK1//FOSL1//CXCL14//RANBP9//ARX//DPYSL2//EFNA1//EFNA5//EPHA5//GAB1//GRB2//KIF5A//OTX2//ENAH//NTN4//SOS1//VLDLR//NR4A3//KLF7//RPS6KA5//FEZ2//EGR2//EPHA4//LHX1//NOG//ADAM10//NRG1//TGFB2//EPHA7//KDR//BMPR2//SEMA4B//MET//NTF3//ITGAV//PPM1F//F3//VEGFB//LPAR1//EPHA2//BCL11B//NFIB//MPP1//EDN1// |
| GO:0090066 | regulation of anatomical structure size | Biological process | 43 | 491 | 934 | 17653 | 1.65523098863047 | 0.00083853077551383 | 0.0122824817577388 | 3.07648099341501 | 0.0460385438972163 | ADRB1//EPS8//TWF1//CLCN6//SLC12A5//MTPN//MSN//GJA1//EDN1//PER2//DPYSL2//RAB21//EPHA4//PFN2//LIMA1//CFL2//PLEK//SLIT2//CDC42EP3//GRB2//NCK1//WNT7A//PTEN//IQGAP2//ARFIP1//DRD1//GOLGA4//NRG1//TSC1//PRR16//BMPR2//EPHA7//ULK2//EFNA5//LPAR3//SEMA4B//FOXC1//WASL//TMOD3//TMOD1//RAB22A//P2RY1//DOCK5// |
| GO:0090596 | sensory organ morphogenesis | Biological process | 26 | 251 | 934 | 17653 | 1.95781328646869 | 0.000840538992566893 | 0.0122824817577388 | 3.07544213485528 | 0.0278372591006424 | STAT3//SOX11//TFAP2A//TOPORS//FJX1//LHX1//LRIG1//NEUROG1//SOBP//FZD6//MAPK1//TSHZ1//OSR1//EDN1//NOG//RARB//GDF11//FOXF2//LRP6//ZEB1//NR4A3//DLL1//MAN2A1//TBC1D20//EPHA2//FRZB// |
| GO:0051961 | negative regulation of nervous system development | Biological process | 28 | 278 | 934 | 17653 | 1.90364025695931 | 0.000840867818103368 | 0.0122824817577388 | 3.0752722686255 | 0.0299785867237687 | STMN2//LPAR1//EPHA4//MYLIP//PTPRG//SPOCK1//TSC1//BAG5//TNFRSF21//PTEN//DLL1//HMG20A//DDX6//REST//EPHA7//ULK2//RGMA//NOG//DUSP10//SEMA4B//WNT7A//SOX11//EFNA1//LDLR//SLIT2//PRKACB//NRG1//STAT3// |
| GO:0003197 | endocardial cushion development | Biological process | 8 | 39 | 934 | 17653 | 3.87701092626146 | 0.00084681467846482 | 0.0123161455278658 | 3.07221162264008 | 0.00856531049250535 | TMEM100//TGFB2//ACVR1//NOG//TGFBR2//ERG//FOXF1//BMPR2// |
| GO:0061014 | positive regulation of mRNA catabolic process | Biological process | 8 | 39 | 934 | 17653 | 3.87701092626146 | 0.00084681467846482 | 0.0123161455278658 | 3.07221162264008 | 0.00856531049250535 | TOB1//CPEB3//CNOT7//BTG2//CNOT6L//YTHDF2//QKI//NANOS1// |
| GO:0090092 | regulation of transmembrane receptor protein serine/threonine kinase signaling pathway | Biological process | 24 | 225 | 934 | 17653 | 2.01604568165596 | 0.000869882660364175 | 0.012624498781208 | 3.06053932599428 | 0.0256959314775161 | GDF11//INHBB//BMP3//BMPR2//TGFB2//ACVR1//PMEPA1//LDLRAD4//DKK3//SOX11//ZEB1//NPNT//TGFB1I1//BAMBI//ASPN//PRDM16//RASL11B//TGFBR2//SULF1//GATA6//TOB1//SOSTDC1//CTDSPL2//NOG// |
| GO:0035303 | regulation of dephosphorylation | Biological process | 22 | 199 | 934 | 17653 | 2.0894945821183 | 0.000874841113040516 | 0.0126692729068373 | 3.05807081554401 | 0.0235546038543897 | TGFB2//TNF//NPNT//MTMR9//SPRED1//PPP1R37//PPP1R12A//PPP1R12C//PLEKHF2//URI1//PLEK//CRY2//MASTL//CALM2//PPP1R15B//DLC1//CNEP1R1//NSMF//UBXN2B//PPP4R2//TSC1//CHRM5// |
| GO:0071496 | cellular response to external stimulus | Biological process | 30 | 307 | 934 | 17653 | 1.84694738751055 | 0.000914990552610132 | 0.0132223955284238 | 3.03858339005428 | 0.0321199143468951 | DSC2//INHBB//RRAGD//BMPR2//KLF10//PLEKHF2//FNIP1//MAP3K2//MTPN//GADD45A//GJA1//IRF1//PTGER4//MAP2K4//BNIP3//CDKN1A//ITGA6//NR4A2//FOSL1//PRKAA1//SESN3//SESN1//MAP3K5//MAPK1//PMAIP1//CPEB4//DCTPP1//JUN//PPARG//TSC1// |
| GO:1900006 | positive regulation of dendrite development | Biological process | 11 | 69 | 934 | 17653 | 3.01311175247494 | 0.000933642066714956 | 0.0134631584161903 | 3.02981958869941 | 0.0117773019271949 | EPHA4//RAB21//CAPRIN2//LPAR1//CPEB3//FMR1//NRG1//IL2//C21ORF91//LRP8//VLDLR// |
| GO:0043086 | negative regulation of catalytic activity | Biological process | 65 | 829 | 934 | 17653 | 1.48193949005923 | 0.000947880681826732 | 0.0136393979812642 | 3.02324632773111 | 0.069593147751606 | DUSP10//SPRED1//DUSP1//DUSP2//DUSP5//PPP2CA//RGS3//ERRFI1//TSG101//SOCS5//GADD45A//PPM1E//FLRT1//GNAQ//LRP6//NCK1//NF2//TAOK3//ASPN//DUS2//YWHAG//CAMK2N2//PPM1F//TGFB2//TNF//PPP1R37//PPP1R12A//PPP1R12C//PLEKHF2//URI1//SERPINI1//SPOCK1//RECK//IQGAP2//FZD10//CPEB2//LRCH1//IPO5//TSC1//SLIT2//CDK6//PTEN//CRY2//MASTL//CNN3//PXK//ARL6IP1//KLF4//FNIP1//HIPK3//AIDA//CDKN1A//LATS2//KAT2B//EGLN1//BAG5//PPARG//MTRR//PRKAR2B//CHCHD10//FRY//UGT1A8//UGT1A7//UGT1A1//ANP32E// |
| GO:0043547 | positive regulation of GTPase activity | Biological process | 37 | 407 | 934 | 17653 | 1.71822075141133 | 0.000965366097952832 | 0.0138615093852548 | 3.01530795694734 | 0.0396145610278373 | EPHA2//TBC1D12//NTF3//TSC1//EVI5//TBC1D17//USP6NL//FAM13A//DLC1//CDC42EP3//GNB5//FZD10//ARAP2//TAGAP//TBC1D20//ADRB1//DENND1B//S1PR1//RAB3GAP1//DOCK9//RGS17//GNAQ//AGFG1//AGFG2//ITGA6//JUN//ARHGAP1//ARHGAP6//ERRFI1//ELMOD1//RGS3//CCL2//CCL8//SOS1//TRIP10//ARHGAP12//GIT2// |
| GO:0045667 | regulation of osteoblast differentiation | Biological process | 15 | 114 | 934 | 17653 | 2.48689845599008 | 0.000983811760609158 | 0.0140964384258469 | 3.00708799013496 | 0.0160599571734475 | TOB1//CDK6//TMEM64//TNF//NOG//HAND2//CEBPB//NPNT//GJA1//IGF1//FAM20C//BMPR2//SOX11//ACVR1//SMOC1// |
| GO:0120035 | regulation of plasma membrane bounded cell projection organization | Biological process | 50 | 602 | 934 | 17653 | 1.56980301208676 | 0.00107689685910552 | 0.0153387129230305 | 2.96782588970697 | 0.0535331905781585 | EPHA2//TWF1//SLIT2//PTEN//SNAP25//ZNF804A//STMN2//NPTN//NCK1//NME1//SERPINI1//LPAR1//EPHA4//MYLIP//PTPRG//SPOCK1//TSC1//BAG5//DPYSL2//RAB21//WNT7A//CDC42EP3//CEP97//GOLGA4//NRG1//EPHA7//ULK2//EFNA5//LPAR3//KLF4//RGMA//FMR1//NSMF//HECW2//FXR1//BMPR2//SEMA4B//EFNA1//POU3F2//CAPRIN2//GPM6A//WASL//CPEB3//IL2//C21ORF91//LRP8//VLDLR//PFN2//EPS8//PLAA// |
| GO:0007272 | ensheathment of neurons | Biological process | 15 | 115 | 934 | 17653 | 2.46527325202495 | 0.00107731607843257 | 0.0153387129230305 | 2.96765685846631 | 0.0160599571734475 | NRG1//CLDN11//KCNJ10//POU3F2//PPP3R1//RARB//TNFRSF21//PTEN//WASF3//PIKFYVE//LPAR1//EGR2//TSC1//SBF2//QKI// |
| GO:0008366 | axon ensheathment | Biological process | 15 | 115 | 934 | 17653 | 2.46527325202495 | 0.00107731607843257 | 0.0153387129230305 | 2.96765685846631 | 0.0160599571734475 | KCNJ10//POU3F2//PPP3R1//RARB//TNFRSF21//PTEN//WASF3//NRG1//PIKFYVE//LPAR1//EGR2//TSC1//SBF2//QKI//CLDN11// |
| GO:0018193 | peptidyl-amino acid modification | Biological process | 93 | 1282 | 934 | 17653 | 1.37109191005756 | 0.00108732719103697 | 0.0154183889200007 | 2.96363975133204 | 0.0995717344753747 | EREG//FBXW7//ERRFI1//TSG101//SOCS5//ASPH//MAPK1//CALM2//SPRED1//EPC2//CLOCK//TOPORS//NUP35//EGR2//NUP43//PIAS4//UBE2I//VKORC1L1//NAA30//KAT2B//CEP41//HIPK3//MORC3//LATS2//MKNK2//STK33//TGFBR2//MASTL//OSR1//ACVR1//EPHA2//EPHA4//EPHA5//EPHA7//DSTYK//NRG1//KDR//MET//MAP2K4//PKDCC//TMX3//ZDHHC1//ST6GALNAC3//ST6GALNAC6//ST3GAL3//ST6GAL2//ST3GAL5//MCFD2//BAG6//CRTAP//EGLN3//EGLN1//PRKAA1//NCK1//PPM1F//NTF3//OSM//PFN2//CAPRIN2//TNF//FNIP1//IFNA1//HMG20A//RNF4//ARID4B//ARID4A//PRDM16//RPS6KA4//RPS6KA5//HMGA2//GRB2//JAK1//CNOT7//IGF1//IL2//STAT3//NF2//PPP2CA//TADA1//DR1//DRD1//EFNA1//EFNA5//ITGA5//LRP8//DPY30//KMT2B//DNMT3B//MYB//JARID2//RAP2C//EZH1//SOX4// |
| GO:1901888 | regulation of cell junction assembly | Biological process | 12 | 81 | 934 | 17653 | 2.80006344674439 | 0.00108747176028986 | 0.0154183889200007 | 2.96358201208323 | 0.012847965738758 | EFNA5//SLK//KDR//TSC1//PPM1F//DLC1//ARHGAP6//PTEN//TNF//GJA1//CBFB//RAP1B// |
| GO:0003198 | epithelial to mesenchymal transition involved in endocardial cushion formation | Biological process | 5 | 16 | 934 | 17653 | 5.90638383297645 | 0.00109809076365065 | 0.0154716413220195 | 2.95936176141278 | 0.00535331905781585 | TGFB2//NOG//TGFBR2//ACVR1//TMEM100// |
| GO:1900151 | regulation of nuclear-transcribed mRNA catabolic process, deadenylation-dependent decay | Biological process | 5 | 16 | 934 | 17653 | 5.90638383297645 | 0.00109809076365065 | 0.0154716413220195 | 2.95936176141278 | 0.00535331905781585 | TOB1//CPEB3//CNOT7//BTG2//NANOS1// |
| GO:1900153 | positive regulation of nuclear-transcribed mRNA catabolic process, deadenylation-dependent decay | Biological process | 5 | 16 | 934 | 17653 | 5.90638383297645 | 0.00109809076365065 | 0.0154716413220195 | 2.95936176141278 | 0.00535331905781585 | TOB1//CPEB3//CNOT7//BTG2//NANOS1// |
| GO:0006893 | Golgi to plasma membrane transport | Biological process | 9 | 50 | 934 | 17653 | 3.40207708779443 | 0.00110401972155444 | 0.0155228386213569 | 2.95702316855812 | 0.00963597430406852 | LYPLA1//PKDCC//GOLGA4//NSF//KIF13A//C16ORF70//EXOC5//OSBPL5//STEAP2// |
| GO:1902532 | negative regulation of intracellular signal transduction | Biological process | 45 | 528 | 934 | 17653 | 1.61083195444812 | 0.00112263948516124 | 0.0157272834228852 | 2.94975968696396 | 0.0481798715203426 | DUSP10//SPRED1//DUSP1//DUSP2//DUSP5//PPP2CA//RGS3//PTEN//KLF4//PRKAA1//TSC1//FNIP1//DLC1//MET//NPLOC4//NF2//ZMYND11//ESR1//EFNA1//HIPK3//AIDA//LPAR1//TAOK3//FLRT1//NEUROD1//ASPN//SOCS5//TGFB2//PLEK//CD2AP//SLIT2//ARHGAP12//EPHA2//OTUD3//PLEKHA1//PKD2//CALM2//RANBP9//ERRFI1//LMO3//BAG5//WFS1//SESN3//SESN1//URI1// |
| GO:0051056 | regulation of small GTPase mediated signal transduction | Biological process | 32 | 339 | 934 | 17653 | 1.78411122270439 | 0.0011232112809779 | 0.0157272834228852 | 2.94953854335365 | 0.0342612419700214 | ARAP2//APPL1//AGFG1//AGFG2//PSD//GIT2//RALGPS2//CRK//FGD4//FGD5//EPS8//ARHGEF4//ARHGEF28//SOS1//SOS2//DLC1//MET//LPAR1//SQSTM1//RASGEF1A//NRG1//IGF1//TGFB2//CD2AP//SLIT2//ARHGAP12//FAM13A//TAGAP//RHOC//ARHGAP1//ARHGAP6//TRIP10// |
| GO:0001558 | regulation of cell growth | Biological process | 36 | 397 | 934 | 17653 | 1.71389273944304 | 0.00117438707866342 | 0.0164098756466957 | 2.93018873577954 | 0.0385438972162741 | ADAM10//MTPN//IL2//DERL2//TGFB2//CDKN1A//FRZB//GJA1//SERTAD3//PPARG//PPP2CA//RBBP7//SOX17//BMPR2//CAPRIN2//BTG1//ADIPOR2//SLIT2//DPYSL2//RAB21//EPHA4//GOLGA4//NRG1//EPHA7//ULK2//EFNA5//LPAR3//SEMA4B//EDN1//IGF1//PLAA//NANOS1//SPOCK1//TSG101//KIAA1109//URI1// |
| GO:0071375 | cellular response to peptide hormone stimulus | Biological process | 30 | 312 | 934 | 17653 | 1.81734887168506 | 0.00118350211646967 | 0.0164709954609438 | 2.92683096108067 | 0.0321199143468951 | GAB1//APPL1//GRB2//SOS1//PIK3R3//CPEB2//INHBB//ERRFI1//PPARG//PTEN//CPEB1//SLC2A4//YWHAG//KAT2B//SESN3//NCK1//TSC1//IGF1//STAT3//MAPK1//NR4A2//NR4A3//ADCY3//GJB2//PRKACB//PRKAR2B//RAP1B//CRK//PRKAA1//EDN1// |
| GO:0046677 | response to antibiotic | Biological process | 31 | 326 | 934 | 17653 | 1.79727998843946 | 0.00118363208546779 | 0.0164709954609438 | 2.92678327065508 | 0.0331905781584582 | EDN1//CRK//DUSP1//JUN//NR4A3//FOSL1//PPP1R15B//ADAM9//IL2//RPL15//SLC2A4//STAT3//ACTC1//EPS8//USP46//KLF2//ETS1//MAP3K5//MYB//PRKAA1//PLEKHA1//BNIP3//KLF4//GLRA1//UGT1A1//PTEN//MET//ADCY3//EFNA5//GJB2//JAK1// |
| GO:0001844 | protein insertion into mitochondrial membrane involved in apoptotic signaling pathway | Biological process | 7 | 32 | 934 | 17653 | 4.13446868308351 | 0.00119241076148435 | 0.0165114356293173 | 2.92357411312231 | 0.00749464668094218 | BCL2L11//DYNLL2//E2F1//PMAIP1//PPP3R1//YWHAG//MOAP1// |
| GO:0060317 | cardiac epithelial to mesenchymal transition | Biological process | 7 | 32 | 934 | 17653 | 4.13446868308351 | 0.00119241076148435 | 0.0165114356293173 | 2.92357411312231 | 0.00749464668094218 | TMEM100//EFNA1//ERG//TGFB2//NOG//TGFBR2//ACVR1// |
| GO:1904029 | regulation of cyclin-dependent protein kinase activity | Biological process | 13 | 93 | 934 | 17653 | 2.6419953489443 | 0.0011938624904238 | 0.0165114356293173 | 2.92304569254441 | 0.0139186295503212 | CDKN1A//GADD45A//PTEN//CCNE2//CDK6//PKD2//LATS2//IPO5//KAT2B//CCNY//MAPRE3//CCNC//CCND2// |
| GO:0060999 | positive regulation of dendritic spine development | Biological process | 8 | 41 | 934 | 17653 | 3.68788844205359 | 0.00119766135290723 | 0.0165301708769624 | 2.92166596437963 | 0.00856531049250535 | CAPRIN2//LRP8//LPAR1//CPEB3//FMR1//NRG1//IL2//C21ORF91// |
| GO:0007626 | locomotory behavior | Biological process | 21 | 191 | 934 | 17653 | 2.07805755798962 | 0.00121569138502164 | 0.0167108147091491 | 2.91517666091285 | 0.0224839400428266 | DRD1//EPHA4//GLRA1//GLRB//KCNJ10//SCN1A//EN1//EPS8//NR4A2//PBX3//TMOD1//TSC1//CCND2//PTEN//ARRDC3//NRG1//HPRT1//NOVA1//SOBP//SNAP25//STRN// |
| GO:0043401 | steroid hormone mediated signaling pathway | Biological process | 21 | 191 | 934 | 17653 | 2.07805755798962 | 0.00121569138502164 | 0.0167108147091491 | 2.91517666091285 | 0.0224839400428266 | ESR1//FHL2//AR//PMEPA1//RNF4//TGFB1I1//UBA5//CBFB//TCF21//CREBRF//CRY2//CLOCK//LMO3//PGRMC2//NR5A2//NR3C2//NR4A2//PPARG//RARB//THRA//NR4A3// |
| GO:0071902 | positive regulation of protein serine/threonine kinase activity | Biological process | 33 | 355 | 934 | 17653 | 1.75694121904877 | 0.00122656520926877 | 0.0168055535158298 | 2.91130935795676 | 0.0353319057815846 | GADD45A//MINK1//TNF//MAP3K2//CRK//CRKL//MAP3K5//TAOK3//LAMTOR3//ADAM9//DUSP5//LPAR1//LPAR3//NRG1//IGF1//NTF3//PIK3CB//PRKAA1//MAPK1//MAP2K4//COPS8//MDFIC//PKD2//EDN1//FZD10//EPHA4//CCNY//MAPRE3//CCNC//CCND2//ACSL1//PPP2CA//CALM2// |
| GO:0007517 | muscle organ development | Biological process | 36 | 398 | 934 | 17653 | 1.70958647627861 | 0.00122755336933608 | 0.0168055535158298 | 2.91095961732776 | 0.0385438972162741 | ZFPM2//NRG1//TGFB2//POU4F1//NOG//S1PR1//ZBTB18//CFL2//MEOX2//TCF21//MTPN//EGR2//FOXN2//MEF2D//SOX11//BTG2//GJA1//DLL1//NEUROG1//PRKAA1//SOX17//SERP1//ARID5B//EGLN1//ACTC1//FOXC1//HAND1//JARID2//PTEN//TGFBR2//GATA6//EDN1//IGF1//NLN//FXR1//SGCE// |
| GO:0046620 | regulation of organ growth | Biological process | 14 | 105 | 934 | 17653 | 2.52005710206995 | 0.00125801774711475 | 0.0171401729623206 | 2.90031323216608 | 0.0149892933618844 | PTEN//ARX//SERP1//GJA1//JARID2//TGFBR2//NOG//ZFPM2//GATA6//EDN1//IGF1//BCL2L11//FOXC1//LATS2// |
| GO:0001574 | ganglioside biosynthetic process | Biological process | 4 | 10 | 934 | 17653 | 7.56017130620985 | 0.00126466750084252 | 0.0171401729623206 | 2.89802364170588 | 0.00428265524625268 | ST6GALNAC3//ST6GALNAC6//ST8SIA3//ST3GAL5// |
| GO:0003183 | mitral valve morphogenesis | Biological process | 4 | 10 | 934 | 17653 | 7.56017130620985 | 0.00126466750084252 | 0.0171401729623206 | 2.89802364170588 | 0.00428265524625268 | EFNA1//BMPR2//SOX4//ACVR1// |
| GO:0003211 | cardiac ventricle formation | Biological process | 4 | 10 | 934 | 17653 | 7.56017130620985 | 0.00126466750084252 | 0.0171401729623206 | 2.89802364170588 | 0.00428265524625268 | HAND1//HAND2//SOX4//SOX11// |
| GO:1902237 | positive regulation of endoplasmic reticulum stress-induced intrinsic apoptotic signaling pathway | Biological process | 4 | 10 | 934 | 17653 | 7.56017130620985 | 0.00126466750084252 | 0.0171401729623206 | 2.89802364170588 | 0.00428265524625268 | BCL2L11//SERINC3//NCK1//PMAIP1// |
| GO:0003171 | atrioventricular valve development | Biological process | 6 | 24 | 934 | 17653 | 4.72510706638116 | 0.0012767759975824 | 0.0172188315253698 | 2.89388529032253 | 0.00642398286937901 | TGFB2//TGFBR2//ACVR1//EFNA1//BMPR2//SOX4// |
| GO:0042573 | retinoic acid metabolic process | Biological process | 6 | 24 | 934 | 17653 | 4.72510706638116 | 0.0012767759975824 | 0.0172188315253698 | 2.89388529032253 | 0.00642398286937901 | RDH10//UGT1A8//UGT1A7//UGT1A9//UGT1A1//UGT1A3// |
| GO:0035306 | positive regulation of dephosphorylation | Biological process | 9 | 51 | 934 | 17653 | 3.33536969391611 | 0.00127872609450252 | 0.0172188315253698 | 2.89322247224181 | 0.00963597430406852 | NPNT//MTMR9//PLEK//CALM2//PPP1R15B//DLC1//CNEP1R1//NSMF//PPP1R12A// |
| GO:0090287 | regulation of cellular response to growth factor stimulus | Biological process | 27 | 272 | 934 | 17653 | 1.87614545282781 | 0.00128065536851412 | 0.0172188315253698 | 2.89256772559452 | 0.0289079229122056 | DKK3//SOX11//ZEB1//NPNT//TGFB1I1//BAMBI//ASPN//PMEPA1//PRDM16//RASL11B//TGFBR2//LDLRAD4//SULF1//GATA6//BMPR2//TOB1//SOSTDC1//CTDSPL2//NOG//ITGA5//OTX2//FAM20C//SHISA2//DSTYK//NPTN//SLIT2//DLL1// |
| GO:0030326 | embryonic limb morphogenesis | Biological process | 16 | 129 | 934 | 17653 | 2.34423916471623 | 0.00128767642317232 | 0.0172446646532958 | 2.89019325598682 | 0.0171306209850107 | OSR1//RDH10//EN1//TFAP2A//WNT7A//RECK//RARB//FZD6//GJA1//GNAQ//NOG//HAND2//BCL2L11//LRP6//MBNL1//TGFB2// |
| GO:0035113 | embryonic appendage morphogenesis | Biological process | 16 | 129 | 934 | 17653 | 2.34423916471623 | 0.00128767642317232 | 0.0172446646532958 | 2.89019325598682 | 0.0171306209850107 | LRP6//MBNL1//TGFB2//OSR1//RDH10//EN1//TFAP2A//WNT7A//RECK//RARB//FZD6//GJA1//GNAQ//NOG//HAND2//BCL2L11// |
| GO:0055017 | cardiac muscle tissue growth | Biological process | 13 | 94 | 934 | 17653 | 2.6138890154449 | 0.0013198407929244 | 0.0176404807955488 | 2.87947845282783 | 0.0139186295503212 | S1PR1//FOXC1//TGFB2//GJA1//JARID2//PTEN//TGFBR2//NOG//ZFPM2//GATA6//MAP2K4//EDN1//IGF1// |
| GO:0050768 | negative regulation of neurogenesis | Biological process | 26 | 259 | 934 | 17653 | 1.89734028920324 | 0.00132849821147389 | 0.0177211704224811 | 2.87663902593383 | 0.0278372591006424 | STMN2//LPAR1//EPHA4//MYLIP//PTPRG//SPOCK1//TSC1//BAG5//DLL1//HMG20A//DDX6//REST//EPHA7//ULK2//RGMA//PTEN//NOG//DUSP10//SEMA4B//SOX11//EFNA1//LDLR//SLIT2//NRG1//STAT3//WNT7A// |
| GO:0045666 | positive regulation of neuron differentiation | Biological process | 32 | 343 | 934 | 17653 | 1.76330526092358 | 0.00136097933831655 | 0.0181187072146355 | 2.86614846798052 | 0.0342612419700214 | STMN2//NPTN//NCK1//NME1//SERPINI1//TWF1//ZNF804A//GOLGA4//NRG1//EFNA5//LPAR3//BMPR2//SLIT2//EPHA4//RAB21//CAPRIN2//LPAR1//CPEB3//FMR1//IL2//C21ORF91//LRP8//VLDLR//PLAA//DNMT3B//MMD//NEUROD1//NEUROG1//PTEN//RARB//SOX11//ZEB1// |
| GO:0030534 | adult behavior | Biological process | 17 | 142 | 934 | 17653 | 2.26272732756281 | 0.00136986663591924 | 0.0182011946143847 | 2.86332171175137 | 0.0182012847965739 | DRD1//EPHA4//GLRA1//GLRB//KCNJ10//SCN1A//USP46//EN1//EPS8//NR4A2//PBX3//TMOD1//TSC1//CCND2//RNF180//PTEN//NR4A3// |
| GO:0006977 | DNA damage response, signal transduction by p53 class mediator resulting in cell cycle arrest | Biological process | 10 | 62 | 934 | 17653 | 3.04845617185881 | 0.00143654760167542 | 0.0190124685521152 | 2.84267997858088 | 0.0107066381156317 | CDKN1A//GADD45A//E2F1//CNOT6L//CNOT7//MDM4//CNOT6//RBL2//SOX4//BTG2// |
| GO:0048645 | animal organ formation | Biological process | 10 | 62 | 934 | 17653 | 3.04845617185881 | 0.00143654760167542 | 0.0190124685521152 | 2.84267997858088 | 0.0107066381156317 | HAND2//RDH10//MAPK1//TGFBR2//NOG//AR//SULF1//SOX17//GATA6//HOXA3// |
| GO:0023056 | positive regulation of signaling | Biological process | 120 | 1743 | 934 | 17653 | 1.30123430399481 | 0.00146870730616703 | 0.0194001318586086 | 2.83306474470117 | 0.12847965738758 | GADD45A//MINK1//TNF//MAP3K2//CRK//CRKL//MAP3K5//TAOK3//LAMTOR3//ADAM9//DUSP5//LPAR1//LPAR3//NRG1//IGF1//NTF3//PIK3CB//PRKAA1//MAPK1//MAP2K4//EPS15//NSF//EREG//FBXW7//COPS8//MDFIC//TOB1//EPS8//ARHGAP1//ARHGAP6//F3//GDF11//INHBB//BMP3//BMPR2//TGFB2//ACVR1//KDR//EDN1//P2RY1//SOX11//SULF1//SOX4//NPNT//TGFB1I1//GATA6//ITGA5//PKD2//RRAGD//RHEB//GJA1//SERP1//AR//LMO3//SYBU//IL2//OSM//STAT3//RHOC//NDFIP2//CC2D1A//MIER1//SLC20A1//UBE2I//EFNA1//EPHA2//EPHA4//EPHA5//EPHA7//ITGAV//MET//FZD10//SPRED1//PMAIP1//ADRB1//SOS1//DSTYK//NPTN//DLL1//KAT2B//WNT7A//RASGEF1A//SOS2//ESR1//GAB1//GRB2//DRD1//TSHZ3//SLC24A2//PTEN//SNAP25//CALM2//RAB3GAP1//JUN//DNAJC27//CCL2//CCL8//HAND2//ARRDC3//BCL2L11//MOAP1//BNIP3//BAMBI//LRP6//CAPRIN2//TBL1XR1//PAIP2//SQSTM1//FMR1//DYNLL2//E2F1//PPP3R1//YWHAG//CREBRF//PIAS4//SERINC3//NCK1//MYB//TMF1//ST18// |
| GO:0060425 | lung morphogenesis | Biological process | 9 | 52 | 934 | 17653 | 3.27122796903311 | 0.00147504692388027 | 0.0194458915130648 | 2.831194163782 | 0.00963597430406852 | RDH10//FOXF1//TGFBR2//TNF//NFIB//MAPK1//SOX11//TCF21//NOG// |
| GO:0060572 | morphogenesis of an epithelial bud | Biological process | 5 | 17 | 934 | 17653 | 5.55894948986018 | 0.00148838238294859 | 0.0195835215094967 | 2.82728547909225 | 0.00535331905781585 | RDH10//NOG//AR//SOSTDC1//SULF1// |
| GO:0009755 | hormone-mediated signaling pathway | Biological process | 24 | 234 | 934 | 17653 | 1.93850546313073 | 0.00149611832648068 | 0.0196470839650269 | 2.82503405720322 | 0.0256959314775161 | THRA//ESR1//FHL2//AR//PMEPA1//RNF4//TGFB1I1//UBA5//CBFB//ACSL1//ADIPOR2//PGRMC2//NR5A2//NR3C2//NR4A2//PPARG//RARB//NR4A3//TCF21//CREBRF//CRY2//CLOCK//LMO3//LATS2// |
| GO:0007498 | mesoderm development | Biological process | 16 | 131 | 934 | 17653 | 2.30844925380453 | 0.00151630993953606 | 0.0198736513974465 | 2.81921201818127 | 0.0171306209850107 | NF2//BMPR2//NR4A3//ACVR1//NOG//HAND1//KLF4//ZFPM2//SOX17//HMGA2//FOXC1//FOXF1//OSR1//GDF11//POU4F1//PPP2CA// |
| GO:0010467 | gene expression | Biological process | 347 | 5761 | 934 | 17653 | 1.13842190733155 | 0.00152376678180156 | 0.0199327557936633 | 2.81708149830306 | 0.37152034261242 | E2F1//NPAT//RRM2//TSHZ1//HMG20A//KLF2//ZBTB18//CEBPB//ZNF268//TCERG1//WWP2//KLF12//OSR1//CRY2//GLIS3//ARX//DNMT3B//DR1//EDN1//EFNA1//CC2D1B//EN1//ESR1//FHL2//CPEB3//FOXF1//FOXC1//MYT1L//CIC//ZFPM2//DNAJB5//GATA6//CNOT7//HSBP1//AR//JARID2//JUN//ZNF568//MAF//MDM4//MYB//NFIA//NFIB//NR4A2//RLIM//KLF3//ZBTB7A//PIAS4//POU4F1//PPARG//CC2D1A//TSHZ3//ZBTB4//RARB//RBBP7//RBBP8//REST//RNF2//PRDM16//SOX17//SOX11//STAT3//ZEB1//TCF21//TFAP2A//TFAP2C//TGIF1//KLF10//TNF//UBE2I//VLDLR//WFS1//ZNF217//LRP8//BTG2//BHLHE41//TBL1XR1//NR4A3//HMGA2//ARID5B//BRMS1L//BHLHE40//RUNX3//URI1//PER2//SQSTM1//NOG//KLF4//HAND1//FNIP1//PHF14//USP3//DCP2//PPP2CA//RPL15//CNOT6L//PAN3//CNOT6//RBFOX3//FMR1//MBNL1//RBM25//SRSF2//ELAVL2//NOVA1//GTF2E1//SECISBP2L//F3//DUS2//IRF9//KLF6//CREBRF//EGR2//ELK3//ERG//FOXJ3//FOXF2//FLI1//GRHL1//HIVEP2//IRF1//LHX1//MEF2D//MEOX2//MYBL1//NEUROD1//NHLH2//OTX2//PAX9//PBX3//POU3F2//RAD21//ARID4A//SHOX//BCL11B//BMPR2//SOX4//BTF3//TEF//TGFB1I1//THRA//TMF1//DEK//FOSL1//KLF7//CBFB//FUBP3//HAND2//CLOCK//POLR3G//PKD2//BAHD1//H2AFZ//KMT2B//DPY30//ARID4B//TOPORS//KHDRBS3//ZMYND11//TADA1//ZNF597//CTNND2//ZNF782//NKX2-3//ZNF800//E2F5//ZNF367//EZH1//ZBTB9//KDM2A//MYCBP2//FLII//EPC2//LHX6//HBP1//ATAD2//SERTAD3//MDFIC//MNX1//HMGB3//HOXA3//HOXD1//FOXN2//ZBTB41//ZBTB34//ATXN3//LHX8//NEUROG1//NFE2//NPAS2//IER5//BRWD1//BNC2//ZNF532//CDCA7L//PRKAA1//TRERF1//LMO3//MAPK1//SLC2A4RG//PHTF2//ZNF630//ZNF287//ZNF512B//ZBTB26//MIER1//RBL2//RNF4//ATXN1//DMRTA1//SMARCD2//STAT4//ZNF2//ZNF3//ZNF131//ZNF655//BRD3//TCF7L1//PCGF5//LCOR//WASL//ZBTB47//ST18//HIPK3//NUP35//NR5A2//NRBF2//MLLT6//RNF141//PAK6//SNAPC1//BTG1//SNIP1//RPS6KA4//RPS6KA5//CDKN1A//CRK//EMX2//ETS1//BOLA3//NR3C2//PLEK//ZNFX1//NKX2-4//RFX7//BDP1//CDK8//E2F3//TEAD1//KAT2B//CCNC//CMTR2//ZFC3H1//RTCA//PPP4R2//ESRP1//CPEB1//QKI//AGFG1//NUP43//CPEB2//CPEB4//MTPN//DDX6//MKNK2//ACO1//TSC1//KBTBD8//IMMP2L//NAA30//ADAM10//G3BP2//EREG//TOB1//CDK6//TOB2//IGF1//PTHLH//TGFBR2//NANOS1//DRD1//RAB3GAP1//GJA1//ITGB8//LDLR//MSN//PIK3CB//PTEN//ACTC1//ZNF804A//PPM1F//KDR//NME1//NDFIP2//TGFB2//FAM129A//SERP1//PRR16//FXR1//INTS8//MAP3K2//MAPRE3//BAMBI//RGMB//LRP6//MAP3K5//WNT7A//ACVR1//DKK3//NRG1//RASD1//TSG101//BTAF1//CAPRIN2//SOSTDC1//TMEM100//RGMA//BMP3//SULF1//CTDSPL2//CMKLR1//EPHA5//NCK1//NABP1//EGLN1//FZD6//FASTK//AHNAK//YTHDF2//S1PR1//DLL1//IL2//ITGA6//MET//PPP1R12A//OSM//P2RY1//PPP3R1//DCAF6//PAIP2//EGLN3//PRKACB//ASPH//PPP1R15B//FBXW7//NPNT//UNK// |
| GO:0031344 | regulation of cell projection organization | Biological process | 50 | 612 | 934 | 17653 | 1.54415263607227 | 0.00152929374270093 | 0.0199664354862672 | 2.81550908843883 | 0.0535331905781585 | EPHA2//TWF1//SLIT2//PTEN//SNAP25//ZNF804A//STMN2//NPTN//NCK1//NME1//SERPINI1//LPAR1//EPHA4//MYLIP//PTPRG//SPOCK1//TSC1//BAG5//DPYSL2//RAB21//WNT7A//CDC42EP3//CEP97//GOLGA4//NRG1//EPHA7//ULK2//EFNA5//LPAR3//KLF4//RGMA//FMR1//NSMF//HECW2//FXR1//BMPR2//SEMA4B//EFNA1//POU3F2//CAPRIN2//GPM6A//WASL//CPEB3//IL2//C21ORF91//LRP8//VLDLR//PFN2//EPS8//PLAA// |
| GO:0006643 | membrane lipid metabolic process | Biological process | 23 | 221 | 934 | 17653 | 1.96701289641207 | 0.00153284953804858 | 0.0199742994717197 | 2.81450047267478 | 0.0246252676659529 | ITGB8//ST6GALNAC3//ST6GALNAC6//ST8SIA3//ST3GAL5//PIGA//KDELC1//SERINC3//SGPP1//SPHK2//PPP2CA//NSMAF//ORMDL3//B3GALT2//CTSA//ST8SIA5//B3GNT5//PPM1L//SPTSSA//ELOVL4//ELOVL7//PRKAA1//TNF// |
| GO:0031329 | regulation of cellular catabolic process | Biological process | 63 | 814 | 934 | 17653 | 1.46280955863397 | 0.00155038211237869 | 0.0201639119731098 | 2.80956125096155 | 0.0674518201284797 | NUP35//NUP43//TMF1//RRAGD//ULK2//MET//MTMR9//PIK3CB//PRKAA1//BNIP3//LDLR//SESN3//RAB12//SESN1//KDR//TSC1//SNX5//RHEB//LAMTOR3//EGLN1//BAG6//BAG5//RNF180//ANKIB1//LONRF3//LONRF1//SOCS5//MYLIP//DCP2//FMR1//YTHDF2//FXR1//STAT3//IGF1//E2F1//SH3D19//TNF//ADAM9//TOB1//CPEB3//CNOT7//BTG2//IDH1//CNOT6L//QKI//NANOS1//CALCOCO2//FYCO1//FBXW7//FEZ2//EFNA1//EPHA4//FBXL5//MSN//DERL2//EDEM1//ATXN3//RNF128//CTSA//PTEN//PIKFYVE//TBC1D12//RAB3GAP1// |
| GO:0016049 | cell growth | Biological process | 41 | 477 | 934 | 17653 | 1.62456511296962 | 0.00156056145507271 | 0.020229816826896 | 2.80671912409994 | 0.0438972162740899 | NANOS1//SPOCK1//TSG101//KIAA1109//URI1//TGFBR2//ADAM10//MTPN//IL2//DERL2//TGFB2//CDKN1A//FRZB//GJA1//SERTAD3//PPARG//PPP2CA//RBBP7//SOX17//BMPR2//CAPRIN2//BTG1//ADIPOR2//SLIT2//DPYSL2//RAB21//EPHA4//GOLGA4//NRG1//EFNA5//EPHA7//ULK2//LPAR3//SEMA4B//MAP2K4//EDN1//IGF1//PLAA//FLRT1//AR//SOCS5// |
| GO:1902533 | positive regulation of intracellular signal transduction | Biological process | 76 | 1021 | 934 | 17653 | 1.40688790223298 | 0.00156143196564242 | 0.020229816826896 | 2.80647693400168 | 0.0813704496788009 | GADD45A//MINK1//TNF//MAP3K2//CRK//CRKL//MAP3K5//TAOK3//LAMTOR3//ADAM9//DUSP5//LPAR1//LPAR3//NRG1//IGF1//NTF3//PIK3CB//PRKAA1//MAPK1//MAP2K4//COPS8//MDFIC//KDR//TGFB2//PKD2//RRAGD//RHEB//SOX11//IL2//OSM//STAT3//GJA1//RHOC//NDFIP2//CC2D1A//MIER1//SLC20A1//UBE2I//EDN1//EFNA1//EPHA2//EPHA4//EPHA5//EPHA7//AR//ITGAV//MET//FZD10//SPRED1//PMAIP1//ADRB1//WNT7A//RASGEF1A//SOS1//SOS2//EREG//ESR1//F3//GAB1//GRB2//CALM2//NPNT//DSTYK//NPTN//JUN//P2RY1//DNAJC27//FBXW7//PTEN//CCL2//CCL8//HAND2//PIAS4//BCL2L11//SERINC3//NCK1// |
| GO:0043410 | positive regulation of MAPK cascade | Biological process | 46 | 552 | 934 | 17653 | 1.57503568879372 | 0.00156870944363822 | 0.0202852427673524 | 2.80445748898799 | 0.0492505353319058 | GADD45A//MINK1//TNF//MAP3K2//CRK//CRKL//MAP3K5//TAOK3//LAMTOR3//ADAM9//DUSP5//LPAR1//LPAR3//NRG1//IGF1//NTF3//PIK3CB//PRKAA1//MAPK1//MAP2K4//MDFIC//TGFB2//EDN1//FZD10//EPHA4//WNT7A//EPHA2//EPHA5//EPHA7//NPNT//DSTYK//NPTN//JUN//KDR//MET//P2RY1//DNAJC27//FBXW7//PTEN//CCL2//CCL8//HAND2//EFNA1//AR//ITGAV//OSM// |
| GO:0003148 | outflow tract septum morphogenesis | Biological process | 6 | 25 | 934 | 17653 | 4.53610278372591 | 0.00160562429023307 | 0.0206834991901833 | 2.7943560703696 | 0.00642398286937901 | ZFPM2//GATA6//RARB//BMPR2//TGFB2//TGFBR2// |
| GO:0032232 | negative regulation of actin filament bundle assembly | Biological process | 6 | 25 | 934 | 17653 | 4.53610278372591 | 0.00160562429023307 | 0.0206834991901833 | 2.7943560703696 | 0.00642398286937901 | DLC1//S1PR1//TMEFF2//ARHGAP6//MET//PPFIA1// |
| GO:0072431 | signal transduction involved in mitotic G1 DNA damage checkpoint | Biological process | 10 | 63 | 934 | 17653 | 3.0000679786547 | 0.00162820810825424 | 0.0208760664081561 | 2.78829008686066 | 0.0107066381156317 | CDKN1A//GADD45A//E2F1//CNOT6L//CNOT7//MDM4//CNOT6//RBL2//SOX4//BTG2// |
| GO:1902400 | intracellular signal transduction involved in G1 DNA damage checkpoint | Biological process | 10 | 63 | 934 | 17653 | 3.0000679786547 | 0.00162820810825424 | 0.0208760664081561 | 2.78829008686066 | 0.0107066381156317 | CDKN1A//GADD45A//E2F1//CNOT6L//CNOT7//MDM4//CNOT6//RBL2//SOX4//BTG2// |
| GO:0051962 | positive regulation of nervous system development | Biological process | 42 | 493 | 934 | 17653 | 1.61017847292502 | 0.0016298333673675 | 0.0208760664081561 | 2.78785679518627 | 0.0449678800856531 | STMN2//NPTN//NCK1//NME1//SERPINI1//TWF1//ZNF804A//LHX1//WASF3//NRG1//DNMT3B//MMD//NEUROD1//NEUROG1//PTEN//RARB//SOX11//ZEB1//GOLGA4//EFNA5//LPAR3//PPARG//RHEB//BMPR2//MAN2A1//SLIT2//EPHA4//RAB21//CAPRIN2//SLITRK3//FLRT1//WNT7A//MYB//LPAR1//CPEB3//FMR1//IL2//C21ORF91//LRP8//VLDLR//PLAA//NSMF// |
| GO:0042475 | odontogenesis of dentin-containing tooth | Biological process | 12 | 85 | 934 | 17653 | 2.66829575513289 | 0.00166783060176767 | 0.0212821478485939 | 2.77784806188385 | 0.012847965738758 | FAM20C//ITGA6//BCL2L11//NKX2-3//FOXC1//SOSTDC1//LRP6//LHX8//NF2//BCL11B//HAND1//HAND2// |
| GO:0043506 | regulation of JUN kinase activity | Biological process | 12 | 85 | 934 | 17653 | 2.66829575513289 | 0.00166783060176767 | 0.0212821478485939 | 2.77784806188385 | 0.012847965738758 | MAP3K2//MDFIC//MAP3K5//MAP2K4//FZD10//EDN1//EPHA4//TAOK3//TNF//HIPK3//DUSP10//AIDA// |
| GO:0001707 | mesoderm formation | Biological process | 11 | 74 | 934 | 17653 | 2.80952312055096 | 0.00168326447968219 | 0.021398341496411 | 2.7738476409806 | 0.0117773019271949 | KLF4//SOX17//HMGA2//FOXC1//FOXF1//NF2//BMPR2//NR4A3//ACVR1//NOG//HAND1// |
| GO:0048864 | stem cell development | Biological process | 11 | 74 | 934 | 17653 | 2.80952312055096 | 0.00168326447968219 | 0.021398341496411 | 2.7738476409806 | 0.0117773019271949 | SEMA4B//ACVR1//HAND2//RDH10//EDN1//FOXC1//NRG1//SOX11//KBTBD8//MAPK1//WNT7A// |
| GO:1901654 | response to ketone | Biological process | 20 | 183 | 934 | 17653 | 2.06562057546717 | 0.0016895156545844 | 0.0214375128929724 | 2.77223777981798 | 0.0214132762312634 | GJB2//TGFB2//FOSL1//DUSP1//EDN1//NME1//CDKN1A//SLIT2//PPARG//PRKAA1//PTGER4//AR//MSN//KLF2//KLF4//FIBIN//DNMT3B//ERRFI1//ADCY3//EFNA5// |
| GO:0043243 | positive regulation of protein complex disassembly | Biological process | 7 | 34 | 934 | 17653 | 3.89126464290213 | 0.0017380481035304 | 0.0219708772414506 | 2.75993820785933 | 0.00749464668094218 | CFL2//PLEK//STMN2//CALCOCO2//FYCO1//BNIP3//TNF// |
| GO:0061384 | heart trabecula morphogenesis | Biological process | 7 | 34 | 934 | 17653 | 3.89126464290213 | 0.0017380481035304 | 0.0219708772414506 | 2.75993820785933 | 0.00749464668094218 | NRG1//TGFB2//FHL2//EGLN1//S1PR1//SOS1//NOG// |
| GO:0002062 | chondrocyte differentiation | Biological process | 13 | 97 | 934 | 17653 | 2.53304708713217 | 0.00176579948584837 | 0.0222800409007323 | 2.75305861397118 | 0.0139186295503212 | SULF1//BMPR2//TGFBR2//MAF//PTHLH//RARB//PKDCC//OSR1//MEF2D//NFIB//WNT7A//HMGA2//RUNX3// |
| GO:0003231 | cardiac ventricle development | Biological process | 15 | 121 | 934 | 17653 | 2.34302829737909 | 0.00180984759112577 | 0.0227932947090942 | 2.74235799591533 | 0.0160599571734475 | TGFB2//TGFBR2//NOG//SOX4//SOX11//FOXF1//HAND1//HAND2//ZFPM2//NRG1//POU4F1//FOXC1//EGLN1//BMPR2//ACVR1// |
| GO:0060389 | pathway-restricted SMAD protein phosphorylation | Biological process | 10 | 64 | 934 | 17653 | 2.95319191648822 | 0.00184006615277288 | 0.0231307944074405 | 2.73516656325797 | 0.0107066381156317 | GDF11//INHBB//BMP3//BMPR2//TGFB2//ACVR1//PMEPA1//LDLRAD4//NOG//TGFBR2// |
| GO:0048661 | positive regulation of smooth muscle cell proliferation | Biological process | 12 | 86 | 934 | 17653 | 2.63726906030576 | 0.0018470616260076 | 0.0231327366234989 | 2.73351861436563 | 0.012847965738758 | IGF1//JUN//MEF2D//MAP3K5//MMP2//TNF//S1PR1//EDN1//EREG//MYB//TGFBR2//NR4A3// |
| GO:1901655 | cellular response to ketone | Biological process | 12 | 86 | 934 | 17653 | 2.63726906030576 | 0.0018470616260076 | 0.0231327366234989 | 2.73351861436563 | 0.012847965738758 | PPARG//PRKAA1//PTGER4//AR//MSN//KLF2//KLF4//DNMT3B//GJB2//ERRFI1//ADCY3//EFNA5// |
| GO:0016482 | cytosolic transport | Biological process | 17 | 146 | 934 | 17653 | 2.20073479804054 | 0.00185628723049402 | 0.0232053059885971 | 2.73135482285469 | 0.0182012847965739 | EPS15//SNX2//STX6//PIKFYVE//TMEM87A//SNX5//RAB9B//EVI5//ZFYVE21//TBC1D17//AKTIP//FAM160A2//SYBU//CHMP3//MAPK1//RAB21//MSN// |
| GO:0098876 | vesicle-mediated transport to the plasma membrane | Biological process | 11 | 75 | 934 | 17653 | 2.77206281227695 | 0.00188028773402371 | 0.0234619666885652 | 2.72577568704639 | 0.0117773019271949 | EXOC5//OSBPL5//STEAP2//LYPLA1//PKDCC//GOLGA4//NSF//KIF13A//C16ORF70//SNAP25//ANKRD50// |
| GO:0051129 | negative regulation of cellular component organization | Biological process | 52 | 649 | 934 | 17653 | 1.51436405209134 | 0.00188921809990536 | 0.023529985284825 | 2.72371790225934 | 0.0556745182012848 | EPS8//TWF1//STMN2//MID1IP1//SLIT2//BNIP3//LPAR1//EPHA4//MYLIP//PTPRG//SPOCK1//TSC1//BAG5//PMEPA1//LDLRAD4//THRA//LIMA1//PFN2//JARID2//ATAD2//DCP2//PTGER4//TNF//CEP97//TMEFF2//AR//MTBP//RAD21//MDM1//KAT2B//EPHA7//ULK2//RGMA//PTEN//SEMA4B//DLC1//S1PR1//ARHGAP6//MET//PPFIA1//DNMT3B//TMOD3//TMOD1//EFNA1//SACS//IGF1//DUSP1//EFNA5//FEZ2//WASL//LDLR//MTPN// |
| GO:0003174 | mitral valve development | Biological process | 4 | 11 | 934 | 17653 | 6.87288300564532 | 0.00190447821074116 | 0.0235719081566019 | 2.72022399176597 | 0.00428265524625268 | EFNA1//BMPR2//SOX4//ACVR1// |
| GO:0060211 | regulation of nuclear-transcribed mRNA poly(A) tail shortening | Biological process | 4 | 11 | 934 | 17653 | 6.87288300564532 | 0.00190447821074116 | 0.0235719081566019 | 2.72022399176597 | 0.00428265524625268 | TOB1//CPEB3//CNOT7//BTG2// |
| GO:0060213 | positive regulation of nuclear-transcribed mRNA poly(A) tail shortening | Biological process | 4 | 11 | 934 | 17653 | 6.87288300564532 | 0.00190447821074116 | 0.0235719081566019 | 2.72022399176597 | 0.00428265524625268 | TOB1//CPEB3//CNOT7//BTG2// |
| GO:0035966 | response to topologically incorrect protein | Biological process | 21 | 198 | 934 | 17653 | 2.00459087664655 | 0.00190652576691723 | 0.0235719081566019 | 2.71975732081027 | 0.0224839400428266 | DNAJB4//UBXN4//DNAJB5//JKAMP//HERPUD2//HSPA13//CREBRF//SERP1//DERL2//DNAJB9//WFS1//EDEM1//CCL2//ATXN3//DNAJB12//UBE2W//KLHL15//BAG6//BCL2L11//NCK1//PPP1R15B// |
| GO:0031018 | endocrine pancreas development | Biological process | 8 | 44 | 934 | 17653 | 3.43644150282266 | 0.00193117567049264 | 0.0238331041232513 | 2.71417821857924 | 0.00856531049250535 | GATA6//CDK6//DLL1//NEUROD1//RHEB//CLOCK//MNX1//SOX4// |
| GO:0002011 | morphogenesis of an epithelial sheet | Biological process | 9 | 54 | 934 | 17653 | 3.15007137758744 | 0.00194016382007056 | 0.0239004151459694 | 2.71216159834352 | 0.00963597430406852 | LRP6//SOS1//ITGA5//WNT7A//TMEFF2//RHOC//SOX11//PTEN//ARHGAP12// |
| GO:0010498 | proteasomal protein catabolic process | Biological process | 39 | 453 | 934 | 17653 | 1.62718918842265 | 0.00194927619540842 | 0.0239690089264494 | 2.71012662078825 | 0.0417558886509636 | TMF1//UBXN4//DNAJB9//DERL2//JKAMP//NPLOC4//WFS1//BAG6//EDEM1//CDC23//FBXL5//FBXW7//SKP1//BAG5//RNF180//ANKIB1//LONRF3//LONRF1//SOCS5//DNAJB12//TOPORS//WWP2//UBXN2B//RNF38//RNF145//ZNRF2//CD2AP//SPOPL//ATXN3//RLIM//UBE2W//HECW2//RNF4//TBL1XR1//PLAA//CLOCK//KLHL15//FMR1//PMAIP1// |
| GO:0001573 | ganglioside metabolic process | Biological process | 5 | 18 | 934 | 17653 | 5.25011896264573 | 0.00197187870786694 | 0.024202932307267 | 2.70511980243951 | 0.00535331905781585 | ST6GALNAC3//ST6GALNAC6//ST8SIA3//ST3GAL5//ITGB8// |
| GO:0035883 | enteroendocrine cell differentiation | Biological process | 6 | 26 | 934 | 17653 | 4.36163729204414 | 0.001995044990771 | 0.0244429153488846 | 2.70004730598061 | 0.00642398286937901 | GATA6//CDK6//DLL1//NEUROD1//RHEB//CLOCK// |
| GO:0019216 | regulation of lipid metabolic process | Biological process | 33 | 366 | 934 | 17653 | 1.70413697476042 | 0.00202267065889405 | 0.0247365672081383 | 2.69407482541943 | 0.0353319057815846 | CNEP1R1//LDLR//FBXW7//DKK3//REST//ADIPOR2//NRBF2//RBL2//PIK3R3//KLF4//PRKAA1//MID1IP1//UGT1A8//UGT1A1//PPARG//NR4A3//THRA//TNF//CHRM5//MTMR9//IDH1//TBL1XR1//ORMDL3//NSMAF//ACSL1//FHL2//RGL1//TNFRSF21//GRHL1//NPAS2//ACOX1//UGT1A9//BCL11B// |
| GO:0006672 | ceramide metabolic process | Biological process | 12 | 87 | 934 | 17653 | 2.60695562283098 | 0.00204179328898838 | 0.0248804468710422 | 2.68998822796697 | 0.012847965738758 | ITGB8//ST6GALNAC3//ST6GALNAC6//ST8SIA3//ST3GAL5//B3GALT2//PRKAA1//SPTSSA//ORMDL3//TNF//NSMAF//PPP2CA// |
| GO:0048644 | muscle organ morphogenesis | Biological process | 12 | 87 | 934 | 17653 | 2.60695562283098 | 0.00204179328898838 | 0.0248804468710422 | 2.68998822796697 | 0.012847965738758 | ZFPM2//NRG1//TGFB2//POU4F1//NOG//S1PR1//EGLN1//ACTC1//FOXC1//HAND1//SERP1//ARID5B// |
| GO:0009605 | response to external stimulus | Biological process | 152 | 2306 | 934 | 17653 | 1.24582181108402 | 0.00207267735931659 | 0.0249685405595977 | 2.68346829654278 | 0.162740899357602 | CRK//GLRA1//GLRB//NRG1//TBL1XR1//ADRB1//F3//CCL2//CCL8//PPARG//OSM//SLIT2//TNF//CEBPB//TMF1//CDK6//CHRM2//FOSL1//HMGA2//URI1//CMKLR1//S1PR1//PIK3CB//MAPK1//CXCL14//CLCN6//ETS1//JUN//P2RY1//PTGER4//TGFBR2//BTG2//RANBP9//ARX//DPYSL2//EFNA1//EFNA5//EPHA5//GAB1//GRB2//KIF5A//OTX2//ENAH//NTN4//SOS1//VLDLR//NR4A3//KLF7//RPS6KA5//FEZ2//ACSL1//UGT1A1//ADIPOR2//CDS1//TRPC3//GNAQ//EGR2//EPHA4//LHX1//NOG//DSC2//INHBB//RRAGD//BMPR2//KLF10//PLEKHF2//FNIP1//ELOVL4//SERINC3//NCK1//WASL//PLEK//ADAM10//FOXF1//MAP3K2//MTPN//GADD45A//GJA1//IRF1//MAP2K4//BNIP3//ENPP4//EDN1//TGFB2//NEUROG1//EPHA7//IGF1//CDKN1A//ITGA6//NR4A2//PRKAA1//TMEM135//DUSP10//GJB2//TNFRSF21//DNMT3B//SRSF2//PTEN//SESN3//SESN1//MAP3K5//RAB38//KDR//NPLOC4//PMAIP1//CPEB4//DCTPP1//ULK2//CRY2//BHLHE40//PER2//KLF4//RGMA//SEMA4B//ESR1//IL2//SOCS5//CDK19//LDLR//CLOCK//MET//NTF3//ITGAV//PPM1F//VEGFB//SCN1A//DENND5B//PKD2//IRF9//POLR3G//ZMYND11//CNOT7//IFNA1//USP46//DRD1//BCL2L11//LPAR1//EPHA2//CD274//PLSCR4//PDCD1LG2//ADAM9//PLAA//BCL11B//NFIB//MPP1//TSC1//NPNT//FMR1//CALCOCO2//ATG16L1//C5ORF30// |
| GO:0072413 | signal transduction involved in mitotic cell cycle checkpoint | Biological process | 10 | 65 | 934 | 17653 | 2.9077581946961 | 0.00207364118258161 | 0.0249685405595977 | 2.68326639062126 | 0.0107066381156317 | CDKN1A//GADD45A//E2F1//CNOT6L//CNOT7//MDM4//CNOT6//RBL2//SOX4//BTG2// |
| GO:1902402 | signal transduction involved in mitotic DNA damage checkpoint | Biological process | 10 | 65 | 934 | 17653 | 2.9077581946961 | 0.00207364118258161 | 0.0249685405595977 | 2.68326639062126 | 0.0107066381156317 | CDKN1A//GADD45A//E2F1//CNOT6L//CNOT7//MDM4//CNOT6//RBL2//SOX4//BTG2// |
| GO:1902403 | signal transduction involved in mitotic DNA integrity checkpoint | Biological process | 10 | 65 | 934 | 17653 | 2.9077581946961 | 0.00207364118258161 | 0.0249685405595977 | 2.68326639062126 | 0.0107066381156317 | CDKN1A//GADD45A//E2F1//CNOT6L//CNOT7//MDM4//CNOT6//RBL2//SOX4//BTG2// |
| GO:0040036 | regulation of fibroblast growth factor receptor signaling pathway | Biological process | 7 | 35 | 934 | 17653 | 3.78008565310492 | 0.00207486615325949 | 0.0249685405595977 | 2.68300991378355 | 0.00749464668094218 | SULF1//SHISA2//DSTYK//NPTN//NOG//OTX2//FAM20C// |
| GO:0051204 | protein insertion into mitochondrial membrane | Biological process | 7 | 35 | 934 | 17653 | 3.78008565310492 | 0.00207486615325949 | 0.0249685405595977 | 2.68300991378355 | 0.00749464668094218 | BCL2L11//PMAIP1//MOAP1//DYNLL2//E2F1//PPP3R1//YWHAG// |
| GO:2000826 | regulation of heart morphogenesis | Biological process | 7 | 35 | 934 | 17653 | 3.78008565310492 | 0.00207486615325949 | 0.0249685405595977 | 2.68300991378355 | 0.00749464668094218 | BMPR2//HAND2//TGFB2//FOXC1//NOG//TGFBR2//ACVR1// |
| GO:0048332 | mesoderm morphogenesis | Biological process | 11 | 76 | 934 | 17653 | 2.73558830158909 | 0.00209568519114176 | 0.0251401181950109 | 2.67867395547568 | 0.0117773019271949 | NF2//BMPR2//NR4A3//ACVR1//NOG//HAND1//KLF4//SOX17//HMGA2//FOXC1//FOXF1// |
| GO:1901360 | organic cyclic compound metabolic process | Biological process | 380 | 6394 | 934 | 17653 | 1.12326599013127 | 0.00209655872571139 | 0.0251401181950109 | 2.67849296827343 | 0.406852248394004 | IGF1//NFIA//NFIB//RBBP7//RBBP8//RRM2//E2F1//NPAT//TSHZ1//HMG20A//KLF2//ZBTB18//CEBPB//ZNF268//TCERG1//WWP2//KLF12//OSR1//CRY2//GLIS3//ARX//DNMT3B//DR1//EDN1//EFNA1//CC2D1B//EN1//ESR1//FHL2//CPEB3//FOXF1//FOXC1//MYT1L//CIC//ZFPM2//DNAJB5//GATA6//CNOT7//HSBP1//AR//JARID2//JUN//ZNF568//MAF//MDM4//MYB//NR4A2//RLIM//KLF3//ZBTB7A//PIAS4//POU4F1//PPARG//CC2D1A//TSHZ3//ZBTB4//RARB//REST//RNF2//PRDM16//SOX17//SOX11//STAT3//ZEB1//TCF21//TFAP2A//TFAP2C//TGIF1//KLF10//TNF//UBE2I//VLDLR//WFS1//ZNF217//LRP8//BTG2//BHLHE41//TBL1XR1//NR4A3//HMGA2//ARID5B//BRMS1L//BHLHE40//RUNX3//URI1//PER2//SQSTM1//NOG//KLF4//HAND1//FNIP1//PHF14//USP3//DCP2//PPP2CA//RPL15//CNOT6L//PAN3//CNOT6//RBFOX3//FMR1//MBNL1//RBM25//SRSF2//ELAVL2//NOVA1//COPS8//PTGES3//CCNE2//NABP1//GTF2E1//DUS2//IRF9//KLF6//CREBRF//EGR2//ELK3//ERG//FOXJ3//FOXF2//FLI1//GRHL1//HIVEP2//IRF1//LHX1//MEF2D//MEOX2//MYBL1//NEUROD1//NHLH2//OTX2//PAX9//PBX3//POU3F2//RAD21//ARID4A//SHOX//BCL11B//BMPR2//SOX4//BTF3//TEF//TGFB1I1//THRA//TMF1//DEK//FOSL1//KLF7//CBFB//FUBP3//HAND2//CLOCK//POLR3G//PKD2//UGP2//GFPT2//NAGK//SLC35D1//NUP35//NUP43//CHCHD10//COX8C//COX7A2L//DPYSL2//TTR//HPRT1//NME1//CMPK1//ADCY3//IMPDH1//PDE10A//DCTPP1//REV3L//GMNC//ESCO2//GADD45A//EPC2//TAOK3//UBE2W//USP28//USP45//ATXN3//KDM2A//MTRR//HMGB3//BAHD1//H2AFZ//KMT2B//DPY30//ARID4B//TOPORS//KHDRBS3//ZMYND11//TADA1//ZNF597//CTNND2//ZNF782//NKX2-3//ZNF800//E2F5//ZNF367//EZH1//ZBTB9//MYCBP2//FLII//LHX6//HBP1//ATAD2//SERTAD3//MDFIC//MNX1//HOXA3//HOXD1//FOXN2//ZBTB41//ZBTB34//LHX8//NEUROG1//NFE2//NPAS2//IER5//BRWD1//BNC2//ZNF532//CDCA7L//PRKAA1//TRERF1//LMO3//MAPK1//SLC2A4RG//PHTF2//ZNF630//ZNF287//ZNF512B//ZBTB26//MIER1//RBL2//RNF4//ATXN1//DMRTA1//SMARCD2//STAT4//ZNF2//ZNF3//ZNF131//ZNF655//BRD3//TCF7L1//PCGF5//LCOR//WASL//ZBTB47//ST18//HIPK3//NR5A2//NRBF2//MLLT6//RNF141//PAK6//SNAPC1//BTG1//SNIP1//RPS6KA4//RPS6KA5//CDKN1A//CRK//EMX2//ETS1//BOLA3//NR3C2//PLEK//ZNFX1//NKX2-4//RFX7//BDP1//CDK8//E2F3//TEAD1//KAT2B//CCNC//CMTR2//ZFC3H1//RTCA//PPP4R2//ESRP1//CPEB1//QKI//MTPN//ACBD3//LBR//MSMO1//IDH1//UGT1A4//UGT1A1//G3BP2//NF2//UGT1A8//OSBPL5//LDLR//PLEKHA1//EPHA2//PTHLH//PSAT1//EREG//UGT1A7//DDX6//ACOT7//PPCS//GLRX//INTS8//RBM47//MAP3K2//MAPRE3//BAMBI//RGMB//LRP6//MAP3K5//WNT7A//ACVR1//DKK3//NRG1//RASD1//TSG101//BTAF1//PPM1F//TOB1//SOSTDC1//TMEM100//RGMA//BMP3//TGFB2//SULF1//CTDSPL2//CALM2//ADRB1//EGLN1//CMKLR1//CCT6A//EPHA5//FBXW7//UXS1//DRD1//RNF180//TDRD5//FZD6//FASTK//AHNAK//YTHDF2//FXR1//NPLOC4//MAP2K4//S1PR1//DLL1//IL2//ITGA6//MET//PPP1R12A//NCK1//OSM//P2RY1//PPP3R1//DCAF6//CAPRIN2//FAM20C//MPP1//RAB23//ENPP4//SLC25A32//PTEN//EGLN3//PFKP//REXO1//ZC3H12C//NANOS1//RAP2C//TOB2//KLHL15//DUSP1//GJA1//NPNT// |
| GO:0048863 | stem cell differentiation | Biological process | 26 | 268 | 934 | 17653 | 1.83362363770015 | 0.00215294592523632 | 0.0257705721988907 | 2.66696687805686 | 0.0278372591006424 | SEMA4B//ACVR1//HAND2//TGFB2//RDH10//EDN1//FOXC1//NRG1//SOX11//KBTBD8//FRZB//LRP6//WNT7A//SOX17//GATA6//REST//MAPK1//MYB//YTHDF2//CBFB//STAT3//OSR1//ESR1//GPM6A//JARID2//HMGA2// |
| GO:0000302 | response to reactive oxygen species | Biological process | 23 | 227 | 934 | 17653 | 1.91502136611042 | 0.00217440600131313 | 0.0259814625209906 | 2.66265936196663 | 0.0246252676659529 | EDN1//JUN//PKD2//MAPK1//CRK//DUSP1//NR4A3//FOSL1//PPP1R15B//ADAM9//KLF2//ETS1//MAP3K5//MYB//PRKAA1//PLEKHA1//BNIP3//KLF4//EGLN1//SESN3//SESN1//MET//TNF// |
| GO:0043388 | positive regulation of DNA binding | Biological process | 9 | 55 | 934 | 17653 | 3.09279735254039 | 0.00221298360070571 | 0.0263957814666185 | 2.65502180439602 | 0.00963597430406852 | FOXC1//KLF4//IGF1//NEUROD1//POU4F1//HMGA2//HAND2//NME1//PPARG// |
| GO:0050773 | regulation of dendrite development | Biological process | 16 | 136 | 934 | 17653 | 2.22357979594407 | 0.00224117167892188 | 0.0266849367333603 | 2.64952487423127 | 0.0171306209850107 | FMR1//NSMF//HECW2//FXR1//EPHA4//RAB21//CAPRIN2//CPEB3//LPAR1//NRG1//IL2//C21ORF91//EFNA1//PTEN//LRP8//VLDLR// |
| GO:0042176 | regulation of protein catabolic process | Biological process | 32 | 355 | 934 | 17653 | 1.70370057604729 | 0.00235489929904689 | 0.0279897784876171 | 2.62802765957684 | 0.0342612419700214 | TMF1//BAG6//BAG5//RNF180//ANKIB1//LONRF3//LONRF1//SOCS5//MYLIP//NRG1//MDM4//WWP2//GJA1//NSF//HECW2//SOX17//TNF//SH3D19//ADAM9//FMR1//FBXW7//EFNA1//EPHA4//FBXL5//MSN//DERL2//EDEM1//ATXN3//RNF128//CTSA//LDLR//PTEN// |
| GO:0060548 | negative regulation of cell death | Biological process | 74 | 1005 | 934 | 17653 | 1.3916733250237 | 0.00237608834994416 | 0.0281920798432848 | 2.62413741504612 | 0.0792291220556745 | IL2//HIPK3//TOPORS//CDKN1A//ZNF268//OSR1//DUSP1//EPHA2//EPHA4//EPHA5//EPHA7//FHL2//ARL6IP1//DSTYK//GATA6//IGF1//KDR//MDM4//MET//ZFAND6//PRKAA1//SPHK2//PTEN//RARB//BCL11B//BNIP3//STAT3//ACTC1//TFAP2A//TMF1//WNT7A//BAG6//HMGA2//SQSTM1//CCND2//HAND2//DLL1//CCL2//EDN1//LRP6//POU4F1//WFS1//KLF4//FNIP1//CEBPB//EN1//JUN//NTF3//NR4A2//BTG2//NR4A3//CPEB4//EFNA1//BTBD10//BAG5//FOXC1//TSC1//MAP2K4//NEUROD1//ITGA5//NOG//ZMYND11//ITGA6//AR//ITGAV//ACVR1//TNF//URI1//ZFPM2//NCK1//NPAS2//DUS2//SOX4//SOX11// |
| GO:0007628 | adult walking behavior | Biological process | 6 | 27 | 934 | 17653 | 4.20009517011658 | 0.0024518019227378 | 0.0290346542048069 | 2.61051461871184 | 0.00642398286937901 | DRD1//EPHA4//GLRA1//GLRB//KCNJ10//SCN1A// |
| GO:0010720 | positive regulation of cell development | Biological process | 42 | 504 | 934 | 17653 | 1.57503568879372 | 0.00245614583071969 | 0.0290346542048069 | 2.60974585105015 | 0.0449678800856531 | STMN2//NPTN//NCK1//NME1//SERPINI1//TWF1//ZNF804A//DNMT3B//MMD//NEUROD1//NEUROG1//PTEN//RARB//SOX11//ZEB1//GOLGA4//NRG1//EFNA5//LPAR3//PPARG//RHEB//BMPR2//MAN2A1//SLIT2//EPHA4//RAB21//CAPRIN2//MYB//EDN1//LPAR1//CPEB3//FMR1//IL2//C21ORF91//LRP8//IGF1//VLDLR//CRK//CRKL//DOCK5//PLAA//NSMF// |
| GO:0003179 | heart valve morphogenesis | Biological process | 7 | 36 | 934 | 17653 | 3.67508327385201 | 0.00245998179200863 | 0.0290346542048069 | 2.60906810739233 | 0.00749464668094218 | EFNA1//TGFB2//TGFBR2//ACVR1//BMPR2//SOX4//ERG// |
| GO:0050769 | positive regulation of neurogenesis | Biological process | 37 | 430 | 934 | 17653 | 1.62631592052189 | 0.00253302095979003 | 0.0298446354548083 | 2.59636121657207 | 0.0396145610278373 | STMN2//NPTN//NCK1//NME1//SERPINI1//TWF1//ZNF804A//DNMT3B//MMD//NEUROD1//NEUROG1//PTEN//RARB//SOX11//ZEB1//GOLGA4//NRG1//EFNA5//LPAR3//PPARG//RHEB//BMPR2//SLIT2//EPHA4//RAB21//CAPRIN2//MYB//LPAR1//CPEB3//FMR1//IL2//C21ORF91//LRP8//VLDLR//PLAA//NSMF//MAN2A1// |
| GO:0010647 | positive regulation of cell communication | Biological process | 118 | 1737 | 934 | 17653 | 1.28396691728953 | 0.00254388384815604 | 0.0298589042832419 | 2.59450272213388 | 0.126338329764454 | GADD45A//MINK1//TNF//MAP3K2//CRK//CRKL//MAP3K5//TAOK3//LAMTOR3//ADAM9//DUSP5//LPAR1//LPAR3//NRG1//IGF1//NTF3//PIK3CB//PRKAA1//MAPK1//MAP2K4//EREG//FBXW7//COPS8//MDFIC//TOB1//EPS8//ARHGAP1//ARHGAP6//F3//GJA1//GDF11//INHBB//BMP3//BMPR2//TGFB2//ACVR1//KDR//EDN1//P2RY1//SOX11//SULF1//SOX4//NPNT//TGFB1I1//GATA6//ITGA5//PKD2//RRAGD//RHEB//SERP1//AR//LMO3//SYBU//IL2//OSM//STAT3//RHOC//NDFIP2//CC2D1A//MIER1//SLC20A1//UBE2I//EFNA1//EPHA2//EPHA4//EPHA5//EPHA7//ITGAV//MET//FZD10//SPRED1//PMAIP1//ADRB1//SOS1//DSTYK//NPTN//DLL1//KAT2B//WNT7A//RASGEF1A//SOS2//ESR1//GAB1//GRB2//DRD1//TSHZ3//SLC24A2//PTEN//SNAP25//CALM2//RAB3GAP1//JUN//DNAJC27//CCL2//CCL8//HAND2//ARRDC3//BCL2L11//MOAP1//BNIP3//BAMBI//LRP6//CAPRIN2//TBL1XR1//PAIP2//SQSTM1//FMR1//DYNLL2//E2F1//PPP3R1//YWHAG//CREBRF//PIAS4//SERINC3//NCK1//MYB//TMF1//ST18// |
| GO:0060419 | heart growth | Biological process | 13 | 101 | 934 | 17653 | 2.43272839061208 | 0.00254737474429375 | 0.0298589042832419 | 2.59390716127616 | 0.0139186295503212 | S1PR1//FOXC1//TGFB2//GJA1//JARID2//PTEN//TGFBR2//NOG//ZFPM2//GATA6//MAP2K4//EDN1//IGF1// |
| GO:0042552 | myelination | Biological process | 14 | 113 | 934 | 17653 | 2.34164597979951 | 0.00254747712131163 | 0.0298589042832419 | 2.59388970766804 | 0.0149892933618844 | KCNJ10//POU3F2//PPP3R1//RARB//TNFRSF21//PTEN//WASF3//NRG1//PIKFYVE//LPAR1//EGR2//TSC1//SBF2//QKI// |
| GO:0032147 | activation of protein kinase activity | Biological process | 30 | 328 | 934 | 17653 | 1.72869770721262 | 0.00255268400490781 | 0.0298681694207466 | 2.59300294289987 | 0.0321199143468951 | GADD45A//MINK1//TNF//MAP3K2//CRK//CRKL//MAP3K5//TAOK3//LAMTOR3//ADAM9//DUSP5//LPAR1//LPAR3//NRG1//IGF1//NTF3//PIK3CB//PRKAA1//MAPK1//MAP2K4//EFNA5//COPS8//MDFIC//ADCY3//PRKACB//PRKAR2B//PAK6//TGFB2//TGFBR2//SLK// |
| GO:0007617 | mating behavior | Biological process | 5 | 19 | 934 | 17653 | 4.97379691198016 | 0.00256076935906201 | 0.0299110244824462 | 2.59162953539231 | 0.00535331905781585 | THRA//PTEN//DMRTA1//DRD1//NHLH2// |
| GO:0001570 | vasculogenesis | Biological process | 11 | 78 | 934 | 17653 | 2.66544501180475 | 0.00258659518701861 | 0.0300987262815329 | 2.58727153487087 | 0.0117773019271949 | WNT7A//SPRED1//KDR//TMEM100//GJC1//FOXF1//ZFPM2//ITGAV//SOX17//TGFBR2//QKI// |
| GO:1905897 | regulation of response to endoplasmic reticulum stress | Biological process | 11 | 78 | 934 | 17653 | 2.66544501180475 | 0.00258659518701861 | 0.0300987262815329 | 2.58727153487087 | 0.0117773019271949 | WFS1//BCL2L11//SERINC3//NCK1//PMAIP1//DNAJB9//PPP1R15B//DERL2//EDEM1//ATXN3//BAG6// |
| GO:0035987 | endodermal cell differentiation | Biological process | 8 | 46 | 934 | 17653 | 3.28703100269993 | 0.00259019055091707 | 0.0300987262815329 | 2.58666828527484 | 0.00856531049250535 | GATA6//SOX17//HSBP1//ITGA5//ITGAV//LAMA3//MMP2//HMGA2// |
| GO:0042542 | response to hydrogen peroxide | Biological process | 16 | 138 | 934 | 17653 | 2.19135400179996 | 0.00260250659672107 | 0.0301382741671654 | 2.58460816098518 | 0.0171306209850107 | KLF2//ETS1//MAP3K5//MYB//PRKAA1//PLEKHA1//BNIP3//KLF4//MET//NR4A3//CRK//DUSP1//JUN//FOSL1//PPP1R15B//ADAM9// |
| GO:0046546 | development of primary male sexual characteristics | Biological process | 16 | 138 | 934 | 17653 | 2.19135400179996 | 0.00260250659672107 | 0.0301382741671654 | 2.58460816098518 | 0.0171306209850107 | BCL2L11//RNF38//ESR1//GATA6//TFAP2C//TGFB2//ARID5B//AR//PLEKHA1//TMF1//TCF21//TBC1D20//ARID4B//ARID4A//ZFPM2//LHFPL2// |
| GO:0051090 | regulation of DNA binding transcription factor activity | Biological process | 36 | 416 | 934 | 17653 | 1.63561398451655 | 0.00261524179111799 | 0.0301900250723878 | 2.58248815242045 | 0.0385438972162741 | PKD2//THRA//G3BP2//CMKLR1//PIAS4//KLF4//EPHA5//LRP8//RPS6KA4//RPS6KA5//WWP2//ESR1//RLIM//EGLN1//RNF2//WFS1//FZD6//BHLHE40//HAND1//HAND2//EDN1//LRP6//NEUROD1//NEUROG1//NHLH2//PPARG//PTEN//TNF//FOSL1//ARID5B//MTPN//AR//CLOCK//JUN//POU4F1//MAPK1// |
| GO:0007165 | signal transduction | Biological process | 369 | 6210 | 934 | 17653 | 1.12306892592248 | 0.00261590339973669 | 0.0301900250723878 | 2.58237829770859 | 0.395074946466809 | RANBP9//PPM1L//SPRED1//DUSP5//EREG//RASGEF1A//GRB2//NRG1//IL2//JAK1//MAP3K5//MET//MINK1//TAOK3//MAPK1//CCL2//SOS1//TNF//LAMTOR3//GADD45A//MAP3K2//CRK//CRKL//ADAM9//LPAR1//LPAR3//IGF1//NTF3//PIK3CB//PRKAA1//MAP2K4//DUSP10//DUSP1//DUSP2//PPP2CA//RGS3//JUN//PMAIP1//BCL2L11//MOAP1//CBFB//P2RY1//THRA//PRKACB//SKP1//RPS6KA5//BAG6//SOX17//S1PR1//GPR6//SPHK2//APCDD1//CTNND2//AMER2//CCNY//SOSTDC1//HBP1//DKK3//LRP6//PIAS4//STRN//TGFB1I1//WNT7A//TCF7L1//PPP3R1//FZD6//KDR//PTPRG//AKAP11//DGKH//DCX//PIKFYVE//DSTYK//LATS2//MKNK2//ARHGEF4//TREM1//PRKAR2B//ARHGEF28//STK33//MASTL//DGKE//SQSTM1//RPS6KA4//STK17B//PALM2//BMPR2//FBXW7//ERRFI1//TSG101//SOCS5//CDKN1A//E2F1//CNOT6L//CNOT7//MDM4//CNOT6//RBL2//SOX4//BTG2//WFS1//PPP1R15B//IRF9//EDN1//CD274//PDCD1LG2//EFNA5//NCK1//EPS8//EPS15//GAB1//CAMLG//COL1A2//TGFB2//TGFBR2//KLF10//ACVR1//ZFYVE9//PDE10A//ADCY3//RAPGEF4//FZD10//CHRM2//CHRM5//FGD4//CMKLR1//ADRB1//GPR180//DRD1//FRZB//GNAQ//RHOC//RASD1//PPARG//GPRC5C//PTGER4//PTHLH//CCL8//SOS2//CALM2//OR6A2//ESR1//GNB5//FMR1//GLRA1//GLRB//ADAM10//FOXC1//DLL1//EPN1//TMEM100//KAT2B//TMEM17//DZIP1//FOXF1//ITGA6//ITGA5//ITGAV//ITGB8//PLEK//OSR1//NKIRAS1//SNIP1//COPS8//MDFIC//PKD2//STAT3//STAT4//NEUROD1//RGL1//DOCK9//RAB38//RND3//ARHGAP1//RALGPS2//DOCK5//USP28//G3BP2//CDC42EP3//ARHGAP6//PAK6//CDS1//TRPC3//GIT2//APPL1//PIK3R3//FLRT1//ESRP1//OTX2//BNIP3//TOPORS//EPHA2//NR5A2//RARB//ADIPOR2//CRY2//YWHAG//TOB1//EPHA4//EPHA5//EPHA7//RGS17//GDF11//IFNA1//INHBB//OGN//OSM//BMP3//TTR//VEGFB//CXCL14//F3//PMEPA1//LDLRAD4//PLEKHA1//PTEN//KLF4//SOX11//ZEB1//MMP2//ASPN//LRP8//DENND1B//TNFRSF21//SLK//SULF1//SHISA2//RGMB//RGMA//NOG//NPNT//BAMBI//PRDM16//RASL11B//GATA6//CTDSPL2//FHL2//AR//RNF4//NR4A2//NR4A3//CREBRF//SERP1//DERL2//FNIP1//TSC1//RRAGD//RHEB//ARAP2//AGFG1//AGFG2//PSD//RAB12//RAB21//RAB30//RAB9B//DNAJC27//RAB23//RAB22A//RAB28//RAP2C//RAP1B//UBA5//ST18//ACSL1//IRF1//FGD5//DLC1//GRIA1//CPEB4//TEAD1//NF2//LMO3//SLIT2//GSTA2//MSN//DNAJB9//EDEM1//VLDLR//WASL//BRWD1//SESN3//NPLOC4//FAM20C//ZFAND6//GJA1//NDFIP2//CC2D1A//MIER1//SLC20A1//UBE2I//ZMYND11//PGRMC2//NR3C2//EFNA1//HIPK3//AIDA//PEX5L//TMEM64//NPTN//WWP2//YTHDF2//ARID5B//FAM13A//TAGAP//TRIP10//ARHGAP12//CD2AP//OTUD3//VANGL1//RAB3GAP1//SLC17A7//TCF21//CEBPB//HAND2//IQGAP2//FIBIN//SEMA4B//ARRDC3//POU4F1//ZMAT3//CAPRIN2//TBL1XR1//FASTK//SGPP1//ITM2B//PPM1F//LYPD6//DYNLL2//RBBP7//RBBP8//BAG5//SERINC3//SESN1//CLOCK//PHF14//URI1//CNIH1//CDK6//PTGES3//RASSF8//CLCN6//DPYSL2//ELK3//STX2//ERG//EVI2A//LRCH1//RASSF3//LRP12//HIVEP2//MPP1//PPP1R12A//KCNK10//PPP1R12C//SMOC1//SMOC2//BCL11B//SPOCK1//ZNF217//DEK//NSMAF//PPFIA1//RHPN2//ARRDC4//PLAA//FEZ2//CD69//ULK2// |
| GO:1901652 | response to peptide | Biological process | 41 | 491 | 934 | 17653 | 1.57824350078719 | 0.00264947733153755 | 0.0305254091877146 | 2.57683979188102 | 0.0438972162740899 | GAB1//APPL1//GRB2//SOS1//PIK3R3//SESN3//EGR2//IGF1//TSC1//CPEB2//INHBB//ERRFI1//PPARG//PTEN//CPEB1//SLC2A4//YWHAG//KAT2B//EREG//GJA1//LRP6//BTG1//BTG2//NCK1//STAT3//MAPK1//CRK//EDN1//NR4A2//NR4A3//ADCY3//GJB2//PRKACB//PRKAR2B//RAP1B//KLF2//KLF3//KLF10//KLF4//EPHA4//PRKAA1// |
| GO:0042594 | response to starvation | Biological process | 19 | 177 | 934 | 17653 | 2.0288595313275 | 0.00267175690739262 | 0.0307297482392794 | 2.57320305913837 | 0.0203426124197002 | DSC2//INHBB//RRAGD//BMPR2//KLF10//PLEKHF2//FNIP1//CDKN1A//SESN3//SESN1//MAP3K5//MAPK1//PMAIP1//PRKAA1//CPEB4//JUN//UGT1A1//PPARG//ULK2// |
| GO:0016050 | vesicle organization | Biological process | 35 | 402 | 934 | 17653 | 1.64555967485911 | 0.00269624362940713 | 0.0309587362744999 | 2.56924086802444 | 0.0374732334047109 | TBC1D20//AGFG1//TMF1//STX6//SYT6//STX2//SNAP25//WASL//RAB22A//AKTIP//FAM160A2//SQSTM1//TBC1D12//EVI5//TBC1D17//USP6NL//RAB38//KIF13A//VPS37A//CHMP3//TSG101//TMED9//CNIH1//SEC23A//GRIA1//NSF//SAR1B//PPP6C//MCFD2//MIA3//SLC17A7//EPS15//CD2AP//SNX5//SNX2// |
| GO:0003207 | cardiac chamber formation | Biological process | 4 | 12 | 934 | 17653 | 6.30014275517488 | 0.00273784235895342 | 0.031171764096973 | 2.56259156153717 | 0.00428265524625268 | SOX4//SOX11//HAND1//HAND2// |
| GO:0051938 | L-glutamate import | Biological process | 4 | 12 | 934 | 17653 | 6.30014275517488 | 0.00273784235895342 | 0.031171764096973 | 2.56259156153717 | 0.00428265524625268 | ARL6IP1//KCNJ10//PER2//SLC17A7// |
| GO:0072178 | nephric duct morphogenesis | Biological process | 4 | 12 | 934 | 17653 | 6.30014275517488 | 0.00273784235895342 | 0.031171764096973 | 2.56259156153717 | 0.00428265524625268 | LHX1//OSR1//EPHA4//EPHA7// |
| GO:1900102 | negative regulation of endoplasmic reticulum unfolded protein response | Biological process | 4 | 12 | 934 | 17653 | 6.30014275517488 | 0.00273784235895342 | 0.031171764096973 | 2.56259156153717 | 0.00428265524625268 | WFS1//DNAJB9//NCK1//PPP1R15B// |
| GO:1903729 | regulation of plasma membrane organization | Biological process | 4 | 12 | 934 | 17653 | 6.30014275517488 | 0.00273784235895342 | 0.031171764096973 | 2.56259156153717 | 0.00428265524625268 | TMEFF2//AR//TGFB2//WASL// |
| GO:0009267 | cellular response to starvation | Biological process | 16 | 139 | 934 | 17653 | 2.17558886509636 | 0.00280054738245921 | 0.0318321041135658 | 2.55275707510504 | 0.0171306209850107 | CDKN1A//SESN3//SESN1//MAP3K5//MAPK1//PMAIP1//PRKAA1//CPEB4//JUN//RRAGD//DSC2//INHBB//BMPR2//KLF10//PLEKHF2//FNIP1// |
| GO:0043161 | proteasome-mediated ubiquitin-dependent protein catabolic process | Biological process | 35 | 403 | 934 | 17653 | 1.64147640023167 | 0.00280752840788485 | 0.0318579104404786 | 2.55167584064969 | 0.0374732334047109 | UBXN4//DNAJB9//DERL2//JKAMP//NPLOC4//WFS1//BAG6//EDEM1//CDC23//FBXL5//FBXW7//SKP1//BAG5//RNF180//ANKIB1//LONRF3//LONRF1//SOCS5//KLHL15//TOPORS//WWP2//UBXN2B//RNF38//RNF145//ZNRF2//CD2AP//SPOPL//ATXN3//RLIM//UBE2W//HECW2//RNF4//TBL1XR1//PLAA//CLOCK// |
| GO:0070997 | neuron death | Biological process | 29 | 316 | 934 | 17653 | 1.73453297373485 | 0.00283977538426351 | 0.0321698507935915 | 2.54671600968279 | 0.0310492505353319 | EGLN3//NSMF//CEBPB//EN1//JUN//NTF3//NR4A2//POU4F1//CCL2//WFS1//BTG2//NR4A3//CPEB4//BCL2L11//EPHA7//MYB//MAP2K4//TFAP2A//TGFB2//TNFRSF21//BNIP3//EGLN1//TSC1//STAT3//BTBD10//MAP3K5//FBXW7//MEOX2//BAG5// |
| GO:0072395 | signal transduction involved in cell cycle checkpoint | Biological process | 11 | 79 | 934 | 17653 | 2.63170520152875 | 0.00286471322399232 | 0.0323439992218031 | 2.54291884715954 | 0.0117773019271949 | CDKN1A//GADD45A//E2F1//CNOT6L//CNOT7//MDM4//CNOT6//RBL2//SOX4//BTG2//SOX11// |
| GO:1901879 | regulation of protein depolymerization | Biological process | 11 | 79 | 934 | 17653 | 2.63170520152875 | 0.00286471322399232 | 0.0323439992218031 | 2.54291884715954 | 0.0117773019271949 | EPS8//TWF1//STMN2//MID1IP1//LIMA1//CFL2//PLEK//TMOD3//TMOD1//MTPN//ASPH// |
| GO:0034405 | response to fluid shear stress | Biological process | 7 | 37 | 934 | 17653 | 3.57575669888304 | 0.00289780298817528 | 0.0326087214792503 | 2.53793114407423 | 0.00749464668094218 | ETS1//ADAM9//KLF2//PKD2//KLF4//SOCS5//GJA1// |
| GO:1905898 | positive regulation of response to endoplasmic reticulum stress | Biological process | 7 | 37 | 934 | 17653 | 3.57575669888304 | 0.00289780298817528 | 0.0326087214792503 | 2.53793114407423 | 0.00749464668094218 | BCL2L11//SERINC3//NCK1//PMAIP1//EDEM1//ATXN3//BAG6// |
| GO:0001649 | osteoblast differentiation | Biological process | 21 | 205 | 934 | 17653 | 1.93614143207813 | 0.00290531933107457 | 0.0326389944120553 | 2.53680612623346 | 0.0224839400428266 | PTHLH//TMEM64//SMOC1//TOB1//CDK6//TNF//NOG//HAND2//CEBPB//NPNT//GJA1//IGF1//FAM20C//BMPR2//SOX11//ACVR1//EPHA2//FHL2//MEF2D//BMP3//CBFB// |
| GO:0055008 | cardiac muscle tissue morphogenesis | Biological process | 10 | 68 | 934 | 17653 | 2.77947474493009 | 0.00292074117316588 | 0.0327578317647112 | 2.53450692712308 | 0.0107066381156317 | ZFPM2//NRG1//TGFB2//POU4F1//NOG//S1PR1//FOXC1//HAND1//EGLN1//ACTC1// |
| GO:0051402 | neuron apoptotic process | Biological process | 22 | 219 | 934 | 17653 | 1.8986731590938 | 0.00298025993347162 | 0.0332888449276497 | 2.52574585580653 | 0.0235546038543897 | EGLN3//NSMF//CEBPB//EN1//JUN//NTF3//NR4A2//POU4F1//CCL2//WFS1//BTG2//NR4A3//CPEB4//BCL2L11//EPHA7//MYB//MAP2K4//TFAP2A//TGFB2//FBXW7//TNFRSF21//BNIP3// |
| GO:0021884 | forebrain neuron development | Biological process | 6 | 28 | 934 | 17653 | 4.05009177118385 | 0.00298285376699035 | 0.0332888449276497 | 2.52536803719974 | 0.00642398286937901 | ARX//SLIT2//DRD1//LHX6//GNAQ//LHX8// |
| GO:0090659 | walking behavior | Biological process | 6 | 28 | 934 | 17653 | 4.05009177118385 | 0.00298285376699035 | 0.0332888449276497 | 2.52536803719974 | 0.00642398286937901 | DRD1//EPHA4//GLRA1//GLRB//KCNJ10//SCN1A// |
| GO:0009791 | post-embryonic development | Biological process | 12 | 91 | 934 | 17653 | 2.49236416688237 | 0.00299592030162089 | 0.0333751886853843 | 2.52346974405223 | 0.012847965738758 | BCL11B//KLF4//BCL2L11//NKX2-3//MORC3//SERP1//GNAQ//LHX1//ACO1//NR4A2//PLEKHA1//ARID5B// |
| GO:0034641 | cellular nitrogen compound metabolic process | Biological process | 403 | 6851 | 934 | 17653 | 1.11178989797204 | 0.00300046055311454 | 0.0333751886853843 | 2.52281207850538 | 0.431477516059957 | IGF1//NFIA//NFIB//RBBP7//RBBP8//RRM2//E2F1//NPAT//TSHZ1//HMG20A//KLF2//ZBTB18//CEBPB//ZNF268//TCERG1//WWP2//KLF12//OSR1//CRY2//GLIS3//ARX//DNMT3B//DR1//EDN1//EFNA1//CC2D1B//EN1//ESR1//FHL2//CPEB3//FOXF1//FOXC1//MYT1L//CIC//ZFPM2//DNAJB5//GATA6//CNOT7//HSBP1//AR//JARID2//JUN//ZNF568//MAF//MDM4//MYB//NR4A2//RLIM//KLF3//ZBTB7A//PIAS4//POU4F1//PPARG//CC2D1A//TSHZ3//ZBTB4//RARB//REST//RNF2//PRDM16//SOX17//SOX11//STAT3//ZEB1//TCF21//TFAP2A//TFAP2C//TGIF1//KLF10//TNF//UBE2I//VLDLR//WFS1//ZNF217//LRP8//BTG2//BHLHE41//TBL1XR1//NR4A3//HMGA2//ARID5B//BRMS1L//BHLHE40//RUNX3//URI1//PER2//SQSTM1//NOG//KLF4//HAND1//FNIP1//PHF14//USP3//DCP2//PPP2CA//RPL15//CNOT6L//PAN3//CNOT6//RBFOX3//FMR1//MBNL1//RBM25//SRSF2//ELAVL2//NOVA1//COPS8//PTGES3//CCNE2//NABP1//GTF2E1//SECISBP2L//ITGB8//ST6GALNAC3//ST6GALNAC6//ST8SIA3//ST3GAL5//DUS2//IRF9//KLF6//CREBRF//EGR2//ELK3//ERG//FOXJ3//FOXF2//FLI1//GRHL1//HIVEP2//IRF1//LHX1//MEF2D//MEOX2//MYBL1//NEUROD1//NHLH2//OTX2//PAX9//PBX3//POU3F2//RAD21//ARID4A//SHOX//BCL11B//BMPR2//SOX4//BTF3//TEF//TGFB1I1//THRA//TMF1//DEK//FOSL1//KLF7//CBFB//FUBP3//HAND2//CLOCK//POLR3G//PKD2//UGP2//GFPT2//NAGK//SLC35D1//NUP35//NUP43//CHCHD10//COX8C//COX7A2L//GSTA2//IDH1//DPYSL2//TTR//HPRT1//NME1//CMPK1//ADCY3//IMPDH1//PDE10A//DCTPP1//REV3L//GMNC//ESCO2//GADD45A//EPC2//TAOK3//UBE2W//USP28//USP45//ATXN3//KDM2A//MTRR//HMGB3//BAHD1//H2AFZ//KMT2B//DPY30//ARID4B//TOPORS//KHDRBS3//ZMYND11//TADA1//ZNF597//CTNND2//ZNF782//NKX2-3//ZNF800//E2F5//ZNF367//EZH1//ZBTB9//MYCBP2//FLII//LHX6//HBP1//ATAD2//SERTAD3//MDFIC//MNX1//HOXA3//HOXD1//FOXN2//ZBTB41//ZBTB34//LHX8//NEUROG1//NFE2//NPAS2//IER5//BRWD1//BNC2//ZNF532//CDCA7L//PRKAA1//TRERF1//LMO3//MAPK1//SLC2A4RG//PHTF2//ZNF630//ZNF287//ZNF512B//ZBTB26//MIER1//RBL2//RNF4//ATXN1//DMRTA1//SMARCD2//STAT4//ZNF2//ZNF3//ZNF131//ZNF655//BRD3//TCF7L1//PCGF5//LCOR//WASL//ZBTB47//ST18//HIPK3//NR5A2//NRBF2//MLLT6//RNF141//PAK6//SNAPC1//BTG1//SNIP1//RPS6KA4//RPS6KA5//CDKN1A//CRK//EMX2//ETS1//BOLA3//NR3C2//PLEK//ZNFX1//NKX2-4//RFX7//BDP1//CDK8//E2F3//TEAD1//KAT2B//CCNC//CMTR2//ZFC3H1//RTCA//PPP4R2//ESRP1//CPEB1//QKI//CPEB2//CPEB4//MTPN//DDX6//MKNK2//ACO1//TSC1//KBTBD8//IMMP2L//NLN//THOP1//NSMAF//ORMDL3//B3GALT2//UGT1A4//UGT1A1//G3BP2//NF2//EPHA2//PTHLH//PSAT1//EREG//ACOT7//PPCS//GLRX//FAM129A//SERP1//PRR16//FXR1//INTS8//RBM47//MAP3K2//MAPRE3//BAMBI//RGMB//LRP6//MAP3K5//WNT7A//ACVR1//DKK3//NRG1//RASD1//TSG101//BTAF1//PPM1F//TOB1//NANOS1//CAPRIN2//SOSTDC1//TMEM100//RGMA//BMP3//TGFB2//SULF1//CTDSPL2//CALM2//ADRB1//EGLN1//CMKLR1//CCT6A//EPHA5//FBXW7//UXS1//NCK1//DRD1//SLC44A1//RNF180//TDRD5//FZD6//FASTK//AHNAK//YTHDF2//NPLOC4//MAP2K4//S1PR1//DLL1//IL2//ITGA6//MET//PPP1R12A//OSM//P2RY1//PPP3R1//DCAF6//PAIP2//FAM20C//MPP1//RAB23//ENPP4//SPHK2//SPTSSA//SLC25A32//PTEN//EGLN3//PFKP//NAPEPLD//REXO1//ZC3H12C//EPHA4//RAP2C//TOB2//PPP1R15B//CTSA//KLHL15//DUSP1//GJA1//NPNT//UNK// |
| GO:0034250 | positive regulation of cellular amide metabolic process | Biological process | 16 | 140 | 934 | 17653 | 2.16004894463139 | 0.00301090867790916 | 0.0334364127893262 | 2.52130241661663 | 0.0171306209850107 | FAM129A//CPEB3//FMR1//SERP1//PRR16//MAPK1//SOX4//FXR1//NCK1//TNF//PPARG//EFNA1//EPHA4//YTHDF2//ESR1//NSMAF// |
| GO:0071559 | response to transforming growth factor beta | Biological process | 23 | 233 | 934 | 17653 | 1.86570751118913 | 0.00303107126884162 | 0.0336051393297965 | 2.51840385205179 | 0.0246252676659529 | COL1A2//JUN//TGFB2//TGFBR2//KLF10//ADAM9//ACVR1//ZFYVE9//DKK3//SOX11//ZEB1//NPNT//TGFB1I1//BAMBI//ASPN//PMEPA1//PRDM16//RASL11B//LDLRAD4//CRK//EDN1//WNT7A//RUNX3// |
| GO:0051101 | regulation of DNA binding | Biological process | 14 | 116 | 934 | 17653 | 2.28108616997711 | 0.00325064128341847 | 0.0358724483425411 | 2.48803095336682 | 0.0149892933618844 | FOXC1//IGF1//NME1//PPARG//E2F1//JUN//HMGA2//HAND2//HAND1//KLF4//SOX11//PER2//NEUROD1//POU4F1// |
| GO:2000134 | negative regulation of G1/S transition of mitotic cell cycle | Biological process | 14 | 116 | 934 | 17653 | 2.28108616997711 | 0.00325064128341847 | 0.0358724483425411 | 2.48803095336682 | 0.0149892933618844 | CDKN1A//GADD45A//E2F1//CNOT6L//CNOT7//MDM4//CNOT6//RBL2//SOX4//BTG2//PKD2//PTEN//ZNF655//KLF4// |
| GO:0003215 | cardiac right ventricle morphogenesis | Biological process | 5 | 20 | 934 | 17653 | 4.72510706638116 | 0.0032674002926224 | 0.0358724483425411 | 2.48579765639458 | 0.00535331905781585 | HAND1//HAND2//ZFPM2//SOX4//TGFB2// |
| GO:0006491 | N-glycan processing | Biological process | 5 | 20 | 934 | 17653 | 4.72510706638116 | 0.0032674002926224 | 0.0358724483425411 | 2.48579765639458 | 0.00535331905781585 | GNPTG//MAN1A1//MAN2A1//ST8SIA3//EDEM1// |
| GO:0033962 | cytoplasmic mRNA processing body assembly | Biological process | 5 | 20 | 934 | 17653 | 4.72510706638116 | 0.0032674002926224 | 0.0358724483425411 | 2.48579765639458 | 0.00535331905781585 | CNOT6L//PAN3//CNOT6//DDX6//CNOT7// |
| GO:0060044 | negative regulation of cardiac muscle cell proliferation | Biological process | 5 | 20 | 934 | 17653 | 4.72510706638116 | 0.0032674002926224 | 0.0358724483425411 | 2.48579765639458 | 0.00535331905781585 | GJA1//JARID2//PTEN//TGFBR2//NOG// |
| GO:0043523 | regulation of neuron apoptotic process | Biological process | 20 | 194 | 934 | 17653 | 1.94849775933244 | 0.0033557852218071 | 0.0367831044652859 | 2.47420584282812 | 0.0214132762312634 | CEBPB//EN1//JUN//NTF3//NR4A2//POU4F1//CCL2//WFS1//BTG2//NR4A3//CPEB4//BCL2L11//EPHA7//MYB//MAP2K4//TFAP2A//TGFB2//FBXW7//EGLN3//NSMF// |
| GO:0050873 | brown fat cell differentiation | Biological process | 7 | 38 | 934 | 17653 | 3.48165783838612 | 0.00339286525540399 | 0.0371293652464356 | 2.46943338762765 | 0.00749464668094218 | DUSP10//PRDM16//CEBPB//ADRB1//ITGA6//SLC2A4//BNIP3// |
| GO:0043525 | positive regulation of neuron apoptotic process | Biological process | 8 | 48 | 934 | 17653 | 3.15007137758744 | 0.00341300340793994 | 0.0372894055701096 | 2.46686327807097 | 0.00856531049250535 | FBXW7//BCL2L11//EPHA7//JUN//MYB//MAP2K4//TFAP2A//TGFB2// |
| GO:0043583 | ear development | Biological process | 21 | 208 | 934 | 17653 | 1.90821631526931 | 0.00345154429468806 | 0.0376496678467344 | 2.46198654881047 | 0.0224839400428266 | LRIG1//NEUROG1//SOBP//TFAP2A//FZD6//MAPK1//TSHZ1//OSR1//EDN1//NOG//DLL1//ZEB1//NR4A3//GJB2//MAF//NEUROD1//TGFB2//CXCL14//FRZB//BCL2L11//RDH10// |
| GO:0048167 | regulation of synaptic plasticity | Biological process | 18 | 168 | 934 | 17653 | 2.02504588559192 | 0.00348075776358447 | 0.0379071896217742 | 2.45832619952501 | 0.019271948608137 | SNAP25//EGR2//NSMF//KCNJ10//NPTN//RAB3GAP1//DRD1//SLC24A2//MAPK1//PTEN//TSHZ3//GRIA1//PAIP2//EPHA4//SQSTM1//FMR1//CPEB3//YWHAG// |
| GO:0050673 | epithelial cell proliferation | Biological process | 35 | 409 | 934 | 17653 | 1.61739606184196 | 0.00356149385576564 | 0.0386705982850777 | 2.44836780055692 | 0.0374732334047109 | PIK3CB//BMPR2//SULF1//GJA1//TNF//F3//JUN//KDR//VEGFB//VASH2//BCL11B//CEBPB//EPHA2//MAPK1//EREG//BTBD10//OSR1//IGF1//NME1//NR4A3//NOG//CDK6//DUSP10//AR//PTEN//TGFB2//RUNX3//WNT7A//ESR1//ERRFI1//PPARG//STAT3//FBXW7//NFIB//SOX11// |
| GO:0044265 | cellular macromolecule catabolic process | Biological process | 79 | 1103 | 934 | 17653 | 1.3537024777665 | 0.00356229228620485 | 0.0386705982850777 | 2.44827044953361 | 0.0845824411134904 | DCP2//PPP2CA//RPL15//ZFAND4//CNOT6L//PAN3//CNOT7//CNOT6//CTSA//TOPORS//USP24//KLHL3//MYLIP//USP27X//ATXN3//RLIM//NPLOC4//USP28//USP46//UBE2I//BAG6//RNF128//KLHL15//USP45//CDC23//SQSTM1//USP3//UBE2W//TMF1//PMAIP1//UBXN4//DNAJB9//DERL2//JKAMP//WFS1//EDEM1//TGFB1I1//FBXL5//FBXW7//SKP1//BAG5//RNF180//ANKIB1//LONRF3//LONRF1//SOCS5//VLDLR//BNIP3//DNAJB12//WWP2//UBXN2B//RNF38//RNF145//ZNRF2//CD2AP//SPOPL//HECW2//RNF4//TBL1XR1//PLAA//CLOCK//VPS37A//TSG101//FMR1//YTHDF2//FXR1//DDX6//RAB12//E2F1//TOB1//CPEB3//BTG2//QKI//NANOS1//EFNA1//EPHA4//MSN//LDLR//PTEN// |
| GO:0001822 | kidney development | Biological process | 25 | 264 | 934 | 17653 | 1.78981328272013 | 0.00356840417289583 | 0.0386748676623309 | 2.44752596143791 | 0.0267665952890792 | GDF11//RDH10//OSR1//FOXC1//NPNT//LHX1//RARB//TCF21//SLIT2//PKD2//SULF1//NOG//SOX4//DLL1//KLHL3//EPHA4//EPHA7//BCL2L11//SOX11//TFAP2A//TGFB2//TSC1//WFS1//BAG6//ARID5B// |
| GO:0070306 | lens fiber cell differentiation | Biological process | 6 | 29 | 934 | 17653 | 3.91043343424647 | 0.00359531194526376 | 0.0389041514973101 | 2.44426342232441 | 0.00642398286937901 | TMOD1//WNT7A//TBC1D20//EPHA2//MAF//NF2// |
| GO:0014032 | neural crest cell development | Biological process | 10 | 70 | 934 | 17653 | 2.70006118078923 | 0.00362450097086646 | 0.0391573483481947 | 2.4407517795807 | 0.0107066381156317 | SEMA4B//ACVR1//HAND2//MAPK1//RDH10//EDN1//FOXC1//NRG1//SOX11//KBTBD8// |
| GO:0007179 | transforming growth factor beta receptor signaling pathway | Biological process | 19 | 182 | 934 | 17653 | 1.97312163211521 | 0.00364686211907118 | 0.0392628698678519 | 2.43808065622516 | 0.0203426124197002 | DKK3//SOX11//ZEB1//NPNT//TGFB1I1//BAMBI//ASPN//PMEPA1//PRDM16//RASL11B//TGFBR2//LDLRAD4//COL1A2//JUN//TGFB2//KLF10//ADAM9//ACVR1//ZFYVE9// |
| GO:0009612 | response to mechanical stimulus | Biological process | 21 | 209 | 934 | 17653 | 1.89908609366515 | 0.00365168492486749 | 0.0392628698678519 | 2.43750670137172 | 0.0224839400428266 | MAP3K2//MTPN//GADD45A//GJA1//IRF1//PTGER4//MAP2K4//BNIP3//NEUROG1//EDN1//JUN//SCN1A//DENND5B//PKD2//CLCN6//ETS1//P2RY1//PPARG//TGFBR2//BTG2//FOSL1// |
| GO:0048705 | skeletal system morphogenesis | Biological process | 21 | 209 | 934 | 17653 | 1.89908609366515 | 0.00365168492486749 | 0.0392628698678519 | 2.43750670137172 | 0.0224839400428266 | THRA//WNT7A//FOXC1//MEF2D//RARB//TGFBR2//OSR1//TFAP2A//RDH10//LHX1//HOXA3//DSCAML1//SOX11//ZEB1//NOG//MMP2//BMPR2//NEUROG1//RAB23//PLEKHA1//ARID5B// |
| GO:0042711 | maternal behavior | Biological process | 4 | 13 | 934 | 17653 | 5.81551638939219 | 0.00379041672403289 | 0.0403694304009991 | 2.42131304042799 | 0.00428265524625268 | CREBRF//DRD1//GNAQ//PTEN// |
| GO:0048934 | peripheral nervous system neuron differentiation | Biological process | 4 | 13 | 934 | 17653 | 5.81551638939219 | 0.00379041672403289 | 0.0403694304009991 | 2.42131304042799 | 0.00428265524625268 | POU4F1//RUNX3//HAND2//NEFH// |
| GO:0048935 | peripheral nervous system neuron development | Biological process | 4 | 13 | 934 | 17653 | 5.81551638939219 | 0.00379041672403289 | 0.0403694304009991 | 2.42131304042799 | 0.00428265524625268 | NEFH//POU4F1//RUNX3//HAND2// |
| GO:0050872 | white fat cell differentiation | Biological process | 4 | 13 | 934 | 17653 | 5.81551638939219 | 0.00379041672403289 | 0.0403694304009991 | 2.42131304042799 | 0.00428265524625268 | PPARG//PRDM16//TBL1XR1//PER2// |
| GO:0061029 | eyelid development in camera-type eye | Biological process | 4 | 13 | 934 | 17653 | 5.81551638939219 | 0.00379041672403289 | 0.0403694304009991 | 2.42131304042799 | 0.00428265524625268 | JUN//SOS1//SOX11//TFAP2A// |
| GO:0070307 | lens fiber cell development | Biological process | 4 | 13 | 934 | 17653 | 5.81551638939219 | 0.00379041672403289 | 0.0403694304009991 | 2.42131304042799 | 0.00428265524625268 | TBC1D20//EPHA2//TMOD1//WNT7A// |
| GO:0080135 | regulation of cellular response to stress | Biological process | 52 | 672 | 934 | 17653 | 1.46253313959417 | 0.00386708648715292 | 0.0411212357116591 | 2.41261611484878 | 0.0556745182012848 | MAP3K2//MDFIC//MAP3K5//MAP2K4//PPP4R2//MAPK1//TAOK3//TGFB2//FZD10//EDN1//EPHA4//TNF//HIPK3//DUSP10//AIDA//SPRED1//PMAIP1//NPAS2//ZMYND11//GADD45A//MINK1//WNT7A//KLF4//RGMA//PTEN//PTGES3//NUP35//HSBP1//NUP43//BAG5//IER5//HAND2//MET//PIAS4//WFS1//BCL2L11//SERINC3//NCK1//TSC1//NR4A3//FBXW7//DNAJB9//PPP1R15B//DERL2//EDEM1//ATXN3//BAG6//KLHL15//CDK6//HMGA2//DEK//FMR1// |
| GO:0010171 | body morphogenesis | Biological process | 8 | 49 | 934 | 17653 | 3.08578420661627 | 0.00389375976229327 | 0.0411460894881084 | 2.40963084714801 | 0.00856531049250535 | RAB3GAP1//LRP6//MMP2//ASPH//PAX9//PLEKHA1//ARID5B//NOG// |
| GO:0030834 | regulation of actin filament depolymerization | Biological process | 8 | 49 | 934 | 17653 | 3.08578420661627 | 0.00389375976229327 | 0.0411460894881084 | 2.40963084714801 | 0.00856531049250535 | EPS8//TWF1//LIMA1//CFL2//PLEK//TMOD3//TMOD1//MTPN// |
| GO:0051205 | protein insertion into membrane | Biological process | 8 | 49 | 934 | 17653 | 3.08578420661627 | 0.00389375976229327 | 0.0411460894881084 | 2.40963084714801 | 0.00856531049250535 | BCL2L11//PMAIP1//MOAP1//BAG6//DYNLL2//E2F1//PPP3R1//YWHAG// |
| GO:0061383 | trabecula morphogenesis | Biological process | 8 | 49 | 934 | 17653 | 3.08578420661627 | 0.00389375976229327 | 0.0411460894881084 | 2.40963084714801 | 0.00856531049250535 | NRG1//TGFB2//MMP2//FHL2//EGLN1//S1PR1//SOS1//NOG// |
| GO:0021782 | glial cell development | Biological process | 12 | 94 | 934 | 17653 | 2.41282062964144 | 0.00392587404140972 | 0.0414207272106926 | 2.4060636385104 | 0.012847965738758 | CDK6//DRD1//DLL1//POU3F2//WASF3//LPAR1//SOX11//KCNJ10//PPP3R1//PTEN//LDLR//SOX4// |
| GO:0030308 | negative regulation of cell growth | Biological process | 18 | 170 | 934 | 17653 | 2.00122181634967 | 0.00394891961246675 | 0.0415989771637268 | 2.40352170703769 | 0.019271948608137 | EPHA4//EPHA7//ULK2//SEMA4B//CDKN1A//FRZB//GJA1//SERTAD3//PPARG//PPP2CA//RBBP7//SOX17//BMPR2//CAPRIN2//BTG1//TGFB2//ADIPOR2//SLIT2// |
| GO:0007010 | cytoskeleton organization | Biological process | 91 | 1306 | 934 | 17653 | 1.3169517397877 | 0.0039612833073387 | 0.0416643219401736 | 2.40216409614305 | 0.0974304068522484 | KIF23//WASL//TACC2//DYNC1LI2//ATXN3//NEFH//MAP7D1//CLIP1//EPS8//RHOC//RND3//MARCKS//MYO1B//LIMA1//TWF1//CFL2//CORO2B//DLC1//FGD4//FGD5//PALLD//FLII//NF2//PFN2//PAK6//TRIP10//PALM2//CD2AP//TMOD3//NCK1//TMOD1//ARHGAP12//STMN2//RANBP9//SLAIN2//MID1IP1//GADD45A//PPP1R12A//HAUS8//WASF3//ARHGAP6//CHMP3//ACTC1//PLEK//SLIT2//CDC42EP3//GRB2//TNF//FRMD6//CNN3//MYLIP//MET//SLK//S1PR1//GAB1//MINK1//FZD10//CRK//EFNA5//EPHA5//TSC1//NEFM//IQGAP2//ARFIP1//AGFG1//MDM1//KAT2B//GOLGA8A//CDC14A//PKD2//MZT1//PTGER4//CLIC4//MAPK1//LPAR1//PPM1E//PPM1F//TMEFF2//PPFIA1//EDN1//RNF4//FRY//CEP97//NTF3//MTPN//SH3D19//DPYSL2//MSN//BRWD1//CCL2//CCDC6// |
| GO:0043434 | response to peptide hormone | Biological process | 36 | 427 | 934 | 17653 | 1.5934787296461 | 0.00400528604446472 | 0.0420617228551474 | 2.39736646257686 | 0.0385438972162741 | GAB1//APPL1//GRB2//SOS1//PIK3R3//SESN3//EGR2//IGF1//TSC1//CPEB2//INHBB//ERRFI1//PPARG//PTEN//CPEB1//SLC2A4//YWHAG//KAT2B//NCK1//STAT3//MAPK1//CRK//EDN1//NR4A2//NR4A3//ADCY3//GJB2//PRKACB//PRKAR2B//RAP1B//PRKAA1//EREG//GJA1//LRP6//BTG1//BTG2// |
| GO:0031571 | mitotic G1 DNA damage checkpoint | Biological process | 10 | 71 | 934 | 17653 | 2.66203215007389 | 0.00402347702960135 | 0.0421219429585045 | 2.3953984737569 | 0.0107066381156317 | CDKN1A//GADD45A//E2F1//CNOT6L//CNOT7//MDM4//CNOT6//RBL2//SOX4//BTG2// |
| GO:0044819 | mitotic G1/S transition checkpoint | Biological process | 10 | 71 | 934 | 17653 | 2.66203215007389 | 0.00402347702960135 | 0.0421219429585045 | 2.3953984737569 | 0.0107066381156317 | CDKN1A//GADD45A//E2F1//CNOT6L//CNOT7//MDM4//CNOT6//RBL2//SOX4//BTG2// |
| GO:0015800 | acidic amino acid transport | Biological process | 9 | 60 | 934 | 17653 | 2.83506423982869 | 0.00406710209201755 | 0.0425128461334076 | 2.39071492583899 | 0.00963597430406852 | ARL6IP1//SLC17A7//SLC17A6//SNAP25//PPFIA1//KCNJ10//SLC6A1//PER2//RAB3GAP1// |
| GO:0003272 | endocardial cushion formation | Biological process | 5 | 21 | 934 | 17653 | 4.50010196798205 | 0.00410415262590323 | 0.0427679263620702 | 2.38677649698127 | 0.00535331905781585 | TMEM100//TGFB2//NOG//TGFBR2//ACVR1// |
| GO:0044321 | response to leptin | Biological process | 5 | 21 | 934 | 17653 | 4.50010196798205 | 0.00410415262590323 | 0.0427679263620702 | 2.38677649698127 | 0.00535331905781585 | STAT3//INHBB//PTEN//NR4A3//EDN1// |
| GO:0048534 | hematopoietic or lymphoid organ development | Biological process | 65 | 882 | 934 | 17653 | 1.3928887043754 | 0.00419326564471788 | 0.0436293162388108 | 2.37744762403079 | 0.069593147751606 | ESCO2//PLEK//RBM47//REST//DLL1//NKX2-3//FNIP1//SOX4//NME1//MKNK2//TGFB2//RUNX3//CBFB//KLF6//IFNA1//DNAJB9//IL2//THRA//JUN//PPARG//EPHA2//OSTM1//TNF//BCL11B//SOS1//ZEB1//KDR//TGFBR2//KLF4//FLI1//FAM20C//PTGER4//KLF2//IRF1//TSC1//HMGB3//DUSP10//MYB//SOCS5//CDK6//ETS1//STAT3//NFE2//NR4A3//DPY30//KAT2B//KMT2B//CEBPB//TOB2//TMEM64//POU4F1//KLF10//BCL2L11//JARID2//TCF21//CRKL//HOXA3//MAPK1//HAND2//TMOD3//ARID4A//MFAP5//FOXC1//YTHDF2//FBXW7// |
| GO:0010921 | regulation of phosphatase activity | Biological process | 18 | 171 | 934 | 17653 | 1.98951876479207 | 0.00420181429413872 | 0.043651106100246 | 2.37656314584083 | 0.019271948608137 | TGFB2//TNF//NPNT//MTMR9//SPRED1//PPP1R37//PPP1R12A//PPP1R12C//PLEKHF2//URI1//PLEK//CRY2//MASTL//CALM2//PPP1R15B//UBXN2B//PPP4R2//TSC1// |
| GO:1990823 | response to leukemia inhibitory factor | Biological process | 12 | 95 | 934 | 17653 | 2.38742251775048 | 0.00428286592616082 | 0.0440918629489756 | 2.36826552084973 | 0.012847965738758 | EPS8//NR5A2//PCOLCE2//JARID2//NEFH//PFKP//PIGA//BCAT2//SOX17//ARID5B//KLF4//GFPT2// |
| GO:1990830 | cellular response to leukemia inhibitory factor | Biological process | 12 | 95 | 934 | 17653 | 2.38742251775048 | 0.00428286592616082 | 0.0440918629489756 | 2.36826552084973 | 0.012847965738758 | EPS8//NR5A2//PCOLCE2//JARID2//NEFH//PFKP//PIGA//BCAT2//SOX17//ARID5B//KLF4//GFPT2// |
| GO:0003382 | epithelial cell morphogenesis | Biological process | 6 | 30 | 934 | 17653 | 3.78008565310492 | 0.00429639770565946 | 0.0440918629489756 | 2.36689552400801 | 0.00642398286937901 | CLIC4//MET//FRMD6//PALLD//AR//BCL11B// |
| GO:0010719 | negative regulation of epithelial to mesenchymal transition | Biological process | 6 | 30 | 934 | 17653 | 3.78008565310492 | 0.00429639770565946 | 0.0440918629489756 | 2.36689552400801 | 0.00642398286937901 | TGFB2//NOG//EFNA1//PPP2CA//PTEN//LDLRAD4// |
| GO:0034390 | smooth muscle cell apoptotic process | Biological process | 6 | 30 | 934 | 17653 | 3.78008565310492 | 0.00429639770565946 | 0.0440918629489756 | 2.36689552400801 | 0.00642398286937901 | EDN1//IGF1//LRP6//MAP2K4//E2F3//PPARG// |
| GO:0034391 | regulation of smooth muscle cell apoptotic process | Biological process | 6 | 30 | 934 | 17653 | 3.78008565310492 | 0.00429639770565946 | 0.0440918629489756 | 2.36689552400801 | 0.00642398286937901 | EDN1//IGF1//LRP6//MAP2K4//E2F3//PPARG// |
| GO:1900739 | regulation of protein insertion into mitochondrial membrane involved in apoptotic signaling pathway | Biological process | 6 | 30 | 934 | 17653 | 3.78008565310492 | 0.00429639770565946 | 0.0440918629489756 | 2.36689552400801 | 0.00642398286937901 | BCL2L11//DYNLL2//E2F1//PMAIP1//PPP3R1//YWHAG// |
| GO:1900740 | positive regulation of protein insertion into mitochondrial membrane involved in apoptotic signaling pathway | Biological process | 6 | 30 | 934 | 17653 | 3.78008565310492 | 0.00429639770565946 | 0.0440918629489756 | 2.36689552400801 | 0.00642398286937901 | BCL2L11//DYNLL2//E2F1//PMAIP1//PPP3R1//YWHAG// |
| GO:0044783 | G1 DNA damage checkpoint | Biological process | 10 | 72 | 934 | 17653 | 2.62505948132286 | 0.00445635758397586 | 0.0456641611218617 | 2.35101996807742 | 0.0107066381156317 | CDKN1A//GADD45A//E2F1//CNOT6L//CNOT7//MDM4//CNOT6//RBL2//SOX4//BTG2// |
| GO:0050896 | response to stimulus | Biological process | 522 | 9123 | 934 | 17653 | 1.08144508983929 | 0.00446636139801932 | 0.0456974313688421 | 2.35004613867268 | 0.558886509635974 | E2F1//USP28//CLOCK//RANBP9//PPM1L//SPRED1//DUSP5//EREG//RASGEF1A//GRB2//NRG1//IL2//JAK1//MAP3K5//MET//MINK1//TAOK3//MAPK1//CCL2//SOS1//TNF//LAMTOR3//GADD45A//MAP3K2//CRK//CRKL//ADAM9//LPAR1//LPAR3//IGF1//NTF3//PIK3CB//PRKAA1//MAP2K4//DUSP10//DUSP1//DUSP2//PPP2CA//RGS3//COPS8//RBBP8//NABP1//DRD1//USP46//EGLN3//DNMT3B//ETS1//MMP2//MYB//NR4A2//EGLN1//BNIP3//TGFB2//TGFBR2//VEGFB//JUN//PMAIP1//BCL2L11//MOAP1//RAET1E//CBFB//GLRA1//GLRB//P2RY1//HPRT1//TBL1XR1//ADRB1//ARL6IP1//THRA//PRKACB//SKP1//RPS6KA5//MCOLN2//TNFRSF21//IFNA1//TSC1//DLL1//NKX2-3//BAG6//ACVR1//F3//GJA1//CCL8//PPARG//OSM//SLIT2//IRF1//GJB2//CPEB4//PER2//SQSTM1//PKD2//SOX17//S1PR1//GPR6//SPHK2//DNAJB4//UBXN4//DNAJB5//JKAMP//HERPUD2//HSPA13//INHBB//CAMLG//CEBPB//UGT1A1//STAT3//TMF1//CDK6//CHRM2//FOSL1//HMGA2//URI1//COL1A2//ENPP4//DOCK9//ZFPM2//GATA6//GNAQ//NFE2//PRKAR2B//APCDD1//CTNND2//AMER2//CCNY//SOSTDC1//HBP1//DKK3//LRP6//PIAS4//STRN//TGFB1I1//WNT7A//TCF7L1//PPP3R1//FZD6//FAM13A//CNIH1//DLC1//CDS1//CDC42EP3//GNB5//PTGES3//IQGAP2//ADCY3//RASSF8//ARAP2//TAGAP//CLCN6//DPYSL2//ELK3//STX2//EPS8//ERG//ESR1//EVI2A//LRCH1//CD2AP//APPL1//RASSF3//RGMB//GRIA1//CD274//CNOT7//LRP12//HIVEP2//AR//NR3C2//MPP1//PPP1R12A//RASD1//KCNK10//NDFIP2//PPP1R12C//CC2D1A//PSD//ARRDC3//MIER1//RARB//RHEB//SMOC1//SMOC2//BCL11B//SLC20A1//RASL11B//SPOCK1//VLDLR//ZNF217//LRP8//DEK//PDCD1LG2//NSMAF//PPFIA1//RHPN2//OR6A2//ARRDC4//TRIP10//PLAA//ARHGAP12//CXCL14//FEZ2//CD69//ULK2//KDR//PTPRG//AKAP11//DGKH//DCX//PIKFYVE//DSTYK//LATS2//MKNK2//ARHGEF4//TREM1//ARHGEF28//STK33//MASTL//DGKE//RPS6KA4//STK17B//PALM2//BMPR2//FBXW7//ERRFI1//TSG101//SOCS5//CALM2//EPC2//UBE2W//BTG2//USP45//USP3//ATXN3//ESCO2//RAD21//KDM2A//UGT1A6//UGT1A9//SLC17A7//SLC17A6//CMKLR1//PTGER4//PXK//CHST1//ZEB1//MNX1//YTHDF2//RAB23//SLC12A5//TOPORS//CDKN1A//FMR1//NPAS2//ZBTB4//ZMAT3//IMMP2L//CNOT6L//MDM4//CNOT6//RBL2//SOX4//IDH1//PON2//BTG1//WFS1//PPP1R15B//IRF9//EDN1//EFNA5//NCK1//EPS15//GAB1//KLF10//ZFYVE9//PDE10A//RAPGEF4//FZD10//CHRM5//FGD4//GPR180//FRZB//RHOC//GPRC5C//PTHLH//SOS2//ADAM10//FOXC1//EPN1//TMEM100//KAT2B//TMEM17//DZIP1//FOXF1//ITGA6//ITGA5//ITGAV//ITGB8//PLEK//OSR1//NKIRAS1//SNIP1//MDFIC//STAT4//NEUROD1//RGL1//RAB38//RND3//ARHGAP1//RALGPS2//DOCK5//G3BP2//ARHGAP6//PAK6//ARX//EFNA1//EPHA5//KIF5A//OTX2//ENAH//NTN4//NR4A3//KLF7//ACSL1//ADIPOR2//TRPC3//EGR2//EPHA4//LHX1//NOG//GIT2//PIK3R3//NPTN//FLRT1//ESRP1//EPHA2//DSC2//RRAGD//PLEKHF2//FNIP1//EMX2//TGIF1//SLC6A14//SLC6A1//SNN//TMEM135//CRY2//ELOVL4//SERINC3//WASL//FHL2//STEAP2//RAP1B//PTEN//NR5A2//YWHAG//TOB1//EPHA7//RGS17//CYBRD1//ACO1//FIBIN//GDF11//OGN//BMP3//TTR//PPP4R2//MTPN//TWF1//HAND2//JARID2//PMEPA1//LDLRAD4//PLEKHA1//KLF4//NME1//SLC32A1//SLC25A32//SOX11//RNF4//ASPN//SESN3//CPEB3//NSMF//SESN1//IPO5//CPEB1//DENND1B//SLK//SULF1//SHISA2//DNAJB9//DERL2//NPLOC4//EDEM1//RGMA//NPNT//BAMBI//PRDM16//CTDSPL2//CREBRF//SERP1//NEUROG1//AGFG1//AGFG2//RAB12//RAB21//RAB30//RAB9B//DNAJC27//RAB22A//RAB28//RAP2C//CPEB2//SLC2A4//UBA5//SRSF2//ST18//CALCOCO2//VKORC1L1//CETN1//IER5//FAM129A//NRBF2//TMX3//FGD5//TEAD1//NF2//LMO3//PPM1E//REST//PPM1F//GSTA2//MSN//SYBU//NAPEPLD//KLF2//DNAJB12//BRWD1//FAM20C//MIA3//DCTPP1//REV3L//ZFAND6//UBE2I//ZMYND11//BHLHE40//CKAP4//GNS//IMPDH1//C6ORF120//CTSA//SNAP25//ORMDL3//PGRMC2//HIPK3//AIDA//PEX5L//TMEFF2//TMEM64//POLR3G//HMGB3//PPP6C//RPL15//ACTC1//WWP2//ARID5B//RBBP7//SEMA4B//CDK19//LDLR//SCN1A//DENND5B//TRPV6//KCNJ3//OTUD3//KCNJ10//UGT1A10//UGT1A8//UGT1A7//UGT1A5//UGT1A4//UGT1A3//VANGL1//RAB3GAP1//TCF21//HSBP1//PLSCR4//CLIC4//TFAP2A//SLC41A1//POU3F2//HCN2//POU4F1//PTPN4//PAX9//H2AFZ//CCND2//RUNX3//KLHL15//NFIB//CAPRIN2//FASTK//SGPP1//ITM2B//ATG16L1//LYPD6//C5ORF30//NUP35//NUP43//BAG5//DYNLL2//KLF3//STMN2//DYNC1LI2//PCOLCE2//NEFH//PFKP//PIGA//BCAT2//GFPT2//PHF14//SLC24A2// |
| GO:0060393 | regulation of pathway-restricted SMAD protein phosphorylation | Biological process | 9 | 61 | 934 | 17653 | 2.78858777688068 | 0.0045523191531154 | 0.0464411451882046 | 2.34176729811165 | 0.00963597430406852 | GDF11//INHBB//BMP3//BMPR2//TGFB2//ACVR1//PMEPA1//LDLRAD4//NOG// |
| GO:0000289 | nuclear-transcribed mRNA poly(A) tail shortening | Biological process | 7 | 40 | 934 | 17653 | 3.30757494646681 | 0.0045733849911791 | 0.0464411451882046 | 2.33976223782619 | 0.00749464668094218 | TOB1//CPEB3//CNOT7//BTG2//CNOT6L//PAN3//CNOT6// |
| GO:0003170 | heart valve development | Biological process | 7 | 40 | 934 | 17653 | 3.30757494646681 | 0.0045733849911791 | 0.0464411451882046 | 2.33976223782619 | 0.00749464668094218 | TGFB2//EFNA1//TGFBR2//ACVR1//BMPR2//SOX4//ERG// |
| GO:0010613 | positive regulation of cardiac muscle hypertrophy | Biological process | 7 | 40 | 934 | 17653 | 3.30757494646681 | 0.0045733849911791 | 0.0464411451882046 | 2.33976223782619 | 0.00749464668094218 | EDN1//IGF1//TRPC3//MTPN//TWF1//NR4A3//HAND2// |
| GO:0014742 | positive regulation of muscle hypertrophy | Biological process | 7 | 40 | 934 | 17653 | 3.30757494646681 | 0.0045733849911791 | 0.0464411451882046 | 2.33976223782619 | 0.00749464668094218 | MTPN//EDN1//IGF1//TWF1//NR4A3//HAND2//TRPC3// |
| GO:0048593 | camera-type eye morphogenesis | Biological process | 13 | 108 | 934 | 17653 | 2.27505155047982 | 0.0045837643687565 | 0.0464767592592207 | 2.33877771564342 | 0.0139186295503212 | SOX11//TFAP2A//TOPORS//FJX1//LHX1//FOXF2//LRP6//ZEB1//DLL1//MAN2A1//TBC1D20//EPHA2//GDF11// |
| GO:0043409 | negative regulation of MAPK cascade | Biological process | 18 | 173 | 934 | 17653 | 1.96651854785805 | 0.00474772250509719 | 0.0480671366795992 | 2.32351467262117 | 0.019271948608137 | DUSP10//SPRED1//DUSP1//DUSP2//DUSP5//PPP2CA//RGS3//HIPK3//AIDA//ZMYND11//TAOK3//RANBP9//ERRFI1//LMO3//PTEN//KLF4//EFNA1//NF2// |
| GO:0034330 | cell junction organization | Biological process | 25 | 270 | 934 | 17653 | 1.75003965421524 | 0.00476761216340035 | 0.0481466265965781 | 2.32169908089909 | 0.0267665952890792 | GRHL1//GJC1//CDH20//ITGA6//ITGA5//GJA1//GJB2//LAMA3//CADM2//RASSF8//RHOC//NF2//TGFB2//DLC1//ARHGAP6//EFNA5//SLK//KDR//TSC1//PPM1F//PTEN//STRN//RAP1B//TNF//CBFB// |
| GO:0045598 | regulation of fat cell differentiation | Biological process | 14 | 121 | 934 | 17653 | 2.18682641088715 | 0.00476981218685603 | 0.0481466265965781 | 2.32149872113135 | 0.0149892933618844 | E2F1//ZFPM2//ZADH2//TGFB1I1//TNF//CEBPB//CMKLR1//CCDC71L//TMEM64//FRZB//PPARG//LMO3//DUSP10//PRDM16// |
| GO:0021700 | developmental maturation | Biological process | 24 | 256 | 934 | 17653 | 1.77191514989293 | 0.00481382003379971 | 0.0485184275537816 | 2.31751014999549 | 0.0256959314775161 | EREG//YTHDF2//S1PR1//MMP2//RECK//GJA1//GNAQ//NTN4//TDRD5//IGF1//RAB38//FAM20C//NR4A2//KLF2//GDF11//LHX6//PPARG//CBFB//TGFB2//NFIA//SYBU//PTEN//CDKN1A//SLC17A7// |
| GO:0070371 | ERK1 and ERK2 cascade | Biological process | 29 | 328 | 934 | 17653 | 1.67107445030553 | 0.00482277935619191 | 0.0485363940266754 | 2.31670260674498 | 0.0310492505353319 | EPHA2//EPHA7//RANBP9//DUSP10//SPRED1//DUSP1//ERRFI1//LMO3//PTEN//KLF4//EPHA4//EPHA5//NPNT//DSTYK//NPTN//JUN//KDR//MET//P2RY1//DNAJC27//FBXW7//CCL2//CCL8//TNF//HAND2//IGF1//ITGAV//MAPK1//PTGER4// |
| GO:0048634 | regulation of muscle organ development | Biological process | 16 | 147 | 934 | 17653 | 2.05718947107751 | 0.00487841881242265 | 0.0490233973676291 | 2.31172091822558 | 0.0171306209850107 | MTPN//GJA1//DLL1//PRKAA1//SOX17//JARID2//PTEN//TGFBR2//NOG//ZFPM2//GATA6//EDN1//IGF1//NLN//NRG1//NEUROG1// |
| GO:0060291 | long-term synaptic potentiation | Biological process | 10 | 73 | 934 | 17653 | 2.58909976240063 | 0.00492507731512826 | 0.0494188395878523 | 2.30758694744657 | 0.0107066381156317 | PAIP2//EPHA4//NPTN//SQSTM1//DRD1//SLC24A2//MAPK1//PTEN//TSHZ3//SNAP25// |
| GO:0033036 | macromolecule localization | Biological process | 186 | 2955 | 934 | 17653 | 1.18967162686551 | 0.00497606771672206 | 0.0497784057656487 | 2.3031137181889 | 0.199143468950749 | RANBP6//IPO5//TNF//DRD1//STAT3//BCL2L11//PMAIP1//MOAP1//PKD2//THRA//TWF1//CEP128//MAPRE3//KIF5A//SQSTM1//ZFAND6//SRSF2//NUP35//AGFG1//NUP43//TSC1//PAN3//YWHAG//EGR2//ZDHHC1//ARL6IP1//RPL15//MTX3//IMMP2L//MID1IP1//VLDLR//APOO//STX6//SEC23A//RAB12//STX2//RAB21//TBC1D12//RAB38//SNX5//ARFIP1//RAB30//NSF//SAR1B//RAB9B//DNAJC27//RAB23//TMED9//CTSA//RAB22A//KIF13A//SNX2//EVI5//TBC1D17//AP1S2//RAB28//ZFYVE9//USP6NL//G3BP2//RAPGEF4//MARCKS//NEUROD1//SNAP25//PER2//CLOCK//CBLN4//MIA3//FBXW7//PPARG//ITGAV//TRIM3//EXOC5//VPS37A//DENND1B//LCA5//EPS15//NECAP1//SERP1//CHMP3//ATG16L1//UEVLD//ANKRD50//ZMAT3//AKTIP//BTF3//TSG101//PLEKHF2//FAM160A2//C5ORF30//MCFD2//PKDCC//ARRDC4//CEP41//OSBPL5//LDLR//GNPTG//PEX5L//DERL2//NPLOC4//GJA1//SOX4//TRAPPC8//EDN1//MMD//WASL//CDKN1A//IGF1//MAPK1//MZT1//ARHGAP1//MTBP//TOPORS//PIKFYVE//CNEP1R1//FRMD6//LATS2//RRAGD//LAMTOR3//SYBU//MCOLN2//MDFIC//LYPLA1//GOLGA4//C16ORF70//ACSL1//CLIP1//WNT7A//DZIP1//NPNT//PLEKHA1//INHBB//REST//CTDSPL2//PTGER4//TGFB2//ADAM9//IL2//FMR1//QKI//WWP2//NDFIP2//MORC3//SKP1//EFNA5//EPHA5//RNF128//PLEK//ESCO2//ESR1//RAD21//NEDD1//BAG6//FCHO2//EFR3A//LRP6//P2RY1//MIEF1//RAB3GAP1//GLRB//NF2//CD2AP//DYNLL2//E2F1//PPP3R1//MSN//APPL1//AR//PPFIA1//EPHA2//ARL5A//CCNE2//NPTN//NRG1//ELMOD1//PRKAA1//SNIP1//EDEM1//ATXN3//CCT6A//DCP2//CENPQ//TGFBR2//TNFRSF21//MYB//TMF1//CD274// |
| GO:0060997 | dendritic spine morphogenesis | Biological process | 8 | 51 | 934 | 17653 | 2.96477306125876 | 0.00501051792895946 | 0.0497784057656487 | 2.30011737950936 | 0.00856531049250535 | EPHA4//EFNA1//PTEN//CAPRIN2//LRP8//CTNND2//WNT7A//WASL// |
| GO:0016239 | positive regulation of macroautophagy | Biological process | 9 | 62 | 934 | 17653 | 2.74361055467293 | 0.00508141243543539 | 0.0497784057656487 | 2.29401555392568 | 0.00963597430406852 | CALCOCO2//FYCO1//RAB3GAP1//SESN3//RAB12//SESN1//KDR//BNIP3//TSC1// |
| GO:0007398 | ectoderm development | Biological process | 5 | 22 | 934 | 17653 | 4.29555187852832 | 0.00508332738395872 | 0.0497784057656487 | 2.29385191932186 | 0.00535331905781585 | LHX1//ITGA6//STX2//AMER2//NF2// |
| GO:0008045 | motor neuron axon guidance | Biological process | 5 | 22 | 934 | 17653 | 4.29555187852832 | 0.00508332738395872 | 0.0497784057656487 | 2.29385191932186 | 0.00535331905781585 | EGR2//EPHA4//LHX1//NOG//SLIT2// |
| GO:0021895 | cerebral cortex neuron differentiation | Biological process | 5 | 22 | 934 | 17653 | 4.29555187852832 | 0.00508332738395872 | 0.0497784057656487 | 2.29385191932186 | 0.00535331905781585 | ARX//DRD1//LHX6//HPRT1//C21ORF91// |
| GO:0043090 | amino acid import | Biological process | 5 | 22 | 934 | 17653 | 4.29555187852832 | 0.00508332738395872 | 0.0497784057656487 | 2.29385191932186 | 0.00535331905781585 | ARL6IP1//KCNJ10//SLC17A7//SLC6A1//PER2// |
| GO:0060487 | lung epithelial cell differentiation | Biological process | 5 | 22 | 934 | 17653 | 4.29555187852832 | 0.00508332738395872 | 0.0497784057656487 | 2.29385191932186 | 0.00535331905781585 | GATA6//NFIB//KLF2//THRA//PPP3R1// |
| GO:0071218 | cellular response to misfolded protein | Biological process | 5 | 22 | 934 | 17653 | 4.29555187852832 | 0.00508332738395872 | 0.0497784057656487 | 2.29385191932186 | 0.00535331905781585 | KLHL15//BAG6//ATXN3//DNAJB12//UBE2W// |
| GO:0060746 | parental behavior | Biological process | 4 | 14 | 934 | 17653 | 5.40012236157846 | 0.00508662267259418 | 0.0497784057656487 | 2.29357047728669 | 0.00428265524625268 | CREBRF//DRD1//GNAQ//PTEN// |
| GO:0071361 | cellular response to ethanol | Biological process | 4 | 14 | 934 | 17653 | 5.40012236157846 | 0.00508662267259418 | 0.0497784057656487 | 2.29357047728669 | 0.00428265524625268 | GLRA1//UGT1A1//PRKAA1//PTEN// |
| GO:0090153 | regulation of sphingolipid biosynthetic process | Biological process | 4 | 14 | 934 | 17653 | 5.40012236157846 | 0.00508662267259418 | 0.0497784057656487 | 2.29357047728669 | 0.00428265524625268 | PRKAA1//ORMDL3//TNF//NSMAF// |
| GO:1905038 | regulation of membrane lipid metabolic process | Biological process | 4 | 14 | 934 | 17653 | 5.40012236157846 | 0.00508662267259418 | 0.0497784057656487 | 2.29357047728669 | 0.00428265524625268 | PRKAA1//ORMDL3//TNF//NSMAF// |
| GO:2000303 | regulation of ceramide biosynthetic process | Biological process | 4 | 14 | 934 | 17653 | 5.40012236157846 | 0.00508662267259418 | 0.0497784057656487 | 2.29357047728669 | 0.00428265524625268 | PRKAA1//ORMDL3//TNF//NSMAF// |
| GO:0071560 | cellular response to transforming growth factor beta stimulus | Biological process | 22 | 229 | 934 | 17653 | 1.81576166743031 | 0.00509138957918954 | 0.0497784057656487 | 2.29316367066664 | 0.0235546038543897 | COL1A2//JUN//TGFB2//TGFBR2//KLF10//ADAM9//ACVR1//ZFYVE9//DKK3//SOX11//ZEB1//NPNT//TGFB1I1//BAMBI//ASPN//PMEPA1//PRDM16//RASL11B//LDLRAD4//CRK//EDN1//WNT7A// |
| GO:0003209 | cardiac atrium morphogenesis | Biological process | 6 | 31 | 934 | 17653 | 3.65814740623057 | 0.0050933989043071 | 0.0497784057656487 | 2.29299230945994 | 0.00642398286937901 | SOX4//TGFB2//ACVR1//NOG//BMPR2//SOS1// |
| GO:0010922 | positive regulation of phosphatase activity | Biological process | 6 | 31 | 934 | 17653 | 3.65814740623057 | 0.0050933989043071 | 0.0497784057656487 | 2.29299230945994 | 0.00642398286937901 | NPNT//PLEK//CALM2//PPP1R15B//PPP1R12A//MTMR9// |
| GO:0045736 | negative regulation of cyclin-dependent protein serine/threonine kinase activity | Biological process | 6 | 31 | 934 | 17653 | 3.65814740623057 | 0.0050933989043071 | 0.0497784057656487 | 2.29299230945994 | 0.00642398286937901 | CDK6//PTEN//CDKN1A//LATS2//IPO5//KAT2B// |
| GO:0016331 | morphogenesis of embryonic epithelium | Biological process | 16 | 148 | 934 | 17653 | 2.04328954221888 | 0.00520921520477313 | 0.0508368289031467 | 2.28322770048344 | 0.0171306209850107 | NOG//SOX4//SOX11//DLC1//LRP6//PRKACB//TGFB2//TSC1//FZD6//HAND1//TFAP2A//RDH10//AR//SULF1//OSR1//TGFB1I1// |
| GO:0046578 | regulation of Ras protein signal transduction | Biological process | 23 | 244 | 934 | 17653 | 1.78159774634044 | 0.00534236704521933 | 0.0520611359176057 | 2.27226627725862 | 0.0246252676659529 | ARAP2//APPL1//AGFG1//AGFG2//PSD//GIT2//RALGPS2//CRK//FGD4//FGD5//EPS8//ARHGEF4//ARHGEF28//SOS1//SOS2//DLC1//MET//LPAR1//RASGEF1A//NRG1//IGF1//TGFB2//SQSTM1// |
| GO:0048193 | Golgi vesicle transport | Biological process | 31 | 360 | 934 | 17653 | 1.62753687842018 | 0.00535880312090452 | 0.0521461661966579 | 2.27093219837259 | 0.0331905781584582 | CNIH1//SEC23A//DCTN6//TBC1D20//DYNLL2//DYNC1LI2//TRAPPC8//GRIA1//MIA3//NSF//IER3IP1//SAR1B//TMED9//MCFD2//KIF5A//ERGIC2//KIF23//GOLGA3//EXOC5//MYO1B//OSBPL5//STEAP2//EPS15//SNX2//LYPLA1//PKDCC//GOLGA4//KIF13A//C16ORF70//PPP6C//STX6// |
| GO:0014031 | mesenchymal cell development | Biological process | 10 | 74 | 934 | 17653 | 2.5541119277736 | 0.00543161057066671 | 0.0527787101859468 | 2.2650713751549 | 0.0107066381156317 | SEMA4B//ACVR1//HAND2//RDH10//EDN1//FOXC1//NRG1//SOX11//KBTBD8//MAPK1// |
| GO:1902807 | negative regulation of cell cycle G1/S phase transition | Biological process | 14 | 123 | 934 | 17653 | 2.15126825786459 | 0.00551894690165362 | 0.053550413050048 | 2.25814378430578 | 0.0149892933618844 | CDKN1A//GADD45A//E2F1//CNOT6L//CNOT7//MDM4//CNOT6//RBL2//SOX4//BTG2//PKD2//PTEN//ZNF655//KLF4// |
| GO:0006914 | autophagy | Biological process | 39 | 482 | 934 | 17653 | 1.52928776422295 | 0.00562286253363722 | 0.0544026027396116 | 2.25004253388568 | 0.0417558886509636 | UBXN2B//TRAPPC8//RAB23//ATG16L1//ULK2//BNIP3//SQSTM1//RRAGD//MET//MTMR9//PIK3CB//PRKAA1//VPS37A//DYNLL2//CHMP3//TSG101//PLEKHF2//PLAA//SESN3//RAB12//SESN1//KDR//TSC1//SNX5//RHEB//LAMTOR3//ACBD5//WDFY3//CALCOCO2//FYCO1//FEZ2//FBXW7//CTSA//PIKFYVE//TBC1D12//RAB3GAP1//NRBF2//TBC1D17//TECPR2// |
| GO:0061919 | process utilizing autophagic mechanism | Biological process | 39 | 482 | 934 | 17653 | 1.52928776422295 | 0.00562286253363722 | 0.0544026027396116 | 2.25004253388568 | 0.0417558886509636 | UBXN2B//TRAPPC8//RAB23//ATG16L1//ULK2//BNIP3//SQSTM1//RAB12//NRBF2//PIK3CB//TBC1D17//TECPR2//RRAGD//MET//MTMR9//PRKAA1//VPS37A//DYNLL2//CHMP3//TSG101//PLEKHF2//PLAA//SESN3//SESN1//KDR//TSC1//SNX5//RHEB//LAMTOR3//ACBD5//WDFY3//CALCOCO2//FYCO1//FEZ2//FBXW7//CTSA//PIKFYVE//TBC1D12//RAB3GAP1// |
| GO:0030042 | actin filament depolymerization | Biological process | 8 | 52 | 934 | 17653 | 2.9077581946961 | 0.00565333083897385 | 0.0546192520914002 | 2.24769559841809 | 0.00856531049250535 | EPS8//TWF1//CFL2//LIMA1//PLEK//TMOD3//TMOD1//MTPN// |
| GO:0034976 | response to endoplasmic reticulum stress | Biological process | 25 | 274 | 934 | 17653 | 1.72449163006612 | 0.00574086975949026 | 0.0553858804328568 | 2.24102230566265 | 0.0267665952890792 | WFS1//PPP1R15B//UBXN4//DNAJB9//DERL2//JKAMP//NPLOC4//BAG6//EDEM1//CREBRF//SERP1//NCK1//CCL2//DNAJB12//CEBPB//MAP3K5//BCL2L11//SERINC3//PMAIP1//ATXN3//JUN//FAM129A//NRBF2//TMX3//UBA5// |
| GO:0043066 | negative regulation of apoptotic process | Biological process | 66 | 911 | 934 | 17653 | 1.3692955713772 | 0.00576881122661886 | 0.055576168555019 | 2.23891367227069 | 0.0706638115631692 | IL2//HAND2//DLL1//CCL2//EDN1//IGF1//LRP6//ARL6IP1//KLF4//FNIP1//CEBPB//EN1//JUN//NTF3//NR4A2//POU4F1//WFS1//BTG2//NR4A3//CPEB4//EFNA1//BAG5//FOXC1//KDR//MAP2K4//NEUROD1//ITGA5//NOG//ZMYND11//ITGA6//AR//ITGAV//ACVR1//TNF//URI1//HIPK3//TOPORS//CDKN1A//ZNF268//OSR1//DUSP1//EPHA2//EPHA4//EPHA5//EPHA7//FHL2//DSTYK//GATA6//MDM4//MET//ZFAND6//PRKAA1//SPHK2//PTEN//RARB//BCL11B//BNIP3//STAT3//ACTC1//TFAP2A//TMF1//WNT7A//BAG6//HMGA2//SQSTM1//CCND2// |
| GO:0045787 | positive regulation of cell cycle | Biological process | 32 | 377 | 934 | 17653 | 1.6042803832806 | 0.00582233765020984 | 0.0558933309784595 | 2.23490261247143 | 0.0342612419700214 | CDKN1A//GADD45A//E2F1//CNOT6L//CNOT7//MDM4//CNOT6//RBL2//SOX4//BTG2//MTBP//NEUROG1//PKD2//KIF23//CCNY//MAPRE3//CCNC//CCND2//EDN1//EREG//IGF1//RAD21//ZNF268//GATA6//POU4F1//HMGA2//TMOD3//BCL2L11//LRP6//TGFB2//NR4A3//FOSL1// |
| GO:0008360 | regulation of cell shape | Biological process | 17 | 163 | 934 | 17653 | 1.97121030990134 | 0.00582304032449532 | 0.0558933309784595 | 2.23485020239667 | 0.0182012847965739 | DLC1//CDC42EP3//WASF3//PALM2//FGD4//CRK//FGD5//LPAR1//EPS8//BAMBI//KDR//RHOC//RND3//MSN//BRWD1//CCL2//WASL// |
| GO:0010976 | positive regulation of neuron projection development | Biological process | 24 | 260 | 934 | 17653 | 1.74465491681766 | 0.00582652644385834 | 0.0558933309784595 | 2.23459027814748 | 0.0256959314775161 | GOLGA4//NRG1//EFNA5//LPAR3//BMPR2//SLIT2//EPHA4//RAB21//CAPRIN2//LPAR1//CPEB3//FMR1//IL2//C21ORF91//LRP8//VLDLR//PLAA//STMN2//NPTN//NCK1//NME1//SERPINI1//TWF1//ZNF804A// |
| GO:0035051 | cardiocyte differentiation | Biological process | 16 | 150 | 934 | 17653 | 2.01604568165596 | 0.00592551955347072 | 0.0567624486404001 | 2.22727356442115 | 0.0171306209850107 | HAND2//TGFB2//GATA6//ACTC1//NRG1//TSC1//RARB//FHL2//REST//SOX17//ACVR1//MAP2K4//EDN1//IGF1//MAPK1//DLL1// |
| GO:0008584 | male gonad development | Biological process | 15 | 137 | 934 | 17653 | 2.06938995607934 | 0.00596665616502167 | 0.0570756656917137 | 2.22426898812142 | 0.0160599571734475 | AR//PLEKHA1//TMF1//TCF21//TBC1D20//ARID4B//ARID4A//ZFPM2//BCL2L11//RNF38//ESR1//GATA6//TFAP2C//TGFB2//ARID5B// |
| GO:0010661 | positive regulation of muscle cell apoptotic process | Biological process | 6 | 32 | 934 | 17653 | 3.54383029978587 | 0.00599362693726619 | 0.0572526821705244 | 2.22231029244291 | 0.00642398286937901 | MAP3K5//PTEN//BNIP3//MAP2K4//E2F3//PPARG// |
| GO:0010769 | regulation of cell morphogenesis involved in differentiation | Biological process | 25 | 275 | 934 | 17653 | 1.71822075141133 | 0.00600838437138877 | 0.057312698876872 | 2.22124229221002 | 0.0267665952890792 | DPYSL2//RAB21//EPHA4//WNT7A//GOLGA4//NRG1//EPHA7//ULK2//EFNA5//LPAR3//FMR1//NSMF//HECW2//FXR1//BMPR2//SEMA4B//EFNA1//POU3F2//PTEN//SLIT2//CAPRIN2//LRP8//CRK//CRKL//DOCK5// |
| GO:0010717 | regulation of epithelial to mesenchymal transition | Biological process | 11 | 87 | 934 | 17653 | 2.3897093209284 | 0.00606153584020483 | 0.0576570561002887 | 2.2174173226295 | 0.0117773019271949 | FOXC1//BAMBI//TGFB1I1//TGFB2//TGFBR2//EFNA1//PPP2CA//PTEN//LDLRAD4//NOG//ACVR1// |
| GO:0048013 | ephrin receptor signaling pathway | Biological process | 11 | 87 | 934 | 17653 | 2.3897093209284 | 0.00606153584020483 | 0.0576570561002887 | 2.2174173226295 | 0.0117773019271949 | CRK//EFNA1//EFNA5//EPHA2//EPHA4//EPHA5//EPHA7//MMP2//NCK1//ARHGEF28//WASL// |
| GO:0097190 | apoptotic signaling pathway | Biological process | 46 | 593 | 934 | 17653 | 1.46613777439145 | 0.00607914962617943 | 0.0577433833172071 | 2.21615716718895 | 0.0492505353319058 | JUN//PMAIP1//BCL2L11//MOAP1//TNF//BNIP3//TOPORS//E2F1//EPHA2//MAP3K5//CDKN1A//USP28//BAG6//CEBPB//POU4F1//ZMAT3//IGF1//TGFB2//SGPP1//APPL1//MKNK2//IL2//ITGAV//ITM2B//PPM1F//DYNLL2//PPP3R1//YWHAG//BAG5//PIAS4//WFS1//SERINC3//NCK1//FBXW7//PTEN//NR4A2//NOG//INHBB//ZMYND11//ITGA6//AR//ACVR1//URI1//ST18//FASTK//TNFRSF21// |
| GO:0031589 | cell-substrate adhesion | Biological process | 28 | 319 | 934 | 17653 | 1.65897175998335 | 0.00611352374171957 | 0.0579884446917945 | 2.21370839688233 | 0.0299785867237687 | MINK1//PIK3CB//TSC1//NF2//CDK6//BCL2L11//NPNT//ITGA6//ITGAV//PPFIA1//ADAM9//SGCE//ATXN3//FOXF1//ITGA5//SMOC2//PPM1F//SPOCK1//EFNA1//DLC1//ARHGAP6//EFNA5//SLK//KDR//PTEN//CRK//CRKL//DOCK5// |
| GO:0032501 | multicellular organismal process | Biological process | 434 | 7496 | 934 | 17653 | 1.09428840277984 | 0.00616191686034186 | 0.0583656074600728 | 2.21028416569749 | 0.464668094218415 | WNT7A//GDF11//CMKLR1//COL1A2//HAPLN1//EN1//FRZB//SERP1//GNAQ//IGF1//PTHLH//SHOX//BMP3//SOX4//SOX11//TGFB2//KLF10//PKDCC//NOG//THRA//RUNX3//S1PR1//EFNA1//EPHA2//ELK3//EREG//FOXC1//GAB1//CLIC4//HOXA3//ITGA5//ITGAV//JUN//KDR//MEOX2//MMP2//TMEM100//PTEN//CCL2//SOX17//VEGFB//HAND1//HAND2//CEBPB//INHBB//DMRTA1//IMMP2L//KMT2B//ESR1//PLEKHA1//YTHDF2//CRKL//FOXF1//SPHK2//TGFBR2//EDN1//PPP3R1//ACVR1//GJC1//ZFPM2//QKI//FHL2//GJA1//MEF2D//CBFB//SMOC1//OSR1//LHX1//RDH10//NPNT//RARB//TCF21//SLIT2//PKD2//ADRB1//DRD1//ARRDC3//STAT3//USP46//TBC1D20//AGFG1//TMF1//CHRM5//BCL2L11//ADAM10//KLF2//GATA6//EPN1//AR//ZNF568//MAN2A1//MBNL1//BTF3//FOSL1//LRP6//RNF2//DUSP1//DUSP2//DUSP5//NF2//BMPR2//NR4A3//SEMA4B//DLL1//DCX//GPM6A//LRP12//NR4A2//SPOCK1//UNK//MAF//RPS6KA4//RPS6KA5//TNF//SULF1//TFAP2A//TSC1//WFS1//BAG6//ARID5B//LATS2//RBBP8//FOXF2//HMGA2//DLC1//PRKACB//FZD6//JARID2//MET//UGT1A1//PPARG//NANOS1//APCDD1//SOS1//FBXW7//RECK//GLRA1//GLRB//NRG1//TBL1XR1//CALM2//NFIB//PBX3//TSHZ3//ESCO2//PLEK//RBM47//REST//NKX2-3//FNIP1//F3//MARCH7//NME1//SGCE//ADCY3//PRKAR2B//ERG//POU4F1//CDK6//NEUROD1//PALLD//BCL11B//SLC17A7//BNC2//TMOD3//HSBP1//ASPH//TMOD1//CALD1//ENPP4//DOCK9//IFNA1//IRF1//NFE2//CHRM2//MYBL1//SYCP3//MASTL//TSHZ1//EPHA4//EPHA5//EPHA7//PRR15//DZIP1//WDR47//WDFY3//FLII//LCLAT1//TNFRSF21//CDH20//HIVEP2//HMGB3//SHISA2//OSM//MINK1//KLF3//ZBTB7A//ZFR//BTBD7//TRERF1//RBBP7//NKX2-4//TEF//TGIF1//ZNF3//ZNF217//PTP4A1//EVI5//VANGL1//ST6GAL2//ADAM18//CRTAP//DDX6//E2F1//GOLGA3//CLDN11//ACOX1//PAIP2//ARID4B//ARID4A//HERPUD2//BTG1//CLOCK//TDRD5//TOB2//NEUROG1//SOSTDC1//ZEB1//E2F5//STX2//FLI1//BHLHE41//TRIM3//FUT9//SCRG1//RBFOX3//DPYSL2//EFNA5//MYT1L//FMR1//MYLIP//ATXN3//NTF3//TAOK3//POU3F2//VLDLR//ZIC5//ITM2B//FEZ2//NTNG1//SLITRK3//NEFH//OGN//DSCAML1//RANBP9//ARX//GRB2//KIF5A//OTX2//PIK3CB//ENAH//MAPK1//NTN4//KLF7//NHLH2//NPAS2//SERPINI1//FXR1//B3GNT5//EGR2//RAB3GAP1//CIC//PTPRG//ATXN1//KLF4//ADIPOR2//KAT2B//ZBTB18//CFL2//ETS1//CLCN5//UGT1A7//SLC24A2//KCNJ10//LRIG1//GJB2//SOBP//OR6A2//LHX8//SLC12A5//SLC6A1//KCNK10//CPEB3//GRIA1//LDLR//CCND2//SCN1A//RNF180//SNAP25//BTG2//EPS8//P2RY1//HS3ST5//NPTN//RNF38//TFAP2C//IDH1//MORC3//ACO1//RAI2//NR5A2//EMX2//AIDA//MTPN//TWF1//DUSP10//PFN2//DOCK5//PPM1F//BAMBI//TGFB1I1//PPP2CA//LDLRAD4//TOPORS//FJX1//ZNF804A//STMN2//NCK1//LPAR1//BAG5//WASF3//KBTBD8//FLRT1//STRN//SPRED1//PER2//HOXD1//MNX1//LRP8//HPRT1//SLC32A1//EZH1//LHX6//C21ORF91//TACC2//BNIP3//PRDM16//TCF7L1//MSN//ACTC1//MKNK2//DGKH//TRPC3//DGKE//FZD10//KLF6//DNAJB9//TSG101//ADAM9//IL2//PTGER4//ASPN//OSTM1//DKK3//FAM20C//RAB21//KIAA1109//KRT27//CDKN1A//FRY//PSD//DSC2//PIKFYVE//NPLOC4//POLR3G//CD274//PDCD1LG2//ERRFI1//SOCS5//CD2AP//CRY2//BHLHE40//DENND1B//ITGA6//FOXN2//MCOLN2//LAMA3//ZNF287//RNF128//PAX9//SBF2//CREBRF//TMEM64//GRHL1//MYB//DPY30//YWHAG//HMG20A//DNMT3B//MMD//TOB1//EGLN1//VASH2//ITGB8//ADAM12//GOLGA4//LHFPL2//PHF14//TEAD1//PRKAA1//ULK2//LPAR3//RGMA//SLC35D1//RHEB//NSMF//HECW2//CXCL14//CAPRIN2//MDM1//NFIA//SYBU//MFAP5//CTNND2//WASL//MAP2K4//NEFM//KLHL3//KCNJ3//RAB23//C5ORF30//NLN//JAK1//PLAA// |
| GO:0016241 | regulation of macroautophagy | Biological process | 17 | 164 | 934 | 17653 | 1.95919073484097 | 0.00618632754753464 | 0.0584781958229443 | 2.20856708913063 | 0.0182012847965739 | SESN3//RAB12//SESN1//KDR//BNIP3//TSC1//CALCOCO2//FYCO1//FEZ2//PIKFYVE//TBC1D12//RAB3GAP1//SNX5//PRKAA1//RRAGD//RHEB//LAMTOR3// |
| GO:0009226 | nucleotide-sugar biosynthetic process | Biological process | 5 | 23 | 934 | 17653 | 4.10878875337492 | 0.00621703723150924 | 0.0584781958229443 | 2.20641653179694 | 0.00535331905781585 | NAGK//GFPT2//SLC35D1//UGP2//UXS1// |
| GO:0015813 | L-glutamate transmembrane transport | Biological process | 5 | 23 | 934 | 17653 | 4.10878875337492 | 0.00621703723150924 | 0.0584781958229443 | 2.20641653179694 | 0.00535331905781585 | ARL6IP1//KCNJ10//PER2//SLC17A7//SLC17A6// |
| GO:0060479 | lung cell differentiation | Biological process | 5 | 23 | 934 | 17653 | 4.10878875337492 | 0.00621703723150924 | 0.0584781958229443 | 2.20641653179694 | 0.00535331905781585 | GATA6//NFIB//PPP3R1//KLF2//THRA// |
| GO:0090140 | regulation of mitochondrial fission | Biological process | 5 | 23 | 934 | 17653 | 4.10878875337492 | 0.00621703723150924 | 0.0584781958229443 | 2.20641653179694 | 0.00535331905781585 | KDR//MIEF1//BNIP3//DDHD1//TMEM135// |
| GO:0060038 | cardiac muscle cell proliferation | Biological process | 9 | 64 | 934 | 17653 | 2.6578727248394 | 0.00628152690877893 | 0.0590027312278776 | 2.201934775458 | 0.00963597430406852 | GJA1//JARID2//PTEN//TGFBR2//NOG//ZFPM2//GATA6//FOXC1//TGFB2// |
| GO:0040017 | positive regulation of locomotion | Biological process | 42 | 532 | 934 | 17653 | 1.49213907359405 | 0.00634975250621452 | 0.059403716960888 | 2.19724320183874 | 0.0449678800856531 | MIA3//CCL8//EDN1//ETS1//KDR//BMPR2//WNT7A//JUN//TGFB2//TGFBR2//DOCK5//PPM1F//CMKLR1//ADAM10//CRK//IGF1//SEMA4B//ZNF268//DRD1//S1PR1//F3//FOXF1//CD274//ITGA6//ITGA5//ITGAV//NTF3//MAPK1//PTP4A1//ACVR1//GAB1//SLIT2//VEGFB//ADAM9//LPAR1//TNF//CHMP3//TSG101//CXCL14//MET//NSMF//PLAA// |
| GO:0048146 | positive regulation of fibroblast proliferation | Biological process | 8 | 53 | 934 | 17653 | 2.85289483253202 | 0.00635705736243336 | 0.059403716960888 | 2.19674386972797 | 0.00856531049250535 | MYB//CDK6//CDKN1A//E2F1//EREG//ESR1//IGF1//JUN// |
| GO:0051641 | cellular localization | Biological process | 174 | 2761 | 934 | 17653 | 1.19111717428514 | 0.00637421221404045 | 0.059403716960888 | 2.19557348183302 | 0.186295503211991 | RANBP6//IPO5//TNF//DRD1//STAT3//BCL2L11//PMAIP1//MOAP1//TWF1//ZFAND6//SRSF2//NUP35//AGFG1//NUP43//TSC1//PAN3//YWHAG//EGR2//ZDHHC1//ARL6IP1//RPL15//MTX3//IMMP2L//MID1IP1//STX6//SEC23A//RAB12//STX2//RAB21//TBC1D12//RAB38//SNX5//ARFIP1//RAB30//NSF//SAR1B//RAB9B//DNAJC27//RAB23//TMED9//CTSA//RAB22A//KIF13A//SNX2//EVI5//TBC1D17//AP1S2//RAB28//ZFYVE9//USP6NL//CNIH1//DCTN6//TBC1D20//DYNLL2//DYNC1LI2//TRAPPC8//GRIA1//MIA3//IER3IP1//MCFD2//EPS15//PLEK//NEUROD1//ANP32E//CDC23//SLC32A1//SNAP25//WNT7A//PPFIA1//FMR1//FXR1//AKTIP//TSG101//FAM160A2//TRPC3//CALM2//VPS37A//ARHGAP1//CHMP3//DPY30//SQSTM1//GNPTG//PEX5L//SYBU//WASL//KIF5A//DERL2//NPLOC4//PKD2//DENND1B//CDKN1A//IGF1//MAPK1//MZT1//ATG16L1//MTBP//TOPORS//PIKFYVE//CNEP1R1//FRMD6//LATS2//RRAGD//LAMTOR3//TMEM87A//ZFYVE21//MDFIC//LYPLA1//PKDCC//GOLGA4//C16ORF70//NR4A3//FOXF1//CLIP1//PPP1R12A//SOX4//CTDSPL2//PPP6C//EXOC5//FCHO2//SYT6//ATXN1//CENPQ//MORC3//SKP1//KCNJ10//PER2//EFNA5//EPHA5//RNF128//RAB3GAP1//ESCO2//ESR1//RAD21//NEDD1//BAG6//FYCO1//KIF23//EFR3A//LRP6//P2RY1//LDLR//MIEF1//GLRB//SLC17A7//PRKAR2B//CEP97//HAUS8//CEP41//SLC17A6//TRPV6//FAM155A//NF2//CD2AP//E2F1//PPP3R1//NEFH//NPTN//MSN//APPL1//AR//EPHA2//ARL5A//PLAA//CCNE2//NRG1//FBXW7//ELMOD1//PRKAA1//SNIP1//EDEM1//CCT6A//ANKRD50//PFN2//PTEN// |
| GO:0010977 | negative regulation of neuron projection development | Biological process | 15 | 138 | 934 | 17653 | 2.05439437668746 | 0.00637691830749737 | 0.059403716960888 | 2.19538914657404 | 0.0160599571734475 | EPHA4//EPHA7//ULK2//RGMA//PTEN//SEMA4B//EFNA1//SLIT2//STMN2//LPAR1//MYLIP//PTPRG//SPOCK1//TSC1//BAG5// |
| GO:0060041 | retina development in camera-type eye | Biological process | 15 | 138 | 934 | 17653 | 2.05439437668746 | 0.00637691830749737 | 0.059403716960888 | 2.19538914657404 | 0.0160599571734475 | RARB//GPM6A//SLC17A7//TGFB2//TOPORS//FJX1//LHX1//TFAP2A//NEUROD1//DLL1//MAN2A1//LRP6//BMPR2//CLIC4//MDM1// |
| GO:1903364 | positive regulation of cellular protein catabolic process | Biological process | 15 | 138 | 934 | 17653 | 2.05439437668746 | 0.00637691830749737 | 0.059403716960888 | 2.19538914657404 | 0.0160599571734475 | RNF180//ANKIB1//LONRF3//LONRF1//SOCS5//FMR1//FBXW7//EDEM1//ATXN3//BAG6//RNF128//LDLR//PTEN//FBXL5//MSN// |
| GO:0030335 | positive regulation of cell migration | Biological process | 39 | 486 | 934 | 17653 | 1.51670103365321 | 0.00642870764204031 | 0.0598037823701769 | 2.19187632417406 | 0.0417558886509636 | MIA3//CCL8//EDN1//ETS1//KDR//BMPR2//WNT7A//JUN//TGFB2//TGFBR2//DOCK5//PPM1F//CMKLR1//ADAM10//CRK//IGF1//GAB1//ADAM9//VEGFB//LPAR1//TNF//CXCL14//MET//NSMF//PLAA//SEMA4B//ZNF268//DRD1//S1PR1//F3//FOXF1//CD274//ITGA6//ITGA5//ITGAV//NTF3//MAPK1//PTP4A1//ACVR1// |
| GO:0003279 | cardiac septum development | Biological process | 12 | 100 | 934 | 17653 | 2.26805139186296 | 0.00647679959627432 | 0.0600858651160538 | 2.1886395405837 | 0.012847965738758 | ZFPM2//GATA6//RARB//BMPR2//TGFB2//TGFBR2//NOG//SOX4//ACVR1//HAND1//EGLN1//SOX11// |
| GO:0030282 | bone mineralization | Biological process | 12 | 100 | 934 | 17653 | 2.26805139186296 | 0.00647679959627432 | 0.0600858651160538 | 2.1886395405837 | 0.012847965738758 | S1PR1//GJA1//OSR1//FAM20C//BMPR2//TFAP2A//ACVR1//PKDCC//IGF1//ASPN//PTHLH//KLF10// |
| GO:0009410 | response to xenobiotic stimulus | Biological process | 25 | 277 | 934 | 17653 | 1.70581482540836 | 0.00657444963809495 | 0.0609082231540221 | 2.18214059708042 | 0.0267665952890792 | DRD1//HPRT1//NR4A2//PTGES3//UGT1A6//UGT1A9//DNMT3B//PPARG//PRKAA1//ACSL1//SLC6A1//UGT1A10//UGT1A8//UGT1A7//UGT1A5//UGT1A4//UGT1A1//UGT1A3//E2F1//EDN1//FIBIN//GJB2//ERRFI1//PRKACB//PRKAR2B// |
| GO:0034616 | response to laminar fluid shear stress | Biological process | 4 | 15 | 934 | 17653 | 5.0401142041399 | 0.00664935954555209 | 0.061350093596956 | 2.17722018315215 | 0.00428265524625268 | KLF2//KLF4//ETS1//ADAM9// |
| GO:0071850 | mitotic cell cycle arrest | Biological process | 4 | 15 | 934 | 17653 | 5.0401142041399 | 0.00664935954555209 | 0.061350093596956 | 2.17722018315215 | 0.00428265524625268 | CDKN1A//GADD45A//DUSP1//CDC14A// |
| GO:0090141 | positive regulation of mitochondrial fission | Biological process | 4 | 15 | 934 | 17653 | 5.0401142041399 | 0.00664935954555209 | 0.061350093596956 | 2.17722018315215 | 0.00428265524625268 | KDR//MIEF1//BNIP3//DDHD1// |
| GO:0030856 | regulation of epithelial cell differentiation | Biological process | 16 | 152 | 934 | 17653 | 1.98951876479207 | 0.00671950489106043 | 0.0618577359411745 | 2.17266272560717 | 0.0171306209850107 | OSR1//DLL1//ZEB1//TMEM100//BTG1//GRHL1//ERRFI1//CBFB//FOXC1//FRZB//CEBPB//TNF//RHEB//CLOCK//LHX1//KIAA1109// |
| GO:0009628 | response to abiotic stimulus | Biological process | 83 | 1198 | 934 | 17653 | 1.30946205846289 | 0.00672267276604514 | 0.0618577359411745 | 2.17245802802233 | 0.088865096359743 | EGLN3//DNMT3B//ETS1//MMP2//MYB//NR4A2//EGLN1//PRKAA1//BNIP3//TGFB2//TGFBR2//VEGFB//CLCN6//JUN//P2RY1//PPARG//PTGER4//BTG2//FOSL1//SLC12A5//OSR1//CDS1//TRPC3//GNAQ//DRD1//NPTN//DNAJB4//IGF1//TGFB1I1//ADRB1//TMEM135//THRA//CRY2//DUSP1//PER2//ELOVL4//CLOCK//TNF//CDKN1A//USP28//NABP1//MAP3K2//MTPN//GADD45A//GJA1//IRF1//MAP2K4//NEUROG1//CETN1//ATXN3//IER5//HSPA13//FMR1//EDN1//CPEB4//BHLHE40//SCN1A//DENND5B//PKD2//KCNJ3//HSBP1//RBBP7//NSMF//PTEN//REST//LPAR1//CPEB2//E2F1//GATA6//MDM4//PMAIP1//CPEB1//SLC2A4//ERRFI1//GRB2//CCND2//TSC1//PTGES3//NUP35//NUP43//MAPK1//BAG5//HMGA2// |
| GO:0009967 | positive regulation of signal transduction | Biological process | 106 | 1586 | 934 | 17653 | 1.26320642884339 | 0.0067881181502089 | 0.0623671406369062 | 2.16825060720038 | 0.113490364025696 | GADD45A//MINK1//TNF//MAP3K2//CRK//CRKL//MAP3K5//TAOK3//LAMTOR3//ADAM9//DUSP5//LPAR1//LPAR3//NRG1//IGF1//NTF3//PIK3CB//PRKAA1//MAPK1//MAP2K4//EREG//FBXW7//COPS8//MDFIC//F3//GDF11//INHBB//BMP3//BMPR2//TGFB2//ACVR1//KDR//SULF1//SOX4//NPNT//TGFB1I1//GATA6//SOX11//ITGA5//PKD2//RRAGD//RHEB//AR//LMO3//IL2//OSM//STAT3//GJA1//RHOC//NDFIP2//CC2D1A//MIER1//SLC20A1//UBE2I//EDN1//EFNA1//EPHA2//EPHA4//EPHA5//EPHA7//ITGAV//MET//FZD10//SPRED1//PMAIP1//ADRB1//SOS1//DSTYK//NPTN//DLL1//KAT2B//WNT7A//RASGEF1A//SOS2//ESR1//GAB1//GRB2//CALM2//JUN//P2RY1//DNAJC27//PTEN//CCL2//CCL8//HAND2//ARRDC3//BCL2L11//MOAP1//BNIP3//BAMBI//LRP6//CAPRIN2//TBL1XR1//DYNLL2//E2F1//PPP3R1//YWHAG//CREBRF//PIAS4//SERINC3//NCK1//ST18//TOB1//EPS8//ARHGAP1//ARHGAP6// |
| GO:2000027 | regulation of organ morphogenesis | Biological process | 23 | 249 | 934 | 17653 | 1.74582269119304 | 0.00679647828617476 | 0.0623671406369062 | 2.16771606627481 | 0.0246252676659529 | BMPR2//HAND2//PAX9//EDN1//SOX17//VANGL1//FZD6//AR//NTN4//SULF1//ESR1//BTBD7//TNF//ASPN//TFAP2A//LHX1//NOG//TGFB2//FOXC1//TGFBR2//ACVR1//FBXW7//NFIB// |
| GO:0035115 | embryonic forelimb morphogenesis | Biological process | 6 | 33 | 934 | 17653 | 3.43644150282266 | 0.00700437423527963 | 0.0640143012881029 | 2.15463065813757 | 0.00642398286937901 | OSR1//RDH10//EN1//TFAP2A//WNT7A//RECK// |
| GO:0043001 | Golgi to plasma membrane protein transport | Biological process | 6 | 33 | 934 | 17653 | 3.43644150282266 | 0.00700437423527963 | 0.0640143012881029 | 2.15463065813757 | 0.00642398286937901 | LYPLA1//PKDCC//GOLGA4//NSF//KIF13A//C16ORF70// |
| GO:1904030 | negative regulation of cyclin-dependent protein kinase activity | Biological process | 6 | 33 | 934 | 17653 | 3.43644150282266 | 0.00700437423527963 | 0.0640143012881029 | 2.15463065813757 | 0.00642398286937901 | CDK6//PTEN//CDKN1A//LATS2//IPO5//KAT2B// |
| GO:0051893 | regulation of focal adhesion assembly | Biological process | 8 | 54 | 934 | 17653 | 2.80006344674439 | 0.00712521276110515 | 0.0649431454223102 | 2.14720216295533 | 0.00856531049250535 | KDR//TSC1//PPM1F//DLC1//ARHGAP6//PTEN//EFNA5//SLK// |
| GO:0090109 | regulation of cell-substrate junction assembly | Biological process | 8 | 54 | 934 | 17653 | 2.80006344674439 | 0.00712521276110515 | 0.0649431454223102 | 2.14720216295533 | 0.00856531049250535 | EFNA5//SLK//KDR//TSC1//PPM1F//DLC1//ARHGAP6//PTEN// |
| GO:0007044 | cell-substrate junction assembly | Biological process | 11 | 89 | 934 | 17653 | 2.33600798787383 | 0.00718718422003487 | 0.0652580233843473 | 2.14344122327749 | 0.0117773019271949 | ITGA6//LAMA3//DLC1//ARHGAP6//EFNA5//SLK//KDR//TSC1//PPM1F//PTEN//ITGA5// |
| GO:0048709 | oligodendrocyte differentiation | Biological process | 11 | 89 | 934 | 17653 | 2.33600798787383 | 0.00718718422003487 | 0.0652580233843473 | 2.14344122327749 | 0.0117773019271949 | WASF3//LPAR1//SOX11//KCNJ10//PTEN//TNFRSF21//PPARG//RHEB//DUSP10//NRG1//BNIP3// |
| GO:0007422 | peripheral nervous system development | Biological process | 10 | 77 | 934 | 17653 | 2.45460107344476 | 0.00719835656435356 | 0.0652580233843473 | 2.14276664474954 | 0.0107066381156317 | EGR2//POU3F2//PPP3R1//POU4F1//RUNX3//HAND2//NEFH//NRG1//NTF3//SERPINI1// |
| GO:0014855 | striated muscle cell proliferation | Biological process | 10 | 77 | 934 | 17653 | 2.45460107344476 | 0.00719835656435356 | 0.0652580233843473 | 2.14276664474954 | 0.0107066381156317 | FOXC1//TGFB2//GJA1//JARID2//PTEN//TGFBR2//NOG//ZFPM2//GATA6//STAT3// |
| GO:0046834 | lipid phosphorylation | Biological process | 13 | 114 | 934 | 17653 | 2.1553119951914 | 0.00722501157116224 | 0.0654119856168276 | 2.14116145302984 | 0.0139186295503212 | PIKFYVE//EREG//ESR1//EFR3A//GAB1//GRB2//NRG1//MET//PIK3CB//ZFYVE21//PIK3R3//SPHK2//DGKE// |
| GO:0040013 | negative regulation of locomotion | Biological process | 30 | 353 | 934 | 17653 | 1.60626869112107 | 0.00729636901225111 | 0.0659164860269264 | 2.13689320977671 | 0.0321199143468951 | SLIT2//DUSP10//PFN2//PTPRG//C5ORF30//DLC1//SULF1//TMEFF2//CLIC4//MIA3//NF2//PTEN//RAP2C//LDLRAD4//RECK//NOG//PPARG//SEMA4B//SPRED1//MEOX2//KLF4//ARRDC3//CHMP3//CRK//WASL//LRCH1//PTGER4//CCL2//NRG1//STAT3// |
| GO:0035304 | regulation of protein dephosphorylation | Biological process | 14 | 127 | 934 | 17653 | 2.08351177730193 | 0.0073002288975555 | 0.0659164860269264 | 2.1366635224293 | 0.0149892933618844 | CRY2//PPP1R37//MASTL//URI1//CALM2//PPP1R15B//DLC1//CNEP1R1//NSMF//PPP1R12A//TNF//UBXN2B//PPP4R2//TSC1// |
| GO:0070647 | protein modification by small protein conjugation or removal | Biological process | 78 | 1119 | 934 | 17653 | 1.31745612574702 | 0.00734245016361662 | 0.0662093206087189 | 2.13415899253955 | 0.0835117773019272 | TOPORS//WWP2//HECTD2//RNF145//ZNRF2//FBXL5//RNF180//MYLIP//RLIM//ANKIB1//UBE2W//FBXW7//HECW2//SKP1//THOP1//LONRF3//FBXO30//CBFB//UBE3B//LONRF1//COPS8//TRIM3//KLHL2//RNF38//MYCBP2//FBXO33//KLHL3//SPOPL//MDM4//DCAF8//DCAF6//FEM1C//KLHL14//MARCH4//RNF4//AKTIP//MARCH7//UBE2Z//RNF128//KLHL15//KBTBD8//ARRDC4//ZFAND4//SOCS5//TSG101//USP3//DMWD//ESR1//OTUD3//USP24//AR//USP27X//ATXN3//PTEN//USP28//USP46//USP45//KAT2B//WDR20//JOSD1//NUP35//EGR2//NUP43//PIAS4//UBE2I//SOX4//PER2//BAG5//NDFIP2//WFS1//HMG20A//RNF2//PCGF5//ARRDC3//RNF141//CDC23//UBA5//PLAA// |
| GO:0031345 | negative regulation of cell projection organization | Biological process | 17 | 167 | 934 | 17653 | 1.92399569170011 | 0.00738777972924296 | 0.0665293665897072 | 2.13148606177002 | 0.0182012847965739 | SLIT2//STMN2//LPAR1//EPHA4//MYLIP//PTPRG//SPOCK1//TSC1//BAG5//CEP97//EPHA7//ULK2//RGMA//PTEN//SEMA4B//EFNA1//PFN2// |
| GO:0001783 | B cell apoptotic process | Biological process | 5 | 24 | 934 | 17653 | 3.9375892219843 | 0.00751710597727946 | 0.0673353479792596 | 2.12394932696247 | 0.00535331905781585 | PTEN//IL2//FNIP1//BCL2L11//TNFRSF21// |
| GO:0002068 | glandular epithelial cell development | Biological process | 5 | 24 | 934 | 17653 | 3.9375892219843 | 0.00751710597727946 | 0.0673353479792596 | 2.12394932696247 | 0.00535331905781585 | CDK6//DLL1//RHEB//CLOCK//RARB// |
| GO:0003309 | type B pancreatic cell differentiation | Biological process | 5 | 24 | 934 | 17653 | 3.9375892219843 | 0.00751710597727946 | 0.0673353479792596 | 2.12394932696247 | 0.00535331905781585 | CDK6//DLL1//RHEB//CLOCK//GATA6// |
| GO:0051788 | response to misfolded protein | Biological process | 5 | 24 | 934 | 17653 | 3.9375892219843 | 0.00751710597727946 | 0.0673353479792596 | 2.12394932696247 | 0.00535331905781585 | ATXN3//DNAJB12//UBE2W//KLHL15//BAG6// |
| GO:0031668 | cellular response to extracellular stimulus | Biological process | 22 | 237 | 934 | 17653 | 1.7544701343525 | 0.00756312786855645 | 0.067657981183925 | 2.12129855699659 | 0.0235546038543897 | DSC2//INHBB//RRAGD//BMPR2//KLF10//PLEKHF2//FNIP1//PRKAA1//CDKN1A//SESN3//SESN1//MAP3K5//MAPK1//PMAIP1//CPEB4//DCTPP1//JUN//PPARG//TSC1//ITGA6//NR4A2//FOSL1// |
| GO:1902905 | positive regulation of supramolecular fiber organization | Biological process | 19 | 195 | 934 | 17653 | 1.84158018997419 | 0.00762941209325126 | 0.0681607846587295 | 2.11750892659217 | 0.0203426124197002 | CFL2//PLEK//CDC42EP3//GRB2//NCK1//PFN2//MET//SLAIN2//CLIP1//STMN2//IQGAP2//ARFIP1//WASL//LPAR1//PPM1E//NF2//TSC1//PPM1F//EDN1// |
| GO:0010611 | regulation of cardiac muscle hypertrophy | Biological process | 9 | 66 | 934 | 17653 | 2.57733112711699 | 0.00768841779302314 | 0.0684476372622376 | 2.11416302488757 | 0.00963597430406852 | MTPN//EDN1//IGF1//TWF1//NR4A3//HAND2//JARID2//ERRFI1//TRPC3// |
| GO:1901565 | organonitrogen compound catabolic process | Biological process | 85 | 1238 | 934 | 17653 | 1.29768691645363 | 0.00769022774546669 | 0.0684476372622376 | 2.1140607984125 | 0.0910064239828694 | ZFAND4//LYPLA1//GNS//PDE10A//DCTPP1//CTSA//TOPORS//USP24//KLHL3//MYLIP//USP27X//ATXN3//RLIM//NPLOC4//USP28//USP46//UBE2I//BAG6//RNF128//KLHL15//USP45//CDC23//SQSTM1//USP3//UBE2W//GLUD2//BCAT2//UGT1A4//UGT1A1//TMF1//PMAIP1//LDLR//UBXN4//DNAJB9//DERL2//JKAMP//WFS1//EDEM1//TGFB1I1//EGLN1//FBXL5//FBXW7//SKP1//BAG5//RNF180//ANKIB1//LONRF3//LONRF1//SOCS5//VLDLR//BNIP3//DNAJB12//NRG1//MDM4//OGN//SLC44A1//WWP2//UBXN2B//RNF38//RNF145//ZNRF2//CD2AP//SPOPL//HECW2//RNF4//TBL1XR1//PLAA//CLOCK//VPS37A//TSG101//MTRR//RAB12//GJA1//NSF//SOX17//TNF//HPRT1//ENPP4//SH3D19//ADAM9//FMR1//EFNA1//EPHA4//MSN//PTEN// |
| GO:0009636 | response to toxic substance | Biological process | 40 | 507 | 934 | 17653 | 1.4911580485621 | 0.00769188293942046 | 0.0684476372622376 | 2.11396733378751 | 0.0428265524625268 | DRD1//HPRT1//NR4A2//EDN1//MAPK1//CRK//DUSP1//JUN//NR4A3//FOSL1//PPP1R15B//ADAM9//IL2//RPL15//SLC2A4//STAT3//ACTC1//EPS8//USP46//KLF2//ETS1//MAP3K5//MYB//PRKAA1//PLEKHA1//BNIP3//KLF4//TNF//GLRA1//UGT1A1//PTEN//SESN1//MET//KDR//CDKN1A//SLC6A14//DNMT3B//PON2//SLC6A1//SNN// |
| GO:0022607 | cellular component assembly | Biological process | 183 | 2934 | 934 | 17653 | 1.1788610676861 | 0.0077445850020221 | 0.0687419613485592 | 2.1110018491973 | 0.19593147751606 | UBXN2B//TRAPPC8//RAB23//ATG16L1//ULK2//WASL//KIF23//TBC1D20//AGFG1//TMF1//NAP1L3//ANP32E//EPS8//RHOC//RND3//MARCKS//MYO1B//LIMA1//TWF1//PTGES3//CD2AP//NCK1//RANBP9//SLAIN2//VBP1//GJC1//CDH20//ITGA6//ITGA5//BAG6//EFNA5//NRG1//ZFYVE9//DRD1//GPM6A//POU4F1//PTEN//WASF3//ARHGAP6//PTPDC1//DYNLL2//TMEM17//DZIP1//GMNC//CDC14A//C5ORF30//CEP41//SKP1//COPS8//EPHA2//SLIT2//CNOT6L//PAN3//CNOT6//PMEPA1//LDLRAD4//SNAP25//GJA1//GJB2//THRA//NEFH//S1PR1//ARHGEF4//TMOD3//TMOD1//ACTC1//TDRD5//PFN2//CDC42EP3//GRB2//STMN2//MET//CLIP1//TNF//FNIP1//BAHD1//HMGA2//LAMA3//LPAR1//LPAR3//PLEK//PIKFYVE//PMAIP1//BCL2L11//IMMP2L//PTGER4//NEFM//DDX6//CNOT7//G3BP2//CENPQ//RBBP7//CENPO//IQGAP2//ARFIP1//PRKACB//DNAJB12//MIA3//KIF13A//VPS37A//CHMP3//TSG101//DYNC1LI2//GLRA1//KCNA4//SLC6A1//CFL2//CEP97//ESR1//MDM1//KAT2B//FGD4//FGD5//DLC1//CNIH1//SEC23A//GRIA1//NSF//SAR1B//PPP6C//MCFD2//FCHO2//EPS15//UXS1//SBF2//GOLGA8A//HAUS8//MID1IP1//KHDRBS3//DGKH//STX2//MCOLN2//AR//SNX2//AHNAK//HPRT1//PKD2//DCTPP1//GLRB//PRKAA1//TSC1//SQSTM1//MZT1//FMR1//PPM1E//NF2//PPM1F//TMEFF2//PPFIA1//SLK//KDR//EPHA7//SLITRK3//FLRT1//WNT7A//EDN1//MDM4//OTX2//TEAD1//COL1A2//STRN//BDP1//CRK//RTN3//ARL6IP1//MSN//BAG5//SACS//RNF4//NEDD1//PRKAR2B//YWHAG//RAP1B//FEZ2//RAB38//TCF7L1//NPTN//JUN//FOSL1//TBC1D12//RAB3GAP1//CBFB//MTPN// |
| GO:0071236 | cellular response to antibiotic | Biological process | 15 | 141 | 934 | 17653 | 2.01068385803453 | 0.00774528678805295 | 0.0687419613485592 | 2.11096249679916 | 0.0160599571734475 | KLF2//ETS1//MAP3K5//MYB//PRKAA1//PLEKHA1//BNIP3//KLF4//GLRA1//UGT1A1//PTEN//MET//NR4A3//ADCY3//EFNA5// |
| GO:0008610 | lipid biosynthetic process | Biological process | 52 | 697 | 934 | 17653 | 1.4100749925499 | 0.00782974134667561 | 0.0693919666414867 | 2.10625258450233 | 0.0556745182012848 | PTGES3//EDN1//ST6GALNAC3//ST6GALNAC6//ST8SIA3//ST3GAL5//RDH10//PIGA//PRKAA1//ELOVL4//SCD5//LCLAT1//DDHD1//CDS1//ABHD3//SLC44A1//PIKFYVE//PIK3CB//PTEN//PLEKHA1//PLEKHA3//MTMR9//PIK3R3//SPHK2//B3GALT2//ST8SIA5//TRERF1//ACBD3//LBR//MSMO1//DGKE//B3GNT5//CNEP1R1//LDLR//FBXW7//ELOVL7//PPM1L//SPTSSA//SGPP1//DKK3//REST//GAB1//GRB2//ADIPOR2//QKI//MID1IP1//ACOT7//TNF//IDH1//ORMDL3//NSMAF//ACSL1// |
| GO:1901214 | regulation of neuron death | Biological process | 25 | 281 | 934 | 17653 | 1.68153276383671 | 0.00783904517434509 | 0.0693919666414867 | 2.10573683282151 | 0.0267665952890792 | EGLN3//NSMF//CEBPB//EN1//JUN//NTF3//NR4A2//POU4F1//CCL2//WFS1//BTG2//NR4A3//CPEB4//BCL2L11//EPHA7//MYB//MAP2K4//TFAP2A//TGFB2//STAT3//BTBD10//MAP3K5//TSC1//FBXW7//EGLN1// |
| GO:0043069 | negative regulation of programmed cell death | Biological process | 66 | 924 | 934 | 17653 | 1.35003059039462 | 0.00785204381679558 | 0.0694161729842987 | 2.10501728556981 | 0.0706638115631692 | IL2//HIPK3//TOPORS//CDKN1A//ZNF268//OSR1//DUSP1//EPHA2//EPHA4//EPHA5//EPHA7//FHL2//ARL6IP1//DSTYK//GATA6//IGF1//KDR//MDM4//MET//ZFAND6//PRKAA1//SPHK2//PTEN//RARB//BCL11B//BNIP3//STAT3//ACTC1//TFAP2A//TMF1//WNT7A//BAG6//HMGA2//SQSTM1//CCND2//HAND2//DLL1//CCL2//EDN1//LRP6//KLF4//FNIP1//CEBPB//EN1//JUN//NTF3//NR4A2//POU4F1//WFS1//BTG2//NR4A3//CPEB4//EFNA1//BAG5//FOXC1//MAP2K4//NEUROD1//ITGA5//NOG//ZMYND11//ITGA6//AR//ITGAV//ACVR1//TNF//URI1// |
| GO:0072401 | signal transduction involved in DNA integrity checkpoint | Biological process | 10 | 78 | 934 | 17653 | 2.42313182891341 | 0.00787655676690282 | 0.0694513082328081 | 2.10366359265487 | 0.0107066381156317 | CDKN1A//GADD45A//E2F1//CNOT6L//CNOT7//MDM4//CNOT6//RBL2//SOX4//BTG2// |
| GO:0072422 | signal transduction involved in DNA damage checkpoint | Biological process | 10 | 78 | 934 | 17653 | 2.42313182891341 | 0.00787655676690282 | 0.0694513082328081 | 2.10366359265487 | 0.0107066381156317 | CDKN1A//GADD45A//E2F1//CNOT6L//CNOT7//MDM4//CNOT6//RBL2//SOX4//BTG2// |
| GO:0000278 | mitotic cell cycle | Biological process | 74 | 1056 | 934 | 17653 | 1.3244618292129 | 0.00788749214221241 | 0.069457173642946 | 2.10306106040651 | 0.0792291220556745 | KIF23//CDK6//CDKN1A//LATS2//PPP6C//RBBP8//ACVR1//CCNE2//E2F1//NPAT//RRM2//NEDD1//CCNY//PPP1R12A//PRKAR2B//SKP1//YWHAG//MASTL//HAUS8//CEP41//RHOC//GADD45A//CNOT6L//CNOT7//MDM4//CNOT6//RBL2//SOX4//BTG2//NUP35//CNEP1R1//NUP43//CDC23//REEP3//PPP2CA//MTBP//NABP1//TAOK3//HMGA2//CDC14A//CDK8//MAP3K2//ZNF268//CDK19//MINK1//PAK6//MAP2K4//SLK//EPS8//RAD21//HECW2//NEUROG1//PTEN//PKD2//EDN1//EREG//IGF1//BTG3//FOXC1//BTG1//TNF//DUSP1//MZT1//CCND2//CEP97//TMOD3//ZNF655//KLF4//MYB//MYBL1//RNF2//CLIP1//SRSF2//USP3// |
| GO:0001541 | ovarian follicle development | Biological process | 8 | 55 | 934 | 17653 | 2.74915320225813 | 0.00796133687351356 | 0.0698144488004959 | 2.09901399909146 | 0.00856531049250535 | ESR1//EREG//CEBPB//FOXC1//INHBB//DMRTA1//IMMP2L//KMT2B// |
| GO:0060688 | regulation of morphogenesis of a branching structure | Biological process | 8 | 55 | 934 | 17653 | 2.74915320225813 | 0.00796133687351356 | 0.0698144488004959 | 2.09901399909146 | 0.00856531049250535 | AR//NTN4//SULF1//ESR1//BTBD7//TNF//LHX1//NOG// |
| GO:0097345 | mitochondrial outer membrane permeabilization | Biological process | 8 | 55 | 934 | 17653 | 2.74915320225813 | 0.00796133687351356 | 0.0698144488004959 | 2.09901399909146 | 0.00856531049250535 | BCL2L11//PMAIP1//MOAP1//DYNLL2//E2F1//PPP3R1//YWHAG//BNIP3// |
| GO:0030336 | negative regulation of cell migration | Biological process | 26 | 296 | 934 | 17653 | 1.66017275305284 | 0.0079693559772265 | 0.0698144488004959 | 2.09857677356473 | 0.0278372591006424 | SLIT2//DUSP10//PFN2//PTPRG//C5ORF30//PPARG//PTEN//SPRED1//MEOX2//KLF4//WASL//LRCH1//PTGER4//CCL2//NRG1//STAT3//DLC1//SULF1//TMEFF2//CLIC4//MIA3//NF2//RAP2C//LDLRAD4//RECK//NOG// |
| GO:2000146 | negative regulation of cell motility | Biological process | 27 | 311 | 934 | 17653 | 1.64087319346998 | 0.00806580310937891 | 0.0705679513955104 | 2.09335238356246 | 0.0289079229122056 | SLIT2//DUSP10//PFN2//PTPRG//C5ORF30//DLC1//SULF1//TMEFF2//CLIC4//MIA3//NF2//PTEN//RAP2C//LDLRAD4//RECK//NOG//PPARG//SPRED1//MEOX2//KLF4//WASL//LRCH1//PTGER4//CCL2//NRG1//STAT3//CRK// |
| GO:0043406 | positive regulation of MAP kinase activity | Biological process | 24 | 268 | 934 | 17653 | 1.69257566556937 | 0.0083834807511791 | 0.0732525585532613 | 2.07657562848548 | 0.0256959314775161 | GADD45A//MINK1//TNF//MAP3K2//CRK//CRKL//MAP3K5//TAOK3//LAMTOR3//ADAM9//DUSP5//LPAR1//LPAR3//NRG1//IGF1//NTF3//PIK3CB//PRKAA1//MAPK1//MAP2K4//MDFIC//FZD10//EDN1//EPHA4// |
| GO:0055024 | regulation of cardiac muscle tissue development | Biological process | 11 | 91 | 934 | 17653 | 2.2846671529755 | 0.00846959677705885 | 0.0736974729024385 | 2.07213726519075 | 0.0117773019271949 | GJA1//JARID2//PTEN//TGFBR2//NOG//ZFPM2//GATA6//EDN1//IGF1//DLL1//NRG1// |
| GO:0003281 | ventricular septum development | Biological process | 9 | 67 | 934 | 17653 | 2.53886349835405 | 0.00847608899302905 | 0.0736974729024385 | 2.07180449214782 | 0.00963597430406852 | TGFB2//TGFBR2//NOG//ZFPM2//EGLN1//BMPR2//SOX4//SOX11//ACVR1// |
| GO:0008284 | positive regulation of cell proliferation | Biological process | 65 | 911 | 934 | 17653 | 1.34854866878057 | 0.00849299021210583 | 0.0736974729024385 | 2.07093937642517 | 0.069593147751606 | F3//JUN//KDR//BMPR2//VEGFB//VASH2//FOXF1//TGFBR2//LHX1//CDKN1A//IL2//ITGAV//CD274//NCK1//PDCD1LG2//IGF1//CDK6//E2F1//EREG//ESR1//S1PR1//EDN1//MYB//TNF//NR4A3//OSR1//NME1//NOG//ZFPM2//GATA6//WNT7A//AR//HMGA2//STAT3//MEF2D//MAP3K5//MMP2//SOX11//ADAM10//ZNF268//CRKL//E2F3//ETS1//CNOT6L//BAMBI//DLL1//CNOT7//NRG1//HOXA3//NTF3//OSM//DERL2//POU3F2//CDCA7L//PRKAA1//MAPK1//SPHK2//PTEN//PTHLH//CNOT6//RARB//SOX4//TGFB2//FOSL1//CCND2// |
| GO:0032793 | positive regulation of CREB transcription factor activity | Biological process | 4 | 16 | 934 | 17653 | 4.72510706638116 | 0.00849978247285258 | 0.0736974729024385 | 2.07059218864437 | 0.00428265524625268 | EPHA5//LRP8//RPS6KA4//RPS6KA5// |
| GO:0060413 | atrial septum morphogenesis | Biological process | 4 | 16 | 934 | 17653 | 4.72510706638116 | 0.00849978247285258 | 0.0736974729024385 | 2.07059218864437 | 0.00428265524625268 | SOX4//TGFB2//ACVR1//BMPR2// |
| GO:0072148 | epithelial cell fate commitment | Biological process | 4 | 16 | 934 | 17653 | 4.72510706638116 | 0.00849978247285258 | 0.0736974729024385 | 2.07059218864437 | 0.00428265524625268 | NEUROD1//DLL1//ACVR1//ARX// |
| GO:0002520 | immune system development | Biological process | 66 | 928 | 934 | 17653 | 1.34421149302223 | 0.00860931465985577 | 0.0745515941672274 | 2.06503141896322 | 0.0706638115631692 | ESCO2//PLEK//RBM47//REST//DLL1//NKX2-3//FNIP1//SOX4//MARCH7//TGFBR2//NME1//MKNK2//TGFB2//RUNX3//CBFB//KLF6//IFNA1//DNAJB9//IL2//THRA//JUN//PPARG//EPHA2//OSTM1//TNF//BCL11B//SOS1//ZEB1//KDR//KLF4//FLI1//FAM20C//PTGER4//KLF2//IRF1//TSC1//HMGB3//DUSP10//MYB//SOCS5//CDK6//ETS1//STAT3//NFE2//NR4A3//DPY30//KAT2B//KMT2B//CEBPB//TOB2//TMEM64//POU4F1//KLF10//BCL2L11//JARID2//TCF21//CRKL//HOXA3//MAPK1//HAND2//TMOD3//ARID4A//MFAP5//FOXC1//YTHDF2//FBXW7// |
| GO:0051099 | positive regulation of binding | Biological process | 17 | 170 | 934 | 17653 | 1.89004282655246 | 0.00877046565354777 | 0.0754569428174254 | 2.0569773478657 | 0.0182012847965739 | BAMBI//AKTIP//CAPRIN2//FOXC1//IGF1//NME1//PPARG//FMR1//MAPRE3//PPP2CA//KLF4//MET//NEUROD1//POU4F1//HMGA2//HAND2//EPHA4// |
| GO:0001764 | neuron migration | Biological process | 15 | 143 | 934 | 17653 | 1.98256240547461 | 0.00878080940371341 | 0.0754569428174254 | 2.05646544954213 | 0.0160599571734475 | ARX//DRD1//LHX6//LHX1//NRG1//STAT3//NSMF//PLAA//DCX//GJA1//GPM6A//LRP12//NR4A2//SPOCK1//UNK// |
| GO:0016202 | regulation of striated muscle tissue development | Biological process | 15 | 143 | 934 | 17653 | 1.98256240547461 | 0.00878080940371341 | 0.0754569428174254 | 2.05646544954213 | 0.0160599571734475 | GJA1//DLL1//PRKAA1//SOX17//JARID2//PTEN//TGFBR2//NOG//ZFPM2//GATA6//EDN1//IGF1//NLN//NRG1//MTPN// |
| GO:0035264 | multicellular organism growth | Biological process | 15 | 143 | 934 | 17653 | 1.98256240547461 | 0.00878080940371341 | 0.0754569428174254 | 2.05646544954213 | 0.0160599571734475 | IGF1//STAT3//ADRB1//POU3F2//KLF2//EN1//CLIC4//AR//SLC12A5//RARB//PLEKHA1//SOS1//TBL1XR1//ARID5B//PKDCC// |
| GO:0048660 | regulation of smooth muscle cell proliferation | Biological process | 15 | 143 | 934 | 17653 | 1.98256240547461 | 0.00878080940371341 | 0.0754569428174254 | 2.05646544954213 | 0.0160599571734475 | S1PR1//EDN1//EREG//IGF1//MYB//TGFBR2//TNF//NR4A3//OGN//PPARG//KLF4//JUN//MEF2D//MAP3K5//MMP2// |
| GO:0051384 | response to glucocorticoid | Biological process | 15 | 143 | 934 | 17653 | 1.98256240547461 | 0.00878080940371341 | 0.0754569428174254 | 2.05646544954213 | 0.0160599571734475 | CDKN1A//FOSL1//SLIT2//BCL2L11//EDN1//UGT1A1//REST//FIBIN//DNMT3B//GJB2//ERRFI1//CREBRF//DUSP1//TNF//ADAM9// |
| GO:0048538 | thymus development | Biological process | 7 | 45 | 934 | 17653 | 2.94006661908161 | 0.00886026739312032 | 0.0759252309483859 | 2.0525531713856 | 0.00749464668094218 | BCL2L11//CRKL//HOXA3//JARID2//MAPK1//BCL11B//HAND2// |
| GO:0033627 | cell adhesion mediated by integrin | Biological process | 8 | 56 | 934 | 17653 | 2.70006118078923 | 0.00886898306213587 | 0.0759252309483859 | 2.05212617451125 | 0.00856531049250535 | EFNA1//EPHA2//TGFB2//ADAM9//NPNT//ITGA5//ITGA6//ITGAV// |
| GO:1901185 | negative regulation of ERBB signaling pathway | Biological process | 8 | 56 | 934 | 17653 | 2.70006118078923 | 0.00886898306213587 | 0.0759252309483859 | 2.05212617451125 | 0.00856531049250535 | ERRFI1//TSG101//SOCS5//EPS15//GRB2//EPN1//EREG//NRG1// |
| GO:0060341 | regulation of cellular localization | Biological process | 59 | 815 | 934 | 17653 | 1.36825186216681 | 0.00890122618128799 | 0.0761044746714388 | 2.05055016334533 | 0.063169164882227 | TRPC3//CALM2//PKD2//CDKN1A//IGF1//MAPK1//SYBU//IPO5//MDFIC//LYPLA1//PKDCC//FOXF1//PPP1R12A//SOX4//CTDSPL2//FMR1//DRD1//PER2//EFNA5//EPHA5//RAB3GAP1//DNAJC27//MIEF1//NF2//CD2AP//BCL2L11//DYNLL2//E2F1//PMAIP1//PPP3R1//YWHAG//NEFH//NPTN//TBC1D20//MSN//APPL1//AR//PPFIA1//EPHA2//SQSTM1//PLAA//STX6//CCNE2//NRG1//FBXW7//ELMOD1//PRKAA1//SNIP1//DERL2//EDEM1//CCT6A//CENPQ//TNF//PFN2//TSG101//CHMP3//RAB21//PTEN//ARHGAP1// |
| GO:0050921 | positive regulation of chemotaxis | Biological process | 14 | 130 | 934 | 17653 | 2.03543073628727 | 0.00891463219872037 | 0.0761044746714388 | 2.04989657030818 | 0.0149892933618844 | CMKLR1//ADAM10//KDR//BMPR2//S1PR1//F3//SLIT2//NTF3//VEGFB//LPAR1//EDN1//CXCL14//MET//PPM1F// |
| GO:0042471 | ear morphogenesis | Biological process | 13 | 117 | 934 | 17653 | 2.10004758505829 | 0.00893493314936011 | 0.0761044746714388 | 2.04890869251174 | 0.0139186295503212 | LRIG1//NEUROG1//SOBP//TFAP2A//FZD6//MAPK1//TSHZ1//OSR1//EDN1//NOG//ZEB1//NR4A3//FRZB// |
| GO:0090101 | negative regulation of transmembrane receptor protein serine/threonine kinase signaling pathway | Biological process | 13 | 117 | 934 | 17653 | 2.10004758505829 | 0.00893493314936011 | 0.0761044746714388 | 2.04890869251174 | 0.0139186295503212 | PMEPA1//LDLRAD4//BAMBI//ASPN//PRDM16//RASL11B//TGFB1I1//TGFBR2//TOB1//SOSTDC1//CTDSPL2//NOG//ACVR1// |
| GO:0050927 | positive regulation of positive chemotaxis | Biological process | 5 | 25 | 934 | 17653 | 3.78008565310492 | 0.00899497639904105 | 0.0765195287883203 | 2.04599997181589 | 0.00535331905781585 | NTF3//VEGFB//S1PR1//F3//KDR// |
| GO:0009057 | macromolecule catabolic process | Biological process | 90 | 1331 | 934 | 17653 | 1.27801543493405 | 0.00915298401420937 | 0.0777658679498718 | 2.03843729613752 | 0.0963597430406852 | DCP2//PPP2CA//RPL15//ZFAND4//CNOT6L//PAN3//CNOT7//CNOT6//LYPLA1//GNS//CTSA//TOPORS//USP24//KLHL3//MYLIP//USP27X//ATXN3//RLIM//NPLOC4//USP28//USP46//UBE2I//BAG6//RNF128//KLHL15//USP45//CDC23//SQSTM1//USP3//UBE2W//TMF1//PMAIP1//UBXN4//DNAJB9//DERL2//JKAMP//WFS1//EDEM1//TGFB1I1//FBXL5//FBXW7//SKP1//BAG5//RNF180//ANKIB1//LONRF3//LONRF1//SOCS5//VLDLR//BNIP3//DNAJB12//LDLR//NRG1//MDM4//OGN//WWP2//UBXN2B//RNF38//RNF145//ZNRF2//CD2AP//SPOPL//HECW2//RNF4//TBL1XR1//PLAA//CLOCK//VPS37A//TSG101//FMR1//YTHDF2//FXR1//DDX6//RAB12//GJA1//NSF//SOX17//TNF//E2F1//SH3D19//ADAM9//TOB1//CPEB3//BTG2//QKI//NANOS1//EFNA1//EPHA4//MSN//PTEN// |
| GO:0006901 | vesicle coating | Biological process | 9 | 68 | 934 | 17653 | 2.50152727043708 | 0.00932351493165227 | 0.0787583516647793 | 2.03042032931563 | 0.00963597430406852 | TMED9//CNIH1//SEC23A//TBC1D20//GRIA1//NSF//SAR1B//PPP6C//MCFD2// |
| GO:0009247 | glycolipid biosynthetic process | Biological process | 9 | 68 | 934 | 17653 | 2.50152727043708 | 0.00932351493165227 | 0.0787583516647793 | 2.03042032931563 | 0.00963597430406852 | ST6GALNAC3//ST6GALNAC6//ST8SIA3//ST3GAL5//PIGA//B3GALT2//ST8SIA5//PRKAA1//B3GNT5// |
| GO:0014743 | regulation of muscle hypertrophy | Biological process | 9 | 68 | 934 | 17653 | 2.50152727043708 | 0.00932351493165227 | 0.0787583516647793 | 2.03042032931563 | 0.00963597430406852 | MTPN//EDN1//IGF1//TWF1//NR4A3//HAND2//JARID2//ERRFI1//TRPC3// |
| GO:0048592 | eye morphogenesis | Biological process | 15 | 144 | 934 | 17653 | 1.96879461099215 | 0.00933820088003713 | 0.0787583516647793 | 2.02973678791718 | 0.0160599571734475 | STAT3//SOX11//TFAP2A//TOPORS//FJX1//LHX1//RARB//GDF11//FOXF2//LRP6//ZEB1//DLL1//MAN2A1//TBC1D20//EPHA2// |
| GO:0000288 | nuclear-transcribed mRNA catabolic process, deadenylation-dependent decay | Biological process | 10 | 80 | 934 | 17653 | 2.36255353319058 | 0.00937955327593083 | 0.0787583516647793 | 2.02781784546083 | 0.0107066381156317 | CNOT6L//PAN3//CNOT7//CNOT6//DCP2//DDX6//TOB1//CPEB3//BTG2//NANOS1// |
| GO:0048194 | Golgi vesicle budding | Biological process | 10 | 80 | 934 | 17653 | 2.36255353319058 | 0.00937955327593083 | 0.0787583516647793 | 2.02781784546083 | 0.0107066381156317 | TMED9//CNIH1//SEC23A//TBC1D20//GRIA1//NSF//SAR1B//PPP6C//MCFD2//MIA3// |
| GO:0003230 | cardiac atrium development | Biological process | 6 | 35 | 934 | 17653 | 3.24007341694708 | 0.00938625335528791 | 0.0787583516647793 | 2.02750772741753 | 0.00642398286937901 | SOS1//SOX4//TGFB2//ACVR1//NOG//BMPR2// |
| GO:0030501 | positive regulation of bone mineralization | Biological process | 6 | 35 | 934 | 17653 | 3.24007341694708 | 0.00938625335528791 | 0.0787583516647793 | 2.02750772741753 | 0.00642398286937901 | OSR1//FAM20C//BMPR2//TFAP2A//ACVR1//PKDCC// |
| GO:0048048 | embryonic eye morphogenesis | Biological process | 6 | 35 | 934 | 17653 | 3.24007341694708 | 0.00938625335528791 | 0.0787583516647793 | 2.02750772741753 | 0.00642398286937901 | TFAP2A//FOXF2//LRP6//ZEB1//SOX11//RARB// |
| GO:1904707 | positive regulation of vascular smooth muscle cell proliferation | Biological process | 6 | 35 | 934 | 17653 | 3.24007341694708 | 0.00938625335528791 | 0.0787583516647793 | 2.02750772741753 | 0.00642398286937901 | IGF1//JUN//MEF2D//MAP3K5//MMP2//TNF// |
| GO:0048584 | positive regulation of response to stimulus | Biological process | 148 | 2334 | 934 | 17653 | 1.19848474005897 | 0.00972414119054099 | 0.0814924000887592 | 2.0121487440052 | 0.158458244111349 | GADD45A//MINK1//TNF//MAP3K2//CRK//CRKL//MAP3K5//TAOK3//LAMTOR3//ADAM9//DUSP5//LPAR1//LPAR3//NRG1//IGF1//NTF3//PIK3CB//PRKAA1//MAPK1//MAP2K4//ARL6IP1//PRKACB//SKP1//RPS6KA5//BAG6//F3//OSM//EREG//FBXW7//COPS8//MDFIC//TOB1//EPS8//ARHGAP1//ARHGAP6//PLEK//CMKLR1//ADAM10//GDF11//INHBB//BMP3//BMPR2//TGFB2//ACVR1//KDR//DENND1B//TNFRSF21//NCK1//SULF1//SOX4//ENPP4//NPNT//TGFB1I1//GATA6//SOX11//ITGA5//PKD2//RRAGD//RHEB//NR4A3//AR//ESR1//IRF1//LMO3//SYBU//GRB2//WASL//NPLOC4//IL2//STAT3//GJA1//RHOC//NDFIP2//CC2D1A//MIER1//SLC20A1//UBE2I//EDN1//EFNA1//EPHA2//EPHA4//EPHA5//EPHA7//ITGAV//MET//TGFBR2//FZD10//SPRED1//PMAIP1//ADRB1//POLR3G//MMP2//MYB//SOCS5//NPAS2//SOS1//DSTYK//NPTN//DLL1//KAT2B//RAET1E//WNT7A//RASGEF1A//ETS1//CDK19//LDLR//PTGER4//CLOCK//PLEKHA1//CBFB//PPM1F//S1PR1//SLIT2//VEGFB//SOS2//GAB1//CALM2//JUN//P2RY1//DNAJC27//PTEN//CCL2//CCL8//HAND2//ARRDC3//BCL2L11//MOAP1//BNIP3//BAMBI//LRP6//CAPRIN2//TBL1XR1//IER5//DYNLL2//E2F1//PPP3R1//YWHAG//CREBRF//PIAS4//SERINC3//TRPC3//EDEM1//ATXN3//CXCL14//HMGA2//FMR1//ST18//PIK3R3// |
| GO:0031334 | positive regulation of protein complex assembly | Biological process | 23 | 257 | 934 | 17653 | 1.6914780159808 | 0.00978800644837004 | 0.0819260985276319 | 2.00930575320556 | 0.0246252676659529 | CNOT6L//PAN3//CNOT6//CDC42EP3//GRB2//NCK1//PFN2//MET//SLAIN2//CLIP1//PMAIP1//BCL2L11//RHOC//PLEK//IQGAP2//ARFIP1//ESR1//WASL//MSN//TNF//JUN//FOSL1//FNIP1// |
| GO:1903391 | regulation of adherens junction organization | Biological process | 8 | 57 | 934 | 17653 | 2.65269168638942 | 0.0098517070401206 | 0.0823573482229118 | 2.00648851124125 | 0.00856531049250535 | EFNA5//SLK//KDR//TSC1//PPM1F//DLC1//ARHGAP6//PTEN// |
| GO:0016197 | endosomal transport | Biological process | 19 | 200 | 934 | 17653 | 1.79554068522484 | 0.0098848433489003 | 0.0825323402081639 | 2.00503020882348 | 0.0203426124197002 | STX6//DENND1B//EPS15//VPS37A//SNX2//PIKFYVE//TMEM87A//SNX5//RAB9B//EVI5//ZFYVE21//TBC1D17//CHMP3//ANKRD50//ARHGAP1//TSG101//DPY30//SQSTM1//ZFYVE9// |
| GO:0034613 | cellular protein localization | Biological process | 115 | 1762 | 934 | 17653 | 1.23356938168861 | 0.00991140954099519 | 0.0825469695732912 | 2.00386457837592 | 0.123126338329764 | RANBP6//IPO5//TNF//DRD1//STAT3//BCL2L11//PMAIP1//MOAP1//TWF1//ZFAND6//NUP35//AGFG1//NUP43//SRSF2//TSC1//PAN3//YWHAG//EGR2//ZDHHC1//ARL6IP1//RPL15//MTX3//IMMP2L//STX6//SEC23A//RAB12//STX2//RAB21//TBC1D12//RAB38//SNX5//ARFIP1//RAB30//NSF//SAR1B//RAB9B//DNAJC27//RAB23//TMED9//CTSA//RAB22A//KIF13A//SNX2//EVI5//TBC1D17//AP1S2//RAB28//ZFYVE9//USP6NL//GNPTG//PEX5L//DERL2//NPLOC4//TRAPPC8//CDKN1A//IGF1//MAPK1//MZT1//ATG16L1//MTBP//TOPORS//PIKFYVE//CNEP1R1//WASL//MDFIC//LYPLA1//PKDCC//GOLGA4//C16ORF70//CLIP1//SOX4//CTDSPL2//MORC3//SKP1//RNF128//ESCO2//ESR1//RAD21//NEDD1//BAG6//FCHO2//EFR3A//LRP6//P2RY1//MIEF1//RAB3GAP1//GLRB//SNAP25//NF2//CD2AP//DYNLL2//E2F1//PPP3R1//MSN//APPL1//AR//PPFIA1//EPHA2//SQSTM1//ARL5A//CCNE2//NPTN//NRG1//FBXW7//ELMOD1//PRKAA1//SNIP1//EDEM1//CCT6A//CENPQ//TSG101//FRMD6//LATS2//RRAGD//LAMTOR3// |
| GO:0030902 | hindbrain development | Biological process | 15 | 145 | 934 | 17653 | 1.95521671712324 | 0.00992321251856953 | 0.0825469695732912 | 2.00334770756081 | 0.0160599571734475 | NOG//POU4F1//LPAR1//EN1//FOXC1//LHX1//NEUROD1//DLC1//LRP6//EGR2//DLL1//MTPN//WNT7A//NANOS1//NFIB// |
| GO:1901861 | regulation of muscle tissue development | Biological process | 15 | 145 | 934 | 17653 | 1.95521671712324 | 0.00992321251856953 | 0.0825469695732912 | 2.00334770756081 | 0.0160599571734475 | MTPN//GJA1//DLL1//PRKAA1//SOX17//JARID2//PTEN//TGFBR2//NOG//ZFPM2//GATA6//EDN1//IGF1//NLN//NRG1// |
| GO:0002067 | glandular epithelial cell differentiation | Biological process | 7 | 46 | 934 | 17653 | 2.87615212736244 | 0.00998566580069527 | 0.0829644444841549 | 2.00062297296373 | 0.00749464668094218 | RARB//GATA6//CDK6//DLL1//NEUROD1//RHEB//CLOCK// |
| GO:0001952 | regulation of cell-matrix adhesion | Biological process | 12 | 106 | 934 | 17653 | 2.13967112439901 | 0.010186770864765 | 0.0844006855542831 | 1.99196346249654 | 0.012847965738758 | NF2//CDK6//EFNA5//SLK//KDR//TSC1//PPM1F//DLC1//ARHGAP6//PTEN//MINK1//PIK3CB// |
| GO:0060415 | muscle tissue morphogenesis | Biological process | 10 | 81 | 934 | 17653 | 2.33338620562032 | 0.01020863046029 | 0.0844006855542831 | 1.99103251681942 | 0.0107066381156317 | ZFPM2//NRG1//TGFB2//POU4F1//NOG//S1PR1//EGLN1//ACTC1//FOXC1//HAND1// |
| GO:0002088 | lens development in camera-type eye | Biological process | 9 | 69 | 934 | 17653 | 2.46527325202495 | 0.0102334115266172 | 0.0844006855542831 | 1.98997956079473 | 0.00963597430406852 | SOX11//MAF//NF2//TMOD1//WNT7A//TBC1D20//EPHA2//GJA1//TGFBR2// |
| GO:0043242 | negative regulation of protein complex disassembly | Biological process | 9 | 69 | 934 | 17653 | 2.46527325202495 | 0.0102334115266172 | 0.0844006855542831 | 1.98997956079473 | 0.00963597430406852 | EPS8//TWF1//STMN2//MID1IP1//LIMA1//TMOD3//TMOD1//MTPN//TNF// |
| GO:0048708 | astrocyte differentiation | Biological process | 9 | 69 | 934 | 17653 | 2.46527325202495 | 0.0102334115266172 | 0.0844006855542831 | 1.98997956079473 | 0.00963597430406852 | CDK6//DRD1//DLL1//POU3F2//EPHA4//NOG//MAPK1//LDLR//STAT3// |
| GO:0055021 | regulation of cardiac muscle tissue growth | Biological process | 9 | 69 | 934 | 17653 | 2.46527325202495 | 0.0102334115266172 | 0.0844006855542831 | 1.98997956079473 | 0.00963597430406852 | GJA1//JARID2//PTEN//TGFBR2//NOG//ZFPM2//GATA6//EDN1//IGF1// |
| GO:0032869 | cellular response to insulin stimulus | Biological process | 19 | 201 | 934 | 17653 | 1.78660764698989 | 0.0103942851583396 | 0.0856230822482956 | 1.98320537285138 | 0.0203426124197002 | GAB1//APPL1//GRB2//SOS1//PIK3R3//SLC2A4//SESN3//NCK1//TSC1//IGF1//PRKAA1//CPEB2//INHBB//ERRFI1//PPARG//PTEN//CPEB1//YWHAG//KAT2B// |
| GO:0006367 | transcription initiation from RNA polymerase II promoter | Biological process | 18 | 187 | 934 | 17653 | 1.81929256031788 | 0.0104131102694029 | 0.0856738014014256 | 1.98241953263268 | 0.019271948608137 | GTF2E1//THRA//ESR1//CDK8//CDKN1A//E2F3//NR5A2//NRBF2//AR//NR3C2//NR4A2//PPARG//RARB//TEAD1//NR4A3//CBFB//KAT2B//CCNC// |
| GO:0048659 | smooth muscle cell proliferation | Biological process | 15 | 146 | 934 | 17653 | 1.94182482180048 | 0.0105367553440888 | 0.0864563328589509 | 1.97729310383867 | 0.0160599571734475 | NR4A3//S1PR1//EDN1//EREG//IGF1//MYB//TGFBR2//TNF//OGN//PPARG//KLF4//JUN//MEF2D//MAP3K5//MMP2// |
| GO:0021756 | striatum development | Biological process | 4 | 17 | 934 | 17653 | 4.44715959188815 | 0.0106571363466692 | 0.0864563328589509 | 1.97235947785319 | 0.00428265524625268 | BCL11B//DRD1//HPRT1//RARB// |
| GO:0032693 | negative regulation of interleukin-10 production | Biological process | 4 | 17 | 934 | 17653 | 4.44715959188815 | 0.0106571363466692 | 0.0864563328589509 | 1.97235947785319 | 0.00428265524625268 | TNFRSF21//DLL1//CD274//PDCD1LG2// |
| GO:0044320 | cellular response to leptin stimulus | Biological process | 4 | 17 | 934 | 17653 | 4.44715959188815 | 0.0106571363466692 | 0.0864563328589509 | 1.97235947785319 | 0.00428265524625268 | STAT3//INHBB//PTEN//NR4A3// |
| GO:2000269 | regulation of fibroblast apoptotic process | Biological process | 4 | 17 | 934 | 17653 | 4.44715959188815 | 0.0106571363466692 | 0.0864563328589509 | 1.97235947785319 | 0.00428265524625268 | BCL2L11//BTG1//STK17B//IER3IP1// |
| GO:2000641 | regulation of early endosome to late endosome transport | Biological process | 4 | 17 | 934 | 17653 | 4.44715959188815 | 0.0106571363466692 | 0.0864563328589509 | 1.97235947785319 | 0.00428265524625268 | RAB21//MSN//CHMP3//MAPK1// |
| GO:0001964 | startle response | Biological process | 5 | 26 | 934 | 17653 | 3.63469774337012 | 0.0106616267343435 | 0.0864563328589509 | 1.97217652628044 | 0.00535331905781585 | DRD1//PTEN//GLRA1//GLRB//NRG1// |
| GO:0048596 | embryonic camera-type eye morphogenesis | Biological process | 5 | 26 | 934 | 17653 | 3.63469774337012 | 0.0106616267343435 | 0.0864563328589509 | 1.97217652628044 | 0.00535331905781585 | TFAP2A//SOX11//FOXF2//LRP6//ZEB1// |
| GO:0050926 | regulation of positive chemotaxis | Biological process | 5 | 26 | 934 | 17653 | 3.63469774337012 | 0.0106616267343435 | 0.0864563328589509 | 1.97217652628044 | 0.00535331905781585 | S1PR1//F3//KDR//NTF3//VEGFB// |
| GO:0055022 | negative regulation of cardiac muscle tissue growth | Biological process | 5 | 26 | 934 | 17653 | 3.63469774337012 | 0.0106616267343435 | 0.0864563328589509 | 1.97217652628044 | 0.00535331905781585 | GJA1//JARID2//PTEN//TGFBR2//NOG// |
| GO:0060571 | morphogenesis of an epithelial fold | Biological process | 5 | 26 | 934 | 17653 | 3.63469774337012 | 0.0106616267343435 | 0.0864563328589509 | 1.97217652628044 | 0.00535331905781585 | RDH10//NOG//AR//SOSTDC1//SULF1// |
| GO:0061117 | negative regulation of heart growth | Biological process | 5 | 26 | 934 | 17653 | 3.63469774337012 | 0.0106616267343435 | 0.0864563328589509 | 1.97217652628044 | 0.00535331905781585 | GJA1//JARID2//PTEN//TGFBR2//NOG// |
| GO:2000147 | positive regulation of cell motility | Biological process | 39 | 502 | 934 | 17653 | 1.46835996485151 | 0.0106879935661005 | 0.0865204771159267 | 1.97110381630265 | 0.0417558886509636 | MIA3//CCL8//EDN1//ETS1//KDR//BMPR2//WNT7A//JUN//TGFB2//TGFBR2//DOCK5//PPM1F//CMKLR1//ADAM10//CRK//IGF1//SEMA4B//ZNF268//DRD1//S1PR1//F3//FOXF1//CD274//ITGA6//ITGA5//ITGAV//NTF3//MAPK1//PTP4A1//ACVR1//GAB1//ADAM9//VEGFB//LPAR1//TNF//CXCL14//MET//NSMF//PLAA// |
| GO:0044772 | mitotic cell cycle phase transition | Biological process | 43 | 565 | 934 | 17653 | 1.43843967330541 | 0.0106951232986714 | 0.0865204771159267 | 1.97081420430199 | 0.0460385438972163 | CDK6//CDKN1A//LATS2//PPP6C//RBBP8//ACVR1//CCNE2//E2F1//NPAT//RRM2//NEDD1//CCNY//PPP1R12A//PRKAR2B//SKP1//YWHAG//MASTL//HAUS8//CEP41//GADD45A//CNOT6L//CNOT7//MDM4//CNOT6//RBL2//SOX4//BTG2//MTBP//CDC23//TAOK3//HMGA2//CDC14A//EPS8//RAD21//HECW2//NEUROG1//PTEN//PKD2//DUSP1//CCND2//TMOD3//ZNF655//KLF4// |
| GO:0009953 | dorsal/ventral pattern formation | Biological process | 11 | 94 | 934 | 17653 | 2.21175224383799 | 0.0107172288673519 | 0.0865957214216259 | 1.96991749479213 | 0.0117773019271949 | LRP6//AIDA//PRKACB//SOSTDC1//ACVR1//EDN1//EN1//LHX1//DSCAML1//WNT7A//NOG// |
| GO:0002931 | response to ischemia | Biological process | 6 | 36 | 934 | 17653 | 3.15007137758744 | 0.0107715076679438 | 0.0866203404973887 | 1.96772350505459 | 0.00642398286937901 | GJA1//GJB2//MAP3K5//CPEB4//PER2//SQSTM1// |
| GO:0007618 | mating | Biological process | 6 | 36 | 934 | 17653 | 3.15007137758744 | 0.0107715076679438 | 0.0866203404973887 | 1.96772350505459 | 0.00642398286937901 | DRD1//NHLH2//THRA//PTEN//DMRTA1//P2RY1// |
| GO:0050919 | negative chemotaxis | Biological process | 6 | 36 | 934 | 17653 | 3.15007137758744 | 0.0107715076679438 | 0.0866203404973887 | 1.96772350505459 | 0.00642398286937901 | SLIT2//NRG1//SEMA4B//EFNA5//EPHA7//ITGAV// |
| GO:0060428 | lung epithelium development | Biological process | 6 | 36 | 934 | 17653 | 3.15007137758744 | 0.0107715076679438 | 0.0866203404973887 | 1.96772350505459 | 0.00642398286937901 | GATA6//NFIB//PPP3R1//KLF2//THRA//ERRFI1// |
| GO:0046661 | male sex differentiation | Biological process | 16 | 160 | 934 | 17653 | 1.89004282655246 | 0.0107917313507524 | 0.0866293693818955 | 1.96690887452389 | 0.0171306209850107 | BCL2L11//RNF38//ESR1//GATA6//TFAP2C//TGFB2//ARID5B//AR//PLEKHA1//TMF1//LHFPL2//TCF21//TBC1D20//ARID4B//ARID4A//ZFPM2// |
| GO:0048565 | digestive tract development | Biological process | 14 | 133 | 934 | 17653 | 1.98951876479207 | 0.0107982490594319 | 0.0866293693818955 | 1.96664665983481 | 0.0149892933618844 | FOXF1//SOX11//TCF21//FOXF2//RARB//TGFB2//TNF//PKDCC//SOX17//NKX2-3//CDKN1A//GATA6//ITGA6//TGFBR2// |
| GO:0010811 | positive regulation of cell-substrate adhesion | Biological process | 12 | 107 | 934 | 17653 | 2.11967419800276 | 0.010939042378222 | 0.0875113773856583 | 1.96102069519246 | 0.012847965738758 | CDK6//KDR//TSC1//PPM1F//CRK//CRKL//DOCK5//FOXF1//NPNT//ITGA6//ITGA5//SMOC2// |
| GO:0044773 | mitotic DNA damage checkpoint | Biological process | 12 | 107 | 934 | 17653 | 2.11967419800276 | 0.010939042378222 | 0.0875113773856583 | 1.96102069519246 | 0.012847965738758 | CDKN1A//GADD45A//E2F1//CNOT6L//CNOT7//MDM4//CNOT6//RBL2//SOX4//BTG2//TAOK3//HMGA2// |
| GO:0045727 | positive regulation of translation | Biological process | 13 | 120 | 934 | 17653 | 2.04754639543183 | 0.0109470095029228 | 0.0875113773856583 | 1.96070450491147 | 0.0139186295503212 | NCK1//TNF//PPARG//YTHDF2//ESR1//FAM129A//CPEB3//FMR1//SERP1//PRR16//MAPK1//SOX4//FXR1// |
| GO:0045445 | myoblast differentiation | Biological process | 10 | 82 | 934 | 17653 | 2.30493027628349 | 0.0110922968947234 | 0.0885681273896273 | 1.95497851467888 | 0.0107066381156317 | SOSTDC1//DLL1//TNF//CXCL14//MAP3K5//BTG1//NRG1//REST//IGF1//MBNL1// |
| GO:0043254 | regulation of protein complex assembly | Biological process | 35 | 441 | 934 | 17653 | 1.50003398932735 | 0.0111255723184492 | 0.0887290631953679 | 1.9536776389222 | 0.0374732334047109 | EPS8//TWF1//CNOT6L//PAN3//CNOT6//PMEPA1//LDLRAD4//THRA//PFN2//SLIT2//CDC42EP3//GRB2//NCK1//STMN2//MET//SLAIN2//CLIP1//TNF//FNIP1//PMAIP1//BCL2L11//RHOC//PTGER4//PLEK//IQGAP2//ARFIP1//ESR1//WASL//TMOD3//TMOD1//MSN//RNF4//JUN//FOSL1//MTPN// |
| GO:0045165 | cell fate commitment | Biological process | 23 | 260 | 934 | 17653 | 1.67196096195026 | 0.0111535200154369 | 0.0887880101628839 | 1.95258804902614 | 0.0246252676659529 | DLL1//DSCAML1//LATS2//NEUROD1//GATA6//SOX17//KLF4//SOSTDC1//MNX1//NRG1//BCL11B//WNT7A//MYT1L//POU3F2//POU4F1//AR//CEBPB//PPARG//ACVR1//ARX//STAT3//NEUROG1//TGFB1I1// |
| GO:1903362 | regulation of cellular protein catabolic process | Biological process | 21 | 231 | 934 | 17653 | 1.71822075141133 | 0.0111619114613668 | 0.0887880101628839 | 1.95226142672102 | 0.0224839400428266 | TMF1//BAG6//BAG5//RNF180//ANKIB1//LONRF3//LONRF1//SOCS5//MYLIP//FMR1//FBXW7//EFNA1//EPHA4//FBXL5//MSN//DERL2//EDEM1//ATXN3//RNF128//LDLR//PTEN// |
| GO:0045184 | establishment of protein localization | Biological process | 129 | 2012 | 934 | 17653 | 1.21180678243175 | 0.0111989014510063 | 0.0887880101628839 | 1.95082457707673 | 0.138115631691649 | RANBP6//IPO5//TNF//DRD1//STAT3//BCL2L11//PMAIP1//MOAP1//ZFAND6//NUP35//AGFG1//NUP43//SRSF2//TSC1//PAN3//YWHAG//EGR2//ZDHHC1//ARL6IP1//RPL15//MTX3//IMMP2L//STX6//SEC23A//RAB12//STX2//RAB21//TBC1D12//RAB38//SNX5//ARFIP1//RAB30//NSF//SAR1B//RAB9B//DNAJC27//RAB23//TMED9//CTSA//RAB22A//KIF13A//SNX2//EVI5//TBC1D17//AP1S2//RAB28//ZFYVE9//USP6NL//RAPGEF4//MARCKS//NEUROD1//SNAP25//PER2//CLOCK//CBLN4//MIA3//TRIM3//EXOC5//VPS37A//DENND1B//LCA5//EPS15//NECAP1//SERP1//CHMP3//ATG16L1//UEVLD//ANKRD50//ZMAT3//AKTIP//BTF3//TSG101//PLEKHF2//FAM160A2//C5ORF30//MCFD2//PKDCC//ARRDC4//CEP41//GNPTG//PEX5L//DERL2//NPLOC4//GJA1//SOX4//CDKN1A//IGF1//MAPK1//ARHGAP1//VLDLR//SYBU//MCOLN2//ITGAV//MDFIC//PPARG//LYPLA1//GOLGA4//C16ORF70//CLIP1//INHBB//REST//CTDSPL2//PTGER4//TGFB2//ADAM9//IL2//WWP2//NDFIP2//EFNA5//EPHA5//PLEK//BAG6//MIEF1//RAB3GAP1//DYNLL2//E2F1//PPP3R1//NRG1//FBXW7//ELMOD1//PRKAA1//SNIP1//EDEM1//CCT6A//TNFRSF21//CD274//DZIP1//NPNT//PLEKHA1// |
| GO:0043507 | positive regulation of JUN kinase activity | Biological process | 9 | 70 | 934 | 17653 | 2.43005506271031 | 0.0112084966905804 | 0.0887880101628839 | 1.95045263207643 | 0.00963597430406852 | MAP3K2//MDFIC//MAP3K5//MAP2K4//FZD10//EDN1//EPHA4//TAOK3//TNF// |
| GO:0097061 | dendritic spine organization | Biological process | 9 | 70 | 934 | 17653 | 2.43005506271031 | 0.0112084966905804 | 0.0887880101628839 | 1.95045263207643 | 0.00963597430406852 | CTNND2//PTEN//WNT7A//WASL//EPHA4//EFNA1//CAPRIN2//LRP8//ZNF804A// |
| GO:0034260 | negative regulation of GTPase activity | Biological process | 7 | 47 | 934 | 17653 | 2.81495740124835 | 0.0112117345377943 | 0.0887880101628839 | 1.95032719365666 | 0.00749464668094218 | IQGAP2//FZD10//CPEB2//LRCH1//IPO5//TSC1//SLIT2// |
| GO:0007166 | cell surface receptor signaling pathway | Biological process | 186 | 3016 | 934 | 17653 | 1.16560996597731 | 0.011281343920583 | 0.0892347706840969 | 1.94763916076694 | 0.199143468950749 | CBFB//P2RY1//PRKACB//SKP1//RPS6KA5//BAG6//SOX17//APCDD1//CTNND2//AMER2//CCNY//SOSTDC1//HBP1//DKK3//LRP6//PIAS4//PRKAA1//STRN//TGFB1I1//WNT7A//TCF7L1//PPP3R1//FZD6//GRB2//JAK1//KDR//NTF3//PIK3CB//PTPRG//PALM2//PPM1L//BMPR2//EREG//FBXW7//ERRFI1//TSG101//SOCS5//EFNA5//NRG1//EPS8//EPS15//GAB1//SOS1//CAMLG//COL1A2//JUN//TGFB2//TGFBR2//KLF10//ADAM9//ACVR1//ZFYVE9//FMR1//GNAQ//ADAM10//FOXC1//DLL1//EPN1//TMEM100//KAT2B//TMEM17//DZIP1//FOXF1//ITGA6//ITGA5//ITGAV//ITGB8//PLEK//APPL1//PIK3R3//SPRED1//FLRT1//ESRP1//MAPK1//OTX2//MOAP1//TNF//F3//GDF11//INHBB//BMP3//PMEPA1//LDLRAD4//SOX11//ZEB1//CDKN1A//CRK//CRKL//S1PR1//IFNA1//IL2//MMP2//OSM//ASPN//CCL2//STAT3//STAT4//LRP8//DENND1B//TNFRSF21//NCK1//ESR1//MDFIC//PPP2CA//SULF1//SOX4//FRZB//SHISA2//TOB1//RGMB//RGMA//NOG//NPNT//BAMBI//PRDM16//RASL11B//GATA6//CTDSPL2//ST18//ACSL1//ADIPOR2//GRIA1//CPEB4//SLIT2//FZD10//GSTA2//MSN//RAP1B//VLDLR//MAP2K4//WASL//NR4A3//BRWD1//FAM20C//IGF1//AR//TMEM64//DSTYK//NPTN//WWP2//YTHDF2//SESN3//TSC1//PTEN//PLEKHA1//ARID5B//VEGFB//MET//EFNA1//EPHA2//EPHA4//EPHA5//EPHA7//ARHGEF28//NR4A2//KLF4//VANGL1//RAB3GAP1//GLRA1//GLRB//SLC17A7//IRF9//IRF1//PPARG//CNOT7//CMKLR1//CCL8//EDN1//SQSTM1//RPS6KA4//SEMA4B//LATS2//CAPRIN2//TBL1XR1//SGPP1//BCL2L11//MKNK2//ITM2B//PMAIP1//CREBRF//MINK1//PHF14//HAND2//ZMYND11//CD274//PDCD1LG2// |
| GO:0051495 | positive regulation of cytoskeleton organization | Biological process | 20 | 217 | 934 | 17653 | 1.74197495534789 | 0.0113449035183788 | 0.089632689830369 | 1.94519919317824 | 0.0214132762312634 | CFL2//PLEK//CDC42EP3//GRB2//NCK1//PFN2//MET//SLAIN2//CLIP1//STMN2//IQGAP2//ARFIP1//WASL//LPAR1//PPM1E//NF2//TSC1//PPM1F//EDN1//NTF3// |
| GO:0045862 | positive regulation of proteolysis | Biological process | 29 | 350 | 934 | 17653 | 1.56603548485775 | 0.0114625363419794 | 0.0904542040211729 | 1.94071927438878 | 0.0310492505353319 | DLC1//EGLN3//F3//PMAIP1//PPARG//TNF//PCOLCE2//RNF180//ANKIB1//LONRF3//LONRF1//SOCS5//BCL2L11//MAP3K5//REST//PPM1F//SH3D19//ADAM9//ASPH//FMR1//FBXW7//EFNA1//EPHA4//EDEM1//ATXN3//BAG6//STAT3//PTEN//ST18// |
| GO:0045665 | negative regulation of neuron differentiation | Biological process | 19 | 203 | 934 | 17653 | 1.76900560120674 | 0.0114760615591116 | 0.0904542040211729 | 1.94020713080677 | 0.0203426124197002 | STMN2//LPAR1//EPHA4//MYLIP//PTPRG//SPOCK1//TSC1//BAG5//DLL1//EPHA7//ULK2//RGMA//PTEN//SEMA4B//EFNA1//SLIT2//HMG20A//DDX6//REST// |
| GO:0050804 | modulation of chemical synaptic transmission | Biological process | 28 | 335 | 934 | 17653 | 1.57973728786474 | 0.0115023828860282 | 0.0904542040211729 | 1.93921217990282 | 0.0299785867237687 | SNAP25//PTEN//USP46//SLC6A1//EDN1//FMR1//CPEB3//YWHAG//EGR2//NSMF//KCNJ10//NPTN//RAB3GAP1//DRD1//TSHZ3//SYBU//SLC24A2//MAPK1//GRIA1//FXR1//PAIP2//EPHA4//SQSTM1//PFN2//WNT7A//NTF3//PXK//LRP8// |
| GO:0099177 | regulation of trans-synaptic signaling | Biological process | 28 | 335 | 934 | 17653 | 1.57973728786474 | 0.0115023828860282 | 0.0904542040211729 | 1.93921217990282 | 0.0299785867237687 | SNAP25//PTEN//USP46//SLC6A1//EDN1//FMR1//CPEB3//YWHAG//EGR2//NSMF//KCNJ10//NPTN//RAB3GAP1//GRIA1//NTF3//PXK//LRP8//DRD1//TSHZ3//SYBU//SLC24A2//MAPK1//FXR1//PAIP2//EPHA4//SQSTM1//PFN2//WNT7A// |
| GO:2000045 | regulation of G1/S transition of mitotic cell cycle | Biological process | 17 | 175 | 934 | 17653 | 1.83604160293668 | 0.011527960132231 | 0.09055005153807 | 1.9382475341356 | 0.0182012847965739 | CDKN1A//GADD45A//E2F1//CNOT6L//CNOT7//MDM4//CNOT6//RBL2//SOX4//BTG2//MTBP//CDK6//PTEN//PKD2//CCND2//ZNF655//KLF4// |
| GO:0006892 | post-Golgi vesicle-mediated transport | Biological process | 11 | 95 | 934 | 17653 | 2.18847064127127 | 0.0115598240144126 | 0.0906949997789703 | 1.93704877752027 | 0.0117773019271949 | EXOC5//OSBPL5//STEAP2//EPS15//LYPLA1//PKDCC//GOLGA4//NSF//KIF13A//C16ORF70//MYO1B// |
| GO:0070727 | cellular macromolecule localization | Biological process | 115 | 1772 | 934 | 17653 | 1.22660792919601 | 0.0116558750105667 | 0.0913426218962487 | 1.9334551182827 | 0.123126338329764 | RANBP6//IPO5//TNF//DRD1//STAT3//BCL2L11//PMAIP1//MOAP1//TWF1//ZFAND6//NUP35//AGFG1//NUP43//SRSF2//TSC1//PAN3//YWHAG//EGR2//ZDHHC1//ARL6IP1//RPL15//MTX3//IMMP2L//STX6//SEC23A//RAB12//STX2//RAB21//TBC1D12//RAB38//SNX5//ARFIP1//RAB30//NSF//SAR1B//RAB9B//DNAJC27//RAB23//TMED9//CTSA//RAB22A//KIF13A//SNX2//EVI5//TBC1D17//AP1S2//RAB28//ZFYVE9//USP6NL//GNPTG//PEX5L//DERL2//NPLOC4//TRAPPC8//CDKN1A//IGF1//MAPK1//MZT1//ATG16L1//MTBP//TOPORS//PIKFYVE//CNEP1R1//FRMD6//LATS2//RRAGD//LAMTOR3//WASL//MDFIC//LYPLA1//PKDCC//GOLGA4//C16ORF70//CLIP1//SOX4//CTDSPL2//MORC3//SKP1//RNF128//ESCO2//ESR1//RAD21//NEDD1//BAG6//FCHO2//EFR3A//LRP6//P2RY1//MIEF1//RAB3GAP1//GLRB//SNAP25//NF2//CD2AP//DYNLL2//E2F1//PPP3R1//MSN//APPL1//AR//PPFIA1//EPHA2//SQSTM1//ARL5A//CCNE2//NPTN//NRG1//FBXW7//ELMOD1//PRKAA1//SNIP1//EDEM1//CCT6A//CENPQ//TSG101// |
| GO:0000082 | G1/S transition of mitotic cell cycle | Biological process | 24 | 276 | 934 | 17653 | 1.64351550134997 | 0.0117914589182676 | 0.0922981905836155 | 1.92843245775861 | 0.0256959314775161 | E2F1//NPAT//RRM2//CDKN1A//GADD45A//CNOT6L//CNOT7//MDM4//CNOT6//RBL2//SOX4//BTG2//MTBP//CDK6//PTEN//PKD2//CCND2//ZNF655//KLF4//LATS2//PPP6C//RBBP8//ACVR1//CCNE2// |
| GO:0060560 | developmental growth involved in morphogenesis | Biological process | 20 | 218 | 934 | 17653 | 1.73398424454354 | 0.0118920551188139 | 0.092977998576345 | 1.92474308653898 | 0.0214132762312634 | S1PR1//TGFBR2//DPYSL2//RAB21//EPHA4//LHX1//GOLGA4//NRG1//EFNA5//EPHA7//ULK2//LPAR3//BMPR2//SEMA4B//SLIT2//RDH10//ESR1//LRP6//PLAA//FLRT1// |
| GO:0010660 | regulation of muscle cell apoptotic process | Biological process | 10 | 83 | 934 | 17653 | 2.27716003199092 | 0.01203271038815 | 0.0938606924510478 | 1.91963653608203 | 0.0107066381156317 | MAP3K5//PTEN//BNIP3//HAND2//EDN1//IGF1//LRP6//MAP2K4//E2F3//PPARG// |
| GO:0048814 | regulation of dendrite morphogenesis | Biological process | 10 | 83 | 934 | 17653 | 2.27716003199092 | 0.01203271038815 | 0.0938606924510478 | 1.91963653608203 | 0.0107066381156317 | EPHA4//RAB21//CAPRIN2//EFNA1//PTEN//LRP8//FMR1//NSMF//HECW2//FXR1// |
| GO:0070059 | intrinsic apoptotic signaling pathway in response to endoplasmic reticulum stress | Biological process | 8 | 59 | 934 | 17653 | 2.56276993430842 | 0.0120565555712564 | 0.0939383471525427 | 1.91877674775616 | 0.00856531049250535 | WFS1//BCL2L11//SERINC3//NCK1//PMAIP1//CEBPB//MAP3K5//BAG6// |
| GO:0090114 | COPII-coated vesicle budding | Biological process | 9 | 71 | 934 | 17653 | 2.3958289350665 | 0.0122514834155612 | 0.0952376808499315 | 1.91181132352895 | 0.00963597430406852 | CNIH1//SEC23A//TBC1D20//GRIA1//NSF//SAR1B//PPP6C//MCFD2//MIA3// |
| GO:0098739 | import across plasma membrane | Biological process | 9 | 71 | 934 | 17653 | 2.3958289350665 | 0.0122514834155612 | 0.0952376808499315 | 1.91181132352895 | 0.00963597430406852 | ARL6IP1//KCNJ10//PER2//TRPV6//FAM155A//STEAP2//ARHGAP1//HCN2//KCNJ3// |
| GO:0016572 | histone phosphorylation | Biological process | 6 | 37 | 934 | 17653 | 3.06493431332832 | 0.0122954505091893 | 0.0952510100729063 | 1.91025555394678 | 0.00642398286937901 | FMR1//RPS6KA4//RPS6KA5//PRKAA1//HMGA2//DRD1// |
| GO:0019068 | virion assembly | Biological process | 6 | 37 | 934 | 17653 | 3.06493431332832 | 0.0122954505091893 | 0.0952510100729063 | 1.91025555394678 | 0.00642398286937901 | DDX6//VPS37A//CHMP3//TSG101//TBC1D20//USP6NL// |
| GO:0055026 | negative regulation of cardiac muscle tissue development | Biological process | 6 | 37 | 934 | 17653 | 3.06493431332832 | 0.0122954505091893 | 0.0952510100729063 | 1.91025555394678 | 0.00642398286937901 | GJA1//JARID2//PTEN//TGFBR2//NOG//DLL1// |
| GO:1900101 | regulation of endoplasmic reticulum unfolded protein response | Biological process | 5 | 27 | 934 | 17653 | 3.50007930843049 | 0.0125274960097084 | 0.0967272886576088 | 1.90213572691811 | 0.00535331905781585 | WFS1//DNAJB9//BCL2L11//NCK1//PPP1R15B// |
| GO:0010463 | mesenchymal cell proliferation | Biological process | 7 | 48 | 934 | 17653 | 2.75631245538901 | 0.0125432252185011 | 0.0967272886576088 | 1.9015907797331 | 0.00749464668094218 | FOXF1//TGFBR2//ZEB1//OSR1//NFIB//PHF14//HAND2// |
| GO:0055010 | ventricular cardiac muscle tissue morphogenesis | Biological process | 7 | 48 | 934 | 17653 | 2.75631245538901 | 0.0125432252185011 | 0.0967272886576088 | 1.9015907797331 | 0.00749464668094218 | ZFPM2//NRG1//TGFB2//POU4F1//NOG//FOXC1//HAND1// |
| GO:0060042 | retina morphogenesis in camera-type eye | Biological process | 7 | 48 | 934 | 17653 | 2.75631245538901 | 0.0125432252185011 | 0.0967272886576088 | 1.9015907797331 | 0.00749464668094218 | TOPORS//FJX1//LHX1//TFAP2A//LRP6//DLL1//MAN2A1// |
| GO:0098732 | macromolecule deacylation | Biological process | 12 | 109 | 934 | 17653 | 2.08078109345225 | 0.0125714687263404 | 0.0968346731164466 | 1.90061398066862 | 0.012847965738758 | LYPLA1//ARID4B//MIER1//ARID4A//RBBP7//TBL1XR1//BRMS1L//PER2//REST//SPRED1//PPT2//FRY// |
| GO:0097305 | response to alcohol | Biological process | 18 | 191 | 934 | 17653 | 1.78119219256253 | 0.0127664385151825 | 0.0982246003164724 | 1.89393024204211 | 0.019271948608137 | IL2//RPL15//SLC2A4//STAT3//ACTC1//EPS8//USP46//TGFBR2//GLRA1//UGT1A1//PRKAA1//PTEN//PPARG//PTGER4//LRP6//LRP8//KLF2//KLF4// |
| GO:0007612 | learning | Biological process | 14 | 136 | 934 | 17653 | 1.94563232145106 | 0.01297948270878 | 0.0997502744994081 | 1.88674261579444 | 0.0149892933618844 | DRD1//SNAP25//BTG2//ADCY3//NPTN//EN1//CIC//SLC24A2//JUN//PRKAR2B//SLC12A5//ATXN1//SLC6A1//FOSL1// |
| GO:0034097 | response to cytokine | Biological process | 79 | 1164 | 934 | 17653 | 1.28276102489385 | 0.0130203737906056 | 0.099950951130381 | 1.88537654780568 | 0.0845824411134904 | CBFB//TNF//PIAS4//CDKN1A//COL1A2//CRK//CRKL//S1PR1//EREG//F3//FLRT1//GRB2//IFNA1//IL2//JAK1//MMP2//OSM//PIK3CB//ASPN//CCL2//SOS1//STAT3//STAT4//ZEB1//LRP8//SOCS5//TNFRSF21//ST18//ACSL1//ADIPOR2//CALCOCO2//ADAM10//ADAM9//IRF1//GSTA2//MSN//RAP1B//BRWD1//IRF9//PPARG//CNOT7//KLF4//CMKLR1//FOXC1//CCL8//SLIT2//EDN1//SKP1//SQSTM1//RPS6KA4//RPS6KA5//ETS1//FOXF1//POU4F1//PTPN4//RAB12//KLF2//CEBPB//NPNT//MAP3K5//ZFAND6//MAPK1//SLC2A4//CREBRF//EPS8//NR5A2//PCOLCE2//JARID2//NEFH//PFKP//PIGA//BCAT2//SOX17//ARID5B//GFPT2//LRCH1//CD274//JUN//FOSL1// |
| GO:0006677 | glycosylceramide metabolic process | Biological process | 4 | 18 | 934 | 17653 | 4.20009517011658 | 0.0131386385291841 | 0.0999942288556065 | 1.88144963552942 | 0.00428265524625268 | B3GALT2//PRKAA1//ST6GALNAC3//ST6GALNAC6// |
| GO:0010002 | cardioblast differentiation | Biological process | 4 | 18 | 934 | 17653 | 4.20009517011658 | 0.0131386385291841 | 0.0999942288556065 | 1.88144963552942 | 0.00428265524625268 | GATA6//TGFB2//NRG1//REST// |
| GO:0051767 | nitric-oxide synthase biosynthetic process | Biological process | 4 | 18 | 934 | 17653 | 4.20009517011658 | 0.0131386385291841 | 0.0999942288556065 | 1.88144963552942 | 0.00428265524625268 | KDR//CCL2//MAP2K4//EDN1// |
| GO:0051769 | regulation of nitric-oxide synthase biosynthetic process | Biological process | 4 | 18 | 934 | 17653 | 4.20009517011658 | 0.0131386385291841 | 0.0999942288556065 | 1.88144963552942 | 0.00428265524625268 | KDR//CCL2//MAP2K4//EDN1// |
| GO:0010639 | negative regulation of organelle organization | Biological process | 29 | 354 | 934 | 17653 | 1.54834016864467 | 0.0132372248463887 | 0.0999942288556065 | 1.87820305418848 | 0.0310492505353319 | EPS8//TWF1//STMN2//MID1IP1//BNIP3//LIMA1//PFN2//SLIT2//JARID2//ATAD2//DCP2//CEP97//MTBP//RAD21//MDM1//KAT2B//DLC1//S1PR1//TMEFF2//ARHGAP6//MET//PPFIA1//DNMT3B//TMOD3//TMOD1//IGF1//DUSP1//FEZ2//MTPN// |
| GO:0003407 | neural retina development | Biological process | 8 | 60 | 934 | 17653 | 2.52005710206995 | 0.0132857022603701 | 0.0999942288556065 | 1.87661548455496 | 0.00856531049250535 | TOPORS//FJX1//LHX1//TFAP2A//NEUROD1//GPM6A//SLC17A7//TGFB2// |
| GO:0030166 | proteoglycan biosynthetic process | Biological process | 8 | 60 | 934 | 17653 | 2.52005710206995 | 0.0132857022603701 | 0.0999942288556065 | 1.87661548455496 | 0.00856531049250535 | XYLT2//HS2ST1//HS3ST5//SLC35D1//CHST7//DSEL//IGF1//BMPR2// |
| GO:0045600 | positive regulation of fat cell differentiation | Biological process | 8 | 60 | 934 | 17653 | 2.52005710206995 | 0.0132857022603701 | 0.0999942288556065 | 1.87661548455496 | 0.00856531049250535 | PRDM16//CEBPB//CMKLR1//CCDC71L//TMEM64//FRZB//PPARG//LMO3// |
| GO:0045669 | positive regulation of osteoblast differentiation | Biological process | 8 | 60 | 934 | 17653 | 2.52005710206995 | 0.0132857022603701 | 0.0999942288556065 | 1.87661548455496 | 0.00856531049250535 | CEBPB//NPNT//GJA1//IGF1//FAM20C//BMPR2//SOX11//ACVR1// |
| GO:0048706 | embryonic skeletal system development | Biological process | 13 | 123 | 934 | 17653 | 1.99760623944569 | 0.0132944071764113 | 0.0999942288556065 | 1.87633102394005 | 0.0139186295503212 | OSR1//TFAP2A//TGFBR2//RDH10//LHX1//HOXA3//DSCAML1//SOX11//ZEB1//NOG//SLC35D1//SULF1//HOXD1// |
| GO:0048199 | vesicle targeting, to, from or within Golgi | Biological process | 9 | 72 | 934 | 17653 | 2.36255353319058 | 0.0133650728200734 | 0.0999942288556065 | 1.87402867063211 | 0.00963597430406852 | TMED9//CNIH1//SEC23A//TBC1D20//GRIA1//NSF//SAR1B//PPP6C//MCFD2// |
| GO:0000290 | deadenylation-dependent decapping of nuclear-transcribed mRNA | Biological process | 3 | 10 | 934 | 17653 | 5.67012847965739 | 0.0133894584296735 | 0.0999942288556065 | 1.87323698876551 | 0.00321199143468951 | DCP2//PAN3//CNOT7// |
| GO:0002328 | pro-B cell differentiation | Biological process | 3 | 10 | 934 | 17653 | 5.67012847965739 | 0.0133894584296735 | 0.0999942288556065 | 1.87323698876551 | 0.00321199143468951 | SOS1//FNIP1//SOX4// |
| GO:0003149 | membranous septum morphogenesis | Biological process | 3 | 10 | 934 | 17653 | 5.67012847965739 | 0.0133894584296735 | 0.0999942288556065 | 1.87323698876551 | 0.00321199143468951 | TGFB2//TGFBR2//NOG// |
| GO:0007028 | cytoplasm organization | Biological process | 3 | 10 | 934 | 17653 | 5.67012847965739 | 0.0133894584296735 | 0.0999942288556065 | 1.87323698876551 | 0.00321199143468951 | SOS1//FOSL1//TDRD5// |
| GO:0021892 | cerebral cortex GABAergic interneuron differentiation | Biological process | 3 | 10 | 934 | 17653 | 5.67012847965739 | 0.0133894584296735 | 0.0999942288556065 | 1.87323698876551 | 0.00321199143468951 | ARX//DRD1//LHX6// |
| GO:0033327 | Leydig cell differentiation | Biological process | 3 | 10 | 934 | 17653 | 5.67012847965739 | 0.0133894584296735 | 0.0999942288556065 | 1.87323698876551 | 0.00321199143468951 | AR//PLEKHA1//TMF1// |
| GO:0048262 | determination of dorsal/ventral asymmetry | Biological process | 3 | 10 | 934 | 17653 | 5.67012847965739 | 0.0133894584296735 | 0.0999942288556065 | 1.87323698876551 | 0.00321199143468951 | AIDA//SOSTDC1//ACVR1// |
| GO:0060513 | prostatic bud formation | Biological process | 3 | 10 | 934 | 17653 | 5.67012847965739 | 0.0133894584296735 | 0.0999942288556065 | 1.87323698876551 | 0.00321199143468951 | AR//SULF1//NOG// |
| GO:0070341 | fat cell proliferation | Biological process | 3 | 10 | 934 | 17653 | 5.67012847965739 | 0.0133894584296735 | 0.0999942288556065 | 1.87323698876551 | 0.00321199143468951 | E2F1//E2F3//PER2// |
| GO:0070344 | regulation of fat cell proliferation | Biological process | 3 | 10 | 934 | 17653 | 5.67012847965739 | 0.0133894584296735 | 0.0999942288556065 | 1.87323698876551 | 0.00321199143468951 | E2F1//E2F3//PER2// |
| GO:1990253 | cellular response to leucine starvation | Biological process | 3 | 10 | 934 | 17653 | 5.67012847965739 | 0.0133894584296735 | 0.0999942288556065 | 1.87323698876551 | 0.00321199143468951 | SESN3//SESN1//RRAGD// |
| GO:2000341 | regulation of chemokine (C-X-C motif) ligand 2 production | Biological process | 3 | 10 | 934 | 17653 | 5.67012847965739 | 0.0133894584296735 | 0.0999942288556065 | 1.87323698876551 | 0.00321199143468951 | KLF4//MCOLN2//TNF// |
| GO:0006900 | vesicle budding from membrane | Biological process | 11 | 97 | 934 | 17653 | 2.14334753526568 | 0.0133956485795031 | 0.0999942288556065 | 1.87303625423343 | 0.0117773019271949 | TMED9//CNIH1//SEC23A//TBC1D20//GRIA1//NSF//SAR1B//PPP6C//MCFD2//MIA3//WASL// |
| GO:0048640 | negative regulation of developmental growth | Biological process | 11 | 97 | 934 | 17653 | 2.14334753526568 | 0.0133956485795031 | 0.0999942288556065 | 1.87303625423343 | 0.0117773019271949 | EPHA4//ADRB1//PTEN//EPHA7//ULK2//SEMA4B//GJA1//JARID2//TGFBR2//NOG//DUSP10// |
| GO:0017015 | regulation of transforming growth factor beta receptor signaling pathway | Biological process | 12 | 110 | 934 | 17653 | 2.0618649016936 | 0.0134545642868286 | 0.100212795453548 | 1.87113036188313 | 0.012847965738758 | NPNT//TGFB1I1//BAMBI//ASPN//PMEPA1//PRDM16//RASL11B//TGFBR2//LDLRAD4//DKK3//SOX11//ZEB1// |
| GO:0030330 | DNA damage response, signal transduction by p53 class mediator | Biological process | 12 | 110 | 934 | 17653 | 2.0618649016936 | 0.0134545642868286 | 0.100212795453548 | 1.87113036188313 | 0.012847965738758 | CDKN1A//GADD45A//E2F1//CNOT6L//CNOT7//MDM4//CNOT6//RBL2//SOX4//BTG2//SPRED1//PMAIP1// |
| GO:0042060 | wound healing | Biological process | 41 | 542 | 934 | 17653 | 1.42973719351755 | 0.0136971485855273 | 0.101907388211134 | 1.86336983302314 | 0.0438972162740899 | F3//COL1A2//ENPP4//DOCK9//ZFPM2//GATA6//GNAQ//IFNA1//IRF1//NFE2//PRKACB//PRKAR2B//PLEK//IGF1//WNT7A//DGKH//P2RY1//PIK3CB//MAPK1//TRPC3//FZD6//DGKE//EDN1//ITGA5//MTPN//GJA1//TGFBR2//TMEFF2//RHOC//ETS1//ADIPOR2//CRK//PTEN//DUSP10//ELK3//EREG//NRG1//MIA3//MAP3K5//TGFB2//NOG// |
| GO:0006865 | amino acid transport | Biological process | 14 | 137 | 934 | 17653 | 1.93143062567405 | 0.0137776906080696 | 0.102393979760851 | 1.86082357199473 | 0.0149892933618844 | ARL6IP1//SLC6A14//SLC32A1//SLC6A6//SLC17A7//SLC17A6//SNAP25//PPFIA1//SLC6A1//SERINC3//KCNJ10//PER2//RAB3GAP1//OSR1// |
| GO:0051271 | negative regulation of cellular component movement | Biological process | 28 | 340 | 934 | 17653 | 1.55650585716085 | 0.0138176160133564 | 0.102577977056344 | 1.85956688037391 | 0.0299785867237687 | SLIT2//DUSP10//PFN2//PTPRG//C5ORF30//DLC1//SULF1//TMEFF2//CLIC4//MIA3//NF2//PTEN//RAP2C//LDLRAD4//RECK//NOG//PPARG//SEMA4B//SPRED1//MEOX2//KLF4//CRK//WASL//LRCH1//PTGER4//CCL2//NRG1//STAT3// |
| GO:0044843 | cell cycle G1/S phase transition | Biological process | 25 | 295 | 934 | 17653 | 1.60173120894276 | 0.0139231088076928 | 0.102890323448268 | 1.85626378288042 | 0.0267665952890792 | CDK6//CDKN1A//LATS2//PPP6C//RBBP8//ACVR1//CCNE2//E2F1//NPAT//RRM2//GADD45A//CNOT6L//CNOT7//MDM4//CNOT6//RBL2//SOX4//BTG2//MTBP//PTEN//PKD2//CCND2//FBXW7//ZNF655//KLF4// |
| GO:0031076 | embryonic camera-type eye development | Biological process | 6 | 38 | 934 | 17653 | 2.9842781471881 | 0.013964685244274 | 0.102890323448268 | 1.8549688485928 | 0.00642398286937901 | TFAP2A//FOXF2//LRP6//ZEB1//SOX11//RDH10// |
| GO:0035307 | positive regulation of protein dephosphorylation | Biological process | 6 | 38 | 934 | 17653 | 2.9842781471881 | 0.013964685244274 | 0.102890323448268 | 1.8549688485928 | 0.00642398286937901 | CALM2//PPP1R15B//PPP1R12A//DLC1//CNEP1R1//NSMF// |
| GO:0045022 | early endosome to late endosome transport | Biological process | 6 | 38 | 934 | 17653 | 2.9842781471881 | 0.013964685244274 | 0.102890323448268 | 1.8549688485928 | 0.00642398286937901 | CHMP3//MAPK1//RAB21//MSN//AKTIP//FAM160A2// |
| GO:1901030 | positive regulation of mitochondrial outer membrane permeabilization involved in apoptotic signaling pathway | Biological process | 6 | 38 | 934 | 17653 | 2.9842781471881 | 0.013964685244274 | 0.102890323448268 | 1.8549688485928 | 0.00642398286937901 | BCL2L11//DYNLL2//E2F1//PMAIP1//PPP3R1//YWHAG// |
| GO:0044089 | positive regulation of cellular component biogenesis | Biological process | 39 | 511 | 934 | 17653 | 1.44249843905178 | 0.0139661861489738 | 0.102890323448268 | 1.85492217374185 | 0.0417558886509636 | CNOT6L//PAN3//CNOT6//CDC42EP3//GRB2//NCK1//PFN2//MET//SLAIN2//CLIP1//TNF//FNIP1//PLEK//PMAIP1//BCL2L11//RHOC//IQGAP2//ARFIP1//ESR1//WASL//FMR1//GPM6A//LPAR1//PPM1E//NF2//TSC1//PPM1F//KDR//EFNA5//SLITRK3//FLRT1//WNT7A//EDN1//MSN//EPS8//TSG101//JUN//FOSL1//RAB3GAP1// |
| GO:1903827 | regulation of cellular protein localization | Biological process | 39 | 511 | 934 | 17653 | 1.44249843905178 | 0.0139661861489738 | 0.102890323448268 | 1.85492217374185 | 0.0417558886509636 | CDKN1A//IGF1//MAPK1//IPO5//MDFIC//LYPLA1//PKDCC//SOX4//CTDSPL2//DNAJC27//MIEF1//NF2//CD2AP//BCL2L11//DYNLL2//E2F1//PMAIP1//PPP3R1//YWHAG//MSN//APPL1//AR//PPFIA1//EPHA2//SQSTM1//NPTN//NRG1//FBXW7//ELMOD1//PRKAA1//SNIP1//DERL2//EDEM1//CCT6A//CENPQ//TNF//TSG101//STX6//CCNE2// |
| GO:0009611 | response to wounding | Biological process | 48 | 655 | 934 | 17653 | 1.38506955228272 | 0.0140240686343798 | 0.103204326631459 | 1.85312597145366 | 0.0513918629550321 | F3//COL1A2//ENPP4//DOCK9//ZFPM2//GATA6//GNAQ//IFNA1//IRF1//NFE2//PRKACB//PRKAR2B//PLEK//FOXF1//IGF1//WNT7A//DGKH//P2RY1//PIK3CB//MAPK1//TRPC3//FZD6//DGKE//EDN1//JUN//ITGA5//ELK3//EREG//NRG1//MIA3//MAP3K5//TGFB2//TGFBR2//NOG//MTPN//GJA1//TMEFF2//RHOC//KLF4//EPHA4//RGMA//PTEN//ETS1//ADIPOR2//CRK//DUSP10//INHBB//MAP2K4// |
| GO:1902806 | regulation of cell cycle G1/S phase transition | Biological process | 18 | 193 | 934 | 17653 | 1.76273424238053 | 0.0140907656552728 | 0.10358244361588 | 1.85106540782565 | 0.019271948608137 | CDKN1A//GADD45A//E2F1//CNOT6L//CNOT7//MDM4//CNOT6//RBL2//SOX4//BTG2//MTBP//CDK6//PTEN//PKD2//CCND2//ZNF655//KLF4//FBXW7// |
| GO:0003300 | cardiac muscle hypertrophy | Biological process | 11 | 98 | 934 | 17653 | 2.12147664204868 | 0.0143924027474885 | 0.105684929187041 | 1.84186669648265 | 0.0117773019271949 | MTPN//EDN1//IGF1//TWF1//NR4A3//HAND2//JARID2//GATA6//MAP2K4//ERRFI1//TRPC3// |
| GO:0005975 | carbohydrate metabolic process | Biological process | 46 | 624 | 934 | 17653 | 1.39330080162521 | 0.0144803144193357 | 0.106086472086004 | 1.83922200795241 | 0.0492505353319058 | CHST7//CHST1//UGP2//PER2//SERP1//PRKAA1//TNF//MAN2A1//IDH1//NUP35//NUP43//NLN//ST6GALNAC3//ST6GALNAC6//ST8SIA3//ST3GAL3//ST6GAL2//ST3GAL5//B3GALT2//PMAIP1//PLEK//HS2ST1//B3GNT5//P2RY1//KAT2B//FUT9//IGF1//STAT3//NAGK//GNPTG//PTEN//UGT1A10//UGT1A7//UGT1A6//UGT1A9//UGT1A4//UGT1A1//UGT1A3//UGT1A8//UGT1A5//PFKP//CNOT7//ST8SIA5//UEVLD//CASD1//SLC2A4// |
| GO:0006687 | glycosphingolipid metabolic process | Biological process | 9 | 73 | 934 | 17653 | 2.33018978616057 | 0.0145519471879563 | 0.106086472086004 | 1.83707889008674 | 0.00963597430406852 | ITGB8//ST6GALNAC3//ST6GALNAC6//ST8SIA3//ST3GAL5//B3GALT2//ST8SIA5//PRKAA1//CTSA// |
| GO:0106027 | neuron projection organization | Biological process | 9 | 73 | 934 | 17653 | 2.33018978616057 | 0.0145519471879563 | 0.106086472086004 | 1.83707889008674 | 0.00963597430406852 | CTNND2//PTEN//WNT7A//WASL//EPHA4//EFNA1//CAPRIN2//LRP8//ZNF804A// |
| GO:0051649 | establishment of localization in cell | Biological process | 134 | 2117 | 934 | 17653 | 1.1963426488334 | 0.0145542982123922 | 0.106086472086004 | 1.83700873078398 | 0.143468950749465 | RANBP6//IPO5//TNF//DRD1//STAT3//BCL2L11//PMAIP1//MOAP1//ZFAND6//SRSF2//NUP35//AGFG1//NUP43//TSC1//PAN3//YWHAG//EGR2//ZDHHC1//ARL6IP1//RPL15//MTX3//IMMP2L//MID1IP1//STX6//SEC23A//RAB12//STX2//RAB21//TBC1D12//RAB38//SNX5//ARFIP1//RAB30//NSF//SAR1B//RAB9B//DNAJC27//RAB23//TMED9//CTSA//RAB22A//KIF13A//SNX2//EVI5//TBC1D17//AP1S2//RAB28//ZFYVE9//USP6NL//CNIH1//DCTN6//TBC1D20//DYNLL2//DYNC1LI2//TRAPPC8//GRIA1//MIA3//IER3IP1//MCFD2//EPS15//NEUROD1//ANP32E//CDC23//SLC32A1//SNAP25//WNT7A//PPFIA1//FMR1//FXR1//AKTIP//TSG101//FAM160A2//TRPC3//CALM2//VPS37A//ARHGAP1//CHMP3//DPY30//SQSTM1//GNPTG//PEX5L//SYBU//WASL//KIF5A//DERL2//NPLOC4//PKD2//DENND1B//CDKN1A//IGF1//MAPK1//PIKFYVE//TMEM87A//ZFYVE21//MDFIC//NR4A3//FOXF1//CLIP1//PPP1R12A//SOX4//CTDSPL2//PPP6C//FCHO2//SYT6//ATXN1//CENPQ//KCNJ10//PER2//EFNA5//EPHA5//RAB3GAP1//FYCO1//KIF23//LDLR//LRP6//MIEF1//SLC17A7//SLC17A6//TRPV6//FAM155A//E2F1//PPP3R1//NEFH//PLAA//NRG1//FBXW7//ELMOD1//PRKAA1//SNIP1//EDEM1//ANKRD50//PFN2//MSN//PTEN// |
| GO:0009266 | response to temperature stimulus | Biological process | 21 | 237 | 934 | 17653 | 1.67472149188193 | 0.0145879360496565 | 0.106086472086004 | 1.83600614920677 | 0.0224839400428266 | DNAJB4//IGF1//TGFB1I1//ADRB1//PPARG//TMEM135//THRA//PRKAA1//CDKN1A//CETN1//ATXN3//IER5//HSPA13//SLC12A5//HSBP1//RBBP7//PTGES3//NUP35//NUP43//MAPK1//BAG5// |
| GO:0050730 | regulation of peptidyl-tyrosine phosphorylation | Biological process | 21 | 237 | 934 | 17653 | 1.67472149188193 | 0.0145879360496565 | 0.106086472086004 | 1.83600614920677 | 0.0224839400428266 | EREG//FBXW7//ERRFI1//TSG101//SOCS5//CNOT7//IGF1//IL2//OSM//STAT3//NF2//PPP2CA//EFNA1//EFNA5//NRG1//ITGA5//NTF3//LRP8//RAP2C//EPHA4//EPHA7// |
| GO:0001702 | gastrulation with mouth forming second | Biological process | 5 | 28 | 934 | 17653 | 3.37507647598654 | 0.0146024182363862 | 0.106086472086004 | 1.83557521683528 | 0.00535331905781585 | LHX1//LRP6//OTX2//RNF2//ACVR1// |
| GO:0060292 | long term synaptic depression | Biological process | 5 | 28 | 934 | 17653 | 3.37507647598654 | 0.0146024182363862 | 0.106086472086004 | 1.83557521683528 | 0.00535331905781585 | FMR1//DRD1//SLC24A2//GRIA1//PTEN// |
| GO:0071385 | cellular response to glucocorticoid stimulus | Biological process | 8 | 61 | 934 | 17653 | 2.47874469056061 | 0.0146039487671255 | 0.106086472086004 | 1.83552969929077 | 0.00856531049250535 | DNMT3B//GJB2//ERRFI1//CREBRF//BCL2L11//EDN1//UGT1A1//REST// |
| GO:1902110 | positive regulation of mitochondrial membrane permeability involved in apoptotic process | Biological process | 8 | 61 | 934 | 17653 | 2.47874469056061 | 0.0146039487671255 | 0.106086472086004 | 1.83552969929077 | 0.00856531049250535 | BCL2L11//PMAIP1//MOAP1//BNIP3//DYNLL2//E2F1//PPP3R1//YWHAG// |
| GO:0050678 | regulation of epithelial cell proliferation | Biological process | 29 | 357 | 934 | 17653 | 1.53532890672329 | 0.0147087539583572 | 0.106733157747178 | 1.83242411666272 | 0.0310492505353319 | SULF1//GJA1//TNF//F3//JUN//KDR//BMPR2//VEGFB//VASH2//BCL11B//OSR1//IGF1//NME1//NR4A3//NOG//CDK6//DUSP10//EREG//AR//PTEN//TGFB2//RUNX3//WNT7A//ERRFI1//PPARG//STAT3//FBXW7//NFIB//SOX11// |
| GO:0090288 | negative regulation of cellular response to growth factor stimulus | Biological process | 15 | 152 | 934 | 17653 | 1.86517384199256 | 0.0148690454819024 | 0.107780658728945 | 1.82771691010939 | 0.0160599571734475 | BAMBI//ASPN//PMEPA1//PRDM16//RASL11B//TGFB1I1//TGFBR2//LDLRAD4//TOB1//SOSTDC1//CTDSPL2//NOG//SULF1//SHISA2//SLIT2// |
| GO:0034329 | cell junction assembly | Biological process | 20 | 223 | 934 | 17653 | 1.69510567403808 | 0.0149508072469865 | 0.10823358930318 | 1.82533535761066 | 0.0214132762312634 | GJC1//CDH20//ITGA6//ITGA5//GJA1//GJB2//LAMA3//RHOC//DLC1//ARHGAP6//EFNA5//SLK//KDR//TSC1//PPM1F//PTEN//STRN//RAP1B//TNF//CBFB// |
| GO:0035967 | cellular response to topologically incorrect protein | Biological process | 16 | 166 | 934 | 17653 | 1.82172802559274 | 0.0149635377788664 | 0.10823358930318 | 1.82496571554922 | 0.0171306209850107 | CREBRF//SERP1//DERL2//HSPA13//DNAJB9//WFS1//EDEM1//CCL2//ATXN3//DNAJB12//UBE2W//KLHL15//BAG6//BCL2L11//NCK1//PPP1R15B// |
| GO:0007346 | regulation of mitotic cell cycle | Biological process | 49 | 674 | 934 | 17653 | 1.37406674333933 | 0.0150555845948505 | 0.108715056415346 | 1.82230237655849 | 0.0524625267665953 | CDKN1A//GADD45A//E2F1//CNOT6L//CNOT7//MDM4//CNOT6//RBL2//SOX4//BTG2//MTBP//CDC23//NABP1//TAOK3//HMGA2//CDC14A//CDK6//NEDD1//PRKAR2B//YWHAG//HAUS8//CEP41//RAD21//SKP1//HECW2//NEUROG1//PTEN//PKD2//EDN1//EREG//IGF1//BTG3//FOXC1//BTG1//TNF//DUSP1//CCND2//CEP97//TMOD3//ZNF655//KLF4//CDK8//MAP3K2//ZNF268//CDK19//MINK1//PAK6//MAP2K4//SLK// |
| GO:0032355 | response to estradiol | Biological process | 13 | 125 | 934 | 17653 | 1.96564453961456 | 0.0150622516429365 | 0.108715056415346 | 1.82211010097675 | 0.0139186295503212 | ESR1//H2AFZ//POU4F1//UGT1A1//DNMT3B//DUSP1//ETS1//GJB2//PTEN//RBBP8//SLC6A1//STAT3//WNT7A// |
| GO:0045216 | cell-cell junction organization | Biological process | 21 | 238 | 934 | 17653 | 1.66768484695806 | 0.0152321179443883 | 0.109823895157674 | 1.81723970614456 | 0.0224839400428266 | GRHL1//GJC1//CDH20//GJA1//GJB2//CADM2//RASSF8//RHOC//DLC1//ARHGAP6//EFNA5//SLK//KDR//TSC1//PPM1F//PTEN//STRN//TNF//CBFB//NF2//TGFB2// |
| GO:0007160 | cell-matrix adhesion | Biological process | 19 | 209 | 934 | 17653 | 1.71822075141133 | 0.0152666018088106 | 0.109955301419581 | 1.8162576217465 | 0.0203426124197002 | MINK1//PIK3CB//TSC1//NF2//CDK6//DLC1//ARHGAP6//EFNA5//SLK//KDR//PPM1F//PTEN//BCL2L11//NPNT//ITGA6//ITGAV//PPFIA1//ADAM9//SGCE// |
| GO:0002065 | columnar/cuboidal epithelial cell differentiation | Biological process | 12 | 112 | 934 | 17653 | 2.02504588559192 | 0.0153618845888761 | 0.110406403267342 | 1.81355550199051 | 0.012847965738758 | RARB//GATA6//CDK6//DLL1//NEUROD1//SOX4//SOX11//CDKN1A//FAM20C//CEBPB//RHEB//CLOCK// |
| GO:1903844 | regulation of cellular response to transforming growth factor beta stimulus | Biological process | 12 | 112 | 934 | 17653 | 2.02504588559192 | 0.0153618845888761 | 0.110406403267342 | 1.81355550199051 | 0.012847965738758 | DKK3//SOX11//ZEB1//NPNT//TGFB1I1//BAMBI//ASPN//PMEPA1//PRDM16//RASL11B//TGFBR2//LDLRAD4// |
| GO:0035335 | peptidyl-tyrosine dephosphorylation | Biological process | 11 | 99 | 934 | 17653 | 2.10004758505829 | 0.0154440621436392 | 0.110879185007889 | 1.81123845953534 | 0.0117773019271949 | DUSP10//PTPN21//PTPDC1//DUSP1//DUSP2//DUSP5//PTEN//PTPN4//PTPRG//PTP4A1//CDC14A// |
| GO:0006979 | response to oxidative stress | Biological process | 34 | 436 | 934 | 17653 | 1.47388660786201 | 0.0155462721154788 | 0.111494632361594 | 1.80837373485409 | 0.0364025695931477 | MAP3K5//EDN1//STX2//CPEB2//VKORC1L1//GJB2//NR4A2//JUN//PKD2//MAPK1//CRK//DUSP1//NR4A3//FOSL1//PPP1R15B//ADAM9//KLF2//ETS1//MYB//PRKAA1//PLEKHA1//BNIP3//KLF4//EGLN1//SESN3//SESN1//MET//TNF//BAG5//TSC1//FBXW7//IDH1//PON2//BTG1// |
| GO:0051272 | positive regulation of cellular component movement | Biological process | 39 | 515 | 934 | 17653 | 1.43129456768051 | 0.0156654788117333 | 0.112230543648043 | 1.80505632646911 | 0.0417558886509636 | MIA3//CCL8//EDN1//ETS1//KDR//BMPR2//WNT7A//JUN//TGFB2//TGFBR2//DOCK5//PPM1F//CMKLR1//ADAM10//CRK//IGF1//SEMA4B//ZNF268//DRD1//S1PR1//F3//FOXF1//CD274//ITGA6//ITGA5//ITGAV//NTF3//MAPK1//PTP4A1//ACVR1//GAB1//ADAM9//VEGFB//LPAR1//TNF//CXCL14//MET//NSMF//PLAA// |
| GO:0050770 | regulation of axonogenesis | Biological process | 16 | 167 | 934 | 17653 | 1.81081947454128 | 0.0157662671086278 | 0.112833084080053 | 1.80227111998927 | 0.0171306209850107 | DPYSL2//RAB21//EPHA4//WNT7A//GOLGA4//NRG1//EPHA7//ULK2//EFNA5//LPAR3//BMPR2//SEMA4B//PTEN//SLIT2//EFNA1//POU3F2// |
| GO:0030835 | negative regulation of actin filament depolymerization | Biological process | 6 | 39 | 934 | 17653 | 2.9077581946961 | 0.0157855709579403 | 0.112851814364218 | 1.80173970522577 | 0.00642398286937901 | EPS8//TWF1//TMOD3//TMOD1//MTPN//LIMA1// |
| GO:0032482 | Rab protein signal transduction | Biological process | 9 | 74 | 934 | 17653 | 2.29870073499624 | 0.0158147630272733 | 0.112941121809345 | 1.80093731128658 | 0.00963597430406852 | RAB12//RAB21//RAB38//RAB30//RAB9B//DNAJC27//RAB23//RAB22A//RAB28// |
| GO:0003323 | type B pancreatic cell development | Biological process | 4 | 19 | 934 | 17653 | 3.97903752958413 | 0.0159594036412167 | 0.113494686462196 | 1.79698334106597 | 0.00428265524625268 | RHEB//CLOCK//CDK6//DLL1// |
| GO:0021533 | cell differentiation in hindbrain | Biological process | 4 | 19 | 934 | 17653 | 3.97903752958413 | 0.0159594036412167 | 0.113494686462196 | 1.79698334106597 | 0.00428265524625268 | LHX1//MTPN//WNT7A//NOG// |
| GO:0042474 | middle ear morphogenesis | Biological process | 4 | 19 | 934 | 17653 | 3.97903752958413 | 0.0159594036412167 | 0.113494686462196 | 1.79698334106597 | 0.00428265524625268 | TSHZ1//OSR1//EDN1//NOG// |
| GO:0060438 | trachea development | Biological process | 4 | 19 | 934 | 17653 | 3.97903752958413 | 0.0159594036412167 | 0.113494686462196 | 1.79698334106597 | 0.00428265524625268 | MAPK1//TGFBR2//LRP6//FOXF1// |
| GO:1901880 | negative regulation of protein depolymerization | Biological process | 8 | 62 | 934 | 17653 | 2.43876493748705 | 0.0160146948903389 | 0.1137682579237 | 1.79548133103728 | 0.00856531049250535 | EPS8//TWF1//STMN2//MID1IP1//LIMA1//TMOD3//TMOD1//MTPN// |
| GO:0010632 | regulation of epithelial cell migration | Biological process | 23 | 269 | 934 | 17653 | 1.61602174760991 | 0.0161905520346597 | 0.114896855624768 | 1.79073834324667 | 0.0246252676659529 | EDN1//ETS1//KDR//BMPR2//WNT7A//SLIT2//DUSP10//PFN2//PTPRG//JUN//TGFB2//TGFBR2//DOCK5//PPM1F//EFNA1//EPHA2//PPARG//PTEN//ADAM9//SPRED1//MEOX2//KLF4//MET// |
| GO:0006023 | aminoglycan biosynthetic process | Biological process | 12 | 113 | 934 | 17653 | 2.00712512554244 | 0.0163890504053544 | 0.115810042509748 | 1.78544620906903 | 0.012847965738758 | HS3ST5//XYLT2//HS2ST1//HS3ST3A1//HS3ST1//OGN//ST3GAL3//CHST1//SLC35D1//CHST7//DSEL//B3GNT5// |
| GO:0044774 | mitotic DNA integrity checkpoint | Biological process | 12 | 113 | 934 | 17653 | 2.00712512554244 | 0.0163890504053544 | 0.115810042509748 | 1.78544620906903 | 0.012847965738758 | CDKN1A//GADD45A//E2F1//CNOT6L//CNOT7//MDM4//CNOT6//RBL2//SOX4//BTG2//TAOK3//HMGA2// |
| GO:0072073 | kidney epithelium development | Biological process | 14 | 140 | 934 | 17653 | 1.89004282655246 | 0.016400822299609 | 0.115810042509748 | 1.78513437687731 | 0.0149892933618844 | GDF11//OSR1//FOXC1//NPNT//LHX1//RARB//TCF21//SLIT2//PKD2//NOG//DLL1//KLHL3//EPHA4//EPHA7// |
| GO:0010657 | muscle cell apoptotic process | Biological process | 10 | 87 | 934 | 17653 | 2.17246301902582 | 0.0164048529830458 | 0.115810042509748 | 1.78502765731927 | 0.0107066381156317 | BNIP3//MAP3K5//PTEN//HAND2//EDN1//IGF1//LRP6//MAP2K4//E2F3//PPARG// |
| GO:0048145 | regulation of fibroblast proliferation | Biological process | 10 | 87 | 934 | 17653 | 2.17246301902582 | 0.0164048529830458 | 0.115810042509748 | 1.78502765731927 | 0.0107066381156317 | CDK6//CDKN1A//E2F1//EREG//ESR1//IGF1//JUN//MORC3//MYB//PPARG// |
| GO:0014897 | striated muscle hypertrophy | Biological process | 11 | 100 | 934 | 17653 | 2.07904710920771 | 0.0165523754921899 | 0.116608036930917 | 1.78113967021953 | 0.0117773019271949 | MTPN//EDN1//IGF1//TWF1//NR4A3//HAND2//JARID2//GATA6//MAP2K4//ERRFI1//TRPC3// |
| GO:0061387 | regulation of extent of cell growth | Biological process | 11 | 100 | 934 | 17653 | 2.07904710920771 | 0.0165523754921899 | 0.116608036930917 | 1.78113967021953 | 0.0117773019271949 | DPYSL2//RAB21//EPHA4//GOLGA4//NRG1//EPHA7//ULK2//EFNA5//LPAR3//BMPR2//SEMA4B// |
| GO:0038127 | ERBB signaling pathway | Biological process | 15 | 154 | 934 | 17653 | 1.84095080508357 | 0.0165836732028183 | 0.116706953039189 | 1.78031926901906 | 0.0160599571734475 | EREG//FBXW7//ERRFI1//TSG101//SOCS5//EPS8//EPS15//GAB1//GRB2//SOS1//CAMLG//RPS6KA5//NRG1//EPN1//MAPK1// |
| GO:0010822 | positive regulation of mitochondrion organization | Biological process | 16 | 168 | 934 | 17653 | 1.80004078719282 | 0.0166019117380255 | 0.116713855596951 | 1.77984189934123 | 0.0171306209850107 | KDR//MIEF1//BNIP3//DDHD1//BCL2L11//PMAIP1//MOAP1//DYNLL2//E2F1//PPP3R1//YWHAG//NRG1//FBXW7//ELMOD1//PRKAA1//SNIP1// |
| GO:0003203 | endocardial cushion morphogenesis | Biological process | 5 | 29 | 934 | 17653 | 3.25869452853873 | 0.01689556538037 | 0.118042054408515 | 1.77222727074517 | 0.00535331905781585 | TMEM100//TGFB2//TGFBR2//ACVR1//NOG// |
| GO:0007176 | regulation of epidermal growth factor-activated receptor activity | Biological process | 5 | 29 | 934 | 17653 | 3.25869452853873 | 0.01689556538037 | 0.118042054408515 | 1.77222727074517 | 0.00535331905781585 | EREG//FBXW7//ERRFI1//TSG101//SOCS5// |
| GO:0021955 | central nervous system neuron axonogenesis | Biological process | 5 | 29 | 934 | 17653 | 3.25869452853873 | 0.01689556538037 | 0.118042054408515 | 1.77222727074517 | 0.00535331905781585 | NR4A2//SLIT2//EPHA4//NFIB//PTEN// |
| GO:0051567 | histone H3-K9 methylation | Biological process | 5 | 29 | 934 | 17653 | 3.25869452853873 | 0.01689556538037 | 0.118042054408515 | 1.77222727074517 | 0.00535331905781585 | ARID4B//ARID4A//DNMT3B//JARID2//MYB// |
| GO:0051955 | regulation of amino acid transport | Biological process | 5 | 29 | 934 | 17653 | 3.25869452853873 | 0.01689556538037 | 0.118042054408515 | 1.77222727074517 | 0.00535331905781585 | ARL6IP1//SLC6A1//PER2//RAB3GAP1//OSR1// |
| GO:2000272 | negative regulation of signaling receptor activity | Biological process | 5 | 29 | 934 | 17653 | 3.25869452853873 | 0.01689556538037 | 0.118042054408515 | 1.77222727074517 | 0.00535331905781585 | ERRFI1//TSG101//SOCS5//LYPD6//PTEN// |
| GO:0060420 | regulation of heart growth | Biological process | 9 | 75 | 934 | 17653 | 2.26805139186296 | 0.017156144176881 | 0.119738909255156 | 1.76558031269172 | 0.00963597430406852 | GJA1//JARID2//PTEN//TGFBR2//NOG//ZFPM2//GATA6//EDN1//IGF1// |
| GO:0043433 | negative regulation of DNA binding transcription factor activity | Biological process | 16 | 169 | 934 | 17653 | 1.78938965827452 | 0.0174713078884659 | 0.121687389546545 | 1.75767458285434 | 0.0171306209850107 | PKD2//THRA//G3BP2//CMKLR1//PIAS4//KLF4//WWP2//ESR1//RLIM//EGLN1//RNF2//WFS1//FZD6//BHLHE40//HAND1//HAND2// |
| GO:0046328 | regulation of JNK cascade | Biological process | 16 | 169 | 934 | 17653 | 1.78938965827452 | 0.0174713078884659 | 0.121687389546545 | 1.75767458285434 | 0.0171306209850107 | MAP3K2//MDFIC//MAP3K5//MAP2K4//FZD10//EDN1//EPHA4//TAOK3//TNF//HIPK3//DUSP10//AIDA//ZMYND11//GADD45A//MINK1//WNT7A// |
| GO:1902686 | mitochondrial outer membrane permeabilization involved in programmed cell death | Biological process | 8 | 63 | 934 | 17653 | 2.40005438292376 | 0.0175212764758477 | 0.121909869142138 | 1.75643425740179 | 0.00856531049250535 | BCL2L11//PMAIP1//MOAP1//BNIP3//DYNLL2//E2F1//PPP3R1//YWHAG// |
| GO:0048144 | fibroblast proliferation | Biological process | 10 | 88 | 934 | 17653 | 2.14777593926416 | 0.0176611660520804 | 0.122092713993602 | 1.75298062616806 | 0.0107066381156317 | CDK6//CDKN1A//E2F1//EREG//ESR1//IGF1//JUN//MORC3//MYB//PPARG// |
| GO:0003157 | endocardium development | Biological process | 3 | 11 | 934 | 17653 | 5.15466225423399 | 0.0176963260117731 | 0.122092713993602 | 1.75211688948198 | 0.00321199143468951 | SOX17//NRG1//ACVR1// |
| GO:0016264 | gap junction assembly | Biological process | 3 | 11 | 934 | 17653 | 5.15466225423399 | 0.0176963260117731 | 0.122092713993602 | 1.75211688948198 | 0.00321199143468951 | GJC1//GJA1//GJB2// |
| GO:0060394 | negative regulation of pathway-restricted SMAD protein phosphorylation | Biological process | 3 | 11 | 934 | 17653 | 5.15466225423399 | 0.0176963260117731 | 0.122092713993602 | 1.75211688948198 | 0.00321199143468951 | PMEPA1//LDLRAD4//NOG// |
| GO:0060439 | trachea morphogenesis | Biological process | 3 | 11 | 934 | 17653 | 5.15466225423399 | 0.0176963260117731 | 0.122092713993602 | 1.75211688948198 | 0.00321199143468951 | MAPK1//TGFBR2//LRP6// |
| GO:0070757 | interleukin-35-mediated signaling pathway | Biological process | 3 | 11 | 934 | 17653 | 5.15466225423399 | 0.0176963260117731 | 0.122092713993602 | 1.75211688948198 | 0.00321199143468951 | JAK1//STAT3//STAT4// |
| GO:0090009 | primitive streak formation | Biological process | 3 | 11 | 934 | 17653 | 5.15466225423399 | 0.0176963260117731 | 0.122092713993602 | 1.75211688948198 | 0.00321199143468951 | LHX1//LRP6//OTX2// |
| GO:0098712 | L-glutamate import across plasma membrane | Biological process | 3 | 11 | 934 | 17653 | 5.15466225423399 | 0.0176963260117731 | 0.122092713993602 | 1.75211688948198 | 0.00321199143468951 | ARL6IP1//KCNJ10//PER2// |
| GO:0001709 | cell fate determination | Biological process | 6 | 40 | 934 | 17653 | 2.83506423982869 | 0.0177641920108982 | 0.122092713993602 | 1.75045454123902 | 0.00642398286937901 | GATA6//SOX17//KLF4//WNT7A//DLL1//DSCAML1// |
| GO:0061647 | histone H3-K9 modification | Biological process | 6 | 40 | 934 | 17653 | 2.83506423982869 | 0.0177641920108982 | 0.122092713993602 | 1.75045454123902 | 0.00642398286937901 | ARID4B//ARID4A//KAT2B//DNMT3B//JARID2//MYB// |
| GO:0070169 | positive regulation of biomineral tissue development | Biological process | 6 | 40 | 934 | 17653 | 2.83506423982869 | 0.0177641920108982 | 0.122092713993602 | 1.75045454123902 | 0.00642398286937901 | OSR1//FAM20C//BMPR2//TFAP2A//ACVR1//PKDCC// |
| GO:1904031 | positive regulation of cyclin-dependent protein kinase activity | Biological process | 6 | 40 | 934 | 17653 | 2.83506423982869 | 0.0177641920108982 | 0.122092713993602 | 1.75045454123902 | 0.00642398286937901 | PKD2//CCNY//MAPRE3//CCNC//CCND2//CDKN1A// |
| GO:0030099 | myeloid cell differentiation | Biological process | 31 | 394 | 934 | 17653 | 1.48708953358189 | 0.0180589955003352 | 0.123992879765246 | 1.74330641021266 | 0.0331905781584582 | NME1//THRA//JUN//PPARG//NKX2-3//EPHA2//OSTM1//TNF//FLI1//FAM20C//TGFBR2//KLF2//CBFB//CDK6//DLL1//HMGB3//ETS1//STAT3//NFE2//NR4A3//DPY30//KAT2B//KMT2B//CEBPB//TOB2//TMEM64//POU4F1//KLF10//TMOD3//ARID4A//FBXW7// |
| GO:0018108 | peptidyl-tyrosine phosphorylation | Biological process | 30 | 379 | 934 | 17653 | 1.49607611600459 | 0.0183672540116207 | 0.12598147959492 | 1.73595576785006 | 0.0321199143468951 | EREG//FBXW7//ERRFI1//TSG101//SOCS5//GRB2//JAK1//KDR//CNOT7//IGF1//IL2//OSM//STAT3//NF2//PPP2CA//EFNA1//EPHA7//EFNA5//NRG1//ITGA5//NTF3//LRP8//RAP2C//EPHA4//EPHA2//EPHA5//DSTYK//MET//MAP2K4//PKDCC// |
| GO:0035050 | embryonic heart tube development | Biological process | 9 | 76 | 934 | 17653 | 2.23820861039107 | 0.0185786749873847 | 0.127173662894416 | 1.73098526268828 | 0.00963597430406852 | GJA1//DLL1//PKD2//SOX17//TGFBR2//HAND1//HAND2//ACVR1//FOXC1// |
| GO:0070167 | regulation of biomineral tissue development | Biological process | 9 | 76 | 934 | 17653 | 2.23820861039107 | 0.0185786749873847 | 0.127173662894416 | 1.73098526268828 | 0.00963597430406852 | S1PR1//GJA1//OSR1//FAM20C//BMPR2//TFAP2A//ACVR1//PKDCC//ASPN// |
| GO:0051098 | regulation of binding | Biological process | 28 | 349 | 934 | 17653 | 1.51636673763521 | 0.0189194918615938 | 0.129164431022786 | 1.7230905320288 | 0.0299785867237687 | BAMBI//AKTIP//CAPRIN2//BTAF1//FOXC1//IGF1//NME1//PPARG//E2F1//JUN//HMGA2//HAND2//CRK//NRG1//AIDA//P2RY1//NOG//FMR1//HAND1//MAPRE3//PPP2CA//KLF4//MET//SOX11//PER2//NEUROD1//POU4F1//EPHA4// |
| GO:0014896 | muscle hypertrophy | Biological process | 11 | 102 | 934 | 17653 | 2.0382814796154 | 0.0189458990942782 | 0.129164431022786 | 1.72248478007394 | 0.0117773019271949 | MTPN//EDN1//IGF1//TWF1//NR4A3//HAND2//JARID2//GATA6//MAP2K4//ERRFI1//TRPC3// |
| GO:0046854 | phosphatidylinositol phosphorylation | Biological process | 11 | 102 | 934 | 17653 | 2.0382814796154 | 0.0189458990942782 | 0.129164431022786 | 1.72248478007394 | 0.0117773019271949 | PIKFYVE//EREG//ESR1//EFR3A//GAB1//GRB2//NRG1//MET//PIK3CB//ZFYVE21//PIK3R3// |
| GO:0090100 | positive regulation of transmembrane receptor protein serine/threonine kinase signaling pathway | Biological process | 11 | 102 | 934 | 17653 | 2.0382814796154 | 0.0189458990942782 | 0.129164431022786 | 1.72248478007394 | 0.0117773019271949 | GDF11//INHBB//BMP3//BMPR2//TGFB2//ACVR1//NPNT//TGFB1I1//SULF1//GATA6//SOX11// |
| GO:0032446 | protein modification by small protein conjugation | Biological process | 63 | 915 | 934 | 17653 | 1.30134096254432 | 0.0190671289726073 | 0.129521973460645 | 1.71971469579631 | 0.0674518201284797 | TOPORS//WWP2//HECTD2//RNF145//ZNRF2//FBXL5//RNF180//MYLIP//RLIM//ANKIB1//UBE2W//FBXW7//HECW2//SKP1//THOP1//LONRF3//FBXO30//CBFB//UBE3B//LONRF1//TRIM3//KLHL2//RNF38//MYCBP2//FBXO33//KLHL3//SPOPL//MDM4//DCAF8//DCAF6//FEM1C//KLHL14//MARCH4//RNF4//AKTIP//MARCH7//UBE2Z//RNF128//KLHL15//KBTBD8//ARRDC4//ZFAND4//SOCS5//TSG101//NUP35//EGR2//NUP43//PIAS4//UBE2I//SOX4//PER2//BAG5//NDFIP2//WFS1//HMG20A//RNF2//PCGF5//ARRDC3//RNF141//CDC23//UBA5//PLAA//PTEN// |
| GO:0048207 | vesicle targeting, rough ER to cis-Golgi | Biological process | 8 | 64 | 934 | 17653 | 2.36255353319058 | 0.019126954985429 | 0.129521973460645 | 1.71835416422705 | 0.00856531049250535 | CNIH1//SEC23A//TBC1D20//GRIA1//NSF//SAR1B//PPP6C//MCFD2// |
| GO:0048208 | COPII vesicle coating | Biological process | 8 | 64 | 934 | 17653 | 2.36255353319058 | 0.019126954985429 | 0.129521973460645 | 1.71835416422705 | 0.00856531049250535 | CNIH1//SEC23A//TBC1D20//GRIA1//NSF//SAR1B//PPP6C//MCFD2// |
| GO:0003283 | atrial septum development | Biological process | 4 | 20 | 934 | 17653 | 3.78008565310493 | 0.0191324044783652 | 0.129521973460645 | 1.71823044627879 | 0.00428265524625268 | SOX4//TGFB2//ACVR1//BMPR2// |
| GO:0021697 | cerebellar cortex formation | Biological process | 4 | 20 | 934 | 17653 | 3.78008565310493 | 0.0191324044783652 | 0.129521973460645 | 1.71823044627879 | 0.00428265524625268 | DLL1//LHX1//MTPN//WNT7A// |
| GO:0044346 | fibroblast apoptotic process | Biological process | 4 | 20 | 934 | 17653 | 3.78008565310493 | 0.0191324044783652 | 0.129521973460645 | 1.71823044627879 | 0.00428265524625268 | IER3IP1//BCL2L11//BTG1//STK17B// |
| GO:1901889 | negative regulation of cell junction assembly | Biological process | 4 | 20 | 934 | 17653 | 3.78008565310493 | 0.0191324044783652 | 0.129521973460645 | 1.71823044627879 | 0.00428265524625268 | DLC1//ARHGAP6//PTEN//TNF// |
| GO:0045930 | negative regulation of mitotic cell cycle | Biological process | 26 | 319 | 934 | 17653 | 1.5404737771274 | 0.0193549373972935 | 0.130897441617896 | 1.71320822904944 | 0.0278372591006424 | CDKN1A//GADD45A//E2F1//CNOT6L//CNOT7//MDM4//CNOT6//RBL2//SOX4//BTG2//NABP1//TAOK3//HMGA2//RAD21//SKP1//MTBP//DUSP1//CDC14A//PKD2//PTEN//ZNF655//KLF4//BTG3//FOXC1//BTG1//TNF// |
| GO:0010564 | regulation of cell cycle process | Biological process | 53 | 750 | 934 | 17653 | 1.33563026409707 | 0.0193812577587472 | 0.130944501720687 | 1.71261804256085 | 0.056745182012848 | CDKN1A//GADD45A//E2F1//CNOT6L//CNOT7//MDM4//CNOT6//RBL2//SOX4//BTG2//MTBP//CDC23//TAOK3//HMGA2//CDC14A//CDK6//NEDD1//PRKAR2B//YWHAG//HAUS8//CEP41//CHMP3//RAD21//SKP1//HECW2//NEUROG1//PTEN//PKD2//KIF13A//CALM2//KIF23//FBXW7//EDN1//EREG//IGF1//MDM1//KAT2B//RBBP7//RBBP8//RNF2//RRM2//NEUROD1//ZNF268//GATA6//POU4F1//RNF4//DUSP1//CCND2//CEP97//TMOD3//YTHDF2//ZNF655//KLF4// |
| GO:0046685 | response to arsenic-containing substance | Biological process | 5 | 30 | 934 | 17653 | 3.15007137758744 | 0.0194153989153122 | 0.131044254355545 | 1.71185368186586 | 0.00535331905781585 | CPEB2//MKNK2//CDKN1A//PTEN//RNF4// |
| GO:0016192 | vesicle-mediated transport | Biological process | 129 | 2050 | 934 | 17653 | 1.18934402256228 | 0.0194529712899247 | 0.131166944001755 | 1.71101405417051 | 0.138115631691649 | FMR1//NTF3//LHFPL2//IGF1//NHLRC2//PLEK//TMX3//CDC37L1//TGFB2//VEGFB//STX6//SYT6//STX2//SNAP25//EXOC5//MIA3//NSF//CCL8//CNIH1//SEC23A//DCTN6//TBC1D20//DYNLL2//DYNC1LI2//TRAPPC8//GRIA1//IER3IP1//SAR1B//TMED9//MCFD2//KIF5A//ERGIC2//KIF23//GOLGA3//MYO1B//OSBPL5//STEAP2//EPS15//DENND1B//DPYSL2//NECAP1//SNX5//EPN1//LRP12//LDLR//NME1//RAB22A//SNX2//LRP8//WASL//TRIP10//ZFYVE9//LMBR1L//TGFBR2//VLDLR//ARHGAP12//RAB12//KIF13A//AKTIP//TSG101//FAM160A2//VPS37A//ARHGAP1//CHMP3//DPY30//SQSTM1//RAPGEF4//RAB21//TRPV6//RUFY2//TBC1D12//EVI5//TBC1D17//USP6NL//GRB2//CRK//NCK1//PIK3CB//MAPK1//PIKFYVE//TMEM87A//RAB9B//ZFYVE21//LYPLA1//PKDCC//GOLGA4//C16ORF70//ITGAV//NR4A3//FOXF1//ADAM10//IQGAP2//CKAP4//ENPP4//GNS//IDH1//IMPDH1//C6ORF120//CTSA//RAP2C//RAP1B//TTR//LAMTOR3//ORMDL3//XKR8//DLL1//REST//PPP6C//CD2AP//FCHO2//PTEN//TNF//PPARG//PRKAA1//LRP6//RAB3GAP1//ANKRD50//PFN2//CCL2//MSN//RTN3//ARAP2//RAB38//APPL1//AGFG1//AGFG2//HSPA13//AP1S2//GIT2// |
| GO:0010948 | negative regulation of cell cycle process | Biological process | 28 | 350 | 934 | 17653 | 1.51203426124197 | 0.0195679227763429 | 0.131776637274778 | 1.70845527422205 | 0.0299785867237687 | CDKN1A//GADD45A//E2F1//CNOT6L//CNOT7//MDM4//CNOT6//RBL2//SOX4//BTG2//TAOK3//HMGA2//RAD21//SKP1//FBXW7//MTBP//MDM1//KAT2B//RBBP7//RBBP8//RNF2//RRM2//ZNF268//DUSP1//PKD2//PTEN//ZNF655//KLF4// |
| GO:0007254 | JNK cascade | Biological process | 18 | 200 | 934 | 17653 | 1.70103854389722 | 0.0195914895280974 | 0.131776637274778 | 1.70793254362541 | 0.019271948608137 | MAP3K2//MDFIC//MAP3K5//MAP2K4//FZD10//EDN1//EPHA4//TAOK3//TNF//HIPK3//DUSP10//AIDA//ZMYND11//GADD45A//MINK1//WNT7A//CRKL//PTGER4// |
| GO:0022402 | cell cycle process | Biological process | 92 | 1408 | 934 | 17653 | 1.23497116507689 | 0.0196018478631416 | 0.131776637274778 | 1.70770298584057 | 0.0985010706638116 | KIF23//CDK6//CDKN1A//LATS2//PPP6C//RBBP8//ACVR1//CCNE2//E2F1//NPAT//RRM2//NEDD1//CCNY//PPP1R12A//PRKAR2B//SKP1//YWHAG//MASTL//HAUS8//CEP41//RHOC//RND3//PAK6//GADD45A//CNOT6L//CNOT7//MDM4//CNOT6//RBL2//SOX4//BTG2//HBP1//MTBP//IRF1//PKD2//PRKAA1//RRAGD//RHEB//TGFB2//TSC1//TSG101//LAMTOR3//KAT2B//DYNLL2//DYNC1LI2//NUP43//CENPQ//FBXW7//RAD21//CLIP1//CENPO//NUP35//CNEP1R1//CDC23//REEP3//PPP2CA//NABP1//TAOK3//HMGA2//CDC14A//BCL2L11//BAG6//MYBL1//SYCP3//EREG//EPS8//CHMP3//HECW2//NEUROG1//PTEN//KIF13A//CALM2//EDN1//IGF1//MDM1//GOLGA8A//WASL//RBBP7//RNF2//NEUROD1//ZNF268//GATA6//POU4F1//DUSP1//RNF4//MZT1//CCND2//CEP97//TMOD3//YTHDF2//ZNF655//KLF4// |
| GO:0008543 | fibroblast growth factor receptor signaling pathway | Biological process | 12 | 116 | 934 | 17653 | 1.95521671712324 | 0.0197820949737305 | 0.132856314108579 | 1.70372771742275 | 0.012847965738758 | SULF1//OTX2//FAM20C//SHISA2//DSTYK//NPTN//NOG//SPRED1//FLRT1//GRB2//ESRP1//MAPK1// |
| GO:0021575 | hindbrain morphogenesis | Biological process | 6 | 41 | 934 | 17653 | 2.76591633154019 | 0.0199063300270455 | 0.133293574230603 | 1.70100880003871 | 0.00642398286937901 | LRP6//DLL1//LHX1//MTPN//WNT7A//DLC1// |
| GO:0035136 | forelimb morphogenesis | Biological process | 6 | 41 | 934 | 17653 | 2.76591633154019 | 0.0199063300270455 | 0.133293574230603 | 1.70100880003871 | 0.00642398286937901 | OSR1//RDH10//EN1//TFAP2A//WNT7A//RECK// |
| GO:0098927 | vesicle-mediated transport between endosomal compartments | Biological process | 6 | 41 | 934 | 17653 | 2.76591633154019 | 0.0199063300270455 | 0.133293574230603 | 1.70100880003871 | 0.00642398286937901 | AKTIP//FAM160A2//CHMP3//MAPK1//RAB21//MSN// |
| GO:0021675 | nerve development | Biological process | 9 | 77 | 934 | 17653 | 2.20914096610028 | 0.0200848936021976 | 0.134223453983856 | 1.69713046455969 | 0.00963597430406852 | LPAR1//NEUROG1//POU4F1//TFAP2A//EGR2//HOXA3//SULF1//LRIG1//NTF3// |
| GO:0061097 | regulation of protein tyrosine kinase activity | Biological process | 9 | 77 | 934 | 17653 | 2.20914096610028 | 0.0200848936021976 | 0.134223453983856 | 1.69713046455969 | 0.00963597430406852 | EREG//FBXW7//ERRFI1//TSG101//SOCS5//EFNA1//EPHA4//LRP8//RAP2C// |
| GO:0018212 | peptidyl-tyrosine modification | Biological process | 30 | 382 | 934 | 17653 | 1.48432682713544 | 0.0202303758933088 | 0.134956779763945 | 1.69399604768529 | 0.0321199143468951 | EREG//FBXW7//ERRFI1//TSG101//SOCS5//EPHA2//EPHA4//EPHA5//EPHA7//DSTYK//NRG1//KDR//MET//MAP2K4//PKDCC//GRB2//JAK1//CNOT7//IGF1//IL2//OSM//STAT3//NF2//PPP2CA//EFNA1//EFNA5//ITGA5//NTF3//LRP8//RAP2C// |
| GO:0051261 | protein depolymerization | Biological process | 11 | 103 | 934 | 17653 | 2.01849233903661 | 0.020234537140417 | 0.134956779763945 | 1.69390672552801 | 0.0117773019271949 | EPS8//TWF1//CFL2//STMN2//MID1IP1//LIMA1//PLEK//TMOD3//TMOD1//ASPH//MTPN// |
| GO:0097193 | intrinsic apoptotic signaling pathway | Biological process | 24 | 290 | 934 | 17653 | 1.56417337369859 | 0.0203662165535532 | 0.135701204484414 | 1.69108964269977 | 0.0256959314775161 | BCL2L11//TOPORS//E2F1//EPHA2//MOAP1//TNF//MAP3K5//CDKN1A//USP28//BAG6//CEBPB//PMAIP1//POU4F1//ZMAT3//BAG5//PIAS4//WFS1//SERINC3//NCK1//FBXW7//BNIP3//URI1//SGPP1//PPM1F// |
| GO:0045927 | positive regulation of growth | Biological process | 22 | 260 | 934 | 17653 | 1.59926700708285 | 0.0204570577433754 | 0.136172324329181 | 1.68915682896711 | 0.0235546038543897 | ADAM10//MTPN//IL2//DERL2//TGFB2//POU3F2//TGFBR2//GOLGA4//NRG1//ARX//SERP1//DLL1//EFNA5//LPAR3//BMPR2//ZFPM2//GATA6//EDN1//IGF1//PLAA//WFS1//PPM1F// |
| GO:0003143 | embryonic heart tube morphogenesis | Biological process | 8 | 65 | 934 | 17653 | 2.32620655575688 | 0.0208349073682776 | 0.13776808309805 | 1.68120842606993 | 0.00856531049250535 | GJA1//DLL1//PKD2//SOX17//TGFBR2//HAND1//HAND2//ACVR1// |
| GO:0035794 | positive regulation of mitochondrial membrane permeability | Biological process | 8 | 65 | 934 | 17653 | 2.32620655575688 | 0.0208349073682776 | 0.13776808309805 | 1.68120842606993 | 0.00856531049250535 | BCL2L11//PMAIP1//MOAP1//BNIP3//DYNLL2//E2F1//PPP3R1//YWHAG// |
| GO:0042733 | embryonic digit morphogenesis | Biological process | 8 | 65 | 934 | 17653 | 2.32620655575688 | 0.0208349073682776 | 0.13776808309805 | 1.68120842606993 | 0.00856531049250535 | FZD6//BCL2L11//OSR1//GJA1//GNAQ//WNT7A//NOG//HAND2// |
| GO:0071384 | cellular response to corticosteroid stimulus | Biological process | 8 | 65 | 934 | 17653 | 2.32620655575688 | 0.0208349073682776 | 0.13776808309805 | 1.68120842606993 | 0.00856531049250535 | BCL2L11//EDN1//UGT1A1//REST//DNMT3B//GJB2//ERRFI1//CREBRF// |
| GO:1903313 | positive regulation of mRNA metabolic process | Biological process | 8 | 65 | 934 | 17653 | 2.32620655575688 | 0.0208349073682776 | 0.13776808309805 | 1.68120842606993 | 0.00856531049250535 | TOB1//CPEB3//CNOT7//BTG2//CNOT6L//YTHDF2//QKI//NANOS1// |
| GO:0044770 | cell cycle phase transition | Biological process | 44 | 606 | 934 | 17653 | 1.37230832290938 | 0.0208535678001426 | 0.13776808309805 | 1.68081963165625 | 0.0471092077087794 | CDK6//CDKN1A//LATS2//PPP6C//RBBP8//ACVR1//CCNE2//E2F1//NPAT//RRM2//NEDD1//CCNY//PPP1R12A//PRKAR2B//SKP1//YWHAG//MASTL//HAUS8//CEP41//GADD45A//CNOT6L//CNOT7//MDM4//CNOT6//RBL2//SOX4//BTG2//MTBP//CDC23//TAOK3//HMGA2//CDC14A//EPS8//RAD21//HECW2//NEUROG1//PTEN//PKD2//DUSP1//CCND2//TMOD3//FBXW7//ZNF655//KLF4// |
| GO:0032872 | regulation of stress-activated MAPK cascade | Biological process | 19 | 216 | 934 | 17653 | 1.66253767150448 | 0.0208583722823315 | 0.13776808309805 | 1.68071958547724 | 0.0203426124197002 | MAP3K2//MDFIC//MAP3K5//MAP2K4//TAOK3//TGFB2//FZD10//EDN1//EPHA4//TNF//HIPK3//DUSP10//AIDA//ZMYND11//GADD45A//MINK1//WNT7A//HAND2//MAPK1// |
| GO:0006584 | catecholamine metabolic process | Biological process | 7 | 53 | 934 | 17653 | 2.49628297846552 | 0.0209412375313907 | 0.13776808309805 | 1.67899765708061 | 0.00749464668094218 | DRD1//NR4A2//RNF180//TGFB2//HPRT1//HAND2//MTPN// |
| GO:0009712 | catechol-containing compound metabolic process | Biological process | 7 | 53 | 934 | 17653 | 2.49628297846552 | 0.0209412375313907 | 0.13776808309805 | 1.67899765708061 | 0.00749464668094218 | MTPN//DRD1//NR4A2//RNF180//TGFB2//HPRT1//HAND2// |
| GO:0021879 | forebrain neuron differentiation | Biological process | 7 | 53 | 934 | 17653 | 2.49628297846552 | 0.0209412375313907 | 0.13776808309805 | 1.67899765708061 | 0.00749464668094218 | BCL11B//ARX//SLIT2//DRD1//LHX6//GNAQ//LHX8// |
| GO:0043392 | negative regulation of DNA binding | Biological process | 7 | 53 | 934 | 17653 | 2.49628297846552 | 0.0209412375313907 | 0.13776808309805 | 1.67899765708061 | 0.00749464668094218 | HAND1//SOX11//PER2//E2F1//JUN//HMGA2//HAND2// |
| GO:0071398 | cellular response to fatty acid | Biological process | 7 | 53 | 934 | 17653 | 2.49628297846552 | 0.0209412375313907 | 0.13776808309805 | 1.67899765708061 | 0.00749464668094218 | PPARG//PRKAA1//PTGER4//E2F1//EDN1//LDLR//NME1// |
| GO:0006352 | DNA-templated transcription, initiation | Biological process | 21 | 246 | 934 | 17653 | 1.61345119339844 | 0.0212279991655534 | 0.139405709051007 | 1.67309093811423 | 0.0224839400428266 | GTF2E1//CDK8//CDKN1A//E2F3//ESR1//NR5A2//NRBF2//AR//NR3C2//NR4A2//PPARG//RARB//TEAD1//THRA//NR4A3//CBFB//KAT2B//CCNC//BDP1//JUN//FOSL1// |
| GO:0051258 | protein polymerization | Biological process | 23 | 276 | 934 | 17653 | 1.57503568879372 | 0.0212313884847756 | 0.139405709051007 | 1.67302160302863 | 0.0246252676659529 | EPS8//TWF1//RANBP9//SLAIN2//WASF3//ARHGAP6//PFN2//SLIT2//CDC42EP3//GRB2//NCK1//STMN2//MET//CLIP1//IQGAP2//ARFIP1//WASL//MZT1//TMOD3//TMOD1//MTPN//CHMP3//MID1IP1// |
| GO:0051648 | vesicle localization | Biological process | 22 | 261 | 934 | 17653 | 1.5931395472856 | 0.0212685454881617 | 0.139514231946108 | 1.67226220958315 | 0.0235546038543897 | SNAP25//WASL//STX2//TMED9//CNIH1//SEC23A//TBC1D20//GRIA1//NSF//SAR1B//PPP6C//MCFD2//KIF5A//SYT6//KIF13A//FYCO1//KIF23//RAB3GAP1//SYBU//PFN2//FMR1//PTEN// |
| GO:0007266 | Rho protein signal transduction | Biological process | 18 | 202 | 934 | 17653 | 1.68419657811606 | 0.0214324880383198 | 0.140453407561198 | 1.66892740998514 | 0.019271948608137 | ARAP2//FGD4//FGD5//EPS8//ARHGEF4//ARHGEF28//SOS1//SOS2//DLC1//MET//LPAR1//CDC42EP3//COL1A2//RHOC//RND3//ARHGAP1//ARHGAP6//PAK6// |
| GO:0000077 | DNA damage checkpoint | Biological process | 15 | 159 | 934 | 17653 | 1.78305927033251 | 0.0215336381073104 | 0.14097966555638 | 1.66688258994193 | 0.0160599571734475 | CDKN1A//GADD45A//E2F1//CNOT6L//CNOT7//MDM4//CNOT6//RBL2//SOX4//BTG2//TAOK3//HMGA2//RBBP8//USP28//CLOCK// |
| GO:0044344 | cellular response to fibroblast growth factor stimulus | Biological process | 14 | 145 | 934 | 17653 | 1.82486893598169 | 0.0215991992459515 | 0.141100559106981 | 1.66556234928939 | 0.0149892933618844 | SPRED1//FLRT1//GRB2//ESRP1//MAPK1//SULF1//OTX2//FAM20C//SHISA2//DSTYK//NPTN//NOG//LHX1//CCL2// |
| GO:0055123 | digestive system development | Biological process | 14 | 145 | 934 | 17653 | 1.82486893598169 | 0.0215991992459515 | 0.141100559106981 | 1.66556234928939 | 0.0149892933618844 | FOXF1//SOX11//TCF21//GATA6//ITGA6//TGFBR2//FOXF2//RARB//TGFB2//TNF//PKDCC//SOX17//NKX2-3//CDKN1A// |
| GO:0031346 | positive regulation of cell projection organization | Biological process | 28 | 353 | 934 | 17653 | 1.499184111713 | 0.0216189024198081 | 0.141100559106981 | 1.66516635872317 | 0.0299785867237687 | STMN2//NPTN//NCK1//NME1//SERPINI1//TWF1//ZNF804A//CDC42EP3//GOLGA4//NRG1//EFNA5//LPAR3//BMPR2//SLIT2//EPHA4//RAB21//CAPRIN2//FMR1//GPM6A//WASL//LPAR1//CPEB3//IL2//C21ORF91//LRP8//VLDLR//EPS8//PLAA// |
| GO:0007009 | plasma membrane organization | Biological process | 9 | 78 | 934 | 17653 | 2.18081864602207 | 0.021677285363323 | 0.141100559106981 | 1.66399510523079 | 0.00963597430406852 | PLSCR4//PTEN//TMEFF2//AR//TGFB2//XKR8//WASL//STX2//SERP1// |
| GO:0043407 | negative regulation of MAP kinase activity | Biological process | 9 | 78 | 934 | 17653 | 2.18081864602207 | 0.021677285363323 | 0.141100559106981 | 1.66399510523079 | 0.00963597430406852 | DUSP10//SPRED1//DUSP1//DUSP2//DUSP5//PPP2CA//RGS3//HIPK3//AIDA// |
| GO:1901216 | positive regulation of neuron death | Biological process | 9 | 78 | 934 | 17653 | 2.18081864602207 | 0.021677285363323 | 0.141100559106981 | 1.66399510523079 | 0.00963597430406852 | BCL2L11//EPHA7//JUN//MYB//MAP2K4//TFAP2A//TGFB2//FBXW7//MAP3K5// |
| GO:0070302 | regulation of stress-activated protein kinase signaling cascade | Biological process | 19 | 217 | 934 | 17653 | 1.6548762075805 | 0.0217712809010947 | 0.141576127628946 | 1.66211601873509 | 0.0203426124197002 | MAP3K2//MDFIC//MAP3K5//MAP2K4//MAPK1//TAOK3//TGFB2//FZD10//EDN1//EPHA4//TNF//HIPK3//DUSP10//AIDA//ZMYND11//GADD45A//MINK1//WNT7A//HAND2// |
| GO:0030316 | osteoclast differentiation | Biological process | 10 | 91 | 934 | 17653 | 2.07697013906864 | 0.021855326182528 | 0.141986139262668 | 1.66044270744407 | 0.0107066381156317 | FAM20C//CEBPB//TOB2//TMEM64//POU4F1//KLF10//TNF//FBXW7//EPHA2//OSTM1// |
| GO:0090068 | positive regulation of cell cycle process | Biological process | 23 | 277 | 934 | 17653 | 1.56934963937569 | 0.02204137200575 | 0.143057388555554 | 1.65676137551985 | 0.0246252676659529 | CDKN1A//GADD45A//E2F1//CNOT6L//CNOT7//MDM4//CNOT6//RBL2//SOX4//BTG2//MTBP//NEUROG1//KIF23//EDN1//EREG//IGF1//RAD21//GATA6//PKD2//POU4F1//HMGA2//CCND2//TMOD3// |
| GO:0014002 | astrocyte development | Biological process | 5 | 31 | 934 | 17653 | 3.04845617185881 | 0.0221696296866593 | 0.14333958467579 | 1.65424156112023 | 0.00535331905781585 | LDLR//CDK6//DRD1//DLL1//POU3F2// |
| GO:0044319 | wound healing, spreading of cells | Biological process | 5 | 31 | 934 | 17653 | 3.04845617185881 | 0.0221696296866593 | 0.14333958467579 | 1.65424156112023 | 0.00535331905781585 | ITGA5//WNT7A//PTEN//TMEFF2//RHOC// |
| GO:0090505 | epiboly involved in wound healing | Biological process | 5 | 31 | 934 | 17653 | 3.04845617185881 | 0.0221696296866593 | 0.14333958467579 | 1.65424156112023 | 0.00535331905781585 | ITGA5//WNT7A//TMEFF2//RHOC//PTEN// |
| GO:1902235 | regulation of endoplasmic reticulum stress-induced intrinsic apoptotic signaling pathway | Biological process | 5 | 31 | 934 | 17653 | 3.04845617185881 | 0.0221696296866593 | 0.14333958467579 | 1.65424156112023 | 0.00535331905781585 | WFS1//BCL2L11//SERINC3//NCK1//PMAIP1// |
| GO:0008154 | actin polymerization or depolymerization | Biological process | 18 | 203 | 934 | 17653 | 1.67590004324849 | 0.022401221425486 | 0.143653209165027 | 1.64972830113518 | 0.019271948608137 | EPS8//TWF1//CFL2//WASF3//ARHGAP6//PFN2//LIMA1//PLEK//SLIT2//CDC42EP3//GRB2//NCK1//IQGAP2//ARFIP1//WASL//TMOD3//TMOD1//MTPN// |
| GO:0031960 | response to corticosteroid | Biological process | 15 | 160 | 934 | 17653 | 1.77191514989293 | 0.0226448698407475 | 0.143653209165027 | 1.64503017123219 | 0.0160599571734475 | DUSP1//TNF//ADAM9//CDKN1A//FOSL1//SLIT2//BCL2L11//EDN1//UGT1A1//REST//FIBIN//DNMT3B//GJB2//ERRFI1//CREBRF// |
| GO:0048670 | regulation of collateral sprouting | Biological process | 4 | 21 | 934 | 17653 | 3.60008157438564 | 0.0226684637377681 | 0.143653209165027 | 1.64457791140492 | 0.00428265524625268 | EPHA7//ULK2//EFNA5//LPAR3// |
| GO:0060065 | uterus development | Biological process | 4 | 21 | 934 | 17653 | 3.60008157438564 | 0.0226684637377681 | 0.143653209165027 | 1.64457791140492 | 0.00428265524625268 | LHX1//WNT7A//ESR1//TGFB2// |
| GO:0071498 | cellular response to fluid shear stress | Biological process | 4 | 21 | 934 | 17653 | 3.60008157438564 | 0.0226684637377681 | 0.143653209165027 | 1.64457791140492 | 0.00428265524625268 | KLF2//KLF4//SOCS5//PKD2// |
| GO:1903649 | regulation of cytoplasmic transport | Biological process | 4 | 21 | 934 | 17653 | 3.60008157438564 | 0.0226684637377681 | 0.143653209165027 | 1.64457791140492 | 0.00428265524625268 | CHMP3//MAPK1//RAB21//MSN// |
| GO:0003376 | sphingosine-1-phosphate signaling pathway | Biological process | 3 | 12 | 934 | 17653 | 4.72510706638116 | 0.0226824872293797 | 0.143653209165027 | 1.64430932496568 | 0.00321199143468951 | S1PR1//GPR6//SPHK2// |
| GO:0021559 | trigeminal nerve development | Biological process | 3 | 12 | 934 | 17653 | 4.72510706638116 | 0.0226824872293797 | 0.143653209165027 | 1.64430932496568 | 0.00321199143468951 | NEUROG1//POU4F1//TFAP2A// |
| GO:0021903 | rostrocaudal neural tube patterning | Biological process | 3 | 12 | 934 | 17653 | 4.72510706638116 | 0.0226824872293797 | 0.143653209165027 | 1.64430932496568 | 0.00321199143468951 | EN1//LRP6//SOX17// |
| GO:0033631 | cell-cell adhesion mediated by integrin | Biological process | 3 | 12 | 934 | 17653 | 4.72510706638116 | 0.0226824872293797 | 0.143653209165027 | 1.64430932496568 | 0.00321199143468951 | NPNT//ITGA5//ADAM9// |
| GO:0034392 | negative regulation of smooth muscle cell apoptotic process | Biological process | 3 | 12 | 934 | 17653 | 4.72510706638116 | 0.0226824872293797 | 0.143653209165027 | 1.64430932496568 | 0.00321199143468951 | IGF1//EDN1//LRP6// |
| GO:0043201 | response to leucine | Biological process | 3 | 12 | 934 | 17653 | 4.72510706638116 | 0.0226824872293797 | 0.143653209165027 | 1.64430932496568 | 0.00321199143468951 | SESN3//SESN1//RRAGD// |
| GO:0043922 | negative regulation by host of viral transcription | Biological process | 3 | 12 | 934 | 17653 | 4.72510706638116 | 0.0226824872293797 | 0.143653209165027 | 1.64430932496568 | 0.00321199143468951 | JUN//REST//HMGA2// |
| GO:0060536 | cartilage morphogenesis | Biological process | 3 | 12 | 934 | 17653 | 4.72510706638116 | 0.0226824872293797 | 0.143653209165027 | 1.64430932496568 | 0.00321199143468951 | LRP6//HAND1//HAND2// |
| GO:0060736 | prostate gland growth | Biological process | 3 | 12 | 934 | 17653 | 4.72510706638116 | 0.0226824872293797 | 0.143653209165027 | 1.64430932496568 | 0.00321199143468951 | ESR1//AR//PTEN// |
| GO:0070493 | thrombin-activated receptor signaling pathway | Biological process | 3 | 12 | 934 | 17653 | 4.72510706638116 | 0.0226824872293797 | 0.143653209165027 | 1.64430932496568 | 0.00321199143468951 | MET//IQGAP2//PLEK// |
| GO:0071257 | cellular response to electrical stimulus | Biological process | 3 | 12 | 934 | 17653 | 4.72510706638116 | 0.0226824872293797 | 0.143653209165027 | 1.64430932496568 | 0.00321199143468951 | NSMF//PTEN//REST// |
| GO:0072567 | chemokine (C-X-C motif) ligand 2 production | Biological process | 3 | 12 | 934 | 17653 | 4.72510706638116 | 0.0226824872293797 | 0.143653209165027 | 1.64430932496568 | 0.00321199143468951 | MCOLN2//KLF4//TNF// |
| GO:0090520 | sphingolipid mediated signaling pathway | Biological process | 3 | 12 | 934 | 17653 | 4.72510706638116 | 0.0226824872293797 | 0.143653209165027 | 1.64430932496568 | 0.00321199143468951 | S1PR1//GPR6//SPHK2// |
| GO:0099563 | modification of synaptic structure | Biological process | 3 | 12 | 934 | 17653 | 4.72510706638116 | 0.0226824872293797 | 0.143653209165027 | 1.64430932496568 | 0.00321199143468951 | EPHA4//FMR1//FXR1// |
| GO:2000543 | positive regulation of gastrulation | Biological process | 3 | 12 | 934 | 17653 | 4.72510706638116 | 0.0226824872293797 | 0.143653209165027 | 1.64430932496568 | 0.00321199143468951 | OSR1//LHX1//OTX2// |
| GO:0007173 | epidermal growth factor receptor signaling pathway | Biological process | 13 | 132 | 934 | 17653 | 1.86140581402894 | 0.0226854395073561 | 0.143653209165027 | 1.64425280230184 | 0.0139186295503212 | EREG//FBXW7//ERRFI1//TSG101//SOCS5//EPS15//GRB2//EPN1//SOS1//EPS8//GAB1//CAMLG//RPS6KA5// |
| GO:0001885 | endothelial cell development | Biological process | 7 | 54 | 934 | 17653 | 2.45005551590134 | 0.0229989433482418 | 0.145126799356189 | 1.6382921165289 | 0.00749464668094218 | CLIC4//MET//WNT7A//MSN//RAP1B//RAP2C//TNF// |
| GO:0003229 | ventricular cardiac muscle tissue development | Biological process | 7 | 54 | 934 | 17653 | 2.45005551590134 | 0.0229989433482418 | 0.145126799356189 | 1.6382921165289 | 0.00749464668094218 | ZFPM2//NRG1//TGFB2//POU4F1//NOG//FOXC1//HAND1// |
| GO:0060043 | regulation of cardiac muscle cell proliferation | Biological process | 7 | 54 | 934 | 17653 | 2.45005551590134 | 0.0229989433482418 | 0.145126799356189 | 1.6382921165289 | 0.00749464668094218 | GJA1//JARID2//PTEN//TGFBR2//NOG//ZFPM2//GATA6// |
| GO:0010508 | positive regulation of autophagy | Biological process | 11 | 105 | 934 | 17653 | 1.9800448659121 | 0.0230039817994728 | 0.145126799356189 | 1.63819698468376 | 0.0117773019271949 | SESN3//RAB12//SESN1//KDR//BNIP3//TSC1//CALCOCO2//FYCO1//RAB3GAP1//PIK3CB//PRKAA1// |
| GO:0044703 | multi-organism reproductive process | Biological process | 67 | 992 | 934 | 17653 | 1.27654102196588 | 0.0232027058604837 | 0.146244081765565 | 1.63446136546458 | 0.0717344753747323 | EREG//YTHDF2//TBC1D20//AGFG1//TMF1//BMPR2//MYBL1//SYCP3//MASTL//DZIP1//RNF2//ACVR1//BCL2L11//CRTAP//DDX6//E2F1//GOLGA3//AR//CLDN11//ACOX1//PAIP2//ARID4B//ARID4A//PLEKHA1//HERPUD2//SOX17//BTG1//BAG6//IMMP2L//ADAM18//CLOCK//TDRD5//QKI//TOB2//SOS1//FOSL1//LHFPL2//ADCY3//TRPC3//STX2//GLRA1//GLRB//ETS1//EPN1//SPHK2//PTHLH//ADIPOR2//MMP2//TGFBR2//RECK//DRD1//NHLH2//THRA//FOXC1//CLIC4//KMT2B//STAT3//GJA1//GJB2//INHBB//EDN1//PTEN//DMRTA1//IGF1//P2RY1//SYT6//ESR1// |
| GO:0046323 | glucose import | Biological process | 9 | 79 | 934 | 17653 | 2.15321334670534 | 0.023358276365293 | 0.146950719124164 | 1.63155920747831 | 0.00963597430406852 | IGF1//ADIPOR2//SLC2A4//APPL1//TNF//PRKAA1//PIK3R3//DRD1//TSC1// |
| GO:0046902 | regulation of mitochondrial membrane permeability | Biological process | 9 | 79 | 934 | 17653 | 2.15321334670534 | 0.023358276365293 | 0.146950719124164 | 1.63155920747831 | 0.00963597430406852 | BCL2L11//PMAIP1//MOAP1//BNIP3//DYNLL2//E2F1//PPP3R1//YWHAG//STAT3// |
| GO:0030097 | hemopoiesis | Biological process | 58 | 842 | 934 | 17653 | 1.30192973800526 | 0.0236247682911792 | 0.148489133785544 | 1.62663244230466 | 0.0620985010706638 | ESCO2//PLEK//RBM47//REST//DLL1//NKX2-3//FNIP1//SOX4//NME1//CBFB//KLF6//IFNA1//DNAJB9//IL2//THRA//JUN//PPARG//EPHA2//OSTM1//TNF//BCL11B//SOS1//ZEB1//KDR//TGFBR2//KLF4//FLI1//FAM20C//PTGER4//KLF2//IRF1//TSC1//HMGB3//DUSP10//MYB//SOCS5//CDK6//ETS1//STAT3//NFE2//NR4A3//DPY30//KAT2B//KMT2B//CEBPB//TOB2//TMEM64//POU4F1//KLF10//TMOD3//ARID4A//MFAP5//FOXC1//YTHDF2//FBXW7//MKNK2//TGFB2//RUNX3// |
| GO:0051650 | establishment of vesicle localization | Biological process | 21 | 249 | 934 | 17653 | 1.59401202239364 | 0.0238958093816543 | 0.150053257983406 | 1.62167825478399 | 0.0224839400428266 | SNAP25//WASL//STX2//TMED9//CNIH1//SEC23A//TBC1D20//GRIA1//NSF//SAR1B//PPP6C//MCFD2//KIF5A//SYT6//KIF13A//FYCO1//KIF23//RAB3GAP1//SYBU//PFN2//FMR1// |
| GO:0099504 | synaptic vesicle cycle | Biological process | 13 | 133 | 934 | 17653 | 1.84741028159263 | 0.0239732184725479 | 0.150382903107627 | 1.62027365678668 | 0.0139186295503212 | SNAP25//STX2//WNT7A//FCHO2//SYT6//SLC17A7//SLC32A1//SLC17A6//RAB3GAP1//PLAA//PFN2//FMR1//PTEN// |
| GO:0000187 | activation of MAPK activity | Biological process | 14 | 147 | 934 | 17653 | 1.80004078719282 | 0.0239927772368963 | 0.150382903107627 | 1.61991947821603 | 0.0149892933618844 | MAP3K2//MDFIC//MAP3K5//MAP2K4//DUSP5//LPAR1//LPAR3//NRG1//IGF1//NTF3//PIK3CB//PRKAA1//MAPK1//TNF// |
| GO:1901990 | regulation of mitotic cell cycle phase transition | Biological process | 32 | 419 | 934 | 17653 | 1.44346946180618 | 0.024046348780982 | 0.15057912667202 | 1.61895085794516 | 0.0342612419700214 | CDKN1A//GADD45A//E2F1//CNOT6L//CNOT7//MDM4//CNOT6//RBL2//SOX4//BTG2//MTBP//TAOK3//HMGA2//CDC14A//CDC23//CDK6//NEDD1//PRKAR2B//YWHAG//HAUS8//CEP41//RAD21//SKP1//HECW2//NEUROG1//PTEN//PKD2//DUSP1//CCND2//TMOD3//ZNF655//KLF4// |
| GO:0030500 | regulation of bone mineralization | Biological process | 8 | 67 | 934 | 17653 | 2.25676755409249 | 0.0245698606524541 | 0.153162226344844 | 1.60959730660669 | 0.00856531049250535 | OSR1//FAM20C//BMPR2//TFAP2A//ACVR1//PKDCC//S1PR1//GJA1// |
| GO:0070373 | negative regulation of ERK1 and ERK2 cascade | Biological process | 8 | 67 | 934 | 17653 | 2.25676755409249 | 0.0245698606524541 | 0.153162226344844 | 1.60959730660669 | 0.00856531049250535 | RANBP9//DUSP10//SPRED1//DUSP1//ERRFI1//LMO3//PTEN//KLF4// |
| GO:1902108 | regulation of mitochondrial membrane permeability involved in apoptotic process | Biological process | 8 | 67 | 934 | 17653 | 2.25676755409249 | 0.0245698606524541 | 0.153162226344844 | 1.60959730660669 | 0.00856531049250535 | BCL2L11//PMAIP1//MOAP1//BNIP3//DYNLL2//E2F1//PPP3R1//YWHAG// |
| GO:1905710 | positive regulation of membrane permeability | Biological process | 8 | 67 | 934 | 17653 | 2.25676755409249 | 0.0245698606524541 | 0.153162226344844 | 1.60959730660669 | 0.00856531049250535 | BCL2L11//PMAIP1//MOAP1//BNIP3//DYNLL2//E2F1//PPP3R1//YWHAG// |
| GO:0050803 | regulation of synapse structure or activity | Biological process | 16 | 176 | 934 | 17653 | 1.71822075141133 | 0.0245720856992689 | 0.153162226344844 | 1.60955797867501 | 0.0171306209850107 | EPHA7//EFNA5//SLITRK3//FLRT1//WNT7A//SYBU//EPHA4//EFNA1//PTEN//CAPRIN2//LRP8//ZNF804A//FMR1//FXR1//SLC17A7//SLC17A6// |
| GO:0045668 | negative regulation of osteoblast differentiation | Biological process | 6 | 43 | 934 | 17653 | 2.63726906030576 | 0.0247026192941599 | 0.153692561441033 | 1.60725699472862 | 0.00642398286937901 | TOB1//CDK6//TMEM64//TNF//NOG//HAND2// |
| GO:0061951 | establishment of protein localization to plasma membrane | Biological process | 6 | 43 | 934 | 17653 | 2.63726906030576 | 0.0247026192941599 | 0.153692561441033 | 1.60725699472862 | 0.00642398286937901 | LYPLA1//PKDCC//GOLGA4//NSF//KIF13A//C16ORF70// |
| GO:0070372 | regulation of ERK1 and ERK2 cascade | Biological process | 25 | 311 | 934 | 17653 | 1.51932703099073 | 0.0249745555456343 | 0.154709834816053 | 1.60250223186258 | 0.0267665952890792 | RANBP9//DUSP10//SPRED1//DUSP1//ERRFI1//LMO3//PTEN//KLF4//EPHA2//EPHA4//EPHA5//EPHA7//NPNT//DSTYK//NPTN//JUN//KDR//MET//P2RY1//DNAJC27//FBXW7//CCL2//CCL8//TNF//HAND2// |
| GO:0034614 | cellular response to reactive oxygen species | Biological process | 15 | 162 | 934 | 17653 | 1.75003965421524 | 0.0249959867444996 | 0.154709834816053 | 1.60212971431317 | 0.0160599571734475 | KLF2//ETS1//MAP3K5//MYB//PRKAA1//PLEKHA1//BNIP3//KLF4//CRK//MET//TNF//NR4A3//JUN//PKD2//MAPK1// |
| GO:1902115 | regulation of organelle assembly | Biological process | 15 | 162 | 934 | 17653 | 1.75003965421524 | 0.0249959867444996 | 0.154709834816053 | 1.60212971431317 | 0.0160599571734475 | CNOT6L//PAN3//CNOT6//CEP97//MDM1//KAT2B//EDN1//MSN//TNF//RNF4//FEZ2//TSG101//PIKFYVE//TBC1D12//RAB3GAP1// |
| GO:0048704 | embryonic skeletal system morphogenesis | Biological process | 10 | 93 | 934 | 17653 | 2.03230411457254 | 0.0250258634778349 | 0.154709834816053 | 1.60161092894597 | 0.0107066381156317 | OSR1//TFAP2A//TGFBR2//RDH10//LHX1//NOG//HOXA3//DSCAML1//SOX11//ZEB1// |
| GO:0070301 | cellular response to hydrogen peroxide | Biological process | 10 | 93 | 934 | 17653 | 2.03230411457254 | 0.0250258634778349 | 0.154709834816053 | 1.60161092894597 | 0.0107066381156317 | MET//NR4A3//KLF2//ETS1//MAP3K5//MYB//PRKAA1//PLEKHA1//BNIP3//KLF4// |
| GO:1903825 | organic acid transmembrane transport | Biological process | 12 | 120 | 934 | 17653 | 1.89004282655246 | 0.0250865853751588 | 0.154709834816053 | 1.60055844803759 | 0.012847965738758 | ARL6IP1//SLC6A14//SLC32A1//SLC6A6//SLC17A7//SLC17A6//MID1IP1//SLC35D1//KCNJ10//PER2//SLC25A32//OSR1// |
| GO:1905039 | carboxylic acid transmembrane transport | Biological process | 12 | 120 | 934 | 17653 | 1.89004282655246 | 0.0250865853751588 | 0.154709834816053 | 1.60055844803759 | 0.012847965738758 | ARL6IP1//SLC6A14//SLC32A1//SLC6A6//SLC17A7//SLC17A6//MID1IP1//SLC35D1//KCNJ10//PER2//SLC25A32//OSR1// |
| GO:0003333 | amino acid transmembrane transport | Biological process | 9 | 80 | 934 | 17653 | 2.12629817987152 | 0.0251302271794375 | 0.154709834816053 | 1.59980358532982 | 0.00963597430406852 | ARL6IP1//SLC17A7//SLC17A6//KCNJ10//PER2//OSR1//SLC6A14//SLC32A1//SLC6A6// |
| GO:0001504 | neurotransmitter uptake | Biological process | 5 | 32 | 934 | 17653 | 2.95319191648822 | 0.0251651857513617 | 0.154709834816053 | 1.59919985960447 | 0.00535331905781585 | ARL6IP1//DRD1//KCNJ10//PER2//SNAP25// |
| GO:0043403 | skeletal muscle tissue regeneration | Biological process | 5 | 32 | 934 | 17653 | 2.95319191648822 | 0.0251651857513617 | 0.154709834816053 | 1.59919985960447 | 0.00535331905781585 | IGF1//WNT7A//TGFBR2//MTPN//GJA1// |
| GO:0060603 | mammary gland duct morphogenesis | Biological process | 5 | 32 | 934 | 17653 | 2.95319191648822 | 0.0251651857513617 | 0.154709834816053 | 1.59919985960447 | 0.00535331905781585 | EPHA2//LRP6//AR//SOSTDC1//ESR1// |
| GO:0090504 | epiboly | Biological process | 5 | 32 | 934 | 17653 | 2.95319191648822 | 0.0251651857513617 | 0.154709834816053 | 1.59919985960447 | 0.00535331905781585 | ITGA5//WNT7A//TMEFF2//RHOC//PTEN// |
| GO:1902893 | regulation of pri-miRNA transcription by RNA polymerase II | Biological process | 5 | 32 | 934 | 17653 | 2.95319191648822 | 0.0251651857513617 | 0.154709834816053 | 1.59919985960447 | 0.00535331905781585 | NFIB//JUN//STAT3//TEAD1//TGFB2// |
| GO:0071345 | cellular response to cytokine stimulus | Biological process | 72 | 1081 | 934 | 17653 | 1.25886293720423 | 0.0251863859429949 | 0.154709834816053 | 1.5988341460239 | 0.0770877944325482 | CBFB//TNF//PIAS4//CDKN1A//COL1A2//CRK//CRKL//S1PR1//EREG//F3//FLRT1//GRB2//IFNA1//IL2//JAK1//MMP2//OSM//PIK3CB//ASPN//CCL2//SOS1//STAT3//STAT4//ZEB1//LRP8//SOCS5//TNFRSF21//ST18//ACSL1//ADIPOR2//IRF1//GSTA2//MSN//RAP1B//BRWD1//IRF9//PPARG//CNOT7//CMKLR1//FOXC1//CCL8//SLIT2//EDN1//SKP1//SQSTM1//RPS6KA4//RPS6KA5//RAB12//KLF2//CEBPB//NPNT//MAP3K5//ZFAND6//MAPK1//SLC2A4//CREBRF//EPS8//NR5A2//PCOLCE2//JARID2//NEFH//PFKP//PIGA//BCAT2//SOX17//ARID5B//KLF4//GFPT2//LRCH1//FOXF1//POU4F1//PTPN4// |
| GO:0031098 | stress-activated protein kinase signaling cascade | Biological process | 24 | 296 | 934 | 17653 | 1.53246715666416 | 0.0252756379115425 | 0.155117186203051 | 1.59729787478872 | 0.0256959314775161 | CRKL//MAP3K5//MINK1//PTGER4//MAP2K4//TNF//MAP3K2//MDFIC//MAPK1//TAOK3//TGFB2//FZD10//EDN1//EPHA4//HIPK3//DUSP10//AIDA//ZMYND11//GADD45A//WNT7A//SKP1//HAND2//PAK6//SLK// |
| GO:0042770 | signal transduction in response to DNA damage | Biological process | 13 | 134 | 934 | 17653 | 1.83362363770015 | 0.025314300742043 | 0.155213613706652 | 1.59663406464556 | 0.0139186295503212 | CDKN1A//GADD45A//E2F1//CNOT6L//CNOT7//MDM4//CNOT6//RBL2//SOX4//BTG2//SPRED1//PMAIP1//GRB2// |
| GO:0046488 | phosphatidylinositol metabolic process | Biological process | 19 | 221 | 934 | 17653 | 1.62492369703605 | 0.025732248651293 | 0.157633331185412 | 1.58952226065412 | 0.0203426124197002 | PIGA//CDS1//PIKFYVE//PIK3CB//PTEN//PLEKHA1//PLEKHA3//MTMR9//PIK3R3//GAB1//GRB2//EREG//ESR1//EFR3A//NRG1//MET//ZFYVE21//CHRM5//PLEK// |
| GO:0006024 | glycosaminoglycan biosynthetic process | Biological process | 11 | 107 | 934 | 17653 | 1.94303468150253 | 0.0260405737912884 | 0.159377738054736 | 1.58434945053223 | 0.0117773019271949 | OGN//ST3GAL3//CHST1//SLC35D1//CHST7//XYLT2//DSEL//HS3ST5//HS2ST1//HS3ST3A1//HS3ST1// |
| GO:0006644 | phospholipid metabolic process | Biological process | 35 | 470 | 934 | 17653 | 1.40747870062417 | 0.0264028203515471 | 0.161448710702996 | 1.57834967927821 | 0.0374732334047109 | PIGA//LCLAT1//DDHD1//CDS1//ABHD3//SLC44A1//SERINC3//PIKFYVE//PIK3CB//PTEN//PLEKHA1//PLEKHA3//MTMR9//PIK3R3//SGPP1//SPHK2//DGKE//NAPEPLD//LDLR//GAB1//GRB2//OSBPL5//KLF4//DGKH//PLEK//EREG//ESR1//EFR3A//NRG1//MET//ZFYVE21//CHRM5//IDH1//GATA6//PLAA// |
| GO:0010842 | retina layer formation | Biological process | 4 | 22 | 934 | 17653 | 3.43644150282266 | 0.0265762718163924 | 0.161937171915959 | 1.57550594299941 | 0.00428265524625268 | LHX1//TOPORS//FJX1//TFAP2A// |
| GO:0032799 | low-density lipoprotein receptor particle metabolic process | Biological process | 4 | 22 | 934 | 17653 | 3.43644150282266 | 0.0265762718163924 | 0.161937171915959 | 1.57550594299941 | 0.00428265524625268 | MYLIP//VLDLR//PPARG//ITGAV// |
| GO:0061157 | mRNA destabilization | Biological process | 4 | 22 | 934 | 17653 | 3.43644150282266 | 0.0265762718163924 | 0.161937171915959 | 1.57550594299941 | 0.00428265524625268 | CPEB3//QKI//CNOT6L//YTHDF2// |
| GO:0071695 | anatomical structure maturation | Biological process | 14 | 149 | 934 | 17653 | 1.77587916588822 | 0.0265784800867536 | 0.161937171915959 | 1.57546985818672 | 0.0149892933618844 | EREG//YTHDF2//S1PR1//MMP2//RECK//GJA1//GNAQ//NTN4//IGF1//FAM20C//NR4A2//KLF2//TGFB2//CDKN1A// |
| GO:0051403 | stress-activated MAPK cascade | Biological process | 22 | 267 | 934 | 17653 | 1.55733865858255 | 0.0266729542726625 | 0.162271157065456 | 1.57392887957599 | 0.0235546038543897 | CRKL//MAP3K5//MINK1//PTGER4//MAP2K4//TNF//MAP3K2//MDFIC//MAPK1//TAOK3//TGFB2//FZD10//EDN1//EPHA4//HIPK3//DUSP10//AIDA//ZMYND11//GADD45A//WNT7A//HAND2//SKP1// |
| GO:0021549 | cerebellum development | Biological process | 10 | 94 | 934 | 17653 | 2.01068385803453 | 0.0267292723600352 | 0.162271157065456 | 1.57301286370223 | 0.0107066381156317 | LRP6//DLL1//LHX1//MTPN//WNT7A//NANOS1//LPAR1//EN1//FOXC1//NEUROD1// |
| GO:0035282 | segmentation | Biological process | 10 | 94 | 934 | 17653 | 2.01068385803453 | 0.0267292723600352 | 0.162271157065456 | 1.57301286370223 | 0.0107066381156317 | FOXF1//FOXC1//DLL1//MEOX2//NEUROG1//TDRD5//LHX1//EGR2//OSR1//LRP6// |
| GO:0036503 | ERAD pathway | Biological process | 10 | 94 | 934 | 17653 | 2.01068385803453 | 0.0267292723600352 | 0.162271157065456 | 1.57301286370223 | 0.0107066381156317 | UBXN4//DNAJB9//DERL2//JKAMP//NPLOC4//WFS1//BAG6//EDEM1//ATXN3//DNAJB12// |
| GO:0007259 | JAK-STAT cascade | Biological process | 16 | 178 | 934 | 17653 | 1.69891490027188 | 0.0269518314125924 | 0.163475547841581 | 1.56941171859008 | 0.0171306209850107 | IFNA1//CNOT7//IGF1//IL2//OSM//STAT3//NF2//PPP2CA//FLRT1//NEUROD1//ASPN//SOCS5//MAPK1//PKD2//CCL2//STAT4// |
| GO:0000209 | protein polyubiquitination | Biological process | 24 | 298 | 934 | 17653 | 1.5221821421899 | 0.0270999273942289 | 0.16422653133259 | 1.56703187268013 | 0.0256959314775161 | WWP2//RNF4//TOPORS//KLHL3//UBE2W//CDC23//PLAA//HECTD2//RNF145//ZNRF2//FBXL5//RNF180//MYLIP//RLIM//ANKIB1//FBXW7//HECW2//SKP1//THOP1//LONRF3//FBXO30//CBFB//UBE3B//LONRF1// |
| GO:0034198 | cellular response to amino acid starvation | Biological process | 6 | 44 | 934 | 17653 | 2.57733112711699 | 0.0273666033907881 | 0.165545920153757 | 1.56277910175601 | 0.00642398286937901 | SESN3//SESN1//RRAGD//CDKN1A//MAP3K5//MAPK1// |
| GO:0042059 | negative regulation of epidermal growth factor receptor signaling pathway | Biological process | 6 | 44 | 934 | 17653 | 2.57733112711699 | 0.0273666033907881 | 0.165545920153757 | 1.56277910175601 | 0.00642398286937901 | ERRFI1//TSG101//SOCS5//EPS15//GRB2//EPN1// |
| GO:0045766 | positive regulation of angiogenesis | Biological process | 17 | 193 | 934 | 17653 | 1.66480456224828 | 0.0274453295624557 | 0.16587378358435 | 1.56153154985972 | 0.0182012847965739 | KDR//DLL1//ITGA5//JAK1//KLF4//ETS1//F3//GAB1//GATA6//ITGB8//STAT3//BTG1//TGFBR2//VEGFB//VASH2//ADAM12//HMGA2// |
| GO:0000186 | activation of MAPKK activity | Biological process | 7 | 56 | 934 | 17653 | 2.36255353319058 | 0.0275237768491879 | 0.16619937752773 | 1.56029197188215 | 0.00749464668094218 | MAP3K2//CRK//CRKL//MAP3K5//TAOK3//LAMTOR3//ADAM9// |
| GO:0035601 | protein deacylation | Biological process | 11 | 108 | 934 | 17653 | 1.92504361963677 | 0.0276630308869658 | 0.166891238080776 | 1.55810023834919 | 0.0117773019271949 | LYPLA1//ARID4B//MIER1//ARID4A//RBBP7//TBL1XR1//BRMS1L//PER2//REST//SPRED1//FRY// |
| GO:0031669 | cellular response to nutrient levels | Biological process | 18 | 208 | 934 | 17653 | 1.63561398451655 | 0.0277536345387436 | 0.167150420499451 | 1.5566801348202 | 0.019271948608137 | DSC2//INHBB//RRAGD//BMPR2//KLF10//PLEKHF2//FNIP1//CDKN1A//SESN3//SESN1//MAP3K5//MAPK1//PMAIP1//PRKAA1//CPEB4//JUN//PPARG//TSC1// |
| GO:0016055 | Wnt signaling pathway | Biological process | 37 | 504 | 934 | 17653 | 1.3875314401278 | 0.0277554224783208 | 0.167150420499451 | 1.55665215768153 | 0.0396145610278373 | SOX17//PPP3R1//FZD6//ESR1//MDFIC//PPP2CA//TCF7L1//CBFB//SULF1//SOX4//APCDD1//FRZB//SOSTDC1//SHISA2//FZD10//WNT7A//LRP6//TMEM64//NR4A2//PTEN//KLF4//VANGL1//CTNND2//AMER2//CCNY//LATS2//DKK3//NOG//BAMBI//CAPRIN2//TBL1XR1//HBP1//PIAS4//PRKAA1//SKP1//STRN//TGFB1I1// |
| GO:0003012 | muscle system process | Biological process | 34 | 456 | 934 | 17653 | 1.40924245839438 | 0.0278891636639452 | 0.167806418024254 | 1.55456450909643 | 0.0364025695931477 | GJC1//GJA1//GLRA1//TMOD3//HSBP1//ASPH//TMOD1//CALD1//CHRM2//EDN1//NPNT//MTPN//IGF1//TWF1//NR4A3//HAND2//JARID2//CALM2//KLF4//SULF1//GATA6//ACTC1//DRD1//NEUROG1//VEGFB//MAP2K4//SCN1A//P2RY1//DSC2//KCNJ3//ERRFI1//TRPC3//DOCK5//SGCE// |
| GO:0001890 | placenta development | Biological process | 14 | 150 | 934 | 17653 | 1.76403997144897 | 0.0279459268859472 | 0.167998492026365 | 1.55368148151143 | 0.0149892933618844 | CEBPB//PKD2//BMPR2//GJA1//GJB2//ZNF568//GRB2//ITGB8//FOSL1//HAND1//EGLN1//MAPK1//VASH2//PPARG// |
| GO:0006986 | response to unfolded protein | Biological process | 16 | 179 | 934 | 17653 | 1.68942375557762 | 0.0282042355729825 | 0.168530328196653 | 1.54968566657977 | 0.0171306209850107 | CREBRF//SERP1//DERL2//HSPA13//DNAJB9//WFS1//EDEM1//CCL2//BCL2L11//NCK1//PPP1R15B//DNAJB4//UBXN4//DNAJB5//JKAMP//HERPUD2// |
| GO:0001967 | suckling behavior | Biological process | 3 | 13 | 934 | 17653 | 4.36163729204414 | 0.0283501083809838 | 0.168530328196653 | 1.54744527648278 | 0.00321199143468951 | DERL2//POU4F1//HAND2// |
| GO:0002087 | regulation of respiratory gaseous exchange by neurological system process | Biological process | 3 | 13 | 934 | 17653 | 4.36163729204414 | 0.0283501083809838 | 0.168530328196653 | 1.54744527648278 | 0.00321199143468951 | GLRA1//PBX3//TSHZ3// |
| GO:0033127 | regulation of histone phosphorylation | Biological process | 3 | 13 | 934 | 17653 | 4.36163729204414 | 0.0283501083809838 | 0.168530328196653 | 1.54744527648278 | 0.00321199143468951 | FMR1//RPS6KA4//RPS6KA5// |
| GO:0035723 | interleukin-15-mediated signaling pathway | Biological process | 3 | 13 | 934 | 17653 | 4.36163729204414 | 0.0283501083809838 | 0.168530328196653 | 1.54744527648278 | 0.00321199143468951 | GRB2//JAK1//STAT3// |
| GO:0048681 | negative regulation of axon regeneration | Biological process | 3 | 13 | 934 | 17653 | 4.36163729204414 | 0.0283501083809838 | 0.168530328196653 | 1.54744527648278 | 0.00321199143468951 | EPHA4//RGMA//PTEN// |
| GO:0060601 | lateral sprouting from an epithelium | Biological process | 3 | 13 | 934 | 17653 | 4.36163729204414 | 0.0283501083809838 | 0.168530328196653 | 1.54744527648278 | 0.00321199143468951 | NOG//AR//SULF1// |
| GO:0071350 | cellular response to interleukin-15 | Biological process | 3 | 13 | 934 | 17653 | 4.36163729204414 | 0.0283501083809838 | 0.168530328196653 | 1.54744527648278 | 0.00321199143468951 | GRB2//JAK1//STAT3// |
| GO:0072189 | ureter development | Biological process | 3 | 13 | 934 | 17653 | 4.36163729204414 | 0.0283501083809838 | 0.168530328196653 | 1.54744527648278 | 0.00321199143468951 | OSR1//FOXF1//NFIA// |
| GO:0090110 | cargo loading into COPII-coated vesicle | Biological process | 3 | 13 | 934 | 17653 | 4.36163729204414 | 0.0283501083809838 | 0.168530328196653 | 1.54744527648278 | 0.00321199143468951 | SEC23A//TBC1D20//MIA3// |
| GO:0097154 | GABAergic neuron differentiation | Biological process | 3 | 13 | 934 | 17653 | 4.36163729204414 | 0.0283501083809838 | 0.168530328196653 | 1.54744527648278 | 0.00321199143468951 | ARX//DRD1//LHX6// |
| GO:1904861 | excitatory synapse assembly | Biological process | 3 | 13 | 934 | 17653 | 4.36163729204414 | 0.0283501083809838 | 0.168530328196653 | 1.54744527648278 | 0.00321199143468951 | PTEN//WNT7A//NPTN// |
| GO:0048665 | neuron fate specification | Biological process | 5 | 33 | 934 | 17653 | 2.86370125235222 | 0.0284081878078049 | 0.168530328196653 | 1.54655646955663 | 0.00535331905781585 | MNX1//MYT1L//DLL1//POU3F2//POU4F1// |
| GO:0060674 | placenta blood vessel development | Biological process | 5 | 33 | 934 | 17653 | 2.86370125235222 | 0.0284081878078049 | 0.168530328196653 | 1.54655646955663 | 0.00535331905781585 | MAPK1//VASH2//ITGB8//PKD2//FOSL1// |
| GO:1902742 | apoptotic process involved in development | Biological process | 5 | 33 | 934 | 17653 | 2.86370125235222 | 0.0284081878078049 | 0.168530328196653 | 1.54655646955663 | 0.00535331905781585 | HAND2//SLIT2//TGFB2//FOXC1//BCL2L11// |
| GO:0043502 | regulation of muscle adaptation | Biological process | 10 | 95 | 934 | 17653 | 1.98951876479207 | 0.0285139133418731 | 0.169009286530314 | 1.54494317461791 | 0.0107066381156317 | MTPN//EDN1//IGF1//TWF1//NR4A3//HAND2//JARID2//KLF4//ERRFI1//TRPC3// |
| GO:0032272 | negative regulation of protein polymerization | Biological process | 8 | 69 | 934 | 17653 | 2.19135400179996 | 0.0287494995645537 | 0.170256449697966 | 1.54136971056546 | 0.00856531049250535 | EPS8//TWF1//PFN2//SLIT2//STMN2//TMOD3//TMOD1//MTPN// |
| GO:0006650 | glycerophospholipid metabolic process | Biological process | 28 | 362 | 934 | 17653 | 1.46191157854887 | 0.0287939124681367 | 0.170370279984259 | 1.54069931991256 | 0.0299785867237687 | PIGA//LCLAT1//DDHD1//CDS1//ABHD3//SLC44A1//SERINC3//PIKFYVE//PIK3CB//PTEN//PLEKHA1//PLEKHA3//MTMR9//PIK3R3//LDLR//GAB1//GRB2//OSBPL5//DGKH//PLEK//EREG//ESR1//EFR3A//NRG1//MET//ZFYVE21//CHRM5//NAPEPLD// |
| GO:0042147 | retrograde transport, endosome to Golgi | Biological process | 9 | 82 | 934 | 17653 | 2.07443724865514 | 0.0289560866168853 | 0.171180081984262 | 1.53826013292841 | 0.00963597430406852 | SNX2//STX6//PIKFYVE//TMEM87A//SNX5//RAB9B//EVI5//ZFYVE21//TBC1D17// |
| GO:0198738 | cell-cell signaling by wnt | Biological process | 37 | 506 | 934 | 17653 | 1.3820471261352 | 0.0292347283088458 | 0.172676390875742 | 1.53410093789707 | 0.0396145610278373 | SOX17//APCDD1//CTNND2//AMER2//CCNY//SOSTDC1//HBP1//DKK3//LRP6//PIAS4//PRKAA1//SKP1//STRN//TGFB1I1//WNT7A//TCF7L1//PPP3R1//FZD6//ESR1//MDFIC//PPP2CA//CBFB//SULF1//SOX4//FRZB//SHISA2//FZD10//TMEM64//NR4A2//PTEN//KLF4//VANGL1//LATS2//NOG//BAMBI//CAPRIN2//TBL1XR1// |
| GO:0043666 | regulation of phosphoprotein phosphatase activity | Biological process | 11 | 109 | 934 | 17653 | 1.9073826689979 | 0.02935698174173 | 0.173140080223554 | 1.53228859726731 | 0.0117773019271949 | CRY2//PPP1R37//MASTL//URI1//CALM2//PPP1R15B//PPP1R12A//TNF//UBXN2B//PPP4R2//TSC1// |
| GO:0032874 | positive regulation of stress-activated MAPK cascade | Biological process | 14 | 151 | 934 | 17653 | 1.75235758753208 | 0.0293644347207477 | 0.173140080223554 | 1.53217835511467 | 0.0149892933618844 | MAP3K2//MDFIC//MAP3K5//MAP2K4//FZD10//EDN1//EPHA4//TAOK3//TNF//GADD45A//MINK1//WNT7A//HAND2//TGFB2// |
| GO:0097696 | STAT cascade | Biological process | 16 | 180 | 934 | 17653 | 1.68003806804663 | 0.0294993757348872 | 0.173784214368504 | 1.53018717445446 | 0.0171306209850107 | PKD2//CCL2//STAT3//STAT4//SOCS5//IFNA1//CNOT7//IGF1//IL2//OSM//NF2//PPP2CA//FLRT1//NEUROD1//ASPN//MAPK1// |
| GO:0021885 | forebrain cell migration | Biological process | 7 | 57 | 934 | 17653 | 2.32110522559074 | 0.0299978666224572 | 0.176260271040554 | 1.52290963018166 | 0.00749464668094218 | LHX6//POU3F2//ARX//SLIT2//NRG1//DRD1//EMX2// |
| GO:0030837 | negative regulation of actin filament polymerization | Biological process | 7 | 57 | 934 | 17653 | 2.32110522559074 | 0.0299978666224572 | 0.176260271040554 | 1.52290963018166 | 0.00749464668094218 | EPS8//TWF1//TMOD3//TMOD1//MTPN//PFN2//SLIT2// |
| GO:0032233 | positive regulation of actin filament bundle assembly | Biological process | 7 | 57 | 934 | 17653 | 2.32110522559074 | 0.0299978666224572 | 0.176260271040554 | 1.52290963018166 | 0.00749464668094218 | LPAR1//PPM1E//NF2//PFN2//TSC1//PPM1F//PLEK// |
| GO:0018279 | protein N-linked glycosylation via asparagine | Biological process | 6 | 45 | 934 | 17653 | 2.52005710206995 | 0.0302137320058139 | 0.177067131330433 | 1.51979562727796 | 0.00642398286937901 | ST6GALNAC3//ST6GALNAC6//ST3GAL3//ST6GAL2//ST3GAL5//MCFD2// |
| GO:0032011 | ARF protein signal transduction | Biological process | 6 | 45 | 934 | 17653 | 2.52005710206995 | 0.0302137320058139 | 0.177067131330433 | 1.51979562727796 | 0.00642398286937901 | ARAP2//APPL1//AGFG1//AGFG2//PSD//GIT2// |
| GO:0032012 | regulation of ARF protein signal transduction | Biological process | 6 | 45 | 934 | 17653 | 2.52005710206995 | 0.0302137320058139 | 0.177067131330433 | 1.51979562727796 | 0.00642398286937901 | ARAP2//APPL1//AGFG1//AGFG2//PSD//GIT2// |
| GO:0032880 | regulation of protein localization | Biological process | 64 | 956 | 934 | 17653 | 1.26530063702257 | 0.0305772730091875 | 0.179042508537779 | 1.51460124913532 | 0.0685224839400428 | TNF//RAPGEF4//MARCKS//NEUROD1//SNAP25//PER2//CLOCK//GJA1//SERP1//SOX4//CDKN1A//IGF1//MAPK1//SYBU//IPO5//MDFIC//LYPLA1//PKDCC//INHBB//REST//CTDSPL2//ARFIP1//PTGER4//TGFB2//ADAM9//IL2//WWP2//NDFIP2//EFNA5//EPHA5//DNAJC27//MIEF1//NF2//CD2AP//BCL2L11//DYNLL2//E2F1//PMAIP1//PPP3R1//YWHAG//MSN//APPL1//AR//PPFIA1//EPHA2//SQSTM1//STX6//CCNE2//NPTN//NRG1//FBXW7//ELMOD1//PRKAA1//SNIP1//DERL2//EDEM1//CCT6A//CENPQ//WNT7A//TSG101//TNFRSF21//CD274//MMD//WASL// |
| GO:0070304 | positive regulation of stress-activated protein kinase signaling cascade | Biological process | 14 | 152 | 934 | 17653 | 1.74082891919306 | 0.0308349814551234 | 0.179161021621018 | 1.51095630843242 | 0.0149892933618844 | MAP3K2//MDFIC//MAP3K5//MAP2K4//TAOK3//TGFB2//FZD10//EDN1//EPHA4//TNF//GADD45A//MINK1//WNT7A//HAND2// |
| GO:0071774 | response to fibroblast growth factor | Biological process | 14 | 152 | 934 | 17653 | 1.74082891919306 | 0.0308349814551234 | 0.179161021621018 | 1.51095630843242 | 0.0149892933618844 | SPRED1//FLRT1//GRB2//ESRP1//MAPK1//SULF1//OTX2//FAM20C//SHISA2//DSTYK//LHX1//CCL2//NPTN//NOG// |
| GO:0008064 | regulation of actin polymerization or depolymerization | Biological process | 16 | 181 | 934 | 17653 | 1.67075608977013 | 0.0308380257276761 | 0.179161021621018 | 1.5109134335725 | 0.0171306209850107 | EPS8//TWF1//PFN2//LIMA1//CFL2//PLEK//SLIT2//CDC42EP3//GRB2//NCK1//IQGAP2//ARFIP1//WASL//TMOD3//TMOD1//MTPN// |
| GO:0018126 | protein hydroxylation | Biological process | 4 | 23 | 934 | 17653 | 3.28703100269993 | 0.0308624264658415 | 0.179161021621018 | 1.51056993182768 | 0.00428265524625268 | ASPH//CRTAP//EGLN3//EGLN1// |
| GO:0021544 | subpallium development | Biological process | 4 | 23 | 934 | 17653 | 3.28703100269993 | 0.0308624264658415 | 0.179161021621018 | 1.51056993182768 | 0.00428265524625268 | DRD1//HPRT1//RARB//BCL11B// |
| GO:0021952 | central nervous system projection neuron axonogenesis | Biological process | 4 | 23 | 934 | 17653 | 3.28703100269993 | 0.0308624264658415 | 0.179161021621018 | 1.51056993182768 | 0.00428265524625268 | EPHA4//NFIB//SLIT2//NR4A2// |
| GO:0043576 | regulation of respiratory gaseous exchange | Biological process | 4 | 23 | 934 | 17653 | 3.28703100269993 | 0.0308624264658415 | 0.179161021621018 | 1.51056993182768 | 0.00428265524625268 | GLRA1//PBX3//TSHZ3//NR4A2// |
| GO:0051457 | maintenance of protein location in nucleus | Biological process | 4 | 23 | 934 | 17653 | 3.28703100269993 | 0.0308624264658415 | 0.179161021621018 | 1.51056993182768 | 0.00428265524625268 | SOX4//TOPORS//MORC3//SKP1// |
| GO:0055093 | response to hyperoxia | Biological process | 4 | 23 | 934 | 17653 | 3.28703100269993 | 0.0308624264658415 | 0.179161021621018 | 1.51056993182768 | 0.00428265524625268 | DNMT3B//PPARG//CDKN1A//BNIP3// |
| GO:1902895 | positive regulation of pri-miRNA transcription by RNA polymerase II | Biological process | 4 | 23 | 934 | 17653 | 3.28703100269993 | 0.0308624264658415 | 0.179161021621018 | 1.51056993182768 | 0.00428265524625268 | JUN//STAT3//TEAD1//TGFB2// |
| GO:0038034 | signal transduction in absence of ligand | Biological process | 8 | 70 | 934 | 17653 | 2.16004894463139 | 0.0310128547740986 | 0.179273459937331 | 1.50845825454393 | 0.00856531049250535 | BCL2L11//APPL1//MKNK2//IL2//ITGAV//MOAP1//ITM2B//TNF// |
| GO:0061180 | mammary gland epithelium development | Biological process | 8 | 70 | 934 | 17653 | 2.16004894463139 | 0.0310128547740986 | 0.179273459937331 | 1.50845825454393 | 0.00856531049250535 | CEBPB//EPHA2//MAPK1//LRP6//AR//SOSTDC1//ESR1//FOXF1// |
| GO:0097192 | extrinsic apoptotic signaling pathway in absence of ligand | Biological process | 8 | 70 | 934 | 17653 | 2.16004894463139 | 0.0310128547740986 | 0.179273459937331 | 1.50845825454393 | 0.00856531049250535 | TNF//BCL2L11//APPL1//MKNK2//IL2//ITGAV//MOAP1//ITM2B// |
| GO:0045844 | positive regulation of striated muscle tissue development | Biological process | 9 | 83 | 934 | 17653 | 2.04944402879183 | 0.0310143350771369 | 0.179273459937331 | 1.50843752533009 | 0.00963597430406852 | DLL1//PRKAA1//SOX17//ZFPM2//GATA6//EDN1//IGF1//NRG1//GJA1// |
| GO:0048636 | positive regulation of muscle organ development | Biological process | 9 | 83 | 934 | 17653 | 2.04944402879183 | 0.0310143350771369 | 0.179273459937331 | 1.50843752533009 | 0.00963597430406852 | GJA1//DLL1//PRKAA1//SOX17//ZFPM2//GATA6//EDN1//IGF1//NRG1// |
| GO:0051049 | regulation of transport | Biological process | 114 | 1821 | 934 | 17653 | 1.18322285682032 | 0.0310870624098012 | 0.179540395454727 | 1.50742031492977 | 0.122055674518201 | ARL6IP1//FMR1//NTF3//TNF//EDN1//RAPGEF4//MARCKS//NEUROD1//SNAP25//PER2//CLOCK//TRPC3//P2RY1//NR4A3//CALM2//SLC6A1//IGF1//ADIPOR2//INHBB//SOX11//RAB21//NSF//SYT6//TRPV6//GJA1//RUFY2//TBC1D12//EVI5//TBC1D17//USP6NL//PKD2//SERP1//SOX4//STIM2//ITGAV//WWP2//NDFIP2//CDKN1A//MAPK1//CLIC4//KCNA4//KCNJ3//KCNJ10//KCNK10//HCN2//SCN1A//SYBU//IPO5//MDFIC//LYPLA1//PKDCC//DRD1//PXK//FOXF1//DLL1//LDLR//REST//APPL1//PPP1R12A//CTDSPL2//OSM//CD2AP//ARFIP1//PTGER4//TGFB2//ADAM9//PTEN//IL2//NRG1//LPAR3//WFS1//PPARG//PRKAA1//EFNA5//EPHA5//RAB3GAP1//DNAJC27//CCL2//MIEF1//BCL2L11//DYNLL2//E2F1//PMAIP1//PPP3R1//YWHAG//AHNAK//GNB5//NEFH//TBC1D20//PLAA//TSG101//FBXW7//ELMOD1//SNIP1//DERL2//EDEM1//SESTD1//OSR1//NEUROG1//CRY2//PFN2//MINK1//PIK3CB//WASL//CHMP3//MSN//HECW2//GLRX//TNFRSF21//MYB//TMF1//ARHGAP1//CD274//PIK3R3// |
| GO:0030879 | mammary gland development | Biological process | 13 | 138 | 934 | 17653 | 1.78047512646246 | 0.0312347570436505 | 0.180239472599154 | 1.50536186781984 | 0.0139186295503212 | NME1//CEBPB//EPHA2//MAPK1//GJA1//TGFBR2//LRP6//AR//SOSTDC1//ESR1//FOXF1//BCL2L11//NRG1// |
| GO:0046330 | positive regulation of JNK cascade | Biological process | 12 | 124 | 934 | 17653 | 1.82907370311529 | 0.0313639244325197 | 0.180830537883317 | 1.50356960119315 | 0.012847965738758 | MAP3K2//MDFIC//MAP3K5//MAP2K4//FZD10//EDN1//EPHA4//TAOK3//TNF//GADD45A//MINK1//WNT7A// |
| GO:0048588 | developmental cell growth | Biological process | 18 | 211 | 934 | 17653 | 1.61235880938125 | 0.0313954148637007 | 0.180857913733567 | 1.50313377373557 | 0.019271948608137 | TGFBR2//DPYSL2//RAB21//EPHA4//GOLGA4//NRG1//EFNA5//EPHA7//ULK2//LPAR3//BMPR2//SEMA4B//SLIT2//MAP2K4//EDN1//IGF1//PLAA//FLRT1// |
| GO:0010821 | regulation of mitochondrion organization | Biological process | 19 | 226 | 934 | 17653 | 1.5889740577211 | 0.0314238884468181 | 0.18086787877943 | 1.5027400756134 | 0.0203426124197002 | BNIP3//TMEM135//KDR//MIEF1//DDHD1//BCL2L11//PMAIP1//MOAP1//IGF1//DYNLL2//E2F1//PPP3R1//YWHAG//FBXW7//NRG1//ELMOD1//PRKAA1//SNIP1//SQSTM1// |
| GO:0071705 | nitrogen compound transport | Biological process | 139 | 2264 | 934 | 17653 | 1.16040615234449 | 0.0317507365345055 | 0.182593733999031 | 1.49824619575275 | 0.148822269807281 | RANBP6//IPO5//TNF//DRD1//STAT3//EDN1//ARL6IP1//SLC6A14//SLC32A1//SLC6A6//ZFAND6//SRSF2//NUP35//AGFG1//NUP43//TSC1//PAN3//YWHAG//EGR2//ZDHHC1//RPL15//MTX3//IMMP2L//SLC17A7//SLC17A6//STX6//SEC23A//RAB12//STX2//RAB21//TBC1D12//RAB38//SNX5//ARFIP1//RAB30//NSF//SAR1B//RAB9B//DNAJC27//RAB23//TMED9//CTSA//RAB22A//KIF13A//SNX2//EVI5//TBC1D17//AP1S2//RAB28//ZFYVE9//USP6NL//RAPGEF4//MARCKS//NEUROD1//SNAP25//PER2//CLOCK//CBLN4//MIA3//P2RY1//PPFIA1//SLC6A1//TRIM3//EXOC5//VPS37A//DENND1B//LCA5//EPS15//NECAP1//SERP1//CHMP3//ATG16L1//UEVLD//ANKRD50//ZMAT3//AKTIP//BTF3//TSG101//PLEKHF2//FAM160A2//C5ORF30//MCFD2//PKDCC//ARRDC4//CEP41//SLC35D1//SERINC3//SLC14A1//GJA1//SLC44A1//CHRM5//SLC25A32//GNPTG//PEX5L//DERL2//NPLOC4//SOX4//CDKN1A//IGF1//MAPK1//ARHGAP1//VLDLR//SYBU//MCOLN2//ITGAV//MDFIC//PPARG//LYPLA1//GOLGA4//C16ORF70//CLIP1//INHBB//REST//CTDSPL2//PTGER4//TGFB2//ADAM9//IL2//FMR1//QKI//G3BP2//WWP2//NDFIP2//KCNJ10//EFNA5//EPHA5//RAB3GAP1//PLEK//MIEF1//NRG1//FBXW7//ELMOD1//PRKAA1//SNIP1//EDEM1//OSR1//TGFBR2//TNFRSF21//CD274// |
| GO:0007435 | salivary gland morphogenesis | Biological process | 5 | 34 | 934 | 17653 | 2.77947474493009 | 0.0319039317932742 | 0.182697959964364 | 1.49615579183623 | 0.00535331905781585 | NFIB//TNF//NTN4//BTBD7//TGFB2// |
| GO:0007616 | long-term memory | Biological process | 5 | 34 | 934 | 17653 | 2.77947474493009 | 0.0319039317932742 | 0.182697959964364 | 1.49615579183623 | 0.00535331905781585 | CPEB3//GRIA1//LDLR//SLC17A7//CCND2// |
| GO:0046621 | negative regulation of organ growth | Biological process | 5 | 34 | 934 | 17653 | 2.77947474493009 | 0.0319039317932742 | 0.182697959964364 | 1.49615579183623 | 0.00535331905781585 | GJA1//JARID2//PTEN//TGFBR2//NOG// |
| GO:0051443 | positive regulation of ubiquitin-protein transferase activity | Biological process | 5 | 34 | 934 | 17653 | 2.77947474493009 | 0.0319039317932742 | 0.182697959964364 | 1.49615579183623 | 0.00535331905781585 | PTEN//TOPORS//FBXW7//ARRDC3//ARRDC4// |
| GO:0051482 | positive regulation of cytosolic calcium ion concentration involved in phospholipase C-activating G-protein coupled signaling pathway | Biological process | 5 | 34 | 934 | 17653 | 2.77947474493009 | 0.0319039317932742 | 0.182697959964364 | 1.49615579183623 | 0.00535331905781585 | DRD1//S1PR1//LPAR1//EDN1//LPAR3// |
| GO:0090130 | tissue migration | Biological process | 26 | 334 | 934 | 17653 | 1.47129082306479 | 0.0319491940587707 | 0.182802368375183 | 1.49554009275576 | 0.0278372591006424 | KDR//SLIT2//EDN1//ETS1//BMPR2//WNT7A//NANOS1//DUSP10//PFN2//PTPRG//JUN//TGFB2//TGFBR2//DOCK5//PPM1F//EFNA1//EPHA2//PPARG//PTEN//ADAM9//SPRED1//MEOX2//KLF4//FOXF1//ACTC1//MET// |
| GO:0030832 | regulation of actin filament length | Biological process | 16 | 182 | 934 | 17653 | 1.66157611125491 | 0.03222094827242 | 0.184201414341823 | 1.49186168230851 | 0.0171306209850107 | EPS8//TWF1//PFN2//LIMA1//CFL2//PLEK//SLIT2//CDC42EP3//GRB2//NCK1//IQGAP2//ARFIP1//WASL//TMOD3//TMOD1//MTPN// |
| GO:0001657 | ureteric bud development | Biological process | 10 | 97 | 934 | 17653 | 1.94849775933244 | 0.0323339777712259 | 0.18453560478211 | 1.49034086458646 | 0.0107066381156317 | NPNT//LHX1//PKD2//TCF21//NOG//GDF11//OSR1//FOXC1//RARB//SLIT2// |
| GO:0048010 | vascular endothelial growth factor receptor signaling pathway | Biological process | 10 | 97 | 934 | 17653 | 1.94849775933244 | 0.0323339777712259 | 0.18453560478211 | 1.49034086458646 | 0.0107066381156317 | ITGA5//KDR//CRK//FOXC1//SULF1//ITGAV//NCK1//PIK3CB//BMPR2//VEGFB// |
| GO:0045765 | regulation of angiogenesis | Biological process | 28 | 366 | 934 | 17653 | 1.44593440282702 | 0.0325167055038787 | 0.185421989310904 | 1.48789346227738 | 0.0299785867237687 | SPRED1//FOXC1//SULF1//PPARG//TGFB2//KLF4//ETS1//F3//GAB1//GATA6//ITGB8//KDR//STAT3//BTG1//TGFBR2//VEGFB//VASH2//ADAM12//HMGA2//MEOX2//KLF2//EPN1//DLL1//ITGA5//JAK1//EFNA1//EPHA2//EGLN1// |
| GO:0046847 | filopodium assembly | Biological process | 7 | 58 | 934 | 17653 | 2.28108616997711 | 0.0326173504830336 | 0.185682778886158 | 1.48655131973141 | 0.00749464668094218 | FMR1//GPM6A//WASL//FGD4//FGD5//ITGA6//ARHGEF4// |
| GO:1905207 | regulation of cardiocyte differentiation | Biological process | 7 | 58 | 934 | 17653 | 2.28108616997711 | 0.0326173504830336 | 0.185682778886158 | 1.48655131973141 | 0.00749464668094218 | GATA6//TGFB2//EDN1//IGF1//SOX17//DLL1//NRG1// |
| GO:0048609 | multicellular organismal reproductive process | Biological process | 55 | 808 | 934 | 17653 | 1.28653905272754 | 0.0327978083878965 | 0.186553051410718 | 1.48415517570427 | 0.0588865096359743 | ESR1//EREG//PLEKHA1//YTHDF2//TBC1D20//AGFG1//TMF1//BMPR2//MYBL1//SYCP3//MASTL//DZIP1//RNF2//ACVR1//BCL2L11//CRTAP//DDX6//E2F1//GOLGA3//AR//CLDN11//ACOX1//PAIP2//ARID4B//ARID4A//HERPUD2//SOX17//BTG1//BAG6//IMMP2L//ADAM18//CLOCK//TDRD5//QKI//TOB2//SOS1//FOSL1//DRD1//NHLH2//THRA//FOXC1//KMT2B//TGFB2//CREBRF//GNAQ//PTEN//ETS1//GJA1//GJB2//INHBB//EDN1//DMRTA1//IGF1//P2RY1//SLIT2// |
| GO:1901863 | positive regulation of muscle tissue development | Biological process | 9 | 84 | 934 | 17653 | 2.02504588559192 | 0.0331722119783197 | 0.187849483138765 | 1.47922556803588 | 0.00963597430406852 | GJA1//DLL1//PRKAA1//SOX17//ZFPM2//GATA6//EDN1//IGF1//NRG1// |
| GO:0050808 | synapse organization | Biological process | 24 | 304 | 934 | 17653 | 1.49213907359405 | 0.0331800622411323 | 0.187849483138765 | 1.4791228036355 | 0.0256959314775161 | DRD1//GPM6A//NRG1//POU4F1//PTEN//EPHA7//EFNA5//SLITRK3//FLRT1//WNT7A//NFIA//SYBU//CTNND2//WASL//EPHA4//EFNA1//CAPRIN2//LRP8//GLRB//ZNF804A//NPTN//FMR1//FXR1//TSC1// |
| GO:0044248 | cellular catabolic process | Biological process | 138 | 2250 | 934 | 17653 | 1.15922626695218 | 0.0332380952213005 | 0.187849483138765 | 1.4783638723302 | 0.147751605995717 | UBXN2B//TRAPPC8//RAB23//ATG16L1//ULK2//DCP2//PPP2CA//RPL15//ZFAND4//CNOT6L//PAN3//CNOT7//CNOT6//BNIP3//SQSTM1//NUP35//NUP43//PDE10A//DCTPP1//CTSA//TOPORS//USP24//KLHL3//MYLIP//USP27X//ATXN3//RLIM//NPLOC4//USP28//USP46//UBE2I//BAG6//RNF128//KLHL15//USP45//CDC23//USP3//UBE2W//GLUD2//BCAT2//UGT1A4//UGT1A1//RAB12//NRBF2//PIK3CB//TBC1D17//TECPR2//TMF1//ACBD5//NAPEPLD//PMAIP1//RRAGD//MET//MTMR9//PRKAA1//LDLR//VPS37A//DYNLL2//CHMP3//TSG101//PLEKHF2//PLAA//SESN3//SESN1//KDR//TSC1//SNX5//RHEB//LAMTOR3//NAGK//PON2//UBXN4//DNAJB9//DERL2//JKAMP//WFS1//EDEM1//TGFB1I1//EGLN1//FBXL5//FBXW7//SKP1//BAG5//RNF180//ANKIB1//LONRF3//LONRF1//SOCS5//VLDLR//ACOX1//WDFY3//ACOT7//DNAJB12//ACSL1//GNS//OGN//SLC44A1//WWP2//RNF38//RNF145//ZNRF2//CD2AP//SPOPL//HECW2//RNF4//TBL1XR1//CLOCK//MTRR//FMR1//YTHDF2//FXR1//DDX6//STAT3//IGF1//HPRT1//ENPP4//E2F1//SH3D19//TNF//ADAM9//ABHD3//TOB1//CPEB3//BTG2//IDH1//QKI//PFKP//CALCOCO2//NANOS1//FYCO1//FEZ2//EFNA1//EPHA4//MSN//PTEN//PIKFYVE//TBC1D12//RAB3GAP1// |
| GO:0000060 | protein import into nucleus, translocation | Biological process | 6 | 46 | 934 | 17653 | 2.46527325202495 | 0.0332479419365817 | 0.187849483138765 | 1.47823523256385 | 0.00642398286937901 | CDKN1A//IGF1//MAPK1//RANBP6//IPO5//TNF// |
| GO:0018196 | peptidyl-asparagine modification | Biological process | 6 | 46 | 934 | 17653 | 2.46527325202495 | 0.0332479419365817 | 0.187849483138765 | 1.47823523256385 | 0.00642398286937901 | ST6GALNAC3//ST6GALNAC6//ST3GAL3//ST6GAL2//ST3GAL5//MCFD2// |
| GO:0045023 | G0 to G1 transition | Biological process | 6 | 46 | 934 | 17653 | 2.46527325202495 | 0.0332479419365817 | 0.187849483138765 | 1.47823523256385 | 0.00642398286937901 | E2F1//RBBP7//RBBP8//RNF2//RRM2//MDM4// |
| GO:0048806 | genitalia development | Biological process | 6 | 46 | 934 | 17653 | 2.46527325202495 | 0.0332479419365817 | 0.187849483138765 | 1.47823523256385 | 0.00642398286937901 | NEUROG1//LRP6//AR//ESR1//LHX1//FOXF2// |
| GO:0060443 | mammary gland morphogenesis | Biological process | 6 | 46 | 934 | 17653 | 2.46527325202495 | 0.0332479419365817 | 0.187849483138765 | 1.47823523256385 | 0.00642398286937901 | EPHA2//LRP6//AR//SOSTDC1//ESR1//TGFBR2// |
| GO:0009991 | response to extracellular stimulus | Biological process | 36 | 495 | 934 | 17653 | 1.37457660112906 | 0.0333868740881307 | 0.188367042068493 | 1.4764242407188 | 0.0385438972162741 | TBL1XR1//ADRB1//ACSL1//UGT1A1//PPARG//TGFBR2//ADIPOR2//DSC2//INHBB//RRAGD//BMPR2//KLF10//PLEKHF2//FNIP1//IGF1//CDKN1A//ITGA6//NR4A2//FOSL1//PRKAA1//TMEM135//DNMT3B//SRSF2//PTEN//TRPC3//SESN3//SESN1//MAP3K5//MAPK1//PMAIP1//CPEB4//DCTPP1//ULK2//JUN//LDLR//TSC1// |
| GO:0031644 | regulation of neurological system process | Biological process | 8 | 71 | 934 | 17653 | 2.12962572005911 | 0.0333952511370875 | 0.188367042068493 | 1.47631528623005 | 0.00856531049250535 | TNFRSF21//PTEN//WASF3//NRG1//EDN1//TMEM100//GLRA1//FMR1// |
| GO:0016567 | protein ubiquitination | Biological process | 56 | 827 | 934 | 17653 | 1.27983552946721 | 0.0342546988531813 | 0.192802156892809 | 1.46527984618507 | 0.0599571734475375 | TOPORS//WWP2//HECTD2//RNF145//ZNRF2//FBXL5//RNF180//MYLIP//RLIM//ANKIB1//UBE2W//FBXW7//HECW2//SKP1//THOP1//LONRF3//FBXO30//CBFB//UBE3B//LONRF1//TSG101//KBTBD8//SPOPL//SOX4//PER2//BAG5//NDFIP2//WFS1//RNF2//PCGF5//ARRDC3//ARRDC4//RNF141//RNF4//KLHL3//CDC23//PLAA//PTEN//TRIM3//KLHL2//RNF38//MYCBP2//FBXO33//MDM4//DCAF8//DCAF6//FEM1C//KLHL14//MARCH4//AKTIP//MARCH7//UBE2Z//RNF128//KLHL15//ZFAND4//SOCS5// |
| GO:0046907 | intracellular transport | Biological process | 110 | 1758 | 934 | 17653 | 1.18262065370177 | 0.0342768569232759 | 0.192802156892809 | 1.46499900832001 | 0.117773019271949 | RANBP6//IPO5//TNF//DRD1//STAT3//ZFAND6//SRSF2//NUP35//AGFG1//NUP43//TSC1//PAN3//YWHAG//EGR2//ZDHHC1//ARL6IP1//RPL15//MTX3//IMMP2L//MID1IP1//STX6//SEC23A//RAB12//STX2//RAB21//TBC1D12//RAB38//SNX5//ARFIP1//RAB30//NSF//SAR1B//RAB9B//DNAJC27//RAB23//TMED9//CTSA//RAB22A//KIF13A//SNX2//EVI5//TBC1D17//AP1S2//RAB28//ZFYVE9//USP6NL//CNIH1//DCTN6//TBC1D20//DYNLL2//DYNC1LI2//TRAPPC8//GRIA1//MIA3//IER3IP1//MCFD2//EPS15//NEUROD1//ANP32E//FMR1//FXR1//AKTIP//TSG101//FAM160A2//SNAP25//VPS37A//ARHGAP1//CHMP3//DPY30//SQSTM1//GNPTG//PEX5L//SYBU//WASL//KIF5A//DERL2//NPLOC4//DENND1B//CDKN1A//IGF1//MAPK1//PIKFYVE//TMEM87A//ZFYVE21//MDFIC//NR4A3//FOXF1//CLIP1//PPP1R12A//SOX4//CTDSPL2//PPP6C//SYT6//ATXN1//FYCO1//KIF23//LDLR//LRP6//MIEF1//NEFH//RAB3GAP1//NRG1//FBXW7//ELMOD1//PRKAA1//SNIP1//EDEM1//ANKRD50//PFN2//MSN// |
| GO:0006476 | protein deacetylation | Biological process | 10 | 98 | 934 | 17653 | 1.92861512913517 | 0.0343727931148324 | 0.192802156892809 | 1.46378517589093 | 0.0107066381156317 | ARID4B//MIER1//ARID4A//RBBP7//TBL1XR1//BRMS1L//PER2//REST//SPRED1//FRY// |
| GO:0072163 | mesonephric epithelium development | Biological process | 10 | 98 | 934 | 17653 | 1.92861512913517 | 0.0343727931148324 | 0.192802156892809 | 1.46378517589093 | 0.0107066381156317 | GDF11//OSR1//FOXC1//NPNT//LHX1//RARB//TCF21//SLIT2//PKD2//NOG// |
| GO:0072164 | mesonephric tubule development | Biological process | 10 | 98 | 934 | 17653 | 1.92861512913517 | 0.0343727931148324 | 0.192802156892809 | 1.46378517589093 | 0.0107066381156317 | GDF11//OSR1//FOXC1//NPNT//LHX1//RARB//TCF21//SLIT2//PKD2//NOG// |
| GO:0061008 | hepaticobiliary system development | Biological process | 13 | 140 | 934 | 17653 | 1.755039767513 | 0.0345442431601451 | 0.192802156892809 | 1.46162431795978 | 0.0139186295503212 | GATA6//JARID2//JUN//MAN2A1//MET//PKD2//UGT1A1//RARB//ARID5B//SOX17//FRZB//CEBPB//FBXW7// |
| GO:0031570 | DNA integrity checkpoint | Biological process | 15 | 169 | 934 | 17653 | 1.67755280463236 | 0.0346696206378188 | 0.192802156892809 | 1.46005091045792 | 0.0160599571734475 | E2F1//USP28//CLOCK//CDKN1A//GADD45A//CNOT6L//CNOT7//MDM4//CNOT6//RBL2//SOX4//BTG2//TAOK3//HMGA2//RBBP8// |
| GO:0001675 | acrosome assembly | Biological process | 3 | 14 | 934 | 17653 | 4.05009177118385 | 0.0346946953923627 | 0.192802156892809 | 1.45973692111495 | 0.00321199143468951 | TBC1D20//AGFG1//TMF1// |
| GO:0003214 | cardiac left ventricle morphogenesis | Biological process | 3 | 14 | 934 | 17653 | 4.05009177118385 | 0.0346946953923627 | 0.192802156892809 | 1.45973692111495 | 0.00321199143468951 | HAND1//FOXF1//TGFBR2// |
| GO:0007175 | negative regulation of epidermal growth factor-activated receptor activity | Biological process | 3 | 14 | 934 | 17653 | 4.05009177118385 | 0.0346946953923627 | 0.192802156892809 | 1.45973692111495 | 0.00321199143468951 | ERRFI1//TSG101//SOCS5// |
| GO:0007183 | SMAD protein complex assembly | Biological process | 3 | 14 | 934 | 17653 | 4.05009177118385 | 0.0346946953923627 | 0.192802156892809 | 1.45973692111495 | 0.00321199143468951 | PMEPA1//LDLRAD4//ZFYVE9// |
| GO:0007512 | adult heart development | Biological process | 3 | 14 | 934 | 17653 | 4.05009177118385 | 0.0346946953923627 | 0.192802156892809 | 1.45973692111495 | 0.00321199143468951 | GJA1//MEF2D//HAND2// |
| GO:0019511 | peptidyl-proline hydroxylation | Biological process | 3 | 14 | 934 | 17653 | 4.05009177118385 | 0.0346946953923627 | 0.192802156892809 | 1.45973692111495 | 0.00321199143468951 | CRTAP//EGLN3//EGLN1// |
| GO:0034349 | glial cell apoptotic process | Biological process | 3 | 14 | 934 | 17653 | 4.05009177118385 | 0.0346946953923627 | 0.192802156892809 | 1.45973692111495 | 0.00321199143468951 | DLL1//CCL2//TNFRSF21// |
| GO:0035112 | genitalia morphogenesis | Biological process | 3 | 14 | 934 | 17653 | 4.05009177118385 | 0.0346946953923627 | 0.192802156892809 | 1.45973692111495 | 0.00321199143468951 | LRP6//AR//NEUROG1// |
| GO:0051770 | positive regulation of nitric-oxide synthase biosynthetic process | Biological process | 3 | 14 | 934 | 17653 | 4.05009177118385 | 0.0346946953923627 | 0.192802156892809 | 1.45973692111495 | 0.00321199143468951 | KDR//CCL2//MAP2K4// |
| GO:0070672 | response to interleukin-15 | Biological process | 3 | 14 | 934 | 17653 | 4.05009177118385 | 0.0346946953923627 | 0.192802156892809 | 1.45973692111495 | 0.00321199143468951 | GRB2//JAK1//STAT3// |
| GO:1904294 | positive regulation of ERAD pathway | Biological process | 3 | 14 | 934 | 17653 | 4.05009177118385 | 0.0346946953923627 | 0.192802156892809 | 1.45973692111495 | 0.00321199143468951 | EDEM1//ATXN3//BAG6// |
| GO:0006664 | glycolipid metabolic process | Biological process | 12 | 126 | 934 | 17653 | 1.80004078719282 | 0.0348934196686595 | 0.193588348826205 | 1.45725646619385 | 0.012847965738758 | ITGB8//ST6GALNAC3//ST6GALNAC6//ST8SIA3//ST3GAL5//PIGA//B3GALT2//CTSA//ST8SIA5//B3GNT5//PRKAA1//KDELC1// |
| GO:1903509 | liposaccharide metabolic process | Biological process | 12 | 126 | 934 | 17653 | 1.80004078719282 | 0.0348934196686595 | 0.193588348826205 | 1.45725646619385 | 0.012847965738758 | ITGB8//ST6GALNAC3//ST6GALNAC6//ST8SIA3//ST3GAL5//PIGA//KDELC1//B3GALT2//CTSA//ST8SIA5//B3GNT5//PRKAA1// |
| GO:0045637 | regulation of myeloid cell differentiation | Biological process | 20 | 244 | 934 | 17653 | 1.54921543160038 | 0.0350160602079125 | 0.194109520644354 | 1.45573271965226 | 0.0214132762312634 | NME1//CDK6//DLL1//HMGB3//ETS1//STAT3//NFE2//NR4A3//DPY30//CBFB//KAT2B//KMT2B//JUN//CEBPB//TOB2//TMEM64//POU4F1//KLF10//TNF//FBXW7// |
| GO:0001947 | heart looping | Biological process | 7 | 59 | 934 | 17653 | 2.24242369251987 | 0.0353851497980805 | 0.194890081655567 | 1.45117896152991 | 0.00749464668094218 | GJA1//DLL1//PKD2//SOX17//TGFBR2//HAND1//HAND2// |
| GO:1904705 | regulation of vascular smooth muscle cell proliferation | Biological process | 7 | 59 | 934 | 17653 | 2.24242369251987 | 0.0353851497980805 | 0.194890081655567 | 1.45117896152991 | 0.00749464668094218 | PPARG//IGF1//JUN//MEF2D//MAP3K5//MMP2//TNF// |
| GO:1990874 | vascular smooth muscle cell proliferation | Biological process | 7 | 59 | 934 | 17653 | 2.24242369251987 | 0.0353851497980805 | 0.194890081655567 | 1.45117896152991 | 0.00749464668094218 | PPARG//IGF1//JUN//MEF2D//MAP3K5//MMP2//TNF// |
| GO:0032410 | negative regulation of transporter activity | Biological process | 9 | 85 | 934 | 17653 | 2.00122181634967 | 0.0354316634823416 | 0.194890081655567 | 1.45060845748216 | 0.00963597430406852 | PKD2//CALM2//PTEN//GNB5//FMR1//OSR1//HECW2//WWP2//NDFIP2// |
| GO:0061053 | somite development | Biological process | 9 | 85 | 934 | 17653 | 2.00122181634967 | 0.0354316634823416 | 0.194890081655567 | 1.45060845748216 | 0.00963597430406852 | FOXF1//FOXC1//DLL1//MEOX2//LHX1//LRP6//FRZB//SOX11//NOG// |
| GO:0030728 | ovulation | Biological process | 4 | 24 | 934 | 17653 | 3.15007137758744 | 0.0355314905635538 | 0.194890081655567 | 1.4493865732608 | 0.00428265524625268 | INHBB//EREG//IMMP2L//KMT2B// |
| GO:0032800 | receptor biosynthetic process | Biological process | 4 | 24 | 934 | 17653 | 3.15007137758744 | 0.0355314905635538 | 0.194890081655567 | 1.4493865732608 | 0.00428265524625268 | EDN1//PPARG//ITGAV//TNF// |
| GO:0033233 | regulation of protein sumoylation | Biological process | 4 | 24 | 934 | 17653 | 3.15007137758744 | 0.0355314905635538 | 0.194890081655567 | 1.4493865732608 | 0.00428265524625268 | HMG20A//RNF4//PIAS4//UBE2I// |
| GO:0045662 | negative regulation of myoblast differentiation | Biological process | 4 | 24 | 934 | 17653 | 3.15007137758744 | 0.0355314905635538 | 0.194890081655567 | 1.4493865732608 | 0.00428265524625268 | SOSTDC1//DLL1//TNF//CXCL14// |
| GO:0045672 | positive regulation of osteoclast differentiation | Biological process | 4 | 24 | 934 | 17653 | 3.15007137758744 | 0.0355314905635538 | 0.194890081655567 | 1.4493865732608 | 0.00428265524625268 | TMEM64//POU4F1//KLF10//TNF// |
| GO:0050779 | RNA destabilization | Biological process | 4 | 24 | 934 | 17653 | 3.15007137758744 | 0.0355314905635538 | 0.194890081655567 | 1.4493865732608 | 0.00428265524625268 | CNOT6L//YTHDF2//CPEB3//QKI// |
| GO:0051220 | cytoplasmic sequestering of protein | Biological process | 4 | 24 | 934 | 17653 | 3.15007137758744 | 0.0355314905635538 | 0.194890081655567 | 1.4493865732608 | 0.00428265524625268 | PKD2//THRA//G3BP2//DZIP1// |
| GO:0060444 | branching involved in mammary gland duct morphogenesis | Biological process | 4 | 24 | 934 | 17653 | 3.15007137758744 | 0.0355314905635538 | 0.194890081655567 | 1.4493865732608 | 0.00428265524625268 | AR//ESR1//EPHA2//LRP6// |
| GO:0031641 | regulation of myelination | Biological process | 5 | 35 | 934 | 17653 | 2.70006118078923 | 0.0356568781996053 | 0.1952611070963 | 1.44785668247399 | 0.00535331905781585 | TNFRSF21//PTEN//WASF3//NRG1//RARB// |
| GO:0046329 | negative regulation of JNK cascade | Biological process | 5 | 35 | 934 | 17653 | 2.70006118078923 | 0.0356568781996053 | 0.1952611070963 | 1.44785668247399 | 0.00535331905781585 | HIPK3//DUSP10//AIDA//ZMYND11//TAOK3// |
| GO:0048844 | artery morphogenesis | Biological process | 8 | 72 | 934 | 17653 | 2.10004758505829 | 0.0358990234267142 | 0.196428070740185 | 1.44491736552079 | 0.00856531049250535 | SOX4//TGFB2//HAND2//PKD2//BMPR2//NOG//FOXF1//FOXC1// |
| GO:0001818 | negative regulation of cytokine production | Biological process | 20 | 245 | 934 | 17653 | 1.54289210330813 | 0.0363159844761666 | 0.198548911085137 | 1.4399021782196 | 0.0214132762312634 | TNF//TGFB2//NPLOC4//CD274//PDCD1LG2//ERRFI1//DLL1//CMKLR1//KLF2//SOCS5//CD2AP//GATA6//RNF128//KLF4//INHBB//PTGER4//C5ORF30//TNFRSF21//RPS6KA4//RPS6KA5// |
| GO:0050732 | negative regulation of peptidyl-tyrosine phosphorylation | Biological process | 6 | 47 | 934 | 17653 | 2.41282062964144 | 0.0364727527754286 | 0.199047818387349 | 1.43803145713969 | 0.00642398286937901 | ERRFI1//TSG101//SOCS5//NF2//PPP2CA//NTF3// |
| GO:1901028 | regulation of mitochondrial outer membrane permeabilization involved in apoptotic signaling pathway | Biological process | 6 | 47 | 934 | 17653 | 2.41282062964144 | 0.0364727527754286 | 0.199047818387349 | 1.43803145713969 | 0.00642398286937901 | BCL2L11//DYNLL2//E2F1//PMAIP1//PPP3R1//YWHAG// |
| GO:1901184 | regulation of ERBB signaling pathway | Biological process | 10 | 99 | 934 | 17653 | 1.90913416823481 | 0.0364996168194609 | 0.199047818387349 | 1.43771169483304 | 0.0107066381156317 | EREG//FBXW7//ERRFI1//TSG101//SOCS5//EPS15//GRB2//EPN1//SOS1//NRG1// |
| GO:0016570 | histone modification | Biological process | 33 | 450 | 934 | 17653 | 1.38603140613847 | 0.0365543975213792 | 0.199047818387349 | 1.43706036961206 | 0.0353319057815846 | RPS6KA4//RPS6KA5//EPC2//CLOCK//ARID4B//MIER1//ARID4A//RBBP7//TBL1XR1//BRMS1L//PPM1F//ARID5B//USP3//JARID2//FMR1//PRDM16//PRKAA1//RNF2//SKP1//HMGA2//PCGF5//TADA1//DR1//KAT2B//DRD1//DPY30//KMT2B//DNMT3B//MYB//KDM2A//EZH1//PER2//REST// |
| GO:0046486 | glycerolipid metabolic process | Biological process | 33 | 450 | 934 | 17653 | 1.38603140613847 | 0.0365543975213792 | 0.199047818387349 | 1.43706036961206 | 0.0353319057815846 | PIGA//NKX2-3//ACSL1//LCLAT1//DDHD1//CDS1//ABHD3//SLC44A1//SERINC3//PIKFYVE//PIK3CB//PTEN//PLEKHA1//PLEKHA3//MTMR9//PIK3R3//CNEP1R1//LDLR//FBXW7//GAB1//GRB2//OSBPL5//DGKH//PLEK//EREG//ESR1//EFR3A//NRG1//MET//ZFYVE21//CHRM5//NAPEPLD//TBL1XR1// |
| GO:0048511 | rhythmic process | Biological process | 22 | 276 | 934 | 17653 | 1.50655587623747 | 0.0366645942347443 | 0.199487249243424 | 1.43575311695738 | 0.0235546038543897 | ESR1//EREG//PLEKHA1//EGR2//CRY2//JUN//NPAS2//KLF10//PER2//CLOCK//GNAQ//BHLHE41//BHLHE40//NHLH2//TGFB2//PPARG//PRKAA1//ETS1//PTEN//SLIT2//TEF//KAT2B// |
| GO:1904018 | positive regulation of vasculature development | Biological process | 18 | 215 | 934 | 17653 | 1.58236143618346 | 0.0367883453026281 | 0.199999661801989 | 1.43428974579483 | 0.019271948608137 | ETS1//F3//GAB1//GATA6//ITGB8//KDR//STAT3//BTG1//TGFBR2//VEGFB//VASH2//ADAM12//HMGA2//DLL1//ITGA5//JAK1//KLF4//TMEM100// |
| GO:1903749 | positive regulation of establishment of protein localization to mitochondrion | Biological process | 11 | 113 | 934 | 17653 | 1.8398646984139 | 0.0368768766945305 | 0.200319933401695 | 1.43324586884864 | 0.0117773019271949 | BCL2L11//DYNLL2//E2F1//PMAIP1//PPP3R1//YWHAG//NRG1//FBXW7//ELMOD1//PRKAA1//SNIP1// |
| GO:0002521 | leukocyte differentiation | Biological process | 35 | 483 | 934 | 17653 | 1.36959625112497 | 0.0371565541108488 | 0.201677187360891 | 1.42996456916805 | 0.0374732334047109 | DLL1//NKX2-3//FNIP1//NME1//CBFB//KLF6//IFNA1//DNAJB9//IL2//SOX4//JUN//PPARG//EPHA2//OSTM1//TNF//BCL11B//SOS1//ZEB1//FAM20C//PTGER4//TGFBR2//IRF1//TSC1//HMGB3//DUSP10//MYB//SOCS5//CDK6//CEBPB//TOB2//TMEM64//POU4F1//KLF10//STAT3//FBXW7// |
| GO:0046883 | regulation of hormone secretion | Biological process | 21 | 261 | 934 | 17653 | 1.52072411331807 | 0.0371978810435403 | 0.201739590615447 | 1.42948179875542 | 0.0224839400428266 | RAPGEF4//MARCKS//NEUROD1//SNAP25//TNF//PER2//CLOCK//INHBB//EDN1//P2RY1//SOX11//GJA1//SERP1//SOX4//SYBU//REST//OSM//EFNA5//EPHA5//MYB//TMF1// |
| GO:0006836 | neurotransmitter transport | Biological process | 20 | 246 | 934 | 17653 | 1.53662018418899 | 0.0376512044151589 | 0.204034531618365 | 1.42422112670123 | 0.0214132762312634 | SNAP25//EDN1//ARL6IP1//SLC17A7//SLC17A6//SLC32A1//WNT7A//PPFIA1//SLC6A1//SLC44A1//STX2//FMR1//SYT6//DRD1//KCNJ10//PER2//RAB3GAP1//PFN2//SLC6A14//SLC6A6// |
| GO:0006029 | proteoglycan metabolic process | Biological process | 9 | 86 | 934 | 17653 | 1.97795179522932 | 0.037794537219288 | 0.204483564171236 | 1.42257096807195 | 0.00963597430406852 | XYLT2//HS2ST1//HS3ST5//IGF1//BMPR2//SULF1//DSEL//SLC35D1//CHST7// |
| GO:0042058 | regulation of epidermal growth factor receptor signaling pathway | Biological process | 9 | 86 | 934 | 17653 | 1.97795179522932 | 0.037794537219288 | 0.204483564171236 | 1.42257096807195 | 0.00963597430406852 | EREG//FBXW7//ERRFI1//TSG101//SOCS5//EPS15//GRB2//EPN1//SOS1// |
| GO:0010950 | positive regulation of endopeptidase activity | Biological process | 15 | 171 | 934 | 17653 | 1.65793230399339 | 0.0378745483007283 | 0.204752654003058 | 1.4216525379397 | 0.0160599571734475 | DLC1//EGLN3//F3//PMAIP1//PPARG//TNF//BCL2L11//MAP3K5//REST//PPM1F//ASPH//EFNA1//EPHA4//STAT3//ST18// |
| GO:0010770 | positive regulation of cell morphogenesis involved in differentiation | Biological process | 13 | 142 | 934 | 17653 | 1.73032089754803 | 0.0380978555303922 | 0.20579536497767 | 1.41909946940387 | 0.0139186295503212 | GOLGA4//NRG1//EFNA5//LPAR3//BMPR2//SLIT2//EPHA4//RAB21//CAPRIN2//LRP8//CRK//CRKL//DOCK5// |
| GO:0031099 | regeneration | Biological process | 16 | 186 | 934 | 17653 | 1.62584329165803 | 0.0382101791265358 | 0.206237383425987 | 1.41782092636593 | 0.0171306209850107 | IGF1//WNT7A//CDKN1A//UGT1A1//PPARG//TGFBR2//NR4A3//JUN//MTPN//GJA1//KLF4//EPHA4//RGMA//PTEN//CEBPB//DUSP10// |
| GO:0007045 | cell-substrate adherens junction assembly | Biological process | 8 | 73 | 934 | 17653 | 2.07127980992051 | 0.0385263558868691 | 0.207447249094662 | 1.41424206788993 | 0.00856531049250535 | DLC1//ARHGAP6//EFNA5//SLK//KDR//TSC1//PPM1F//PTEN// |
| GO:0048041 | focal adhesion assembly | Biological process | 8 | 73 | 934 | 17653 | 2.07127980992051 | 0.0385263558868691 | 0.207447249094662 | 1.41424206788993 | 0.00856531049250535 | EFNA5//SLK//KDR//TSC1//PPM1F//DLC1//ARHGAP6//PTEN// |
| GO:0070664 | negative regulation of leukocyte proliferation | Biological process | 8 | 73 | 934 | 17653 | 2.07127980992051 | 0.0385263558868691 | 0.207447249094662 | 1.41424206788993 | 0.00856531049250535 | TNFRSF21//CEBPB//CD274//MARCH7//PDCD1LG2//IL2//SOX11//CCL8// |
| GO:0021510 | spinal cord development | Biological process | 10 | 100 | 934 | 17653 | 1.89004282655246 | 0.0387159769669355 | 0.208302428184077 | 1.41210977718579 | 0.0107066381156317 | GDF11//PBX3//LHX1//VLDLR//MNX1//SOX4//DLL1//PKD2//SOX11//NOG// |
| GO:0043112 | receptor metabolic process | Biological process | 15 | 172 | 934 | 17653 | 1.6482931626911 | 0.0395545664629587 | 0.211928176861078 | 1.40280337120085 | 0.0160599571734475 | CAMLG//EPS15//NSF//FMR1//NTF3//EDN1//PPARG//GRB2//GRIA1//TNF//MYLIP//VLDLR//NRG1//ITGAV//SNAP25// |
| GO:0050796 | regulation of insulin secretion | Biological process | 15 | 172 | 934 | 17653 | 1.6482931626911 | 0.0395545664629587 | 0.211928176861078 | 1.40280337120085 | 0.0160599571734475 | GJA1//SERP1//SOX4//SYBU//INHBB//REST//EFNA5//EPHA5//RAPGEF4//MARCKS//NEUROD1//SNAP25//TNF//PER2//CLOCK// |
| GO:0006692 | prostanoid metabolic process | Biological process | 5 | 36 | 934 | 17653 | 2.62505948132286 | 0.0396706476401958 | 0.211928176861078 | 1.40153070942584 | 0.00535331905781585 | PTGES3//EDN1//ZADH2//ACOX1//PLAA// |
| GO:0006693 | prostaglandin metabolic process | Biological process | 5 | 36 | 934 | 17653 | 2.62505948132286 | 0.0396706476401958 | 0.211928176861078 | 1.40153070942584 | 0.00535331905781585 | PTGES3//EDN1//ZADH2//ACOX1//PLAA// |
| GO:0010464 | regulation of mesenchymal cell proliferation | Biological process | 5 | 36 | 934 | 17653 | 2.62505948132286 | 0.0396706476401958 | 0.211928176861078 | 1.40153070942584 | 0.00535331905781585 | FOXF1//TGFBR2//NFIB//PHF14//ZEB1// |
| GO:0045737 | positive regulation of cyclin-dependent protein serine/threonine kinase activity | Biological process | 5 | 36 | 934 | 17653 | 2.62505948132286 | 0.0396706476401958 | 0.211928176861078 | 1.40153070942584 | 0.00535331905781585 | PKD2//CCNY//MAPRE3//CCNC//CCND2// |
| GO:0051693 | actin filament capping | Biological process | 5 | 36 | 934 | 17653 | 2.62505948132286 | 0.0396706476401958 | 0.211928176861078 | 1.40153070942584 | 0.00535331905781585 | EPS8//TWF1//TMOD3//TMOD1//MTPN// |
| GO:2000279 | negative regulation of DNA biosynthetic process | Biological process | 5 | 36 | 934 | 17653 | 2.62505948132286 | 0.0396706476401958 | 0.211928176861078 | 1.40153070942584 | 0.00535331905781585 | DCP2//PPARG//DUSP1//GJA1//BMPR2// |
| GO:0006325 | chromatin organization | Biological process | 54 | 801 | 934 | 17653 | 1.27418617520391 | 0.0397479222085914 | 0.211928176861078 | 1.40068556881703 | 0.0578158458244111 | NAP1L3//ANP32E//NFE2//SMARCD2//HMG20A//ESR1//HMGB3//MYB//DEK//KAT2B//BAHD1//H2AFZ//KMT2B//DPY30//RPS6KA4//RPS6KA5//EPC2//CLOCK//ARID4B//MIER1//ARID4A//RBBP7//TBL1XR1//BRMS1L//PPM1F//ARID5B//USP3//JARID2//HMGA2//ATAD2//FMR1//CENPQ//CENPO//PRDM16//SYCP3//PRKAA1//RNF2//SKP1//PCGF5//TADA1//DR1//DRD1//DNMT3B//KDM2A//EZH1//PER2//REST//ZMYND11//BRWD1//RSBN1//RBL2//BAG6//BRD3//TCF7L1// |
| GO:0007268 | chemical synaptic transmission | Biological process | 46 | 667 | 934 | 17653 | 1.30347781141549 | 0.0397912666248968 | 0.211928176861078 | 1.40021223628844 | 0.0492505353319058 | DRD1//SLC32A1//SNAP25//WNT7A//PPFIA1//PTEN//CHRM2//CHRM5//STX2//USP46//SLC6A1//GRIA1//SLC17A7//SLC17A6//EDN1//FMR1//CPEB3//YWHAG//EGR2//NSMF//KCNJ10//NPTN//RAB3GAP1//SYT6//NTF3//PXK//LRP8//TSHZ3//GLRA1//GLRB//SYBU//SLC24A2//MAPK1//FXR1//PAIP2//EPHA4//SQSTM1//PFN2//GJC1//LPAR3//KIF5A//LRP6//ATXN3//NOVA1//MINK1//SLC12A5// |
| GO:0098916 | anterograde trans-synaptic signaling | Biological process | 46 | 667 | 934 | 17653 | 1.30347781141549 | 0.0397912666248968 | 0.211928176861078 | 1.40021223628844 | 0.0492505353319058 | DRD1//GJC1//LPAR3//GLRA1//GLRB//GRIA1//KIF5A//LRP6//ATXN3//NOVA1//MINK1//MAPK1//SLC12A5//SLC6A1//SNAP25//SLC32A1//WNT7A//PPFIA1//PTEN//CHRM2//CHRM5//STX2//USP46//SLC17A7//SLC17A6//EDN1//FMR1//CPEB3//YWHAG//EGR2//NSMF//KCNJ10//NPTN//RAB3GAP1//SYT6//NTF3//PXK//LRP8//TSHZ3//SYBU//SLC24A2//FXR1//PAIP2//EPHA4//SQSTM1//PFN2// |
| GO:0050920 | regulation of chemotaxis | Biological process | 17 | 202 | 934 | 17653 | 1.59063010155405 | 0.0398680020398873 | 0.211928176861078 | 1.39937552818997 | 0.0182012847965739 | SLIT2//CMKLR1//ADAM10//KDR//BMPR2//SEMA4B//PPM1F//S1PR1//F3//NTF3//VEGFB//LPAR1//MPP1//EDN1//CCL2//CXCL14//MET// |
| GO:0010718 | positive regulation of epithelial to mesenchymal transition | Biological process | 6 | 48 | 934 | 17653 | 2.36255353319058 | 0.0398912567121325 | 0.211928176861078 | 1.39912228170101 | 0.00642398286937901 | TGFB2//TGFBR2//ACVR1//FOXC1//BAMBI//TGFB1I1// |
| GO:0010862 | positive regulation of pathway-restricted SMAD protein phosphorylation | Biological process | 6 | 48 | 934 | 17653 | 2.36255353319058 | 0.0398912567121325 | 0.211928176861078 | 1.39912228170101 | 0.00642398286937901 | GDF11//INHBB//BMP3//BMPR2//TGFB2//ACVR1// |
| GO:0021545 | cranial nerve development | Biological process | 6 | 48 | 934 | 17653 | 2.36255353319058 | 0.0398912567121325 | 0.211928176861078 | 1.39912228170101 | 0.00642398286937901 | LPAR1//NEUROG1//POU4F1//TFAP2A//EGR2//HOXA3// |
| GO:0051496 | positive regulation of stress fiber assembly | Biological process | 6 | 48 | 934 | 17653 | 2.36255353319058 | 0.0398912567121325 | 0.211928176861078 | 1.39912228170101 | 0.00642398286937901 | LPAR1//PPM1E//NF2//PFN2//TSC1//PPM1F// |
| GO:0006835 | dicarboxylic acid transport | Biological process | 9 | 87 | 934 | 17653 | 1.95521671712324 | 0.0402625777080852 | 0.213397972601709 | 1.39509842396896 | 0.00963597430406852 | ARL6IP1//SLC17A7//SLC17A6//SNAP25//PPFIA1//SLC25A32//KCNJ10//PER2//RAB3GAP1// |
| GO:0006903 | vesicle targeting | Biological process | 9 | 87 | 934 | 17653 | 1.95521671712324 | 0.0402625777080852 | 0.213397972601709 | 1.39509842396896 | 0.00963597430406852 | TMED9//CNIH1//SEC23A//TBC1D20//GRIA1//NSF//SAR1B//PPP6C//MCFD2// |
| GO:0090559 | regulation of membrane permeability | Biological process | 9 | 87 | 934 | 17653 | 1.95521671712324 | 0.0402625777080852 | 0.213397972601709 | 1.39509842396896 | 0.00963597430406852 | BCL2L11//PMAIP1//MOAP1//BNIP3//STAT3//DYNLL2//E2F1//PPP3R1//YWHAG// |
| GO:1901987 | regulation of cell cycle phase transition | Biological process | 33 | 454 | 934 | 17653 | 1.37381967568791 | 0.0405550601611796 | 0.213605879766019 | 1.39195495001085 | 0.0353319057815846 | CDKN1A//GADD45A//E2F1//CNOT6L//CNOT7//MDM4//CNOT6//RBL2//SOX4//BTG2//MTBP//TAOK3//HMGA2//CDC14A//CDC23//CDK6//NEDD1//PRKAR2B//YWHAG//HAUS8//CEP41//RAD21//SKP1//HECW2//NEUROG1//PTEN//PKD2//DUSP1//CCND2//TMOD3//FBXW7//ZNF655//KLF4// |
| GO:0009299 | mRNA transcription | Biological process | 4 | 25 | 934 | 17653 | 3.02406852248394 | 0.0405860646901278 | 0.213605879766019 | 1.39162305674425 | 0.00428265524625268 | SOX17//STAT3//HIPK3//EREG// |
| GO:0009648 | photoperiodism | Biological process | 4 | 25 | 934 | 17653 | 3.02406852248394 | 0.0405860646901278 | 0.213605879766019 | 1.39162305674425 | 0.00428265524625268 | CRY2//BHLHE40//PER2//CLOCK// |
| GO:0045939 | negative regulation of steroid metabolic process | Biological process | 4 | 25 | 934 | 17653 | 3.02406852248394 | 0.0405860646901278 | 0.213605879766019 | 1.39162305674425 | 0.00428265524625268 | DKK3//REST//UGT1A8//UGT1A1// |
| GO:0046885 | regulation of hormone biosynthetic process | Biological process | 4 | 25 | 934 | 17653 | 3.02406852248394 | 0.0405860646901278 | 0.213605879766019 | 1.39162305674425 | 0.00428265524625268 | DKK3//REST//TNF//TRERF1// |
| GO:0048679 | regulation of axon regeneration | Biological process | 4 | 25 | 934 | 17653 | 3.02406852248394 | 0.0405860646901278 | 0.213605879766019 | 1.39162305674425 | 0.00428265524625268 | EPHA4//RGMA//PTEN//KLF4// |
| GO:0060037 | pharyngeal system development | Biological process | 4 | 25 | 934 | 17653 | 3.02406852248394 | 0.0405860646901278 | 0.213605879766019 | 1.39162305674425 | 0.00428265524625268 | BMPR2//TGFB2//NOG//ACVR1// |
| GO:0060561 | apoptotic process involved in morphogenesis | Biological process | 4 | 25 | 934 | 17653 | 3.02406852248394 | 0.0405860646901278 | 0.213605879766019 | 1.39162305674425 | 0.00428265524625268 | HAND2//TGFB2//FOXC1//BCL2L11// |
| GO:0061037 | negative regulation of cartilage development | Biological process | 4 | 25 | 934 | 17653 | 3.02406852248394 | 0.0405860646901278 | 0.213605879766019 | 1.39162305674425 | 0.00428265524625268 | PTHLH//RARB//FRZB//NOG// |
| GO:0009101 | glycoprotein biosynthetic process | Biological process | 26 | 342 | 934 | 17653 | 1.43687466346094 | 0.0408134932973171 | 0.214469040535941 | 1.38919623162173 | 0.0278372591006424 | FUT9//SERP1//ST8SIA5//MAN1A1//MAN2A1//ST8SIA3//B3GNT5//B3GALT2//CCDC126//GFPT2//VEGFB//IGF1//XYLT2//HS2ST1//HS3ST5//ST3GAL3//ST6GALNAC3//ST6GALNAC6//ST6GAL2//ST3GAL5//MCFD2//BMPR2//SLC35D1//CHST7//DSEL//ITM2B// |
| GO:0019932 | second-messenger-mediated signaling | Biological process | 26 | 342 | 934 | 17653 | 1.43687466346094 | 0.0408134932973171 | 0.214469040535941 | 1.38919623162173 | 0.0278372591006424 | ADCY3//DRD1//GNAQ//PTGER4//PTHLH//EDN1//NEUROD1//RASD1//CALM2//RAPGEF4//EPHA5//PKD2//PPP3R1//KDR//PDE10A//PEX5L//ADRB1//LPAR1//CMKLR1//TMEM100//PLEK//PRKAA1//NRG1//IGF1//NR5A2//PPP2CA// |
| GO:0010923 | negative regulation of phosphatase activity | Biological process | 10 | 101 | 934 | 17653 | 1.87132953124006 | 0.0410233282598396 | 0.215237214135993 | 1.38696910783294 | 0.0107066381156317 | TGFB2//TNF//CRY2//PPP1R37//MASTL//URI1//SPRED1//PPP1R12A//PPP1R12C//PLEKHF2// |
| GO:2000278 | regulation of DNA biosynthetic process | Biological process | 10 | 101 | 934 | 17653 | 1.87132953124006 | 0.0410233282598396 | 0.215237214135993 | 1.38696910783294 | 0.0107066381156317 | DCP2//MAPK1//CCT6A//PTGES3//KLF4//PPARG//DUSP1//GJA1//BMPR2//CDKN1A// |
| GO:0034754 | cellular hormone metabolic process | Biological process | 11 | 115 | 934 | 17653 | 1.80786705148496 | 0.0411023785595622 | 0.215484795502573 | 1.38613304514576 | 0.0117773019271949 | RDH10//ESR1//PLEKHA1//UGT1A1//DKK3//REST//TTR//UGT1A8//UGT1A7//UGT1A9//UGT1A3// |
| GO:0030433 | ubiquitin-dependent ERAD pathway | Biological process | 8 | 74 | 934 | 17653 | 2.04328954221888 | 0.0412792762334867 | 0.216244574103076 | 1.38426792597771 | 0.00856531049250535 | EDEM1//UBXN4//DNAJB9//DERL2//JKAMP//NPLOC4//WFS1//BAG6// |
| GO:0010952 | positive regulation of peptidase activity | Biological process | 16 | 188 | 934 | 17653 | 1.60854708642763 | 0.0414892933864695 | 0.216302052547197 | 1.3820639616755 | 0.0171306209850107 | DLC1//EGLN3//F3//PMAIP1//PPARG//TNF//BCL2L11//MAP3K5//REST//PPM1F//ASPH//EFNA1//EPHA4//STAT3//ST18//PCOLCE2// |
| GO:0031645 | negative regulation of neurological system process | Biological process | 3 | 15 | 934 | 17653 | 3.78008565310492 | 0.0417060293540654 | 0.216302052547197 | 1.37980115543597 | 0.00321199143468951 | TNFRSF21//PTEN//GLRA1// |
| GO:0031657 | regulation of cyclin-dependent protein serine/threonine kinase activity involved in G1/S transition of mitotic cell cycle | Biological process | 3 | 15 | 934 | 17653 | 3.78008565310492 | 0.0417060293540654 | 0.216302052547197 | 1.37980115543597 | 0.00321199143468951 | CDK6//PTEN//PKD2// |
| GO:0042921 | glucocorticoid receptor signaling pathway | Biological process | 3 | 15 | 934 | 17653 | 3.78008565310492 | 0.0417060293540654 | 0.216302052547197 | 1.37980115543597 | 0.00321199143468951 | CRY2//CLOCK//LMO3// |
| GO:0048569 | post-embryonic animal organ development | Biological process | 3 | 15 | 934 | 17653 | 3.78008565310492 | 0.0417060293540654 | 0.216302052547197 | 1.37980115543597 | 0.00321199143468951 | BCL11B//KLF4//BCL2L11// |
| GO:0051044 | positive regulation of membrane protein ectodomain proteolysis | Biological process | 3 | 15 | 934 | 17653 | 3.78008565310492 | 0.0417060293540654 | 0.216302052547197 | 1.37980115543597 | 0.00321199143468951 | SH3D19//TNF//ADAM9// |
| GO:0051895 | negative regulation of focal adhesion assembly | Biological process | 3 | 15 | 934 | 17653 | 3.78008565310492 | 0.0417060293540654 | 0.216302052547197 | 1.37980115543597 | 0.00321199143468951 | DLC1//ARHGAP6//PTEN// |
| GO:0051957 | positive regulation of amino acid transport | Biological process | 3 | 15 | 934 | 17653 | 3.78008565310492 | 0.0417060293540654 | 0.216302052547197 | 1.37980115543597 | 0.00321199143468951 | ARL6IP1//SLC6A1//RAB3GAP1// |
| GO:0070571 | negative regulation of neuron projection regeneration | Biological process | 3 | 15 | 934 | 17653 | 3.78008565310492 | 0.0417060293540654 | 0.216302052547197 | 1.37980115543597 | 0.00321199143468951 | EPHA4//RGMA//PTEN// |
| GO:0072075 | metanephric mesenchyme development | Biological process | 3 | 15 | 934 | 17653 | 3.78008565310492 | 0.0417060293540654 | 0.216302052547197 | 1.37980115543597 | 0.00321199143468951 | OSR1//TCF21//PKD2// |
| GO:1901070 | guanosine-containing compound biosynthetic process | Biological process | 3 | 15 | 934 | 17653 | 3.78008565310492 | 0.0417060293540654 | 0.216302052547197 | 1.37980115543597 | 0.00321199143468951 | IMPDH1//NME1//HPRT1// |
| GO:1905288 | vascular associated smooth muscle cell apoptotic process | Biological process | 3 | 15 | 934 | 17653 | 3.78008565310492 | 0.0417060293540654 | 0.216302052547197 | 1.37980115543597 | 0.00321199143468951 | IGF1//E2F3//PPARG// |
| GO:1905459 | regulation of vascular associated smooth muscle cell apoptotic process | Biological process | 3 | 15 | 934 | 17653 | 3.78008565310492 | 0.0417060293540654 | 0.216302052547197 | 1.37980115543597 | 0.00321199143468951 | IGF1//E2F3//PPARG// |
| GO:0035148 | tube formation | Biological process | 13 | 144 | 934 | 17653 | 1.70628866285986 | 0.0419034992921785 | 0.217159667213029 | 1.37774970830275 | 0.0139186295503212 | SOX4//SOX11//DLC1//LRP6//PRKACB//TGFB2//TSC1//FZD6//NOG//HAND1//NFIB//OSR1//BCL2L11// |
| GO:0099537 | trans-synaptic signaling | Biological process | 46 | 670 | 934 | 17653 | 1.29764134360318 | 0.0423750046984826 | 0.219435035816109 | 1.37289024063488 | 0.0492505353319058 | DRD1//GJC1//LPAR3//GLRA1//GLRB//GRIA1//KIF5A//LRP6//ATXN3//NOVA1//MINK1//MAPK1//SLC12A5//SLC6A1//SNAP25//SLC32A1//WNT7A//PPFIA1//PTEN//CHRM2//CHRM5//STX2//USP46//SLC17A7//SLC17A6//EDN1//FMR1//CPEB3//YWHAG//EGR2//NSMF//KCNJ10//NPTN//RAB3GAP1//SYT6//NTF3//PXK//LRP8//TSHZ3//SYBU//SLC24A2//FXR1//PAIP2//EPHA4//SQSTM1//PFN2// |
| GO:0002181 | cytoplasmic translation | Biological process | 9 | 88 | 934 | 17653 | 1.93299834533775 | 0.0428374220739592 | 0.221659897082009 | 1.36817667259138 | 0.00963597430406852 | CPEB2//CPEB3//NCK1//YTHDF2//FMR1//CPEB1//CPEB4//UNK//RPL15// |
| GO:0009408 | response to heat | Biological process | 15 | 174 | 934 | 17653 | 1.62934726426936 | 0.0430734832524721 | 0.222540845864376 | 1.36579000643198 | 0.0160599571734475 | CDKN1A//CETN1//ATXN3//IER5//HSPA13//HSBP1//RBBP7//PTGES3//NUP35//NUP43//MAPK1//BAG5//DNAJB4//IGF1//TGFB1I1// |
| GO:0071466 | cellular response to xenobiotic stimulus | Biological process | 15 | 174 | 934 | 17653 | 1.62934726426936 | 0.0430734832524721 | 0.222540845864376 | 1.36579000643198 | 0.0160599571734475 | PTGES3//UGT1A6//UGT1A9//ACSL1//UGT1A10//UGT1A8//UGT1A7//UGT1A5//UGT1A4//UGT1A1//UGT1A3//DNMT3B//GJB2//ERRFI1//E2F1// |
| GO:0099536 | synaptic signaling | Biological process | 46 | 671 | 934 | 17653 | 1.29570745188395 | 0.0432639953462027 | 0.223354504218602 | 1.36387337679042 | 0.0492505353319058 | DRD1//GJC1//LPAR3//GLRA1//GLRB//GRIA1//KIF5A//LRP6//ATXN3//NOVA1//MINK1//MAPK1//SLC12A5//SLC6A1//SNAP25//SLC32A1//WNT7A//PPFIA1//PTEN//CHRM2//CHRM5//STX2//USP46//SLC17A7//SLC17A6//EDN1//FMR1//CPEB3//YWHAG//EGR2//NSMF//KCNJ10//NPTN//RAB3GAP1//SYT6//NTF3//PXK//LRP8//TSHZ3//SYBU//SLC24A2//FXR1//PAIP2//EPHA4//SQSTM1//PFN2// |
| GO:0045785 | positive regulation of cell adhesion | Biological process | 29 | 392 | 934 | 17653 | 1.398245968623 | 0.0433770451408074 | 0.22366342664017 | 1.36274003560329 | 0.0310492505353319 | CDK6//FOXF1//NPNT//ITGA6//ITGA5//SMOC2//PPM1F//GRB2//CD274//PDCD1LG2//TGFB2//ADAM9//TNF//IL2//NCK1//IGF1//DUSP10//MYB//SOCS5//CCL2//TGFBR2//KDR//TSC1//CRK//CRKL//DOCK5//NR4A3//ETS1//ITGAV// |
| GO:0001823 | mesonephros development | Biological process | 10 | 102 | 934 | 17653 | 1.85298316328673 | 0.0434230488213136 | 0.22366342664017 | 1.36227968710481 | 0.0107066381156317 | GDF11//OSR1//FOXC1//NPNT//LHX1//RARB//TCF21//SLIT2//PKD2//NOG// |
| GO:0099173 | postsynapse organization | Biological process | 10 | 102 | 934 | 17653 | 1.85298316328673 | 0.0434230488213136 | 0.22366342664017 | 1.36227968710481 | 0.0107066381156317 | CTNND2//PTEN//WNT7A//WASL//EPHA4//EFNA1//CAPRIN2//LRP8//GLRB//ZNF804A// |
| GO:0009994 | oocyte differentiation | Biological process | 6 | 49 | 934 | 17653 | 2.3143381549622 | 0.043506110747146 | 0.223750438770303 | 1.36144973896766 | 0.00642398286937901 | EREG//YTHDF2//TDRD5//INHBB//IGF1//KMT2B// |
| GO:2000401 | regulation of lymphocyte migration | Biological process | 6 | 49 | 934 | 17653 | 2.3143381549622 | 0.043506110747146 | 0.223750438770303 | 1.36144973896766 | 0.00642398286937901 | ADAM10//WASL//LRCH1//CCL2//CXCL14//MSN// |
| GO:0032504 | multicellular organism reproduction | Biological process | 55 | 823 | 934 | 17653 | 1.26309058882607 | 0.0437899613723019 | 0.224826379791169 | 1.35862543790455 | 0.0588865096359743 | ESR1//EREG//PLEKHA1//YTHDF2//TBC1D20//AGFG1//TMF1//BMPR2//MYBL1//SYCP3//MASTL//DZIP1//RNF2//ACVR1//BCL2L11//CRTAP//DDX6//E2F1//GOLGA3//AR//CLDN11//ACOX1//PAIP2//ARID4B//ARID4A//HERPUD2//SOX17//BTG1//BAG6//IMMP2L//ADAM18//CLOCK//TDRD5//QKI//TOB2//SOS1//FOSL1//DRD1//NHLH2//THRA//FOXC1//KMT2B//TGFB2//CREBRF//GNAQ//PTEN//ETS1//GJA1//GJB2//INHBB//EDN1//DMRTA1//IGF1//P2RY1//SLIT2// |
| GO:0007340 | acrosome reaction | Biological process | 5 | 37 | 934 | 17653 | 2.5541119277736 | 0.0439480221919156 | 0.224826379791169 | 1.35706066485562 | 0.00535331905781585 | SYT6//GLRA1//ADCY3//STX2//GLRB// |
| GO:0009225 | nucleotide-sugar metabolic process | Biological process | 5 | 37 | 934 | 17653 | 2.5541119277736 | 0.0439480221919156 | 0.224826379791169 | 1.35706066485562 | 0.00535331905781585 | UGP2//GFPT2//NAGK//SLC35D1//UXS1// |
| GO:0021587 | cerebellum morphogenesis | Biological process | 5 | 37 | 934 | 17653 | 2.5541119277736 | 0.0439480221919156 | 0.224826379791169 | 1.35706066485562 | 0.00535331905781585 | DLL1//LHX1//MTPN//WNT7A//LRP6// |
| GO:0042149 | cellular response to glucose starvation | Biological process | 5 | 37 | 934 | 17653 | 2.5541119277736 | 0.0439480221919156 | 0.224826379791169 | 1.35706066485562 | 0.00535331905781585 | SESN3//SESN1//PMAIP1//PRKAA1//CPEB4// |
| GO:0061001 | regulation of dendritic spine morphogenesis | Biological process | 5 | 37 | 934 | 17653 | 2.5541119277736 | 0.0439480221919156 | 0.224826379791169 | 1.35706066485562 | 0.00535331905781585 | EFNA1//PTEN//CAPRIN2//LRP8//EPHA4// |
| GO:0090503 | RNA phosphodiester bond hydrolysis, exonucleolytic | Biological process | 5 | 37 | 934 | 17653 | 2.5541119277736 | 0.0439480221919156 | 0.224826379791169 | 1.35706066485562 | 0.00535331905781585 | DCP2//CNOT6L//PAN3//CNOT7//CNOT6// |
| GO:0007032 | endosome organization | Biological process | 8 | 75 | 934 | 17653 | 2.01604568165596 | 0.0441596502103948 | 0.225338471326887 | 1.35497437532182 | 0.00856531049250535 | VPS37A//CHMP3//TSG101//STX6//RAB22A//AKTIP//FAM160A2//SQSTM1// |
| GO:0030512 | negative regulation of transforming growth factor beta receptor signaling pathway | Biological process | 8 | 75 | 934 | 17653 | 2.01604568165596 | 0.0441596502103948 | 0.225338471326887 | 1.35497437532182 | 0.00856531049250535 | BAMBI//ASPN//PMEPA1//PRDM16//RASL11B//TGFB1I1//TGFBR2//LDLRAD4// |
| GO:0033143 | regulation of intracellular steroid hormone receptor signaling pathway | Biological process | 8 | 75 | 934 | 17653 | 2.01604568165596 | 0.0441596502103948 | 0.225338471326887 | 1.35497437532182 | 0.00856531049250535 | ESR1//UBA5//CBFB//AR//TCF21//CRY2//CLOCK//LMO3// |
| GO:0060627 | regulation of vesicle-mediated transport | Biological process | 35 | 490 | 934 | 17653 | 1.35003059039462 | 0.0441814007067059 | 0.225338471326887 | 1.35476051957481 | 0.0374732334047109 | FMR1//NTF3//RAPGEF4//RAB21//NSF//SYT6//TRPV6//RUFY2//TBC1D12//EVI5//TBC1D17//USP6NL//LYPLA1//PKDCC//FOXF1//DLL1//LDLR//REST//CD2AP//ITGAV//PTEN//TNF//PPARG//TBC1D20//RAB3GAP1//TSG101//PFN2//PIK3CB//WASL//CCL2//CHMP3//MAPK1//MSN//ARHGAP1//PRKAA1// |
| GO:1901991 | negative regulation of mitotic cell cycle phase transition | Biological process | 19 | 236 | 934 | 17653 | 1.52164464849563 | 0.0455439652403401 | 0.232112914031967 | 1.34156916065475 | 0.0203426124197002 | CDKN1A//GADD45A//E2F1//CNOT6L//CNOT7//MDM4//CNOT6//RBL2//SOX4//BTG2//TAOK3//HMGA2//RAD21//SKP1//DUSP1//PKD2//PTEN//ZNF655//KLF4// |
| GO:0008630 | intrinsic apoptotic signaling pathway in response to DNA damage | Biological process | 10 | 103 | 934 | 17653 | 1.83499303548783 | 0.0459164371740982 | 0.233343127864428 | 1.33803181781519 | 0.0107066381156317 | TOPORS//CDKN1A//USP28//BAG6//PIAS4//BCL2L11//E2F1//EPHA2//MOAP1//TNF// |
| GO:0022037 | metencephalon development | Biological process | 10 | 103 | 934 | 17653 | 1.83499303548783 | 0.0459164371740982 | 0.233343127864428 | 1.33803181781519 | 0.0107066381156317 | LPAR1//EN1//FOXC1//LHX1//NEUROD1//LRP6//DLL1//MTPN//WNT7A//NANOS1// |
| GO:0050806 | positive regulation of synaptic transmission | Biological process | 13 | 146 | 934 | 17653 | 1.68291484556041 | 0.0459685696713607 | 0.233343127864428 | 1.33753900926904 | 0.0139186295503212 | DRD1//TSHZ3//SLC24A2//NPTN//MAPK1//PTEN//SNAP25//RAB3GAP1//PAIP2//EPHA4//SQSTM1//FMR1//WNT7A// |
| GO:0006515 | protein quality control for misfolded or incompletely synthesized proteins | Biological process | 4 | 26 | 934 | 17653 | 2.9077581946961 | 0.0460268715911794 | 0.233343127864428 | 1.33698854274365 | 0.00428265524625268 | KLHL15//BAG6//ATXN3//UBE2W// |
| GO:0042481 | regulation of odontogenesis | Biological process | 4 | 26 | 934 | 17653 | 2.9077581946961 | 0.0460268715911794 | 0.233343127864428 | 1.33698854274365 | 0.00428265524625268 | EDN1//ASPN//TFAP2A//PAX9// |
| GO:0060740 | prostate gland epithelium morphogenesis | Biological process | 4 | 26 | 934 | 17653 | 2.9077581946961 | 0.0460268715911794 | 0.233343127864428 | 1.33698854274365 | 0.00428265524625268 | NOG//AR//ESR1//SULF1// |
| GO:1900745 | positive regulation of p38MAPK cascade | Biological process | 4 | 26 | 934 | 17653 | 2.9077581946961 | 0.0460268715911794 | 0.233343127864428 | 1.33698854274365 | 0.00428265524625268 | GADD45A//MAP3K5//MINK1//HAND2// |
| GO:0043491 | protein kinase B signaling | Biological process | 20 | 252 | 934 | 17653 | 1.50003398932735 | 0.0464303372442539 | 0.235212262758718 | 1.33319816184779 | 0.0214132762312634 | SESN3//EREG//ESR1//F3//GAB1//GRB2//NRG1//MET//PIK3CB//TNF//EPHA2//OTUD3//PTEN//PLEKHA1//KLF4//CDKN1A//IGF1//KDR//TMEM100//CCL2// |
| GO:0002573 | myeloid leukocyte differentiation | Biological process | 16 | 191 | 934 | 17653 | 1.58328194894447 | 0.0467787154146195 | 0.236422258973346 | 1.32995170847818 | 0.0171306209850107 | NME1//JUN//PPARG//NKX2-3//EPHA2//OSTM1//TNF//FAM20C//TGFBR2//CDK6//CEBPB//TOB2//TMEM64//POU4F1//KLF10//FBXW7// |
| GO:0002685 | regulation of leukocyte migration | Biological process | 15 | 176 | 934 | 17653 | 1.61083195444812 | 0.0468090203704436 | 0.236422258973346 | 1.32967044778866 | 0.0160599571734475 | MIA3//CCL8//SLIT2//CMKLR1//ADAM10//VEGFB//TNF//MPP1//EDN1//MSN//WASL//LRCH1//PTGER4//CCL2//CXCL14// |
| GO:0030041 | actin filament polymerization | Biological process | 15 | 176 | 934 | 17653 | 1.61083195444812 | 0.0468090203704436 | 0.236422258973346 | 1.32967044778866 | 0.0160599571734475 | EPS8//TWF1//PFN2//SLIT2//CDC42EP3//GRB2//NCK1//IQGAP2//ARFIP1//WASL//TMOD3//TMOD1//MTPN//WASF3//ARHGAP6// |
| GO:2001235 | positive regulation of apoptotic signaling pathway | Biological process | 15 | 176 | 934 | 17653 | 1.61083195444812 | 0.0468090203704436 | 0.236422258973346 | 1.32967044778866 | 0.0160599571734475 | BCL2L11//PMAIP1//MOAP1//BNIP3//DYNLL2//E2F1//PPP3R1//YWHAG//PIAS4//SERINC3//NCK1//FBXW7//PTEN//ST18//INHBB// |
| GO:0031333 | negative regulation of protein complex assembly | Biological process | 12 | 132 | 934 | 17653 | 1.71822075141133 | 0.0471574300161689 | 0.237229050962544 | 1.32644987109623 | 0.012847965738758 | EPS8//TWF1//PMEPA1//LDLRAD4//THRA//PFN2//SLIT2//STMN2//PTGER4//TMOD3//TMOD1//MTPN// |
| GO:0034605 | cellular response to heat | Biological process | 12 | 132 | 934 | 17653 | 1.71822075141133 | 0.0471574300161689 | 0.237229050962544 | 1.32644987109623 | 0.012847965738758 | HSBP1//RBBP7//PTGES3//NUP35//NUP43//MAPK1//BAG5//IER5//CDKN1A//CETN1//ATXN3//HSPA13// |
| GO:0021536 | diencephalon development | Biological process | 8 | 76 | 934 | 17653 | 1.98951876479207 | 0.0471691767072196 | 0.237229050962544 | 1.32634170388217 | 0.00856531049250535 | ARX//LRP6//ETS1//RAB3GAP1//POU3F2//NOG//NR4A2//POU4F1// |
| GO:1903829 | positive regulation of cellular protein localization | Biological process | 26 | 347 | 934 | 17653 | 1.41617041758974 | 0.0472107002885519 | 0.237229050962544 | 1.32595955751837 | 0.0278372591006424 | IGF1//MAPK1//IPO5//CTDSPL2//MIEF1//CD2AP//BCL2L11//DYNLL2//E2F1//PMAIP1//PPP3R1//YWHAG//MSN//EPHA2//SQSTM1//NRG1//FBXW7//ELMOD1//PRKAA1//SNIP1//EDEM1//CCT6A//CENPQ//TNF//TSG101//NPTN// |
| GO:0032890 | regulation of organic acid transport | Biological process | 6 | 50 | 934 | 17653 | 2.26805139186296 | 0.0473195312359119 | 0.237229050962544 | 1.32495956629823 | 0.00642398286937901 | ARL6IP1//SLC6A1//EDN1//PER2//RAB3GAP1//OSR1// |
| GO:0035272 | exocrine system development | Biological process | 6 | 50 | 934 | 17653 | 2.26805139186296 | 0.0473195312359119 | 0.237229050962544 | 1.32495956629823 | 0.00642398286937901 | TGFB2//FOXC1//NFIB//TNF//NTN4//BTBD7// |
| GO:0045661 | regulation of myoblast differentiation | Biological process | 6 | 50 | 934 | 17653 | 2.26805139186296 | 0.0473195312359119 | 0.237229050962544 | 1.32495956629823 | 0.00642398286937901 | SOSTDC1//DLL1//TNF//CXCL14//MAP3K5//BTG1// |
| GO:0098659 | inorganic cation import across plasma membrane | Biological process | 6 | 50 | 934 | 17653 | 2.26805139186296 | 0.0473195312359119 | 0.237229050962544 | 1.32495956629823 | 0.00642398286937901 | TRPV6//FAM155A//STEAP2//ARHGAP1//HCN2//KCNJ3// |
| GO:0099587 | inorganic ion import across plasma membrane | Biological process | 6 | 50 | 934 | 17653 | 2.26805139186296 | 0.0473195312359119 | 0.237229050962544 | 1.32495956629823 | 0.00642398286937901 | TRPV6//FAM155A//STEAP2//ARHGAP1//HCN2//KCNJ3// |
| GO:1990090 | cellular response to nerve growth factor stimulus | Biological process | 6 | 50 | 934 | 17653 | 2.26805139186296 | 0.0473195312359119 | 0.237229050962544 | 1.32495956629823 | 0.00642398286937901 | NTF3//STMN2//CRK//DYNC1LI2//E2F1//PTEN// |
| GO:0071453 | cellular response to oxygen levels | Biological process | 18 | 222 | 934 | 17653 | 1.53246715666416 | 0.0478087312470741 | 0.239504036610342 | 1.32049278150199 | 0.019271948608137 | CPEB4//EGLN3//EGLN1//DNMT3B//PPARG//CPEB2//E2F1//EDN1//GATA6//MDM4//PMAIP1//PRKAA1//PTEN//CPEB1//SLC2A4//BNIP3//TSC1//LPAR1// |
| GO:0030032 | lamellipodium assembly | Biological process | 7 | 63 | 934 | 17653 | 2.10004758505829 | 0.0479893293167692 | 0.240230817297787 | 1.31885531958334 | 0.00749464668094218 | EPHA2//TWF1//SLIT2//WASF3//S1PR1//NCK1//ARHGEF4// |
| GO:0043500 | muscle adaptation | Biological process | 11 | 118 | 934 | 17653 | 1.76190432983704 | 0.0480503137417841 | 0.240358189227578 | 1.31830377228512 | 0.0117773019271949 | MTPN//EDN1//IGF1//TWF1//NR4A3//HAND2//JARID2//KLF4//GATA6//ERRFI1//TRPC3// |
| GO:0014074 | response to purine-containing compound | Biological process | 13 | 147 | 934 | 17653 | 1.67146644525048 | 0.0481005495344058 | 0.240431645603242 | 1.31784996191314 | 0.0139186295503212 | DNMT3B//PPARG//PRKAA1//PTEN//TRPC3//DUSP1//JUN//NME1//FOSL1//PKD2//RAP1B//HCN2//SLC6A1// |
| GO:1901342 | regulation of vasculature development | Biological process | 29 | 396 | 934 | 17653 | 1.38412227197024 | 0.0483063846980386 | 0.241026708087559 | 1.31599546436496 | 0.0310492505353319 | SPRED1//FOXC1//SULF1//PPARG//TGFB2//KLF4//EFNA1//EPHA2//ETS1//EGLN1//VASH2//F3//GAB1//GATA6//ITGB8//KDR//STAT3//BTG1//TGFBR2//VEGFB//ADAM12//HMGA2//MEOX2//KLF2//EPN1//DLL1//ITGA5//JAK1//TMEM100// |
| GO:0001523 | retinoid metabolic process | Biological process | 9 | 90 | 934 | 17653 | 1.89004282655246 | 0.0483135104234779 | 0.241026708087559 | 1.31593140585528 | 0.00963597430406852 | RDH10//TTR//UGT1A8//UGT1A7//UGT1A9//UGT1A1//UGT1A3//NAPEPLD//LRP8// |
| GO:0019058 | viral life cycle | Biological process | 24 | 316 | 934 | 17653 | 1.43547556447022 | 0.0483423666614001 | 0.241026708087559 | 1.31567209197498 | 0.0256959314775161 | NFIA//CCL2//DEK//WWP2//EPHA2//EPS15//ITGA5//ITGAV//LDLR//TBC1D20//USP6NL//DDX6//ATG16L1//VPS37A//CHMP3//TSG101//FMR1//NR5A2//CNOT7//TNF//HMGA2//HS3ST5//NUP35//NUP43// |
| GO:0002686 | negative regulation of leukocyte migration | Biological process | 5 | 38 | 934 | 17653 | 2.48689845599008 | 0.0484909520324674 | 0.241026708087559 | 1.31433928921117 | 0.00535331905781585 | SLIT2//WASL//LRCH1//PTGER4//CCL2// |
| GO:0007431 | salivary gland development | Biological process | 5 | 38 | 934 | 17653 | 2.48689845599008 | 0.0484909520324674 | 0.241026708087559 | 1.31433928921117 | 0.00535331905781585 | TGFB2//NFIB//TNF//NTN4//BTBD7// |
| GO:0040019 | positive regulation of embryonic development | Biological process | 5 | 38 | 934 | 17653 | 2.48689845599008 | 0.0484909520324674 | 0.241026708087559 | 1.31433928921117 | 0.00535331905781585 | AR//IGF1//OSR1//LHX1//OTX2// |
| GO:1905209 | positive regulation of cardiocyte differentiation | Biological process | 5 | 38 | 934 | 17653 | 2.48689845599008 | 0.0484909520324674 | 0.241026708087559 | 1.31433928921117 | 0.00535331905781585 | GATA6//TGFB2//EDN1//IGF1//NRG1// |
| GO:1901800 | positive regulation of proteasomal protein catabolic process | Biological process | 10 | 104 | 934 | 17653 | 1.81734887168506 | 0.0485047094051704 | 0.241026708087559 | 1.31421609295679 | 0.0107066381156317 | RNF180//ANKIB1//LONRF3//LONRF1//SOCS5//EDEM1//ATXN3//BAG6//FMR1//FBXW7// |
| GO:0030509 | BMP signaling pathway | Biological process | 14 | 162 | 934 | 17653 | 1.63337034393423 | 0.0486032367699481 | 0.241338979644023 | 1.31333480759945 | 0.0149892933618844 | SULF1//GATA6//BMPR2//SOX11//TOB1//SOSTDC1//CTDSPL2//NOG//ACVR1//RGMB//TMEM100//RGMA//BMP3//TGFB2// |
| GO:0009100 | glycoprotein metabolic process | Biological process | 30 | 413 | 934 | 17653 | 1.37291246480808 | 0.0493538022707348 | 0.242295073001731 | 1.30667938318506 | 0.0321199143468951 | FUT9//SERP1//ST8SIA5//MAN1A1//MAN2A1//ST8SIA3//B3GNT5//B3GALT2//CCDC126//GFPT2//EDEM1//VEGFB//IGF1//XYLT2//HS2ST1//HS3ST5//GNPTG//ST3GAL3//ST6GALNAC3//ST6GALNAC6//ST6GAL2//ST3GAL5//MCFD2//BMPR2//SULF1//DSEL//SLC35D1//CHST7//ITM2B//DERL2// |
| GO:0006474 | N-terminal protein amino acid acetylation | Biological process | 3 | 16 | 934 | 17653 | 3.54383029978587 | 0.0493690094035761 | 0.242295073001731 | 1.3065455868756 | 0.00321199143468951 | NAA30//KAT2B//SOX4// |
| GO:0007379 | segment specification | Biological process | 3 | 16 | 934 | 17653 | 3.54383029978587 | 0.0493690094035761 | 0.242295073001731 | 1.3065455868756 | 0.00321199143468951 | DLL1//MEOX2//OSR1// |
| GO:0010663 | positive regulation of striated muscle cell apoptotic process | Biological process | 3 | 16 | 934 | 17653 | 3.54383029978587 | 0.0493690094035761 | 0.242295073001731 | 1.3065455868756 | 0.00321199143468951 | MAP3K5//PTEN//BNIP3// |
| GO:0010666 | positive regulation of cardiac muscle cell apoptotic process | Biological process | 3 | 16 | 934 | 17653 | 3.54383029978587 | 0.0493690094035761 | 0.242295073001731 | 1.3065455868756 | 0.00321199143468951 | MAP3K5//PTEN//BNIP3// |
| GO:0034393 | positive regulation of smooth muscle cell apoptotic process | Biological process | 3 | 16 | 934 | 17653 | 3.54383029978587 | 0.0493690094035761 | 0.242295073001731 | 1.3065455868756 | 0.00321199143468951 | E2F3//PPARG//MAP2K4// |
| GO:0043508 | negative regulation of JUN kinase activity | Biological process | 3 | 16 | 934 | 17653 | 3.54383029978587 | 0.0493690094035761 | 0.242295073001731 | 1.3065455868756 | 0.00321199143468951 | HIPK3//DUSP10//AIDA// |
| GO:0044065 | regulation of respiratory system process | Biological process | 3 | 16 | 934 | 17653 | 3.54383029978587 | 0.0493690094035761 | 0.242295073001731 | 1.3065455868756 | 0.00321199143468951 | GLRA1//PBX3//TSHZ3// |
| GO:0048532 | anatomical structure arrangement | Biological process | 3 | 16 | 934 | 17653 | 3.54383029978587 | 0.0493690094035761 | 0.242295073001731 | 1.3065455868756 | 0.00321199143468951 | TFAP2A//EGR2//DLL1// |
| GO:0050884 | neuromuscular process controlling posture | Biological process | 3 | 16 | 934 | 17653 | 3.54383029978587 | 0.0493690094035761 | 0.242295073001731 | 1.3065455868756 | 0.00321199143468951 | POU4F1//GLRA1//SCN1A// |
| GO:0060850 | regulation of transcription involved in cell fate commitment | Biological process | 3 | 16 | 934 | 17653 | 3.54383029978587 | 0.0493690094035761 | 0.242295073001731 | 1.3065455868756 | 0.00321199143468951 | SOX17//CEBPB//PPARG// |
| GO:0070262 | peptidyl-serine dephosphorylation | Biological process | 3 | 16 | 934 | 17653 | 3.54383029978587 | 0.0493690094035761 | 0.242295073001731 | 1.3065455868756 | 0.00321199143468951 | NCK1//PPP2CA//PPP1R15B// |
| GO:0071786 | endoplasmic reticulum tubular network organization | Biological process | 3 | 16 | 934 | 17653 | 3.54383029978587 | 0.0493690094035761 | 0.242295073001731 | 1.3065455868756 | 0.00321199143468951 | RTN3//ARL6IP1//RAB3GAP1// |
| GO:0072224 | metanephric glomerulus development | Biological process | 3 | 16 | 934 | 17653 | 3.54383029978587 | 0.0493690094035761 | 0.242295073001731 | 1.3065455868756 | 0.00321199143468951 | OSR1//TCF21//LHX1// |
| GO:1903392 | negative regulation of adherens junction organization | Biological process | 3 | 16 | 934 | 17653 | 3.54383029978587 | 0.0493690094035761 | 0.242295073001731 | 1.3065455868756 | 0.00321199143468951 | DLC1//ARHGAP6//PTEN// |
| GO:1905314 | semi-lunar valve development | Biological process | 3 | 16 | 934 | 17653 | 3.54383029978587 | 0.0493690094035761 | 0.242295073001731 | 1.3065455868756 | 0.00321199143468951 | EFNA1//TGFB2//BMPR2// |
| GO:0031214 | biomineral tissue development | Biological process | 12 | 133 | 934 | 17653 | 1.7053017983932 | 0.049456690665123 | 0.242549382863109 | 1.30577494729457 | 0.012847965738758 | ASPN//PTHLH//KLF10//PKDCC//S1PR1//GJA1//OSR1//FAM20C//BMPR2//TFAP2A//ACVR1//IGF1// |
| GO:0032868 | response to insulin | Biological process | 20 | 254 | 934 | 17653 | 1.48822269807281 | 0.0496600822172503 | 0.243370388431351 | 1.30399256576276 | 0.0214132762312634 | GAB1//APPL1//GRB2//SOS1//PIK3R3//CPEB2//INHBB//ERRFI1//PPARG//PTEN//CPEB1//SLC2A4//YWHAG//KAT2B//SESN3//NCK1//TSC1//IGF1//PRKAA1//EGR2// |
